# Comparative Analysis on Single- and Multiherb Strategies in Coronary Artery Atherosclerosis Therapy

**DOI:** 10.1155/2021/6621925

**Published:** 2021-04-29

**Authors:** Yu Cao, Yang Liu, Tian Zhang, Wei Lei, Boli Zhang

**Affiliations:** ^1^Institute of Traditional Chinese Medicine, Tianjin University of Traditional Chinese Medicine, No. 10 Poyanghu Road, Tianjin 301617, China; ^2^School of Chemical Engineering and Technology, Tianjin University, No. 135 Yaguan Road, Tianjin 300350, China; ^3^State Key Laboratory of Dao-di Herbs, National Resource Center for Chinese Materia Medica, China Academy of Chinese Medical Sciences, No. 16 Neinan Street, Beijing 100700, China

## Abstract

Herbal medicine unswervingly serves human health by modernizing preparation and administration. Coronary artery atherosclerosis is a serious threat to human health and survival all over the world. Following experimental and clinical evidence, we collected four herbal treatments containing herbal strategy I (San Qi), II (Injectio Salvia Miltiorrhizae), III (Danhong injection), and IV (Taoren Honghua Jian granule) against coronary artery disease. In order to analyze their similarities and differences in controlling coronary artery atherosclerosis, we investigated each herb of four strategies and revealed that the number of active components and molecule targets is increasing with the herb category of herbal strategy. Nitric oxide-associated carbonate dehydratase activity and nitrogen metabolism are tacitly enriched by target corresponding genes with statistical significance in four strategies. The herbal strategy with multiherb not merely possesses more amounts and interactions of target proteins than the strategy with single-herb but also enlarges interaction partners of target proteins like PTPN11 and STAT3 in strategy II, III, and IV. Whereas single-herb also involves regulating network core proteins in consistent with compatibility, such as SRC and PIK3R1 that are mostly targeted by strategy I, III, and IV. Comparing the targets of the herbal strategies and three existing drugs (atenolol, pravastatin and propranolol) and the symbols of coronary artery atherosclerosis, we discovered that MAOA, HTR1A, and ABCG2 are overlapping in the three groups. Hence, our work enables people to better understand the connections and distinctions of single- and multiherb on the healing of coronary artery atherosclerosis.

## 1. Introduction

As a treasure of culture, herbal medicine is an integral branch of Traditional Chinese Medicine (TCM) and reliably serves human health by modernizing preparation and administration in China for thousands of years [1]. Since the recognized therapeutic properties of herbs, herbal medicine obtains growing acceptance from the public health and medical field under many situations in western countries along with China, Japan, Korea, and several Southeast Asian countries [2].

Coronary atherosclerotic disease (CAD), a global issue, exists with high morbidity and mortality in human disease, which is triggered by coronary artery atherosclerosis (CAA) with stenosis or occlusion of the vessels as well as myocardial ischemia [3]. Atherosclerosis results from lipid metabolism disorder and following inflammatory actions that involve the accumulation of modified low-density lipoproteins like oxidized low density lipoproteins (ox-LDL) with loaded cholesterol, vascular endothelium damage, recruitment of monocytes into the subendothelial space, differentiated macrophages developing to foam cells elicited by dysregulated uptake of the ox-LDL, the appearance of fatty streaks in the intima of arterial walls, and formation of atherosclerotic plaques [4].

Plenty of studies over the past couple of decades have reported that herbs contribute to positive responses to atherosclerosis-associated disease. Atractylenolide I, II, and III are the essential bioactive constituents of Rhizoma Atractylodis Macrocephalae (*Atractylodes Macrocephala Koidz*.) [5]. Atractylenolide II and III weaken platelet aggregation caused by an agonist, lessen platelet extending on immobilized fibrinogen, and postpone clot retraction in platelet-depleted plasma [5]. *Terminalia arjuna* is a medicinal plant, and the uptake of its encapsulated plant extract appreciably reduces rat body weight compared to high cholesterol fed Wistar rats [6]. Also, in the high cholesterol fed group, serum lipids such as total cholesterol, very-low-density lipoprotein cholesterol, low-density lipoprotein cholesterol, triglycerides, and atherogenic index are uncovered with an obvious decrease under exerting encapsulated extract [6]. Astragaloside IV, as the major active ingredient of the herb *Astragalus membranaceus* is conducive to raising nitric oxide content in a concentration-dependent manner [7]. Besides, Astragaloside IV plays a vasodilator role on the aortic rings and dramatically promotes eNOS mRNA expression and release, leading to the vasodilator response by PI3K/Akt/eNOS signaling pathway [7].

At present, the advancement model of one-drug and one-target for disease therapy has attained enormous success. However, in many cases, certain disease like cancer is hard to be eradicated via therapy targeting a single gene, protein, or pathway. Along with multiple targets, the pharmacological actions of drugs have superior curative outcomes [8]. Hence network pharmacology emerged, developed, and is being exploited. For seeking molecule targets of drugs or pharmaceutical compounds, network pharmacology is mostly invoked to analyze the correlation between drugs, targets, and diseases in biological networks, with potential mechanisms on the drugs demolishing the bionetworks of disease [9]. The emphasis of network pharmacology focuses on not merely examining the role of single target or few targets but also the balance of network and its perturbations.

Although abundant achievements support herbs effectiveness on atherosclerosis, the differences of clinical herbal strategies have been seldom pursued especially against coronary artery atherosclerosis. Herein by using open data services, we designed a project to compare the chemical compositions, molecular targets, or mechanisms of diverse herbal strategies coupled with treatment of coronary atherosclerotic disease and attempted to interpret associations and diversities of molecular actions of single- and multiherb strategies against coronary artery atherosclerosis.

## 2. Materials and Methods

### 2.1. Candidate Herb Search

To find out a number of herbal medicines that have been investigated for atherosclerosis-resistant activity in coronary artery disease with human trials, information search was conducted by using ScienceDirect, Web of Science, and PubMed, all of which are free search engines for biomedical literature. The keywords for the search referred to combinations of the following terms: coronary artery atherosclerosis, herb, and clinical. Literatures published in the last fifteen years were considered, and single- and multiherb strategies including clinical and experimental evidence were further collected.

### 2.2. Herbal Component Screening

Traditional Chinese Medicine Systems Pharmacology Database and Analysis Platform (TCMSP) (https://tcmspw.com/tcmsp.php) is constructed to explore herbal medicines, taking the framework of systems pharmacology as a basis and providing twelve important properties linked with herbal absorption, distribution, metabolism, and excretion [10]. In Chemistry Database (http://www.organchem.csdb.cn), an archive of traditional Chinese medicine and chemical components shows details of diverse herbs, such as morphology and active element. PubChem (https://pubchem.ncbi.nlm.nih.gov) is a public repository and exhibits chemical structures, safety and toxicity data of small and larger molecules for instance, which has developed into the pivotal information resource involving chemical biology investigation and drug discovery [11]. Integrated with the above three platforms, the effective components were selected in light of the benchmarks of parameter OB (oral bioavailability) ≥30% and DL (drug-likeness) ≥0.18, and consistent Pubchem Cid or InChIKey.

### 2.3. Target Prediction

SwissTargetPrediction (http://www.swisstargetprediction.ch), an analysis implement on web, runs ligand-based target prediction for any small molecule with bioactivity and serves for one hundred and fifty-nine countries over the whole world [12], which exact target prediction is achieved through merging diverse measures of chemical similarity in line with both molecular shape and chemical structure [13]. The targets of component were considered credible and available when the probability value was 1.

### 2.4. Enrichment of Targets Corresponding Genes

The Database for Annotation, Visualization and Integrated Discovery (DAVID) 6.8 (https://david.ncifcrf.gov) is a web-based portal engaged to prepare a comprehensive gene annotation and analysis resource for biological scientists [14]. Gene Ontology (GO) and Kyoto Encyclopedia of Genes and Genomes (KEGG) pathway enrichment analyses of targets corresponding genes were carried out by DAVID. False Discovery Rate (FDR) <0.05 was defined as statistical significance.

### 2.5. Functional Protein Network

STRING 11.0 (http://string-db.org) focuses on a quantity of organisms and consolidates predicted and known protein-protein correlation data [15]. The correlation contains direct (physical) and indirect (functional) interactions in STRING database, wherein the two interaction categories are specific and biologically meaningful. “Experiments” and “Databases” were ticked as active interaction sources in options. The minimum required interaction score was set as highest confidence (0.900). In different species, sequence conservation was detected under best-hit similarity only (the color intensity denotes the degree of similarity) which similarity level was chosen as meaning of the conservation symbols. Similarity scale represents “no detectable similarity” to “100% sequence conservation” with increasing color depth.

### 2.6. Coronary Artery Atherosclerosis-Centric Drug

DrugBank (https://www.drugbank.ca) is a facilitated online database covering comprehensive molecular information about drugs and their targets, interactions, and mechanisms [16]. A great deal of data in DrugBank encompasses enormous details of drugs effect on the levels of gene expression (pharmacotranscriptomics), protein expression (pharmacoprotoemics), and metabolite (pharmacometabolomics), and the status of accessible drug repurposing trials and new drug clinical trials. Coronary artery atherosclerosis-centric drugs and their targets were recorded by seeking indications.

### 2.7. Coronary Artery Atherosclerosis-Centric Symbol

GeneCards (https://www.genecards.org) is an integrative and searchable database providing comprehensive, authoritative compendium of annotative information about human genes. The knowledge database integrates gene-related data from nearly one hundred and fifty web sources, embodying genomic, transcriptomic, proteomic, genetic, clinical, and functional information [17]. Coronary artery atherosclerosis was input as the content of keywords and identifiers, and the disease symbols were recorded subsequently.

## 3. Results

### 3.1. Herbal Strategy Sifting

**Figure 1:**
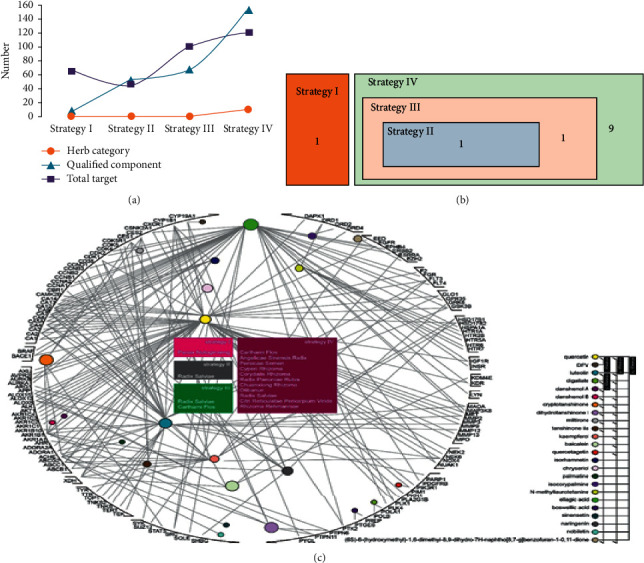
Four herbal strategies. (a) Number and category of herbs and chemical components and targets of each herbal strategy. (b) Number of common or unique herb in four herbal strategies. (c) Correspondence between component and target in herbal strategy.

Four herbal strategies containing San Qi (*Panax Notoginseng*) as strategy I, Injectio Salvia Miltiorrhizae (*Radix Salviae*) as strategy II, Danhong injection (*Radix Salviae* and *Carthami Flos*) as strategy III, and Taoren Honghua Jian granule (*Persicae Semen*, *Carthami Flos*, *Angelicae Sinensis Radix*, *Cyperi Rhizoma*, *Corydalis Rhizoma*, *Radix Paeoniae Rubra*, *Chuanxiong Rhizoma*, *Olibanun*, *Radix Salviae*, *Citri Reticulatae Pericarpium Viride,* and *Rhizoma Rehmanniae glutinosae*) as strategy IV (Figure 1(a)) were considered to be eligible treatment approach for coronary artery atherosclerosis by literature retrieval [18–21]. Latin name of eleven herbs was checked and in accord with description of TCMSP except that *Rhizoma Rehmanniae glutinosa* was inconsistent with Chemistry Database. San Qi (strategy I) modulates signaling pathways relevant to lipid metabolism, inflammation, atherosclerosis, and myocardial ischemia and shares their superiority on coronary artery disease as a novel option [18]. Injectio Salvia Miltiorrhizae (strategy II) restrains platelet aggregation and serotonin release triggered by either epinephrine or ADP in a dose-dependent manner with curative effect in experimental and clinical coronary artery disease [19]. Danhong injection (strategy III) is mostly utilized in the clinical therapy of acute coronary syndrome and angina pectoris in China [20]. Taoren Honghua Jian granule (strategy IV) alleviates symptoms and controls inflammation in patients with stable coronary artery disease [21]. *Radix Salviae* takes part in strategies II, III, IV, as well as *Carthami Flos* which is used in strategies III and IV (Figure 1(b)).

### 3.2. Intrinsic Effective Ingredients of Herbs

In conjunction with TCMSP, Chemistry Database and PubChem, we separately screened out 8, 65, 84 and 207 bioactive elements in strategies I, II, III, and IV by preset parameters (Table S1). Considering unique Pubchem Cid or InChIKey, we further narrowed above elements into 8, 51, 68, and 154 ingredients as candidates in strategy I to IV in turn, respectively (Figure 1(a); Table S1 (except yellow highlighted)). There are common 1 ingredient (mandenol) between strategies I and IV, 14 (e.g. 7,8-dimethyl-1H-pyrimido[5,6-g]quinoxaline-2,4-dione and 6-Hydroxynaringenin) between strategies III and IV, 3 (e.g. quercetin and stigmasterol) between strategies I, III, and IV, and 51 (e.g. danshenol A and neocryptotanshinone) between strategies II, III, and IV, with 4 (e.g. ginsenoside rh2 and diop) and 85 (e.g. spinasterol and GA54) specific ingredients severally in strategy I and strategy IV (Table S2).

### 3.3. Targets of Herbal Constituents

**Figure 2:**
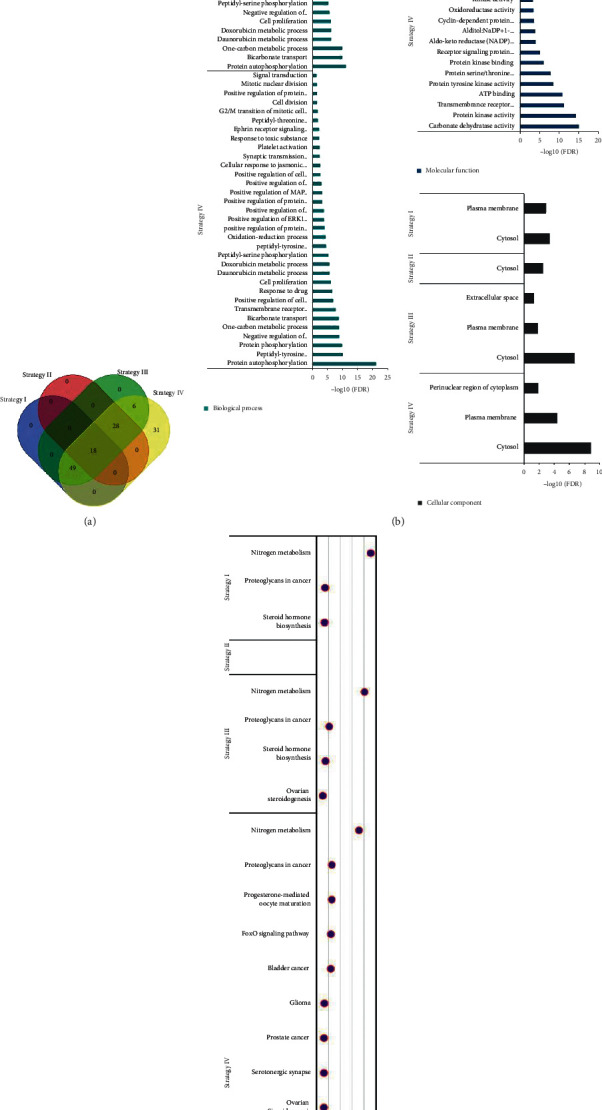
Statistical and enrichment analyses of herbal targets. (a) Number of common or unique targets in diverse herbal strategy. (b, c) -log10(FDR) values of recruited (b) biological processes, molecular functions, cellular components, and (c) pathways in each herbal strategy.

Using SwissTargetPrediction, we predicted total 67, 46, 101, and 132 targets (with fixed 100% probability) responding to partial candidate ingredients of herbal strategy I to IV in order (Figure 1(c); Tables S3 and S4). For strategy I, both quercetin and DFV are aiming to CYP19A1 (Table S5). Luteolin, digallate, danshenol A, danshenol B, cryptotanshinone, dihydrotanshinone I, miltirone, tanshinone iia, and (6S)-6-(hydroxymethyl)-1,6-dimethyl-8,9-dihydro-7H-naphtho[8,7-g]benzofuran-10,11-dione provide qualified targets in strategy II while AKR1B1, ACHE, CES1, CES2, PTPN6 and PTPN11 are pursued by more than one component (Table S5). Likewise, there are 13 components (e.g., luteolin and quercetin) and their 29 repetitive targets (NOX4, AKR1B1, XDH, MAOA, FLT3, CA2, ALOX5, ADORA1, CA7, GLO1, GSK3B, MMP9, CA12, MMP2, CA4, CYP1B1, ABCG2, ACHE, CES1, CES2, PTPN6, PTPN11, PIM1, HSD17B2, ALOX15, ABCC1, CDK1, ALOX12, ABCB1) in strategy III, and 23 components (e.g. isorhamnetin and ellagic acid) with corresponding 50 common targets (AKR1B1, CYP1B1, CA12, CA7, CA2, XDH, CA4, ABCG2, CES1, CES2, ABCC1, ALOX5, NOX4, FLT3, GSK3B, CDK1, ACHE, MMP2, GLO1, MAOA, ADORA1, MMP9, PTPN11, PTPN6, PIM1, HSD17B2, ABCB1, AURKB, PTK2, NUAK1, KDR, CA13, IGF1R, MET, CA1, CA6, BACE1, CSNK2A1, AKT1, CA14, CA9, PLK1, EGFR, SRC, CA5A, ALOX12, ALOX15, CYP19A1, HSD17B1, DRD1) in strategy IV (Table S5). Several targets are shared between different herbal strategies, especially the 18 targets (e.g., CDK1 and MAOA) in four strategies, in addition to 31 unique targets (e.g., CDK2 and HTR1A) in strategy IV (Figure 2(a); Table S6).

### 3.4. Enrichment Analyses on Bioprocess

Targets corresponding genes applied DAVID on enrichment analysis of GO term and KEGG pathway. The total terms are significantly (FDR <0.05) recruited to 14 (e.g., negative regulation of apoptotic process and oxidation-reduction process), 3 (e.g., peptidyl-threonine phosphorylation and cellular response to hydrogen peroxide), 20 (e.g., one-carbon metabolic process and platelet activation), and 33 (e.g., bicarbonate transport and positive regulation of ERK1 and ERK2 cascade) biological processes (Figure 2(b); Table S7), 12 (e.g., carbonate dehydratase activity and alditol: NADP+ 1-oxidoreductase activity), 3 (e.g., protein kinase binding and cyclin-dependent protein serine/threonine kinase activity), 13 (e.g., aldo-keto reductase (NADP) activity and oxidoreductase activity), and 19 (e.g., ATP binding and protein serine/threonine kinase activity) molecular functions (Figure 2(b); Table S7), 2 (cytosol and plasma membrane), 1 (cytosol), 3 (e.g., plasma membrane and extracellular space), and 3 (e.g., cytosol and perinuclear region of cytoplasm) cellular components in strategy I to IV sequentially (Figure 2(b); Table S7). Three (e.g., nitrogen metabolism and steroid hormone biosynthesis), 0, 4 (e.g., steroid hormone biosynthesis and ovarian steroidogenesis), and 15 (e.g., Focal adhesion and Rap1 signaling pathway) KEGG pathways are independently enriched with statistical significance (FDR <0.05) (Figure 2(c); Table S7).

### 3.5. Protein-Protein Interaction Prediction and Nodal Protein Identification

**Figure 3:**
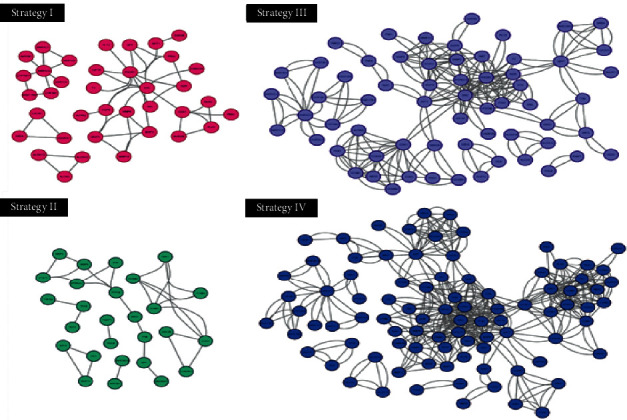
Potential protein-protein interaction networks in four herbal strategies with clarified clusters.

**Figure 4:**
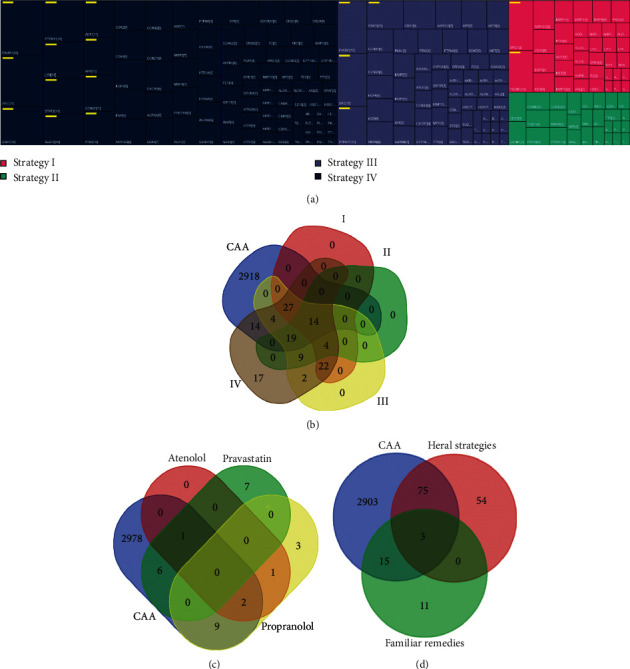
Protein network member and target amount. (a) Member information in interaction systems. Number of interaction partner is proportional to shape size and marked in square brackets. Yellow bar sign indicates that the number of interaction partner is greater than or equal to 10. (b–d) Number of common or unique targets comparing coronary artery atherosclerosis to herbal strategy (I, II, III, IV) or/and familiar remedy (atenolol, pravastatin, propranolol). I, II, III, IV, and CAA signify severally strategy I, strategy II, strategy III, strategy IV, and coronary artery atherosclerosis.

Using STRING allowed by background resource of both experiments and databases, we clarified the potential protein-protein interaction (PPI) with highest confidence that PPI score is greater than or equal to 0.9, based on the targets of four herbal strategies. The current PPI networks outstood 4, 6, 5, and 5 clusters in strategies I, II, III, and IV, respectively (Figure 3; Table S8). In order to probe whether attractive targets exist in different herbal strategies, we calculated the number of interactive lines of every member in clusters. We then discovered that SRC and PIK3R1 as pivot have the high number of immediate partners in strategies I, III, and IV with CDK5 in strategy II (Figure 4(a); Table S9). Taking 10 dynamic partners per member as threshold, we revealed that PTPN11 and STAT3 are less interactive in strategy II but appear higher interactive degree in strategies III and IV (Figure 4(a); Table S9).

### 3.6. Correlation Analysis with Familiar Remedies and Disease Symbol

Atenolol (DB00335), pravastatin (DB00175), and propranolol (DB00571) are three familiar remedies coupled with coronary artery atherosclerosis treatment in DrugBank. Annotated 4 (e.g. ADRB1 and CYP2D6), 14 (e.g. HMGCR and SLCO2B1), and 15 (e.g. HTR1A and MAOA) objects are identified severally as targets of atenolol, pravastatin, and propranolol (Table S10). We found out 2996 CAA-centric symbols including 10 identifiers by GeneCards (Table S11). Comparing to CAA-centric symbols, there are individually 41 (e.g. MMP2 and XDH), 33 (e.g. CCNB1 and CD38), 64 (e.g. CD38 and MAOA), and 78 (e.g. MET and TEK) common objects in targets of herbal strategies I, II, III, and IV (Figure 4(b); Table S12), and 3 (e.g. ADRB1 and ABCB11), 7 (e.g. ABCC2 and SLCO1B1), and 11 (e.g. CYP2C19 and MAOA) objects overlapped in targets of atenolol, pravastatin and propranolol (Figure 4(c); Table S12). Notably, MAOA is common target in four herbal strategies and propranolol, with HTR1A in strategy IV and propranolol, and ABCG2 in four herbal strategies and pravastatin, and in coronary artery atherosclerosis (Figure 4(d); Table S12). Neither four herbal strategies nor three familiar remedies expose common targets matched with CAA-centric 10 identifiers.

## 4. Discussion

The four therapy schemes filled by herbal strategy I (*Panax Notoginseng*), strategy II (*Radix Salviae*), strategy III (*Radix Salviae* and *Carthami Flos*), and strategy IV (*Persicae Semen*, *Carthami Flos*, *Angelicae Sinensis Radix*, *Cyperi Rhizoma*, *Corydalis Rhizoma*, *Radix Paeoniae Rubra*, *Chuanxiong Rhizoma*, *Olibanun*, *Radix Salviae*, *Citri Reticulatae Pericarpium Viride,* and *Rhizoma Rehmanniae glutinosae*) have been confirmed to be able to improve conditions of diseases related to coronary artery atherosclerosis in previous works [18–21]. Moreover, ischemic heart disease is conventionally tantamount to flow-limiting obstruction-induced one or more atherosclerotic plaques in large-medium sized coronary arteries, but recently Severino et al. put forward a new explanation that myocardial ischemia is immediately decided by the loss of cross talk between coronary (such as coronary microvessel) blood flow and myocardial energy state [22]. Severino et al. profiled that coronary microvascular dysfunction triggered by coronary blood flow regulatory disequilibrium, including ion channels, breaks myocardial metabolic demands supplied from coronary circulation and brings about hypoxia, fibrosis and tissue death, perhaps further impairs myocardial function [23]. In Eastern countries, *Panax Notoginseng* is recognized as a therapeutic herb that has clinical efficacy in hypertension and myocardial ischemia [24]. There is evidence that *Panax Notoginseng* saponins can individually regulate apoptosis and mitochondrial autophagy by the PI3K/Akt and HIF-1*α*/BNIP3 pathways to protect rat heart from myocardial ischemia and its reperfusion injury [25, 26]. Ginsenosides is devoted to diminishing energy metabolism disturbance, oxidative stress, inflammation response, and cardiomyocyte apoptosis [27, 28]. *Radix Salviae* water extract inhibits the tumor necrosis factor (proinflammatory cytokine) cascade downstreamed mitogen-activated protein kinase signaling pathway with restricted extracellular signal-regulated protein kinases and Jun N-terminal kinase phosphorylation against inflammation caused by Toll-like receptor 2 [29]. Magnesium lithospermate B is a bioactive component of *Radix Salviae*. It disables NF-*κ*B via PKC- and PI3K/Akt-mediated Nrf2 activation in human dermal microvascular endothelial cells (HMEC-1), preventing lipopolysaccharide-evoked endothelial dysfunction from acute inflammation [30]. Salvianolate as medicinal product of *Radix Salviae* is implicated in alleviated oxidative stress and apoptosis and has palpably positive avail on myocardial microvascular reflow by injection [31]. *Carthami Flos* intervention is credited as an herb therapeutics for cardiovascular disease since ameliorating blood circulation, and the effects of *Angelicae Sinensis Radix*, *Chuanxiong Rhizoma* and *Radix Salviae* on cardiac microvascular endothelial cells, and *Carthami Flos* whole extract enhances HMEC-1 cells proliferation [32, 33].

Relying on TCMSP, Chemistry Database and PubChem combinations, we selected 8, 51, 68, and 154 active ingredients from herbal strategy I to IV in turn with consistent Pubchem Cid or InChIKey. Since herb *Radix Salviae* is mutually described in strategies II, III, and IV, with more amount of chemical compound shared, 51 components are comprised in all the three herbal strategies. There are 3 common components: (1) beta-sitosterol in *Panax Notoginseng*, *Carthami Flos*, *Angelicae Sinensis Radix*, *Persicae Semen*, *Cyperi Rhizoma* and *Radix Paeoniae Rubra*; (2) stigmasterol in *Panax Notoginseng*, *Carthami Flos*, *Angelicae Sinensis Radix*, *Cyperi Rhizoma*, *Corydalis Rhizoma* and *Radix Paeoniae Rubra*; and (3) quercetin in *Panax Notoginseng*, *Carthami Flos*, *Cyperi Rhizoma* and *Corydalis Rhizoma* by comparing strategy I to strategy III and IV; and 1 common component of mandenol in *Panax Notoginseng* and *Chuanxiong Rhizoma* by comparing specific herbs of the four strategies. Analogous to cholesterol structure, beta-sitosterol as a plant-derived compound is generally utilized to treat hypercholesterolemia and coronary artery disease [34]. Besides beta-sitosterol, stigmasterol belongs to phytosterol that is in favor of reducing heart attack risk via lowering cholesterol levels in a natural way [35]. Quercetin is a main flavonoid. The increased levels of tumor necrosis factor, interleukin-1*β,* and interleukin-10 are manifested in the serum of patients with coronary artery disease; nevertheless, the levels of the above three cytokines are declined under the impact of quercetin [36]. The anti-inflammatory properties of quercetin also reflect in blocking gene expression of the inhibitor of kappa B *α* in patients with coronary artery disease [36]. Mandenol, also known as ethyl linoleate, is an unsaturated fatty acid used in diminishing inflammation. The Yam extracts embodying beta-sitosterol and ethyl linoleate obviously reduce the total atherosclerotic lesion area in the aortic root of apolipoprotein E-deficient mice with Western-type diet [37]. Four and 85 exclusive components were severally detected in herbal strategies I and IV. For instance, DFV also named as liquiritigenin is specifically applied in strategy I as well as unique naringenin in strategy IV. Mice experiments note that liquiritigenin treatment markedly decreases the concentrations of proinflammatory cytokines like interleukin-6, interleukin-1*β,* and tumor necrosis factor-*α* in serum and hippocampus and increases levels of superoxide dismutase, catalase, and glutathione [38]. Naringenin is a flavonoid usually consumed by humans that possesses anti-inflammatory, hypolipidemic, antithrombotic, and antiatherogenic activity. In pressure-overloaded mice by performing aortic banding, naringenin reverses left ventricular function dysfunction with attenuation of cardiac hypertrophy and interstitial fibrosis, while its cardioprotective effect is focused on restraining c-Jun N-terminal kinase, extracellular signal-regulated kinase, and phosphoinositide 3-kinase/protein kinase B signaling pathways [39]. This shows that the number of herbal active constituents may increase with the categories of herbs in CAA-associated herbal strategies, in which constituents could be common or unique for promoting recovery from CAA.

**Figure 5:**
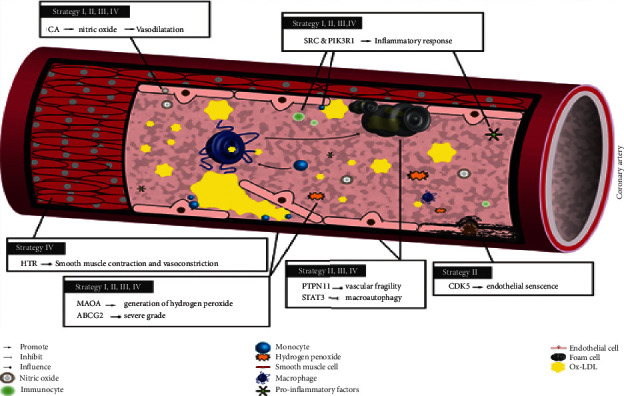
Active molecule targets and bioactions of four herbal strategies against coronary artery atherosclerosis.

Based on these active components, we predicted total 67, 46, 101, and 132 targets of herbal strategy I to IV in turn by SwissTargetPrediction with 100% probability of screening criteria. We uncovered that the shared target is emphasized in not only diverse herbal strategies but also different active components; for example, CDK1 is directed by luteolin, quercetin, and baicalein in all herbal strategies. Another interesting finding is that most of molecular functions of targets are constantly invoked in diverse strategies using GO enrichment analysis, with unique GO terms like electron carrier activity in strategy I and protein binding, protein phosphatase binding, and enzyme binding in strategy IV. As a common molecular function term enriched in four herbal strategies, carbonate dehydratase activity is significantly recruited by targets relevant to members of carbonate dehydratase (CA) family (CA1, CA2, CA3, CA4, CA5A, CA6, CA7, CA9, CA12, CA13, and CA14) in part or whole. The interconversion between carbon dioxide and bicarbonate is an indispensable physiological reaction catalyzed by carbonate dehydratase (also carbonic anhydrase), such as on respiration, pH homeostasis, secretion of electrolytes, and carboxylation reactions for biological survival [40]. Nitric oxide is a potent endogenous vasodilator, and its impaired synthesis and weakened bioavailability in the endothelium and the circulation, respectively, are recognized to be primary contributors to the development and progression of cardiovascular diseases [41]. Carbonate dehydratase participates in transport inorganic nitrite reserving abundant nitric oxide in tissues and cells [41]. Besides, nitrogen metabolism as most significant pathway is enriched by targets in accord with members of carbonate dehydratase family in strategies I, III, IV (FDR <0.001), and strategy II (*p*=0.000092). We noticed that 5-hydroxytryptamine receptors (HTRs) containing HTR1A, HTR2B, HTR5A, HTR6, and HTR7 are specific targets of herbal strategy IV, of which the 5 members of 5-hydroxytryptamine receptor family are enriched to vasoconstriction (FDR = 0.0058) and smooth muscle contraction (*p*=0.0043). In terms of reversing situation of coronary artery atherosclerosis, these herbal strategies availability may be reflected on mediating uniformly carbonate dehydratase activity and nitrogen metabolism pathway by carbonate dehydratase family members, and regulating partly vasoconstriction and smooth muscle contraction by 5-hydroxytryptamine receptor family members (Figure 5).

With highest confidence, the potential protein-protein interaction was illustrated on targets of each herbal strategy by using STRING. We distinguished 4, 6, 5, and 5 clusters in PPI networks of strategy I to IV consecutively, further the cluster engaging maximum amount of inner member was chose as core to analyze. As the result shown, SRC and PIK3R1 were screened out and considered as core target, because both of them attract more interaction partners in the strategies I, III, IV. In the present work, proto-oncogene tyrosine-protein kinase SRC encoded by *SRC* and phosphoinositide-3-kinase regulatory subunit 1 encoded by *PIK3R1* were identified as targets of quercetin in all herbal strategies. PIK3R1 constitutes PI3K regulatory subunit alpha. Prior researches demonstrated that quercetin arrests SRC and PI3K activation in a dose- and time-dependent manner [42], while quercetin consumption suppresses inflammatory response by downregulating SRC/PI3K/Akt-NF-*κ*B-inflammatory pathways and inhibits the progression of atherosclerosis in ApoE-null mice [43]. In addition, cyclin dependent kinase 5 encoded by *CDK5* is a member of cyclin dependent kinase family, while it is targeted by luteolin and most interactive in herbal strategy II. CDK5 facilitates the development of endothelial senescence and atherosclerosis by mediating hyperphosphorylation of sirtuin-1, and its knockdown or inhibition decreases the number of senescent endothelial cells and attenuates inflammatory genes expression in porcine aortic endothelial cells [44], but the flavonoids like luteolin can inactivate CDK5 [45]. PTPN11 and STAT3 are targeted by certain chemical compounds (such as tanshinone IIA and cryptotanshinone) in herbal strategies without strategy I, while the interactive degrees of PTPN11 and STAT3 are higher in herbal strategies III and IV than herbal strategy II. Protein tyrosine phosphatase nonreceptor type 11 corresponding *PTPN11* mutation induces diffuse bilateral dilatation of the coronary arteries and influences vascular fragility in the coronary artery, even if the patient shows no symptoms of ischemic heart disease [46, 47]. In ApoE mice fed by high-fat diet, tanshinone IIA treatment significantly increases high-density lipoprotein-cholesterol levels and decreases serum levels of total cholesterol, triglycerides, and low-density lipoprotein-cholesterol, as well as improving plaque size and lipid deposition [48]. Further, tanshinone IIA can stimulate both *PTPN11* and its encoded protein [49]. Foam cells are chiefly delivered from macrophages and herald atherosclerotic lesions, but autophagy can reduce foam cell formation against atherosclerosis [50]. However, cytoplasmic signal transducer and activator of transcription 3 is encoded by *STAT3* dampens macroautophagy by repressing EIF2AK2/PKR activity [51], while its gene expression is also increased dramatically in patients with coronary artery disease [52]. Previous study suggests that cryptotanshinone attenuates STAT3 expression and IL-6-mediated STAT3 activation [53]. These results suggest that herbs targeting SRC and PIK3R1 may play hub role for reversing the network balance disturbed by coronary artery atherosclerosis (Figure 5). Whereas *Radix Salviae* is involved in herbal strategies II, III, and IV, the interactive degrees of each common target like PTPN11 and STAT3 increase with the increment of herbal variety and target category in strategy (Figure 5).

Searching coronary artery atherosclerosis as indication in DrugBank, there are atenolol, pravastatin, and propranolol defined as curability, acting on 4, 14, and 15 annotated targets orderly. Atenolol is a synthetic beta-1 selective blocker and can diminish mortality following myocardial infarction in hemodynamically stable patients with care of hypertension and chronic angina. Treatment with atenolol is conducive to mitigating the risk of coronary artery disease, congestive heart failure, and stroke as well as morbidity and mortality of postmyocardial infarction [54]. Pravastatin belongs to HMG-CoA reductase inhibitor, in contrast to placebo, pravastatin utilization cuts down the concentration of low-density lipoprotein cholesterol and minimizes nonfatal myocardial infarction risk and coronary heart disease mortality [55]. As a nonselective beta-adrenergic antagonist, propranolol can improve poor healthy situations like hypertrophic subaortic stenosis, hypertension, and angina. Past investigation deciphered that propranolol is considered to act the relaxant effect partly by the Ca^2+^-activated K^+^ channels [56], while the antiatherogenic effect of propranolol serves as protection in some extent against CAA among individuals for the susceptibility to coronary heart disease, independently of the influences of serum lipid concentrations, blood pressure, and resting heart rate [57]. Coronary artery atherosclerosis exists with 2996 symbols including 10 identifiers in GeneCards. Comparing the targets of four herbal strategies and three familiar drugs, and the symbols of coronary artery atherosclerosis, we unveiled popular MAOA targeted by four herbal strategies and propranolol, HTR1A aimed by herbal strategy IV and propranolol, and ABCG2 directed by all herbal strategies and pravastatin, besides acting as disease symbols. Monoamine oxidase A encoded by *MAOA*, a member of monoamine oxidases, is mitochondrial enzyme and promotes cardiovascular oxidative stress via constant generation of hydrogen peroxide [58]. MAOA inhibitor appreciably attenuates constant impairment of endothelial-dependent relaxation in patients with coronary heart disease aside from in human mammary arteries [58]. In the investigation of three white subjects, ATP binding cassette subfamily G member 2 encoded by *ABCG2* is associated with severe coronary artery disease under the influence of single-nucleotide polymorphisms [59]. Herbal quercetin and luteolin were predicted to target MAOA and ABCG2, and the two compounds have been severally evidenced on well inhibiting enzyme MAOA [60] and depressing vasculogenic mimicry and lowering the expression of ABCG2 [61]. There is a report indicating that 5-hydroxytryptamine receptor 1A encoded by *HTR1A* is recognized to correlate cardiometabolic diseases risk and mood disorders [62]. Regretfully, what is still lacking here is an investigation of definitive correlation between N-methyllaurotetanine and its underlying target HTR1A. The atherosclerosis risk factors, such as hypertension and hyperlipidemia, fuel the formation of atherosclerotic lesions containing low-density lipoprotein oxidation, inflammatory process, endothelial dysfunction, vascular smooth muscle cell proliferation, and platelet aggregation [63]. Taking diabetes mellitus for example, it is one of the decisive atherosclerosis risk factors for ischemic heart disease. In diabetic patients, the existing pathophysiology of myocardial ischemia embodies the following two aspects: (1) coronary stenosis blocking blood flow to the myocardium; (2) coronary microvascular disease that caused by ion channels abnormality but without plaques in the epicardial vessels [64]. With oxidant increment and antioxidant absence, oxidative stress (also known as redox state disorder) is identified as downstream characteristic of certain myocardial metabolic products being considered to disturb ion channel function [65]. Thereby the upregulated antioxidant activity encourages a positive response for oxidative stress and further reverses the adverse outcomes drove by atherosclerosis risk factor in myocardial ischemia. The invaluable evidence correlating herbal therapy and familiar drug indicates that active Salvianolic acid B of *Radix Salviae* confers antimyocardial ischemia effect primarily by lowering the concentration of cyclic adenosine monophosphate and Ca^2+^ and depressing protein kinase A and shows similar metabolomic profiles with propranolol [66], which signifies a prospective synergic effect of *Radix Salviae* and propranolol. Yet, in terms of heart function, the protective effect of *Angelicae Sinensis Radix* is not influenced by propranolol [67]. It is important to highlight that the majority of common targets are presented between coronary artery atherosclerosis and herbal strategy filled by complex herbs, separately comparing herbal strategy (single herb or complex herbs) or familiar drug to coronary artery atherosclerosis. These discoveries indicate that besides overlapping therapeutic effect with familiar drugs by targeting MAOA/HTR1A/ABCG2 (Figure 5), herbal strategy may retain superiority on coronary artery atherosclerosis-related therapy through regulating disease symbols extensively, especially herbal strategy application with multiple herbs. Likewise, CA1, CA2, CA3 of carbonate dehydratase family enriched in carbonate dehydratase activity and nitrogen metabolism, and SRC, PIK3R1, CDK5, PTPN11, STAT3 in PPI network were identified as CAA-centric symbols. These assume that herbal strategy maximum covering appropriate family members, interactive cores, and CAA-centric symbols may be distinctive for improving patients with coronary artery atherosclerosis.

## 5. Conclusions

In the present study, we collected four herbal strategies coupled with coronary artery atherosclerosis treatment. In the light of evaluation methods with ingredient screening, target predicting and annotating and enriching, we unveiled that the four herbal strategies possess common chemical components and molecule targets, and significantly regulate consistent bioactions like carbonate dehydratase activity as well as nitrogen metabolism, furtherly reaching their availability against coronary artery atherosclerosis. Herbal strategy with multiherb underlines not merely more chemical compounds and targets, but also increasing interaction partners of target protein comparing to herbal strategy with single-herb. Single-herb scheme also can be like complex herbs to direct identical CAA-centric symbols, while the four herbal strategies target more CAA-centric symbols contrasting to existing drugs. We expect to promote understanding on single- and multiherb strategies serving to resist coronary artery atherosclerosis and provide inspirations on trial selection of drug targets at molecular level.

## 6. Study Limitations

The present study has several limitations. First, these known herbal components screened from TCMSP as active ones of medicament are governed by cognition status on herbs. Second, the details of the CAA-centric symbols and the symbol types showed in GeneCards are incomplete, which carry over into vague expression toward CAA. Third, there is a shortage of analysis for the correlation between each herbal strategy and overall survival in patients with CAA.

## Data Availability

All data analyzed during this study are included in this article.

## Abbreviations

CA: Carbonate dehydratase
CAA: Coronary artery atherosclerosis
CAD: Coronary atherosclerotic disease
DAVID: The database for annotation, visualization and integrated discovery
FDA: Food and drug administration
FDR: False discovery rate
DL: Drug-likeness
HTRs: 5-hydroxytryptamine receptors
GO: Gene ontology
KEGG: Kyoto encyclopedia of genes and genomes
OB: Oral bioavailability
ox-LDL: Oxidized low density lipoproteins
PPI: Potential protein-protein interaction
TCM: Traditional Chinese medicine
TCMSP: Traditional Chinese medicine systems pharmacology database and analysis platform.

## Supplementary Materials

Supplementary Materials Table S1: Active components in four herbal strategies. Table S2: Common and unique component in four herbal strategies. Table S3: Targets of different components in all herbal strategies. Table S4: Targets of each herbal strategy by deduplication. Table S5: Common targets of different components in herbal strategy I, II, III, and IV. Table S6: Common and unique target in four herbal strategies. Table S7: GO and KEGG pathway enrichment analyses of targets in four herbal strategies. Table S8: Score of potential protein-protein interactions. Table S9: Interaction count of targets. Table S10: Targets of three familiar drugs in DrugBank. Table S11: Symbols associated with coronary artery atherosclerosis in GeneCards. Table S12: Common and unique target in coronary artery atherosclerosis, herbal strategies, and familiar remedies.
