# Supplementary material for: Comparative Analysis on Single- and Multiherb Strategies in Coronary Artery Atherosclerosis Therapy
Source: Cardiol Res Pract. 2021 Apr 29;2021:6621925. doi: 10.1155/2021/6621925 (PMC8105113; doi:10.1155/2021/6621925)
Supplement: Supplementary Materials — Table S1: Active components in four herbal strategies. Table S2: Common and unique component in four herbal strategies. Table S3: Targets of different components in all herbal strategies. Table S4: Targets of each herbal strategy by deduplication. Table S5: Common targets of different components in herbal strategy I, II, III, and IV. Table S6: Common and unique target in four herbal strategies. Table S7: GO and KEGG pathway enrichment analyses of targets in four herbal strategies. Table S8: Score of potential protein-protein interactions. Table S9: Interaction count of targets. Table S10: Targets of three familiar drugs in DrugBank. Table S11: Symbols associated with coronary artery atherosclerosis in GeneCards. Table S12: Common and unique target in coronary artery atherosclerosis, herbal strategies, and familiar remedies. [file 6621925.f1.docx]

**Table S1.** Active components in four herbal strategies

| **Strategy** | **Herb Name** | **Mol ID** | **Molecule Name** | **Pubchem Cid** | **MW** | **AlogP** | **Hdon** | **Hacc** | **OB (%)** | **Caco-2** | **BBB** | **DL** | **FASA-** | **HL** |
| --- | --- | --- | --- | --- | --- | --- | --- | --- | --- | --- | --- | --- | --- | --- |
| I | *Panax Notoginseng* | MOL001494 | Mandenol | 5282184 | 308.56 | 6.99 | 0 | 2 | 42 | 1.46 | 1.14 | 0.19 | 0.25 | 5.39 |
|  |  | MOL001792 | DFV | 114829 | 256.27 | 2.57 | 2 | 4 | 32.76 | 0.51 | -0.29 | 0.18 | 0.42 | 17.89 |
|  |  | MOL002879 | Diop | 33934 | 390.62 | 7.44 | 0 | 4 | 43.59 | 0.79 | 0.26 | 0.39 | 0.28 | 3.6 |
|  |  | MOL000358 | beta-sitosterol | 222284 | 414.79 | 8.08 | 1 | 1 | 36.91 | 1.32 | 0.99 | 0.75 | 0.23 | 5.36 |
|  |  | MOL000449 | Stigmasterol | 5280794 | 412.77 | 7.64 | 1 | 1 | 43.83 | 1.44 | 1 | 0.76 | 0.22 | 5.57 |
|  |  | MOL005344 | ginsenoside rh2 | 119307 | 622.98 | 4.04 | 6 | 8 | 36.32 | -0.51 | -1.38 | 0.56 | 0.24 | 11.08 |
|  |  | MOL007475 | ginsenoside f2 | 131705287 | 785.14 | 2.3 | 9 | 13 | 36.43 | -1.8 | -3.03 | 0.25 | 0.22 | 13.11 |
|  |  | MOL000098 | quercetin | 5280343 | 302.25 | 1.5 | 5 | 7 | 46.43 | 0.05 | -0.77 | 0.28 | 0.38 | 14.4 |
| II/III/IV | *Radix Salviae* | MOL001659 | Poriferasterol | 5281330 | 412.77 | 7.64 | 1 | 1 | 43.83 | 1.44 | 1.03 | 0.76 | 0.22 | 5.34 |
|  |  | MOL001771 | poriferast-5-en-3beta-ol | 457801 | 414.79 | 8.08 | 1 | 1 | 36.91 | 1.45 | 1.14 | 0.75 | 0 | 5.07 |
|  |  | MOL001942 | isoimperatorin | 68081 | 270.3 | 3.65 | 0 | 4 | 45.46 | 0.97 | 0.66 | 0.23 | 0.27 | -1.44 |
|  |  | MOL002651 | Dehydrotanshinone II A | 128994 | 292.35 | 4.22 | 0 | 3 | 43.76 | 1.02 | 0.52 | 0.4 | 0.33 | 23.71 |
|  |  | MOL002776 | Baicalin | 64982 | 446.39 | 0.64 | 6 | 11 | 40.12 | -0.85 | -1.74 | 0.75 | 0.36 | 17.36 |
|  |  | MOL000569 | digallate | 341 | 322.24 | 1.53 | 6 | 9 | 61.85 | -0.76 | -1.52 | 0.26 | 0.43 | 5.29 |
|  |  | MOL000006 | luteolin | 5280445 | 286.25 | 2.07 | 4 | 6 | 36.16 | 0.19 | -0.84 | 0.25 | 0.39 | 15.94 |
|  |  | MOL006824 | α-amyrin | 12358389 | 426.8 | 7.35 | 1 | 1 | 39.51 | 1.37 | 1.2 | 0.76 | 0.23 | 3.06 |
|  |  | MOL007036 | 5,6-dihydroxy-7-isopropyl-1,1-dimethyl-2,3-dihydrophenanthren-4-one | 11011966 | 298.41 | 4.38 | 2 | 3 | 33.77 | 1.19 | 0.8 | 0.29 | 0.29 | 14.91 |
|  |  | MOL007041 | 2-isopropyl-8-methylphenanthrene-3,4-dione | 135872 | 264.34 | 4.16 | 0 | 2 | 40.86 | 1.23 | 0.81 | 0.23 | 0.43 | 14.89 |
|  |  | MOL007045 | 3α-hydroxytanshinoneⅡa | 14626764 | 310.37 | 3.56 | 1 | 4 | 44.93 | 0.53 | 0.22 | 0.44 | 0.3 | 23.78 |
|  |  | MOL007048 | (E)-3-[2-(3,4-dihydroxyphenyl)-7-hydroxy-benzofuran-4-yl]acrylic acid | 10403222 | 312.29 | 3.21 | 4 | 6 | 48.24 | 0.18 | -0.89 | 0.31 | 0.4 | 8.87 |
|  |  | MOL007049 | 4-methylenemiltirone | 14609851 | 266.36 | 4.33 | 0 | 2 | 34.35 | 1.25 | 0.87 | 0.23 | 0.38 | 14.6 |
|  |  | MOL007050 | 2-(4-hydroxy-3-methoxyphenyl)-5-(3-hydroxypropyl)-7-methoxy-3-benzofurancarboxaldehyde | 6709746 | 356.4 | 3.58 | 2 | 6 | 62.78 | 0.35 | -0.73 | 0.4 | 0.24 | 7.89 |
|  |  | MOL007058 | formyltanshinone | 14609847 | 290.28 | 3.36 | 0 | 4 | 73.44 | 0.54 | -0.28 | 0.42 | 0.41 | 24.12 |
|  |  | MOL007059 | 3-beta-Hydroxymethyllenetanshiquinone | 5318290 | 294.32 | 3.16 | 1 | 4 | 32.16 | 0.38 | -0.48 | 0.41 | 0.36 | 22.51 |
|  |  | MOL007061 | Methylenetanshinquinone | 105118 | 278.32 | 4.26 | 0 | 3 | 37.07 | 1.03 | 0.46 | 0.36 | 0.36 | 24.33 |
|  |  | MOL007063 | przewalskin a | 16090912 | 398.49 | 2.25 | 1 | 6 | 37.11 | -0.26 | -0.69 | 0.65 | 0.38 | 1.63 |
|  |  | MOL007064 | przewalskin b | 135033255 | 330.46 | 3.18 | 1 | 4 | 110.32 | 0.34 | 0.22 | 0.44 | 0.32 | 2.17 |
|  |  | MOL007068 | Przewaquinone B | 622085 | 292.3 | 2.99 | 1 | 4 | 62.24 | 0.39 | -0.45 | 0.41 | 0.38 | 24.94 |
|  |  | MOL007069 | przewaquinone c | 124307629 | 296.34 | 3.31 | 1 | 4 | 55.74 | 0.42 | -0.3 | 0.4 | 0.32 | 23.7 |
|  |  | MOL007070 | (6S,7R)-6,7-dihydroxy-1,6-dimethyl-8,9-dihydro-7H-naphtho[8,7-g]benzofuran-10,11-dione | 10470747 | 312.34 | 2.34 | 2 | 5 | 41.31 | -0.06 | -0.68 | 0.45 | 0.32 | 22.54 |
|  |  | MOL007077 | sclareol | 7060889 | 308.56 | 4.27 | 2 | 2 | 43.67 | 0.84 | 0.51 | 0.21 | 0.27 | 4.71 |
|  |  | MOL007081 | Danshenol B | 3083515 | 354.48 | 2.59 | 1 | 4 | 57.95 | 0.53 | 0.11 | 0.56 | 0.3 | 4.28 |
|  |  | MOL007082 | Danshenol A | 3083514 | 336.41 | 2.01 | 1 | 4 | 56.97 | 0.33 | -0.01 | 0.52 | 0.34 | 5.15 |
|  |  | MOL007085 | Salvilenone | 11011966 | 292.4 | 4.26 | 0 | 2 | 30.38 | 1.46 | 1.07 | 0.38 | 0.35 | 20.81 |
|  |  | MOL007088 | cryptotanshinone | 160254 | 296.39 | 3.44 | 0 | 3 | 52.34 | 0.95 | 0.51 | 0.4 | 0.29 | 17.3 |
|  |  | MOL007093 | dan-shexinkum d | 124307626 | 336.41 | 2.83 | 1 | 4 | 38.88 | 0.67 | -0.15 | 0.55 | 0.35 | 30 |
|  |  | MOL007098 | deoxyneocryptotanshinone | 15690458 | 298.41 | 4.32 | 1 | 3 | 49.4 | 0.85 | 0.24 | 0.29 | 0.3 | 27.17 |
|  |  | MOL007101 | dihydrotanshinoneⅠ | 40785034 | 278.32 | 2.86 | 0 | 3 | 45.04 | 0.95 | 0.43 | 0.36 | 0.4 | 18.32 |
|  |  | MOL007107 | C09092 | 442027 | 286.5 | 5.98 | 1 | 1 | 36.07 | 1.63 | 1.54 | 0.25 | 0.25 | -0.16 |
|  |  | MOL007108 | isocryptotanshi-none | 101603194 | 296.39 | 3.59 | 0 | 3 | 54.98 | 0.93 | 0.34 | 0.39 | 0.3 | 31.92 |
|  |  | MOL007111 | Isotanshinone II | 626354 | 294.37 | 4.66 | 0 | 3 | 49.92 | 1.03 | 0.45 | 0.4 | 0.3 | 24.73 |
|  |  | MOL007118 | microstegiol | 403772 | 298.46 | 4.75 | 1 | 2 | 39.61 | 1.05 | 0.99 | 0.28 | 0.33 | 4.52 |
|  |  | MOL007119 | miltionone Ⅰ | 5319835 | 312.39 | 3.33 | 1 | 4 | 49.68 | 0.35 | -0.11 | 0.32 | 0.35 | 41.49 |
|  |  | MOL007120 | miltionone Ⅱ | 5319836 | 312.39 | 2.14 | 1 | 4 | 71.03 | 0.62 | 0.03 | 0.44 | 0.28 | 2.91 |
|  |  | MOL007121 | miltipolone | 10086184 | 300.43 | 2.74 | 1 | 3 | 36.56 | 0.5 | 0.17 | 0.37 | 0.3 | 1.7 |
|  |  | MOL007122 | Miltirone | 160142 | 282.41 | 4.73 | 0 | 2 | 38.76 | 1.23 | 0.87 | 0.25 | 0.32 | 14.82 |
|  |  | MOL007124 | neocryptotanshinone ii | 5320066 | 270.35 | 3.61 | 1 | 3 | 39.46 | 0.76 | 0.16 | 0.23 | 0.32 | 26.98 |
|  |  | MOL007125 | neocryptotanshinone | 44425165 | 314.41 | 3.01 | 2 | 4 | 52.49 | 0.35 | -0.13 | 0.32 | 0.28 | 14.46 |
|  |  | MOL007127 | 1-methyl-8,9-dihydro-7H-naphtho[5,6-g]benzofuran-6,10,11-trione | 10062187 | 280.29 | 3.21 | 0 | 4 | 34.72 | 0.5 | -0.27 | 0.37 | 0.33 | 37.89 |
|  |  | MOL007132 | (2R)-3-(3,4-dihydroxyphenyl)-2-[(Z)-3-(3,4-dihydroxyphenyl)acryloyl]oxy-propionic acid | 9841799 | 360.34 | 2.69 | 5 | 8 | 109.38 | -0.33 | -1.02 | 0.35 | 0.41 | 2.01 |
|  |  | MOL007140 | (Z)-3-[2-[(E)-2-(3,4-dihydroxyphenyl)vinyl]-3,4-dihydroxy-phenyl]acrylic acid | 11602092 | 314.31 | 2.82 | 5 | 6 | 88.54 | -0.09 | -0.77 | 0.26 | 0.43 | 4.31 |
|  |  | MOL007145 | salviolone | 10355691 | 268.38 | 4.05 | 1 | 2 | 31.72 | 1.04 | 0.72 | 0.24 | 0.36 | 0.33 |
|  |  | MOL007149 | NSC 122421 | 94162 | 300.48 | 4.99 | 1 | 2 | 34.49 | 1.08 | 0.63 | 0.28 | 0.29 | 14.56 |
|  |  | MOL007150 | (6S)-6-hydroxy-1-methyl-6-methylol-8,9-dihydro-7H-naphtho[8,7-g]benzofuran-10,11-quinone | 16730071 | 312.34 | 2.42 | 2 | 5 | 75.39 | 0.03 | -0.74 | 0.46 | 0.29 | 23.45 |
|  |  | MOL007151 | Tanshindiol B | 5321620 | 312.34 | 2.34 | 2 | 5 | 42.67 | 0.05 | -0.63 | 0.45 | 0.33 | 22.25 |
|  |  | MOL007152 | Przewaquinone E | 126072 | 312.34 | 2.34 | 2 | 5 | 42.85 | -0.04 | -0.65 | 0.45 | 0.32 | 22.44 |
|  |  | MOL007154 | tanshinone iia | 164676 | 294.37 | 4.66 | 0 | 3 | 49.89 | 1.05 | 0.7 | 0.4 | 0.31 | 23.56 |
|  |  | MOL007155 | (6S)-6-(hydroxymethyl)-1,6-dimethyl-8,9-dihydro-7H-naphtho[8,7-g]benzofuran-10,11-dione | 9926694 | 310.37 | 3.57 | 1 | 4 | 65.26 | 0.44 | -0.31 | 0.45 | 0.29 | 23.48 |
|  |  | MOL007156 | tanshinone Ⅵ | 98054352 | 296.34 | 2.44 | 2 | 4 | 45.64 | 0.48 | -0.28 | 0.3 | 0.38 | 15.21 |
|  |  | MOL001601 | 1,2,5,6-tetrahydrotanshinone |  | 280.34 | 2.98 | 0 | 3 | 38.75 | 0.96 | 0.39 | 0.36 | 0.33 | 18.05 |
|  |  | MOL002222 | sugiol |  | 300.48 | 4.99 | 1 | 2 | 36.11 | 1.14 | 0.7 | 0.28 | 0.27 | 14.62 |
|  |  | MOL007051 | 6-o-syringyl-8-o-acetyl shanzhiside methyl ester |  | 628.64 | -1.13 | 5 | 16 | 46.69 | -1.73 | -2.08 | 0.71 | 0.22 | 9.94 |
|  |  | MOL007071 | przewaquinone f |  | 312.34 | 2.07 | 2 | 5 | 40.31 | -0.09 | -0.9 | 0.46 | 0.29 | 22.45 |
|  |  | MOL007079 | tanshinaldehyde |  | 308.35 | 3.83 | 0 | 4 | 52.47 | 0.57 | -0.07 | 0.45 | 0.32 | 23.49 |
|  |  | MOL007094 | danshenspiroketallactone |  | 282.36 | 3.24 | 0 | 3 | 50.43 | 0.88 | 0.51 | 0.31 | 0.34 | 15.19 |
|  |  | MOL007100 | dihydrotanshinlactone |  | 266.31 | 2.77 | 0 | 3 | 38.68 | 1.26 | 0.81 | 0.32 | 0.38 | 5.42 |
|  |  | MOL007105 | epidanshenspiroketallactone |  | 284.38 | 2.37 | 0 | 3 | 68.27 | 0.9 | 0.61 | 0.31 | 0.33 | 1.77 |
|  |  | MOL007115 | manool |  | 304.57 | 5.5 | 1 | 1 | 45.04 | 1.28 | 1.16 | 0.2 | 0.28 | 5.81 |
|  |  | MOL007123 | miltirone Ⅱ |  | 272.32 | 0.77 | 1 | 4 | 44.95 | 0.04 | -0.25 | 0.24 | 0.35 | 2.24 |
|  |  | MOL007130 | prolithospermic acid |  | 314.31 | 2.77 | 4 | 6 | 64.37 | 0.1 | -0.75 | 0.31 | 0.42 | 8.82 |
|  |  | MOL007141 | salvianolic acid g |  | 340.3 | 2.2 | 4 | 7 | 45.56 | -0.14 | -0.97 | 0.61 | 0.45 | 2.4 |
|  |  | MOL007142 | salvianolic acid j |  | 538.49 | 3.78 | 6 | 12 | 43.38 | -0.82 | -2.14 | 0.72 | 0.44 | 5.77 |
|  |  | MOL007143 | salvilenone Ⅰ |  | 270.4 | 2.88 | 1 | 2 | 32.43 | 1.13 | 0.77 | 0.23 | 0.3 | 1 |
| III/IV | *Carthami Flos* | MOL001771 | poriferast-5-en-3beta-ol | 457801 | 414.79 | 8.08 | 1 | 1 | 36.91 | 1.45 | 1.14 | 0.75 | 0 | 5.07 |
|  |  | MOL002680 | Flavoxanthin | 5281238 | 584.96 | 8.24 | 2 | 3 | 60.41 | 0.97 | -0.9 | 0.56 | 0.32 | 16.38 |
|  |  | MOL002694 | 4-[(E)-4-(3,5-dimethoxy-4-oxo-1-cyclohexa-2,5-dienylidene)but-2-enylidene]-2,6-dimethoxycyclohexa-2,5-dien-1-one | 10237057 | 356.4 | 0.36 | 0 | 6 | 48.47 | 0.81 | -0.34 | 0.36 | 0.27 | 3.24 |
|  |  | MOL002695 | lignan | 92242026 | 458.55 | 3.78 | 0 | 8 | 43.32 | 0.42 | -0.16 | 0.65 | 0.19 | 14.88 |
|  |  | MOL002706 | Phytoene | 5280784 | 545.04 | 14.54 | 0 | 0 | 39.56 | 2.22 | 1.7 | 0.5 | 0.3 | 2 |
|  |  | MOL002710 | Pyrethrin II | 5281555 | 372.5 | 3.74 | 0 | 5 | 48.36 | 0.53 | -0.21 | 0.35 | 0.32 | 1.79 |
|  |  | MOL002712 | 6-Hydroxykaempferol | 5281638 | 302.25 | 1.5 | 5 | 7 | 62.13 | 0.16 | -0.71 | 0.27 | 0.35 | 14.29 |
|  |  | MOL002714 | baicalein | 5281605 | 270.25 | 2.33 | 3 | 5 | 33.52 | 0.63 | -0.05 | 0.21 | 0.36 | 16.25 |
|  |  | MOL002717 | qt_carthamone | 131833009 | 286.25 | 0.7 | 3 | 6 | 51.03 | -0.31 | -0.92 | 0.2 | 0.47 | 3.64 |
|  |  | MOL002719 | 6-Hydroxynaringenin | 188308 | 288.27 | 2.03 | 4 | 6 | 33.23 | 0.27 | -0.27 | 0.24 | 0.34 | 15.67 |
|  |  | MOL002721 | quercetagetin | 5281680 | 318.25 | 1.24 | 6 | 8 | 45.01 | -0.06 | -0.93 | 0.31 | 0.34 | 13.82 |
|  |  | MOL002757 | 7,8-dimethyl-1H-pyrimido[5,6-g]quinoxaline-2,4-dione | 21786815 | 242.26 | 0.59 | 2 | 6 | 45.75 | 0.06 | -0.67 | 0.19 | 0.39 | -0.72 |
|  |  | MOL002773 | beta-carotene | 5280489 | 536.96 | 12 | 0 | 0 | 37.18 | 2.25 | 1.52 | 0.58 | 0.33 | 4.36 |
|  |  | MOL002776 | Baicalin | 64982 | 446.39 | 0.64 | 6 | 11 | 40.12 | -0.85 | -1.74 | 0.75 | 0.36 | 17.36 |
|  |  | MOL000358 | beta-sitosterol | 222284 | 414.79 | 8.08 | 1 | 1 | 36.91 | 1.32 | 0.99 | 0.75 | 0.23 | 5.36 |
|  |  | MOL000422 | kaempferol | 5280863 | 286.25 | 1.77 | 4 | 6 | 41.88 | 0.26 | -0.55 | 0.24 | 0 | 14.74 |
|  |  | MOL000449 | Stigmasterol | 5280794 | 412.77 | 7.64 | 1 | 1 | 43.83 | 1.44 | 1 | 0.76 | 0.22 | 5.57 |
|  |  | MOL000006 | luteolin | 5280445 | 286.25 | 2.07 | 4 | 6 | 36.16 | 0.19 | -0.84 | 0.25 | 0.39 | 15.94 |
|  |  | MOL000953 | CLR | 5997 | 386.73 | 7.38 | 1 | 1 | 37.87 | 1.43 | 1.13 | 0.68 | 0.2 | 4.52 |
|  |  | MOL000098 | quercetin | 5280343 | 302.25 | 1.5 | 5 | 7 | 46.43 | 0.05 | -0.77 | 0.28 | 0.38 | 14.4 |
|  |  | MOL002698 | lupeol-palmitate |  | 665.26 | 14.38 | 0 | 2 | 33.98 | 1.52 | 0.89 | 0.32 | 0.21 | 10.21 |
|  |  | MOL002707 | phytofluene |  | 543.02 | 14.1 | 0 | 0 | 43.18 | 2.29 | 1.76 | 0.5 | 0.3 | 2.6 |
| IV | *Angelicae Sinensis Radix* | MOL000358 | beta-sitosterol | 222284 | 414.79 | 8.08 | 1 | 1 | 36.91 | 1.32 | 0.99 | 0.75 | 0.23 | 5.36 |
|  |  | MOL000449 | Stigmasterol | 5280794 | 412.77 | 7.64 | 1 | 1 | 43.83 | 1.44 | 1 | 0.76 | 0.22 | 5.57 |
| IV | *Persicae Semen* | MOL001323 | Sitosterol alpha1 | 9548595 | 426.8 | 8.15 | 1 | 1 | 43.28 | 1.41 | 0.97 | 0.78 | 0.24 | 5.64 |
|  |  | MOL001328 | 2,3-didehydro GA70 | 101603111 | 330.41 | 1.16 | 2 | 5 | 63.29 | -0.27 | -0.84 | 0.5 | 0.36 | 7.62 |
|  |  | MOL001339 | GA119 | 100947721 | 332.43 | 1.81 | 2 | 5 | 76.36 | -0.12 | -0.48 | 0.49 | 0.32 | 8.35 |
|  |  | MOL001340 | GA120 | 100947720 | 314.41 | 2.47 | 1 | 4 | 84.85 | 0.38 | 0.1 | 0.45 | 0.35 | 8.4 |
|  |  | MOL001343 | GA122 | 101135488 | 330.41 | 1.3 | 2 | 5 | 64.79 | -0.17 | -0.73 | 0.5 | 0.34 | 7.01 |
|  |  | MOL001348 | gibberellin 17 | 5460657 | 378.46 | 1.49 | 4 | 7 | 94.64 | -0.87 | -1.14 | 0.49 | 0.32 | 11.67 |
|  |  | MOL001349 | 4a-formyl-7alpha-hydroxy-1-methyl-8-methylidene-4aalpha,4bbeta-gibbane-1alpha,10beta-dicarboxylic acid | 5460209 | 362.46 | 1.54 | 3 | 6 | 88.6 | -0.75 | -1.17 | 0.46 | 0.34 | 11.29 |
|  |  | MOL001350 | GA30 | 21596344 | 346.41 | 0.33 | 3 | 6 | 61.72 | -0.83 | -1.3 | 0.54 | 0.35 | 8.74 |
|  |  | MOL001351 | Gibberellin A44 | 443756 | 346.46 | 1.89 | 2 | 5 | 101.61 | -0.13 | -0.48 | 0.54 | 0.32 | 11.8 |
|  |  | MOL001352 | GA54 | 101603122 | 348.43 | 0.58 | 3 | 6 | 64.21 | -0.5 | -0.64 | 0.53 | 0.31 | 10.19 |
|  |  | MOL001353 | GA60 | 13071237 | 348.43 | 0.73 | 3 | 6 | 93.17 | -0.79 | -1.48 | 0.53 | 0.33 | 7.9 |
|  |  | MOL001355 | GA63 | 14160552 | 348.43 | 0.71 | 3 | 6 | 65.54 | -0.57 | -0.73 | 0.54 | 0.33 | 9.85 |
|  |  | MOL001358 | gibberellin 7 | 92782 | 330.41 | 1.5 | 2 | 5 | 73.8 | -0.18 | -0.4 | 0.5 | 0.35 | 9.79 |
|  |  | MOL001361 | GA87 | 101663385 | 362.41 | -0.55 | 4 | 7 | 68.85 | -1.14 | -1.7 | 0.57 | 0.37 | 8.76 |
|  |  | MOL000358 | beta-sitosterol | 222284 | 414.79 | 8.08 | 1 | 1 | 36.91 | 1.32 | 0.99 | 0.75 | 0.23 | 5.36 |
|  |  | MOL000493 | campesterol | 12358798 | 400.76 | 7.63 | 1 | 1 | 37.58 | 1.31 | 0.93 | 0.71 | 0 | 4.71 |
|  |  | MOL001329 | 2,3-didehydro GA77 |  | 346.41 | 0.28 | 3 | 6 | 88.08 | -0.67 | -1.1 | 0.53 | 0.37 | 7.6 |
|  |  | MOL001342 | GA121-isolactone |  | 330.41 | 1.34 | 2 | 5 | 72.7 | -0.26 | -0.75 | 0.54 | 0.34 | 7.63 |
|  |  | MOL001344 | GA122-isolactone |  | 330.41 | 1.34 | 2 | 5 | 88.11 | -0.18 | -0.65 | 0.54 | 0.32 | 7.4 |
|  |  | MOL001360 | GA77 |  | 348.43 | 0.73 | 3 | 6 | 87.89 | -0.64 | -1.11 | 0.53 | 0.34 | 7.28 |
|  |  | MOL001368 | 3-O-p-coumaroylquinic acid |  | 338.34 | -0.15 | 5 | 8 | 37.63 | -1.2 | -1.53 | 0.29 | 0.4 | 5.15 |
|  |  | MOL001371 | Populoside_qt |  | 286.3 | 2.94 | 3 | 5 | 108.89 | 0.49 | -0.42 | 0.2 | 0.42 | 5.86 |
|  |  | MOL000296 | hederagenin |  | 414.79 | 8.08 | 1 | 1 | 36.91 | 1.32 | 0.96 | 0.75 | 0 | 5.35 |
| IV | *Cyperi Rhizoma* | MOL003044 | Chryseriol | 5280666 | 300.28 | 2.32 | 3 | 6 | 35.85 | 0.39 | -0.53 | 0.27 | 0.32 | 16.31 |
|  |  | MOL000354 | isorhamnetin | 5281654 | 316.28 | 1.76 | 4 | 7 | 49.6 | 0.31 | -0.54 | 0.31 | 0.32 | 14.34 |
|  |  | MOL003542 | 8-Isopentenyl-kaempferol | 5318624 | 354.38 | 3.63 | 4 | 6 | 38.04 | 0.53 | -0.49 | 0.39 | 0 | 15.37 |
|  |  | MOL000358 | beta-sitosterol | 222284 | 414.79 | 8.08 | 1 | 1 | 36.91 | 1.32 | 0.99 | 0.75 | 0.23 | 5.36 |
|  |  | MOL000359 | sitosterol | 12303645 | 414.79 | 8.08 | 1 | 1 | 36.91 | 1.32 | 0.87 | 0.75 | 0.22 | 5.37 |
|  |  | MOL004053 | Isodalbergin | 5318543 | 268.28 | 3.1 | 1 | 4 | 35.45 | 0.8 | 0.2 | 0.2 | 0 | 3.62 |
|  |  | MOL004058 | Khell | 3828 | 260.26 | 2.17 | 0 | 5 | 33.19 | 1.12 | 0.6 | 0.19 | 0 | 10.87 |
|  |  | MOL004059 | khellol glucoside | 70697662 | 408.39 | -0.45 | 4 | 10 | 74.96 | -1.05 | -1.56 | 0.72 | 0 | 14.34 |
|  |  | MOL010489 | Resivit | 440833 | 306.29 | 1.09 | 6 | 7 | 30.84 | -0.49 | -1.03 | 0.27 | 0.36 | 1.44 |
|  |  | MOL004071 | Hyndarin | 72301 | 355.47 | 3.6 | 0 | 5 | 73.94 | 1 | 0.62 | 0.64 | 0 | 2.42 |
|  |  | MOL004077 | sugeonyl acetate | 52929817 | 276.41 | 2.52 | 0 | 3 | 45.08 | 0.72 | 0.58 | 0.2 | 0 | 4.22 |
|  |  | MOL000422 | kaempferol | 5280863 | 286.25 | 1.77 | 4 | 6 | 41.88 | 0.26 | -0.55 | 0.24 | 0 | 14.74 |
|  |  | MOL000449 | Stigmasterol | 5280794 | 412.77 | 7.64 | 1 | 1 | 43.83 | 1.44 | 1 | 0.76 | 0.22 | 5.57 |
|  |  | MOL000006 | luteolin | 5280445 | 286.25 | 2.07 | 4 | 6 | 36.16 | 0.19 | -0.84 | 0.25 | 0.39 | 15.94 |
|  |  | MOL000098 | quercetin | 5280343 | 302.25 | 1.5 | 5 | 7 | 46.43 | 0.05 | -0.77 | 0.28 | 0.38 | 14.4 |
|  |  | MOL004027 | 1,4-Epoxy-16-hydroxyheneicos-1,3,12,14,18-pentaene |  | 316.53 | 6.15 | 1 | 2 | 45.1 | 1.28 | 0.66 | 0.24 | 0 | -1.43 |
|  |  | MOL004068 | rosenonolactone |  | 316.48 | 3.38 | 0 | 3 | 79.84 | 0.72 | 0.43 | 0.37 | 0 | 6.55 |
|  |  | MOL004074 | stigmasterol glucoside_qt |  | 412.77 | 7.64 | 1 | 1 | 43.83 | 1.31 | 0.84 | 0.76 | 0 | 5.64 |
| IV | *Corydalis Rhizoma* | MOL001460 | Cryptopin | 72616 | 369.45 | 3.15 | 0 | 6 | 78.74 | 0.79 | 0.41 | 0.72 | 0.21 | 21.25 |
|  |  | MOL001461 | Dihydrochelerythrine | 485077 | 349.41 | 3.91 | 0 | 5 | 32.73 | 1.13 | 0.61 | 0.81 | 0.26 | 5.6 |
|  |  | MOL001463 | Dihydrosanguinarine | 124069 | 333.36 | 3.71 | 0 | 5 | 59.31 | 1 | 0.49 | 0.86 | 0.31 | 7.46 |
|  |  | MOL000217 | (S)-Scoulerine | 439654 | 327.41 | 3.1 | 2 | 5 | 32.28 | 0.89 | 0.34 | 0.54 | 0.2 | 1.69 |
|  |  | MOL002670 | Cavidine | 193148 | 353.45 | 3.72 | 0 | 5 | 35.64 | 1.08 | 0.63 | 0.81 | 0 | 5.78 |
|  |  | MOL002903 | (R)-Canadine | 443422 | 339.42 | 3.4 | 0 | 5 | 55.37 | 1.04 | 0.57 | 0.77 | 0.2 | 6.41 |
|  |  | MOL000359 | sitosterol | 12303645 | 414.79 | 8.08 | 1 | 1 | 36.91 | 1.32 | 0.87 | 0.75 | 0.22 | 5.37 |
|  |  | MOL004071 | Hyndarin | 72301 | 355.47 | 3.6 | 0 | 5 | 73.94 | 1 | 0.62 | 0.64 | 0 | 2.42 |
|  |  | MOL004191 | Capaurine | 94149 | 371.47 | 3.33 | 1 | 6 | 62.91 | 0.86 | 0.51 | 0.69 | 0.16 | 2.74 |
|  |  | MOL004193 | Clarkeanidine | 127376 | 327.41 | 3.1 | 2 | 5 | 86.65 | 0.86 | 0.42 | 0.54 | 0.21 | 2.74 |
|  |  | MOL004195 | CORYDALINE | 101301 | 369.5 | 3.92 | 0 | 5 | 65.84 | 1.23 | 0.9 | 0.68 | 0.14 | 1.77 |
|  |  | MOL004197 | Corydine | 10153 | 341.44 | 3.28 | 1 | 5 | 37.16 | 1.29 | 0.79 | 0.55 | 0.22 | 2.25 |
|  |  | MOL004198 | 18797-79-0 | 177014 | 367.43 | 2.73 | 1 | 6 | 46.06 | 0.72 | 0.19 | 0.85 | 0.26 | 7.66 |
|  |  | MOL004199 | Corynoloxine | 146167748 | 365.41 | 3.16 | 0 | 6 | 38.12 | 0.62 | 0.25 | 0.6 | 0.29 | 7.97 |
|  |  | MOL004200 | methyl-[2-(3,4,6,7-tetramethoxy-1-phenanthryl)ethyl]amine | 11462401 | 355.47 | 3.44 | 1 | 5 | 61.15 | 1.06 | 0.19 | 0.44 | 0.22 | 2.01 |
|  |  | MOL004202 | dehydrocavidine | 92043552 | 351.43 | 3.33 | 0 | 5 | 38.99 | 1.21 | 0.62 | 0.81 | 0.25 | 6.05 |
|  |  | MOL004203 | Dehydrocorybulbine | 5316439 | 352.44 | 3.88 | 1 | 4 | 46.97 | 1.32 | 0.49 | 0.63 | 0.15 | 1.06 |
|  |  | MOL004204 | dehydrocorydaline | 34781 | 366.47 | 4.13 | 0 | 4 | 41.98 | 1.35 | 0.48 | 0.68 | 0.13 | 1.67 |
|  |  | MOL004205 | Dehydrocorydalmine | 3083983 | 338.41 | 3.4 | 1 | 4 | 43.9 | 1.2 | 0.31 | 0.59 | 0.16 | 1.94 |
|  |  | MOL004208 | demethylcorydalmatine | 101663205 | 327.41 | 3.1 | 2 | 5 | 38.99 | 0.85 | 0.46 | 0.54 | 0.23 | 2.03 |
|  |  | MOL004209 | 13-methyldehydrocorydalmine | 25254728 | 352.44 | 3.88 | 1 | 4 | 35.94 | 1.15 | 0.2 | 0.63 | 0.16 | 1.68 |
|  |  | MOL004210 | (1S,8'R)-6,7-dimethoxy-2-methylspiro[3,4-dihydroisoquinoline-1,7'-6,8-dihydrocyclopenta[g][1,3]benzodioxole]-8'-ol | 21770852 | 369.45 | 2.75 | 1 | 6 | 43.95 | 0.62 | 0.22 | 0.72 | 0.21 | 10.89 |
|  |  | MOL004763 | Izoteolin | 133323 | 327.41 | 3.03 | 2 | 5 | 39.53 | 0.84 | 0.37 | 0.51 | 0.23 | 0.02 |
|  |  | MOL004216 | 13-methylpalmatrubine | 12275616 | 352.44 | 3.88 | 1 | 4 | 40.97 | 1.09 | 0.23 | 0.63 | 0.17 | 1.53 |
|  |  | MOL004220 | N-methyllaurotetanine | 6543699 | 341.44 | 3.28 | 1 | 5 | 41.62 | 0.84 | 0.32 | 0.56 | 0.21 | 0.14 |
|  |  | MOL004221 | norglaucing | 129316716 | 341.44 | 3 | 1 | 5 | 30.35 | 0.95 | 0.37 | 0.56 | 0.15 | 1.27 |
|  |  | MOL004224 | pontevedrine | 11047165 | 381.41 | 2.63 | 0 | 7 | 30.28 | 0.64 | -0.22 | 0.71 | 0.24 | 13.26 |
|  |  | MOL004226 | 24240-05-9 | 185559 | 353.4 | 2.95 | 0 | 6 | 53.75 | 0.8 | 0.39 | 0.83 | 0.29 | 22.7 |
|  |  | MOL004230 | stylopine | 697545 | 323.37 | 3.2 | 0 | 5 | 48.25 | 0.93 | 0.52 | 0.85 | 0.27 | 9.57 |
|  |  | MOL004231 | Tetrahydrocorysamine | 14315597 | 337.4 | 3.52 | 0 | 5 | 34.17 | 1.07 | 0.48 | 0.86 | 0.27 | 8.96 |
|  |  | MOL004232 | tetrahydroprotopapaverine | 40512630 | 329.43 | 3.34 | 2 | 5 | 57.28 | 0.79 | 0.32 | 0.33 | 0.26 | 2.6 |
|  |  | MOL004233 | ST057701 | 6992288 | 341.44 | 3.28 | 1 | 5 | 31.87 | 1.2 | 0.62 | 0.56 | 0.18 | -0.34 |
|  |  | MOL004234 | 2,3,9,10-tetramethoxy-13-methyl-5,6-dihydroisoquinolino[2,1-b]isoquinolin-8-one | 10362429 | 381.46 | 3.14 | 0 | 6 | 76.77 | 1.17 | 0.36 | 0.73 | 0.19 | 1.97 |
|  |  | MOL000449 | Stigmasterol | 5280794 | 412.77 | 7.64 | 1 | 1 | 43.83 | 1.44 | 1 | 0.76 | 0.22 | 5.57 |
|  |  | MOL000785 | palmatine | 19009 | 352.44 | 3.65 | 0 | 4 | 64.6 | 1.33 | 0.37 | 0.65 | 0.13 | 2.25 |
|  |  | MOL000787 | Fumarine | 4970 | 353.4 | 2.95 | 0 | 6 | 59.26 | 0.56 | -0.13 | 0.83 | 0.3 | 23.46 |
|  |  | MOL000790 | Isocorypalmine | 440229 | 341.44 | 3.35 | 1 | 5 | 35.77 | 0.85 | 0.43 | 0.59 | 0.21 | 2.47 |
|  |  | MOL000791 | bicuculline | 10237 | 367.38 | 2.83 | 0 | 7 | 69.67 | 0.72 | 0.02 | 0.88 | 0.33 | 15.83 |
|  |  | MOL000793 | C09367 | 12441 | 325.39 | 3.08 | 1 | 5 | 47.54 | 1.2 | 0.7 | 0.69 | 0.25 | 5.17 |
|  |  | MOL000098 | quercetin | 5280343 | 302.25 | 1.5 | 5 | 7 | 46.43 | 0.05 | -0.77 | 0.28 | 0.38 | 14.4 |
|  |  | MOL001454 | berberine |  | 336.39 | 3.45 | 0 | 4 | 36.86 | 1.24 | 0.57 | 0.78 | 0.19 | 6.57 |
|  |  | MOL001458 | coptisine |  | 320.34 | 3.25 | 0 | 4 | 30.67 | 1.21 | 0.32 | 0.86 | 0.26 | 9.33 |
|  |  | MOL001474 | sanguinarine |  | 332.35 | 3.47 | 0 | 4 | 37.81 | 1.26 | 0.15 | 0.86 | 0.3 | 7.84 |
|  |  | MOL004190 | (-)-alpha-N-methylcanadine |  | 354.46 | 3.49 | 0 | 4 | 45.06 | 1.15 | 0.45 | 0.8 | 0.19 | 5.78 |
|  |  | MOL004196 | Corydalmine |  | 340.45 | 4.68 | 1 | 4 | 52.5 | 1.21 | 0.57 | 0.59 | 0.23 | 2.1 |
|  |  | MOL004214 | isocorybulbine |  | 368.51 | 5.14 | 1 | 4 | 40.18 | 1.18 | 0.69 | 0.66 | 0.21 | 2.26 |
|  |  | MOL004215 | leonticine |  | 327.46 | 3.91 | 1 | 4 | 45.79 | 1.21 | 0.7 | 0.26 | 0.25 | 5.4 |
|  |  | MOL004225 | pseudocoptisine |  | 320.34 | 3.25 | 0 | 4 | 38.97 | 1.23 | 0.12 | 0.86 | 0.27 | 8.56 |
|  |  | MOL004228 | saulatine |  | 396.47 | 3.25 | 0 | 6 | 42.74 | 0.55 | -0.08 | 0.79 | 0.21 | 15.02 |
| IV | *Radix Paeoniae Rubra* | MOL007022 | evofolinB | 101504852 | 318.35 | 2.07 | 3 | 6 | 64.74 | 0 | -0.66 | 0.22 | 0.29 | 17.01 |
|  |  | MOL007016 | Paeoniflorigenone | 70698143 | 318.35 | 0.79 | 1 | 6 | 65.33 | -0.13 | -0.43 | 0.37 | 0.36 | 6.18 |
|  |  | MOL005043 | campest-5-en-3beta-ol | 173183 | 400.76 | 7.63 | 1 | 1 | 37.58 | 1.32 | 0.94 | 0.71 | 0.23 | 4.43 |
|  |  | MOL004355 | Spinasterol | 5281331 | 412.77 | 7.64 | 1 | 1 | 42.98 | 1.44 | 1.04 | 0.76 | 0.21 | 5.32 |
|  |  | MOL002883 | Ethyl oleate (NF) | 5363269 | 310.58 | 7.44 | 0 | 2 | 32.4 | 1.4 | 1.1 | 0.19 | 0.19 | 4.85 |
|  |  | MOL002776 | Baicalin | 64982 | 446.39 | 0.64 | 6 | 11 | 40.12 | -0.85 | -1.74 | 0.75 | 0.36 | 17.36 |
|  |  | MOL002714 | baicalein | 5281605 | 270.25 | 2.33 | 3 | 5 | 33.52 | 0.63 | -0.05 | 0.21 | 0.36 | 16.25 |
|  |  | MOL001925 | paeoniflorin_qt | 11973336 | 318.35 | 0.46 | 2 | 6 | 68.18 | -0.34 | -0.73 | 0.4 | 0.39 | 8.81 |
|  |  | MOL001924 | paeoniflorin | 442534 | 480.51 | -1.28 | 5 | 11 | 53.87 | -1.47 | -1.86 | 0.79 | 0.34 | 13.88 |
|  |  | MOL001002 | ellagic acid | 5281855 | 302.2 | 1.48 | 4 | 8 | 43.06 | -0.44 | -1.41 | 0.43 | 0.43 | -1.04 |
|  |  | MOL000492 | (+)-catechin | 9064 | 290.29 | 1.92 | 5 | 6 | 54.83 | -0.03 | -0.73 | 0.24 | 0 | 0.61 |
|  |  | MOL000449 | Stigmasterol | 5280794 | 412.77 | 7.64 | 1 | 1 | 43.83 | 1.44 | 1 | 0.76 | 0.22 | 5.57 |
|  |  | MOL000359 | sitosterol | 12303645 | 414.79 | 8.08 | 1 | 1 | 36.91 | 1.32 | 0.87 | 0.75 | 0.22 | 5.37 |
|  |  | MOL000358 | beta-sitosterol | 222284 | 414.79 | 8.08 | 1 | 1 | 36.91 | 1.32 | 0.99 | 0.75 | 0.23 | 5.36 |
|  |  | MOL007025 | isobenzoylpaeoniflorin |  | 584.62 | 0.76 | 4 | 12 | 31.14 | -0.85 | -1.33 | 0.54 | 0.35 | 21.1 |
|  |  | MOL007018 | 9-ethyl-neo-paeoniaflorin A_qt |  | 334.4 | 1.48 | 1 | 6 | 64.42 | -0.01 | -0.32 | 0.3 | 0.35 | 2.08 |
|  |  | MOL007014 | 8-debenzoylpaeonidanin |  | 390.43 | -3.28 | 5 | 10 | 31.74 | -1.56 | -2.17 | 0.45 | 0.2 | 7.63 |
|  |  | MOL007012 | 4-o-methyl-paeoniflorin_qt |  | 332.38 | 0.87 | 1 | 6 | 56.7 | 0.4 | -0.2 | 0.43 | 0.3 | 8.94 |
|  |  | MOL007008 | 4-ethyl-paeoniflorin_qt |  | 332.38 | 1.02 | 1 | 6 | 56.87 | -0.17 | -0.52 | 0.44 | 0.35 | 8.88 |
|  |  | MOL007005 | Albiflorin_qt |  | 318.35 | 0.42 | 2 | 6 | 48.7 | -0.38 | -0.75 | 0.33 | 0.41 | 5.91 |
|  |  | MOL007004 | Albiflorin |  | 480.51 | -1.33 | 5 | 11 | 30.25 | -1.52 | -2.33 | 0.77 | 0.35 | 7.83 |
|  |  | MOL007003 | benzoyl paeoniflorin |  | 584.62 | 0.76 | 4 | 12 | 31.14 | -1.35 | -2.08 | 0.54 | 0.4 | 15.66 |
|  |  | MOL006999 | stigmast-7-en-3-ol |  | 414.79 | 8.08 | 1 | 1 | 37.42 | 1.32 | 0.85 | 0.75 | 0.22 | 5.85 |
|  |  | MOL006996 | 1-o-beta-d-glucopyranosylpaeonisuffrone_qt |  | 332.38 | 0.51 | 1 | 6 | 65.08 | -0.05 | -0.49 | 0.35 | 0.38 | 7.19 |
|  |  | MOL006994 | 1-o-beta-d-glucopyranosyl-8-o-benzoylpaeonisuffrone_qt |  | 302.35 | 0.44 | 1 | 5 | 36.01 | -0.03 | -0.47 | 0.3 | 0.41 | 5.98 |
|  |  | MOL006992 | (2R,3R)-4-methoxyl-distylin |  | 318.3 | 1.89 | 4 | 7 | 59.98 | 0.17 | -0.61 | 0.3 | 0.33 | 15.08 |
|  |  | MOL006990 | (1S,2S,4R)-trans-2-hydroxy-1,8-cineole-B-D-glucopyranoside |  | 332.44 | -0.57 | 4 | 7 | 30.25 | -0.77 | -1.14 | 0.27 | 0.25 | 0.98 |
|  |  | MOL001921 | Lactiflorin |  | 462.49 | -0.57 | 3 | 10 | 49.12 | -1.13 | -1.76 | 0.8 | 0.34 | 7.26 |
|  |  | MOL001918 | paeoniflorgenone |  | 318.35 | 0.79 | 1 | 6 | 87.59 | -0.09 | -0.56 | 0.37 | 0.37 | 7.45 |
| IV | *Chuanxiong Rhizoma* | MOL001494 | Mandenol | 5282184 | 308.56 | 6.99 | 0 | 2 | 42 | 1.46 | 1.14 | 0.19 | 0.25 | 5.39 |
|  |  | MOL002135 | Myricanone | 161748 | 356.45 | 4.1 | 2 | 5 | 40.6 | 0.67 | -0.08 | 0.51 | 0.24 | 4.39 |
|  |  | MOL002140 | Perlolyrine | 160179 | 264.3 | 3.2 | 2 | 3 | 65.95 | 0.88 | 0.15 | 0.27 | 0.21 | 12.62 |
|  |  | MOL002151 | senkyunone | 91726743 | 326.52 | 6.32 | 0 | 2 | 47.66 | 1.15 | 0.5 | 0.24 | 0.34 | 2.42 |
|  |  | MOL000359 | sitosterol | 12303645 | 414.79 | 8.08 | 1 | 1 | 36.91 | 1.32 | 0.87 | 0.75 | 0.22 | 5.37 |
|  |  | MOL000433 | FA | 6037 | 441.45 | 0.01 | 7 | 13 | 68.96 | -1.5 | -2.59 | 0.71 | 0 | 24.81 |
|  |  | MOL002157 | wallichilide |  | 412.57 | 4.82 | 0 | 5 | 42.31 | 0.82 | 0.73 | 0.71 | 0.24 | 6.85 |
| IV | *Olibanun* | MOL001243 | 3alpha-Hydroxy-olean-12-en-24-oic-acid | 637234 | 456.78 | 6.42 | 2 | 3 | 39.32 | 0.6 | 0.2 | 0.75 | 0.25 | 2.39 |
|  |  | MOL001255 | Boswellic acid | 168928 | 456.78 | 6.47 | 2 | 3 | 39.55 | 0.59 | 0.24 | 0.75 | 0.25 | 2.94 |
|  |  | MOL001263 | 3-oxo-tirucallic,acid | 134071966 | 454.76 | 6.99 | 1 | 3 | 42.86 | 0.58 | -0.06 | 0.81 | 0.28 | 6.6 |
|  |  | MOL001265 | acetyl-alpha-boswellic,acid | 15181201 | 498.82 | 6.8 | 1 | 4 | 42.73 | 0.6 | 0.28 | 0.7 | 0.25 | 2.99 |
|  |  | MOL001272 | incensole | 44583885 | 306.54 | 4.97 | 1 | 2 | 45.59 | 1.33 | 1.2 | 0.22 | 0.26 | 6.94 |
|  |  | MOL001295 | phyllocladene | 44559813 | 272.52 | 5.63 | 0 | 0 | 33.4 | 1.81 | 2.1 | 0.27 | 0.23 | 3.93 |
|  |  | MOL001215 | tirucallol |  | 426.8 | 8.12 | 1 | 1 | 42.12 | 1.38 | 1.07 | 0.75 | 0.24 | 6.9 |
|  |  | MOL001241 | O-acetyl-α-boswellic acid |  | 498.82 | 6.8 | 1 | 4 | 42.73 | 0.68 | 0.41 | 0.7 | 0.25 | 4.13 |
| IV | *Citri Reticulatae Pericarpium Viride* | MOL001798 | neohesperidin_qt | 42607889 | 302.3 | 2.28 | 3 | 6 | 71.17 | 0.26 | -0.47 | 0.27 | 0 | 15.96 |
|  |  | MOL001803 | Sinensetin | 145659 | 372.4 | 3.06 | 0 | 7 | 50.56 | 1.12 | 0.04 | 0.45 | 0.13 | 15.52 |
|  |  | MOL004328 | naringenin | 439246 | 272.27 | 2.3 | 3 | 5 | 59.29 | 0.28 | -0.37 | 0.21 | 0.4 | 16.98 |
|  |  | MOL005100 | 5,7-dihydroxy-2-(3-hydroxy-4-methoxyphenyl)chroman-4-one | 676152 | 302.3 | 2.28 | 3 | 6 | 47.74 | 0.28 | -0.3 | 0.27 | 0.31 | 16.51 |
|  |  | MOL005828 | nobiletin | 72344 | 402.43 | 3.04 | 0 | 8 | 61.67 | 1.05 | -0.08 | 0.52 | 0.13 | 16.2 |
| IV | *Rhizoma Rehmanniae glutinosae* | NA | NA | NA | NA | NA | NA | NA | NA | NA | NA | NA | NA | NA |
|  |  |  |  |  |  |  |  |  |  |  |  |  |  |  |
| Note: |  |  |  |  |  |  |  |  |  |  |  |  |  |  |
|  | Eliminated term | |  |  |  |  |  |  |  |  |  |  |  |  |
|  | Pubchem Cid corresponded by identical InChIKey | | |  |  |  |  |  |  |  |  |  |  |  |

**Table S2.** Common and unique component in four herbal strategies

| **Strategy** | **Number** | **Mol ID of component** |
| --- | --- | --- |
| I/ III/IV | 3 | MOL000098 |
|  |  | MOL000449 |
|  |  | MOL000358 |
| II/III/IV | 51 | MOL007082 |
|  |  | MOL007125 |
|  |  | MOL007069 |
|  |  | MOL007156 |
|  |  | MOL000006 |
|  |  | MOL007140 |
|  |  | MOL007101 |
|  |  | MOL000569 |
|  |  | MOL001659 |
|  |  | MOL002651 |
|  |  | MOL007059 |
|  |  | MOL007063 |
|  |  | MOL007150 |
|  |  | MOL007145 |
|  |  | MOL007124 |
|  |  | MOL007093 |
|  |  | MOL007155 |
|  |  | MOL007068 |
|  |  | MOL007085 |
|  |  | MOL007108 |
|  |  | MOL007120 |
|  |  | MOL007036 |
|  |  | MOL007122 |
|  |  | MOL007119 |
|  |  | MOL007058 |
|  |  | MOL007121 |
|  |  | MOL006824 |
|  |  | MOL007118 |
|  |  | MOL007127 |
|  |  | MOL007088 |
|  |  | MOL007050 |
|  |  | MOL007048 |
|  |  | MOL007132 |
|  |  | MOL007081 |
|  |  | MOL007070 |
|  |  | MOL007151 |
|  |  | MOL007045 |
|  |  | MOL007064 |
|  |  | MOL007077 |
|  |  | MOL001942 |
|  |  | MOL007107 |
|  |  | MOL007149 |
|  |  | MOL001771 |
|  |  | MOL007154 |
|  |  | MOL007111 |
|  |  | MOL007152 |
|  |  | MOL007098 |
|  |  | MOL007041 |
|  |  | MOL007049 |
|  |  | MOL002776 |
|  |  | MOL007061 |
| I/IV | 1 | MOL001494 |
| III/IV | 14 | MOL002694 |
|  |  | MOL002719 |
|  |  | MOL002717 |
|  |  | MOL002757 |
|  |  | MOL002721 |
|  |  | MOL000422 |
|  |  | MOL002714 |
|  |  | MOL000953 |
|  |  | MOL002680 |
|  |  | MOL002706 |
|  |  | MOL002710 |
|  |  | MOL002712 |
|  |  | MOL002773 |
|  |  | MOL002695 |
| I | 4 | MOL005344 |
|  |  | MOL002879 |
|  |  | MOL007475 |
|  |  | MOL001792 |
| IV | 85 | MOL004355 |
|  |  | MOL001352 |
|  |  | MOL004221 |
|  |  | MOL000790 |
|  |  | MOL000354 |
|  |  | MOL000787 |
|  |  | MOL000359 |
|  |  | MOL004232 |
|  |  | MOL004210 |
|  |  | MOL000793 |
|  |  | MOL007016 |
|  |  | MOL004328 |
|  |  | MOL007022 |
|  |  | MOL001463 |
|  |  | MOL000785 |
|  |  | MOL004230 |
|  |  | MOL004193 |
|  |  | MOL003044 |
|  |  | MOL001263 |
|  |  | MOL000433 |
|  |  | MOL001358 |
|  |  | MOL004071 |
|  |  | MOL001255 |
|  |  | MOL004224 |
|  |  | MOL004220 |
|  |  | MOL001339 |
|  |  | MOL001328 |
|  |  | MOL001924 |
|  |  | MOL001355 |
|  |  | MOL004200 |
|  |  | MOL004195 |
|  |  | MOL004198 |
|  |  | MOL004059 |
|  |  | MOL001348 |
|  |  | MOL001343 |
|  |  | MOL000791 |
|  |  | MOL004234 |
|  |  | MOL001340 |
|  |  | MOL001461 |
|  |  | MOL004053 |
|  |  | MOL001295 |
|  |  | MOL002903 |
|  |  | MOL004203 |
|  |  | MOL002151 |
|  |  | MOL002883 |
|  |  | MOL001353 |
|  |  | MOL004208 |
|  |  | MOL004058 |
|  |  | MOL001798 |
|  |  | MOL004077 |
|  |  | MOL000492 |
|  |  | MOL010489 |
|  |  | MOL001265 |
|  |  | MOL004197 |
|  |  | MOL001925 |
|  |  | MOL001243 |
|  |  | MOL002135 |
|  |  | MOL004209 |
|  |  | MOL001361 |
|  |  | MOL005100 |
|  |  | MOL004191 |
|  |  | MOL004202 |
|  |  | MOL000493 |
|  |  | MOL004216 |
|  |  | MOL001002 |
|  |  | MOL004199 |
|  |  | MOL001323 |
|  |  | MOL001350 |
|  |  | MOL004231 |
|  |  | MOL005828 |
|  |  | MOL003542 |
|  |  | MOL001351 |
|  |  | MOL002140 |
|  |  | MOL000217 |
|  |  | MOL005043 |
|  |  | MOL004233 |
|  |  | MOL001460 |
|  |  | MOL002670 |
|  |  | MOL001349 |
|  |  | MOL004205 |
|  |  | MOL004204 |
|  |  | MOL004763 |
|  |  | MOL004226 |
|  |  | MOL001803 |
|  |  | MOL001272 |

**Table S3.** Targets of different components in all herbal strategies

| **Mol ID** | **Molecule Name** | **Target** | **Probability** |
| --- | --- | --- | --- |
| MOL000098 | quercetin | NOX4 | 1 |
|  |  | AKR1B1 | 1 |
|  |  | CDK5R1 | 1 |
|  |  | CDK5 | 1 |
|  |  | XDH | 1 |
|  |  | MAOA | 1 |
|  |  | FLT3 | 1 |
|  |  | CA2 | 1 |
|  |  | CDK1 | 1 |
|  |  | CCNB1 | 1 |
|  |  | CCNB2 | 1 |
|  |  | CCNB3 | 1 |
|  |  | ALOX5 | 1 |
|  |  | ADORA1 | 1 |
|  |  | CA7 | 1 |
|  |  | GLO1 | 1 |
|  |  | APP | 1 |
|  |  | SYK | 1 |
|  |  | GSK3B | 1 |
|  |  | PARP1 | 1 |
|  |  | TTR | 1 |
|  |  | MMP9 | 1 |
|  |  | CA12 | 1 |
|  |  | MMP2 | 1 |
|  |  | CA4 | 1 |
|  |  | MMP12 | 1 |
|  |  | CD38 | 1 |
|  |  | CYP1B1 | 1 |
|  |  | ABCG2 | 1 |
|  |  | AKR1B10 | 1 |
|  |  | TNKS2 | 1 |
|  |  | TNKS | 1 |
|  |  | TOP1 | 1 |
|  |  | ARG1 | 1 |
| MOL001792 | DFV | CYP19A1 | 1 |
| MOL000006 | luteolin | NOX4 | 1 |
|  |  | AKR1B1 | 1 |
|  |  | CDK5R1 | 1 |
|  |  | CDK5 | 1 |
|  |  | XDH | 1 |
|  |  | MAOA | 1 |
|  |  | FLT3 | 1 |
|  |  | CA2 | 1 |
|  |  | CDK1 | 1 |
|  |  | CCNB1 | 1 |
|  |  | CCNB2 | 1 |
|  |  | CCNB3 | 1 |
|  |  | ALOX5 | 1 |
|  |  | ADORA1 | 1 |
|  |  | CA7 | 1 |
|  |  | GLO1 | 1 |
|  |  | APP | 1 |
|  |  | SYK | 1 |
|  |  | GSK3B | 1 |
|  |  | PARP1 | 1 |
|  |  | TTR | 1 |
|  |  | MMP9 | 1 |
|  |  | CA12 | 1 |
|  |  | MMP2 | 1 |
|  |  | CA4 | 1 |
|  |  | MMP12 | 1 |
|  |  | CD38 | 1 |
|  |  | CYP1B1 | 1 |
|  |  | ABCG2 | 1 |
|  |  | AKR1B10 | 1 |
|  |  | TNKS2 | 1 |
|  |  | TNKS | 1 |
|  |  | TOP1 | 1 |
|  |  | ARG1 | 1 |
| MOL000569 | digallate | POLA1 | 1 |
|  |  | POLB | 1 |
| MOL007081 | danshenol A | AKR1B1 | 1 |
| MOL007082 | danshenol B | AKR1B1 | 1 |
| MOL007088 | cryptotanshinone | AKR1B1 | 1 |
|  |  | ACHE | 1 |
|  |  | CES1 | 1 |
|  |  | CES2 | 1 |
|  |  | STAT3 | 1 |
| MOL007101 | dihydrotanshinoneⅠ | AKR1B1 | 1 |
|  |  | ACHE | 1 |
|  |  | CES1 | 1 |
|  |  | PTPN6 | 1 |
|  |  | CES2 | 1 |
|  |  | PTPN11 | 1 |
| MOL007122 | miltirone | CES1 | 1 |
|  |  | CES2 | 1 |
| MOL007154 | tanshinone iia | AKR1B1 | 1 |
|  |  | CES1 | 1 |
|  |  | TERT | 1 |
|  |  | PTPN6 | 1 |
|  |  | CES2 | 1 |
|  |  | PTPN11 | 1 |
| MOL007155 | (6S)-6-(hydroxymethyl)-1,6-dimethyl-8,9-dihydro-7H-naphtho[8,7-g]benzofuran-10,11-dione | EED | 1 |
|  |  | EZH2 | 1 |
|  |  | SUZ12 | 1 |
| MOL000422 | kaempferol | NOX4 | 1 |
|  |  | AKR1B1 | 1 |
|  |  | XDH | 1 |
|  |  | TYR | 1 |
|  |  | FLT3 | 1 |
|  |  | CA2 | 1 |
|  |  | ALOX5 | 1 |
|  |  | CA7 | 1 |
|  |  | HSD17B2 | 1 |
|  |  | ABCC1 | 1 |
|  |  | HSD17B1 | 1 |
|  |  | AHR | 1 |
|  |  | CA12 | 1 |
|  |  | ESRRA | 1 |
|  |  | ABCB1 | 1 |
|  |  | CYP1B1 | 1 |
|  |  | ABCG2 | 1 |
| MOL002714 | baicalein | KDM4E | 1 |
|  |  | XDH | 1 |
|  |  | ALOX15 | 1 |
|  |  | CDK1 | 1 |
|  |  | ALOX12 | 1 |
|  |  | GRK6 | 1 |
| MOL002721 | quercetagetin | PIM1 | 1 |
| MOL000354 | isorhamnetin | XDH | 1 |
|  |  | CA2 | 1 |
|  |  | CA7 | 1 |
|  |  | CA12 | 1 |
|  |  | CA4 | 1 |
|  |  | CYP1B1 | 1 |
| MOL003044 | chryseriol | ABCC1 | 1 |
|  |  | CYP1B1 | 1 |
| MOL000785 | palmatine | ACHE | 1 |
| MOL000790 | isocorypalmine | DRD1 | 1 |
|  |  | DRD2 | 1 |
| MOL004220 | N-methyllaurotetanine | HTR2B | 1 |
|  |  | DRD1 | 1 |
|  |  | HTR1A | 1 |
|  |  | HTR7 | 1 |
|  |  | HTR6 | 1 |
|  |  | HTR5A | 1 |
| MOL001002 | ellagic acid | GPR35 | 1 |
|  |  | ERBB2 | 1 |
|  |  | AKR1B1 | 1 |
|  |  | CCND1 | 1 |
|  |  | CDK4 | 1 |
|  |  | PDGFRB | 1 |
|  |  | FLT4 | 1 |
|  |  | IGF1R | 1 |
|  |  | INSR | 1 |
|  |  | EGFR | 1 |
|  |  | CA2 | 1 |
|  |  | CDK2 | 1 |
|  |  | CCNA1 | 1 |
|  |  | CCNA2 | 1 |
|  |  | AURKB | 1 |
|  |  | CA7 | 1 |
|  |  | CA1 | 1 |
|  |  | GSK3B | 1 |
|  |  | SRC | 1 |
|  |  | PTK2 | 1 |
|  |  | KDR | 1 |
|  |  | PLK1 | 1 |
|  |  | CA6 | 1 |
|  |  | CA12 | 1 |
|  |  | CA14 | 1 |
|  |  | CA9 | 1 |
|  |  | CSNK2A1 | 1 |
|  |  | MET | 1 |
|  |  | CA4 | 1 |
|  |  | PLK4 | 1 |
|  |  | CA13 | 1 |
|  |  | TEK | 1 |
|  |  | AKT1 | 1 |
|  |  | AURKA | 1 |
|  |  | CA5A | 1 |
|  |  | BACE1 | 1 |
|  |  | MAP3K8 | 1 |
|  |  | BRAF | 1 |
|  |  | EPHB4 | 1 |
|  |  | HSPA1A | 1 |
|  |  | NUAK1 | 1 |
|  |  | SQLE | 1 |
|  |  | FGR | 1 |
|  |  | LYN | 1 |
| MOL001255 | boswellic acid | PREP | 1 |
|  |  | PTGES | 1 |
| MOL001803 | sinensetin | ABCG2 | 1 |
| MOL004328 | naringenin | CYP19A1 | 1 |
|  |  | CA7 | 1 |
|  |  | ABCC1 | 1 |
|  |  | HSD17B1 | 1 |
|  |  | CA12 | 1 |
|  |  | SHBG | 1 |
|  |  | CA4 | 1 |
|  |  | CYP1B1 | 1 |
|  |  | CBR1 | 1 |
| MOL005828 | nobiletin | ABCG2 | 1 |

**Table S4.** Targets of each herbal strategy by de-duplication

| **Strategy** | **Target** |
| --- | --- |
| I | ABCB1 |
|  | ABCC1 |
|  | ABCG2 |
|  | ADORA1 |
|  | ADORA2A |
|  | AKR1A1 |
|  | AKR1B1 |
|  | AKR1C1 |
|  | AKR1C2 |
|  | AKR1C3 |
|  | AKR1C4 |
|  | AKT1 |
|  | ALK |
|  | ALOX12 |
|  | ALOX15 |
|  | ALOX5 |
|  | AURKB |
|  | AVPR2 |
|  | AXL |
|  | BACE1 |
|  | CA1 |
|  | CA12 |
|  | CA13 |
|  | CA14 |
|  | CA2 |
|  | CA3 |
|  | CA4 |
|  | CA5A |
|  | CA6 |
|  | CA7 |
|  | CA9 |
|  | CAMK2B |
|  | CDK1 |
|  | CSNK2A1 |
|  | CXCR1 |
|  | CYP19A1 |
|  | CYP1B1 |
|  | DAPK1 |
|  | DRD4 |
|  | EGFR |
|  | F2 |
|  | FLT3 |
|  | GLO1 |
|  | GSK3B |
|  | HSD17B2 |
|  | IGF1R |
|  | KDR |
|  | MAOA |
|  | MET |
|  | MMP13 |
|  | MMP2 |
|  | MMP3 |
|  | MMP9 |
|  | MPO |
|  | NEK2 |
|  | NEK6 |
|  | NOX4 |
|  | NUAK1 |
|  | PIK3R1 |
|  | PIM1 |
|  | PKN1 |
|  | PLA2G1B |
|  | PLK1 |
|  | PTK2 |
|  | PYGL |
|  | SRC |
|  | XDH |
| II | ABCG2 |
|  | ACHE |
|  | ADORA1 |
|  | AKR1B1 |
|  | AKR1B10 |
|  | ALOX5 |
|  | APP |
|  | ARG1 |
|  | CA12 |
|  | CA2 |
|  | CA4 |
|  | CA7 |
|  | CCNB1 |
|  | CCNB2 |
|  | CCNB3 |
|  | CD38 |
|  | CDK1 |
|  | CDK5 |
|  | CDK5R1 |
|  | CES1 |
|  | CES2 |
|  | CYP1B1 |
|  | EED |
|  | EZH2 |
|  | FLT3 |
|  | GLO1 |
|  | GSK3B |
|  | MAOA |
|  | MMP12 |
|  | MMP2 |
|  | MMP9 |
|  | NOX4 |
|  | PARP1 |
|  | POLA1 |
|  | POLB |
|  | PTPN11 |
|  | PTPN6 |
|  | STAT3 |
|  | SUZ12 |
|  | SYK |
|  | TERT |
|  | TNKS |
|  | TNKS2 |
|  | TOP1 |
|  | TTR |
|  | XDH |
| III | ABCB1 |
|  | ABCC1 |
|  | ABCG2 |
|  | ACHE |
|  | ADORA1 |
|  | ADORA2A |
|  | AHR |
|  | AKR1A1 |
|  | AKR1B1 |
|  | AKR1B10 |
|  | AKR1C1 |
|  | AKR1C2 |
|  | AKR1C3 |
|  | AKR1C4 |
|  | AKT1 |
|  | ALK |
|  | ALOX12 |
|  | ALOX15 |
|  | ALOX5 |
|  | APP |
|  | ARG1 |
|  | AURKB |
|  | AVPR2 |
|  | AXL |
|  | BACE1 |
|  | CA1 |
|  | CA12 |
|  | CA13 |
|  | CA14 |
|  | CA2 |
|  | CA3 |
|  | CA4 |
|  | CA5A |
|  | CA6 |
|  | CA7 |
|  | CA9 |
|  | CAMK2B |
|  | CCNB1 |
|  | CCNB2 |
|  | CCNB3 |
|  | CD38 |
|  | CDK1 |
|  | CDK5 |
|  | CDK5R1 |
|  | CES1 |
|  | CES2 |
|  | CSNK2A1 |
|  | CXCR1 |
|  | CYP19A1 |
|  | CYP1B1 |
|  | DAPK1 |
|  | DRD4 |
|  | EED |
|  | EGFR |
|  | ESRRA |
|  | EZH2 |
|  | F2 |
|  | FLT3 |
|  | GLO1 |
|  | GRK6 |
|  | GSK3B |
|  | HSD17B1 |
|  | HSD17B2 |
|  | IGF1R |
|  | KDM4E |
|  | KDR |
|  | MAOA |
|  | MET |
|  | MMP12 |
|  | MMP13 |
|  | MMP2 |
|  | MMP3 |
|  | MMP9 |
|  | MPO |
|  | NEK2 |
|  | NEK6 |
|  | NOX4 |
|  | NUAK1 |
|  | PARP1 |
|  | PIK3R1 |
|  | PIM1 |
|  | PKN1 |
|  | PLA2G1B |
|  | PLK1 |
|  | POLA1 |
|  | POLB |
|  | PTK2 |
|  | PTPN11 |
|  | PTPN6 |
|  | PYGL |
|  | SRC |
|  | STAT3 |
|  | SUZ12 |
|  | SYK |
|  | TERT |
|  | TNKS |
|  | TNKS2 |
|  | TOP1 |
|  | TTR |
|  | TYR |
|  | XDH |
| IV | NOX4 |
|  | AKR1B1 |
|  | CDK5R1 |
|  | CDK5 |
|  | XDH |
|  | MAOA |
|  | FLT3 |
|  | CA2 |
|  | CCNB3 |
|  | CDK1 |
|  | CCNB1 |
|  | CCNB2 |
|  | ALOX5 |
|  | ADORA1 |
|  | CA7 |
|  | GLO1 |
|  | APP |
|  | SYK |
|  | GSK3B |
|  | PARP1 |
|  | TTR |
|  | MMP9 |
|  | CA12 |
|  | MMP2 |
|  | CA4 |
|  | MMP12 |
|  | CD38 |
|  | CYP1B1 |
|  | ABCG2 |
|  | AKR1B10 |
|  | TNKS2 |
|  | TNKS |
|  | TOP1 |
|  | ARG1 |
|  | POLA1 |
|  | POLB |
|  | ACHE |
|  | CES1 |
|  | CES2 |
|  | STAT3 |
|  | PTPN6 |
|  | PTPN11 |
|  | TERT |
|  | EED |
|  | SUZ12 |
|  | EZH2 |
|  | AVPR2 |
|  | IGF1R |
|  | CYP19A1 |
|  | EGFR |
|  | F2 |
|  | PIM1 |
|  | AURKB |
|  | DRD4 |
|  | MPO |
|  | PIK3R1 |
|  | ADORA2A |
|  | DAPK1 |
|  | PYGL |
|  | CA1 |
|  | SRC |
|  | PTK2 |
|  | HSD17B2 |
|  | KDR |
|  | MMP13 |
|  | MMP3 |
|  | CA3 |
|  | ALOX15 |
|  | ABCC1 |
|  | PLK1 |
|  | CA6 |
|  | PKN1 |
|  | CA14 |
|  | CA9 |
|  | CSNK2A1 |
|  | ALOX12 |
|  | MET |
|  | NEK2 |
|  | CXCR1 |
|  | CAMK2B |
|  | ALK |
|  | AKT1 |
|  | ABCB1 |
|  | NEK6 |
|  | PLA2G1B |
|  | CA5A |
|  | BACE1 |
|  | AXL |
|  | NUAK1 |
|  | AKR1C2 |
|  | AKR1C1 |
|  | AKR1C3 |
|  | AKR1C4 |
|  | CA13 |
|  | AKR1A1 |
|  | TYR |
|  | HSD17B1 |
|  | AHR |
|  | ESRRA |
|  | KDM4E |
|  | GRK6 |
|  | DRD1 |
|  | DRD2 |
|  | HTR2B |
|  | HTR1A |
|  | HTR7 |
|  | HTR6 |
|  | HTR5A |
|  | GPR35 |
|  | ERBB2 |
|  | CCND1 |
|  | CDK4 |
|  | PDGFRB |
|  | FLT4 |
|  | INSR |
|  | CDK2 |
|  | CCNA1 |
|  | CCNA2 |
|  | PLK4 |
|  | TEK |
|  | AURKA |
|  | MAP3K8 |
|  | BRAF |
|  | EPHB4 |
|  | HSPA1A |
|  | SQLE |
|  | FGR |
|  | LYN |
|  | PREP |
|  | PTGES |
|  | SHBG |
|  | CBR1 |

**Table S5.** Common targets of different components in herbal strategy I, II, III and IV

| **Strategy** | **Component name** | **Number** | **Common target** |
| --- | --- | --- | --- |
| I | DFV quercetin | 1 | CYP19A1 |
|  | quercetin | 66 | CA2 |
|  |  |  | BACE1 |
|  |  |  | CA3 |
|  |  |  | AKR1C3 |
|  |  |  | MMP2 |
|  |  |  | CDK1 |
|  |  |  | AURKB |
|  |  |  | CSNK2A1 |
|  |  |  | PTK2 |
|  |  |  | AKT1 |
|  |  |  | NUAK1 |
|  |  |  | PIK3R1 |
|  |  |  | XDH |
|  |  |  | GSK3B |
|  |  |  | CA14 |
|  |  |  | CA4 |
|  |  |  | DAPK1 |
|  |  |  | ALOX15 |
|  |  |  | DRD4 |
|  |  |  | PYGL |
|  |  |  | HSD17B2 |
|  |  |  | KDR |
|  |  |  | F2 |
|  |  |  | CA9 |
|  |  |  | ABCG2 |
|  |  |  | CA12 |
|  |  |  | AKR1C2 |
|  |  |  | PLA2G1B |
|  |  |  | AKR1A1 |
|  |  |  | GLO1 |
|  |  |  | NEK2 |
|  |  |  | CA13 |
|  |  |  | CAMK2B |
|  |  |  | MMP13 |
|  |  |  | PLK1 |
|  |  |  | ALOX12 |
|  |  |  | CYP1B1 |
|  |  |  | EGFR |
|  |  |  | ADORA2A |
|  |  |  | IGF1R |
|  |  |  | ABCC1 |
|  |  |  | SRC |
|  |  |  | PKN1 |
|  |  |  | MMP3 |
|  |  |  | MET |
|  |  |  | NOX4 |
|  |  |  | CA1 |
|  |  |  | AKR1C1 |
|  |  |  | ABCB1 |
|  |  |  | AXL |
|  |  |  | AVPR2 |
|  |  |  | AKR1C4 |
|  |  |  | CA5A |
|  |  |  | FLT3 |
|  |  |  | PIM1 |
|  |  |  | MAOA |
|  |  |  | ALK |
|  |  |  | MPO |
|  |  |  | CA7 |
|  |  |  | ALOX5 |
|  |  |  | ADORA1 |
|  |  |  | CA6 |
|  |  |  | MMP9 |
|  |  |  | NEK6 |
|  |  |  | CXCR1 |
|  |  |  | AKR1B1 |
| II | Danshenol A Danshenol B cryptotanshinone dihydrotanshinoneⅠ kaempferol luteolin quercetin tanshinone iia | 1 | AKR1B1 |
|  | baicalein kaempferol luteolin quercetin | 1 | XDH |
|  | Miltirone cryptotanshinone dihydrotanshinoneⅠ tanshinone iia | 2 | CES1 |
|  |  |  | CES2 |
|  | kaempferol luteolin quercetin | 8 | CA2 |
|  |  |  | ABCG2 |
|  |  |  | CA12 |
|  |  |  | CYP1B1 |
|  |  |  | NOX4 |
|  |  |  | FLT3 |
|  |  |  | CA7 |
|  |  |  | ALOX5 |
|  | baicalein luteolin quercetin | 1 | CDK1 |
|  | luteolin quercetin | 7 | MMP2 |
|  |  |  | GSK3B |
|  |  |  | CA4 |
|  |  |  | GLO1 |
|  |  |  | MAOA |
|  |  |  | ADORA1 |
|  |  |  | MMP9 |
|  | cryptotanshinone dihydrotanshinoneⅠ | 1 | ACHE |
|  | dihydrotanshinoneⅠ tanshinone iia | 2 | PTPN11 |
|  |  |  | PTPN6 |
|  | kaempferol quercetin | 3 | HSD17B2 |
|  |  |  | ABCC1 |
|  |  |  | ABCB1 |
|  | baicalein quercetin | 2 | ALOX15 |
|  |  |  | ALOX12 |
|  | quercetagetin quercetin | 1 | PIM1 |
|  | luteolin | 16 | SYK |
|  |  |  | AKR1B10 |
|  |  |  | CCNB3 |
|  |  |  | CDK5R1 |
|  |  |  | ARG1 |
|  |  |  | TNKS2 |
|  |  |  | MMP12 |
|  |  |  | CCNB1 |
|  |  |  | APP |
|  |  |  | CD38 |
|  |  |  | CDK5 |
|  |  |  | TNKS |
|  |  |  | CCNB2 |
|  |  |  | PARP1 |
|  |  |  | TOP1 |
|  |  |  | TTR |
|  | digallate | 2 | POLA1 |
|  |  |  | POLB |
|  | cryptotanshinone | 1 | STAT3 |
|  | tanshinone iia | 1 | TERT |
|  | (6S)-6-(hydroxymethyl)-1,6-dimethyl-8,9-dihydro-7H-naphtho[8,7-g]benzofuran-10,11-dione | 3 | SUZ12 |
|  |  |  | EED |
|  |  |  | EZH2 |
|  | quercetin | 43 | BACE1 |
|  |  |  | CA3 |
|  |  |  | AKR1C3 |
|  |  |  | AURKB |
|  |  |  | CSNK2A1 |
|  |  |  | PTK2 |
|  |  |  | AKT1 |
|  |  |  | NUAK1 |
|  |  |  | PIK3R1 |
|  |  |  | CA14 |
|  |  |  | DAPK1 |
|  |  |  | DRD4 |
|  |  |  | CYP19A1 |
|  |  |  | PYGL |
|  |  |  | KDR |
|  |  |  | F2 |
|  |  |  | CA9 |
|  |  |  | AKR1C2 |
|  |  |  | PLA2G1B |
|  |  |  | AKR1A1 |
|  |  |  | NEK2 |
|  |  |  | CA13 |
|  |  |  | CAMK2B |
|  |  |  | MMP13 |
|  |  |  | PLK1 |
|  |  |  | EGFR |
|  |  |  | ADORA2A |
|  |  |  | IGF1R |
|  |  |  | PKN1 |
|  |  |  | SRC |
|  |  |  | MMP3 |
|  |  |  | MET |
|  |  |  | AKR1C1 |
|  |  |  | CA1 |
|  |  |  | AXL |
|  |  |  | AVPR2 |
|  |  |  | AKR1C4 |
|  |  |  | CA5A |
|  |  |  | ALK |
|  |  |  | MPO |
|  |  |  | CA6 |
|  |  |  | NEK6 |
|  |  |  | CXCR1 |
|  | kaempferol | 4 | TYR |
|  |  |  | HSD17B1 |
|  |  |  | ESRRA |
|  |  |  | AHR |
|  | baicalein | 2 | GRK6 |
|  |  |  | KDM4E |
| III | Danshenol A Danshenol B cryptotanshinone dihydrotanshinoneⅠ kaempferol luteolin quercetin tanshinone iia | 1 | AKR1B1 |
|  | baicalein kaempferol luteolin quercetin | 1 | XDH |
|  | Miltirone cryptotanshinone dihydrotanshinoneⅠ tanshinone iia | 2 | CES1 |
|  |  |  | CES2 |
|  | kaempferol luteolin quercetin | 8 | CA2 |
|  |  |  | ABCG2 |
|  |  |  | CA12 |
|  |  |  | CYP1B1 |
|  |  |  | NOX4 |
|  |  |  | FLT3 |
|  |  |  | CA7 |
|  |  |  | ALOX5 |
|  | baicalein luteolin quercetin | 1 | CDK1 |
|  | luteolin quercetin | 7 | MMP2 |
|  |  |  | GSK3B |
|  |  |  | CA4 |
|  |  |  | GLO1 |
|  |  |  | MAOA |
|  |  |  | ADORA1 |
|  |  |  | MMP9 |
|  | cryptotanshinone dihydrotanshinoneⅠ | 1 | ACHE |
|  | dihydrotanshinoneⅠ tanshinone iia | 2 | PTPN11 |
|  |  |  | PTPN6 |
|  | kaempferol quercetin | 3 | HSD17B2 |
|  |  |  | ABCC1 |
|  |  |  | ABCB1 |
|  | baicalein quercetin | 2 | ALOX15 |
|  |  |  | ALOX12 |
|  | quercetagetin quercetin | 1 | PIM1 |
|  | luteolin | 16 | SYK |
|  |  |  | AKR1B10 |
|  |  |  | CCNB3 |
|  |  |  | CDK5R1 |
|  |  |  | ARG1 |
|  |  |  | TNKS2 |
|  |  |  | MMP12 |
|  |  |  | CCNB1 |
|  |  |  | APP |
|  |  |  | CD38 |
|  |  |  | CDK5 |
|  |  |  | TNKS |
|  |  |  | CCNB2 |
|  |  |  | PARP1 |
|  |  |  | TOP1 |
|  |  |  | TTR |
|  | digallate | 2 | POLA1 |
|  |  |  | POLB |
|  | cryptotanshinone | 1 | STAT3 |
|  | tanshinone iia | 1 | TERT |
|  | (6S)-6-(hydroxymethyl)-1,6-dimethyl-8,9-dihydro-7H-naphtho[8,7-g]benzofuran-10,11-dione | 3 | SUZ12 |
|  |  |  | EED |
|  |  |  | EZH2 |
|  | quercetin | 43 | BACE1 |
|  |  |  | CA3 |
|  |  |  | AKR1C3 |
|  |  |  | AURKB |
|  |  |  | CSNK2A1 |
|  |  |  | PTK2 |
|  |  |  | AKT1 |
|  |  |  | NUAK1 |
|  |  |  | PIK3R1 |
|  |  |  | CA14 |
|  |  |  | DAPK1 |
|  |  |  | DRD4 |
|  |  |  | CYP19A1 |
|  |  |  | PYGL |
|  |  |  | KDR |
|  |  |  | F2 |
|  |  |  | CA9 |
|  |  |  | AKR1C2 |
|  |  |  | PLA2G1B |
|  |  |  | AKR1A1 |
|  |  |  | NEK2 |
|  |  |  | CA13 |
|  |  |  | CAMK2B |
|  |  |  | MMP13 |
|  |  |  | PLK1 |
|  |  |  | EGFR |
|  |  |  | ADORA2A |
|  |  |  | IGF1R |
|  |  |  | PKN1 |
|  |  |  | SRC |
|  |  |  | MMP3 |
|  |  |  | MET |
|  |  |  | AKR1C1 |
|  |  |  | CA1 |
|  |  |  | AXL |
|  |  |  | AVPR2 |
|  |  |  | AKR1C4 |
|  |  |  | CA5A |
|  |  |  | ALK |
|  |  |  | MPO |
|  |  |  | CA6 |
|  |  |  | NEK6 |
|  |  |  | CXCR1 |
|  | kaempferol | 4 | TYR |
|  |  |  | HSD17B1 |
|  |  |  | ESRRA |
|  |  |  | AHR |
|  | baicalein | 2 | GRK6 |
|  |  |  | KDM4E |
| IV | Danshenol A Danshenol B cryptotanshinone dihydrotanshinoneⅠ ellagic acid kaempferol luteolin quercetin tanshinone iia | 1 | AKR1B1 |
|  | Chryseriol isorhamnetin kaempferol luteolin naringenin quercetin | 1 | CYP1B1 |
|  | ellagic acid isorhamnetin kaempferol luteolin naringenin quercetin | 2 | CA12 |
|  |  |  | CA7 |
|  | ellagic acid isorhamnetin kaempferol luteolin quercetin | 1 | CA2 |
|  | baicalein isorhamnetin kaempferol luteolin quercetin | 1 | XDH |
|  | ellagic acid isorhamnetin luteolin naringenin quercetin | 1 | CA4 |
|  | Sinensetin kaempferol luteolin nobiletin quercetin | 1 | ABCG2 |
|  | Miltirone cryptotanshinone dihydrotanshinoneⅠ tanshinone iia | 2 | CES1 |
|  |  |  | CES2 |
|  | Chryseriol kaempferol naringenin quercetin | 1 | ABCC1 |
|  | kaempferol luteolin quercetin | 3 | ALOX5 |
|  |  |  | NOX4 |
|  |  |  | FLT3 |
|  | ellagic acid luteolin quercetin | 1 | GSK3B |
|  | baicalein luteolin quercetin | 1 | CDK1 |
|  | cryptotanshinone dihydrotanshinoneⅠ palmatine | 1 | ACHE |
|  | luteolin quercetin | 5 | MMP2 |
|  |  |  | GLO1 |
|  |  |  | MAOA |
|  |  |  | ADORA1 |
|  |  |  | MMP9 |
|  | dihydrotanshinoneⅠ tanshinone iia | 2 | PTPN11 |
|  |  |  | PTPN6 |
|  | quercetagetin quercetin | 1 | PIM1 |
|  | kaempferol quercetin | 2 | HSD17B2 |
|  |  |  | ABCB1 |
|  | ellagic acid quercetin | 18 | AURKB |
|  |  |  | PTK2 |
|  |  |  | NUAK1 |
|  |  |  | KDR |
|  |  |  | CA13 |
|  |  |  | IGF1R |
|  |  |  | MET |
|  |  |  | CA1 |
|  |  |  | CA6 |
|  |  |  | BACE1 |
|  |  |  | CSNK2A1 |
|  |  |  | AKT1 |
|  |  |  | CA14 |
|  |  |  | CA9 |
|  |  |  | PLK1 |
|  |  |  | EGFR |
|  |  |  | SRC |
|  |  |  | CA5A |
|  | baicalein quercetin | 2 | ALOX12 |
|  |  |  | ALOX15 |
|  | naringenin quercetin | 1 | CYP19A1 |
|  | kaempferol naringenin | 1 | HSD17B1 |
|  | Isocorypalmine N-methyllaurotetanine | 1 | DRD1 |
|  | luteolin | 16 | SYK |
|  |  |  | TNKS2 |
|  |  |  | CCNB1 |
|  |  |  | CDK5 |
|  |  |  | TTR |
|  |  |  | AKR1B10 |
|  |  |  | CCNB3 |
|  |  |  | CDK5R1 |
|  |  |  | ARG1 |
|  |  |  | MMP12 |
|  |  |  | APP |
|  |  |  | CD38 |
|  |  |  | TNKS |
|  |  |  | CCNB2 |
|  |  |  | PARP1 |
|  |  |  | TOP1 |
|  | digallate | 2 | POLA1 |
|  |  |  | POLB |
|  | cryptotanshinone | 1 | STAT3 |
|  | tanshinone iia | 1 | TERT |
|  | (6S)-6-(hydroxymethyl)-1,6-dimethyl-8,9-dihydro-7H-naphtho[8,7-g]benzofuran-10,11-dione | 3 | SUZ12 |
|  |  |  | EED |
|  |  |  | EZH2 |
|  | quercetin | 24 | CA3 |
|  |  |  | AKR1C3 |
|  |  |  | AKR1A1 |
|  |  |  | CAMK2B |
|  |  |  | MMP13 |
|  |  |  | ADORA2A |
|  |  |  | MMP3 |
|  |  |  | AKR1C1 |
|  |  |  | AXL |
|  |  |  | AVPR2 |
|  |  |  | AKR1C4 |
|  |  |  | MPO |
|  |  |  | CXCR1 |
|  |  |  | PIK3R1 |
|  |  |  | DAPK1 |
|  |  |  | DRD4 |
|  |  |  | PYGL |
|  |  |  | F2 |
|  |  |  | AKR1C2 |
|  |  |  | PLA2G1B |
|  |  |  | NEK2 |
|  |  |  | PKN1 |
|  |  |  | ALK |
|  |  |  | NEK6 |
|  | kaempferol | 3 | TYR |
|  |  |  | ESRRA |
|  |  |  | AHR |
|  | Isocorypalmine | 1 | DRD2 |
|  | N-methyllaurotetanine | 5 | HTR5A |
|  |  |  | HTR6 |
|  |  |  | HTR1A |
|  |  |  | HTR2B |
|  |  |  | HTR7 |
|  | ellagic acid | 20 | CDK4 |
|  |  |  | CCNA1 |
|  |  |  | PDGFRB |
|  |  |  | INSR |
|  |  |  | MAP3K8 |
|  |  |  | FGR |
|  |  |  | GPR35 |
|  |  |  | PLK4 |
|  |  |  | CCNA2 |
|  |  |  | FLT4 |
|  |  |  | LYN |
|  |  |  | BRAF |
|  |  |  | TEK |
|  |  |  | EPHB4 |
|  |  |  | SQLE |
|  |  |  | AURKA |
|  |  |  | CDK2 |
|  |  |  | CCND1 |
|  |  |  | ERBB2 |
|  |  |  | HSPA1A |
|  | baicalein | 2 | GRK6 |
|  |  |  | KDM4E |
|  | Boswellic acid | 2 | PREP |
|  |  |  | PTGES |
|  | naringenin | 2 | SHBG |
|  |  |  | CBR1 |

**Table S6.** Common and unique target in four herbal strategies

| **Strategy** | **Number** | **Target** |
| --- | --- | --- |
| I/II/III/IV | 18 | MMP2 |
|  |  | CDK1 |
|  |  | XDH |
|  |  | ABCG2 |
|  |  | GLO1 |
|  |  | ALOX5 |
|  |  | AKR1B1 |
|  |  | CA2 |
|  |  | GSK3B |
|  |  | CA4 |
|  |  | CA12 |
|  |  | CYP1B1 |
|  |  | NOX4 |
|  |  | FLT3 |
|  |  | MAOA |
|  |  | CA7 |
|  |  | ADORA1 |
|  |  | MMP9 |
| I/III/IV | 49 | CA3 |
|  |  | AKR1C3 |
|  |  | AURKB |
|  |  | PTK2 |
|  |  | NUAK1 |
|  |  | CYP19A1 |
|  |  | HSD17B2 |
|  |  | KDR |
|  |  | AKR1A1 |
|  |  | CA13 |
|  |  | CAMK2B |
|  |  | MMP13 |
|  |  | ALOX12 |
|  |  | ADORA2A |
|  |  | IGF1R |
|  |  | MMP3 |
|  |  | MET |
|  |  | AKR1C1 |
|  |  | CA1 |
|  |  | AXL |
|  |  | AVPR2 |
|  |  | AKR1C4 |
|  |  | MPO |
|  |  | CA6 |
|  |  | CXCR1 |
|  |  | BACE1 |
|  |  | CSNK2A1 |
|  |  | AKT1 |
|  |  | PIK3R1 |
|  |  | CA14 |
|  |  | DAPK1 |
|  |  | ALOX15 |
|  |  | DRD4 |
|  |  | PYGL |
|  |  | F2 |
|  |  | CA9 |
|  |  | AKR1C2 |
|  |  | PLA2G1B |
|  |  | NEK2 |
|  |  | PLK1 |
|  |  | EGFR |
|  |  | ABCC1 |
|  |  | PKN1 |
|  |  | SRC |
|  |  | ABCB1 |
|  |  | CA5A |
|  |  | PIM1 |
|  |  | ALK |
|  |  | NEK6 |
| II/III/IV | 28 | SYK |
|  |  | POLA1 |
|  |  | TNKS2 |
|  |  | CCNB1 |
|  |  | CDK5 |
|  |  | TTR |
|  |  | AKR1B10 |
|  |  | ACHE |
|  |  | CCNB3 |
|  |  | STAT3 |
|  |  | SUZ12 |
|  |  | CDK5R1 |
|  |  | TERT |
|  |  | EED |
|  |  | CES1 |
|  |  | PTPN11 |
|  |  | ARG1 |
|  |  | POLB |
|  |  | MMP12 |
|  |  | CES2 |
|  |  | APP |
|  |  | PTPN6 |
|  |  | CD38 |
|  |  | TNKS |
|  |  | EZH2 |
|  |  | CCNB2 |
|  |  | PARP1 |
|  |  | TOP1 |
| III/IV | 6 | TYR |
|  |  | HSD17B1 |
|  |  | ESRRA |
|  |  | GRK6 |
|  |  | AHR |
|  |  | KDM4E |
| Ⅳ | 31 | CDK4 |
|  |  | SHBG |
|  |  | PREP |
|  |  | CCNA1 |
|  |  | HTR5A |
|  |  | HTR6 |
|  |  | PDGFRB |
|  |  | INSR |
|  |  | HTR1A |
|  |  | MAP3K8 |
|  |  | FGR |
|  |  | GPR35 |
|  |  | DRD2 |
|  |  | PLK4 |
|  |  | CCNA2 |
|  |  | HTR2B |
|  |  | FLT4 |
|  |  | LYN |
|  |  | PTGES |
|  |  | BRAF |
|  |  | TEK |
|  |  | EPHB4 |
|  |  | SQLE |
|  |  | AURKA |
|  |  | HTR7 |
|  |  | CDK2 |
|  |  | CCND1 |
|  |  | CBR1 |
|  |  | DRD1 |
|  |  | ERBB2 |
|  |  | HSPA1A |

**Table S7.** GO and KEGG pathway enrichment analyses of targets in four herbal strategies

| **Type** | **Strategy** | **Term** | **FDR** | **Member** |
| --- | --- | --- | --- | --- |
| molecular function | I | carbonate dehydratase activity | 5.18E-19 | CA14, CA9, CA13, CA12, CA7, CA5A, CA6, CA4, CA3, CA2, CA1 |
|  |  | protein kinase activity | 7.93E-09 | EGFR, CDK1, NEK2, NUAK1, MET, PKN1, AURKB, SRC, DAPK1, AKT1, PTK2, CSNK2A1, PLK1, GSK3B, CAMK2B, NEK6 |
|  |  | ATP binding | 1.49E-07 | NUAK1, NEK2, AURKB, SRC, AKT1, IGF1R, PTK2, CSNK2A1, CAMK2B, EGFR, CDK1, FLT3, MET, PIM1, AXL, PKN1, ABCB1, ALK, KDR, ABCG2, DAPK1, PYGL, PLK1, GSK3B, ABCC1, NEK6 |
|  |  | transmembrane receptor protein tyrosine kinase activity | 9.10E-06 | EGFR, IGF1R, FLT3, MET, AXL, ALK, KDR |
|  |  | alditol:NADP+ 1-oxidoreductase activity | 9.50E-06 | AKR1C3, AKR1C2, AKR1A1, AKR1B1, AKR1C1 |
|  |  | protein serine/threonine kinase activity | 2.68E-05 | AKT1, CDK1, CSNK2A1, NUAK1, PLK1, NEK2, GSK3B, PIM1, PKN1, CAMK2B, AURKB, NEK6, DAPK1 |
|  |  | aldo-keto reductase (NADP) activity | 3.62E-04 | AKR1C3, AKR1C4, AKR1A1, AKR1B1, AKR1C1 |
|  |  | protein tyrosine kinase activity | 0.001092736 | EGFR, IGF1R, PTK2, MET, AXL, ALK, SRC, KDR |
|  |  | bile acid binding | 0.004049615 | AKR1C2, PYGL, PLA2G1B, AKR1C1 |
|  |  | oxidoreductase activity, acting on NAD(P)H, quinone or similar compound as acceptor | 0.004049615 | AKR1C3, AKR1C2, AKR1C4, AKR1C1 |
|  |  | oxidoreductase activity | 0.016337041 | NOX4, AKR1C3, AKR1C2, HSD17B2, AKR1A1, MAOA, AKR1B1, AKR1C1 |
|  |  | electron carrier activity | 0.03420838 | NOX4, XDH, AKR1C4, AKR1A1, AKR1B1, CYP19A1 |
|  | II | protein kinase binding | 6.70E-04 | CCNB1, PTPN6, CCNB3, CDK5R1, GSK3B, POLA1, PARP1, CDK5, STAT3, SYK |
|  |  | cyclin-dependent protein serine/threonine kinase activity | 0.002413409 | CCNB1, CDK1, CDK5R1, CCNB2, CDK5 |
|  |  | carbonate dehydratase activity | 0.007905202 | CA12, CA7, CA4, CA2 |
|  | III | carbonate dehydratase activity | 4.46E-17 | CA14, CA9, CA13, CA12, CA7, CA5A, CA6, CA4, CA3, CA2, CA1 |
|  |  | protein kinase activity | 4.42E-09 | EGFR, CDK1, CDK5R1, NEK2, NUAK1, MET, PKN1, AURKB, CDK5, SRC, DAPK1, AKT1, PTK2, CSNK2A1, PLK1, GSK3B, CAMK2B, NEK6, SYK |
|  |  | aldo-keto reductase (NADP) activity | 2.57E-05 | AKR1C3, AKR1C4, AKR1A1, AKR1B10, AKR1B1, AKR1C1 101 |
|  |  | ATP binding | 3.28E-05 | NUAK1, NEK2, AURKB, SRC, AKT1, IGF1R, PTK2, CSNK2A1, CAMK2B, SYK, EGFR, CDK1, FLT3, MET, PIM1, AXL, PKN1, ABCB1, ALK, CDK5, KDR, ABCG2, DAPK1, PYGL, PLK1, GSK3B, GRK6, ABCC1, NEK6 |
|  |  | alditol:NADP+ 1-oxidoreductase activity | 5.44E-05 | AKR1C3, AKR1C2, AKR1A1, AKR1B1, AKR1C1 |
|  |  | protein serine/threonine kinase activity | 6.16E-05 | CDK1, NEK2, NUAK1, PIM1, PKN1, AURKB, CDK5, DAPK1, AKT1, CSNK2A1, PLK1, GSK3B, CAMK2B, NEK6, SYK |
|  |  | transmembrane receptor protein tyrosine kinase activity | 1.20E-04 | EGFR, IGF1R, FLT3, MET, AXL, ALK, KDR |
|  |  | protein kinase binding | 4.54E-04 | EGFR, PTPN6, CDK5R1, POLA1, CDK5, STAT3, CCNB1, CCNB3, PTK2, PLK1, GSK3B, PARP1, NEK6, SYK |
|  |  | protein tyrosine kinase activity | 0.00165432 | EGFR, IGF1R, PTK2, MET, AXL, ALK, SRC, KDR, SYK |
|  |  | oxidoreductase activity, acting on NAD(P)H, quinone or similar compound as acceptor | 0.015026783 | AKR1C3, AKR1C2, AKR1C4, AKR1C1 |
|  |  | bile acid binding | 0.015026783 | AKR1C2, PYGL, PLA2G1B, AKR1C1 |
|  |  | kinase activity | 0.017745093 | AKT1, CDK5R1, CSNK2A1, PLK1, GSK3B, ALK, CDK5, PIK3R1, SRC, DAPK1 |
|  |  | oxidoreductase activity | 0.033613782 | NOX4, AKR1C3, AKR1C2, TYR, HSD17B2, AKR1A1, MAOA, AKR1B1, AKR1C1 |
|  | IV | carbonate dehydratase activity | 7.51881E-16 | CA14, CA9, CA13, CA12, CA7, CA5A, CA6, CA4, CA3, CA2, CA1 |
|  |  | protein kinase activity | 4.39014E-15 | EGFR, CDK1, CDK5R1, BRAF, NUAK1, NEK2, ERBB2, MET, PKN1, AURKA, AURKB, CDK4, CDK5, SRC, CDK2, DAPK1, AKT1, PTK2, CCND1, CSNK2A1, PLK1, GSK3B, MAP3K8, TEK, CAMK2B, NEK6, SYK |
|  |  | transmembrane receptor protein tyrosine kinase activity | 5.68434E-12 | EGFR, IGF1R, FLT3, FLT4, ERBB2, TEK, MET, AXL, ALK, INSR, EPHB4, KDR |
|  |  | ATP binding | 1.39888E-11 | FGR, NEK2, NUAK1, ERBB2, HSPA1A, AURKA, AURKB, EPHB4, SRC, AKT1, IGF1R, PTK2, CSNK2A1, MAP3K8, TEK, CAMK2B, INSR, SYK, EGFR, CDK1, LYN, BRAF, FLT3, FLT4, MET, AXL, PIM1, PKN1, ABCB1, ALK, CDK4, CDK5, CDK2, KDR, DAPK1, ABCG2, PLK4, PLK1, PYGL, GSK3B, GRK6, PDGFRB, ABCC1, NEK6 |
|  |  | protein tyrosine kinase activity | 2.74828E-09 | EGFR, FGR, LYN, ERBB2, MET, AXL, ALK, SRC, KDR, IGF1R, PTK2, TEK, PDGFRB, INSR, SYK |
|  |  | protein serine/threonine kinase activity | 1.56531E-08 | CDK1, BRAF, NEK2, NUAK1, PIM1, PKN1, AURKA, AURKB, CDK4, CDK5, CDK2, DAPK1, AKT1, PLK4, CSNK2A1, PLK1, GSK3B, MAP3K8, CAMK2B, NEK6, SYK |
|  |  | protein kinase binding | 1.00505E-06 | EGFR, PTPN6, CDK5R1, FGR, POLA1, AURKA, CDK5, STAT3, CCNB1, CCNB3, PTK2, CCND1, PLK1, GSK3B, PDGFRB, PARP1, CCNA2, NEK6, SYK |
|  |  | receptor signaling protein tyrosine kinase activity | 8.81444E-06 | EGFR, LYN, ERBB2, INSR, KDR, SYK |
|  |  | aldo-keto reductase (NADP) activity | 0.000101814 | AKR1C3, AKR1C4, AKR1A1, AKR1B10, AKR1B1, AKR1C1 |
|  |  | alditol:NADP+ 1-oxidoreductase activity | 0.00016483 | AKR1C3, AKR1C2, AKR1A1, AKR1B1, AKR1C1 |
|  |  | cyclin-dependent protein serine/threonine kinase activity | 0.000303227 | CCNB1, CDK1, CDK5R1, CCNB2, CDK4, CDK5, CDK2 |
|  |  | oxidoreductase activity, acting on NAD(P)H, quinone or similar compound as acceptor | 0.000327677 | AKR1C3, AKR1C2, CBR1, AKR1C4, AKR1C1 |
|  |  | kinase activity | 0.000534849 | AKT1, CDK5R1, CSNK2A1, BRAF, PLK1, GSK3B, ERBB2, ALK, CDK5, PIK3R1, SRC, CDK2, DAPK1 |
|  |  | protein binding | 0.009780168 | NUAK1, MMP9, EZH2, AURKA, AURKB, MMP3, MMP2, ADORA1, AKT1, TOP1, TTR, TYR, APP, CSNK2A1, HTR1A, MAP3K8, EED, CCNA1, INSR, CCNA2, TERT, AKR1C1, SYK, EGFR, CDK1, BRAF, LYN, PIM1, PKN1, POLB, ALK, CDK4, CDK5, CDK2, AHR, TNKS2, DAPK1, SUZ12, ALOX15, CCND1, AKR1B10, F2, HTR6, BACE1, CA4, PDGFRB, CA2, CA1, NEK6, ALOX12, PREP, AVPR2, CDK5R1, DRD1, ACHE, FGR, DRD2, ADORA2A, NEK2, ERBB2, DRD4, POLA1, HSPA1A, EPHB4, SRC, IGF1R, PTK2, AKR1A1, TEK, TNKS, CAMK2B, PIK3R1, NOX4, PTPN6, ESRRA, FLT3, FLT4, MET, AXL, ABCB1, STAT3, ABCG2, PTPN11, KDR, CCNB1, CCNB3, PLK4, CCNB2, PYGL, PLK1, GSK3B, GRK6, ALOX5, PARP1 |
|  |  | protein phosphatase binding | 0.012743272 | EGFR, NEK2, FLT4, ERBB2, MET, PIK3R1, STAT3 |
|  |  | enzyme binding | 0.015323608 | AKT1, EGFR, APP, CCND1, LYN, ADORA2A, BACE1, PDGFRB, POLB, HSPA1A, PARP1, SRC, TNKS2 |
|  |  | phosphatidylinositol 3-kinase binding | 0.016978739 | IGF1R, AXL, PDGFRB, INSR, PIK3R1 |
|  |  | bile acid binding | 0.034410146 | AKR1C2, PYGL, PLA2G1B, AKR1C1 |
|  |  | oxidoreductase activity | 0.036180489 | AKR1C3, NOX4, AKR1C2, TYR, HSD17B2, AKR1A1, SQLE, MAOA, AKR1B1, AKR1C1 |
| biological process | I | bicarbonate transport | 1.58E-12 | CA14, CA9, CA13, CA12, CA7, CA5A, CA6, CA4, CA3, CA2, CA1 |
|  |  | one-carbon metabolic process | 2.62E-12 | CA9, CA13, CA12, CA7, CA5A, CA6, CA4, CA3, CA2, CA1 |
|  |  | protein autophosphorylation | 6.28E-12 | EGFR, NEK2, FLT3, PIM1, AURKB, ALK, SRC, KDR, DAPK1, AKT1, IGF1R, PTK2, GSK3B, CAMK2B, NEK6 |
|  |  | negative regulation of apoptotic process | 2.95E-07 | EGFR, CDK1, MMP9, PIM1, ADORA1, SRC, KDR, AKT1, IGF1R, PTK2, PLK1, GSK3B, MPO, GLO1, PIK3R1, ALOX12 |
|  |  | doxorubicin metabolic process | 2.37E-05 | AKR1C3, AKR1C2, AKR1C4, AKR1B1, AKR1C1 |
|  |  | daunorubicin metabolic process | 2.37E-05 | AKR1C3, AKR1C2, AKR1C4, AKR1B1, AKR1C1 |
|  |  | oxidation-reduction process | 8.82E-05 | XDH, NOX4, CYP1B1, HSD17B2, MAOA, AKR1C3, AKR1C2, ALOX15, AKR1C4, AKR1A1, AKR1B1, MPO, ALOX5, AKR1C1, CYP19A1 |
|  |  | protein phosphorylation | 2.85E-04 | AKT1, CSNK2A1, NUAK1, PLK1, NEK2, GSK3B, PIM1, PKN1, CAMK2B, AURKB, PIK3R1, NEK6, DAPK1 |
|  |  | cellular response to jasmonic acid stimulus | 3.64E-04 | AKR1C3, AKR1C2, AKR1C4, AKR1C1 |
|  |  | positive regulation of protein kinase B signaling | 0.001469521 | NOX4, AKR1C3, EGFR, AKR1C2, PTK2, AXL, SRC |
|  |  | positive regulation of protein localization to nucleus | 0.001947638 | AKT1, CDK1, PLK1, F2, SRC |
|  |  | peptidyl-tyrosine phosphorylation | 0.003546609 | EGFR, PTK2, FLT3, MET, AXL, ALK, SRC, KDR |
|  |  | peptidyl-serine phosphorylation | 0.014954702 | AKT1, CDK1, PLK1, GSK3B, PKN1, SRC, NEK6 |
|  |  | positive regulation of cell proliferation | 0.020283208 | AKR1C3, EGFR, AVPR2, IGF1R, AKR1C2, PTK2, CSNK2A1, FLT3, F2, KDR, ALOX12 |
|  | II | peptidyl-threonine phosphorylation | 0.004777698 | CDK1, CDK5R1, GSK3B, TNKS, CDK5 |
|  |  | cellular response to hydrogen peroxide | 0.024633564 | CDK1, ARG1, CYP1B1, AKR1B1, EZH2 |
|  |  | peptidyl-serine phosphorylation | 0.031212298 | CDK1, CDK5R1, GSK3B, TNKS, CDK5, SYK |
|  | III | protein autophosphorylation | 7.65E-12 | EGFR, NEK2, FLT3, PIM1, AURKB, ALK, CDK5, SRC, KDR, DAPK1, AKT1, IGF1R, PTK2, GSK3B, CAMK2B, NEK6, SYK |
|  |  | bicarbonate transport | 1.22E-10 | CA14, CA9, CA13, CA12, CA7, CA5A, CA6, CA4, CA3, CA2, CA1 |
|  |  | one-carbon metabolic process | 1.38E-10 | CA9, CA13, CA12, CA7, CA5A, CA6, CA4, CA3, CA2, CA1 |
|  |  | daunorubicin metabolic process | 6.13E-07 | AKR1C3, AKR1C2, AKR1C4, AKR1B10, AKR1B1, AKR1C1 |
|  |  | doxorubicin metabolic process | 6.13E-07 | AKR1C3, AKR1C2, AKR1C4, AKR1B10, AKR1B1, AKR1C1 |
|  |  | cell proliferation | 7.83E-07 | EGFR, PTPN6, CDK1, CDK5R1, ACHE, MET, POLA1, PIM1, AURKB, ALK, CDK5, STAT3, SRC, AKT1, TYR, PLK1, SYK |
|  |  | negative regulation of apoptotic process | 2.35E-06 | EGFR, CDK1, MMP9, PIM1, ADORA1, STAT3, SRC, KDR, AKT1, IGF1R, CD38, PTK2, PLK1, GSK3B, MPO, GLO1, PIK3R1, ALOX12 |
|  |  | peptidyl-serine phosphorylation | 5.86E-06 | AKT1, CDK1, CDK5R1, PLK1, GSK3B, PKN1, TNKS, CDK5, SRC, NEK6, SYK |
|  |  | oxidation-reduction process | 1.86E-05 | NOX4, XDH, CYP1B1, HSD17B2, HSD17B1, MAOA, AKR1C3, AKR1C2, ALOX15, TYR, AKR1C4, AKR1A1, AKR1B10, AKR1B1, MPO, ALOX5, AKR1C1, CYP19A1, KDM4E |
|  |  | protein phosphorylation | 1.29E-04 | NEK2, NUAK1, PIM1, PKN1, AURKB, DAPK1, AKT1, APP, CSNK2A1, PLK1, GSK3B, GRK6, CAMK2B, NEK6, PIK3R1, SYK |
|  |  | cellular response to jasmonic acid stimulus | 0.001338757 | AKR1C3, AKR1C2, AKR1C4, AKR1C1 |
|  |  | response to drug | 0.003174498 | CCNB1, CDK1, ARG1, TOP1, CD38, CA9, ADORA2A, ABCC1, ABCB1, SRC, STAT3, ABCG2 |
|  |  | peptidyl-threonine phosphorylation | 0.004770231 | AKT1, CDK1, CDK5R1, GSK3B, TNKS, CDK5 |
|  |  | peptidyl-tyrosine phosphorylation | 0.00594142 | EGFR, PTPN6, PTK2, FLT3, MET, AXL, ALK, SRC, KDR |
|  |  | positive regulation of cell proliferation | 0.006347225 | EGFR, AVPR2, PTPN6, FLT3, STAT3, KDR, SUZ12, AKR1C3, IGF1R, AKR1C2, PTK2, CSNK2A1, F2, ALOX12 |
|  |  | platelet activation | 0.009129797 | AKT1, PTPN6, F2, AXL, PIK3R1, SRC, SYK, PTPN11 |
|  |  | positive regulation of protein localization to nucleus | 0.010752413 | AKT1, CDK1, PLK1, F2, SRC |
|  |  | positive regulation of protein kinase B signaling | 0.017564641 | NOX4, AKR1C3, EGFR, AKR1C2, PTK2, AXL, SRC |
|  |  | cellular response to hydrogen peroxide | 0.036380884 | CDK1, ARG1, CYP1B1, AKR1B1, EZH2, AXL |
|  |  | transmembrane receptor protein tyrosine kinase signaling pathway | 0.037817773 | EGFR, IGF1R, FLT3, MET, ALK, KDR, SYK |
|  | IV | protein autophosphorylation | 7.58637E-22 | FGR, NEK2, ERBB2, AURKA, AURKB, EPHB4, SRC, AKT1, IGF1R, PTK2, TEK, CAMK2B, INSR, SYK, EGFR, LYN, FLT3, FLT4, PIM1, ALK, CDK5, KDR, DAPK1, GSK3B, PDGFRB, NEK6 |
|  |  | peptidyl-tyrosine phosphorylation | 8.51097E-11 | EGFR, PTPN6, FGR, LYN, FLT3, FLT4, ERBB2, MET, AXL, ALK, EPHB4, SRC, KDR, PTK2, TEK, PDGFRB, INSR |
|  |  | protein phosphorylation | 1.52145E-10 | BRAF, FGR, LYN, NEK2, NUAK1, ERBB2, PIM1, PKN1, AURKA, AURKB, CDK4, DAPK1, AKT1, CCND1, PLK4, APP, CSNK2A1, PLK1, GSK3B, MAP3K8, GRK6, CAMK2B, NEK6, PIK3R1, SYK |
|  |  | negative regulation of apoptotic process | 1.22965E-09 | EGFR, CDK1, BRAF, FLT4, MMP9, PIM1, AURKA, ADORA1, STAT3, SRC, KDR, AKT1, IGF1R, CD38, PTK2, PLK1, GSK3B, TEK, MPO, PDGFRB, GLO1, HTR2B, PIK3R1, ALOX12 |
|  |  | one-carbon metabolic process | 1.57883E-09 | CA9, CA13, CA12, CA7, CA5A, CA6, CA4, CA3, CA2, CA1 |
|  |  | bicarbonate transport | 1.81328E-09 | CA14, CA9, CA13, CA12, CA7, CA5A, CA6, CA4, CA3, CA2, CA1 |
|  |  | transmembrane receptor protein tyrosine kinase signaling pathway | 1.6713E-08 | EGFR, FGR, LYN, FLT3, ERBB2, FLT4, MET, ALK, KDR, IGF1R, TEK, INSR, SYK |
|  |  | positive regulation of cell proliferation | 1.17188E-07 | EGFR, PTPN6, AVPR2, LYN, FLT3, FLT4, CDK4, CDK2, STAT3, KDR, AKR1C3, SUZ12, IGF1R, AKR1C2, PTK2, HTR1A, CSNK2A1, F2, PDGFRB, HTR2B, INSR, ALOX12 |
|  |  | response to drug | 3.34117E-07 | CDK1, DRD1, LYN, DRD2, ADORA2A, ABCB1, CDK4, STAT3, SRC, ABCG2, CCNB1, CD38, TOP1, ARG1, CCND1, CA9, ABCC1, HTR2B |
|  |  | cell proliferation | 7.55863E-07 | EGFR, PTPN6, CDK1, CDK5R1, ACHE, ERBB2, MET, POLA1, PIM1, AURKB, ALK, CDK5, STAT3, SRC, AKT1, TYR, HTR1A, PLK1, SYK |
|  |  | daunorubicin metabolic process | 2.37341E-06 | AKR1C3, AKR1C2, AKR1C4, AKR1B10, AKR1B1, AKR1C1 |
|  |  | doxorubicin metabolic process | 2.37341E-06 | AKR1C3, AKR1C2, AKR1C4, AKR1B10, AKR1B1, AKR1C1 |
|  |  | peptidyl-serine phosphorylation | 5.74865E-06 | AKT1, CDK1, CDK5R1, PLK1, GSK3B, PKN1, TNKS, CDK5, SRC, NEK6, CDK2, SYK |
|  |  | peptidyl-tyrosine autophosphorylation | 3.58194E-05 | IGF1R, PTK2, LYN, FGR, INSR, SRC, KDR, SYK |
|  |  | oxidation-reduction process | 4.80538E-05 | NOX4, XDH, CYP1B1, HSD17B2, HSD17B1, MAOA, AKR1C3, AKR1C2, CBR1, ALOX15, TYR, AKR1C4, AKR1A1, SQLE, AKR1B10, AKR1B1, MPO, ALOX5, AKR1C1, CYP19A1, KDM4E |
|  |  | positive regulation of protein phosphorylation | 8.89475E-05 | AKT1, EGFR, PTK2, CCND1, FLT4, MMP9, ERBB2, F2, TEK, INSR, KDR |
|  |  | positive regulation of ERK1 and ERK2 cascade | 0.000191697 | NOX4, EGFR, ALOX15, BRAF, DRD2, FLT4, TEK, PDGFRB, HTR2B, SRC, KDR, PTPN11 |
|  |  | positive regulation of phosphatidylinositol 3-kinase activity | 0.000202012 | PTK2, LYN, FGR, FLT3, TEK, PDGFRB, SRC |
|  |  | positive regulation of protein kinase B signaling | 0.000463893 | AKR1C3, NOX4, EGFR, AKR1C2, PTK2, TEK, AXL, INSR, SRC |
|  |  | positive regulation of MAP kinase activity | 0.000580191 | NOX4, EGFR, FLT3, ERBB2, EZH2, PDGFRB, HTR2B, SRC |
|  |  | positive regulation of phosphatidylinositol 3-kinase signaling | 0.001139306 | PTPN6, PTK2, FGR, FLT3, F2, TEK, PDGFRB, KDR |
|  |  | positive regulation of cell migration | 0.002803598 | EGFR, IGF1R, DRD1, PTK2, LYN, FGR, PDGFRB, INSR, PIK3R1, KDR, ALOX12 |
|  |  | cellular response to jasmonic acid stimulus | 0.003025003 | AKR1C3, AKR1C2, AKR1C4, AKR1C1 |
|  |  | synaptic transmission, dopaminergic | 0.003896724 | DRD1, ADORA2A, DRD2, DRD4, CDK5 |
|  |  | platelet activation | 0.005151145 | AKT1, PTPN6, LYN, F2, AXL, PIK3R1, SRC, SYK, PTPN11 |
|  |  | response to toxic substance | 0.00710549 | CDK1, CYP1B1, CES1, LYN, DRD2, PDGFRB, CDK4, AHR |
|  |  | ephrin receptor signaling pathway | 0.007685685 | CDK5R1, PTK2, LYN, MMP9, MMP2, EPHB4, SRC, PTPN11 |
|  |  | peptidyl-threonine phosphorylation | 0.017655867 | AKT1, CDK1, CDK5R1, GSK3B, TNKS, CDK5 |
|  |  | G2/M transition of mitotic cell cycle | 0.018882692 | CCNB1, CDK1, PLK4, CCNB2, PLK1, NEK2, AURKA, ABCB1, CDK2 |
|  |  | cell division | 0.029708665 | CDK1, NEK2, AURKA, CDK4, CDK5, CDK2, CCNB1, CCNB3, CCND1, CCNB2, TNKS, CCNA1, CCNA2 |
|  |  | positive regulation of protein localization to nucleus | 0.031083649 | AKT1, CDK1, PLK1, F2, SRC |
|  |  | mitotic nuclear division | 0.039014336 | CDK1, CCNB2, PLK1, NEK2, TNKS, AURKA, AURKB, CCNA1, CCNA2, NEK6, CDK2 |
|  |  | signal transduction | 0.044663452 | EGFR, LYN, ERBB2, MET, AXL, PKN1, ALK, CDK4, ADORA1, SRC, STAT3, DAPK1, AKT1, IGF1R, CD38, CSNK2A1, PTGES, TEK, GRK6, PLA2G1B, PDGFRB, CAMK2B, PIK3R1, NEK6 |
| cellular component | I | cytosol | 3.79E-04 | XDH, NEK2, CA13, AURKB, SRC, AKT1, AKR1C3, PTK2, AKR1C4, CSNK2A1, AKR1A1, GLO1, CAMK2B, PIK3R1, AKR1C1, CDK1, FLT3, PKN1, ALOX15, PYGL, PLK1, CA7, GSK3B, AKR1B1, CA6, CA3, ALOX5, CA2, CA1, NEK6, ALOX12 |
|  |  | plasma membrane | 0.001194755 | AVPR2, CA14, ADORA2A, DRD4, CXCR1, MMP2, ADORA1, SRC, AKT1, IGF1R, PTK2, CSNK2A1, CAMK2B, PIK3R1, EGFR, FLT3, CA12, MET, PIM1, AXL, PKN1, ABCB1, KDR, ABCG2, DAPK1, ALOX15, CA9, PYGL, GSK3B, BACE1, F2, CA4, ABCC1, CA2 |
|  | II | cytosol | 0.003095701 | XDH, PTPN6, CDK1, CDK5R1, CES1, FLT3, CDK5, STAT3, PTPN11, TNKS2, CCNB1, ARG1, APP, CCNB2, GSK3B, CA7, AKR1B10, AKR1B1, TNKS, GLO1, ALOX5, CA2, SYK |
|  | III | cytosol | 1.89E-07 | XDH, CDK5R1, CA13, HSD17B1, NEK2, AURKB, SRC, AKR1C3, AKT1, ARG1, PTK2, TYR, APP, CSNK2A1, AKR1C4, AKR1A1, CAMK2B, GLO1, TNKS, AKR1C1, PIK3R1, SYK, CDK1, PTPN6, CES1, FLT3, PKN1, CDK5, STAT3, TNKS2, PTPN11, CCNB1, ALOX15, CCNB2, PYGL, PLK1, AKR1B10, CA7, GSK3B, AKR1B1, CA6, CA3, ALOX5, CA2, CA1, NEK6, ALOX12 |
|  |  | plasma membrane | 0.01456746 | AVPR2, CDK5R1, ACHE, CA14, ADORA2A, DRD4, CXCR1, MMP2, ADORA1, SRC, AKT1, IGF1R, PTK2, APP, CSNK2A1, CAMK2B, TERT, PIK3R1, SYK, EGFR, FLT3, CA12, MET, PIM1, AXL, PKN1, ABCB1, CDK5, STAT3, KDR, DAPK1, ABCG2, CD38, ALOX15, CA9, PYGL, GSK3B, BACE1, F2, GRK6, CA4, ABCC1, CA2 |
|  |  | extracellular space | 0.046020825 | XDH, EGFR, CES2, ACHE, CES1, MMP9, AXL, MMP3, MMP2, MMP13, TTR, ARG1, APP, AKR1A1, CA6, F2, AKR1B1, PLA2G1B, MPO, ALOX5, CA2 |
|  | IV | cytosol | 1.57918E-09 | AURKA, AURKB, AKR1C3, AKT1, TYR, APP, AKR1C4, CSNK2A1, MAP3K8, CCNA1, AKR1C1, SYK, CDK1, LYN, BRAF, PKN1, CDK4, CDK5, CDK2, TNKS2, ALOX15, CCND1, AKR1B10, CA7, CA6, CA3, CA2, CA1, NEK6, ALOX12, XDH, CDK5R1, FGR, CA13, NEK2, HSD17B1, HSPA1A, EPHB4, SRC, ARG1, PTK2, CBR1, AKR1A1, CAMK2B, GLO1, TNKS, PIK3R1, PTPN6, CES1, FLT3, STAT3, PTPN11, CCNB1, PLK4, CCNB2, PLK1, PYGL, GSK3B, AKR1B1, ALOX5 |
|  |  | plasma membrane | 3.80812E-05 | CXCR1, MMP2, ADORA1, AKT1, APP, HTR1A, CSNK2A1, INSR, HTR5A, TERT, SYK, EGFR, LYN, BRAF, CA12, PIM1, PKN1, CDK5, DAPK1, CD38, ALOX15, CA9, HTR7, F2, HTR6, BACE1, PDGFRB, CA4, CA2, AVPR2, CDK5R1, DRD1, ACHE, CA14, FGR, DRD2, ADORA2A, ERBB2, DRD4, EPHB4, SRC, IGF1R, PTK2, TEK, CAMK2B, PIK3R1, FLT3, FLT4, MET, AXL, ABCB1, STAT3, KDR, ABCG2, PYGL, GSK3B, GRK6, ABCC1, HTR2B |
|  |  | perinuclear region of cytoplasm | 0.012922767 | NOX4, EGFR, CDK5R1, ACHE, LYN, ERBB2, AURKA, HSPA1A, CDK4, SRC, TNKS2, APP, TYR, PTGES, GSK3B, AKR1B1, CA4 |
| pathway | I | Nitrogen metabolism | 2.17E-14 | CA14, CA9, CA13, CA12, CA7, CA5A, CA6, CA4, CA3, CA2, CA1 |
|  |  | Proteoglycans in cancer | 0.007589296 | AKT1, EGFR, IGF1R, PTK2, MMP9, MET, CAMK2B, MMP2, PIK3R1, SRC, KDR |
|  |  | Steroid hormone biosynthesis | 0.01067867 | AKR1C3, AKR1C2, AKR1C4, CYP1B1, HSD17B2, AKR1C1, CYP19A1 |
|  | II | NA | NA |  |
|  | III | Nitrogen metabolism | 8.10E-13 | CA14, CA9, CA13, CA12, CA7, CA5A, CA6, CA4, CA3, CA2, CA1 |
|  |  | Proteoglycans in cancer | 7.77E-04 | EGFR, PTPN6, MMP9, MET, MMP2, STAT3, SRC, KDR, PTPN11, AKT1, IGF1R, PTK2, CAMK2B, PIK3R1 |
|  |  | Steroid hormone biosynthesis | 0.006460391 | AKR1C3, AKR1C2, AKR1C4, CYP1B1, HSD17B2, HSD17B1, AKR1C1, CYP19A1 |
|  |  | Ovarian steroidogenesis | 0.029088485 | AKR1C3, IGF1R, CYP1B1, HSD17B2, HSD17B1, ALOX5, CYP19A1 |
|  | IV | Nitrogen metabolism | 2.08278E-11 | CA14, CA9, CA13, CA12, CA7, CA5A, CA6, CA4, CA3, CA2, CA1 |
|  |  | Proteoglycans in cancer | 0.000163506 | EGFR, PTPN6, BRAF, ERBB2, MMP9, MET, MMP2, STAT3, SRC, KDR, PTPN11, AKT1, IGF1R, PTK2, CCND1, CAMK2B, PIK3R1 |
|  |  | Progesterone-mediated oocyte maturation | 0.000174537 | CCNB1, AKT1, CDK1, IGF1R, CCNB3, CCNB2, BRAF, PLK1, CCNA1, CCNA2, PIK3R1, CDK2 |
|  |  | FoxO signaling pathway | 0.00029232 | EGFR, BRAF, CDK2, STAT3, AKT1, CCNB1, IGF1R, CCNB3, PLK4, CCND1, CCNB2, PLK1, INSR, PIK3R1 |
|  |  | Bladder cancer | 0.000310311 | EGFR, CCND1, BRAF, MMP9, ERBB2, CDK4, MMP2, SRC, DAPK1 |
|  |  | Glioma | 0.011814017 | AKT1, EGFR, IGF1R, CCND1, BRAF, PDGFRB, CAMK2B, CDK4, PIK3R1 |
|  |  | Prostate cancer | 0.015070849 | AKT1, EGFR, IGF1R, CCND1, BRAF, GSK3B, ERBB2, PDGFRB, PIK3R1, CDK2 |
|  |  | Serotonergic synapse | 0.015187993 | APP, ALOX15, HTR1A, BRAF, HTR7, MAOA, HTR6, ALOX5, HTR2B, HTR5A, ALOX12 |
|  |  | Ovarian steroidogenesis | 0.016062509 | AKR1C3, IGF1R, CYP1B1, HSD17B2, HSD17B1, ALOX5, INSR, CYP19A1 |
|  |  | Melanoma | 0.022945418 | AKT1, EGFR, IGF1R, CCND1, BRAF, MET, PDGFRB, CDK4, PIK3R1 |
|  |  | Focal adhesion | 0.036100559 | EGFR, AKT1, IGF1R, PTK2, CCND1, BRAF, FLT4, GSK3B, ERBB2, MET, PDGFRB, PIK3R1, SRC, KDR |
|  |  | Non-small cell lung cancer | 0.039444396 | AKT1, EGFR, CCND1, BRAF, ERBB2, CDK4, ALK, PIK3R1 |
|  |  | Cell cycle | 0.040220713 | CCNB1, CDK1, CCNB3, CCND1, CCNB2, PLK1, GSK3B, CCNA1, CDK4, CCNA2, CDK2 |
|  |  | Rap1 signaling pathway | 0.044203009 | EGFR, AKT1, IGF1R, BRAF, ADORA2A, DRD2, FLT4, TEK, MET, PDGFRB, INSR, PIK3R1, SRC, KDR |
|  |  | Steroid hormone biosynthesis | 0.049772054 | AKR1C3, AKR1C2, AKR1C4, CYP1B1, HSD17B2, HSD17B1, AKR1C1, CYP19A1 |

**Table S8.** Score of potential protein-protein interactions

| **Startegy** | **Node1** | **Node2** | **Node1 accession** | **Node2 accession** | **Score** |
| --- | --- | --- | --- | --- | --- |
| I | ADORA1 | CXCR1 | ENSP00000356205 | ENSP00000295683 | 0.9 |
|  | ADORA1 | DRD4 | ENSP00000356205 | ENSP00000176183 | 0.9 |
|  | AKR1B1 | AKR1C3 | ENSP00000285930 | ENSP00000369927 | 0.9 |
|  | AKR1C1 | AKR1C2 | ENSP00000370254 | ENSP00000370129 | 0.985 |
|  | AKR1C1 | AKR1C3 | ENSP00000370254 | ENSP00000369927 | 0.994 |
|  | AKR1C2 | AKR1C1 | ENSP00000370129 | ENSP00000370254 | 0.985 |
|  | AKR1C2 | AKR1C3 | ENSP00000370129 | ENSP00000369927 | 0.981 |
|  | AKR1C3 | AKR1B1 | ENSP00000369927 | ENSP00000285930 | 0.9 |
|  | AKR1C3 | AKR1C1 | ENSP00000369927 | ENSP00000370254 | 0.994 |
|  | AKR1C3 | AKR1C2 | ENSP00000369927 | ENSP00000370129 | 0.981 |
|  | AKR1C3 | AKR1C4 | ENSP00000369927 | ENSP00000369814 | 0.933 |
|  | AKR1C3 | CYP19A1 | ENSP00000369927 | ENSP00000379683 | 0.9 |
|  | AKR1C3 | CYP1B1 | ENSP00000369927 | ENSP00000478561 | 0.9 |
|  | AKR1C4 | AKR1C3 | ENSP00000369814 | ENSP00000369927 | 0.933 |
|  | AKT1 | GSK3B | ENSP00000451828 | ENSP00000324806 | 0.999 |
|  | AKT1 | PIK3R1 | ENSP00000451828 | ENSP00000428056 | 0.942 |
|  | AKT1 | SRC | ENSP00000451828 | ENSP00000362680 | 0.952 |
|  | ALOX12 | ALOX15 | ENSP00000251535 | ENSP00000458832 | 0.9 |
|  | ALOX12 | ALOX5 | ENSP00000251535 | ENSP00000363512 | 0.9 |
|  | ALOX15 | ALOX12 | ENSP00000458832 | ENSP00000251535 | 0.9 |
|  | ALOX15 | ALOX5 | ENSP00000458832 | ENSP00000363512 | 0.9 |
|  | ALOX5 | ALOX12 | ENSP00000363512 | ENSP00000251535 | 0.9 |
|  | ALOX5 | ALOX15 | ENSP00000363512 | ENSP00000458832 | 0.9 |
|  | AURKB | CDK1 | ENSP00000313950 | ENSP00000378699 | 0.937 |
|  | AURKB | PLK1 | ENSP00000313950 | ENSP00000300093 | 0.933 |
|  | AVPR2 | EGFR | ENSP00000351805 | ENSP00000275493 | 0.904 |
|  | AXL | KDR | ENSP00000301178 | ENSP00000263923 | 0.905 |
|  | AXL | PIK3R1 | ENSP00000301178 | ENSP00000428056 | 0.937 |
|  | CAMK2B | SRC | ENSP00000379098 | ENSP00000362680 | 0.908 |
|  | CDK1 | AURKB | ENSP00000378699 | ENSP00000313950 | 0.937 |
|  | CDK1 | NEK2 | ENSP00000378699 | ENSP00000355966 | 0.9 |
|  | CDK1 | PKN1 | ENSP00000378699 | ENSP00000343325 | 0.903 |
|  | CDK1 | PLK1 | ENSP00000378699 | ENSP00000300093 | 0.923 |
|  | CDK1 | SRC | ENSP00000378699 | ENSP00000362680 | 0.905 |
|  | CXCR1 | ADORA1 | ENSP00000295683 | ENSP00000356205 | 0.9 |
|  | CXCR1 | DRD4 | ENSP00000295683 | ENSP00000176183 | 0.9 |
|  | CYP19A1 | AKR1C3 | ENSP00000379683 | ENSP00000369927 | 0.9 |
|  | CYP19A1 | HSD17B2 | ENSP00000379683 | ENSP00000199936 | 0.904 |
|  | CYP1B1 | AKR1C3 | ENSP00000478561 | ENSP00000369927 | 0.9 |
|  | CYP1B1 | HSD17B2 | ENSP00000478561 | ENSP00000199936 | 0.902 |
|  | DRD4 | ADORA1 | ENSP00000176183 | ENSP00000356205 | 0.9 |
|  | DRD4 | CXCR1 | ENSP00000176183 | ENSP00000295683 | 0.9 |
|  | EGFR | AVPR2 | ENSP00000275493 | ENSP00000351805 | 0.904 |
|  | EGFR | MMP3 | ENSP00000275493 | ENSP00000299855 | 0.9 |
|  | EGFR | PIK3R1 | ENSP00000275493 | ENSP00000428056 | 0.987 |
|  | EGFR | SRC | ENSP00000275493 | ENSP00000362680 | 0.946 |
|  | F2 | PIK3R1 | ENSP00000308541 | ENSP00000428056 | 0.9 |
|  | F2 | SRC | ENSP00000308541 | ENSP00000362680 | 0.9 |
|  | FLT3 | PIK3R1 | ENSP00000241453 | ENSP00000428056 | 0.935 |
|  | GSK3B | AKT1 | ENSP00000324806 | ENSP00000451828 | 0.999 |
|  | HSD17B2 | CYP19A1 | ENSP00000199936 | ENSP00000379683 | 0.904 |
|  | HSD17B2 | CYP1B1 | ENSP00000199936 | ENSP00000478561 | 0.902 |
|  | IGF1R | PIK3R1 | ENSP00000268035 | ENSP00000428056 | 0.956 |
|  | IGF1R | SRC | ENSP00000268035 | ENSP00000362680 | 0.938 |
|  | KDR | AXL | ENSP00000263923 | ENSP00000301178 | 0.905 |
|  | KDR | PIK3R1 | ENSP00000263923 | ENSP00000428056 | 0.912 |
|  | KDR | PTK2 | ENSP00000263923 | ENSP00000341189 | 0.905 |
|  | KDR | SRC | ENSP00000263923 | ENSP00000362680 | 0.943 |
|  | MET | PIK3R1 | ENSP00000317272 | ENSP00000428056 | 0.938 |
|  | MET | PTK2 | ENSP00000317272 | ENSP00000341189 | 0.908 |
|  | MET | SRC | ENSP00000317272 | ENSP00000362680 | 0.938 |
|  | MMP13 | MMP2 | ENSP00000260302 | ENSP00000219070 | 0.9 |
|  | MMP13 | MMP3 | ENSP00000260302 | ENSP00000299855 | 0.9 |
|  | MMP13 | MMP9 | ENSP00000260302 | ENSP00000361405 | 0.9 |
|  | MMP2 | MMP13 | ENSP00000219070 | ENSP00000260302 | 0.9 |
|  | MMP2 | MMP3 | ENSP00000219070 | ENSP00000299855 | 0.9 |
|  | MMP2 | MMP9 | ENSP00000219070 | ENSP00000361405 | 0.9 |
|  | MMP2 | SRC | ENSP00000219070 | ENSP00000362680 | 0.9 |
|  | MMP3 | EGFR | ENSP00000299855 | ENSP00000275493 | 0.9 |
|  | MMP3 | MMP13 | ENSP00000299855 | ENSP00000260302 | 0.9 |
|  | MMP3 | MMP2 | ENSP00000299855 | ENSP00000219070 | 0.9 |
|  | MMP3 | MMP9 | ENSP00000299855 | ENSP00000361405 | 0.9 |
|  | MMP9 | MMP13 | ENSP00000361405 | ENSP00000260302 | 0.9 |
|  | MMP9 | MMP2 | ENSP00000361405 | ENSP00000219070 | 0.9 |
|  | MMP9 | MMP3 | ENSP00000361405 | ENSP00000299855 | 0.9 |
|  | MMP9 | SRC | ENSP00000361405 | ENSP00000362680 | 0.9 |
|  | NEK2 | CDK1 | ENSP00000355966 | ENSP00000378699 | 0.9 |
|  | NEK2 | PLK1 | ENSP00000355966 | ENSP00000300093 | 0.9 |
|  | PIK3R1 | AKT1 | ENSP00000428056 | ENSP00000451828 | 0.942 |
|  | PIK3R1 | AXL | ENSP00000428056 | ENSP00000301178 | 0.937 |
|  | PIK3R1 | EGFR | ENSP00000428056 | ENSP00000275493 | 0.987 |
|  | PIK3R1 | F2 | ENSP00000428056 | ENSP00000308541 | 0.9 |
|  | PIK3R1 | FLT3 | ENSP00000428056 | ENSP00000241453 | 0.935 |
|  | PIK3R1 | IGF1R | ENSP00000428056 | ENSP00000268035 | 0.956 |
|  | PIK3R1 | KDR | ENSP00000428056 | ENSP00000263923 | 0.912 |
|  | PIK3R1 | MET | ENSP00000428056 | ENSP00000317272 | 0.938 |
|  | PIK3R1 | PTK2 | ENSP00000428056 | ENSP00000341189 | 0.935 |
|  | PIK3R1 | SRC | ENSP00000428056 | ENSP00000362680 | 0.968 |
|  | PKN1 | CDK1 | ENSP00000343325 | ENSP00000378699 | 0.903 |
|  | PLK1 | AURKB | ENSP00000300093 | ENSP00000313950 | 0.933 |
|  | PLK1 | CDK1 | ENSP00000300093 | ENSP00000378699 | 0.923 |
|  | PLK1 | NEK2 | ENSP00000300093 | ENSP00000355966 | 0.9 |
|  | PTK2 | KDR | ENSP00000341189 | ENSP00000263923 | 0.905 |
|  | PTK2 | MET | ENSP00000341189 | ENSP00000317272 | 0.908 |
|  | PTK2 | PIK3R1 | ENSP00000341189 | ENSP00000428056 | 0.935 |
|  | PTK2 | SRC | ENSP00000341189 | ENSP00000362680 | 0.961 |
|  | SRC | AKT1 | ENSP00000362680 | ENSP00000451828 | 0.952 |
|  | SRC | CAMK2B | ENSP00000362680 | ENSP00000379098 | 0.908 |
|  | SRC | CDK1 | ENSP00000362680 | ENSP00000378699 | 0.905 |
|  | SRC | EGFR | ENSP00000362680 | ENSP00000275493 | 0.946 |
|  | SRC | F2 | ENSP00000362680 | ENSP00000308541 | 0.9 |
|  | SRC | IGF1R | ENSP00000362680 | ENSP00000268035 | 0.938 |
|  | SRC | KDR | ENSP00000362680 | ENSP00000263923 | 0.943 |
|  | SRC | MET | ENSP00000362680 | ENSP00000317272 | 0.938 |
|  | SRC | MMP2 | ENSP00000362680 | ENSP00000219070 | 0.9 |
|  | SRC | MMP9 | ENSP00000362680 | ENSP00000361405 | 0.9 |
|  | SRC | PIK3R1 | ENSP00000362680 | ENSP00000428056 | 0.968 |
|  | SRC | PTK2 | ENSP00000362680 | ENSP00000341189 | 0.961 |
| II | ADORA1 | APP | ENSP00000356205 | ENSP00000284981 | 0.9 |
|  | AKR1B1 | AKR1B10 | ENSP00000285930 | ENSP00000352584 | 0.9 |
|  | AKR1B10 | AKR1B1 | ENSP00000352584 | ENSP00000285930 | 0.9 |
|  | APP | ADORA1 | ENSP00000284981 | ENSP00000356205 | 0.9 |
|  | APP | TTR | ENSP00000284981 | ENSP00000237014 | 0.935 |
|  | ARG1 | PTPN6 | ENSP00000349446 | ENSP00000391592 | 0.9 |
|  | ARG1 | TTR | ENSP00000349446 | ENSP00000237014 | 0.9 |
|  | CCNB1 | CCNB2 | ENSP00000256442 | ENSP00000288207 | 0.994 |
|  | CCNB1 | CCNB3 | ENSP00000256442 | ENSP00000365210 | 0.944 |
|  | CCNB1 | CDK1 | ENSP00000256442 | ENSP00000378699 | 0.999 |
|  | CCNB1 | CDK5 | ENSP00000256442 | ENSP00000419782 | 0.923 |
|  | CCNB2 | CCNB1 | ENSP00000288207 | ENSP00000256442 | 0.994 |
|  | CCNB2 | CCNB3 | ENSP00000288207 | ENSP00000365210 | 0.944 |
|  | CCNB2 | CDK1 | ENSP00000288207 | ENSP00000378699 | 0.996 |
|  | CCNB2 | CDK5 | ENSP00000288207 | ENSP00000419782 | 0.902 |
|  | CCNB3 | CCNB1 | ENSP00000365210 | ENSP00000256442 | 0.944 |
|  | CCNB3 | CCNB2 | ENSP00000365210 | ENSP00000288207 | 0.944 |
|  | CCNB3 | CDK1 | ENSP00000365210 | ENSP00000378699 | 0.957 |
|  | CDK1 | CCNB1 | ENSP00000378699 | ENSP00000256442 | 0.999 |
|  | CDK1 | CCNB2 | ENSP00000378699 | ENSP00000288207 | 0.996 |
|  | CDK1 | CCNB3 | ENSP00000378699 | ENSP00000365210 | 0.957 |
|  | CDK1 | CDK5 | ENSP00000378699 | ENSP00000419782 | 0.902 |
|  | CDK5 | CCNB1 | ENSP00000419782 | ENSP00000256442 | 0.923 |
|  | CDK5 | CCNB2 | ENSP00000419782 | ENSP00000288207 | 0.902 |
|  | CDK5 | CDK1 | ENSP00000419782 | ENSP00000378699 | 0.902 |
|  | CDK5 | CDK5R1 | ENSP00000419782 | ENSP00000318486 | 0.997 |
|  | CDK5 | GSK3B | ENSP00000419782 | ENSP00000324806 | 0.937 |
|  | CDK5R1 | CDK5 | ENSP00000318486 | ENSP00000419782 | 0.997 |
|  | CDK5R1 | GSK3B | ENSP00000318486 | ENSP00000324806 | 0.904 |
|  | EED | EZH2 | ENSP00000263360 | ENSP00000320147 | 0.998 |
|  | EED | SUZ12 | ENSP00000263360 | ENSP00000316578 | 0.999 |
|  | EZH2 | EED | ENSP00000320147 | ENSP00000263360 | 0.998 |
|  | EZH2 | SUZ12 | ENSP00000320147 | ENSP00000316578 | 0.998 |
|  | GSK3B | CDK5 | ENSP00000324806 | ENSP00000419782 | 0.937 |
|  | GSK3B | CDK5R1 | ENSP00000324806 | ENSP00000318486 | 0.904 |
|  | MMP2 | MMP9 | ENSP00000219070 | ENSP00000361405 | 0.9 |
|  | MMP2 | STAT3 | ENSP00000219070 | ENSP00000264657 | 0.9 |
|  | MMP9 | MMP2 | ENSP00000361405 | ENSP00000219070 | 0.9 |
|  | MMP9 | PTPN6 | ENSP00000361405 | ENSP00000391592 | 0.9 |
|  | MMP9 | STAT3 | ENSP00000361405 | ENSP00000264657 | 0.9 |
|  | PARP1 | POLB | ENSP00000355759 | ENSP00000265421 | 0.973 |
|  | POLB | PARP1 | ENSP00000265421 | ENSP00000355759 | 0.973 |
|  | PTPN11 | PTPN6 | ENSP00000340944 | ENSP00000391592 | 0.935 |
|  | PTPN11 | STAT3 | ENSP00000340944 | ENSP00000264657 | 0.941 |
|  | PTPN11 | SYK | ENSP00000340944 | ENSP00000364907 | 0.912 |
|  | PTPN6 | ARG1 | ENSP00000391592 | ENSP00000349446 | 0.9 |
|  | PTPN6 | MMP9 | ENSP00000391592 | ENSP00000361405 | 0.9 |
|  | PTPN6 | PTPN11 | ENSP00000391592 | ENSP00000340944 | 0.935 |
|  | PTPN6 | SYK | ENSP00000391592 | ENSP00000364907 | 0.943 |
|  | STAT3 | MMP2 | ENSP00000264657 | ENSP00000219070 | 0.9 |
|  | STAT3 | MMP9 | ENSP00000264657 | ENSP00000361405 | 0.9 |
|  | STAT3 | PTPN11 | ENSP00000264657 | ENSP00000340944 | 0.941 |
|  | SUZ12 | EED | ENSP00000316578 | ENSP00000263360 | 0.999 |
|  | SUZ12 | EZH2 | ENSP00000316578 | ENSP00000320147 | 0.998 |
|  | SYK | PTPN11 | ENSP00000364907 | ENSP00000340944 | 0.912 |
|  | SYK | PTPN6 | ENSP00000364907 | ENSP00000391592 | 0.943 |
|  | TERT | TNKS | ENSP00000309572 | ENSP00000311579 | 0.9 |
|  | TNKS | TERT | ENSP00000311579 | ENSP00000309572 | 0.9 |
|  | TNKS | TNKS2 | ENSP00000311579 | ENSP00000360689 | 0.927 |
|  | TNKS2 | TNKS | ENSP00000360689 | ENSP00000311579 | 0.927 |
|  | TTR | APP | ENSP00000237014 | ENSP00000284981 | 0.935 |
|  | TTR | ARG1 | ENSP00000237014 | ENSP00000349446 | 0.9 |
| III | ADORA1 | APP | ENSP00000356205 | ENSP00000284981 | 0.9 |
|  | ADORA1 | CXCR1 | ENSP00000356205 | ENSP00000295683 | 0.9 |
|  | ADORA1 | DRD4 | ENSP00000356205 | ENSP00000176183 | 0.9 |
|  | AKR1B1 | AKR1B10 | ENSP00000285930 | ENSP00000352584 | 0.9 |
|  | AKR1B1 | AKR1C3 | ENSP00000285930 | ENSP00000369927 | 0.9 |
|  | AKR1B10 | AKR1B1 | ENSP00000352584 | ENSP00000285930 | 0.9 |
|  | AKR1B10 | AKR1C3 | ENSP00000352584 | ENSP00000369927 | 0.9 |
|  | AKR1C1 | AKR1C2 | ENSP00000370254 | ENSP00000370129 | 0.985 |
|  | AKR1C1 | AKR1C3 | ENSP00000370254 | ENSP00000369927 | 0.994 |
|  | AKR1C2 | AKR1C1 | ENSP00000370129 | ENSP00000370254 | 0.985 |
|  | AKR1C2 | AKR1C3 | ENSP00000370129 | ENSP00000369927 | 0.981 |
|  | AKR1C3 | AKR1B1 | ENSP00000369927 | ENSP00000285930 | 0.9 |
|  | AKR1C3 | AKR1B10 | ENSP00000369927 | ENSP00000352584 | 0.9 |
|  | AKR1C3 | AKR1C1 | ENSP00000369927 | ENSP00000370254 | 0.994 |
|  | AKR1C3 | AKR1C2 | ENSP00000369927 | ENSP00000370129 | 0.981 |
|  | AKR1C3 | AKR1C4 | ENSP00000369927 | ENSP00000369814 | 0.933 |
|  | AKR1C3 | CYP19A1 | ENSP00000369927 | ENSP00000379683 | 0.9 |
|  | AKR1C3 | CYP1B1 | ENSP00000369927 | ENSP00000478561 | 0.9 |
|  | AKR1C4 | AKR1C3 | ENSP00000369814 | ENSP00000369927 | 0.933 |
|  | AKT1 | GSK3B | ENSP00000451828 | ENSP00000324806 | 0.999 |
|  | AKT1 | PIK3R1 | ENSP00000451828 | ENSP00000428056 | 0.942 |
|  | AKT1 | PTPN11 | ENSP00000451828 | ENSP00000340944 | 0.912 |
|  | AKT1 | SRC | ENSP00000451828 | ENSP00000362680 | 0.952 |
|  | AKT1 | STAT3 | ENSP00000451828 | ENSP00000264657 | 0.908 |
|  | AKT1 | TERT | ENSP00000451828 | ENSP00000309572 | 0.935 |
|  | ALOX12 | ALOX15 | ENSP00000251535 | ENSP00000458832 | 0.9 |
|  | ALOX12 | ALOX5 | ENSP00000251535 | ENSP00000363512 | 0.9 |
|  | ALOX15 | ALOX12 | ENSP00000458832 | ENSP00000251535 | 0.9 |
|  | ALOX15 | ALOX5 | ENSP00000458832 | ENSP00000363512 | 0.9 |
|  | ALOX5 | ALOX12 | ENSP00000363512 | ENSP00000251535 | 0.9 |
|  | ALOX5 | ALOX15 | ENSP00000363512 | ENSP00000458832 | 0.9 |
|  | APP | ADORA1 | ENSP00000284981 | ENSP00000356205 | 0.9 |
|  | APP | BACE1 | ENSP00000284981 | ENSP00000318585 | 0.938 |
|  | APP | CXCR1 | ENSP00000284981 | ENSP00000295683 | 0.9 |
|  | APP | DRD4 | ENSP00000284981 | ENSP00000176183 | 0.9 |
|  | APP | F2 | ENSP00000284981 | ENSP00000308541 | 0.909 |
|  | APP | PIK3R1 | ENSP00000284981 | ENSP00000428056 | 0.9 |
|  | APP | TTR | ENSP00000284981 | ENSP00000237014 | 0.935 |
|  | ARG1 | MPO | ENSP00000349446 | ENSP00000225275 | 0.9 |
|  | ARG1 | PTPN6 | ENSP00000349446 | ENSP00000391592 | 0.9 |
|  | ARG1 | TTR | ENSP00000349446 | ENSP00000237014 | 0.9 |
|  | AURKB | CCNB1 | ENSP00000313950 | ENSP00000256442 | 0.926 |
|  | AURKB | CCNB2 | ENSP00000313950 | ENSP00000288207 | 0.926 |
|  | AURKB | CDK1 | ENSP00000313950 | ENSP00000378699 | 0.937 |
|  | AURKB | PLK1 | ENSP00000313950 | ENSP00000300093 | 0.933 |
|  | AVPR2 | EGFR | ENSP00000351805 | ENSP00000275493 | 0.904 |
|  | AXL | KDR | ENSP00000301178 | ENSP00000263923 | 0.905 |
|  | AXL | PIK3R1 | ENSP00000301178 | ENSP00000428056 | 0.937 |
|  | BACE1 | APP | ENSP00000318585 | ENSP00000284981 | 0.938 |
|  | CAMK2B | SRC | ENSP00000379098 | ENSP00000362680 | 0.908 |
|  | CCNB1 | AURKB | ENSP00000256442 | ENSP00000313950 | 0.926 |
|  | CCNB1 | CCNB2 | ENSP00000256442 | ENSP00000288207 | 0.994 |
|  | CCNB1 | CCNB3 | ENSP00000256442 | ENSP00000365210 | 0.944 |
|  | CCNB1 | CDK1 | ENSP00000256442 | ENSP00000378699 | 0.999 |
|  | CCNB1 | CDK5 | ENSP00000256442 | ENSP00000419782 | 0.923 |
|  | CCNB1 | PLK1 | ENSP00000256442 | ENSP00000300093 | 0.976 |
|  | CCNB2 | AURKB | ENSP00000288207 | ENSP00000313950 | 0.926 |
|  | CCNB2 | CCNB1 | ENSP00000288207 | ENSP00000256442 | 0.994 |
|  | CCNB2 | CCNB3 | ENSP00000288207 | ENSP00000365210 | 0.944 |
|  | CCNB2 | CDK1 | ENSP00000288207 | ENSP00000378699 | 0.996 |
|  | CCNB2 | CDK5 | ENSP00000288207 | ENSP00000419782 | 0.902 |
|  | CCNB2 | PLK1 | ENSP00000288207 | ENSP00000300093 | 0.927 |
|  | CCNB3 | CCNB1 | ENSP00000365210 | ENSP00000256442 | 0.944 |
|  | CCNB3 | CCNB2 | ENSP00000365210 | ENSP00000288207 | 0.944 |
|  | CCNB3 | CDK1 | ENSP00000365210 | ENSP00000378699 | 0.957 |
|  | CDK1 | AURKB | ENSP00000378699 | ENSP00000313950 | 0.937 |
|  | CDK1 | CCNB1 | ENSP00000378699 | ENSP00000256442 | 0.999 |
|  | CDK1 | CCNB2 | ENSP00000378699 | ENSP00000288207 | 0.996 |
|  | CDK1 | CCNB3 | ENSP00000378699 | ENSP00000365210 | 0.957 |
|  | CDK1 | CDK5 | ENSP00000378699 | ENSP00000419782 | 0.902 |
|  | CDK1 | NEK2 | ENSP00000378699 | ENSP00000355966 | 0.9 |
|  | CDK1 | PKN1 | ENSP00000378699 | ENSP00000343325 | 0.903 |
|  | CDK1 | PLK1 | ENSP00000378699 | ENSP00000300093 | 0.923 |
|  | CDK1 | SRC | ENSP00000378699 | ENSP00000362680 | 0.905 |
|  | CDK5 | CCNB1 | ENSP00000419782 | ENSP00000256442 | 0.923 |
|  | CDK5 | CCNB2 | ENSP00000419782 | ENSP00000288207 | 0.902 |
|  | CDK5 | CDK1 | ENSP00000419782 | ENSP00000378699 | 0.902 |
|  | CDK5 | CDK5R1 | ENSP00000419782 | ENSP00000318486 | 0.997 |
|  | CDK5 | GSK3B | ENSP00000419782 | ENSP00000324806 | 0.937 |
|  | CDK5R1 | CDK5 | ENSP00000318486 | ENSP00000419782 | 0.997 |
|  | CDK5R1 | GSK3B | ENSP00000318486 | ENSP00000324806 | 0.904 |
|  | CES1 | MPO | ENSP00000353720 | ENSP00000225275 | 0.903 |
|  | CXCR1 | ADORA1 | ENSP00000295683 | ENSP00000356205 | 0.9 |
|  | CXCR1 | APP | ENSP00000295683 | ENSP00000284981 | 0.9 |
|  | CXCR1 | DRD4 | ENSP00000295683 | ENSP00000176183 | 0.9 |
|  | CYP19A1 | AKR1C3 | ENSP00000379683 | ENSP00000369927 | 0.9 |
|  | CYP19A1 | HSD17B1 | ENSP00000379683 | ENSP00000466799 | 0.901 |
|  | CYP19A1 | HSD17B2 | ENSP00000379683 | ENSP00000199936 | 0.904 |
|  | CYP1B1 | AKR1C3 | ENSP00000478561 | ENSP00000369927 | 0.9 |
|  | CYP1B1 | HSD17B1 | ENSP00000478561 | ENSP00000466799 | 0.905 |
|  | CYP1B1 | HSD17B2 | ENSP00000478561 | ENSP00000199936 | 0.902 |
|  | DRD4 | ADORA1 | ENSP00000176183 | ENSP00000356205 | 0.9 |
|  | DRD4 | APP | ENSP00000176183 | ENSP00000284981 | 0.9 |
|  | DRD4 | CXCR1 | ENSP00000176183 | ENSP00000295683 | 0.9 |
|  | EED | EZH2 | ENSP00000263360 | ENSP00000320147 | 0.998 |
|  | EED | SUZ12 | ENSP00000263360 | ENSP00000316578 | 0.999 |
|  | EGFR | AVPR2 | ENSP00000275493 | ENSP00000351805 | 0.904 |
|  | EGFR | MMP3 | ENSP00000275493 | ENSP00000299855 | 0.9 |
|  | EGFR | PIK3R1 | ENSP00000275493 | ENSP00000428056 | 0.987 |
|  | EGFR | PTPN11 | ENSP00000275493 | ENSP00000340944 | 0.979 |
|  | EGFR | SRC | ENSP00000275493 | ENSP00000362680 | 0.946 |
|  | EGFR | STAT3 | ENSP00000275493 | ENSP00000264657 | 0.974 |
|  | EZH2 | EED | ENSP00000320147 | ENSP00000263360 | 0.998 |
|  | EZH2 | SUZ12 | ENSP00000320147 | ENSP00000316578 | 0.998 |
|  | F2 | APP | ENSP00000308541 | ENSP00000284981 | 0.909 |
|  | F2 | PIK3R1 | ENSP00000308541 | ENSP00000428056 | 0.9 |
|  | F2 | SRC | ENSP00000308541 | ENSP00000362680 | 0.9 |
|  | FLT3 | PIK3R1 | ENSP00000241453 | ENSP00000428056 | 0.935 |
|  | GSK3B | AKT1 | ENSP00000324806 | ENSP00000451828 | 0.999 |
|  | GSK3B | CDK5 | ENSP00000324806 | ENSP00000419782 | 0.937 |
|  | GSK3B | CDK5R1 | ENSP00000324806 | ENSP00000318486 | 0.904 |
|  | HSD17B1 | CYP19A1 | ENSP00000466799 | ENSP00000379683 | 0.901 |
|  | HSD17B1 | CYP1B1 | ENSP00000466799 | ENSP00000478561 | 0.905 |
|  | HSD17B2 | CYP19A1 | ENSP00000199936 | ENSP00000379683 | 0.904 |
|  | HSD17B2 | CYP1B1 | ENSP00000199936 | ENSP00000478561 | 0.902 |
|  | IGF1R | PIK3R1 | ENSP00000268035 | ENSP00000428056 | 0.956 |
|  | IGF1R | PTPN11 | ENSP00000268035 | ENSP00000340944 | 0.943 |
|  | IGF1R | SRC | ENSP00000268035 | ENSP00000362680 | 0.938 |
|  | KDR | AXL | ENSP00000263923 | ENSP00000301178 | 0.905 |
|  | KDR | PIK3R1 | ENSP00000263923 | ENSP00000428056 | 0.912 |
|  | KDR | PTK2 | ENSP00000263923 | ENSP00000341189 | 0.905 |
|  | KDR | PTPN11 | ENSP00000263923 | ENSP00000340944 | 0.941 |
|  | KDR | PTPN6 | ENSP00000263923 | ENSP00000391592 | 0.941 |
|  | KDR | SRC | ENSP00000263923 | ENSP00000362680 | 0.943 |
|  | MET | PIK3R1 | ENSP00000317272 | ENSP00000428056 | 0.938 |
|  | MET | PTK2 | ENSP00000317272 | ENSP00000341189 | 0.908 |
|  | MET | PTPN11 | ENSP00000317272 | ENSP00000340944 | 0.944 |
|  | MET | SRC | ENSP00000317272 | ENSP00000362680 | 0.938 |
|  | MET | STAT3 | ENSP00000317272 | ENSP00000264657 | 0.94 |
|  | MMP13 | MMP2 | ENSP00000260302 | ENSP00000219070 | 0.9 |
|  | MMP13 | MMP3 | ENSP00000260302 | ENSP00000299855 | 0.9 |
|  | MMP13 | MMP9 | ENSP00000260302 | ENSP00000361405 | 0.9 |
|  | MMP2 | MMP13 | ENSP00000219070 | ENSP00000260302 | 0.9 |
|  | MMP2 | MMP3 | ENSP00000219070 | ENSP00000299855 | 0.9 |
|  | MMP2 | MMP9 | ENSP00000219070 | ENSP00000361405 | 0.9 |
|  | MMP2 | SRC | ENSP00000219070 | ENSP00000362680 | 0.9 |
|  | MMP2 | STAT3 | ENSP00000219070 | ENSP00000264657 | 0.9 |
|  | MMP3 | EGFR | ENSP00000299855 | ENSP00000275493 | 0.9 |
|  | MMP3 | MMP13 | ENSP00000299855 | ENSP00000260302 | 0.9 |
|  | MMP3 | MMP2 | ENSP00000299855 | ENSP00000219070 | 0.9 |
|  | MMP3 | MMP9 | ENSP00000299855 | ENSP00000361405 | 0.9 |
|  | MMP3 | STAT3 | ENSP00000299855 | ENSP00000264657 | 0.935 |
|  | MMP9 | MMP13 | ENSP00000361405 | ENSP00000260302 | 0.9 |
|  | MMP9 | MMP2 | ENSP00000361405 | ENSP00000219070 | 0.9 |
|  | MMP9 | MMP3 | ENSP00000361405 | ENSP00000299855 | 0.9 |
|  | MMP9 | PTPN6 | ENSP00000361405 | ENSP00000391592 | 0.9 |
|  | MMP9 | SRC | ENSP00000361405 | ENSP00000362680 | 0.9 |
|  | MMP9 | STAT3 | ENSP00000361405 | ENSP00000264657 | 0.9 |
|  | MPO | ARG1 | ENSP00000225275 | ENSP00000349446 | 0.9 |
|  | MPO | CES1 | ENSP00000225275 | ENSP00000353720 | 0.903 |
|  | MPO | TTR | ENSP00000225275 | ENSP00000237014 | 0.9 |
|  | NEK2 | CDK1 | ENSP00000355966 | ENSP00000378699 | 0.9 |
|  | NEK2 | PLK1 | ENSP00000355966 | ENSP00000300093 | 0.9 |
|  | PARP1 | POLB | ENSP00000355759 | ENSP00000265421 | 0.973 |
|  | PIK3R1 | AKT1 | ENSP00000428056 | ENSP00000451828 | 0.942 |
|  | PIK3R1 | APP | ENSP00000428056 | ENSP00000284981 | 0.9 |
|  | PIK3R1 | AXL | ENSP00000428056 | ENSP00000301178 | 0.937 |
|  | PIK3R1 | EGFR | ENSP00000428056 | ENSP00000275493 | 0.987 |
|  | PIK3R1 | F2 | ENSP00000428056 | ENSP00000308541 | 0.9 |
|  | PIK3R1 | FLT3 | ENSP00000428056 | ENSP00000241453 | 0.935 |
|  | PIK3R1 | IGF1R | ENSP00000428056 | ENSP00000268035 | 0.956 |
|  | PIK3R1 | KDR | ENSP00000428056 | ENSP00000263923 | 0.912 |
|  | PIK3R1 | MET | ENSP00000428056 | ENSP00000317272 | 0.938 |
|  | PIK3R1 | PTK2 | ENSP00000428056 | ENSP00000341189 | 0.935 |
|  | PIK3R1 | PTPN11 | ENSP00000428056 | ENSP00000340944 | 0.95 |
|  | PIK3R1 | PTPN6 | ENSP00000428056 | ENSP00000391592 | 0.945 |
|  | PIK3R1 | SRC | ENSP00000428056 | ENSP00000362680 | 0.968 |
|  | PIK3R1 | STAT3 | ENSP00000428056 | ENSP00000264657 | 0.949 |
|  | PIK3R1 | SYK | ENSP00000428056 | ENSP00000364907 | 0.938 |
|  | PIM1 | STAT3 | ENSP00000362608 | ENSP00000264657 | 0.927 |
|  | PKN1 | CDK1 | ENSP00000343325 | ENSP00000378699 | 0.903 |
|  | PLK1 | AURKB | ENSP00000300093 | ENSP00000313950 | 0.933 |
|  | PLK1 | CCNB1 | ENSP00000300093 | ENSP00000256442 | 0.976 |
|  | PLK1 | CCNB2 | ENSP00000300093 | ENSP00000288207 | 0.927 |
|  | PLK1 | CDK1 | ENSP00000300093 | ENSP00000378699 | 0.923 |
|  | PLK1 | NEK2 | ENSP00000300093 | ENSP00000355966 | 0.9 |
|  | POLB | PARP1 | ENSP00000265421 | ENSP00000355759 | 0.973 |
|  | PTK2 | KDR | ENSP00000341189 | ENSP00000263923 | 0.905 |
|  | PTK2 | MET | ENSP00000341189 | ENSP00000317272 | 0.908 |
|  | PTK2 | PIK3R1 | ENSP00000341189 | ENSP00000428056 | 0.935 |
|  | PTK2 | PTPN11 | ENSP00000341189 | ENSP00000340944 | 0.944 |
|  | PTK2 | SRC | ENSP00000341189 | ENSP00000362680 | 0.961 |
|  | PTK2 | SYK | ENSP00000341189 | ENSP00000364907 | 0.94 |
|  | PTPN11 | AKT1 | ENSP00000340944 | ENSP00000451828 | 0.912 |
|  | PTPN11 | EGFR | ENSP00000340944 | ENSP00000275493 | 0.979 |
|  | PTPN11 | IGF1R | ENSP00000340944 | ENSP00000268035 | 0.943 |
|  | PTPN11 | KDR | ENSP00000340944 | ENSP00000263923 | 0.941 |
|  | PTPN11 | MET | ENSP00000340944 | ENSP00000317272 | 0.944 |
|  | PTPN11 | PIK3R1 | ENSP00000340944 | ENSP00000428056 | 0.95 |
|  | PTPN11 | PTK2 | ENSP00000340944 | ENSP00000341189 | 0.944 |
|  | PTPN11 | PTPN6 | ENSP00000340944 | ENSP00000391592 | 0.935 |
|  | PTPN11 | SRC | ENSP00000340944 | ENSP00000362680 | 0.944 |
|  | PTPN11 | STAT3 | ENSP00000340944 | ENSP00000264657 | 0.941 |
|  | PTPN11 | SYK | ENSP00000340944 | ENSP00000364907 | 0.912 |
|  | PTPN6 | ARG1 | ENSP00000391592 | ENSP00000349446 | 0.9 |
|  | PTPN6 | KDR | ENSP00000391592 | ENSP00000263923 | 0.941 |
|  | PTPN6 | MMP9 | ENSP00000391592 | ENSP00000361405 | 0.9 |
|  | PTPN6 | PIK3R1 | ENSP00000391592 | ENSP00000428056 | 0.945 |
|  | PTPN6 | PTPN11 | ENSP00000391592 | ENSP00000340944 | 0.935 |
|  | PTPN6 | SYK | ENSP00000391592 | ENSP00000364907 | 0.943 |
|  | SRC | AKT1 | ENSP00000362680 | ENSP00000451828 | 0.952 |
|  | SRC | CAMK2B | ENSP00000362680 | ENSP00000379098 | 0.908 |
|  | SRC | CDK1 | ENSP00000362680 | ENSP00000378699 | 0.905 |
|  | SRC | EGFR | ENSP00000362680 | ENSP00000275493 | 0.946 |
|  | SRC | F2 | ENSP00000362680 | ENSP00000308541 | 0.9 |
|  | SRC | IGF1R | ENSP00000362680 | ENSP00000268035 | 0.938 |
|  | SRC | KDR | ENSP00000362680 | ENSP00000263923 | 0.943 |
|  | SRC | MET | ENSP00000362680 | ENSP00000317272 | 0.938 |
|  | SRC | MMP2 | ENSP00000362680 | ENSP00000219070 | 0.9 |
|  | SRC | MMP9 | ENSP00000362680 | ENSP00000361405 | 0.9 |
|  | SRC | PIK3R1 | ENSP00000362680 | ENSP00000428056 | 0.968 |
|  | SRC | PTK2 | ENSP00000362680 | ENSP00000341189 | 0.961 |
|  | SRC | PTPN11 | ENSP00000362680 | ENSP00000340944 | 0.944 |
|  | SRC | STAT3 | ENSP00000362680 | ENSP00000264657 | 0.94 |
|  | SRC | SYK | ENSP00000362680 | ENSP00000364907 | 0.936 |
|  | STAT3 | AKT1 | ENSP00000264657 | ENSP00000451828 | 0.908 |
|  | STAT3 | EGFR | ENSP00000264657 | ENSP00000275493 | 0.974 |
|  | STAT3 | MET | ENSP00000264657 | ENSP00000317272 | 0.94 |
|  | STAT3 | MMP2 | ENSP00000264657 | ENSP00000219070 | 0.9 |
|  | STAT3 | MMP3 | ENSP00000264657 | ENSP00000299855 | 0.935 |
|  | STAT3 | MMP9 | ENSP00000264657 | ENSP00000361405 | 0.9 |
|  | STAT3 | PIK3R1 | ENSP00000264657 | ENSP00000428056 | 0.949 |
|  | STAT3 | PIM1 | ENSP00000264657 | ENSP00000362608 | 0.927 |
|  | STAT3 | PTPN11 | ENSP00000264657 | ENSP00000340944 | 0.941 |
|  | STAT3 | SRC | ENSP00000264657 | ENSP00000362680 | 0.94 |
|  | SUZ12 | EED | ENSP00000316578 | ENSP00000263360 | 0.999 |
|  | SUZ12 | EZH2 | ENSP00000316578 | ENSP00000320147 | 0.998 |
|  | SYK | PIK3R1 | ENSP00000364907 | ENSP00000428056 | 0.938 |
|  | SYK | PTK2 | ENSP00000364907 | ENSP00000341189 | 0.94 |
|  | SYK | PTPN11 | ENSP00000364907 | ENSP00000340944 | 0.912 |
|  | SYK | PTPN6 | ENSP00000364907 | ENSP00000391592 | 0.943 |
|  | SYK | SRC | ENSP00000364907 | ENSP00000362680 | 0.936 |
|  | TERT | AKT1 | ENSP00000309572 | ENSP00000451828 | 0.935 |
|  | TERT | TNKS | ENSP00000309572 | ENSP00000311579 | 0.9 |
|  | TNKS | TERT | ENSP00000311579 | ENSP00000309572 | 0.9 |
|  | TNKS | TNKS2 | ENSP00000311579 | ENSP00000360689 | 0.927 |
|  | TNKS2 | TNKS | ENSP00000360689 | ENSP00000311579 | 0.927 |
|  | TTR | APP | ENSP00000237014 | ENSP00000284981 | 0.935 |
|  | TTR | ARG1 | ENSP00000237014 | ENSP00000349446 | 0.9 |
|  | TTR | MPO | ENSP00000237014 | ENSP00000225275 | 0.9 |
| IV | ADORA1 | APP | ENSP00000356205 | ENSP00000284981 | 0.9 |
|  | ADORA1 | CXCR1 | ENSP00000356205 | ENSP00000295683 | 0.9 |
|  | ADORA1 | DRD2 | ENSP00000356205 | ENSP00000354859 | 0.9 |
|  | ADORA1 | DRD4 | ENSP00000356205 | ENSP00000176183 | 0.9 |
|  | ADORA1 | HTR1A | ENSP00000356205 | ENSP00000316244 | 0.9 |
|  | ADORA1 | HTR5A | ENSP00000356205 | ENSP00000287907 | 0.9 |
|  | AKR1B1 | AKR1B10 | ENSP00000285930 | ENSP00000352584 | 0.9 |
|  | AKR1B1 | AKR1C3 | ENSP00000285930 | ENSP00000369927 | 0.9 |
|  | AKR1B10 | AKR1B1 | ENSP00000352584 | ENSP00000285930 | 0.9 |
|  | AKR1B10 | AKR1C3 | ENSP00000352584 | ENSP00000369927 | 0.9 |
|  | AKR1C1 | AKR1C2 | ENSP00000370254 | ENSP00000370129 | 0.985 |
|  | AKR1C1 | AKR1C3 | ENSP00000370254 | ENSP00000369927 | 0.994 |
|  | AKR1C2 | AKR1C1 | ENSP00000370129 | ENSP00000370254 | 0.985 |
|  | AKR1C2 | AKR1C3 | ENSP00000370129 | ENSP00000369927 | 0.981 |
|  | AKR1C3 | AKR1B1 | ENSP00000369927 | ENSP00000285930 | 0.9 |
|  | AKR1C3 | AKR1B10 | ENSP00000369927 | ENSP00000352584 | 0.9 |
|  | AKR1C3 | AKR1C1 | ENSP00000369927 | ENSP00000370254 | 0.994 |
|  | AKR1C3 | AKR1C2 | ENSP00000369927 | ENSP00000370129 | 0.981 |
|  | AKR1C3 | AKR1C4 | ENSP00000369927 | ENSP00000369814 | 0.933 |
|  | AKR1C3 | CBR1 | ENSP00000369927 | ENSP00000290349 | 0.9 |
|  | AKR1C3 | CYP19A1 | ENSP00000369927 | ENSP00000379683 | 0.9 |
|  | AKR1C3 | CYP1B1 | ENSP00000369927 | ENSP00000478561 | 0.9 |
|  | AKR1C4 | AKR1C3 | ENSP00000369814 | ENSP00000369927 | 0.933 |
|  | AKT1 | AURKA | ENSP00000451828 | ENSP00000216911 | 0.912 |
|  | AKT1 | GSK3B | ENSP00000451828 | ENSP00000324806 | 0.999 |
|  | AKT1 | INSR | ENSP00000451828 | ENSP00000303830 | 0.925 |
|  | AKT1 | LYN | ENSP00000451828 | ENSP00000428924 | 0.9 |
|  | AKT1 | MAP3K8 | ENSP00000451828 | ENSP00000263056 | 0.935 |
|  | AKT1 | PDGFRB | ENSP00000451828 | ENSP00000261799 | 0.905 |
|  | AKT1 | PIK3R1 | ENSP00000451828 | ENSP00000428056 | 0.942 |
|  | AKT1 | PTPN11 | ENSP00000451828 | ENSP00000340944 | 0.912 |
|  | AKT1 | SRC | ENSP00000451828 | ENSP00000362680 | 0.952 |
|  | AKT1 | STAT3 | ENSP00000451828 | ENSP00000264657 | 0.908 |
|  | AKT1 | TERT | ENSP00000451828 | ENSP00000309572 | 0.935 |
|  | ALOX12 | ALOX15 | ENSP00000251535 | ENSP00000458832 | 0.9 |
|  | ALOX12 | ALOX5 | ENSP00000251535 | ENSP00000363512 | 0.9 |
|  | ALOX15 | ALOX12 | ENSP00000458832 | ENSP00000251535 | 0.9 |
|  | ALOX15 | ALOX5 | ENSP00000458832 | ENSP00000363512 | 0.9 |
|  | ALOX5 | ALOX12 | ENSP00000363512 | ENSP00000251535 | 0.9 |
|  | ALOX5 | ALOX15 | ENSP00000363512 | ENSP00000458832 | 0.9 |
|  | APP | ADORA1 | ENSP00000284981 | ENSP00000356205 | 0.9 |
|  | APP | BACE1 | ENSP00000284981 | ENSP00000318585 | 0.938 |
|  | APP | CXCR1 | ENSP00000284981 | ENSP00000295683 | 0.9 |
|  | APP | DRD2 | ENSP00000284981 | ENSP00000354859 | 0.9 |
|  | APP | DRD4 | ENSP00000284981 | ENSP00000176183 | 0.9 |
|  | APP | F2 | ENSP00000284981 | ENSP00000308541 | 0.909 |
|  | APP | HTR1A | ENSP00000284981 | ENSP00000316244 | 0.9 |
|  | APP | HTR2B | ENSP00000284981 | ENSP00000258400 | 0.9 |
|  | APP | HTR5A | ENSP00000284981 | ENSP00000287907 | 0.9 |
|  | APP | PIK3R1 | ENSP00000284981 | ENSP00000428056 | 0.9 |
|  | APP | TTR | ENSP00000284981 | ENSP00000237014 | 0.935 |
|  | ARG1 | MPO | ENSP00000349446 | ENSP00000225275 | 0.9 |
|  | ARG1 | PTPN6 | ENSP00000349446 | ENSP00000391592 | 0.9 |
|  | ARG1 | TTR | ENSP00000349446 | ENSP00000237014 | 0.9 |
|  | AURKA | AKT1 | ENSP00000216911 | ENSP00000451828 | 0.912 |
|  | AURKA | AURKB | ENSP00000216911 | ENSP00000313950 | 0.905 |
|  | AURKA | CDK1 | ENSP00000216911 | ENSP00000378699 | 0.987 |
|  | AURKA | GSK3B | ENSP00000216911 | ENSP00000324806 | 0.936 |
|  | AURKA | NEK2 | ENSP00000216911 | ENSP00000355966 | 0.9 |
|  | AURKA | PLK1 | ENSP00000216911 | ENSP00000300093 | 0.957 |
|  | AURKA | PLK4 | ENSP00000216911 | ENSP00000270861 | 0.931 |
|  | AURKB | AURKA | ENSP00000313950 | ENSP00000216911 | 0.905 |
|  | AURKB | CCNB1 | ENSP00000313950 | ENSP00000256442 | 0.926 |
|  | AURKB | CCNB2 | ENSP00000313950 | ENSP00000288207 | 0.926 |
|  | AURKB | CDK1 | ENSP00000313950 | ENSP00000378699 | 0.937 |
|  | AURKB | PLK1 | ENSP00000313950 | ENSP00000300093 | 0.933 |
|  | AVPR2 | DRD1 | ENSP00000351805 | ENSP00000377353 | 0.9 |
|  | AVPR2 | EGFR | ENSP00000351805 | ENSP00000275493 | 0.904 |
|  | AVPR2 | HTR6 | ENSP00000351805 | ENSP00000289753 | 0.9 |
|  | AVPR2 | HTR7 | ENSP00000351805 | ENSP00000337949 | 0.9 |
|  | AXL | KDR | ENSP00000301178 | ENSP00000263923 | 0.905 |
|  | AXL | PIK3R1 | ENSP00000301178 | ENSP00000428056 | 0.937 |
|  | BACE1 | APP | ENSP00000318585 | ENSP00000284981 | 0.938 |
|  | BRAF | CAMK2B | ENSP00000288602 | ENSP00000379098 | 0.908 |
|  | BRAF | SRC | ENSP00000288602 | ENSP00000362680 | 0.912 |
|  | CAMK2B | BRAF | ENSP00000379098 | ENSP00000288602 | 0.908 |
|  | CAMK2B | SRC | ENSP00000379098 | ENSP00000362680 | 0.908 |
|  | CBR1 | AKR1C3 | ENSP00000290349 | ENSP00000369927 | 0.9 |
|  | CBR1 | PTGES | ENSP00000290349 | ENSP00000342385 | 0.9 |
|  | CCNA1 | CCNA2 | ENSP00000255465 | ENSP00000274026 | 0.917 |
|  | CCNA1 | CCNB1 | ENSP00000255465 | ENSP00000256442 | 0.904 |
|  | CCNA1 | CCND1 | ENSP00000255465 | ENSP00000227507 | 0.902 |
|  | CCNA1 | CDK1 | ENSP00000255465 | ENSP00000378699 | 0.951 |
|  | CCNA1 | CDK2 | ENSP00000255465 | ENSP00000266970 | 0.978 |
|  | CCNA2 | CCNA1 | ENSP00000274026 | ENSP00000255465 | 0.917 |
|  | CCNA2 | CCNB1 | ENSP00000274026 | ENSP00000256442 | 0.914 |
|  | CCNA2 | CDK1 | ENSP00000274026 | ENSP00000378699 | 0.976 |
|  | CCNA2 | CDK2 | ENSP00000274026 | ENSP00000266970 | 0.999 |
|  | CCNA2 | CDK4 | ENSP00000274026 | ENSP00000257904 | 0.999 |
|  | CCNB1 | AURKB | ENSP00000256442 | ENSP00000313950 | 0.926 |
|  | CCNB1 | CCNA1 | ENSP00000256442 | ENSP00000255465 | 0.904 |
|  | CCNB1 | CCNA2 | ENSP00000256442 | ENSP00000274026 | 0.914 |
|  | CCNB1 | CCNB2 | ENSP00000256442 | ENSP00000288207 | 0.994 |
|  | CCNB1 | CCNB3 | ENSP00000256442 | ENSP00000365210 | 0.944 |
|  | CCNB1 | CCND1 | ENSP00000256442 | ENSP00000227507 | 0.902 |
|  | CCNB1 | CDK1 | ENSP00000256442 | ENSP00000378699 | 0.999 |
|  | CCNB1 | CDK2 | ENSP00000256442 | ENSP00000266970 | 0.997 |
|  | CCNB1 | CDK4 | ENSP00000256442 | ENSP00000257904 | 0.923 |
|  | CCNB1 | CDK5 | ENSP00000256442 | ENSP00000419782 | 0.923 |
|  | CCNB1 | PLK1 | ENSP00000256442 | ENSP00000300093 | 0.976 |
|  | CCNB2 | AURKB | ENSP00000288207 | ENSP00000313950 | 0.926 |
|  | CCNB2 | CCNB1 | ENSP00000288207 | ENSP00000256442 | 0.994 |
|  | CCNB2 | CCNB3 | ENSP00000288207 | ENSP00000365210 | 0.944 |
|  | CCNB2 | CDK1 | ENSP00000288207 | ENSP00000378699 | 0.996 |
|  | CCNB2 | CDK2 | ENSP00000288207 | ENSP00000266970 | 0.999 |
|  | CCNB2 | CDK4 | ENSP00000288207 | ENSP00000257904 | 0.923 |
|  | CCNB2 | CDK5 | ENSP00000288207 | ENSP00000419782 | 0.902 |
|  | CCNB2 | PLK1 | ENSP00000288207 | ENSP00000300093 | 0.927 |
|  | CCNB3 | CCNB1 | ENSP00000365210 | ENSP00000256442 | 0.944 |
|  | CCNB3 | CCNB2 | ENSP00000365210 | ENSP00000288207 | 0.944 |
|  | CCNB3 | CDK1 | ENSP00000365210 | ENSP00000378699 | 0.957 |
|  | CCND1 | CCNA1 | ENSP00000227507 | ENSP00000255465 | 0.902 |
|  | CCND1 | CCNB1 | ENSP00000227507 | ENSP00000256442 | 0.902 |
|  | CCND1 | CDK1 | ENSP00000227507 | ENSP00000378699 | 0.932 |
|  | CCND1 | CDK2 | ENSP00000227507 | ENSP00000266970 | 0.983 |
|  | CCND1 | CDK4 | ENSP00000227507 | ENSP00000257904 | 0.999 |
|  | CCND1 | GSK3B | ENSP00000227507 | ENSP00000324806 | 0.95 |
|  | CCND1 | LYN | ENSP00000227507 | ENSP00000428924 | 0.9 |
|  | CCND1 | STAT3 | ENSP00000227507 | ENSP00000264657 | 0.943 |
|  | CDK1 | AURKA | ENSP00000378699 | ENSP00000216911 | 0.987 |
|  | CDK1 | AURKB | ENSP00000378699 | ENSP00000313950 | 0.937 |
|  | CDK1 | CCNA1 | ENSP00000378699 | ENSP00000255465 | 0.951 |
|  | CDK1 | CCNA2 | ENSP00000378699 | ENSP00000274026 | 0.976 |
|  | CDK1 | CCNB1 | ENSP00000378699 | ENSP00000256442 | 0.999 |
|  | CDK1 | CCNB2 | ENSP00000378699 | ENSP00000288207 | 0.996 |
|  | CDK1 | CCNB3 | ENSP00000378699 | ENSP00000365210 | 0.957 |
|  | CDK1 | CCND1 | ENSP00000378699 | ENSP00000227507 | 0.932 |
|  | CDK1 | CDK2 | ENSP00000378699 | ENSP00000266970 | 0.981 |
|  | CDK1 | CDK4 | ENSP00000378699 | ENSP00000257904 | 0.915 |
|  | CDK1 | CDK5 | ENSP00000378699 | ENSP00000419782 | 0.902 |
|  | CDK1 | NEK2 | ENSP00000378699 | ENSP00000355966 | 0.9 |
|  | CDK1 | PKN1 | ENSP00000378699 | ENSP00000343325 | 0.903 |
|  | CDK1 | PLK1 | ENSP00000378699 | ENSP00000300093 | 0.923 |
|  | CDK1 | PLK4 | ENSP00000378699 | ENSP00000270861 | 0.922 |
|  | CDK1 | SRC | ENSP00000378699 | ENSP00000362680 | 0.905 |
|  | CDK2 | CCNA1 | ENSP00000266970 | ENSP00000255465 | 0.978 |
|  | CDK2 | CCNA2 | ENSP00000266970 | ENSP00000274026 | 0.999 |
|  | CDK2 | CCNB1 | ENSP00000266970 | ENSP00000256442 | 0.997 |
|  | CDK2 | CCNB2 | ENSP00000266970 | ENSP00000288207 | 0.999 |
|  | CDK2 | CCND1 | ENSP00000266970 | ENSP00000227507 | 0.983 |
|  | CDK2 | CDK1 | ENSP00000266970 | ENSP00000378699 | 0.981 |
|  | CDK2 | CDK4 | ENSP00000266970 | ENSP00000257904 | 0.908 |
|  | CDK2 | CDK5 | ENSP00000266970 | ENSP00000419782 | 0.902 |
|  | CDK2 | POLA1 | ENSP00000266970 | ENSP00000368349 | 0.942 |
|  | CDK4 | CCNA2 | ENSP00000257904 | ENSP00000274026 | 0.999 |
|  | CDK4 | CCNB1 | ENSP00000257904 | ENSP00000256442 | 0.923 |
|  | CDK4 | CCNB2 | ENSP00000257904 | ENSP00000288207 | 0.923 |
|  | CDK4 | CCND1 | ENSP00000257904 | ENSP00000227507 | 0.999 |
|  | CDK4 | CDK1 | ENSP00000257904 | ENSP00000378699 | 0.915 |
|  | CDK4 | CDK2 | ENSP00000257904 | ENSP00000266970 | 0.908 |
|  | CDK4 | CDK5 | ENSP00000257904 | ENSP00000419782 | 0.905 |
|  | CDK4 | GSK3B | ENSP00000257904 | ENSP00000324806 | 0.905 |
|  | CDK4 | LYN | ENSP00000257904 | ENSP00000428924 | 0.912 |
|  | CDK5 | CCNB1 | ENSP00000419782 | ENSP00000256442 | 0.923 |
|  | CDK5 | CCNB2 | ENSP00000419782 | ENSP00000288207 | 0.902 |
|  | CDK5 | CDK1 | ENSP00000419782 | ENSP00000378699 | 0.902 |
|  | CDK5 | CDK2 | ENSP00000419782 | ENSP00000266970 | 0.902 |
|  | CDK5 | CDK4 | ENSP00000419782 | ENSP00000257904 | 0.905 |
|  | CDK5 | CDK5R1 | ENSP00000419782 | ENSP00000318486 | 0.997 |
|  | CDK5 | GSK3B | ENSP00000419782 | ENSP00000324806 | 0.937 |
|  | CDK5R1 | CDK5 | ENSP00000318486 | ENSP00000419782 | 0.997 |
|  | CDK5R1 | GSK3B | ENSP00000318486 | ENSP00000324806 | 0.904 |
|  | CES1 | MPO | ENSP00000353720 | ENSP00000225275 | 0.903 |
|  | CXCR1 | ADORA1 | ENSP00000295683 | ENSP00000356205 | 0.9 |
|  | CXCR1 | APP | ENSP00000295683 | ENSP00000284981 | 0.9 |
|  | CXCR1 | DRD2 | ENSP00000295683 | ENSP00000354859 | 0.9 |
|  | CXCR1 | DRD4 | ENSP00000295683 | ENSP00000176183 | 0.9 |
|  | CXCR1 | FGR | ENSP00000295683 | ENSP00000363117 | 0.903 |
|  | CXCR1 | HTR1A | ENSP00000295683 | ENSP00000316244 | 0.9 |
|  | CXCR1 | HTR5A | ENSP00000295683 | ENSP00000287907 | 0.9 |
|  | CXCR1 | LYN | ENSP00000295683 | ENSP00000428924 | 0.903 |
|  | CYP19A1 | AKR1C3 | ENSP00000379683 | ENSP00000369927 | 0.9 |
|  | CYP19A1 | HSD17B1 | ENSP00000379683 | ENSP00000466799 | 0.901 |
|  | CYP19A1 | HSD17B2 | ENSP00000379683 | ENSP00000199936 | 0.904 |
|  | CYP1B1 | AKR1C3 | ENSP00000478561 | ENSP00000369927 | 0.9 |
|  | CYP1B1 | HSD17B1 | ENSP00000478561 | ENSP00000466799 | 0.905 |
|  | CYP1B1 | HSD17B2 | ENSP00000478561 | ENSP00000199936 | 0.902 |
|  | DRD1 | AVPR2 | ENSP00000377353 | ENSP00000351805 | 0.9 |
|  | DRD1 | HTR6 | ENSP00000377353 | ENSP00000289753 | 0.9 |
|  | DRD1 | HTR7 | ENSP00000377353 | ENSP00000337949 | 0.9 |
|  | DRD2 | ADORA1 | ENSP00000354859 | ENSP00000356205 | 0.9 |
|  | DRD2 | APP | ENSP00000354859 | ENSP00000284981 | 0.9 |
|  | DRD2 | CXCR1 | ENSP00000354859 | ENSP00000295683 | 0.9 |
|  | DRD2 | DRD4 | ENSP00000354859 | ENSP00000176183 | 0.935 |
|  | DRD2 | HTR1A | ENSP00000354859 | ENSP00000316244 | 0.9 |
|  | DRD2 | HTR5A | ENSP00000354859 | ENSP00000287907 | 0.9 |
|  | DRD4 | ADORA1 | ENSP00000176183 | ENSP00000356205 | 0.9 |
|  | DRD4 | APP | ENSP00000176183 | ENSP00000284981 | 0.9 |
|  | DRD4 | CXCR1 | ENSP00000176183 | ENSP00000295683 | 0.9 |
|  | DRD4 | DRD2 | ENSP00000176183 | ENSP00000354859 | 0.935 |
|  | DRD4 | HTR1A | ENSP00000176183 | ENSP00000316244 | 0.9 |
|  | DRD4 | HTR5A | ENSP00000176183 | ENSP00000287907 | 0.9 |
|  | EED | EZH2 | ENSP00000263360 | ENSP00000320147 | 0.998 |
|  | EED | SUZ12 | ENSP00000263360 | ENSP00000316578 | 0.999 |
|  | EGFR | AVPR2 | ENSP00000275493 | ENSP00000351805 | 0.904 |
|  | EGFR | ERBB2 | ENSP00000275493 | ENSP00000269571 | 0.968 |
|  | EGFR | FGR | ENSP00000275493 | ENSP00000363117 | 0.94 |
|  | EGFR | LYN | ENSP00000275493 | ENSP00000428924 | 0.94 |
|  | EGFR | MMP3 | ENSP00000275493 | ENSP00000299855 | 0.9 |
|  | EGFR | PIK3R1 | ENSP00000275493 | ENSP00000428056 | 0.987 |
|  | EGFR | PTPN11 | ENSP00000275493 | ENSP00000340944 | 0.979 |
|  | EGFR | SRC | ENSP00000275493 | ENSP00000362680 | 0.946 |
|  | EGFR | STAT3 | ENSP00000275493 | ENSP00000264657 | 0.974 |
|  | EPHB4 | MMP2 | ENSP00000350896 | ENSP00000219070 | 0.9 |
|  | EPHB4 | MMP9 | ENSP00000350896 | ENSP00000361405 | 0.9 |
|  | EPHB4 | PIK3R1 | ENSP00000350896 | ENSP00000428056 | 0.904 |
|  | ERBB2 | EGFR | ENSP00000269571 | ENSP00000275493 | 0.968 |
|  | ERBB2 | PIK3R1 | ENSP00000269571 | ENSP00000428056 | 0.975 |
|  | ERBB2 | PTK2 | ENSP00000269571 | ENSP00000341189 | 0.947 |
|  | ERBB2 | PTPN11 | ENSP00000269571 | ENSP00000340944 | 0.979 |
|  | ERBB2 | SRC | ENSP00000269571 | ENSP00000362680 | 0.944 |
|  | EZH2 | EED | ENSP00000320147 | ENSP00000263360 | 0.998 |
|  | EZH2 | SUZ12 | ENSP00000320147 | ENSP00000316578 | 0.998 |
|  | F2 | APP | ENSP00000308541 | ENSP00000284981 | 0.909 |
|  | F2 | FGR | ENSP00000308541 | ENSP00000363117 | 0.9 |
|  | F2 | HTR2B | ENSP00000308541 | ENSP00000258400 | 0.9 |
|  | F2 | PIK3R1 | ENSP00000308541 | ENSP00000428056 | 0.9 |
|  | F2 | SRC | ENSP00000308541 | ENSP00000362680 | 0.9 |
|  | FGR | CXCR1 | ENSP00000363117 | ENSP00000295683 | 0.903 |
|  | FGR | EGFR | ENSP00000363117 | ENSP00000275493 | 0.94 |
|  | FGR | F2 | ENSP00000363117 | ENSP00000308541 | 0.9 |
|  | FGR | LYN | ENSP00000363117 | ENSP00000428924 | 0.9 |
|  | FGR | PDGFRB | ENSP00000363117 | ENSP00000261799 | 0.905 |
|  | FGR | PTK2 | ENSP00000363117 | ENSP00000341189 | 0.94 |
|  | FGR | SRC | ENSP00000363117 | ENSP00000362680 | 0.985 |
|  | FGR | STAT3 | ENSP00000363117 | ENSP00000264657 | 0.933 |
|  | FGR | SYK | ENSP00000363117 | ENSP00000364907 | 0.944 |
|  | FLT3 | PIK3R1 | ENSP00000241453 | ENSP00000428056 | 0.935 |
|  | FLT4 | KDR | ENSP00000261937 | ENSP00000263923 | 0.944 |
|  | FLT4 | PIK3R1 | ENSP00000261937 | ENSP00000428056 | 0.912 |
|  | FLT4 | PTK2 | ENSP00000261937 | ENSP00000341189 | 0.938 |
|  | FLT4 | SRC | ENSP00000261937 | ENSP00000362680 | 0.912 |
|  | GSK3B | AKT1 | ENSP00000324806 | ENSP00000451828 | 0.999 |
|  | GSK3B | AURKA | ENSP00000324806 | ENSP00000216911 | 0.936 |
|  | GSK3B | CCND1 | ENSP00000324806 | ENSP00000227507 | 0.95 |
|  | GSK3B | CDK4 | ENSP00000324806 | ENSP00000257904 | 0.905 |
|  | GSK3B | CDK5 | ENSP00000324806 | ENSP00000419782 | 0.937 |
|  | GSK3B | CDK5R1 | ENSP00000324806 | ENSP00000318486 | 0.904 |
|  | HSD17B1 | CYP19A1 | ENSP00000466799 | ENSP00000379683 | 0.901 |
|  | HSD17B1 | CYP1B1 | ENSP00000466799 | ENSP00000478561 | 0.905 |
|  | HSD17B2 | CYP19A1 | ENSP00000199936 | ENSP00000379683 | 0.904 |
|  | HSD17B2 | CYP1B1 | ENSP00000199936 | ENSP00000478561 | 0.902 |
|  | HTR1A | ADORA1 | ENSP00000316244 | ENSP00000356205 | 0.9 |
|  | HTR1A | APP | ENSP00000316244 | ENSP00000284981 | 0.9 |
|  | HTR1A | CXCR1 | ENSP00000316244 | ENSP00000295683 | 0.9 |
|  | HTR1A | DRD2 | ENSP00000316244 | ENSP00000354859 | 0.9 |
|  | HTR1A | DRD4 | ENSP00000316244 | ENSP00000176183 | 0.9 |
|  | HTR1A | HTR5A | ENSP00000316244 | ENSP00000287907 | 0.9 |
|  | HTR2B | APP | ENSP00000258400 | ENSP00000284981 | 0.9 |
|  | HTR2B | F2 | ENSP00000258400 | ENSP00000308541 | 0.9 |
|  | HTR2B | PIK3R1 | ENSP00000258400 | ENSP00000428056 | 0.901 |
|  | HTR5A | ADORA1 | ENSP00000287907 | ENSP00000356205 | 0.9 |
|  | HTR5A | APP | ENSP00000287907 | ENSP00000284981 | 0.9 |
|  | HTR5A | CXCR1 | ENSP00000287907 | ENSP00000295683 | 0.9 |
|  | HTR5A | DRD2 | ENSP00000287907 | ENSP00000354859 | 0.9 |
|  | HTR5A | DRD4 | ENSP00000287907 | ENSP00000176183 | 0.9 |
|  | HTR5A | HTR1A | ENSP00000287907 | ENSP00000316244 | 0.9 |
|  | HTR6 | AVPR2 | ENSP00000289753 | ENSP00000351805 | 0.9 |
|  | HTR6 | DRD1 | ENSP00000289753 | ENSP00000377353 | 0.9 |
|  | HTR6 | HTR7 | ENSP00000289753 | ENSP00000337949 | 0.9 |
|  | HTR7 | AVPR2 | ENSP00000337949 | ENSP00000351805 | 0.9 |
|  | HTR7 | DRD1 | ENSP00000337949 | ENSP00000377353 | 0.9 |
|  | HTR7 | HTR6 | ENSP00000337949 | ENSP00000289753 | 0.9 |
|  | IGF1R | INSR | ENSP00000268035 | ENSP00000303830 | 0.994 |
|  | IGF1R | PIK3R1 | ENSP00000268035 | ENSP00000428056 | 0.956 |
|  | IGF1R | PTPN11 | ENSP00000268035 | ENSP00000340944 | 0.943 |
|  | IGF1R | SRC | ENSP00000268035 | ENSP00000362680 | 0.938 |
|  | INSR | AKT1 | ENSP00000303830 | ENSP00000451828 | 0.925 |
|  | INSR | IGF1R | ENSP00000303830 | ENSP00000268035 | 0.994 |
|  | INSR | PIK3R1 | ENSP00000303830 | ENSP00000428056 | 0.944 |
|  | INSR | PTPN11 | ENSP00000303830 | ENSP00000340944 | 0.943 |
|  | KDR | AXL | ENSP00000263923 | ENSP00000301178 | 0.905 |
|  | KDR | FLT4 | ENSP00000263923 | ENSP00000261937 | 0.944 |
|  | KDR | PIK3R1 | ENSP00000263923 | ENSP00000428056 | 0.912 |
|  | KDR | PTK2 | ENSP00000263923 | ENSP00000341189 | 0.905 |
|  | KDR | PTPN11 | ENSP00000263923 | ENSP00000340944 | 0.941 |
|  | KDR | PTPN6 | ENSP00000263923 | ENSP00000391592 | 0.941 |
|  | KDR | SRC | ENSP00000263923 | ENSP00000362680 | 0.943 |
|  | LYN | AKT1 | ENSP00000428924 | ENSP00000451828 | 0.9 |
|  | LYN | CCND1 | ENSP00000428924 | ENSP00000227507 | 0.9 |
|  | LYN | CDK4 | ENSP00000428924 | ENSP00000257904 | 0.912 |
|  | LYN | CXCR1 | ENSP00000428924 | ENSP00000295683 | 0.903 |
|  | LYN | EGFR | ENSP00000428924 | ENSP00000275493 | 0.94 |
|  | LYN | FGR | ENSP00000428924 | ENSP00000363117 | 0.9 |
|  | LYN | PDGFRB | ENSP00000428924 | ENSP00000261799 | 0.905 |
|  | LYN | PIK3R1 | ENSP00000428924 | ENSP00000428056 | 0.925 |
|  | LYN | PTK2 | ENSP00000428924 | ENSP00000341189 | 0.94 |
|  | LYN | PTPN11 | ENSP00000428924 | ENSP00000340944 | 0.912 |
|  | LYN | PTPN6 | ENSP00000428924 | ENSP00000391592 | 0.943 |
|  | LYN | SRC | ENSP00000428924 | ENSP00000362680 | 0.932 |
|  | LYN | STAT3 | ENSP00000428924 | ENSP00000264657 | 0.937 |
|  | LYN | SYK | ENSP00000428924 | ENSP00000364907 | 0.943 |
|  | MAP3K8 | AKT1 | ENSP00000263056 | ENSP00000451828 | 0.935 |
|  | MET | PIK3R1 | ENSP00000317272 | ENSP00000428056 | 0.938 |
|  | MET | PTK2 | ENSP00000317272 | ENSP00000341189 | 0.908 |
|  | MET | PTPN11 | ENSP00000317272 | ENSP00000340944 | 0.944 |
|  | MET | SRC | ENSP00000317272 | ENSP00000362680 | 0.938 |
|  | MET | STAT3 | ENSP00000317272 | ENSP00000264657 | 0.94 |
|  | MMP13 | MMP2 | ENSP00000260302 | ENSP00000219070 | 0.9 |
|  | MMP13 | MMP3 | ENSP00000260302 | ENSP00000299855 | 0.9 |
|  | MMP13 | MMP9 | ENSP00000260302 | ENSP00000361405 | 0.9 |
|  | MMP2 | EPHB4 | ENSP00000219070 | ENSP00000350896 | 0.9 |
|  | MMP2 | MMP13 | ENSP00000219070 | ENSP00000260302 | 0.9 |
|  | MMP2 | MMP3 | ENSP00000219070 | ENSP00000299855 | 0.9 |
|  | MMP2 | MMP9 | ENSP00000219070 | ENSP00000361405 | 0.9 |
|  | MMP2 | SRC | ENSP00000219070 | ENSP00000362680 | 0.9 |
|  | MMP2 | STAT3 | ENSP00000219070 | ENSP00000264657 | 0.9 |
|  | MMP2 | TEK | ENSP00000219070 | ENSP00000369375 | 0.9 |
|  | MMP3 | EGFR | ENSP00000299855 | ENSP00000275493 | 0.9 |
|  | MMP3 | MMP13 | ENSP00000299855 | ENSP00000260302 | 0.9 |
|  | MMP3 | MMP2 | ENSP00000299855 | ENSP00000219070 | 0.9 |
|  | MMP3 | MMP9 | ENSP00000299855 | ENSP00000361405 | 0.9 |
|  | MMP3 | STAT3 | ENSP00000299855 | ENSP00000264657 | 0.935 |
|  | MMP9 | EPHB4 | ENSP00000361405 | ENSP00000350896 | 0.9 |
|  | MMP9 | MMP13 | ENSP00000361405 | ENSP00000260302 | 0.9 |
|  | MMP9 | MMP2 | ENSP00000361405 | ENSP00000219070 | 0.9 |
|  | MMP9 | MMP3 | ENSP00000361405 | ENSP00000299855 | 0.9 |
|  | MMP9 | PTPN6 | ENSP00000361405 | ENSP00000391592 | 0.9 |
|  | MMP9 | SRC | ENSP00000361405 | ENSP00000362680 | 0.9 |
|  | MMP9 | STAT3 | ENSP00000361405 | ENSP00000264657 | 0.9 |
|  | MPO | ARG1 | ENSP00000225275 | ENSP00000349446 | 0.9 |
|  | MPO | CES1 | ENSP00000225275 | ENSP00000353720 | 0.903 |
|  | MPO | TTR | ENSP00000225275 | ENSP00000237014 | 0.9 |
|  | NEK2 | AURKA | ENSP00000355966 | ENSP00000216911 | 0.9 |
|  | NEK2 | CDK1 | ENSP00000355966 | ENSP00000378699 | 0.9 |
|  | NEK2 | PLK1 | ENSP00000355966 | ENSP00000300093 | 0.9 |
|  | NEK2 | PLK4 | ENSP00000355966 | ENSP00000270861 | 0.9 |
|  | PARP1 | POLB | ENSP00000355759 | ENSP00000265421 | 0.973 |
|  | PDGFRB | AKT1 | ENSP00000261799 | ENSP00000451828 | 0.905 |
|  | PDGFRB | FGR | ENSP00000261799 | ENSP00000363117 | 0.905 |
|  | PDGFRB | LYN | ENSP00000261799 | ENSP00000428924 | 0.905 |
|  | PDGFRB | PIK3R1 | ENSP00000261799 | ENSP00000428056 | 0.944 |
|  | PDGFRB | PTPN11 | ENSP00000261799 | ENSP00000340944 | 0.969 |
|  | PDGFRB | SRC | ENSP00000261799 | ENSP00000362680 | 0.941 |
|  | PDGFRB | STAT3 | ENSP00000261799 | ENSP00000264657 | 0.969 |
|  | PIK3R1 | AKT1 | ENSP00000428056 | ENSP00000451828 | 0.942 |
|  | PIK3R1 | APP | ENSP00000428056 | ENSP00000284981 | 0.9 |
|  | PIK3R1 | AXL | ENSP00000428056 | ENSP00000301178 | 0.937 |
|  | PIK3R1 | EGFR | ENSP00000428056 | ENSP00000275493 | 0.987 |
|  | PIK3R1 | EPHB4 | ENSP00000428056 | ENSP00000350896 | 0.904 |
|  | PIK3R1 | ERBB2 | ENSP00000428056 | ENSP00000269571 | 0.975 |
|  | PIK3R1 | F2 | ENSP00000428056 | ENSP00000308541 | 0.9 |
|  | PIK3R1 | FLT3 | ENSP00000428056 | ENSP00000241453 | 0.935 |
|  | PIK3R1 | FLT4 | ENSP00000428056 | ENSP00000261937 | 0.912 |
|  | PIK3R1 | HTR2B | ENSP00000428056 | ENSP00000258400 | 0.901 |
|  | PIK3R1 | IGF1R | ENSP00000428056 | ENSP00000268035 | 0.956 |
|  | PIK3R1 | INSR | ENSP00000428056 | ENSP00000303830 | 0.944 |
|  | PIK3R1 | KDR | ENSP00000428056 | ENSP00000263923 | 0.912 |
|  | PIK3R1 | LYN | ENSP00000428056 | ENSP00000428924 | 0.925 |
|  | PIK3R1 | MET | ENSP00000428056 | ENSP00000317272 | 0.938 |
|  | PIK3R1 | PDGFRB | ENSP00000428056 | ENSP00000261799 | 0.944 |
|  | PIK3R1 | PTK2 | ENSP00000428056 | ENSP00000341189 | 0.935 |
|  | PIK3R1 | PTPN11 | ENSP00000428056 | ENSP00000340944 | 0.95 |
|  | PIK3R1 | PTPN6 | ENSP00000428056 | ENSP00000391592 | 0.945 |
|  | PIK3R1 | SRC | ENSP00000428056 | ENSP00000362680 | 0.968 |
|  | PIK3R1 | STAT3 | ENSP00000428056 | ENSP00000264657 | 0.949 |
|  | PIK3R1 | SYK | ENSP00000428056 | ENSP00000364907 | 0.938 |
|  | PIK3R1 | TEK | ENSP00000428056 | ENSP00000369375 | 0.937 |
|  | PIM1 | STAT3 | ENSP00000362608 | ENSP00000264657 | 0.927 |
|  | PKN1 | CDK1 | ENSP00000343325 | ENSP00000378699 | 0.903 |
|  | PLK1 | AURKA | ENSP00000300093 | ENSP00000216911 | 0.957 |
|  | PLK1 | AURKB | ENSP00000300093 | ENSP00000313950 | 0.933 |
|  | PLK1 | CCNB1 | ENSP00000300093 | ENSP00000256442 | 0.976 |
|  | PLK1 | CCNB2 | ENSP00000300093 | ENSP00000288207 | 0.927 |
|  | PLK1 | CDK1 | ENSP00000300093 | ENSP00000378699 | 0.923 |
|  | PLK1 | NEK2 | ENSP00000300093 | ENSP00000355966 | 0.9 |
|  | PLK1 | PLK4 | ENSP00000300093 | ENSP00000270861 | 0.914 |
|  | PLK4 | AURKA | ENSP00000270861 | ENSP00000216911 | 0.931 |
|  | PLK4 | CDK1 | ENSP00000270861 | ENSP00000378699 | 0.922 |
|  | PLK4 | NEK2 | ENSP00000270861 | ENSP00000355966 | 0.9 |
|  | PLK4 | PLK1 | ENSP00000270861 | ENSP00000300093 | 0.914 |
|  | POLA1 | CDK2 | ENSP00000368349 | ENSP00000266970 | 0.942 |
|  | POLB | PARP1 | ENSP00000265421 | ENSP00000355759 | 0.973 |
|  | PTGES | CBR1 | ENSP00000342385 | ENSP00000290349 | 0.9 |
|  | PTK2 | ERBB2 | ENSP00000341189 | ENSP00000269571 | 0.947 |
|  | PTK2 | FGR | ENSP00000341189 | ENSP00000363117 | 0.94 |
|  | PTK2 | FLT4 | ENSP00000341189 | ENSP00000261937 | 0.938 |
|  | PTK2 | KDR | ENSP00000341189 | ENSP00000263923 | 0.905 |
|  | PTK2 | LYN | ENSP00000341189 | ENSP00000428924 | 0.94 |
|  | PTK2 | MET | ENSP00000341189 | ENSP00000317272 | 0.908 |
|  | PTK2 | PIK3R1 | ENSP00000341189 | ENSP00000428056 | 0.935 |
|  | PTK2 | PTPN11 | ENSP00000341189 | ENSP00000340944 | 0.944 |
|  | PTK2 | SRC | ENSP00000341189 | ENSP00000362680 | 0.961 |
|  | PTK2 | SYK | ENSP00000341189 | ENSP00000364907 | 0.94 |
|  | PTPN11 | AKT1 | ENSP00000340944 | ENSP00000451828 | 0.912 |
|  | PTPN11 | EGFR | ENSP00000340944 | ENSP00000275493 | 0.979 |
|  | PTPN11 | ERBB2 | ENSP00000340944 | ENSP00000269571 | 0.979 |
|  | PTPN11 | IGF1R | ENSP00000340944 | ENSP00000268035 | 0.943 |
|  | PTPN11 | INSR | ENSP00000340944 | ENSP00000303830 | 0.943 |
|  | PTPN11 | KDR | ENSP00000340944 | ENSP00000263923 | 0.941 |
|  | PTPN11 | LYN | ENSP00000340944 | ENSP00000428924 | 0.912 |
|  | PTPN11 | MET | ENSP00000340944 | ENSP00000317272 | 0.944 |
|  | PTPN11 | PDGFRB | ENSP00000340944 | ENSP00000261799 | 0.969 |
|  | PTPN11 | PIK3R1 | ENSP00000340944 | ENSP00000428056 | 0.95 |
|  | PTPN11 | PTK2 | ENSP00000340944 | ENSP00000341189 | 0.944 |
|  | PTPN11 | PTPN6 | ENSP00000340944 | ENSP00000391592 | 0.935 |
|  | PTPN11 | SRC | ENSP00000340944 | ENSP00000362680 | 0.944 |
|  | PTPN11 | STAT3 | ENSP00000340944 | ENSP00000264657 | 0.941 |
|  | PTPN11 | SYK | ENSP00000340944 | ENSP00000364907 | 0.912 |
|  | PTPN11 | TEK | ENSP00000340944 | ENSP00000369375 | 0.937 |
|  | PTPN6 | ARG1 | ENSP00000391592 | ENSP00000349446 | 0.9 |
|  | PTPN6 | KDR | ENSP00000391592 | ENSP00000263923 | 0.941 |
|  | PTPN6 | LYN | ENSP00000391592 | ENSP00000428924 | 0.943 |
|  | PTPN6 | MMP9 | ENSP00000391592 | ENSP00000361405 | 0.9 |
|  | PTPN6 | PIK3R1 | ENSP00000391592 | ENSP00000428056 | 0.945 |
|  | PTPN6 | PTPN11 | ENSP00000391592 | ENSP00000340944 | 0.935 |
|  | PTPN6 | SYK | ENSP00000391592 | ENSP00000364907 | 0.943 |
|  | SRC | AKT1 | ENSP00000362680 | ENSP00000451828 | 0.952 |
|  | SRC | BRAF | ENSP00000362680 | ENSP00000288602 | 0.912 |
|  | SRC | CAMK2B | ENSP00000362680 | ENSP00000379098 | 0.908 |
|  | SRC | CDK1 | ENSP00000362680 | ENSP00000378699 | 0.905 |
|  | SRC | EGFR | ENSP00000362680 | ENSP00000275493 | 0.946 |
|  | SRC | ERBB2 | ENSP00000362680 | ENSP00000269571 | 0.944 |
|  | SRC | F2 | ENSP00000362680 | ENSP00000308541 | 0.9 |
|  | SRC | FGR | ENSP00000362680 | ENSP00000363117 | 0.985 |
|  | SRC | FLT4 | ENSP00000362680 | ENSP00000261937 | 0.912 |
|  | SRC | IGF1R | ENSP00000362680 | ENSP00000268035 | 0.938 |
|  | SRC | KDR | ENSP00000362680 | ENSP00000263923 | 0.943 |
|  | SRC | LYN | ENSP00000362680 | ENSP00000428924 | 0.932 |
|  | SRC | MET | ENSP00000362680 | ENSP00000317272 | 0.938 |
|  | SRC | MMP2 | ENSP00000362680 | ENSP00000219070 | 0.9 |
|  | SRC | MMP9 | ENSP00000362680 | ENSP00000361405 | 0.9 |
|  | SRC | PDGFRB | ENSP00000362680 | ENSP00000261799 | 0.941 |
|  | SRC | PIK3R1 | ENSP00000362680 | ENSP00000428056 | 0.968 |
|  | SRC | PTK2 | ENSP00000362680 | ENSP00000341189 | 0.961 |
|  | SRC | PTPN11 | ENSP00000362680 | ENSP00000340944 | 0.944 |
|  | SRC | STAT3 | ENSP00000362680 | ENSP00000264657 | 0.94 |
|  | SRC | SYK | ENSP00000362680 | ENSP00000364907 | 0.936 |
|  | STAT3 | AKT1 | ENSP00000264657 | ENSP00000451828 | 0.908 |
|  | STAT3 | CCND1 | ENSP00000264657 | ENSP00000227507 | 0.943 |
|  | STAT3 | EGFR | ENSP00000264657 | ENSP00000275493 | 0.974 |
|  | STAT3 | FGR | ENSP00000264657 | ENSP00000363117 | 0.933 |
|  | STAT3 | LYN | ENSP00000264657 | ENSP00000428924 | 0.937 |
|  | STAT3 | MET | ENSP00000264657 | ENSP00000317272 | 0.94 |
|  | STAT3 | MMP2 | ENSP00000264657 | ENSP00000219070 | 0.9 |
|  | STAT3 | MMP3 | ENSP00000264657 | ENSP00000299855 | 0.935 |
|  | STAT3 | MMP9 | ENSP00000264657 | ENSP00000361405 | 0.9 |
|  | STAT3 | PDGFRB | ENSP00000264657 | ENSP00000261799 | 0.969 |
|  | STAT3 | PIK3R1 | ENSP00000264657 | ENSP00000428056 | 0.949 |
|  | STAT3 | PIM1 | ENSP00000264657 | ENSP00000362608 | 0.927 |
|  | STAT3 | PTPN11 | ENSP00000264657 | ENSP00000340944 | 0.941 |
|  | STAT3 | SRC | ENSP00000264657 | ENSP00000362680 | 0.94 |
|  | SUZ12 | EED | ENSP00000316578 | ENSP00000263360 | 0.999 |
|  | SUZ12 | EZH2 | ENSP00000316578 | ENSP00000320147 | 0.998 |
|  | SYK | FGR | ENSP00000364907 | ENSP00000363117 | 0.944 |
|  | SYK | LYN | ENSP00000364907 | ENSP00000428924 | 0.943 |
|  | SYK | PIK3R1 | ENSP00000364907 | ENSP00000428056 | 0.938 |
|  | SYK | PTK2 | ENSP00000364907 | ENSP00000341189 | 0.94 |
|  | SYK | PTPN11 | ENSP00000364907 | ENSP00000340944 | 0.912 |
|  | SYK | PTPN6 | ENSP00000364907 | ENSP00000391592 | 0.943 |
|  | SYK | SRC | ENSP00000364907 | ENSP00000362680 | 0.936 |
|  | TEK | MMP2 | ENSP00000369375 | ENSP00000219070 | 0.9 |
|  | TEK | PIK3R1 | ENSP00000369375 | ENSP00000428056 | 0.937 |
|  | TEK | PTPN11 | ENSP00000369375 | ENSP00000340944 | 0.937 |
|  | TERT | AKT1 | ENSP00000309572 | ENSP00000451828 | 0.935 |
|  | TERT | TNKS | ENSP00000309572 | ENSP00000311579 | 0.9 |
|  | TNKS | TERT | ENSP00000311579 | ENSP00000309572 | 0.9 |
|  | TNKS | TNKS2 | ENSP00000311579 | ENSP00000360689 | 0.927 |
|  | TNKS2 | TNKS | ENSP00000360689 | ENSP00000311579 | 0.927 |
|  | TTR | APP | ENSP00000237014 | ENSP00000284981 | 0.935 |
|  | TTR | ARG1 | ENSP00000237014 | ENSP00000349446 | 0.9 |
|  | TTR | MPO | ENSP00000237014 | ENSP00000225275 | 0.9 |

**Table S9.** Interaction count of targets

| **Strategy** | **Target** | **Interaction count** |
| --- | --- | --- |
| I | SRC | 12 |
|  | PIK3R1 | 10 |
|  | AKR1C3 | 6 |
|  | CDK1 | 5 |
|  | EGFR | 4 |
|  | KDR | 4 |
|  | MMP2 | 4 |
|  | MMP3 | 4 |
|  | MMP9 | 4 |
|  | PKN1 | 4 |
|  | PTK2 | 4 |
|  | AKT1 | 3 |
|  | MET | 3 |
|  | MMP13 | 3 |
|  | ADORA1 | 2 |
|  | AKR1C1 | 2 |
|  | AKR1C2 | 2 |
|  | ALOX12 | 2 |
|  | ALOX15 | 2 |
|  | ALOX5 | 2 |
|  | AURKB | 2 |
|  | AXL | 2 |
|  | CXCR1 | 2 |
|  | CYP19A1 | 2 |
|  | CYP1B1 | 2 |
|  | DRD4 | 2 |
|  | F2 | 2 |
|  | HSD17B2 | 2 |
|  | IGF1R | 2 |
|  | NEK2 | 2 |
|  | AKR1B1 | 1 |
|  | AKR1C4 | 1 |
|  | AVPR2 | 1 |
|  | CAMK2B | 1 |
|  | FLT3 | 1 |
|  | GSK3B | 1 |
| II | CDK5 | 5 |
|  | CCNB1 | 4 |
|  | CCNB2 | 4 |
|  | CDK1 | 4 |
|  | PTPN6 | 4 |
|  | CCNB3 | 3 |
|  | MMP9 | 3 |
|  | PTPN11 | 3 |
|  | STAT3 | 3 |
|  | APP | 2 |
|  | ARG1 | 2 |
|  | CDK5R1 | 2 |
|  | EED | 2 |
|  | EZH2 | 2 |
|  | GSK3B | 2 |
|  | MMP2 | 2 |
|  | SUZ12 | 2 |
|  | SYK | 2 |
|  | TNKS | 2 |
|  | TTR | 2 |
|  | ADORA1 | 1 |
|  | AKR1B1 | 1 |
|  | AKR1B10 | 1 |
|  | PARP1 | 1 |
|  | POLB | 1 |
|  | TERT | 1 |
|  | TNKS2 | 1 |
| III | PIK3R1 | 15 |
|  | SRC | 15 |
|  | PTPN11 | 11 |
|  | STAT3 | 10 |
|  | CDK1 | 9 |
|  | AKR1C3 | 7 |
|  | APP | 7 |
|  | AKT1 | 6 |
|  | CCNB1 | 6 |
|  | CCNB2 | 6 |
|  | EGFR | 6 |
|  | KDR | 6 |
|  | MMP9 | 6 |
|  | PKN1 | 6 |
|  | PTK2 | 6 |
|  | PTPN6 | 6 |
|  | CDK5 | 5 |
|  | MET | 5 |
|  | MMP2 | 5 |
|  | MMP3 | 5 |
|  | SYK | 5 |
|  | AURKB | 4 |
|  | ADORA1 | 3 |
|  | ARG1 | 3 |
|  | CCNB3 | 3 |
|  | CXCR1 | 3 |
|  | CYP19A1 | 3 |
|  | CYP1B1 | 3 |
|  | DRD4 | 3 |
|  | F2 | 3 |
|  | GSK3B | 3 |
|  | IGF1R | 3 |
|  | MMP13 | 3 |
|  | MPO | 3 |
|  | TTR | 3 |
|  | AKR1B1 | 2 |
|  | AKR1B10 | 2 |
|  | AKR1C1 | 2 |
|  | AKR1C2 | 2 |
|  | ALOX12 | 2 |
|  | ALOX15 | 2 |
|  | ALOX5 | 2 |
|  | AXL | 2 |
|  | CDK5R1 | 2 |
|  | EED | 2 |
|  | EZH2 | 2 |
|  | HSD17B1 | 2 |
|  | HSD17B2 | 2 |
|  | NEK2 | 2 |
|  | SUZ12 | 2 |
|  | TERT | 2 |
|  | TNKS | 2 |
|  | AKR1C4 | 1 |
|  | AVPR2 | 1 |
|  | BACE1 | 1 |
|  | CAMK2B | 1 |
|  | CES1 | 1 |
|  | FLT3 | 1 |
|  | PARP1 | 1 |
|  | PIM1 | 1 |
|  | POLB | 1 |
|  | TNKS2 | 1 |
| IV | PIK3R1 | 23 |
|  | SRC | 21 |
|  | CDK1 | 16 |
|  | PTPN11 | 16 |
|  | LYN | 14 |
|  | STAT3 | 14 |
|  | SUZ12 | 12 |
|  | AKT1 | 11 |
|  | APP | 11 |
|  | CCNB1 | 11 |
|  | PTK2 | 10 |
|  | CDK2 | 9 |
|  | CDK4 | 9 |
|  | EGFR | 9 |
|  | FGR | 9 |
|  | AKR1C3 | 8 |
|  | CCNB2 | 8 |
|  | CCND1 | 8 |
|  | CXCR1 | 8 |
|  | AURKA | 7 |
|  | CDK5 | 7 |
|  | KDR | 7 |
|  | MMP2 | 7 |
|  | MMP9 | 7 |
|  | PDGFRB | 7 |
|  | PLK1 | 7 |
|  | PTPN6 | 7 |
|  | SYK | 7 |
|  | ADORA1 | 6 |
|  | DRD2 | 6 |
|  | DRD4 | 6 |
|  | GSK3B | 6 |
|  | HTR1A | 6 |
|  | HTR5A | 6 |
|  | AURKB | 5 |
|  | CCNA1 | 5 |
|  | CCNA2 | 5 |
|  | ERBB2 | 5 |
|  | F2 | 5 |
|  | MET | 5 |
|  | MMP3 | 5 |
|  | AVPR2 | 4 |
|  | FLT4 | 4 |
|  | IGF1R | 4 |
|  | INSR | 4 |
|  | NEK2 | 4 |
|  | PLK4 | 4 |
|  | ARG1 | 3 |
|  | CCNB3 | 3 |
|  | CYP19A1 | 3 |
|  | CYP1B1 | 3 |
|  | DRD1 | 3 |
|  | EPHB4 | 3 |
|  | HTR2B | 3 |
|  | HTR6 | 3 |
|  | HTR7 | 3 |
|  | MMP13 | 3 |
|  | MPO | 3 |
|  | TEK | 3 |
|  | TTR | 3 |
|  | AKR1B1 | 2 |
|  | AKR1B10 | 2 |
|  | AKR1C1 | 2 |
|  | AKR1C2 | 2 |
|  | ALOX12 | 2 |
|  | ALOX15 | 2 |
|  | ALOX5 | 2 |
|  | AXL | 2 |
|  | BRAF | 2 |
|  | CAMK2B | 2 |
|  | CBR1 | 2 |
|  | CDK5R1 | 2 |
|  | EED | 2 |
|  | EZH2 | 2 |
|  | HSD17B1 | 2 |
|  | HSD17B2 | 2 |
|  | TERT | 2 |
|  | TNKS | 2 |
|  | AKR1C4 | 1 |
|  | BACE1 | 1 |
|  | CES1 | 1 |
|  | FLT3 | 1 |
|  | MAP3K8 | 1 |
|  | PARP1 | 1 |
|  | PIM1 | 1 |
|  | PKN1 | 1 |
|  | POLA1 | 1 |
|  | POLB | 1 |
|  | PTGES | 1 |
|  | TNKS2 | 1 |

**Table S10.** Targets of three familiar drugs in DrugBank

| **Drug** | **Drug Name** | **Target** |
| --- | --- | --- |
| DB00335 | Atenolol | ADRB1 |
|  |  | ADRB2 |
|  |  | CYP2D6 |
|  |  | ABCB11 |
| DB00175 | Pravastatin | HMGCR |
|  |  | SLCO2B1 |
|  |  | SLCO1A2 |
|  |  | SLC22A6 |
|  |  | SLC22A8 |
|  |  | ABCC2 |
|  |  | SLC22A11 |
|  |  | ABCG2 |
|  |  | SLC22A7 |
|  |  | SLCO1B1 |
|  |  | SLC16A1 |
|  |  | ABCB11 |
|  |  | SLCO1B3 |
|  |  | HDAC2 |
| DB00571 | Propranolol | ADRB1 |
|  |  | ADRB2 |
|  |  | ADRB3 |
|  |  | HTR1A |
|  |  | HTR1B |
|  |  | CYP1A2 |
|  |  | CYP2D6 |
|  |  | ORM1 |
|  |  | CYP2C19 |
|  |  | CYP3A4 |
|  |  | CYP3A5 |
|  |  | CYP3A7 |
|  |  | SLC22A2 |
|  |  | CYP1A1 |
|  |  | MAOA |

**Table S11.** Symbols associated with coronary artery atherosclerosis in GeneCards

| **Symbol** | **Description** | **Score** | **Identifier (Y/N)** |
| --- | --- | --- | --- |
| APOE | Apolipoprotein E | 119.16 | N |
| APOB | Apolipoprotein B | 112.27 | N |
| ACE | Angiotensin I Converting Enzyme | 102.87 | N |
| APOA1 | Apolipoprotein A1 | 102.85 | N |
| NOS3 | Nitric Oxide Synthase 3 | 96.12 | N |
| LDLR | Low Density Lipoprotein Receptor | 95.91 | N |
| IL6 | Interleukin 6 | 90.14 | N |
| ABCA1 | ATP Binding Cassette Subfamily A Member 1 | 87.46 | N |
| ELN | Elastin | 85.72 | N |
| LPL | Lipoprotein Lipase | 77.8 | N |
| LIPC | Lipase C, Hepatic Type | 77.11 | N |
| CETP | Cholesteryl Ester Transfer Protein | 76.58 | N |
| CRP | C-Reactive Protein | 74.25 | N |
| PCSK9 | Proprotein Convertase Subtilisin/Kexin Type 9 | 72.28 | N |
| PPARG | Peroxisome Proliferator Activated Receptor Gamma | 71.62 | N |
| ALB | Albumin | 70.63 | N |
| ABCG5 | ATP Binding Cassette Subfamily G Member 5 | 67.09 | N |
| AGTR1 | Angiotensin II Receptor Type 1 | 65.92 | N |
| ACTA2 | Actin Alpha 2, Smooth Muscle | 65.9 | N |
| F2 | Coagulation Factor II, Thrombin | 64.01 | N |
| THBD | Thrombomodulin | 63.58 | N |
| ABCG8 | ATP Binding Cassette Subfamily G Member 8 | 62.19 | N |
| ENPP1 | Ectonucleotide Pyrophosphatase/Phosphodiesterase 1 | 60.98 | N |
| LCAT | Lecithin-Cholesterol Acyltransferase | 60.72 | N |
| MMP3 | Matrix Metallopeptidase 3 | 58.96 | N |
| EDN1 | Endothelin 1 | 58.95 | N |
| SERPINC1 | Serpin Family C Member 1 | 58.5 | N |
| IL10 | Interleukin 10 | 57.3 | N |
| APOC3 | Apolipoprotein C3 | 56.83 | N |
| APOA2 | Apolipoprotein A2 | 56.69 | N |
| APOA5 | Apolipoprotein A5 | 56.33 | N |
| PON1 | Paraoxonase 1 | 56.23 | N |
| MYH11 | Myosin Heavy Chain 11 | 55.94 | N |
| LMNA | Lamin A/C | 55.93 | N |
| FBN1 | Fibrillin 1 | 55.28 | N |
| CCL2 | C-C Motif Chemokine Ligand 2 | 54.51 | N |
| LPA | Lipoprotein(A) | 53.95 | N |
| SERPINE1 | Serpin Family E Member 1 | 53.02 | N |
| OLR1 | Oxidized Low Density Lipoprotein Receptor 1 | 52.91 | N |
| TLR4 | Toll Like Receptor 4 | 52.81 | N |
| SELP | Selectin P | 52.56 | N |
| TGFBR2 | Transforming Growth Factor Beta Receptor 2 | 52.27 | N |
| AGT | Angiotensinogen | 52.27 | N |
| TGFBR1 | Transforming Growth Factor Beta Receptor 1 | 51.95 | N |
| TNF | Tumor Necrosis Factor | 51.72 | N |
| MTHFR | Methylenetetrahydrofolate Reductase | 51.7 | N |
| SMAD3 | SMAD Family Member 3 | 51.48 | N |
| MMP9 | Matrix Metallopeptidase 9 | 50.98 | N |
| PLAT | Plasminogen Activator, Tissue Type | 50.48 | N |
| ADIPOQ | Adiponectin, C1Q And Collagen Domain Containing | 50.42 | N |
| SELE | Selectin E | 50.32 | N |
| VEGFA | Vascular Endothelial Growth Factor A | 49.85 | N |
| ESR1 | Estrogen Receptor 1 | 48.96 | N |
| ENG | Endoglin | 48.72 | N |
| TGFB2 | Transforming Growth Factor Beta 2 | 48.47 | N |
| TP53 | Tumor Protein P53 | 48.22 | N |
| SLC2A10 | Solute Carrier Family 2 Member 10 | 47.4 | N |
| INS | Insulin | 47.24 | N |
| REN | Renin | 46.38 | N |
| VWF | Von Willebrand Factor | 46.17 | N |
| SPP1 | Secreted Phosphoprotein 1 | 45.91 | N |
| LDLRAP1 | Low Density Lipoprotein Receptor Adaptor Protein 1 | 45.88 | N |
| NPPB | Natriuretic Peptide B | 45.76 | N |
| MMP2 | Matrix Metallopeptidase 2 | 45.66 | N |
| F5 | Coagulation Factor V | 45.65 | N |
| VCAM1 | Vascular Cell Adhesion Molecule 1 | 45.07 | N |
| CST3 | Cystatin C | 44.8 | N |
| F3 | Coagulation Factor III, Tissue Factor | 44.8 | N |
| PRKG1 | Protein Kinase CGMP-Dependent 1 | 44.65 | N |
| MGP | Matrix Gla Protein | 44.48 | N |
| MYLK | Myosin Light Chain Kinase | 44.18 | N |
| COL3A1 | Collagen Type III Alpha 1 Chain | 44.04 | N |
| KCNJ5 | Potassium Inwardly Rectifying Channel Subfamily J Member 5 | 43.66 | N |
| TNNI3 | Troponin I3, Cardiac Type | 43.61 | N |
| TGFB3 | Transforming Growth Factor Beta 3 | 43.56 | N |
| ABCC6 | ATP Binding Cassette Subfamily C Member 6 | 43.46 | N |
| LOX | Lysyl Oxidase | 42.85 | N |
| HMGCR | 3-Hydroxy-3-Methylglutaryl-CoA Reductase | 42.69 | N |
| TNNT2 | Troponin T2, Cardiac Type | 42.29 | N |
| BMPR2 | Bone Morphogenetic Protein Receptor Type 2 | 41.5 | N |
| ICAM1 | Intercellular Adhesion Molecule 1 | 41.21 | N |
| NOS2 | Nitric Oxide Synthase 2 | 40.64 | N |
| NOTCH1 | Notch Receptor 1 | 40.31 | N |
| MIR126 | MicroRNA 126 | 40.3 | N |
| ITGB3 | Integrin Subunit Beta 3 | 40.3 | N |
| CYP7A1 | Cytochrome P450 Family 7 Subfamily A Member 1 | 40.25 | N |
| IGF1 | Insulin Like Growth Factor 1 | 40.06 | N |
| MEF2A | Myocyte Enhancer Factor 2A | 38.99 | N |
| ACVRL1 | Activin A Receptor Like Type 1 | 38.88 | N |
| CX3CR1 | C-X3-C Motif Chemokine Receptor 1 | 38.83 | N |
| GATA4 | GATA Binding Protein 4 | 38.61 | N |
| KNG1 | Kininogen 1 | 38.33 | N |
| CAT | Catalase | 37.78 | N |
| COG2 | Component Of Oligomeric Golgi Complex 2 | 37.71 | N |
| CBS | Cystathionine Beta-Synthase | 37.27 | N |
| F7 | Coagulation Factor VII | 37.15 | N |
| FGB | Fibrinogen Beta Chain | 37.05 | N |
| IL1B | Interleukin 1 Beta | 36.85 | N |
| MPO | Myeloperoxidase | 36.69 | N |
| FGA | Fibrinogen Alpha Chain | 36.14 | N |
| NKX2-5 | NK2 Homeobox 5 | 36.1 | N |
| PLA2G7 | Phospholipase A2 Group VII | 35.98 | N |
| MIR21 | MicroRNA 21 | 35.93 | N |
| GJA1 | Gap Junction Protein Alpha 1 | 35.51 | N |
| CXCL8 | C-X-C Motif Chemokine Ligand 8 | 35.38 | N |
| LEP | Leptin | 34.99 | N |
| MIR145 | MicroRNA 145 | 34.76 | N |
| CAV1 | Caveolin 1 | 34.41 | N |
| TIMP1 | TIMP Metallopeptidase Inhibitor 1 | 33.57 | N |
| CD40LG | CD40 Ligand | 33.49 | N |
| TNFRSF11B | TNF Receptor Superfamily Member 11b | 33.15 | N |
| WRN | WRN RecQ Like Helicase | 33.1 | N |
| TGFB1 | Transforming Growth Factor Beta 1 | 32.93 | N |
| AKT1 | AKT Serine/Threonine Kinase 1 | 32.72 | N |
| LRP6 | LDL Receptor Related Protein 6 | 32.7 | N |
| IL18 | Interleukin 18 | 32.66 | N |
| MFAP5 | Microfibril Associated Protein 5 | 32.32 | N |
| MIR155 | MicroRNA 155 | 32.1 | N |
| ZMPSTE24 | Zinc Metallopeptidase STE24 | 31.85 | N |
| RETN | Resistin | 31.83 | N |
| NPPA | Natriuretic Peptide A | 31.81 | N |
| CDKN2B-AS1 | CDKN2B Antisense RNA 1 | 31.49 | N |
| PPARA | Peroxisome Proliferator Activated Receptor Alpha | 30.88 | N |
| TNFRSF1A | TNF Receptor Superfamily Member 1A | 30.73 | N |
| GP1BA | Glycoprotein Ib Platelet Subunit Alpha | 30.25 | N |
| AGER | Advanced Glycosylation End-Product Specific Receptor | 30.15 | N |
| APP | Amyloid Beta Precursor Protein | 30.11 | N |
| LIPA | Lipase A, Lysosomal Acid Type | 30.07 | N |
| CYBA | Cytochrome B-245 Alpha Chain | 30.05 | N |
| FBLN5 | Fibulin 5 | 30.02 | Y |
| EDNRB | Endothelin Receptor Type B | 29.89 | N |
| GATA6 | GATA Binding Protein 6 | 29.83 | N |
| SCARB1 | Scavenger Receptor Class B Member 1 | 29.69 | N |
| SCN5A | Sodium Voltage-Gated Channel Alpha Subunit 5 | 29.63 | N |
| SMAD4 | SMAD Family Member 4 | 29.47 | N |
| MYH7 | Myosin Heavy Chain 7 | 29.33 | N |
| STAT3 | Signal Transducer And Activator Of Transcription 3 | 28.99 | N |
| PON2 | Paraoxonase 2 | 28.96 | N |
| GGCX | Gamma-Glutamyl Carboxylase | 28.76 | N |
| MYH6 | Myosin Heavy Chain 6 | 28.62 | N |
| PF4 | Platelet Factor 4 | 28.58 | N |
| MIR208A | MicroRNA 208a | 28.45 | N |
| F13A1 | Coagulation Factor XIII A Chain | 28.05 | N |
| ITGAM | Integrin Subunit Alpha M | 28.02 | N |
| EFEMP2 | EGF Containing Fibulin Extracellular Matrix Protein 2 | 27.71 | N |
| MAT2A | Methionine Adenosyltransferase 2A | 27.7 | N |
| ADM | Adrenomedullin | 27.57 | N |
| PTGIS | Prostaglandin I2 Synthase | 27.29 | N |
| PTGS2 | Prostaglandin-Endoperoxide Synthase 2 | 27.15 | N |
| MMP1 | Matrix Metallopeptidase 1 | 27.02 | N |
| ADD1 | Adducin 1 | 26.93 | N |
| MIR17 | MicroRNA 17 | 26.88 | N |
| HMOX1 | Heme Oxygenase 1 | 26.88 | N |
| HP | Haptoglobin | 26.34 | N |
| EPHX2 | Epoxide Hydrolase 2 | 26.32 | N |
| FOXE3 | Forkhead Box E3 | 26.15 | N |
| GUCY1A1 | Guanylate Cyclase 1 Soluble Subunit Alpha 1 | 26.09 | N |
| PECAM1 | Platelet And Endothelial Cell Adhesion Molecule 1 | 26.07 | N |
| CELA2A | Chymotrypsin Like Elastase 2A | 25.98 | N |
| HTR2A | 5-Hydroxytryptamine Receptor 2A | 25.97 | N |
| PLG | Plasminogen | 25.87 | N |
| CD36 | CD36 Molecule | 25.85 | N |
| MB | Myoglobin | 25.71 | N |
| PLTP | Phospholipid Transfer Protein | 25.69 | N |
| FOS | Fos Proto-Oncogene, AP-1 Transcription Factor Subunit | 25.56 | N |
| TNFSF4 | TNF Superfamily Member 4 | 25.49 | N |
| GNB3 | G Protein Subunit Beta 3 | 25.47 | N |
| GPT | Glutamic--Pyruvic Transaminase | 25.34 | N |
| PIK3C2A | Phosphatidylinositol-4-Phosphate 3-Kinase Catalytic Subunit Type 2 Alpha | 25.31 | N |
| TBX5 | T-Box Transcription Factor 5 | 25.28 | N |
| KDR | Kinase Insert Domain Receptor | 25.24 | N |
| GJA5 | Gap Junction Protein Alpha 5 | 25.11 | N |
| MIR33A | MicroRNA 33a | 25.05 | N |
| IL1RN | Interleukin 1 Receptor Antagonist | 24.96 | N |
| IFNG | Interferon Gamma | 24.82 | N |
| JAK2 | Janus Kinase 2 | 24.81 | N |
| LTBP4 | Latent Transforming Growth Factor Beta Binding Protein 4 | 24.76 | N |
| EDNRA | Endothelin Receptor Type A | 24.6 | N |
| ATHS | Atherosclerosis Susceptibility (Lipoprotein Associated) | 24.6 | Y |
| LTA | Lymphotoxin Alpha | 24.6 | N |
| CYP2C19 | Cytochrome P450 Family 2 Subfamily C Member 19 | 24.53 | N |
| NAMPT | Nicotinamide Phosphoribosyltransferase | 24.49 | N |
| NPY | Neuropeptide Y | 24.47 | N |
| VCL | Vinculin | 24.42 | N |
| APOC2 | Apolipoprotein C2 | 24.4 | N |
| SOD1 | Superoxide Dismutase 1 | 24.31 | N |
| HSPD1 | Heat Shock Protein Family D (Hsp60) Member 1 | 24.15 | N |
| CCN2 | Cellular Communication Network Factor 2 | 24.14 | N |
| ABCC9 | ATP Binding Cassette Subfamily C Member 9 | 23.97 | N |
| CDKN2A | Cyclin Dependent Kinase Inhibitor 2A | 23.94 | N |
| FGF2 | Fibroblast Growth Factor 2 | 23.71 | N |
| COL4A1 | Collagen Type IV Alpha 1 Chain | 23.62 | N |
| CEP19 | Centrosomal Protein 19 | 23.61 | N |
| APOH | Apolipoprotein H | 23.6 | N |
| PTPN11 | Protein Tyrosine Phosphatase Non-Receptor Type 11 | 23.54 | N |
| PPBP | Pro-Platelet Basic Protein | 23.45 | N |
| HLA-DRB1 | Major Histocompatibility Complex, Class II, DR Beta 1 | 23.19 | N |
| ADRB1 | Adrenoceptor Beta 1 | 23.13 | N |
| MTTP | Microsomal Triglyceride Transfer Protein | 23.13 | N |
| AGPAT2 | 1-Acylglycerol-3-Phosphate O-Acyltransferase 2 | 23.11 | N |
| GGT1 | Gamma-Glutamyltransferase 1 | 23.07 | N |
| JAG1 | Jagged Canonical Notch Ligand 1 | 23 | N |
| FN1 | Fibronectin 1 | 22.96 | N |
| APOA4 | Apolipoprotein A4 | 22.92 | N |
| MIR210 | MicroRNA 210 | 22.66 | N |
| XYLT1 | Xylosyltransferase 1 | 22.64 | N |
| ALOX5AP | Arachidonate 5-Lipoxygenase Activating Protein | 22.64 | N |
| NT5E | 5'-Nucleotidase Ecto | 22.61 | N |
| NOTCH3 | Notch Receptor 3 | 22.61 | N |
| IL17A | Interleukin 17A | 22.6 | N |
| XYLT2 | Xylosyltransferase 2 | 22.58 | N |
| MIR499A | MicroRNA 499a | 22.57 | N |
| P2RY12 | Purinergic Receptor P2Y12 | 22.45 | N |
| CD40 | CD40 Molecule | 22.41 | N |
| CXCL12 | C-X-C Motif Chemokine Ligand 12 | 22.28 | N |
| CD14 | CD14 Molecule | 22.26 | N |
| ALOX5 | Arachidonate 5-Lipoxygenase | 22.12 | N |
| GATA5 | GATA Binding Protein 5 | 22.11 | N |
| FABP4 | Fatty Acid Binding Protein 4 | 22.03 | N |
| FAS | Fas Cell Surface Death Receptor | 21.97 | N |
| LRP1 | LDL Receptor Related Protein 1 | 21.86 | N |
| CASP3 | Caspase 3 | 21.76 | N |
| BGN | Biglycan | 21.75 | N |
| FCGR2A | Fc Fragment Of IgG Receptor IIa | 21.75 | N |
| LIPG | Lipase G, Endothelial Type | 21.74 | N |
| GHR | Growth Hormone Receptor | 21.7 | N |
| ADRB2 | Adrenoceptor Beta 2 | 21.52 | N |
| SOD2 | Superoxide Dismutase 2 | 21.46 | N |
| EPO | Erythropoietin | 21.37 | N |
| CDKN2B | Cyclin Dependent Kinase Inhibitor 2B | 21.37 | N |
| PTGS1 | Prostaglandin-Endoperoxide Synthase 1 | 21.3 | N |
| SELL | Selectin L | 21.11 | N |
| ABCG1 | ATP Binding Cassette Subfamily G Member 1 | 21.08 | N |
| CAVIN1 | Caveolae Associated Protein 1 | 21.08 | N |
| FABP3 | Fatty Acid Binding Protein 3 | 21.02 | N |
| CALCA | Calcitonin Related Polypeptide Alpha | 20.88 | N |
| BANF1 | BAF Nuclear Assembly Factor 1 | 20.82 | N |
| ABCA3 | ATP Binding Cassette Subfamily A Member 3 | 20.82 | N |
| IL4 | Interleukin 4 | 20.81 | N |
| SOD3 | Superoxide Dismutase 3 | 20.74 | N |
| CXCR4 | C-X-C Motif Chemokine Receptor 4 | 20.73 | N |
| PHACTR1 | Phosphatase And Actin Regulator 1 | 20.68 | N |
| ACTC1 | Actin Alpha Cardiac Muscle 1 | 20.64 | N |
| BSCL2 | BSCL2 Lipid Droplet Biogenesis Associated, Seipin | 20.58 | N |
| ELANE | Elastase, Neutrophil Expressed | 20.4 | N |
| PTX3 | Pentraxin 3 | 20.29 | N |
| STAT1 | Signal Transducer And Activator Of Transcription 1 | 20.16 | N |
| SOAT1 | Sterol O-Acyltransferase 1 | 20.15 | N |
| NPC1 | NPC Intracellular Cholesterol Transporter 1 | 20.12 | N |
| RHOA | Ras Homolog Family Member A | 20.09 | N |
| SMPD1 | Sphingomyelin Phosphodiesterase 1 | 20.03 | N |
| ITGA2 | Integrin Subunit Alpha 2 | 20.01 | N |
| ZNF687 | Zinc Finger Protein 687 | 20.01 | N |
| SREBF2 | Sterol Regulatory Element Binding Transcription Factor 2 | 20.01 | N |
| AHSG | Alpha 2-HS Glycoprotein | 19.99 | N |
| LCN2 | Lipocalin 2 | 19.92 | N |
| CTLA4 | Cytotoxic T-Lymphocyte Associated Protein 4 | 19.92 | N |
| APLN | Apelin | 19.86 | N |
| TIMP2 | TIMP Metallopeptidase Inhibitor 2 | 19.86 | N |
| MIR140 | MicroRNA 140 | 19.84 | N |
| MAPK1 | Mitogen-Activated Protein Kinase 1 | 19.81 | N |
| PAPPA | Pappalysin 1 | 19.75 | N |
| RNF213 | Ring Finger Protein 213 | 19.74 | N |
| HIF1A | Hypoxia Inducible Factor 1 Subunit Alpha | 19.7 | N |
| ITGA2B | Integrin Subunit Alpha 2b | 19.68 | N |
| CCL5 | C-C Motif Chemokine Ligand 5 | 19.67 | N |
| B2M | Beta-2-Microglobulin | 19.54 | N |
| PGF | Placental Growth Factor | 19.46 | N |
| TBX20 | T-Box Transcription Factor 20 | 19.43 | N |
| ANGPT1 | Angiopoietin 1 | 19.39 | N |
| KITLG | KIT Ligand | 19.3 | N |
| C1S | Complement C1s | 19.28 | N |
| RAF1 | Raf-1 Proto-Oncogene, Serine/Threonine Kinase | 19.24 | N |
| LDB3 | LIM Domain Binding 3 | 19.23 | N |
| SLC6A4 | Solute Carrier Family 6 Member 4 | 19.12 | N |
| MIR146A | MicroRNA 146a | 19.08 | N |
| H19 | H19 Imprinted Maternally Expressed Transcript | 19.05 | N |
| SIRT1 | Sirtuin 1 | 19.02 | N |
| CHKB | Choline Kinase Beta | 19.01 | N |
| CCR5 | C-C Motif Chemokine Receptor 5 (Gene/Pseudogene) | 18.98 | N |
| COL5A1 | Collagen Type V Alpha 1 Chain | 18.77 | N |
| TLR2 | Toll Like Receptor 2 | 18.74 | N |
| GNAS | GNAS Complex Locus | 18.73 | N |
| PDE5A | Phosphodiesterase 5A | 18.69 | N |
| CD4 | CD4 Molecule | 18.62 | N |
| MTR | 5-Methyltetrahydrofolate-Homocysteine Methyltransferase | 18.58 | N |
| UTS2 | Urotensin 2 | 18.47 | N |
| BMP6 | Bone Morphogenetic Protein 6 | 18.45 | N |
| ANGPTL3 | Angiopoietin Like 3 | 18.43 | N |
| F10 | Coagulation Factor X | 18.4 | N |
| ANXA5 | Annexin A5 | 18.38 | N |
| PON3 | Paraoxonase 3 | 18.38 | N |
| IL6ST | Interleukin 6 Signal Transducer | 18.36 | N |
| MIR92B | MicroRNA 92b | 18.31 | N |
| MYOCD | Myocardin | 18.31 | N |
| NOX4 | NADPH Oxidase 4 | 18.22 | N |
| F8 | Coagulation Factor VIII | 18.12 | N |
| GHRL | Ghrelin And Obestatin Prepropeptide | 18.09 | N |
| COMT | Catechol-O-Methyltransferase | 18.07 | N |
| NOS1 | Nitric Oxide Synthase 1 | 18.05 | N |
| F2R | Coagulation Factor II Thrombin Receptor | 18.04 | N |
| CYP2C9 | Cytochrome P450 Family 2 Subfamily C Member 9 | 18.02 | N |
| CLU | Clusterin | 18.01 | N |
| CFH | Complement Factor H | 17.97 | N |
| CFTR | CF Transmembrane Conductance Regulator | 17.95 | N |
| CYP11B2 | Cytochrome P450 Family 11 Subfamily B Member 2 | 17.9 | N |
| CALR | Calreticulin | 17.9 | N |
| MAPK14 | Mitogen-Activated Protein Kinase 14 | 17.83 | N |
| AHSP | Alpha Hemoglobin Stabilizing Protein | 17.72 | N |
| MSR1 | Macrophage Scavenger Receptor 1 | 17.7 | N |
| AGTR2 | Angiotensin II Receptor Type 2 | 17.7 | N |
| CD55 | CD55 Molecule (Cromer Blood Group) | 17.69 | N |
| TNFRSF1B | TNF Receptor Superfamily Member 1B | 17.69 | N |
| SOS1 | SOS Ras/Rac Guanine Nucleotide Exchange Factor 1 | 17.68 | N |
| HGF | Hepatocyte Growth Factor | 17.66 | N |
| LGALS2 | Galectin 2 | 17.63 | N |
| XDH | Xanthine Dehydrogenase | 17.52 | N |
| NPPC | Natriuretic Peptide C | 17.5 | N |
| ANKRD1 | Ankyrin Repeat Domain 1 | 17.47 | N |
| SHBG | Sex Hormone Binding Globulin | 17.46 | N |
| ALDH2 | Aldehyde Dehydrogenase 2 Family Member | 17.42 | N |
| PPP1R17 | Protein Phosphatase 1 Regulatory Subunit 17 | 17.41 | N |
| SERPIND1 | Serpin Family D Member 1 | 17.3 | N |
| ALMS1 | ALMS1 Centrosome And Basal Body Associated Protein | 17.29 | N |
| CDH5 | Cadherin 5 | 17.26 | N |
| HMGB1 | High Mobility Group Box 1 | 17.14 | N |
| HGD | Homogentisate 1,2-Dioxygenase | 17.14 | N |
| CHDS2 | Coronary Heart Disease, Susceptibility To, 2 | 17.08 | Y |
| CHDS3 | Coronary Heart Disease, Susceptibility To, 3 | 17.08 | Y |
| GDF15 | Growth Differentiation Factor 15 | 17.08 | N |
| MT-CO1 | Mitochondrially Encoded Cytochrome C Oxidase I | 17.05 | N |
| TAGLN | Transgelin | 17.05 | N |
| PSMA6 | Proteasome 20S Subunit Alpha 6 | 17.02 | N |
| BLK | BLK Proto-Oncogene, Src Family Tyrosine Kinase | 17 | N |
| GP6 | Glycoprotein VI Platelet | 16.98 | N |
| CHDS4 | Coronary Heart Disease, Susceptibility To, 4 | 16.95 | Y |
| MIAT | Myocardial Infarction Associated Transcript | 16.93 | N |
| CCR2 | C-C Motif Chemokine Receptor 2 | 16.93 | N |
| TTN | Titin | 16.88 | N |
| CD34 | CD34 Molecule | 16.84 | N |
| IL2 | Interleukin 2 | 16.76 | N |
| ACE2 | Angiotensin I Converting Enzyme 2 | 16.71 | N |
| COL5A2 | Collagen Type V Alpha 2 Chain | 16.71 | N |
| GCLM | Glutamate-Cysteine Ligase Modifier Subunit | 16.68 | N |
| SH2B3 | SH2B Adaptor Protein 3 | 16.64 | N |
| CHDS8 | Coronary Heart Disease, Susceptibility To, 8 | 16.52 | Y |
| CHDS9 | Coronary Heart Disease, Suscpetibility To, 9 | 16.52 | Y |
| MYBPC3 | Myosin Binding Protein C3 | 16.48 | N |
| POSTN | Periostin | 16.44 | N |
| COL1A1 | Collagen Type I Alpha 1 Chain | 16.41 | N |
| GJA4 | Gap Junction Protein Alpha 4 | 16.41 | N |
| SGCD | Sarcoglycan Delta | 16.4 | N |
| SERPINA3 | Serpin Family A Member 3 | 16.34 | N |
| MIR182 | MicroRNA 182 | 16.33 | N |
| KALRN | Kalirin RhoGEF Kinase | 16.3 | N |
| EGF | Epidermal Growth Factor | 16.29 | N |
| CSF3 | Colony Stimulating Factor 3 | 16.26 | N |
| HFE | Homeostatic Iron Regulator | 16.19 | N |
| SREBF1 | Sterol Regulatory Element Binding Transcription Factor 1 | 16.17 | N |
| HTRA1 | HtrA Serine Peptidase 1 | 16.14 | N |
| DCN | Decorin | 16.12 | N |
| CD163 | CD163 Molecule | 16.12 | N |
| LRP8 | LDL Receptor Related Protein 8 | 16.09 | N |
| GPX1 | Glutathione Peroxidase 1 | 16.03 | N |
| CHDS1 | Coronary Heart Disease, Susceptibility To, 1 | 16.01 | Y |
| PSEN1 | Presenilin 1 | 16 | N |
| LGALS3 | Galectin 3 | 15.95 | N |
| ABCB1 | ATP Binding Cassette Subfamily B Member 1 | 15.88 | N |
| SAA1 | Serum Amyloid A1 | 15.87 | N |
| HSPA4 | Heat Shock Protein Family A (Hsp70) Member 4 | 15.78 | N |
| MMP7 | Matrix Metallopeptidase 7 | 15.77 | N |
| NR3C2 | Nuclear Receptor Subfamily 3 Group C Member 2 | 15.73 | N |
| DNASE1 | Deoxyribonuclease 1 | 15.71 | N |
| ECE1 | Endothelin Converting Enzyme 1 | 15.69 | N |
| TFPI | Tissue Factor Pathway Inhibitor | 15.68 | N |
| PDE3A | Phosphodiesterase 3A | 15.67 | N |
| GLB1 | Galactosidase Beta 1 | 15.6 | N |
| H2AC18 | H2A Clustered Histone 18 | 15.55 | N |
| PDE4D | Phosphodiesterase 4D | 15.51 | N |
| TMPO | Thymopoietin | 15.38 | N |
| GCLC | Glutamate-Cysteine Ligase Catalytic Subunit | 15.37 | N |
| EGFR | Epidermal Growth Factor Receptor | 15.37 | N |
| NR3C1 | Nuclear Receptor Subfamily 3 Group C Member 1 | 15.34 | N |
| FGF23 | Fibroblast Growth Factor 23 | 15.33 | N |
| ROCK1 | Rho Associated Coiled-Coil Containing Protein Kinase 1 | 15.29 | N |
| TAZ | Tafazzin | 15.22 | N |
| MMP12 | Matrix Metallopeptidase 12 | 15.2 | N |
| MBL2 | Mannose Binding Lectin 2 | 15.2 | N |
| KCNQ1 | Potassium Voltage-Gated Channel Subfamily Q Member 1 | 15.19 | N |
| PTEN | Phosphatase And Tensin Homolog | 15.15 | N |
| LEMD3 | LEM Domain Containing 3 | 15.15 | N |
| KIF6 | Kinesin Family Member 6 | 15.15 | N |
| IGFBP1 | Insulin Like Growth Factor Binding Protein 1 | 15.15 | N |
| THBS1 | Thrombospondin 1 | 15.14 | N |
| PIK3CG | Phosphatidylinositol-4,5-Bisphosphate 3-Kinase Catalytic Subunit Gamma | 15.13 | N |
| MIR221 | MicroRNA 221 | 15.12 | N |
| AOC3 | Amine Oxidase Copper Containing 3 | 15.08 | N |
| PSEN2 | Presenilin 2 | 15.04 | N |
| S100B | S100 Calcium Binding Protein B | 15.03 | N |
| TTR | Transthyretin | 14.97 | N |
| ADAMTS13 | ADAM Metallopeptidase With Thrombospondin Type 1 Motif 13 | 14.95 | N |
| CCL11 | C-C Motif Chemokine Ligand 11 | 14.84 | N |
| FMR1 | FMRP Translational Regulator 1 | 14.84 | N |
| GLA | Galactosidase Alpha | 14.83 | N |
| ADRB3 | Adrenoceptor Beta 3 | 14.78 | N |
| GSR | Glutathione-Disulfide Reductase | 14.77 | N |
| CYP27A1 | Cytochrome P450 Family 27 Subfamily A Member 1 | 14.75 | N |
| SLC17A5 | Solute Carrier Family 17 Member 5 | 14.69 | N |
| BDNF | Brain Derived Neurotrophic Factor | 14.67 | N |
| SULT1A3 | Sulfotransferase Family 1A Member 3 | 14.62 | N |
| PTPN22 | Protein Tyrosine Phosphatase Non-Receptor Type 22 | 14.61 | N |
| VTN | Vitronectin | 14.59 | N |
| CPB2 | Carboxypeptidase B2 | 14.53 | N |
| BDKRB2 | Bradykinin Receptor B2 | 14.41 | N |
| MAPK8 | Mitogen-Activated Protein Kinase 8 | 14.4 | N |
| C3 | Complement C3 | 14.38 | N |
| CYBB | Cytochrome B-245 Beta Chain | 14.37 | N |
| IGFBP3 | Insulin Like Growth Factor Binding Protein 3 | 14.36 | N |
| CYCS | Cytochrome C, Somatic | 14.36 | N |
| GCG | Glucagon | 14.34 | N |
| THBS2 | Thrombospondin 2 | 14.32 | N |
| NR1H4 | Nuclear Receptor Subfamily 1 Group H Member 4 | 14.31 | N |
| DMD | Dystrophin | 14.28 | N |
| MLXIPL | MLX Interacting Protein Like | 14.25 | N |
| MIR199A1 | MicroRNA 199a-1 | 14.22 | N |
| DSP | Desmoplakin | 14.22 | N |
| MIR196A2 | MicroRNA 196a-2 | 14.21 | N |
| SERPINF2 | Serpin Family F Member 2 | 14.2 | N |
| RARRES2 | Retinoic Acid Receptor Responder 2 | 14.2 | N |
| PDGFB | Platelet Derived Growth Factor Subunit B | 14.17 | N |
| SLC8A1 | Solute Carrier Family 8 Member A1 | 14.17 | N |
| IRS1 | Insulin Receptor Substrate 1 | 14.14 | N |
| SORT1 | Sortilin 1 | 14.11 | N |
| PPIG | Peptidylprolyl Isomerase G | 14.07 | N |
| PDCD10 | Programmed Cell Death 10 | 14.05 | N |
| AVP | Arginine Vasopressin | 14.04 | N |
| KL | Klotho | 14.03 | N |
| NDE1 | NudE Neurodevelopment Protein 1 | 13.95 | N |
| ADRA2B | Adrenoceptor Alpha 2B | 13.92 | N |
| ITGB1 | Integrin Subunit Beta 1 | 13.92 | N |
| ITGB2 | Integrin Subunit Beta 2 | 13.91 | N |
| BMP4 | Bone Morphogenetic Protein 4 | 13.89 | N |
| TREX1 | Three Prime Repair Exonuclease 1 | 13.87 | N |
| GAPDH | Glyceraldehyde-3-Phosphate Dehydrogenase | 13.86 | N |
| FOXP3 | Forkhead Box P3 | 13.81 | N |
| PROCR | Protein C Receptor | 13.8 | N |
| TBX4 | T-Box Transcription Factor 4 | 13.8 | N |
| MIR143 | MicroRNA 143 | 13.76 | N |
| F9 | Coagulation Factor IX | 13.73 | N |
| PSRC1 | Proline And Serine Rich Coiled-Coil 1 | 13.68 | N |
| TXN | Thioredoxin | 13.68 | N |
| DLL4 | Delta Like Canonical Notch Ligand 4 | 13.66 | N |
| PDGFA | Platelet Derived Growth Factor Subunit A | 13.65 | N |
| MIR33B | MicroRNA 33b | 13.53 | N |
| ITGAL | Integrin Subunit Alpha L | 13.53 | N |
| IL33 | Interleukin 33 | 13.49 | N |
| TNC | Tenascin C | 13.48 | N |
| MIR223 | MicroRNA 223 | 13.46 | N |
| NOX1 | NADPH Oxidase 1 | 13.45 | N |
| SORL1 | Sortilin Related Receptor 1 | 13.44 | N |
| RYR2 | Ryanodine Receptor 2 | 13.44 | N |
| HTR3A | 5-Hydroxytryptamine Receptor 3A | 13.44 | N |
| DES | Desmin | 13.42 | N |
| VKORC1 | Vitamin K Epoxide Reductase Complex Subunit 1 | 13.42 | N |
| TPM1 | Tropomyosin 1 | 13.42 | N |
| PRKAR1A | Protein Kinase CAMP-Dependent Type I Regulatory Subunit Alpha | 13.42 | N |
| MIR125A | MicroRNA 125a | 13.41 | N |
| LTBP1 | Latent Transforming Growth Factor Beta Binding Protein 1 | 13.41 | N |
| DPP4 | Dipeptidyl Peptidase 4 | 13.39 | N |
| CACNA1D | Calcium Voltage-Gated Channel Subunit Alpha1 D | 13.39 | N |
| RBP4 | Retinol Binding Protein 4 | 13.38 | N |
| MMP13 | Matrix Metallopeptidase 13 | 13.36 | N |
| FLNA | Filamin A | 13.32 | N |
| MIR222 | MicroRNA 222 | 13.27 | N |
| ERCC6 | ERCC Excision Repair 6, Chromatin Remodeling Factor | 13.23 | N |
| MME | Membrane Metalloendopeptidase | 13.23 | N |
| F11 | Coagulation Factor XI | 13.13 | N |
| TIMP3 | TIMP Metallopeptidase Inhibitor 3 | 13.11 | N |
| KCNJ8 | Potassium Inwardly Rectifying Channel Subfamily J Member 8 | 13.1 | N |
| MTRR | 5-Methyltetrahydrofolate-Homocysteine Methyltransferase Reductase | 13.09 | N |
| DSG2 | Desmoglein 2 | 13.09 | N |
| MIR150 | MicroRNA 150 | 13.08 | N |
| GAA | Glucosidase Alpha, Acid | 13.06 | N |
| MAPK3 | Mitogen-Activated Protein Kinase 3 | 13.03 | N |
| TNNT1 | Troponin T1, Slow Skeletal Type | 13.02 | N |
| RAC1 | Rac Family Small GTPase 1 | 12.92 | N |
| SELPLG | Selectin P Ligand | 12.91 | N |
| CTF1 | Cardiotrophin 1 | 12.84 | N |
| CCT7 | Chaperonin Containing TCP1 Subunit 7 | 12.83 | N |
| MCI2 | Myocardial Infarction, Susceptiblity To, 2 | 12.83 | N |
| NFE2L2 | Nuclear Factor, Erythroid 2 Like 2 | 12.82 | N |
| ALPL | Alkaline Phosphatase, Biomineralization Associated | 12.8 | N |
| CMA1 | Chymase 1 | 12.79 | N |
| ATM | ATM Serine/Threonine Kinase | 12.75 | N |
| CSF2 | Colony Stimulating Factor 2 | 12.72 | N |
| C4A | Complement C4A (Rodgers Blood Group) | 12.67 | N |
| SRC | SRC Proto-Oncogene, Non-Receptor Tyrosine Kinase | 12.65 | N |
| TERT | Telomerase Reverse Transcriptase | 12.62 | N |
| TP53COR1 | Tumor Protein P53 Pathway Corepressor 1 | 12.61 | N |
| SMAD2 | SMAD Family Member 2 | 12.56 | N |
| CX3CL1 | C-X3-C Motif Chemokine Ligand 1 | 12.55 | N |
| CSRP3 | Cysteine And Glycine Rich Protein 3 | 12.54 | N |
| KCNH2 | Potassium Voltage-Gated Channel Subfamily H Member 2 | 12.52 | N |
| CNR1 | Cannabinoid Receptor 1 | 12.51 | N |
| LAMA2 | Laminin Subunit Alpha 2 | 12.47 | N |
| MIR423 | MicroRNA 423 | 12.45 | N |
| SPARC | Secreted Protein Acidic And Cysteine Rich | 12.44 | N |
| ANGPT2 | Angiopoietin 2 | 12.44 | N |
| PLN | Phospholamban | 12.44 | N |
| EEF1A2 | Eukaryotic Translation Elongation Factor 1 Alpha 2 | 12.36 | N |
| FLNC | Filamin C | 12.35 | N |
| HLA-B | Major Histocompatibility Complex, Class I, B | 12.34 | N |
| LAMP2 | Lysosomal Associated Membrane Protein 2 | 12.33 | N |
| SOCS1 | Suppressor Of Cytokine Signaling 1 | 12.33 | N |
| TUG1 | Taurine Up-Regulated 1 | 12.31 | N |
| CCL3 | C-C Motif Chemokine Ligand 3 | 12.28 | N |
| IL6R | Interleukin 6 Receptor | 12.28 | N |
| AAT1 | Aortic Aneurysm, Familial Thoracic 1 | 12.27 | N |
| MEF2C | Myocyte Enhancer Factor 2C | 12.24 | N |
| MC4R | Melanocortin 4 Receptor | 12.24 | N |
| BAZ1B | Bromodomain Adjacent To Zinc Finger Domain 1B | 12.22 | N |
| CACNA1C | Calcium Voltage-Gated Channel Subunit Alpha1 C | 12.2 | N |
| NPR1 | Natriuretic Peptide Receptor 1 | 12.18 | N |
| SOAT2 | Sterol O-Acyltransferase 2 | 12.16 | N |
| APLNR | Apelin Receptor | 12.14 | N |
| CSF1 | Colony Stimulating Factor 1 | 12.09 | N |
| NPR2 | Natriuretic Peptide Receptor 2 | 12.05 | N |
| GAS5 | Growth Arrest Specific 5 | 12.05 | N |
| LPXN | Leupaxin | 12.04 | N |
| CKM | Creatine Kinase, M-Type | 11.97 | N |
| MYD88 | MYD88 Innate Immune Signal Transduction Adaptor | 11.88 | N |
| FLT1 | Fms Related Receptor Tyrosine Kinase 1 | 11.86 | N |
| NR2F2 | Nuclear Receptor Subfamily 2 Group F Member 2 | 11.82 | N |
| PPARGC1A | PPARG Coactivator 1 Alpha | 11.8 | N |
| SCNN1A | Sodium Channel Epithelial 1 Subunit Alpha | 11.78 | N |
| THBS4 | Thrombospondin 4 | 11.75 | N |
| MAPT | Microtubule Associated Protein Tau | 11.74 | N |
| HS3ST1 | Heparan Sulfate-Glucosamine 3-Sulfotransferase 1 | 11.73 | N |
| MMRN1 | Multimerin 1 | 11.7 | N |
| PTH | Parathyroid Hormone | 11.62 | N |
| NPC2 | NPC Intracellular Cholesterol Transporter 2 | 11.62 | N |
| PITX2 | Paired Like Homeodomain 2 | 11.62 | N |
| HSPA8 | Heat Shock Protein Family A (Hsp70) Member 8 | 11.62 | N |
| CASR | Calcium Sensing Receptor | 11.61 | N |
| BMP2 | Bone Morphogenetic Protein 2 | 11.61 | N |
| LIMK1 | LIM Domain Kinase 1 | 11.61 | N |
| POMC | Proopiomelanocortin | 11.58 | N |
| COX5A | Cytochrome C Oxidase Subunit 5A | 11.52 | N |
| CASP1 | Caspase 1 | 11.49 | N |
| SP1 | Sp1 Transcription Factor | 11.46 | N |
| SLC2A4 | Solute Carrier Family 2 Member 4 | 11.44 | N |
| BRCA1 | BRCA1 DNA Repair Associated | 11.39 | N |
| FBN2 | Fibrillin 2 | 11.39 | N |
| NOTCH2 | Notch Receptor 2 | 11.38 | N |
| IGFBP7 | Insulin Like Growth Factor Binding Protein 7 | 11.35 | N |
| PRDM16 | PR/SET Domain 16 | 11.34 | N |
| CTNNB1 | Catenin Beta 1 | 11.33 | N |
| HOXC-AS1 | HOXC Cluster Antisense RNA 1 | 11.31 | N |
| PTPRC | Protein Tyrosine Phosphatase Receptor Type C | 11.3 | N |
| HRAS | HRas Proto-Oncogene, GTPase | 11.28 | N |
| MIR6886 | MicroRNA 6886 | 11.26 | N |
| IBSP | Integrin Binding Sialoprotein | 11.25 | N |
| SMAD6 | SMAD Family Member 6 | 11.24 | N |
| PLCG1 | Phospholipase C Gamma 1 | 11.23 | N |
| ABCG4 | ATP Binding Cassette Subfamily G Member 4 | 11.21 | N |
| AR | Androgen Receptor | 11.19 | N |
| MMP8 | Matrix Metallopeptidase 8 | 11.17 | N |
| ANKH | ANKH Inorganic Pyrophosphate Transport Regulator | 11.15 | N |
| HSP90AA1 | Heat Shock Protein 90 Alpha Family Class A Member 1 | 11.15 | N |
| TNFSF11 | TNF Superfamily Member 11 | 11.11 | N |
| PRKAG2 | Protein Kinase AMP-Activated Non-Catalytic Subunit Gamma 2 | 11.09 | N |
| GRK2 | G Protein-Coupled Receptor Kinase 2 | 11.04 | N |
| FKRP | Fukutin Related Protein | 11.01 | N |
| PPCS | Phosphopantothenoylcysteine Synthetase | 10.99 | N |
| PDCD1 | Programmed Cell Death 1 | 10.99 | N |
| SERPINF1 | Serpin Family F Member 1 | 10.98 | N |
| CTSL | Cathepsin L | 10.95 | N |
| IL1R1 | Interleukin 1 Receptor Type 1 | 10.93 | N |
| RBM20 | RNA Binding Motif Protein 20 | 10.91 | N |
| CKB | Creatine Kinase B | 10.88 | N |
| TRIB1 | Tribbles Pseudokinase 1 | 10.85 | N |
| TEK | TEK Receptor Tyrosine Kinase | 10.83 | N |
| KCNJ11 | Potassium Inwardly Rectifying Channel Subfamily J Member 11 | 10.83 | N |
| TET2 | Tet Methylcytosine Dioxygenase 2 | 10.83 | N |
| LBP | Lipopolysaccharide Binding Protein | 10.8 | N |
| BMPR1A | Bone Morphogenetic Protein Receptor Type 1A | 10.79 | N |
| ADCY10 | Adenylate Cyclase 10 | 10.75 | N |
| PALLD | Palladin, Cytoskeletal Associated Protein | 10.73 | N |
| AAT2 | Aortic Aneurysm, Familial Thoracic 2 | 10.72 | N |
| CREB1 | CAMP Responsive Element Binding Protein 1 | 10.65 | N |
| MIR214 | MicroRNA 214 | 10.65 | N |
| SST | Somatostatin | 10.64 | N |
| CP | Ceruloplasmin | 10.63 | N |
| UCP1 | Uncoupling Protein 1 | 10.6 | N |
| BCL2L1 | BCL2 Like 1 | 10.59 | N |
| CD59 | CD59 Molecule (CD59 Blood Group) | 10.56 | N |
| PPARD | Peroxisome Proliferator Activated Receptor Delta | 10.54 | N |
| GSTM1 | Glutathione S-Transferase Mu 1 | 10.52 | N |
| PLA2G2A | Phospholipase A2 Group IIA | 10.52 | N |
| FKTN | Fukutin | 10.51 | N |
| ERCC1 | ERCC Excision Repair 1, Endonuclease Non-Catalytic Subunit | 10.51 | N |
| ACTN2 | Actinin Alpha 2 | 10.5 | N |
| ENSG00000247287 | | 10.48 | N |
| LTA4H | Leukotriene A4 Hydrolase | 10.43 | N |
| PAOD1 | Peripheral Arterial Occlusive Disease 1 | 10.43 | Y |
| IL1A | Interleukin 1 Alpha | 10.41 | N |
| HULC | Hepatocellular Carcinoma Up-Regulated Long Non-Coding RNA | 10.38 | N |
| SCAP | SREBF Chaperone | 10.38 | N |
| NFIA-AS1 | NFIA Antisense RNA 1 | 10.37 | N |
| CYP3A5 | Cytochrome P450 Family 3 Subfamily A Member 5 | 10.34 | N |
| HAND2 | Heart And Neural Crest Derivatives Expressed 2 | 10.34 | N |
| CCR3 | C-C Motif Chemokine Receptor 3 | 10.31 | N |
| AKT2 | AKT Serine/Threonine Kinase 2 | 10.29 | N |
| SMTN | Smoothelin | 10.29 | N |
| MIR320A | MicroRNA 320a | 10.26 | N |
| NEXN | Nexilin F-Actin Binding Protein | 10.25 | N |
| MYLIP | Myosin Regulatory Light Chain Interacting Protein | 10.24 | N |
| VIP | Vasoactive Intestinal Peptide | 10.23 | N |
| TLR3 | Toll Like Receptor 3 | 10.22 | N |
| RAD51 | RAD51 Recombinase | 10.19 | N |
| ADRA2C | Adrenoceptor Alpha 2C | 10.16 | N |
| ADAMTS7 | ADAM Metallopeptidase With Thrombospondin Type 1 Motif 7 | 10.16 | N |
| TGFBR3 | Transforming Growth Factor Beta Receptor 3 | 10.14 | N |
| HELLS | Helicase, Lymphoid Specific | 10.09 | N |
| PROM1 | Prominin 1 | 10.07 | N |
| SGCB | Sarcoglycan Beta | 10.05 | N |
| TBXA2R | Thromboxane A2 Receptor | 10.02 | N |
| MIF | Macrophage Migration Inhibitory Factor | 10.01 | N |
| APOC1 | Apolipoprotein C1 | 9.99 | N |
| MIR30A | MicroRNA 30a | 9.98 | N |
| S100A12 | S100 Calcium Binding Protein A12 | 9.93 | N |
| GSTT1 | Glutathione S-Transferase Theta 1 | 9.89 | N |
| DNAH8 | Dynein Axonemal Heavy Chain 8 | 9.88 | N |
| ADRA1D | Adrenoceptor Alpha 1D | 9.84 | N |
| IGF2-AS | IGF2 Antisense RNA | 9.83 | N |
| ACAN | Aggrecan | 9.82 | N |
| BRD4 | Bromodomain Containing 4 | 9.81 | N |
| SRF | Serum Response Factor | 9.8 | N |
| MT-ND1 | Mitochondrially Encoded NADH:Ubiquinone Oxidoreductase Core Subunit 1 | 9.8 | N |
| EDN3 | Endothelin 3 | 9.77 | N |
| RHOD | Ras Homolog Family Member D | 9.72 | N |
| CYP17A1 | Cytochrome P450 Family 17 Subfamily A Member 1 | 9.72 | N |
| ABCC8 | ATP Binding Cassette Subfamily C Member 8 | 9.71 | N |
| SERPINB2 | Serpin Family B Member 2 | 9.69 | N |
| PRL | Prolactin | 9.69 | N |
| FGF4 | Fibroblast Growth Factor 4 | 9.68 | N |
| IL12A | Interleukin 12A | 9.68 | N |
| BAG3 | BAG Cochaperone 3 | 9.66 | N |
| BHMT | Betaine--Homocysteine S-Methyltransferase | 9.66 | N |
| NPC1L1 | NPC1 Like Intracellular Cholesterol Transporter 1 | 9.64 | N |
| LEPR | Leptin Receptor | 9.61 | N |
| PRODH | Proline Dehydrogenase 1 | 9.61 | N |
| PDGFRA | Platelet Derived Growth Factor Receptor Alpha | 9.61 | N |
| DDX58 | DExD/H-Box Helicase 58 | 9.58 | N |
| MIR486-1 | MicroRNA 486-1 | 9.57 | N |
| CHI3L1 | Chitinase 3 Like 1 | 9.57 | N |
| RYR1 | Ryanodine Receptor 1 | 9.47 | N |
| MIR296 | MicroRNA 296 | 9.47 | N |
| OGN | Osteoglycin | 9.46 | N |
| JUN | Jun Proto-Oncogene, AP-1 Transcription Factor Subunit | 9.44 | N |
| P2RX1 | Purinergic Receptor P2X 1 | 9.42 | N |
| CIITA | Class II Major Histocompatibility Complex Transactivator | 9.41 | N |
| EMD | Emerin | 9.35 | N |
| NBN | Nibrin | 9.35 | N |
| ABCA4 | ATP Binding Cassette Subfamily A Member 4 | 9.3 | N |
| S100A1 | S100 Calcium Binding Protein A1 | 9.3 | N |
| CYP3A4 | Cytochrome P450 Family 3 Subfamily A Member 4 | 9.3 | N |
| KCNE2 | Potassium Voltage-Gated Channel Subfamily E Regulatory Subunit 2 | 9.28 | N |
| NFKB1 | Nuclear Factor Kappa B Subunit 1 | 9.27 | N |
| CXCR1 | C-X-C Motif Chemokine Receptor 1 | 9.26 | N |
| ABCC1 | ATP Binding Cassette Subfamily C Member 1 | 9.26 | N |
| NLRP3 | NLR Family Pyrin Domain Containing 3 | 9.24 | N |
| PRKDC | Protein Kinase, DNA-Activated, Catalytic Subunit | 9.23 | N |
| ADPRH | ADP-Ribosylarginine Hydrolase | 9.23 | N |
| LBR | Lamin B Receptor | 9.22 | N |
| JCAD | Junctional Cadherin 5 Associated | 9.21 | Y |
| IGF2R | Insulin Like Growth Factor 2 Receptor | 9.21 | N |
| GC | GC Vitamin D Binding Protein | 9.19 | N |
| THPO | Thrombopoietin | 9.18 | N |
| DYNC2LI1 | Dynein Cytoplasmic 2 Light Intermediate Chain 1 | 9.16 | N |
| BCAR1 | BCAR1 Scaffold Protein, Cas Family Member | 9.15 | N |
| DCAF8 | DDB1 And CUL4 Associated Factor 8 | 9.15 | N |
| TNFRSF10C | TNF Receptor Superfamily Member 10c | 9.13 | N |
| INSR | Insulin Receptor | 9.12 | N |
| TIMP4 | TIMP Metallopeptidase Inhibitor 4 | 9.12 | N |
| MLX | MAX Dimerization Protein MLX | 9.11 | N |
| MIR133A1 | MicroRNA 133a-1 | 9.1 | N |
| GFAP | Glial Fibrillary Acidic Protein | 9.1 | N |
| CASP8 | Caspase 8 | 9.09 | N |
| SIRT6 | Sirtuin 6 | 9.03 | N |
| CORIN | Corin, Serine Peptidase | 9.02 | N |
| NF2 | Neurofibromin 2 | 9.02 | N |
| H2AX | H2A.X Variant Histone | 8.98 | N |
| RBPJ | Recombination Signal Binding Protein For Immunoglobulin Kappa J Region | 8.95 | N |
| GGT2 | Gamma-Glutamyltransferase 2 | 8.95 | N |
| CHKA | Choline Kinase Alpha | 8.9 | N |
| VDR | Vitamin D Receptor | 8.9 | N |
| HDAC9 | Histone Deacetylase 9 | 8.9 | N |
| LIN9 | Lin-9 DREAM MuvB Core Complex Component | 8.86 | N |
| BCHE | Butyrylcholinesterase | 8.86 | N |
| EPOR | Erythropoietin Receptor | 8.85 | N |
| TNXB | Tenascin XB | 8.84 | N |
| MYC | MYC Proto-Oncogene, BHLH Transcription Factor | 8.84 | N |
| COL4A2 | Collagen Type IV Alpha 2 Chain | 8.84 | N |
| MIR30E | MicroRNA 30e | 8.81 | N |
| GAST | Gastrin | 8.81 | N |
| ATP2A2 | ATPase Sarcoplasmic/Endoplasmic Reticulum Ca2+ Transporting 2 | 8.8 | N |
| TCAP | Titin-Cap | 8.8 | N |
| COQ10A | Coenzyme Q10A | 8.79 | N |
| SPEG | Striated Muscle Enriched Protein Kinase | 8.76 | N |
| SERPINA1 | Serpin Family A Member 1 | 8.76 | N |
| SLCO1B1 | Solute Carrier Organic Anion Transporter Family Member 1B1 | 8.75 | N |
| MYH9 | Myosin Heavy Chain 9 | 8.73 | N |
| ACTA2-AS1 | ACTA2 Antisense RNA 1 | 8.73 | N |
| ARF1 | ADP Ribosylation Factor 1 | 8.73 | N |
| LDLR-AS1 | LDLR-AS1 | 8.72 | N |
| NR4A1 | Nuclear Receptor Subfamily 4 Group A Member 1 | 8.71 | N |
| NSD1 | Nuclear Receptor Binding SET Domain Protein 1 | 8.7 | N |
| IFIH1 | Interferon Induced With Helicase C Domain 1 | 8.66 | N |
| BGLAP | Bone Gamma-Carboxyglutamate Protein | 8.65 | N |
| DAB2 | DAB Adaptor Protein 2 | 8.65 | N |
| CHIT1 | Chitinase 1 | 8.65 | N |
| CXCL10 | C-X-C Motif Chemokine Ligand 10 | 8.65 | N |
| COL18A1 | Collagen Type XVIII Alpha 1 Chain | 8.63 | N |
| WT1 | WT1 Transcription Factor | 8.61 | N |
| PPP1R12A | Protein Phosphatase 1 Regulatory Subunit 12A | 8.6 | N |
| MTOR | Mechanistic Target Of Rapamycin Kinase | 8.6 | N |
| SERPING1 | Serpin Family G Member 1 | 8.59 | N |
| TLR8 | Toll Like Receptor 8 | 8.59 | N |
| PRKCE | Protein Kinase C Epsilon | 8.59 | N |
| HLA-DQB1 | Major Histocompatibility Complex, Class II, DQ Beta 1 | 8.59 | N |
| ABL1 | ABL Proto-Oncogene 1, Non-Receptor Tyrosine Kinase | 8.56 | N |
| HLA-DPB1 | Major Histocompatibility Complex, Class II, DP Beta 1 | 8.56 | N |
| HEY2 | Hes Related Family BHLH Transcription Factor With YRPW Motif 2 | 8.56 | N |
| IL2RA | Interleukin 2 Receptor Subunit Alpha | 8.56 | N |
| ENO2 | Enolase 2 | 8.54 | N |
| SYNE1 | Spectrin Repeat Containing Nuclear Envelope Protein 1 | 8.54 | N |
| PDGFRB | Platelet Derived Growth Factor Receptor Beta | 8.52 | N |
| CYP2J2 | Cytochrome P450 Family 2 Subfamily J Member 2 | 8.52 | N |
| HSPA1A | Heat Shock Protein Family A (Hsp70) Member 1A | 8.51 | N |
| TCF7L2 | Transcription Factor 7 Like 2 | 8.51 | N |
| CCR1 | C-C Motif Chemokine Receptor 1 | 8.5 | N |
| HBA1 | Hemoglobin Subunit Alpha 1 | 8.5 | N |
| BRINP3 | BMP/Retinoic Acid Inducible Neural Specific 3 | 8.5 | N |
| MYL3 | Myosin Light Chain 3 | 8.5 | N |
| RTN4 | Reticulon 4 | 8.49 | N |
| PCSK2 | Proprotein Convertase Subtilisin/Kexin Type 2 | 8.48 | N |
| F12 | Coagulation Factor XII | 8.45 | N |
| CYP19A1 | Cytochrome P450 Family 19 Subfamily A Member 1 | 8.43 | N |
| APEX2 | Apurinic/Apyrimidinic Endodeoxyribonuclease 2 | 8.43 | N |
| ABCC2 | ATP Binding Cassette Subfamily C Member 2 | 8.41 | N |
| CXCR3 | C-X-C Motif Chemokine Receptor 3 | 8.41 | N |
| ADRA2A | Adrenoceptor Alpha 2A | 8.38 | N |
| FTO | FTO Alpha-Ketoglutarate Dependent Dioxygenase | 8.38 | N |
| CYP27B1 | Cytochrome P450 Family 27 Subfamily B Member 1 | 8.35 | N |
| ADAM17 | ADAM Metallopeptidase Domain 17 | 8.33 | N |
| MIR122 | MicroRNA 122 | 8.33 | N |
| ITGAV | Integrin Subunit Alpha V | 8.33 | N |
| CRAT | Carnitine O-Acetyltransferase | 8.33 | N |
| SLC2A9 | Solute Carrier Family 2 Member 9 | 8.31 | N |
| MIR30C1 | MicroRNA 30c-1 | 8.29 | N |
| FABP2 | Fatty Acid Binding Protein 2 | 8.29 | N |
| NR1H2 | Nuclear Receptor Subfamily 1 Group H Member 2 | 8.29 | N |
| LIPE | Lipase E, Hormone Sensitive Type | 8.28 | N |
| PROC | Protein C, Inactivator Of Coagulation Factors Va And VIIIa | 8.27 | N |
| FNDC5 | Fibronectin Type III Domain Containing 5 | 8.26 | N |
| ACTA1 | Actin Alpha 1, Skeletal Muscle | 8.26 | N |
| S100A9 | S100 Calcium Binding Protein A9 | 8.25 | N |
| HOTTIP | HOXA Distal Transcript Antisense RNA | 8.24 | N |
| CASP9 | Caspase 9 | 8.22 | N |
| KCNA5 | Potassium Voltage-Gated Channel Subfamily A Member 5 | 8.22 | N |
| LIPF | Lipase F, Gastric Type | 8.22 | N |
| SYNE2 | Spectrin Repeat Containing Nuclear Envelope Protein 2 | 8.22 | N |
| CYP1A1 | Cytochrome P450 Family 1 Subfamily A Member 1 | 8.21 | N |
| GDF2 | Growth Differentiation Factor 2 | 8.2 | N |
| PLAU | Plasminogen Activator, Urokinase | 8.17 | N |
| STAP1 | Signal Transducing Adaptor Family Member 1 | 8.16 | N |
| CDH13 | Cadherin 13 | 8.15 | N |
| APOA1-AS | APOA1 Antisense RNA | 8.13 | N |
| CIDEC | Cell Death Inducing DFFA Like Effector C | 8.13 | N |
| KCNJ2 | Potassium Inwardly Rectifying Channel Subfamily J Member 2 | 8.12 | N |
| OBSCN | Obscurin, Cytoskeletal Calmodulin And Titin-Interacting RhoGEF | 8.11 | N |
| ABCA12 | ATP Binding Cassette Subfamily A Member 12 | 8.1 | N |
| MACROD1 | Mono-ADP Ribosylhydrolase 1 | 8.09 | N |
| ATP1A2 | ATPase Na+/K+ Transporting Subunit Alpha 2 | 8.09 | N |
| MACROD2 | Mono-ADP Ribosylhydrolase 2 | 8.08 | N |
| VCAN | Versican | 8.08 | N |
| SSNA1 | SS Nuclear Autoantigen 1 | 8.08 | N |
| LOC110973015 | NOS3 5' Regulatory Region | 8.07 | N |
| CDKN1B | Cyclin Dependent Kinase Inhibitor 1B | 8.07 | N |
| ACTG1 | Actin Gamma 1 | 8.03 | N |
| IL12B | Interleukin 12B | 8.02 | N |
| DNAH5 | Dynein Axonemal Heavy Chain 5 | 8.01 | N |
| IL13 | Interleukin 13 | 8 | N |
| PROS1 | Protein S | 7.99 | N |
| ACTB | Actin Beta | 7.99 | N |
| CD93 | CD93 Molecule | 7.98 | N |
| FDXR | Ferredoxin Reductase | 7.95 | N |
| AGRP | Agouti Related Neuropeptide | 7.95 | N |
| MICU1 | Mitochondrial Calcium Uptake 1 | 7.95 | N |
| ZBTB8OS | Zinc Finger And BTB Domain Containing 8 Opposite Strand | 7.95 | N |
| OARD1 | O-Acyl-ADP-Ribose Deacylase 1 | 7.95 | N |
| CHURC1 | Churchill Domain Containing 1 | 7.95 | N |
| PARPBP | PARP1 Binding Protein | 7.95 | N |
| ADPRS | ADP-Ribosylserine Hydrolase | 7.95 | N |
| TKT | Transketolase | 7.95 | N |
| UCP2 | Uncoupling Protein 2 | 7.94 | N |
| ADORA1 | Adenosine A1 Receptor | 7.93 | N |
| CFLAR | CASP8 And FADD Like Apoptosis Regulator | 7.89 | N |
| FASLG | Fas Ligand | 7.89 | N |
| MALAT1 | Metastasis Associated Lung Adenocarcinoma Transcript 1 | 7.87 | N |
| M6PR | Mannose-6-Phosphate Receptor, Cation Dependent | 7.86 | N |
| THY1 | Thy-1 Cell Surface Antigen | 7.84 | N |
| KCNMA1 | Potassium Calcium-Activated Channel Subfamily M Alpha 1 | 7.83 | N |
| ANK2 | Ankyrin 2 | 7.81 | N |
| HBG2 | Hemoglobin Subunit Gamma 2 | 7.81 | N |
| CXCL16 | C-X-C Motif Chemokine Ligand 16 | 7.79 | N |
| TNFRSF11A | TNF Receptor Superfamily Member 11a | 7.79 | N |
| LIPI | Lipase I | 7.78 | N |
| TF | Transferrin | 7.77 | N |
| INHBA | Inhibin Subunit Beta A | 7.74 | N |
| PNLIP | Pancreatic Lipase | 7.74 | N |
| PITPNA | Phosphatidylinositol Transfer Protein Alpha | 7.73 | N |
| HBB | Hemoglobin Subunit Beta | 7.72 | N |
| RUNX2 | RUNX Family Transcription Factor 2 | 7.72 | N |
| CD86 | CD86 Molecule | 7.72 | N |
| PARP1 | Poly(ADP-Ribose) Polymerase 1 | 7.71 | N |
| WFDC21P | WAP Four-Disulfide Core Domain 21, Pseudogene | 7.69 | N |
| SMARCAL1 | SWI/SNF Related, Matrix Associated, Actin Dependent Regulator Of Chromatin, Subfamily A Like 1 | 7.69 | N |
| CD44 | CD44 Molecule (Indian Blood Group) | 7.67 | N |
| S100A8 | S100 Calcium Binding Protein A8 | 7.66 | N |
| MIR142 | MicroRNA 142 | 7.65 | N |
| ARSH | Arylsulfatase Family Member H | 7.65 | N |
| PYGB | Glycogen Phosphorylase B | 7.65 | N |
| CHGA | Chromogranin A | 7.64 | N |
| SLC9A1 | Solute Carrier Family 9 Member A1 | 7.64 | N |
| PTGIR | Prostaglandin I2 Receptor | 7.63 | N |
| JUP | Junction Plakoglobin | 7.62 | N |
| CRYAA | Crystallin Alpha A | 7.61 | N |
| NRG1 | Neuregulin 1 | 7.61 | N |
| MIR23A | MicroRNA 23a | 7.61 | N |
| CH25H | Cholesterol 25-Hydroxylase | 7.61 | N |
| PLAUR | Plasminogen Activator, Urokinase Receptor | 7.6 | N |
| TNFSF12 | TNF Superfamily Member 12 | 7.6 | N |
| ALOX15 | Arachidonate 15-Lipoxygenase | 7.58 | N |
| MFGE8 | Milk Fat Globule-EGF Factor 8 Protein | 7.58 | N |
| APOL1 | Apolipoprotein L1 | 7.57 | N |
| CCDC92 | Coiled-Coil Domain Containing 92 | 7.56 | N |
| lnc-KDM5D-4 |  | 7.56 | N |
| MTHFD1L | Methylenetetrahydrofolate Dehydrogenase (NADP+ Dependent) 1 Like | 7.56 | N |
| SCN9A | Sodium Voltage-Gated Channel Alpha Subunit 9 | 7.56 | N |
| SMARCA4 | SWI/SNF Related, Matrix Associated, Actin Dependent Regulator Of Chromatin, Subfamily A, Member 4 | 7.53 | N |
| ITIH4 | Inter-Alpha-Trypsin Inhibitor Heavy Chain 4 | 7.52 | N |
| FGG | Fibrinogen Gamma Chain | 7.52 | N |
| ESR2 | Estrogen Receptor 2 | 7.52 | N |
| KRIT1 | KRIT1 Ankyrin Repeat Containing | 7.47 | N |
| LIPJ | Lipase Family Member J | 7.47 | N |
| CACNA2D1 | Calcium Voltage-Gated Channel Auxiliary Subunit Alpha2delta 1 | 7.45 | N |
| CA3 | Carbonic Anhydrase 3 | 7.44 | N |
| ADRA1B | Adrenoceptor Alpha 1B | 7.42 | N |
| C5 | Complement C5 | 7.42 | N |
| ACHE | Acetylcholinesterase (Cartwright Blood Group) | 7.41 | N |
| MEFV | MEFV Innate Immuity Regulator, Pyrin | 7.4 | N |
| DSC2 | Desmocollin 2 | 7.4 | N |
| VHL | Von Hippel-Lindau Tumor Suppressor | 7.39 | N |
| NGF | Nerve Growth Factor | 7.39 | N |
| CFI | Complement Factor I | 7.37 | N |
| CDKN1C | Cyclin Dependent Kinase Inhibitor 1C | 7.35 | N |
| HADHA | Hydroxyacyl-CoA Dehydrogenase Trifunctional Multienzyme Complex Subunit Alpha | 7.34 | N |
| NRP1 | Neuropilin 1 | 7.34 | N |
| FCGR3B | Fc Fragment Of IgG Receptor IIIb | 7.33 | N |
| CCL4 | C-C Motif Chemokine Ligand 4 | 7.32 | N |
| ADCY3 | Adenylate Cyclase 3 | 7.32 | N |
| SUN2 | Sad1 And UNC84 Domain Containing 2 | 7.31 | N |
| MIR9-1 | MicroRNA 9-1 | 7.31 | N |
| VLDLR | Very Low Density Lipoprotein Receptor | 7.31 | N |
| MYDGF | Myeloid Derived Growth Factor | 7.31 | N |
| TMEM43 | Transmembrane Protein 43 | 7.3 | N |
| MT-LIPCAR | Mitochondrially Encoded Long Non-Coding Cardiac Associated RNA | 7.29 | N |
| CTH | Cystathionine Gamma-Lyase | 7.28 | N |
| SAA4 | Serum Amyloid A4, Constitutive | 7.25 | N |
| TPM2 | Tropomyosin 2 | 7.23 | N |
| CS | Citrate Synthase | 7.22 | N |
| IGF2 | Insulin Like Growth Factor 2 | 7.21 | N |
| MT-ATP6 | Mitochondrially Encoded ATP Synthase Membrane Subunit 6 | 7.21 | N |
| TRPC3 | Transient Receptor Potential Cation Channel Subfamily C Member 3 | 7.21 | N |
| ITLN1 | Intelectin 1 | 7.19 | N |
| GATAD1 | GATA Zinc Finger Domain Containing 1 | 7.19 | N |
| MIR133B | MicroRNA 133b | 7.18 | N |
| FOXC2 | Forkhead Box C2 | 7.18 | N |
| KLK15 | Kallikrein Related Peptidase 15 | 7.17 | N |
| CXCL1 | C-X-C Motif Chemokine Ligand 1 | 7.15 | N |
| ADIPOR1 | Adiponectin Receptor 1 | 7.14 | N |
| KCNE1 | Potassium Voltage-Gated Channel Subfamily E Regulatory Subunit 1 | 7.14 | N |
| PCNA | Proliferating Cell Nuclear Antigen | 7.13 | N |
| ANGPTL4 | Angiopoietin Like 4 | 7.12 | N |
| HNF1A | HNF1 Homeobox A | 7.12 | N |
| PIK3CA | Phosphatidylinositol-4,5-Bisphosphate 3-Kinase Catalytic Subunit Alpha | 7.12 | N |
| ANK3 | Ankyrin 3 | 7.12 | N |
| BCL2 | BCL2 Apoptosis Regulator | 7.12 | N |
| CCM2 | CCM2 Scaffold Protein | 7.07 | N |
| IL23R | Interleukin 23 Receptor | 7.07 | N |
| PLIN1 | Perilipin 1 | 7.07 | N |
| TGM2 | Transglutaminase 2 | 7.06 | N |
| CXCL9 | C-X-C Motif Chemokine Ligand 9 | 7.04 | N |
| NR1H3 | Nuclear Receptor Subfamily 1 Group H Member 3 | 7.02 | N |
| LMNB1 | Lamin B1 | 7.02 | N |
| ADORA2A | Adenosine A2a Receptor | 7.01 | N |
| HSD11B1 | Hydroxysteroid 11-Beta Dehydrogenase 1 | 7.01 | N |
| CES3 | Carboxylesterase 3 | 6.98 | N |
| PNPLA5 | Patatin Like Phospholipase Domain Containing 5 | 6.98 | N |
| LIPK | Lipase Family Member K | 6.98 | N |
| CES4A | Carboxylesterase 4A | 6.98 | N |
| CES5A | Carboxylesterase 5A | 6.98 | N |
| VSTM4 | V-Set And Transmembrane Domain Containing 4 | 6.98 | N |
| LIPM | Lipase Family Member M | 6.98 | N |
| MIR208B | MicroRNA 208b | 6.97 | N |
| ICMT | Isoprenylcysteine Carboxyl Methyltransferase | 6.97 | N |
| RFC1 | Replication Factor C Subunit 1 | 6.97 | N |
| BBS2 | Bardet-Biedl Syndrome 2 | 6.96 | N |
| SOCS3 | Suppressor Of Cytokine Signaling 3 | 6.96 | N |
| MT-TL1 | Mitochondrially Encoded TRNA-Leu (UUA/G) 1 | 6.96 | N |
| LAMA4 | Laminin Subunit Alpha 4 | 6.95 | N |
| ADAMTSL1 | ADAMTS Like 1 | 6.93 | N |
| ITGA1 | Integrin Subunit Alpha 1 | 6.93 | N |
| ILK | Integrin Linked Kinase | 6.9 | N |
| EGR1 | Early Growth Response 1 | 6.89 | N |
| HABP2 | Hyaluronan Binding Protein 2 | 6.87 | N |
| NEBL | Nebulette | 6.86 | N |
| EDN2 | Endothelin 2 | 6.86 | N |
| MMP14 | Matrix Metallopeptidase 14 | 6.86 | N |
| BSG | Basigin (Ok Blood Group) | 6.85 | N |
| ABO | ABO, Alpha 1-3-N-Acetylgalactosaminyltransferase And Alpha 1-3-Galactosyltransferase | 6.85 | N |
| MIR505 | MicroRNA 505 | 6.85 | N |
| GSTP1 | Glutathione S-Transferase Pi 1 | 6.85 | N |
| TNFAIP3 | TNF Alpha Induced Protein 3 | 6.83 | N |
| ABRAXAS2 | Abraxas 2, BRISC Complex Subunit | 6.83 | N |
| LMOD1 | Leiomodin 1 | 6.81 | N |
| DNMT1 | DNA Methyltransferase 1 | 6.8 | N |
| LEPQTL1 | Leptin, Serum Levels Of | 6.79 | N |
| NOMO3 | NODAL Modulator 3 | 6.79 | N |
| KIF2C | Kinesin Family Member 2C | 6.78 | N |
| ITGA4 | Integrin Subunit Alpha 4 | 6.78 | N |
| FKBP1A | FKBP Prolyl Isomerase 1A | 6.77 | N |
| MYL2 | Myosin Light Chain 2 | 6.77 | N |
| MYPN | Myopalladin | 6.76 | N |
| CASQ2 | Calsequestrin 2 | 6.76 | N |
| FGFR1 | Fibroblast Growth Factor Receptor 1 | 6.75 | N |
| PAH | Phenylalanine Hydroxylase | 6.73 | N |
| AHCY | Adenosylhomocysteinase | 6.72 | N |
| MYL4 | Myosin Light Chain 4 | 6.72 | N |
| TYR | Tyrosinase | 6.71 | N |
| LAMA1 | Laminin Subunit Alpha 1 | 6.71 | N |
| AEBP1 | AE Binding Protein 1 | 6.71 | N |
| CELSR2 | Cadherin EGF LAG Seven-Pass G-Type Receptor 2 | 6.7 | N |
| GCK | Glucokinase | 6.69 | N |
| PKP2 | Plakophilin 2 | 6.68 | N |
| TH | Tyrosine Hydroxylase | 6.68 | N |
| LOC106560211 | APOB 5' Regulatory Region | 6.68 | N |
| TLR9 | Toll Like Receptor 9 | 6.67 | N |
| NOMO1 | NODAL Modulator 1 | 6.67 | N |
| TP53BP1 | Tumor Protein P53 Binding Protein 1 | 6.66 | N |
| GRP | Gastrin Releasing Peptide | 6.66 | N |
| HSPG2 | Heparan Sulfate Proteoglycan 2 | 6.66 | N |
| C1QTNF1 | C1q And TNF Related 1 | 6.65 | N |
| SERPINA12 | Serpin Family A Member 12 | 6.65 | N |
| ETS1 | ETS Proto-Oncogene 1, Transcription Factor | 6.64 | N |
| XPA | XPA, DNA Damage Recognition And Repair Factor | 6.63 | N |
| PLAG1 | PLAG1 Zinc Finger | 6.63 | N |
| NOMO2 | NODAL Modulator 2 | 6.63 | N |
| LOC102723692 | Uncharacterized LOC102723692 | 6.63 | N |
| ABCG2 | ATP Binding Cassette Subfamily G Member 2 (Junior Blood Group) | 6.62 | N |
| RNLS | Renalase, FAD Dependent Amine Oxidase | 6.61 | N |
| HMCN1 | Hemicentin 1 | 6.61 | N |
| MCTP2 | Multiple C2 And Transmembrane Domain Containing 2 | 6.61 | N |
| TXNRD2 | Thioredoxin Reductase 2 | 6.61 | N |
| UBA7 | Ubiquitin Like Modifier Activating Enzyme 7 | 6.61 | N |
| EGID-106632268 | APOB 3' Scaffold/Matrix Attachment Region (S/MAR) | 6.58 | N |
| SUN1 | Sad1 And UNC84 Domain Containing 1 | 6.58 | N |
| LMNB2 | Lamin B2 | 6.56 | N |
| MKKS | McKusick-Kaufman Syndrome | 6.56 | N |
| APOM | Apolipoprotein M | 6.55 | N |
| IL1RAPL2 | Interleukin 1 Receptor Accessory Protein Like 2 | 6.54 | N |
| EMC10 | ER Membrane Protein Complex Subunit 10 | 6.53 | N |
| FADS1 | Fatty Acid Desaturase 1 | 6.53 | N |
| ANTXR1 | ANTXR Cell Adhesion Molecule 1 | 6.51 | N |
| TAT | Tyrosine Aminotransferase | 6.49 | N |
| FGF21 | Fibroblast Growth Factor 21 | 6.47 | N |
| IGFBP2 | Insulin Like Growth Factor Binding Protein 2 | 6.47 | N |
| RELA | RELA Proto-Oncogene, NF-KB Subunit | 6.47 | N |
| ERCC8 | ERCC Excision Repair 8, CSA Ubiquitin Ligase Complex Subunit | 6.46 | N |
| CTNNA1 | Catenin Alpha 1 | 6.45 | N |
| ESM1 | Endothelial Cell Specific Molecule 1 | 6.44 | N |
| STAR | Steroidogenic Acute Regulatory Protein | 6.43 | N |
| ANKRD2 | Ankyrin Repeat Domain 2 | 6.43 | N |
| CDKN1A | Cyclin Dependent Kinase Inhibitor 1A | 6.42 | N |
| SLC20A2 | Solute Carrier Family 20 Member 2 | 6.42 | N |
| IFNA1 | Interferon Alpha 1 | 6.4 | N |
| SDHB | Succinate Dehydrogenase Complex Iron Sulfur Subunit B | 6.4 | N |
| SIRT2 | Sirtuin 2 | 6.39 | N |
| IL17F | Interleukin 17F | 6.38 | N |
| USF1 | Upstream Transcription Factor 1 | 6.38 | N |
| BCAR3 | BCAR3 Adaptor Protein, NSP Family Member | 6.37 | N |
| PRKCB | Protein Kinase C Beta | 6.36 | N |
| TPM3 | Tropomyosin 3 | 6.35 | N |
| TCF21 | Transcription Factor 21 | 6.34 | N |
| GCKR | Glucokinase Regulator | 6.34 | N |
| GAS6 | Growth Arrest Specific 6 | 6.34 | N |
| CDH2 | Cadherin 2 | 6.34 | N |
| FOXC1 | Forkhead Box C1 | 6.33 | N |
| DTNA | Dystrobrevin Alpha | 6.32 | N |
| H3-2 | H3.2 Histone (Putative) | 6.31 | N |
| RCE1 | Ras Converting CAAX Endopeptidase 1 | 6.3 | N |
| CHAT | Choline O-Acetyltransferase | 6.29 | N |
| MAPK10 | Mitogen-Activated Protein Kinase 10 | 6.29 | N |
| MTPN | Myotrophin | 6.28 | N |
| SPG7 | SPG7 Matrix AAA Peptidase Subunit, Paraplegin | 6.28 | N |
| DDAH2 | Dimethylarginine Dimethylaminohydrolase 2 | 6.28 | N |
| HTR1A | 5-Hydroxytryptamine Receptor 1A | 6.25 | N |
| TWIST2 | Twist Family BHLH Transcription Factor 2 | 6.23 | N |
| AKAP9 | A-Kinase Anchoring Protein 9 | 6.23 | N |
| MCAM | Melanoma Cell Adhesion Molecule | 6.23 | N |
| IRF5 | Interferon Regulatory Factor 5 | 6.21 | N |
| SCN4B | Sodium Voltage-Gated Channel Beta Subunit 4 | 6.21 | N |
| CCL21 | C-C Motif Chemokine Ligand 21 | 6.21 | N |
| PRKCA | Protein Kinase C Alpha | 6.2 | N |
| EFEMP1 | EGF Containing Fibulin Extracellular Matrix Protein 1 | 6.2 | N |
| LTF | Lactotransferrin | 6.19 | N |
| MMP17 | Matrix Metallopeptidase 17 | 6.19 | N |
| TSPO | Translocator Protein | 6.19 | N |
| NPR3 | Natriuretic Peptide Receptor 3 | 6.18 | N |
| CD79A | CD79a Molecule | 6.18 | N |
| CLEC4A | C-Type Lectin Domain Family 4 Member A | 6.16 | N |
| NAT10 | N-Acetyltransferase 10 | 6.16 | N |
| MIR29A | MicroRNA 29a | 6.16 | N |
| SYNE3 | Spectrin Repeat Containing Nuclear Envelope Family Member 3 | 6.15 | N |
| FAH | Fumarylacetoacetate Hydrolase | 6.15 | N |
| VEGFC | Vascular Endothelial Growth Factor C | 6.15 | N |
| FCGR3A | Fc Fragment Of IgG Receptor IIIa | 6.15 | N |
| CXCR2 | C-X-C Motif Chemokine Receptor 2 | 6.14 | N |
| MAP2 | Microtubule Associated Protein 2 | 6.14 | N |
| STAT5B | Signal Transducer And Activator Of Transcription 5B | 6.14 | N |
| HIF1A-AS1 | HIF1A Antisense RNA 1 | 6.13 | N |
| RAN | RAN, Member RAS Oncogene Family | 6.12 | N |
| PRPS1 | Phosphoribosyl Pyrophosphate Synthetase 1 | 6.12 | N |
| SUV39H1 | Suppressor Of Variegation 3-9 Homolog 1 | 6.12 | N |
| SYNE4 | Spectrin Repeat Containing Nuclear Envelope Family Member 4 | 6.12 | N |
| LEXM | Lymphocyte Expansion Molecule | 6.12 | N |
| CPT2 | Carnitine Palmitoyltransferase 2 | 6.11 | N |
| G6PD | Glucose-6-Phosphate Dehydrogenase | 6.11 | N |
| PGM1 | Phosphoglucomutase 1 | 6.11 | N |
| HAMP | Hepcidin Antimicrobial Peptide | 6.11 | N |
| TNFSF10 | TNF Superfamily Member 10 | 6.11 | N |
| GLMN | Glomulin, FKBP Associated Protein | 6.1 | N |
| MEG3 | Maternally Expressed 3 | 6.08 | N |
| ANKRD23 | Ankyrin Repeat Domain 23 | 6.08 | N |
| CES1 | Carboxylesterase 1 | 6.06 | N |
| DOCK6 | Dedicator Of Cytokinesis 6 | 6.05 | N |
| MSTN | Myostatin | 6.03 | N |
| IGF1R | Insulin Like Growth Factor 1 Receptor | 6.03 | N |
| GALNT3 | Polypeptide N-Acetylgalactosaminyltransferase 3 | 6 | N |
| HCN4 | Hyperpolarization Activated Cyclic Nucleotide Gated Potassium Channel 4 | 6 | N |
| IL5 | Interleukin 5 | 5.99 | N |
| RB1 | RB Transcriptional Corepressor 1 | 5.99 | N |
| KLF4 | Kruppel Like Factor 4 | 5.98 | N |
| CRH | Corticotropin Releasing Hormone | 5.98 | N |
| EGFL7 | EGF Like Domain Multiple 7 | 5.97 | N |
| MAP2K1 | Mitogen-Activated Protein Kinase Kinase 1 | 5.95 | N |
| IL37 | Interleukin 37 | 5.95 | N |
| TFAM | Transcription Factor A, Mitochondrial | 5.94 | N |
| ACTN1 | Actinin Alpha 1 | 5.94 | N |
| IL3 | Interleukin 3 | 5.92 | N |
| SNTA1 | Syntrophin Alpha 1 | 5.92 | N |
| VIM | Vimentin | 5.91 | N |
| ARL15 | ADP Ribosylation Factor Like GTPase 15 | 5.91 | N |
| HSPB1 | Heat Shock Protein Family B (Small) Member 1 | 5.91 | N |
| BMPER | BMP Binding Endothelial Regulator | 5.9 | N |
| SOST | Sclerostin | 5.87 | N |
| CCL19 | C-C Motif Chemokine Ligand 19 | 5.86 | N |
| NOD2 | Nucleotide Binding Oligomerization Domain Containing 2 | 5.85 | N |
| TPO | Thyroid Peroxidase | 5.85 | N |
| TLR7 | Toll Like Receptor 7 | 5.85 | N |
| FKBP1B | FKBP Prolyl Isomerase 1B | 5.85 | N |
| HAVCR1 | Hepatitis A Virus Cellular Receptor 1 | 5.84 | N |
| VASP | Vasodilator Stimulated Phosphoprotein | 5.84 | N |
| IL15 | Interleukin 15 | 5.83 | N |
| ZPR1 | ZPR1 Zinc Finger | 5.82 | N |
| EPS15 | Epidermal Growth Factor Receptor Pathway Substrate 15 | 5.81 | N |
| F2RL1 | F2R Like Trypsin Receptor 1 | 5.8 | N |
| HLA-A | Major Histocompatibility Complex, Class I, A | 5.8 | N |
| TMEM59 | Transmembrane Protein 59 | 5.79 | N |
| TRERF1 | Transcriptional Regulating Factor 1 | 5.79 | N |
| BCAR4 | Breast Cancer Anti-Estrogen Resistance 4 | 5.79 | N |
| CHD7 | Chromodomain Helicase DNA Binding Protein 7 | 5.75 | N |
| PKD1 | Polycystin 1, Transient Receptor Potential Channel Interacting | 5.74 | N |
| OXT | Oxytocin/Neurophysin I Prepropeptide | 5.74 | N |
| MAOA | Monoamine Oxidase A | 5.73 | N |
| DDAH1 | Dimethylarginine Dimethylaminohydrolase 1 | 5.72 | N |
| CPS1 | Carbamoyl-Phosphate Synthase 1 | 5.72 | N |
| NFATC4 | Nuclear Factor Of Activated T Cells 4 | 5.7 | N |
| FOXO3 | Forkhead Box O3 | 5.69 | N |
| CXADR | CXADR Ig-Like Cell Adhesion Molecule | 5.68 | N |
| ZFHX3 | Zinc Finger Homeobox 3 | 5.68 | N |
| FDFT1 | Farnesyl-Diphosphate Farnesyltransferase 1 | 5.68 | N |
| NOTCH4 | Notch Receptor 4 | 5.67 | N |
| SMDT1 | Single-Pass Membrane Protein With Aspartate Rich Tail 1 | 5.67 | N |
| DMP1 | Dentin Matrix Acidic Phosphoprotein 1 | 5.67 | N |
| FADS2 | Fatty Acid Desaturase 2 | 5.66 | N |
| BAX | BCL2 Associated X, Apoptosis Regulator | 5.64 | N |
| KLK1 | Kallikrein 1 | 5.63 | N |
| KLF2 | Kruppel Like Factor 2 | 5.62 | N |
| HEYL | Hes Related Family BHLH Transcription Factor With YRPW Motif Like | 5.61 | N |
| STAT5A | Signal Transducer And Activator Of Transcription 5A | 5.6 | N |
| PLA2G5 | Phospholipase A2 Group V | 5.6 | N |
| IRAK1 | Interleukin 1 Receptor Associated Kinase 1 | 5.59 | N |
| FHL2 | Four And A Half LIM Domains 2 | 5.57 | N |
| STAG3 | Stromal Antigen 3 | 5.57 | N |
| TNNI1 | Troponin I1, Slow Skeletal Type | 5.57 | N |
| ERBB2 | Erb-B2 Receptor Tyrosine Kinase 2 | 5.56 | N |
| CTSK | Cathepsin K | 5.55 | N |
| TLR1 | Toll Like Receptor 1 | 5.55 | N |
| PLA2G10 | Phospholipase A2 Group X | 5.55 | N |
| MIR483 | MicroRNA 483 | 5.54 | N |
| TARID | TCF21 Antisense RNA Inducing Promoter Demethylation | 5.54 | N |
| CCR7 | C-C Motif Chemokine Receptor 7 | 5.54 | N |
| MRAP | Melanocortin 2 Receptor Accessory Protein | 5.53 | N |
| SDHA | Succinate Dehydrogenase Complex Flavoprotein Subunit A | 5.52 | N |
| CYP1B1 | Cytochrome P450 Family 1 Subfamily B Member 1 | 5.52 | N |
| MIR195 | MicroRNA 195 | 5.52 | N |
| CACNB2 | Calcium Voltage-Gated Channel Auxiliary Subunit Beta 2 | 5.51 | N |
| BLOC1S1 | Biogenesis Of Lysosomal Organelles Complex 1 Subunit 1 | 5.51 | N |
| NCF2 | Neutrophil Cytosolic Factor 2 | 5.5 | N |
| ITGA5 | Integrin Subunit Alpha 5 | 5.5 | N |
| IL1RL1 | Interleukin 1 Receptor Like 1 | 5.49 | N |
| FMOD | Fibromodulin | 5.45 | N |
| MIA3 | MIA SH3 Domain ER Export Factor 3 | 5.45 | N |
| ACADVL | Acyl-CoA Dehydrogenase Very Long Chain | 5.43 | N |
| MYOZ2 | Myozenin 2 | 5.4 | N |
| GSK3B | Glycogen Synthase Kinase 3 Beta | 5.39 | N |
| RGS2 | Regulator Of G Protein Signaling 2 | 5.39 | N |
| POU2F3 | POU Class 2 Homeobox 3 | 5.38 | N |
| CMKLR1 | Chemerin Chemokine-Like Receptor 1 | 5.37 | N |
| COL8A1 | Collagen Type VIII Alpha 1 Chain | 5.36 | N |
| GRN | Granulin Precursor | 5.36 | N |
| PPIA | Peptidylprolyl Isomerase A | 5.36 | N |
| CYP2C8 | Cytochrome P450 Family 2 Subfamily C Member 8 | 5.36 | N |
| SGCA | Sarcoglycan Alpha | 5.36 | N |
| SLC12A5-AS1 | SLC12A5 And MMP9 Antisense RNA 1 | 5.36 | N |
| LOC100506472 | Uncharacterized LOC100506472 | 5.36 | N |
| AMPD1 | Adenosine Monophosphate Deaminase 1 | 5.36 | N |
| DOLK | Dolichol Kinase | 5.34 | N |
| SLC25A4 | Solute Carrier Family 25 Member 4 | 5.34 | N |
| MHRT | Myosin Heavy Chain Associated RNA Transcript | 5.33 | N |
| CRYAB | Crystallin Alpha B | 5.33 | N |
| GPER1 | G Protein-Coupled Estrogen Receptor 1 | 5.31 | N |
| TNNI3K | TNNI3 Interacting Kinase | 5.3 | N |
| MAPK7 | Mitogen-Activated Protein Kinase 7 | 5.3 | N |
| LIMS2 | LIM Zinc Finger Domain Containing 2 | 5.3 | N |
| MIR590 | MicroRNA 590 | 5.3 | N |
| ATP2B1 | ATPase Plasma Membrane Ca2+ Transporting 1 | 5.29 | N |
| SAMHD1 | SAM And HD Domain Containing Deoxynucleoside Triphosphate Triphosphohydrolase 1 | 5.29 | N |
| C4B | Complement C4B (Chido Blood Group) | 5.28 | N |
| PDGFC | Platelet Derived Growth Factor C | 5.27 | N |
| PNPLA2 | Patatin Like Phospholipase Domain Containing 2 | 5.26 | N |
| PHOSPHO1 | Phosphoethanolamine/Phosphocholine Phosphatase 1 | 5.26 | N |
| UGT1A1 | UDP Glucuronosyltransferase Family 1 Member A1 | 5.25 | N |
| MT-ND2 | Mitochondrially Encoded NADH:Ubiquinone Oxidoreductase Core Subunit 2 | 5.25 | N |
| CCL13 | C-C Motif Chemokine Ligand 13 | 5.25 | N |
| FURIN | Furin, Paired Basic Amino Acid Cleaving Enzyme | 5.23 | N |
| SLC10A2 | Solute Carrier Family 10 Member 2 | 5.22 | N |
| DNAH10 | Dynein Axonemal Heavy Chain 10 | 5.21 | N |
| ADCY5 | Adenylate Cyclase 5 | 5.2 | N |
| PTGDS | Prostaglandin D2 Synthase | 5.2 | N |
| SLC22A5 | Solute Carrier Family 22 Member 5 | 5.2 | N |
| ADAMTS4 | ADAM Metallopeptidase With Thrombospondin Type 1 Motif 4 | 5.18 | N |
| CCND1 | Cyclin D1 | 5.18 | N |
| SRFBP1 | Serum Response Factor Binding Protein 1 | 5.18 | N |
| SMARCA1 | SWI/SNF Related, Matrix Associated, Actin Dependent Regulator Of Chromatin, Subfamily A, Member 1 | 5.17 | N |
| RPS27A | Ribosomal Protein S27a | 5.17 | N |
| TRPV2 | Transient Receptor Potential Cation Channel Subfamily V Member 2 | 5.15 | N |
| NFKBIA | NFKB Inhibitor Alpha | 5.14 | N |
| ITGA7 | Integrin Subunit Alpha 7 | 5.14 | N |
| VEGFB | Vascular Endothelial Growth Factor B | 5.12 | N |
| ADA | Adenosine Deaminase | 5.11 | N |
| PHEX | Phosphate Regulating Endopeptidase Homolog X-Linked | 5.11 | N |
| CTNNA3 | Catenin Alpha 3 | 5.1 | N |
| PTPN1 | Protein Tyrosine Phosphatase Non-Receptor Type 1 | 5.09 | N |
| EYA4 | EYA Transcriptional Coactivator And Phosphatase 4 | 5.08 | N |
| MIR204 | MicroRNA 204 | 5.08 | N |
| ARG1 | Arginase 1 | 5.07 | N |
| RPA1 | Replication Protein A1 | 5.07 | N |
| PDE4A | Phosphodiesterase 4A | 5.06 | N |
| CYP4A11 | Cytochrome P450 Family 4 Subfamily A Member 11 | 5.06 | N |
| TNNC1 | Troponin C1, Slow Skeletal And Cardiac Type | 5.06 | N |
| AIF1 | Allograft Inflammatory Factor 1 | 5.06 | N |
| CHRM2 | Cholinergic Receptor Muscarinic 2 | 5.04 | N |
| SMAD1 | SMAD Family Member 1 | 5.03 | N |
| ADAMTS1 | ADAM Metallopeptidase With Thrombospondin Type 1 Motif 1 | 5.02 | N |
| P2RY2 | Purinergic Receptor P2Y2 | 5.01 | N |
| IL2RB | Interleukin 2 Receptor Subunit Beta | 5.01 | N |
| CEL | Carboxyl Ester Lipase | 5 | N |
| CFL1 | Cofilin 1 | 5 | N |
| ADAR | Adenosine Deaminase RNA Specific | 5 | N |
| ALOX12 | Arachidonate 12-Lipoxygenase, 12S Type | 5 | N |
| SCD | Stearoyl-CoA Desaturase | 4.99 | N |
| MT-ND6 | Mitochondrially Encoded NADH:Ubiquinone Oxidoreductase Core Subunit 6 | 4.98 | N |
| DRD2 | Dopamine Receptor D2 | 4.98 | N |
| CTSD | Cathepsin D | 4.98 | N |
| JPH2 | Junctophilin 2 | 4.97 | N |
| CR1 | Complement C3b/C4b Receptor 1 (Knops Blood Group) | 4.96 | N |
| APOC4 | Apolipoprotein C4 | 4.95 | N |
| KCNIP2 | Potassium Voltage-Gated Channel Interacting Protein 2 | 4.95 | N |
| ADTRP | Androgen Dependent TFPI Regulating Protein | 4.95 | N |
| ADIPOR2 | Adiponectin Receptor 2 | 4.94 | N |
| SP2 | Sp2 Transcription Factor | 4.93 | N |
| COL1A2 | Collagen Type I Alpha 2 Chain | 4.92 | N |
| ST3GAL4 | ST3 Beta-Galactoside Alpha-2,3-Sialyltransferase 4 | 4.91 | N |
| TLN1 | Talin 1 | 4.91 | N |
| ACP5 | Acid Phosphatase 5, Tartrate Resistant | 4.91 | N |
| S1PR2 | Sphingosine-1-Phosphate Receptor 2 | 4.9 | N |
| NCF1 | Neutrophil Cytosolic Factor 1 | 4.9 | N |
| CCL18 | C-C Motif Chemokine Ligand 18 | 4.89 | N |
| PRKAA1 | Protein Kinase AMP-Activated Catalytic Subunit Alpha 1 | 4.88 | N |
| ENTPD1 | Ectonucleoside Triphosphate Diphosphohydrolase 1 | 4.87 | N |
| SOX9 | SRY-Box Transcription Factor 9 | 4.86 | N |
| CAV2 | Caveolin 2 | 4.86 | N |
| IAPP | Islet Amyloid Polypeptide | 4.85 | N |
| CNN1 | Calponin 1 | 4.83 | N |
| OSM | Oncostatin M | 4.83 | N |
| HBEGF | Heparin Binding EGF Like Growth Factor | 4.82 | N |
| ATP2A3 | ATPase Sarcoplasmic/Endoplasmic Reticulum Ca2+ Transporting 3 | 4.81 | N |
| XK | X-Linked Kx Blood Group | 4.81 | N |
| DNAJC19 | DnaJ Heat Shock Protein Family (Hsp40) Member C19 | 4.81 | N |
| XRCC1 | X-Ray Repair Cross Complementing 1 | 4.81 | N |
| LDB2 | LIM Domain Binding 2 | 4.8 | N |
| SCNN1B | Sodium Channel Epithelial 1 Subunit Beta | 4.8 | N |
| KCND3 | Potassium Voltage-Gated Channel Subfamily D Member 3 | 4.8 | N |
| C5AR1 | Complement C5a Receptor 1 | 4.8 | N |
| FHL5 | Four And A Half LIM Domains 5 | 4.8 | N |
| FBXO32 | F-Box Protein 32 | 4.78 | N |
| MIR27B | MicroRNA 27b | 4.78 | N |
| HSPB7 | Heat Shock Protein Family B (Small) Member 7 | 4.78 | N |
| MIR92A1 | MicroRNA 92a-1 | 4.77 | N |
| GPD1L | Glycerol-3-Phosphate Dehydrogenase 1 Like | 4.77 | N |
| DAG1 | Dystroglycan 1 | 4.76 | N |
| SFTPD | Surfactant Protein D | 4.76 | N |
| LGR6 | Leucine Rich Repeat Containing G Protein-Coupled Receptor 6 | 4.76 | N |
| MAOB | Monoamine Oxidase B | 4.75 | N |
| CTSB | Cathepsin B | 4.74 | N |
| MIR19B1 | MicroRNA 19b-1 | 4.74 | N |
| MIR124-1 | MicroRNA 124-1 | 4.74 | N |
| IKBKG | Inhibitor Of Nuclear Factor Kappa B Kinase Regulatory Subunit Gamma | 4.74 | N |
| CYP2E1 | Cytochrome P450 Family 2 Subfamily E Member 1 | 4.74 | N |
| SRSF2 | Serine And Arginine Rich Splicing Factor 2 | 4.73 | N |
| CD68 | CD68 Molecule | 4.73 | N |
| PLA2G6 | Phospholipase A2 Group VI | 4.73 | N |
| MYH10 | Myosin Heavy Chain 10 | 4.72 | N |
| PDGFD | Platelet Derived Growth Factor D | 4.72 | N |
| ANG | Angiogenin | 4.72 | N |
| GCH1 | GTP Cyclohydrolase 1 | 4.72 | N |
| PINK1 | PTEN Induced Kinase 1 | 4.72 | N |
| PROZ | Protein Z, Vitamin K Dependent Plasma Glycoprotein | 4.71 | N |
| CTSG | Cathepsin G | 4.71 | N |
| POLB | DNA Polymerase Beta | 4.7 | N |
| LRP5 | LDL Receptor Related Protein 5 | 4.7 | N |
| MIR494 | MicroRNA 494 | 4.69 | N |
| NQO1 | NAD(P)H Quinone Dehydrogenase 1 | 4.69 | N |
| TRDN | Triadin | 4.68 | N |
| RECK | Reversion Inducing Cysteine Rich Protein With Kazal Motifs | 4.67 | N |
| TRPV1 | Transient Receptor Potential Cation Channel Subfamily V Member 1 | 4.66 | N |
| TRPV4 | Transient Receptor Potential Cation Channel Subfamily V Member 4 | 4.66 | N |
| COCH | Cochlin | 4.66 | N |
| SLC27A6 | Solute Carrier Family 27 Member 6 | 4.65 | N |
| GH1 | Growth Hormone 1 | 4.65 | N |
| DKK1 | Dickkopf WNT Signaling Pathway Inhibitor 1 | 4.64 | N |
| GLP1R | Glucagon Like Peptide 1 Receptor | 4.63 | N |
| P2RY1 | Purinergic Receptor P2Y1 | 4.63 | N |
| SCNN1G | Sodium Channel Epithelial 1 Subunit Gamma | 4.62 | N |
| NEK8 | NIMA Related Kinase 8 | 4.61 | N |
| PLEC | Plectin | 4.61 | N |
| ADM2 | Adrenomedullin 2 | 4.61 | N |
| FEN1 | Flap Structure-Specific Endonuclease 1 | 4.61 | N |
| MASP2 | Mannan Binding Lectin Serine Peptidase 2 | 4.6 | N |
| CTSS | Cathepsin S | 4.6 | N |
| LCMT2 | Leucine Carboxyl Methyltransferase 2 | 4.6 | N |
| GALNT2 | Polypeptide N-Acetylgalactosaminyltransferase 2 | 4.6 | N |
| PSMD4 | Proteasome 26S Subunit, Non-ATPase 4 | 4.6 | N |
| ICOSLG | Inducible T Cell Costimulator Ligand | 4.59 | N |
| PPIF | Peptidylprolyl Isomerase F | 4.59 | N |
| ARID3A | AT-Rich Interaction Domain 3A | 4.59 | N |
| VCP | Valosin Containing Protein | 4.58 | N |
| IL16 | Interleukin 16 | 4.57 | N |
| PPP1R3A | Protein Phosphatase 1 Regulatory Subunit 3A | 4.57 | N |
| ENO1 | Enolase 1 | 4.57 | N |
| LYVE1 | Lymphatic Vessel Endothelial Hyaluronan Receptor 1 | 4.56 | N |
| BRAP | BRCA1 Associated Protein | 4.56 | N |
| ANXA6 | Annexin A6 | 4.55 | N |
| IL27 | Interleukin 27 | 4.55 | N |
| KLKB1 | Kallikrein B1 | 4.55 | N |
| CAPN10 | Calpain 10 | 4.55 | N |
| MRE11 | MRE11 Homolog, Double Strand Break Repair Nuclease | 4.54 | N |
| JMJD1C | Jumonji Domain Containing 1C | 4.54 | N |
| SARDH | Sarcosine Dehydrogenase | 4.54 | N |
| MYOT | Myotilin | 4.54 | N |
| TLR5 | Toll Like Receptor 5 | 4.53 | N |
| SRA1 | Steroid Receptor RNA Activator 1 | 4.53 | N |
| KCNJ12 | Potassium Inwardly Rectifying Channel Subfamily J Member 12 | 4.53 | N |
| IRS2 | Insulin Receptor Substrate 2 | 4.52 | N |
| TNXA | Tenascin XA (Pseudogene) | 4.52 | N |
| STAG3L4 | Stromal Antigen 3-Like 4 (Pseudogene) | 4.52 | N |
| TOR2A | Torsin Family 2 Member A | 4.51 | N |
| TGIF1 | TGFB Induced Factor Homeobox 1 | 4.51 | N |
| ZEB2 | Zinc Finger E-Box Binding Homeobox 2 | 4.51 | N |
| PRKAA2 | Protein Kinase AMP-Activated Catalytic Subunit Alpha 2 | 4.51 | N |
| GDNF | Glial Cell Derived Neurotrophic Factor | 4.5 | N |
| FGFR2 | Fibroblast Growth Factor Receptor 2 | 4.5 | N |
| KCNMB1 | Potassium Calcium-Activated Channel Subfamily M Regulatory Beta Subunit 1 | 4.5 | N |
| RRAGC | Ras Related GTP Binding C | 4.49 | N |
| NR4A3 | Nuclear Receptor Subfamily 4 Group A Member 3 | 4.49 | N |
| AKR1B1 | Aldo-Keto Reductase Family 1 Member B | 4.48 | N |
| ITGB6 | Integrin Subunit Beta 6 | 4.47 | N |
| AHR | Aryl Hydrocarbon Receptor | 4.47 | N |
| EIF2B2 | Eukaryotic Translation Initiation Factor 2B Subunit Beta | 4.45 | N |
| CRYGC | Crystallin Gamma C | 4.45 | N |
| FSTL1 | Follistatin Like 1 | 4.45 | N |
| TERF2 | Telomeric Repeat Binding Factor 2 | 4.44 | N |
| ACKR3 | Atypical Chemokine Receptor 3 | 4.43 | N |
| KRT8 | Keratin 8 | 4.43 | N |
| CXCL5 | C-X-C Motif Chemokine Ligand 5 | 4.42 | N |
| ATP6AP2 | ATPase H+ Transporting Accessory Protein 2 | 4.42 | N |
| HMOX2 | Heme Oxygenase 2 | 4.42 | N |
| ARMS2 | Age-Related Maculopathy Susceptibility 2 | 4.42 | N |
| MS4A2 | Membrane Spanning 4-Domains A2 | 4.42 | N |
| ANXA2 | Annexin A2 | 4.41 | N |
| CD80 | CD80 Molecule | 4.41 | N |
| NFS1 | NFS1 Cysteine Desulfurase | 4.4 | N |
| C8A | Complement C8 Alpha Chain | 4.4 | N |
| PARG | Poly(ADP-Ribose) Glycohydrolase | 4.4 | N |
| L3MBTL3 | L3MBTL Histone Methyl-Lysine Binding Protein 3 | 4.4 | N |
| SPAG17 | Sperm Associated Antigen 17 | 4.4 | N |
| PDCL2 | Phosducin Like 2 | 4.4 | N |
| C1orf210 | Chromosome 1 Open Reading Frame 210 | 4.4 | N |
| ARL13B | ADP Ribosylation Factor Like GTPase 13B | 4.4 | N |
| DNMT3A | DNA Methyltransferase 3 Alpha | 4.4 | N |
| XRCC5 | X-Ray Repair Cross Complementing 5 | 4.4 | N |
| POU5F1 | POU Class 5 Homeobox 1 | 4.4 | N |
| GNAQ | G Protein Subunit Alpha Q | 4.39 | N |
| MFAP4 | Microfibril Associated Protein 4 | 4.39 | N |
| MIR130A | MicroRNA 130a | 4.38 | N |
| CAVIN4 | Caveolae Associated Protein 4 | 4.36 | N |
| FGF1 | Fibroblast Growth Factor 1 | 4.36 | N |
| CDKN3 | Cyclin Dependent Kinase Inhibitor 3 | 4.36 | N |
| HEY1 | Hes Related Family BHLH Transcription Factor With YRPW Motif 1 | 4.35 | N |
| MVK | Mevalonate Kinase | 4.35 | N |
| ROCK2 | Rho Associated Coiled-Coil Containing Protein Kinase 2 | 4.34 | N |
| FLT4 | Fms Related Receptor Tyrosine Kinase 4 | 4.34 | N |
| ADH1B | Alcohol Dehydrogenase 1B (Class I), Beta Polypeptide | 4.33 | N |
| LPAL2 | Lipoprotein(A) Like 2, Pseudogene | 4.32 | N |
| CD28 | CD28 Molecule | 4.32 | N |
| GP1BB | Glycoprotein Ib Platelet Subunit Beta | 4.32 | N |
| GATA2 | GATA Binding Protein 2 | 4.32 | N |
| GREM1 | Gremlin 1, DAN Family BMP Antagonist | 4.31 | N |
| NLRP1 | NLR Family Pyrin Domain Containing 1 | 4.31 | N |
| DOCK7 | Dedicator Of Cytokinesis 7 | 4.3 | N |
| TNFRSF4 | TNF Receptor Superfamily Member 4 | 4.3 | N |
| IFNB1 | Interferon Beta 1 | 4.29 | N |
| C5AR2 | Complement Component 5a Receptor 2 | 4.28 | N |
| ACP1 | Acid Phosphatase 1 | 4.28 | N |
| HOPX | HOP Homeobox | 4.27 | N |
| NEU1 | Neuraminidase 1 | 4.27 | N |
| PGR | Progesterone Receptor | 4.27 | N |
| ADAM10 | ADAM Metallopeptidase Domain 10 | 4.27 | N |
| NR1I2 | Nuclear Receptor Subfamily 1 Group I Member 2 | 4.26 | N |
| TRPC1 | Transient Receptor Potential Cation Channel Subfamily C Member 1 | 4.25 | N |
| HNF4A | Hepatocyte Nuclear Factor 4 Alpha | 4.25 | N |
| CDH23 | Cadherin Related 23 | 4.24 | N |
| CACNA1S | Calcium Voltage-Gated Channel Subunit Alpha1 S | 4.24 | N |
| KRT18 | Keratin 18 | 4.24 | N |
| CCL1 | C-C Motif Chemokine Ligand 1 | 4.24 | N |
| S1PR3 | Sphingosine-1-Phosphate Receptor 3 | 4.21 | N |
| PCSK1 | Proprotein Convertase Subtilisin/Kexin Type 1 | 4.21 | N |
| MIR24-1 | MicroRNA 24-1 | 4.19 | N |
| SLC27A1 | Solute Carrier Family 27 Member 1 | 4.19 | N |
| PPOX | Protoporphyrinogen Oxidase | 4.19 | N |
| RGS5 | Regulator Of G Protein Signaling 5 | 4.18 | N |
| GPR182 | G Protein-Coupled Receptor 182 | 4.17 | N |
| PEAR1 | Platelet Endothelial Aggregation Receptor 1 | 4.17 | N |
| RMRP | RNA Component Of Mitochondrial RNA Processing Endoribonuclease | 4.17 | N |
| MIR20A | MicroRNA 20a | 4.17 | N |
| MTAP | Methylthioadenosine Phosphorylase | 4.16 | N |
| HRC | Histidine Rich Calcium Binding Protein | 4.16 | N |
| LMO7 | LIM Domain 7 | 4.15 | N |
| LIMS1 | LIM Zinc Finger Domain Containing 1 | 4.14 | N |
| MIR144 | MicroRNA 144 | 4.14 | N |
| SQSTM1 | Sequestosome 1 | 4.14 | N |
| GZMB | Granzyme B | 4.13 | N |
| RXRA | Retinoid X Receptor Alpha | 4.13 | N |
| PTAFR | Platelet Activating Factor Receptor | 4.12 | N |
| PIK3CB | Phosphatidylinositol-4,5-Bisphosphate 3-Kinase Catalytic Subunit Beta | 4.12 | N |
| AXL | AXL Receptor Tyrosine Kinase | 4.11 | N |
| MYH14 | Myosin Heavy Chain 14 | 4.1 | N |
| PDLIM3 | PDZ And LIM Domain 3 | 4.1 | N |
| SGCG | Sarcoglycan Gamma | 4.1 | N |
| GRK4 | G Protein-Coupled Receptor Kinase 4 | 4.1 | N |
| UTS2R | Urotensin 2 Receptor | 4.1 | N |
| PTGER2 | Prostaglandin E Receptor 2 | 4.1 | N |
| SCN2B | Sodium Voltage-Gated Channel Beta Subunit 2 | 4.09 | N |
| C2 | Complement C2 | 4.08 | N |
| SHC1 | SHC Adaptor Protein 1 | 4.08 | N |
| MYBPC1 | Myosin Binding Protein C1 | 4.08 | N |
| HSD11B2 | Hydroxysteroid 11-Beta Dehydrogenase 2 | 4.08 | N |
| RNASE3 | Ribonuclease A Family Member 3 | 4.07 | N |
| ADCY9 | Adenylate Cyclase 9 | 4.07 | N |
| SPON1 | Spondin 1 | 4.07 | N |
| EXOSC4 | Exosome Component 4 | 4.06 | N |
| ZNHIT3 | Zinc Finger HIT-Type Containing 3 | 4.06 | N |
| ATXN2 | Ataxin 2 | 4.06 | N |
| MIR34A | MicroRNA 34a | 4.06 | N |
| C1QTNF9 | C1q And TNF Related 9 | 4.04 | N |
| IRF1 | Interferon Regulatory Factor 1 | 4.04 | N |
| FOXO1 | Forkhead Box O1 | 4.04 | N |
| TTF2 | Transcription Termination Factor 2 | 4.04 | N |
| CXCL11 | C-X-C Motif Chemokine Ligand 11 | 4.03 | N |
| NOS1AP | Nitric Oxide Synthase 1 Adaptor Protein | 4.03 | N |
| PAX6 | Paired Box 6 | 4.03 | N |
| FBLIM1 | Filamin Binding LIM Protein 1 | 4.02 | N |
| F2RL3 | F2R Like Thrombin Or Trypsin Receptor 3 | 4.02 | N |
| BDKRB1 | Bradykinin Receptor B1 | 4.02 | N |
| ACVR1 | Activin A Receptor Type 1 | 4.02 | N |
| SYP | Synaptophysin | 4.02 | N |
| AKT3 | AKT Serine/Threonine Kinase 3 | 4.01 | N |
| MYOM3 | Myomesin 3 | 4.01 | N |
| CANT1 | Calcium Activated Nucleotidase 1 | 4.01 | N |
| IL23A | Interleukin 23 Subunit Alpha | 4 | N |
| TNNI2 | Troponin I2, Fast Skeletal Type | 3.99 | N |
| ZBTB17 | Zinc Finger And BTB Domain Containing 17 | 3.99 | N |
| MYOZ1 | Myozenin 1 | 3.99 | N |
| PLEKHM2 | Pleckstrin Homology And RUN Domain Containing M2 | 3.99 | N |
| CXCL13 | C-X-C Motif Chemokine Ligand 13 | 3.99 | N |
| CAMK2D | Calcium/Calmodulin Dependent Protein Kinase II Delta | 3.99 | N |
| NRAP | Nebulin Related Anchoring Protein | 3.99 | N |
| ITGB1BP2 | Integrin Subunit Beta 1 Binding Protein 2 | 3.99 | N |
| NIT2 | Nitrilase Family Member 2 | 3.99 | N |
| AGGF1 | Angiogenic Factor With G-Patch And FHA Domains 1 | 3.99 | N |
| ALG6 | ALG6 Alpha-1,3-Glucosyltransferase | 3.99 | N |
| TPM4 | Tropomyosin 4 | 3.99 | N |
| TNNC2 | Troponin C2, Fast Skeletal Type | 3.99 | N |
| ALPK3 | Alpha Kinase 3 | 3.99 | N |
| PTK2 | Protein Tyrosine Kinase 2 | 3.98 | N |
| HPSE | Heparanase | 3.97 | N |
| HOTAIR | HOX Transcript Antisense RNA | 3.97 | N |
| RASA1 | RAS P21 Protein Activator 1 | 3.97 | N |
| FABP12 | Fatty Acid Binding Protein 12 | 3.97 | N |
| ADCY6 | Adenylate Cyclase 6 | 3.96 | N |
| NEK9 | NIMA Related Kinase 9 | 3.96 | N |
| C9orf72 | C9orf72-SMCR8 Complex Subunit | 3.96 | N |
| PLPP3 | Phospholipid Phosphatase 3 | 3.96 | N |
| ADCYAP1 | Adenylate Cyclase Activating Polypeptide 1 | 3.95 | N |
| ACAT1 | Acetyl-CoA Acetyltransferase 1 | 3.95 | N |
| HRH1 | Histamine Receptor H1 | 3.94 | N |
| CYP4F2 | Cytochrome P450 Family 4 Subfamily F Member 2 | 3.93 | N |
| ATXN3 | Ataxin 3 | 3.92 | N |
| CACNB1 | Calcium Voltage-Gated Channel Auxiliary Subunit Beta 1 | 3.92 | N |
| HLA-C | Major Histocompatibility Complex, Class I, C | 3.91 | N |
| MTM1 | Myotubularin 1 | 3.91 | N |
| ID3 | Inhibitor Of DNA Binding 3, HLH Protein | 3.91 | N |
| MYBPC2 | Myosin Binding Protein C2 | 3.9 | N |
| PRKCH | Protein Kinase C Eta | 3.9 | N |
| NAT2 | N-Acetyltransferase 2 | 3.9 | N |
| TNFRSF10A | TNF Receptor Superfamily Member 10a | 3.89 | N |
| KISS1 | KiSS-1 Metastasis Suppressor | 3.88 | N |
| ATP2A1 | ATPase Sarcoplasmic/Endoplasmic Reticulum Ca2+ Transporting 1 | 3.88 | N |
| CFL2 | Cofilin 2 | 3.88 | N |
| SLC25A5 | Solute Carrier Family 25 Member 5 | 3.88 | N |
| CACNG1 | Calcium Voltage-Gated Channel Auxiliary Subunit Gamma 1 | 3.88 | N |
| PITX3 | Paired Like Homeodomain 3 | 3.88 | N |
| CACNG5 | Calcium Voltage-Gated Channel Auxiliary Subunit Gamma 5 | 3.88 | N |
| MYOM1 | Myomesin 1 | 3.88 | N |
| OBSL1 | Obscurin Like Cytoskeletal Adaptor 1 | 3.88 | N |
| CACNG8 | Calcium Voltage-Gated Channel Auxiliary Subunit Gamma 8 | 3.88 | N |
| CALR3 | Calreticulin 3 | 3.88 | N |
| FHOD3 | Formin Homology 2 Domain Containing 3 | 3.88 | N |
| LRRC10 | Leucine Rich Repeat Containing 10 | 3.88 | N |
| FOXD4 | Forkhead Box D4 | 3.88 | N |
| CEP85L | Centrosomal Protein 85 Like | 3.88 | N |
| ATP6V1G2-DDX39B | ATP6V1G2-DDX39B Readthrough (NMD Candidate) | 3.88 | N |
| ARSA | Arylsulfatase A | 3.88 | N |
| PLA2G2D | Phospholipase A2 Group IID | 3.87 | N |
| ADH1C | Alcohol Dehydrogenase 1C (Class I), Gamma Polypeptide | 3.87 | N |
| IDO1 | Indoleamine 2,3-Dioxygenase 1 | 3.87 | N |
| BMP7 | Bone Morphogenetic Protein 7 | 3.87 | N |
| PTGER4 | Prostaglandin E Receptor 4 | 3.86 | N |
| CD46 | CD46 Molecule | 3.86 | N |
| BPI | Bactericidal Permeability Increasing Protein | 3.86 | N |
| S1PR1 | Sphingosine-1-Phosphate Receptor 1 | 3.85 | N |
| MOCOS | Molybdenum Cofactor Sulfurase | 3.85 | N |
| INSIG2 | Insulin Induced Gene 2 | 3.84 | N |
| DAB2IP | DAB2 Interacting Protein | 3.84 | N |
| IGFBP4 | Insulin Like Growth Factor Binding Protein 4 | 3.84 | N |
| DLL3 | Delta Like Canonical Notch Ligand 3 | 3.83 | N |
| NFATC1 | Nuclear Factor Of Activated T Cells 1 | 3.83 | N |
| RREB1 | Ras Responsive Element Binding Protein 1 | 3.83 | N |
| AQP4 | Aquaporin 4 | 3.83 | N |
| PRKCI | Protein Kinase C Iota | 3.82 | N |
| ANGPTL8 | Angiopoietin Like 8 | 3.82 | N |
| FADS3 | Fatty Acid Desaturase 3 | 3.82 | N |
| CD69 | CD69 Molecule | 3.82 | N |
| CHRM1 | Cholinergic Receptor Muscarinic 1 | 3.81 | N |
| ZC3HC1 | Zinc Finger C3HC-Type Containing 1 | 3.8 | N |
| ABCB11 | ATP Binding Cassette Subfamily B Member 11 | 3.8 | N |
| FMN2 | Formin 2 | 3.8 | N |
| PRKCD | Protein Kinase C Delta | 3.79 | N |
| PEMT | Phosphatidylethanolamine N-Methyltransferase | 3.79 | N |
| MCPH1 | Microcephalin 1 | 3.78 | N |
| CPE | Carboxypeptidase E | 3.78 | N |
| IFNA2 | Interferon Alpha 2 | 3.78 | N |
| SFTPB | Surfactant Protein B | 3.77 | N |
| IL1R2 | Interleukin 1 Receptor Type 2 | 3.77 | N |
| NFKB2 | Nuclear Factor Kappa B Subunit 2 | 3.76 | N |
| SRRT | Serrate, RNA Effector Molecule | 3.76 | N |
| GSTZ1 | Glutathione S-Transferase Zeta 1 | 3.76 | N |
| MIR206 | MicroRNA 206 | 3.75 | N |
| CCL17 | C-C Motif Chemokine Ligand 17 | 3.75 | N |
| TREM1 | Triggering Receptor Expressed On Myeloid Cells 1 | 3.74 | N |
| TBXAS1 | Thromboxane A Synthase 1 | 3.74 | N |
| KLF6 | Kruppel Like Factor 6 | 3.74 | N |
| CHUK | Component Of Inhibitor Of Nuclear Factor Kappa B Kinase Complex | 3.73 | N |
| GPX3 | Glutathione Peroxidase 3 | 3.73 | N |
| TPH1 | Tryptophan Hydroxylase 1 | 3.72 | N |
| IGHE | Immunoglobulin Heavy Constant Epsilon | 3.72 | N |
| BMPR1B | Bone Morphogenetic Protein Receptor Type 1B | 3.71 | N |
| NTN1 | Netrin 1 | 3.71 | N |
| ARID5B | AT-Rich Interaction Domain 5B | 3.71 | N |
| CCR4 | C-C Motif Chemokine Receptor 4 | 3.71 | N |
| YWHAZ | Tyrosine 3-Monooxygenase/Tryptophan 5-Monooxygenase Activation Protein Zeta | 3.71 | N |
| MAPK9 | Mitogen-Activated Protein Kinase 9 | 3.7 | N |
| PARK7 | Parkinsonism Associated Deglycase | 3.7 | N |
| G6PC | Glucose-6-Phosphatase Catalytic Subunit | 3.7 | N |
| CSN1S1 | Casein Alpha S1 | 3.69 | N |
| TRAF6 | TNF Receptor Associated Factor 6 | 3.69 | N |
| POLG | DNA Polymerase Gamma, Catalytic Subunit | 3.69 | N |
| MMP10 | Matrix Metallopeptidase 10 | 3.68 | N |
| PPP1R3B | Protein Phosphatase 1 Regulatory Subunit 3B | 3.68 | N |
| KIT | KIT Proto-Oncogene, Receptor Tyrosine Kinase | 3.66 | N |
| ST2 | Suppression Of Tumorigenicity 2 | 3.66 | N |
| KISS1R | KISS1 Receptor | 3.66 | N |
| UCP3 | Uncoupling Protein 3 | 3.66 | N |
| RECQL5 | RecQ Like Helicase 5 | 3.65 | N |
| ITGA11 | Integrin Subunit Alpha 11 | 3.65 | N |
| SMO | Smoothened, Frizzled Class Receptor | 3.64 | N |
| USP8 | Ubiquitin Specific Peptidase 8 | 3.63 | N |
| DNM2 | Dynamin 2 | 3.63 | N |
| HPD | 4-Hydroxyphenylpyruvate Dioxygenase | 3.62 | N |
| KLF5 | Kruppel Like Factor 5 | 3.61 | N |
| FADD | Fas Associated Via Death Domain | 3.61 | N |
| EP300 | E1A Binding Protein P300 | 3.61 | N |
| DUSP1 | Dual Specificity Phosphatase 1 | 3.6 | N |
| ADORA2B | Adenosine A2b Receptor | 3.6 | N |
| NLRP12 | NLR Family Pyrin Domain Containing 12 | 3.59 | N |
| IKBKB | Inhibitor Of Nuclear Factor Kappa B Kinase Subunit Beta | 3.58 | N |
| WWTR1 | WW Domain Containing Transcription Regulator 1 | 3.57 | N |
| NISCH | Nischarin | 3.57 | N |
| MRTFA | Myocardin Related Transcription Factor A | 3.56 | N |
| PAFAH1B1 | Platelet Activating Factor Acetylhydrolase 1b Regulatory Subunit 1 | 3.55 | N |
| APCS | Amyloid P Component, Serum | 3.55 | N |
| PTPRN2 | Protein Tyrosine Phosphatase Receptor Type N2 | 3.55 | N |
| HERPUD1 | Homocysteine Inducible ER Protein With Ubiquitin Like Domain 1 | 3.55 | N |
| MUC16 | Mucin 16, Cell Surface Associated | 3.55 | N |
| MED18 | Mediator Complex Subunit 18 | 3.53 | N |
| TIE1 | Tyrosine Kinase With Immunoglobulin Like And EGF Like Domains 1 | 3.53 | N |
| F13B | Coagulation Factor XIII B Chain | 3.53 | N |
| SDC1 | Syndecan 1 | 3.53 | N |
| UBE2L3 | Ubiquitin Conjugating Enzyme E2 L3 | 3.53 | N |
| VEGFD | Vascular Endothelial Growth Factor D | 3.52 | N |
| TNFRSF9 | TNF Receptor Superfamily Member 9 | 3.52 | N |
| CREG1 | Cellular Repressor Of E1A Stimulated Genes 1 | 3.52 | N |
| CCN3 | Cellular Communication Network Factor 3 | 3.51 | N |
| MOCS1 | Molybdenum Cofactor Synthesis 1 | 3.51 | N |
| MFSD10 | Major Facilitator Superfamily Domain Containing 10 | 3.51 | N |
| NPB | Neuropeptide B | 3.51 | N |
| FAM223A | Family With Sequence Similarity 223 Member A | 3.51 | N |
| MMACHC | Metabolism Of Cobalamin Associated C | 3.5 | N |
| THSD7A | Thrombospondin Type 1 Domain Containing 7A | 3.5 | N |
| CD63 | CD63 Molecule | 3.5 | N |
| PXK | PX Domain Containing Serine/Threonine Kinase Like | 3.5 | N |
| ALOX15B | Arachidonate 15-Lipoxygenase Type B | 3.49 | N |
| AGXT2 | Alanine--Glyoxylate Aminotransferase 2 | 3.49 | N |
| PDYN | Prodynorphin | 3.48 | N |
| HADHB | Hydroxyacyl-CoA Dehydrogenase Trifunctional Multienzyme Complex Subunit Beta | 3.48 | N |
| NECTIN2 | Nectin Cell Adhesion Molecule 2 | 3.48 | N |
| ACP6 | Acid Phosphatase 6, Lysophosphatidic | 3.48 | N |
| PRKAB1 | Protein Kinase AMP-Activated Non-Catalytic Subunit Beta 1 | 3.46 | N |
| AMH | Anti-Mullerian Hormone | 3.46 | N |
| CACNA2D3 | Calcium Voltage-Gated Channel Auxiliary Subunit Alpha2delta 3 | 3.46 | N |
| P2RX7 | Purinergic Receptor P2X 7 | 3.46 | N |
| DBN1 | Drebrin 1 | 3.45 | N |
| PLA2G4A | Phospholipase A2 Group IVA | 3.44 | N |
| MIR10A | MicroRNA 10a | 3.44 | N |
| CDK2 | Cyclin Dependent Kinase 2 | 3.43 | N |
| SPHK1 | Sphingosine Kinase 1 | 3.43 | N |
| FBXO3 | F-Box Protein 3 | 3.43 | N |
| PDPN | Podoplanin | 3.42 | N |
| ITGAX | Integrin Subunit Alpha X | 3.41 | N |
| TERF1 | Telomeric Repeat Binding Factor 1 | 3.41 | N |
| IL32 | Interleukin 32 | 3.41 | N |
| STK11 | Serine/Threonine Kinase 11 | 3.39 | N |
| TYMS | Thymidylate Synthetase | 3.39 | N |
| ATG9B | Autophagy Related 9B | 3.38 | N |
| CSK | C-Terminal Src Kinase | 3.38 | N |
| CCNA2 | Cyclin A2 | 3.37 | N |
| TLR6 | Toll Like Receptor 6 | 3.37 | N |
| ELAVL1 | ELAV Like RNA Binding Protein 1 | 3.36 | N |
| MIR22 | MicroRNA 22 | 3.36 | N |
| DHCR7 | 7-Dehydrocholesterol Reductase | 3.35 | N |
| CUBN | Cubilin | 3.35 | N |
| CCDC3 | Coiled-Coil Domain Containing 3 | 3.35 | N |
| MDM2 | MDM2 Proto-Oncogene | 3.34 | N |
| EBF1 | EBF Transcription Factor 1 | 3.34 | N |
| TRIB3 | Tribbles Pseudokinase 3 | 3.34 | N |
| PINX1 | PIN2 (TERF1) Interacting Telomerase Inhibitor 1 | 3.33 | N |
| FPR2 | Formyl Peptide Receptor 2 | 3.33 | N |
| TRPM3 | Transient Receptor Potential Cation Channel Subfamily M Member 3 | 3.32 | N |
| SCN1A | Sodium Voltage-Gated Channel Alpha Subunit 1 | 3.32 | N |
| MIR181A1 | MicroRNA 181a-1 | 3.32 | N |
| HCRT | Hypocretin Neuropeptide Precursor | 3.32 | N |
| TLL1 | Tolloid Like 1 | 3.31 | N |
| RXRB | Retinoid X Receptor Beta | 3.31 | N |
| CACNA1H | Calcium Voltage-Gated Channel Subunit Alpha1 H | 3.31 | N |
| TSBP1 | Testis Expressed Basic Protein 1 | 3.31 | N |
| LYZ | Lysozyme | 3.31 | N |
| TRPM7 | Transient Receptor Potential Cation Channel Subfamily M Member 7 | 3.3 | N |
| GHSR | Growth Hormone Secretagogue Receptor | 3.29 | N |
| LY96 | Lymphocyte Antigen 96 | 3.29 | N |
| RPS6KB1 | Ribosomal Protein S6 Kinase B1 | 3.29 | N |
| IFI27 | Interferon Alpha Inducible Protein 27 | 3.29 | N |
| SLC2A1 | Solute Carrier Family 2 Member 1 | 3.28 | N |
| CSTB | Cystatin B | 3.28 | N |
| STAT6 | Signal Transducer And Activator Of Transcription 6 | 3.27 | N |
| MT2A | Metallothionein 2A | 3.27 | N |
| GP5 | Glycoprotein V Platelet | 3.27 | N |
| CXCR6 | C-X-C Motif Chemokine Receptor 6 | 3.27 | N |
| BMP1 | Bone Morphogenetic Protein 1 | 3.27 | N |
| PIK3R1 | Phosphoinositide-3-Kinase Regulatory Subunit 1 | 3.27 | N |
| NPNT | Nephronectin | 3.27 | N |
| NUMB | NUMB Endocytic Adaptor Protein | 3.27 | N |
| SERPINH1 | Serpin Family H Member 1 | 3.27 | N |
| CDC42 | Cell Division Cycle 42 | 3.26 | N |
| SLC30A8 | Solute Carrier Family 30 Member 8 | 3.25 | N |
| CCL7 | C-C Motif Chemokine Ligand 7 | 3.24 | N |
| ARSB | Arylsulfatase B | 3.23 | N |
| HYAL1 | Hyaluronidase 1 | 3.23 | N |
| TXNIP | Thioredoxin Interacting Protein | 3.23 | N |
| MIR503 | MicroRNA 503 | 3.23 | N |
| HNRNPA2B1 | Heterogeneous Nuclear Ribonucleoprotein A2/B1 | 3.22 | N |
| CYP2B6 | Cytochrome P450 Family 2 Subfamily B Member 6 | 3.22 | N |
| HSPB2 | Heat Shock Protein Family B (Small) Member 2 | 3.22 | N |
| KLF14 | Kruppel Like Factor 14 | 3.22 | N |
| PRDX1 | Peroxiredoxin 1 | 3.22 | N |
| AMBP | Alpha-1-Microglobulin/Bikunin Precursor | 3.21 | N |
| CD274 | CD274 Molecule | 3.21 | N |
| HLA-DQA1 | Major Histocompatibility Complex, Class II, DQ Alpha 1 | 3.2 | N |
| SELENOS | Selenoprotein S | 3.2 | N |
| BNC2 | Basonuclin 2 | 3.2 | N |
| KLK3 | Kallikrein Related Peptidase 3 | 3.2 | N |
| SHH | Sonic Hedgehog Signaling Molecule | 3.2 | N |
| SBF2 | SET Binding Factor 2 | 3.19 | N |
| ACVR2A | Activin A Receptor Type 2A | 3.19 | N |
| BIRC5 | Baculoviral IAP Repeat Containing 5 | 3.19 | N |
| NPHS1 | NPHS1 Adhesion Molecule, Nephrin | 3.19 | N |
| NES | Nestin | 3.18 | N |
| MT-CYB | Mitochondrially Encoded Cytochrome B | 3.17 | N |
| ANXA1 | Annexin A1 | 3.17 | N |
| LIFR | LIF Receptor Subunit Alpha | 3.17 | N |
| DHCR24 | 24-Dehydrocholesterol Reductase | 3.17 | N |
| CCN1 | Cellular Communication Network Factor 1 | 3.16 | N |
| SORCS1 | Sortilin Related VPS10 Domain Containing Receptor 1 | 3.16 | N |
| C1QTNF3 | C1q And TNF Related 3 | 3.15 | N |
| MIR361 | MicroRNA 361 | 3.15 | N |
| APOD | Apolipoprotein D | 3.15 | N |
| SPRY2 | Sprouty RTK Signaling Antagonist 2 | 3.14 | N |
| S100A4 | S100 Calcium Binding Protein A4 | 3.14 | N |
| HRH2 | Histamine Receptor H2 | 3.14 | N |
| GLRX | Glutaredoxin | 3.13 | N |
| PRDX5 | Peroxiredoxin 5 | 3.13 | N |
| ELOVL2 | ELOVL Fatty Acid Elongase 2 | 3.13 | N |
| LTB4R | Leukotriene B4 Receptor | 3.13 | N |
| NUCB2 | Nucleobindin 2 | 3.12 | N |
| TCN2 | Transcobalamin 2 | 3.12 | N |
| ZNF385D | Zinc Finger Protein 385D | 3.12 | N |
| ABCB4 | ATP Binding Cassette Subfamily B Member 4 | 3.11 | N |
| CAMP | Cathelicidin Antimicrobial Peptide | 3.11 | N |
| IL7 | Interleukin 7 | 3.11 | N |
| PCOLCE2 | Procollagen C-Endopeptidase Enhancer 2 | 3.11 | N |
| ARNTL | Aryl Hydrocarbon Receptor Nuclear Translocator Like | 3.11 | N |
| C12orf43 | Chromosome 12 Open Reading Frame 43 | 3.11 | N |
| IL5RA | Interleukin 5 Receptor Subunit Alpha | 3.11 | N |
| TLR10 | Toll Like Receptor 10 | 3.11 | N |
| NOX5 | NADPH Oxidase 5 | 3.11 | N |
| FST | Follistatin | 3.1 | N |
| PDE3B | Phosphodiesterase 3B | 3.1 | N |
| EBI3 | Epstein-Barr Virus Induced 3 | 3.1 | N |
| HRG | Histidine Rich Glycoprotein | 3.1 | N |
| IL9 | Interleukin 9 | 3.09 | N |
| MIR92A2 | MicroRNA 92a-2 | 3.09 | N |
| PTPA | Protein Phosphatase 2 Phosphatase Activator | 3.09 | N |
| FGFR4 | Fibroblast Growth Factor Receptor 4 | 3.06 | N |
| CPOX | Coproporphyrinogen Oxidase | 3.06 | N |
| POU2F1 | POU Class 2 Homeobox 1 | 3.06 | N |
| UCN | Urocortin | 3.06 | N |
| HDAC4 | Histone Deacetylase 4 | 3.05 | N |
| DYRK1A | Dual Specificity Tyrosine Phosphorylation Regulated Kinase 1A | 3.04 | N |
| MTHFD1 | Methylenetetrahydrofolate Dehydrogenase, Cyclohydrolase And Formyltetrahydrofolate Synthetase 1 | 3.04 | N |
| GSTA4 | Glutathione S-Transferase Alpha 4 | 3.03 | N |
| MAP3K5 | Mitogen-Activated Protein Kinase Kinase Kinase 5 | 3.03 | N |
| PDE4B | Phosphodiesterase 4B | 3.03 | N |
| CALM1 | Calmodulin 1 | 3.03 | N |
| F2RL2 | Coagulation Factor II Thrombin Receptor Like 2 | 3.02 | N |
| DMPK | DM1 Protein Kinase | 3.01 | N |
| BHMT2 | Betaine--Homocysteine S-Methyltransferase 2 | 3.01 | N |
| GNLY | Granulysin | 3.01 | N |
| STEAP2-AS1 | STEAP2 Antisense RNA 1 | 3.01 | N |
| CDH4 | Cadherin 4 | 3 | N |
| BTNL2 | Butyrophilin Like 2 | 3 | N |
| DDIT3 | DNA Damage Inducible Transcript 3 | 3 | N |
| FLRT2 | Fibronectin Leucine Rich Transmembrane Protein 2 | 3 | N |
| ANGPTL2 | Angiopoietin Like 2 | 3 | N |
| ERBB4 | Erb-B2 Receptor Tyrosine Kinase 4 | 2.99 | N |
| CARD8 | Caspase Recruitment Domain Family Member 8 | 2.99 | N |
| DEFA1 | Defensin Alpha 1 | 2.99 | N |
| P4HA3 | Prolyl 4-Hydroxylase Subunit Alpha 3 | 2.98 | N |
| MEOX2 | Mesenchyme Homeobox 2 | 2.98 | N |
| NPY1R | Neuropeptide Y Receptor Y1 | 2.98 | N |
| CYSLTR1 | Cysteinyl Leukotriene Receptor 1 | 2.97 | N |
| TFAP2A | Transcription Factor AP-2 Alpha | 2.97 | N |
| TACR1 | Tachykinin Receptor 1 | 2.97 | N |
| LRGUK | Leucine Rich Repeats And Guanylate Kinase Domain Containing | 2.97 | N |
| AIFM1 | Apoptosis Inducing Factor Mitochondria Associated 1 | 2.97 | N |
| SCGB1A1 | Secretoglobin Family 1A Member 1 | 2.97 | N |
| IFNGR2 | Interferon Gamma Receptor 2 | 2.96 | N |
| PNPLA3 | Patatin Like Phospholipase Domain Containing 3 | 2.96 | N |
| TNFRSF12A | TNF Receptor Superfamily Member 12A | 2.96 | N |
| CFDP1 | Craniofacial Development Protein 1 | 2.96 | N |
| TMEM170A | Transmembrane Protein 170A | 2.96 | N |
| IDH2 | Isocitrate Dehydrogenase (NADP(+)) 2 | 2.95 | N |
| CAV3 | Caveolin 3 | 2.95 | N |
| DICER1 | Dicer 1, Ribonuclease III | 2.95 | N |
| LOXL1 | Lysyl Oxidase Like 1 | 2.94 | N |
| BLVRA | Biliverdin Reductase A | 2.94 | N |
| IGFBP5 | Insulin Like Growth Factor Binding Protein 5 | 2.93 | N |
| UBA52P6 | Ubiquitin A-52 Residue Ribosomal Protein Fusion Product 1 Pseudogene 6 | 2.93 | N |
| RYR3 | Ryanodine Receptor 3 | 2.93 | N |
| LARGE1 | LARGE Xylosyl- And Glucuronyltransferase 1 | 2.93 | N |
| CLEC4C | C-Type Lectin Domain Family 4 Member C | 2.93 | N |
| RNPC3 | RNA Binding Region (RNP1, RRM) Containing 3 | 2.93 | N |
| HLA-G | Major Histocompatibility Complex, Class I, G | 2.92 | N |
| EREG | Epiregulin | 2.92 | N |
| CDKAL1 | CDK5 Regulatory Subunit Associated Protein 1 Like 1 | 2.91 | N |
| NUDT6 | Nudix Hydrolase 6 | 2.91 | N |
| CYP1A2 | Cytochrome P450 Family 1 Subfamily A Member 2 | 2.91 | N |
| ESAM | Endothelial Cell Adhesion Molecule | 2.91 | N |
| AP3B1 | Adaptor Related Protein Complex 3 Subunit Beta 1 | 2.91 | N |
| IGF2BP2 | Insulin Like Growth Factor 2 MRNA Binding Protein 2 | 2.9 | N |
| LPIN3 | Lipin 3 | 2.9 | N |
| PTGES | Prostaglandin E Synthase | 2.9 | N |
| ALPP | Alkaline Phosphatase, Placental | 2.9 | N |
| SHOX | Short Stature Homeobox | 2.89 | N |
| LGALS3BP | Galectin 3 Binding Protein | 2.89 | N |
| ICAM2 | Intercellular Adhesion Molecule 2 | 2.88 | N |
| EPAS1 | Endothelial PAS Domain Protein 1 | 2.87 | N |
| HNRNPA1 | Heterogeneous Nuclear Ribonucleoprotein A1 | 2.87 | N |
| WNT5A | Wnt Family Member 5A | 2.87 | N |
| LOC157273 | Uncharacterized LOC157273 | 2.87 | N |
| HSPA5 | Heat Shock Protein Family A (Hsp70) Member 5 | 2.87 | N |
| NFKBIB | NFKB Inhibitor Beta | 2.87 | N |
| COL2A1 | Collagen Type II Alpha 1 Chain | 2.87 | N |
| FAT4 | FAT Atypical Cadherin 4 | 2.86 | N |
| QKI | QKI, KH Domain Containing RNA Binding | 2.85 | N |
| PSMB8 | Proteasome 20S Subunit Beta 8 | 2.85 | N |
| FOSL1 | FOS Like 1, AP-1 Transcription Factor Subunit | 2.85 | N |
| FASN | Fatty Acid Synthase | 2.85 | N |
| TFPI2 | Tissue Factor Pathway Inhibitor 2 | 2.85 | N |
| F11R | F11 Receptor | 2.85 | N |
| LMAN1 | Lectin, Mannose Binding 1 | 2.84 | N |
| TANC1 | Tetratricopeptide Repeat, Ankyrin Repeat And Coiled-Coil Containing 1 | 2.84 | N |
| EPHA3 | EPH Receptor A3 | 2.84 | N |
| CYP21A2 | Cytochrome P450 Family 21 Subfamily A Member 2 | 2.84 | N |
| TCN1 | Transcobalamin 1 | 2.84 | N |
| MAP1LC3A | Microtubule Associated Protein 1 Light Chain 3 Alpha | 2.84 | N |
| MYB | MYB Proto-Oncogene, Transcription Factor | 2.84 | N |
| FOXP2 | Forkhead Box P2 | 2.83 | N |
| MBL1P | Mannose Binding Lectin 1, Pseudogene | 2.82 | N |
| ITGA6 | Integrin Subunit Alpha 6 | 2.82 | N |
| SLC12A3 | Solute Carrier Family 12 Member 3 | 2.82 | N |
| MYCN | MYCN Proto-Oncogene, BHLH Transcription Factor | 2.82 | N |
| RPL15P15 | Ribosomal Protein L15 Pseudogene 15 | 2.81 | N |
| FGF19 | Fibroblast Growth Factor 19 | 2.81 | N |
| MSH5 | MutS Homolog 5 | 2.81 | N |
| RRAS | RAS Related | 2.81 | N |
| MERTK | MER Proto-Oncogene, Tyrosine Kinase | 2.8 | N |
| FCGR2C | Fc Fragment Of IgG Receptor IIc (Gene/Pseudogene) | 2.8 | N |
| ELK1 | ETS Transcription Factor ELK1 | 2.8 | N |
| APOF | Apolipoprotein F | 2.8 | N |
| GRIN3A | Glutamate Ionotropic Receptor NMDA Type Subunit 3A | 2.8 | N |
| HSPB6 | Heat Shock Protein Family B (Small) Member 6 | 2.8 | N |
| PLXNA4 | Plexin A4 | 2.8 | N |
| BUD13 | BUD13 Homolog | 2.8 | N |
| OGG1 | 8-Oxoguanine DNA Glycosylase | 2.79 | N |
| OLFML2B | Olfactomedin Like 2B | 2.79 | N |
| AGK | Acylglycerol Kinase | 2.79 | N |
| TSLP | Thymic Stromal Lymphopoietin | 2.79 | N |
| SDC4 | Syndecan 4 | 2.79 | N |
| C4BPA | Complement Component 4 Binding Protein Alpha | 2.78 | N |
| TMEM106B | Transmembrane Protein 106B | 2.78 | N |
| GABRG1 | Gamma-Aminobutyric Acid Type A Receptor Subunit Gamma1 | 2.78 | N |
| IL22 | Interleukin 22 | 2.78 | N |
| STS | Steroid Sulfatase | 2.77 | N |
| OMD | Osteomodulin | 2.77 | N |
| GNAI1 | G Protein Subunit Alpha I1 | 2.77 | N |
| TNFRSF6B | TNF Receptor Superfamily Member 6b | 2.77 | N |
| SERPINB1 | Serpin Family B Member 1 | 2.77 | N |
| PAK1 | P21 (RAC1) Activated Kinase 1 | 2.76 | N |
| LPAR1 | Lysophosphatidic Acid Receptor 1 | 2.76 | N |
| CREBBP | CREB Binding Protein | 2.76 | N |
| AQP1 | Aquaporin 1 (Colton Blood Group) | 2.76 | N |
| NOD1 | Nucleotide Binding Oligomerization Domain Containing 1 | 2.76 | N |
| IL20 | Interleukin 20 | 2.76 | N |
| HSPA1B | Heat Shock Protein Family A (Hsp70) Member 1B | 2.75 | N |
| MTFP1 | Mitochondrial Fission Process 1 | 2.75 | N |
| TXNL4B | Thioredoxin Like 4B | 2.75 | N |
| MSRA | Methionine Sulfoxide Reductase A | 2.75 | N |
| LTC4S | Leukotriene C4 Synthase | 2.74 | N |
| CCL26 | C-C Motif Chemokine Ligand 26 | 2.74 | N |
| PTK2B | Protein Tyrosine Kinase 2 Beta | 2.73 | N |
| PTHLH | Parathyroid Hormone Like Hormone | 2.73 | N |
| CYSLTR2 | Cysteinyl Leukotriene Receptor 2 | 2.73 | N |
| CCDC71L | Coiled-Coil Domain Containing 71 Like | 2.72 | N |
| GPR132 | G Protein-Coupled Receptor 132 | 2.71 | N |
| CNR2 | Cannabinoid Receptor 2 | 2.71 | N |
| ETV6 | ETS Variant Transcription Factor 6 | 2.7 | N |
| PRKAB2 | Protein Kinase AMP-Activated Non-Catalytic Subunit Beta 2 | 2.69 | N |
| RGS9BP | Regulator Of G Protein Signaling 9 Binding Protein | 2.69 | N |
| EFNA5 | Ephrin A5 | 2.69 | N |
| TNFSF15 | TNF Superfamily Member 15 | 2.69 | N |
| NCF1C | Neutrophil Cytosolic Factor 1C Pseudogene | 2.68 | N |
| TYMP | Thymidine Phosphorylase | 2.68 | N |
| FCGR2B | Fc Fragment Of IgG Receptor IIb | 2.68 | N |
| LRP2 | LDL Receptor Related Protein 2 | 2.67 | N |
| IFNGR1 | Interferon Gamma Receptor 1 | 2.67 | N |
| PI3 | Peptidase Inhibitor 3 | 2.67 | N |
| CD47 | CD47 Molecule | 2.67 | N |
| GNRH1 | Gonadotropin Releasing Hormone 1 | 2.66 | N |
| MET | MET Proto-Oncogene, Receptor Tyrosine Kinase | 2.66 | N |
| FCAMR | Fc Fragment Of IgA And IgM Receptor | 2.66 | N |
| LGALS1 | Galectin 1 | 2.66 | N |
| REG1A | Regenerating Family Member 1 Alpha | 2.66 | N |
| TBX21 | T-Box Transcription Factor 21 | 2.65 | N |
| CSMD1 | CUB And Sushi Multiple Domains 1 | 2.64 | N |
| TERC | Telomerase RNA Component | 2.63 | N |
| SELENOP | Selenoprotein P | 2.63 | N |
| HPR | Haptoglobin-Related Protein | 2.63 | N |
| MAP2K6 | Mitogen-Activated Protein Kinase Kinase 6 | 2.63 | N |
| TUSC1 | Tumor Suppressor Candidate 1 | 2.63 | N |
| PLIN2 | Perilipin 2 | 2.63 | N |
| ROS1 | ROS Proto-Oncogene 1, Receptor Tyrosine Kinase | 2.62 | N |
| INSIG1 | Insulin Induced Gene 1 | 2.62 | N |
| IRF2BP2 | Interferon Regulatory Factor 2 Binding Protein 2 | 2.62 | N |
| CHRNB4 | Cholinergic Receptor Nicotinic Beta 4 Subunit | 2.62 | N |
| PIGR | Polymeric Immunoglobulin Receptor | 2.62 | N |
| SUPT3H | SPT3 Homolog, SAGA And STAGA Complex Component | 2.62 | N |
| TRPC4 | Transient Receptor Potential Cation Channel Subfamily C Member 4 | 2.62 | N |
| NOG | Noggin | 2.61 | N |
| ICOS | Inducible T Cell Costimulator | 2.61 | N |
| MAFB | MAF BZIP Transcription Factor B | 2.61 | N |
| PEX5 | Peroxisomal Biogenesis Factor 5 | 2.59 | N |
| PLD5 | Phospholipase D Family Member 5 | 2.59 | N |
| IL24 | Interleukin 24 | 2.59 | N |
| TRB | T Cell Receptor Beta Locus | 2.59 | N |
| FOLH1 | Folate Hydrolase 1 | 2.58 | N |
| COMP | Cartilage Oligomeric Matrix Protein | 2.58 | N |
| VENTXP2 | VENT Homeobox Pseudogene 2 | 2.58 | N |
| RPL36AP23 | Ribosomal Protein L36a Pseudogene 23 | 2.58 | N |
| NCOA1 | Nuclear Receptor Coactivator 1 | 2.57 | N |
| SLC9A3R2 | SLC9A3 Regulator 2 | 2.57 | N |
| SLC22A12 | Solute Carrier Family 22 Member 12 | 2.57 | N |
| CACNA1E | Calcium Voltage-Gated Channel Subunit Alpha1 E | 2.57 | N |
| HLX | H2.0 Like Homeobox | 2.57 | N |
| THSD1 | Thrombospondin Type 1 Domain Containing 1 | 2.56 | N |
| HSP90B1 | Heat Shock Protein 90 Beta Family Member 1 | 2.56 | N |
| IFT88 | Intraflagellar Transport 88 | 2.56 | N |
| KCNQ3 | Potassium Voltage-Gated Channel Subfamily Q Member 3 | 2.56 | N |
| SRY | Sex Determining Region Y | 2.55 | N |
| RAMP3 | Receptor Activity Modifying Protein 3 | 2.55 | N |
| ATP5IF1 | ATP Synthase Inhibitory Factor Subunit 1 | 2.55 | N |
| MIR19B2 | MicroRNA 19b-2 | 2.55 | N |
| AGBL1 | ATP/GTP Binding Protein Like 1 | 2.55 | N |
| NR4A2 | Nuclear Receptor Subfamily 4 Group A Member 2 | 2.55 | N |
| TNFSF14 | TNF Superfamily Member 14 | 2.55 | N |
| MXD1 | MAX Dimerization Protein 1 | 2.55 | N |
| MAGI2 | Membrane Associated Guanylate Kinase, WW And PDZ Domain Containing 2 | 2.54 | N |
| NUP107 | Nucleoporin 107 | 2.54 | N |
| MIR185 | MicroRNA 185 | 2.53 | N |
| SPATA7 | Spermatogenesis Associated 7 | 2.52 | N |
| ADAM8 | ADAM Metallopeptidase Domain 8 | 2.52 | N |
| SEMA3E | Semaphorin 3E | 2.52 | N |
| CDH9 | Cadherin 9 | 2.52 | N |
| FBXO15 | F-Box Protein 15 | 2.52 | N |
| LOC111365141 | NOS2 5' Regulatory Region | 2.52 | N |
| ATF1 | Activating Transcription Factor 1 | 2.51 | N |
| SLC24A3 | Solute Carrier Family 24 Member 3 | 2.51 | N |
| PAFAH2 | Platelet Activating Factor Acetylhydrolase 2 | 2.51 | N |
| MASP1 | Mannan Binding Lectin Serine Peptidase 1 | 2.51 | N |
| GSTM3 | Glutathione S-Transferase Mu 3 | 2.51 | N |
| SEMA5A | Semaphorin 5A | 2.5 | N |
| RHOB | Ras Homolog Family Member B | 2.5 | N |
| C3AR1 | Complement C3a Receptor 1 | 2.5 | N |
| BCO1 | Beta-Carotene Oxygenase 1 | 2.5 | N |
| KCNN4 | Potassium Calcium-Activated Channel Subfamily N Member 4 | 2.5 | N |
| ADAMTS3 | ADAM Metallopeptidase With Thrombospondin Type 1 Motif 3 | 2.49 | N |
| SCML4 | Scm Polycomb Group Protein Like 4 | 2.49 | N |
| GNA12 | G Protein Subunit Alpha 12 | 2.49 | N |
| GPX4 | Glutathione Peroxidase 4 | 2.49 | N |
| SLC19A1 | Solute Carrier Family 19 Member 1 | 2.48 | N |
| FGF10 | Fibroblast Growth Factor 10 | 2.48 | N |
| SGIP1 | SH3GL Interacting Endocytic Adaptor 1 | 2.48 | N |
| SEZ6L | Seizure Related 6 Homolog Like | 2.47 | N |
| TUBA4A | Tubulin Alpha 4a | 2.47 | N |
| H6PD | Hexose-6-Phosphate Dehydrogenase/Glucose 1-Dehydrogenase | 2.47 | N |
| FHL1 | Four And A Half LIM Domains 1 | 2.47 | N |
| TARDBP | TAR DNA Binding Protein | 2.47 | N |
| MIR31 | MicroRNA 31 | 2.47 | N |
| EPRS1 | Glutamyl-Prolyl-TRNA Synthetase 1 | 2.46 | N |
| PLEKHA1 | Pleckstrin Homology Domain Containing A1 | 2.46 | N |
| DUSP2 | Dual Specificity Phosphatase 2 | 2.46 | N |
| SCG2 | Secretogranin II | 2.46 | N |
| UTS2B | Urotensin 2B | 2.46 | N |
| ANPEP | Alanyl Aminopeptidase, Membrane | 2.46 | N |
| PXN | Paxillin | 2.46 | N |
| PEPD | Peptidase D | 2.46 | N |
| XKR4 | XK Related 4 | 2.46 | N |
| TRPC4AP | Transient Receptor Potential Cation Channel Subfamily C Member 4 Associated Protein | 2.45 | N |
| FAP | Fibroblast Activation Protein Alpha | 2.45 | N |
| RXRG | Retinoid X Receptor Gamma | 2.45 | N |
| FABP5 | Fatty Acid Binding Protein 5 | 2.45 | N |
| ADAM33 | ADAM Metallopeptidase Domain 33 | 2.45 | N |
| KCNJ1 | Potassium Inwardly Rectifying Channel Subfamily J Member 1 | 2.45 | N |
| GRK6 | G Protein-Coupled Receptor Kinase 6 | 2.44 | N |
| LTB | Lymphotoxin Beta | 2.44 | N |
| NGFR | Nerve Growth Factor Receptor | 2.44 | N |
| MIR125B1 | MicroRNA 125b-1 | 2.44 | N |
| FEM1A | Fem-1 Homolog A | 2.44 | N |
| TBK1 | TANK Binding Kinase 1 | 2.44 | N |
| TRA | T Cell Receptor Alpha Locus | 2.44 | N |
| HDLC3 | High Density Lipoprotein Cholesterol, Low Serum, 3 | 2.44 | N |
| ODC1 | Ornithine Decarboxylase 1 | 2.43 | N |
| PRKAG3 | Protein Kinase AMP-Activated Non-Catalytic Subunit Gamma 3 | 2.43 | N |
| GLO1 | Glyoxalase I | 2.43 | N |
| ID1 | Inhibitor Of DNA Binding 1, HLH Protein | 2.43 | N |
| GSTM4 | Glutathione S-Transferase Mu 4 | 2.42 | N |
| USP24 | Ubiquitin Specific Peptidase 24 | 2.42 | N |
| MIR93 | MicroRNA 93 | 2.42 | N |
| SAMSN1 | SAM Domain, SH3 Domain And Nuclear Localization Signals 1 | 2.41 | N |
| PCSK5 | Proprotein Convertase Subtilisin/Kexin Type 5 | 2.41 | N |
| IL7R | Interleukin 7 Receptor | 2.41 | N |
| MSX2 | Msh Homeobox 2 | 2.41 | N |
| MPRIP | Myosin Phosphatase Rho Interacting Protein | 2.41 | N |
| KEAP1 | Kelch Like ECH Associated Protein 1 | 2.41 | N |
| CLIP1 | CAP-Gly Domain Containing Linker Protein 1 | 2.41 | N |
| KCNK1 | Potassium Two Pore Domain Channel Subfamily K Member 1 | 2.4 | N |
| HAS1 | Hyaluronan Synthase 1 | 2.4 | N |
| TRPS1 | Transcriptional Repressor GATA Binding 1 | 2.4 | N |
| PRKCG | Protein Kinase C Gamma | 2.4 | N |
| NME2 | NME/NM23 Nucleoside Diphosphate Kinase 2 | 2.4 | N |
| SLC34A1 | Solute Carrier Family 34 Member 1 | 2.4 | N |
| NFKBIE | NFKB Inhibitor Epsilon | 2.4 | N |
| SFRP4 | Secreted Frizzled Related Protein 4 | 2.4 | N |
| POR | Cytochrome P450 Oxidoreductase | 2.4 | N |
| SF3A1 | Splicing Factor 3a Subunit 1 | 2.4 | N |
| TGFA | Transforming Growth Factor Alpha | 2.39 | N |
| HHEX | Hematopoietically Expressed Homeobox | 2.39 | N |
| RGS9 | Regulator Of G Protein Signaling 9 | 2.39 | N |
| CNTNAP2 | Contactin Associated Protein 2 | 2.39 | N |
| MIR15A | MicroRNA 15a | 2.39 | N |
| IGES | Immunoglobulin E Concentration, Serum | 2.38 | N |
| GATA3 | GATA Binding Protein 3 | 2.38 | N |
| FGF3 | Fibroblast Growth Factor 3 | 2.37 | N |
| CLOCK | Clock Circadian Regulator | 2.37 | N |
| SDC2 | Syndecan 2 | 2.37 | N |
| SLC17A3 | Solute Carrier Family 17 Member 3 | 2.37 | N |
| MIR149 | MicroRNA 149 | 2.37 | N |
| FBLN1 | Fibulin 1 | 2.37 | N |
| E2F1 | E2F Transcription Factor 1 | 2.37 | N |
| PRDM9 | PR/SET Domain 9 | 2.37 | N |
| TMEM132B | Transmembrane Protein 132B | 2.37 | N |
| RIT2 | Ras Like Without CAAX 2 | 2.36 | N |
| TMEM258 | Transmembrane Protein 258 | 2.36 | N |
| VPS33A | VPS33A Core Subunit Of CORVET And HOPS Complexes | 2.36 | N |
| UMOD | Uromodulin | 2.36 | N |
| ST8SIA4 | ST8 Alpha-N-Acetyl-Neuraminide Alpha-2,8-Sialyltransferase 4 | 2.36 | N |
| MIPEP | Mitochondrial Intermediate Peptidase | 2.36 | N |
| ECE2 | Endothelin Converting Enzyme 2 | 2.36 | N |
| TRPC5 | Transient Receptor Potential Cation Channel Subfamily C Member 5 | 2.36 | N |
| MCF2L | MCF.2 Cell Line Derived Transforming Sequence Like | 2.35 | N |
| PSAP | Prosaposin | 2.35 | N |
| ST3GAL1 | ST3 Beta-Galactoside Alpha-2,3-Sialyltransferase 1 | 2.35 | N |
| ATXN1 | Ataxin 1 | 2.34 | N |
| TRIM9 | Tripartite Motif Containing 9 | 2.34 | N |
| ACAT2 | Acetyl-CoA Acetyltransferase 2 | 2.34 | N |
| HYOU1 | Hypoxia Up-Regulated 1 | 2.34 | N |
| SUCLG1 | Succinate-CoA Ligase GDP/ADP-Forming Subunit Alpha | 2.34 | N |
| NDUFS4 | NADH:Ubiquinone Oxidoreductase Subunit S4 | 2.34 | N |
| LGMN | Legumain | 2.33 | N |
| APH1B | Aph-1 Homolog B, Gamma-Secretase Subunit | 2.33 | N |
| SLC2A13 | Solute Carrier Family 2 Member 13 | 2.33 | N |
| C1QL3 | Complement C1q Like 3 | 2.33 | N |
| AQP7 | Aquaporin 7 | 2.33 | N |
| FETUB | Fetuin B | 2.33 | N |
| TSC1 | TSC Complex Subunit 1 | 2.33 | N |
| ENHO | Energy Homeostasis Associated | 2.32 | N |
| KCNK2 | Potassium Two Pore Domain Channel Subfamily K Member 2 | 2.32 | N |
| SH3GL2 | SH3 Domain Containing GRB2 Like 2, Endophilin A1 | 2.32 | N |
| NFATC2 | Nuclear Factor Of Activated T Cells 2 | 2.31 | N |
| GSTM5 | Glutathione S-Transferase Mu 5 | 2.31 | N |
| LINC01535 | Long Intergenic Non-Protein Coding RNA 1535 | 2.31 | N |
| B4GALT1 | Beta-1,4-Galactosyltransferase 1 | 2.31 | N |
| ATP5F1B | ATP Synthase F1 Subunit Beta | 2.31 | N |
| TJP1 | Tight Junction Protein 1 | 2.3 | N |
| TCIRG1 | T Cell Immune Regulator 1, ATPase H+ Transporting V0 Subunit A3 | 2.3 | N |
| TYROBP | Transmembrane Immune Signaling Adaptor TYROBP | 2.3 | N |
| CSRP1 | Cysteine And Glycine Rich Protein 1 | 2.3 | N |
| UBLCP1 | Ubiquitin Like Domain Containing CTD Phosphatase 1 | 2.3 | N |
| NR1I3 | Nuclear Receptor Subfamily 1 Group I Member 3 | 2.3 | N |
| AMPD2 | Adenosine Monophosphate Deaminase 2 | 2.29 | N |
| MIR27A | MicroRNA 27a | 2.29 | N |
| SNF8 | SNF8 Subunit Of ESCRT-II | 2.28 | N |
| METRNL | Meteorin Like, Glial Cell Differentiation Regulator | 2.28 | N |
| SLC29A1 | Solute Carrier Family 29 Member 1 (Augustine Blood Group) | 2.28 | N |
| A2M | Alpha-2-Macroglobulin | 2.28 | N |
| MIR26A1 | MicroRNA 26a-1 | 2.28 | N |
| CCNB1 | Cyclin B1 | 2.28 | N |
| YAP1 | Yes Associated Protein 1 | 2.27 | N |
| APEX1 | Apurinic/Apyrimidinic Endodeoxyribonuclease 1 | 2.27 | N |
| XRCC3 | X-Ray Repair Cross Complementing 3 | 2.27 | N |
| KCNK13 | Potassium Two Pore Domain Channel Subfamily K Member 13 | 2.27 | N |
| LPP | LIM Domain Containing Preferred Translocation Partner In Lipoma | 2.27 | N |
| PRKAG1 | Protein Kinase AMP-Activated Non-Catalytic Subunit Gamma 1 | 2.27 | N |
| MSH3 | MutS Homolog 3 | 2.27 | N |
| CAPN5 | Calpain 5 | 2.27 | N |
| MYO7A | Myosin VIIA | 2.27 | N |
| MRPL10 | Mitochondrial Ribosomal Protein L10 | 2.27 | N |
| FEM1B | Fem-1 Homolog B | 2.27 | N |
| WDR33 | WD Repeat Domain 33 | 2.27 | N |
| CCDC157 | Coiled-Coil Domain Containing 157 | 2.27 | N |
| MIR124-3 | MicroRNA 124-3 | 2.26 | N |
| LYSMD4 | LysM Domain Containing 4 | 2.26 | N |
| CD151 | CD151 Molecule (Raph Blood Group) | 2.26 | N |
| PIK3R3 | Phosphoinositide-3-Kinase Regulatory Subunit 3 | 2.26 | N |
| CLEC3B | C-Type Lectin Domain Family 3 Member B | 2.25 | N |
| SVEP1 | Sushi, Von Willebrand Factor Type A, EGF And Pentraxin Domain Containing 1 | 2.25 | N |
| MSBP1 | Minisatellite Binding Protein 1 | 2.25 | N |
| CTTN | Cortactin | 2.24 | N |
| ADGRE5 | Adhesion G Protein-Coupled Receptor E5 | 2.24 | N |
| CNTN1 | Contactin 1 | 2.24 | N |
| PCCB | Propionyl-CoA Carboxylase Subunit Beta | 2.23 | N |
| NRG3 | Neuregulin 3 | 2.23 | N |
| FBXO33 | F-Box Protein 33 | 2.23 | N |
| RPL23P4 | Ribosomal Protein L23 Pseudogene 4 | 2.23 | N |
| RPL35AP15 | Ribosomal Protein L35a Pseudogene 15 | 2.23 | N |
| RTN3 | Reticulon 3 | 2.23 | N |
| AOC1 | Amine Oxidase Copper Containing 1 | 2.23 | N |
| ANK1 | Ankyrin 1 | 2.23 | N |
| RCAN1 | Regulator Of Calcineurin 1 | 2.23 | N |
| KRI1 | KRI1 Homolog | 2.23 | N |
| CPT1A | Carnitine Palmitoyltransferase 1A | 2.23 | N |
| IPMK | Inositol Polyphosphate Multikinase | 2.23 | N |
| ALDH8A1 | Aldehyde Dehydrogenase 8 Family Member A1 | 2.23 | N |
| SPC24 | SPC24 Component Of NDC80 Kinetochore Complex | 2.22 | N |
| DUOX1 | Dual Oxidase 1 | 2.22 | N |
| SGK1 | Serum/Glucocorticoid Regulated Kinase 1 | 2.22 | N |
| TES | Testin LIM Domain Protein | 2.22 | N |
| TGFBI | Transforming Growth Factor Beta Induced | 2.22 | N |
| NCOA2 | Nuclear Receptor Coactivator 2 | 2.22 | N |
| MKI67 | Marker Of Proliferation Ki-67 | 2.22 | N |
| SLC1A1 | Solute Carrier Family 1 Member 1 | 2.21 | N |
| MIR30C2 | MicroRNA 30c-2 | 2.2 | N |
| SOX6 | SRY-Box Transcription Factor 6 | 2.2 | N |
| RRM1 | Ribonucleotide Reductase Catalytic Subunit M1 | 2.2 | N |
| TOR1A | Torsin Family 1 Member A | 2.2 | N |
| EMP1 | Epithelial Membrane Protein 1 | 2.19 | N |
| ZNF383 | Zinc Finger Protein 383 | 2.19 | N |
| SEMA6D | Semaphorin 6D | 2.19 | N |
| UTP20 | UTP20 Small Subunit Processome Component | 2.18 | N |
| HDGFL1 | HDGF Like 1 | 2.18 | N |
| HDLBP | High Density Lipoprotein Binding Protein | 2.18 | N |
| ILF3 | Interleukin Enhancer Binding Factor 3 | 2.18 | N |
| CALU | Calumenin | 2.18 | N |
| CDK14 | Cyclin Dependent Kinase 14 | 2.18 | N |
| KCNA3 | Potassium Voltage-Gated Channel Subfamily A Member 3 | 2.17 | N |
| IL19 | Interleukin 19 | 2.17 | N |
| MX1 | MX Dynamin Like GTPase 1 | 2.16 | N |
| PMM2 | Phosphomannomutase 2 | 2.16 | N |
| MSI2 | Musashi RNA Binding Protein 2 | 2.16 | N |
| ADAMTS5 | ADAM Metallopeptidase With Thrombospondin Type 1 Motif 5 | 2.16 | N |
| RLN2 | Relaxin 2 | 2.16 | N |
| MIR30B | MicroRNA 30b | 2.16 | N |
| GSTO1 | Glutathione S-Transferase Omega 1 | 2.15 | N |
| HNRNPA1P10 | Heterogeneous Nuclear Ribonucleoprotein A1 Pseudogene 10 | 2.15 | N |
| FAM13A | Family With Sequence Similarity 13 Member A | 2.14 | N |
| ORAI1 | ORAI Calcium Release-Activated Calcium Modulator 1 | 2.14 | N |
| LGALS9 | Galectin 9 | 2.14 | N |
| GSTM2 | Glutathione S-Transferase Mu 2 | 2.14 | N |
| SIRT3 | Sirtuin 3 | 2.13 | N |
| PRMT5P1 | Protein Arginine Methyltransferase 5 Pseudogene 1 | 2.13 | N |
| HTN3 | Histatin 3 | 2.13 | N |
| P2RX4 | Purinergic Receptor P2X 4 | 2.13 | N |
| NHLRC1 | NHL Repeat Containing E3 Ubiquitin Protein Ligase 1 | 2.13 | N |
| HDAC2 | Histone Deacetylase 2 | 2.12 | N |
| ESRRB | Estrogen Related Receptor Beta | 2.12 | N |
| WNT1 | Wnt Family Member 1 | 2.12 | N |
| LIG4 | DNA Ligase 4 | 2.12 | N |
| ATN1 | Atrophin 1 | 2.12 | N |
| FMN1 | Formin 1 | 2.11 | N |
| CCDC159 | Coiled-Coil Domain Containing 159 | 2.11 | N |
| MIR616 | MicroRNA 616 | 2.11 | N |
| PPP3R1 | Protein Phosphatase 3 Regulatory Subunit B, Alpha | 2.11 | N |
| CTCF | CCCTC-Binding Factor | 2.11 | N |
| IFNAR2 | Interferon Alpha And Beta Receptor Subunit 2 | 2.1 | N |
| CD70 | CD70 Molecule | 2.1 | N |
| PRICKLE2 | Prickle Planar Cell Polarity Protein 2 | 2.09 | N |
| SORCS2 | Sortilin Related VPS10 Domain Containing Receptor 2 | 2.09 | N |
| BCAM | Basal Cell Adhesion Molecule (Lutheran Blood Group) | 2.09 | N |
| GPD2 | Glycerol-3-Phosphate Dehydrogenase 2 | 2.09 | N |
| MIR370 | MicroRNA 370 | 2.09 | N |
| HSPA14 | Heat Shock Protein Family A (Hsp70) Member 14 | 2.09 | N |
| CTSH | Cathepsin H | 2.09 | N |
| B3GALT4 | Beta-1,3-Galactosyltransferase 4 | 2.08 | N |
| PYY | Peptide YY | 2.08 | N |
| TMSB10 | Thymosin Beta 10 | 2.08 | N |
| GUSB | Glucuronidase Beta | 2.08 | N |
| PDZK1 | PDZ Domain Containing 1 | 2.08 | N |
| EIF2B4 | Eukaryotic Translation Initiation Factor 2B Subunit Delta | 2.08 | N |
| DHX38 | DEAH-Box Helicase 38 | 2.08 | N |
| PDE9A | Phosphodiesterase 9A | 2.08 | N |
| SLC25A1 | Solute Carrier Family 25 Member 1 | 2.07 | N |
| SLC22A4 | Solute Carrier Family 22 Member 4 | 2.07 | N |
| AZU1 | Azurocidin 1 | 2.07 | N |
| STEAP1 | STEAP Family Member 1 | 2.07 | N |
| EEF1E1 | Eukaryotic Translation Elongation Factor 1 Epsilon 1 | 2.07 | N |
| CAPS2 | Calcyphosine 2 | 2.07 | N |
| SPATA6L | Spermatogenesis Associated 6 Like | 2.07 | N |
| ARMH3 | Armadillo Like Helical Domain Containing 3 | 2.07 | N |
| MROH5 | Maestro Heat Like Repeat Family Member 5 (Gene/Pseudogene) | 2.07 | N |
| BLOC1S5-TXNDC5 | BLOC1S5-TXNDC5 Readthrough (NMD Candidate) | 2.07 | N |
| LINC00907 | Long Intergenic Non-Protein Coding RNA 907 | 2.07 | N |
| LINC02618 | Long Intergenic Non-Protein Coding RNA 2618 | 2.07 | N |
| LOC102724465 | Uncharacterized LOC102724465 | 2.07 | N |
| ACACA | Acetyl-CoA Carboxylase Alpha | 2.06 | N |
| PRSS1 | Serine Protease 1 | 2.06 | N |
| CEACAM3 | CEA Cell Adhesion Molecule 3 | 2.06 | N |
| SPARCL1 | SPARC Like 1 | 2.06 | N |
| ANO6 | Anoctamin 6 | 2.05 | N |
| IRAK4 | Interleukin 1 Receptor Associated Kinase 4 | 2.05 | N |
| P2RY4 | Pyrimidinergic Receptor P2Y4 | 2.05 | N |
| C4BPB | Complement Component 4 Binding Protein Beta | 2.05 | N |
| SLC2A3 | Solute Carrier Family 2 Member 3 | 2.05 | N |
| NAB1 | NGFI-A Binding Protein 1 | 2.05 | N |
| DMRTA1 | DMRT Like Family A1 | 2.05 | N |
| RPL29P27 | Ribosomal Protein L29 Pseudogene 27 | 2.05 | N |
| TNFSF13 | TNF Superfamily Member 13 | 2.04 | N |
| JPH3 | Junctophilin 3 | 2.04 | N |
| CCL23 | C-C Motif Chemokine Ligand 23 | 2.04 | N |
| APC | APC Regulator Of WNT Signaling Pathway | 2.04 | N |
| CAMK2G | Calcium/Calmodulin Dependent Protein Kinase II Gamma | 2.03 | N |
| HDC | Histidine Decarboxylase | 2.03 | N |
| RUNX3 | RUNX Family Transcription Factor 3 | 2.03 | N |
| PRF1 | Perforin 1 | 2.03 | N |
| RSAD2 | Radical S-Adenosyl Methionine Domain Containing 2 | 2.03 | N |
| LAMA3 | Laminin Subunit Alpha 3 | 2.03 | N |
| RELB | RELB Proto-Oncogene, NF-KB Subunit | 2.02 | N |
| HGFAC | HGF Activator | 2.02 | N |
| PLCH1 | Phospholipase C Eta 1 | 2.02 | N |
| AIMP1 | Aminoacyl TRNA Synthetase Complex Interacting Multifunctional Protein 1 | 2.01 | N |
| MICA | MHC Class I Polypeptide-Related Sequence A | 2.01 | N |
| HCG27 | HLA Complex Group 27 | 2.01 | N |
| SUMO1 | Small Ubiquitin Like Modifier 1 | 2.01 | N |
| CA10 | Carbonic Anhydrase 10 | 2.01 | N |
| BLVRB | Biliverdin Reductase B | 2.01 | N |
| MUC1 | Mucin 1, Cell Surface Associated | 2.01 | N |
| CCK | Cholecystokinin | 2.01 | N |
| HPX | Hemopexin | 2.01 | N |
| CCL24 | C-C Motif Chemokine Ligand 24 | 2.01 | N |
| LTB4R2 | Leukotriene B4 Receptor 2 | 2 | N |
| CAMTA1 | Calmodulin Binding Transcription Activator 1 | 2 | N |
| EPHB3 | EPH Receptor B3 | 2 | N |
| PPP1R12B | Protein Phosphatase 1 Regulatory Subunit 12B | 2 | N |
| EIF2S1 | Eukaryotic Translation Initiation Factor 2 Subunit Alpha | 2 | N |
| NTRK2 | Neurotrophic Receptor Tyrosine Kinase 2 | 2 | N |
| TBC1D4 | TBC1 Domain Family Member 4 | 1.99 | N |
| ALDH9A1 | Aldehyde Dehydrogenase 9 Family Member A1 | 1.99 | N |
| ANGPTL1 | Angiopoietin Like 1 | 1.99 | N |
| SIRT4 | Sirtuin 4 | 1.98 | N |
| P4HB | Prolyl 4-Hydroxylase Subunit Beta | 1.98 | N |
| SOX18 | SRY-Box Transcription Factor 18 | 1.98 | N |
| MIR29B1 | MicroRNA 29b-1 | 1.98 | N |
| HK2 | Hexokinase 2 | 1.98 | N |
| STC1 | Stanniocalcin 1 | 1.98 | N |
| PTRHD1 | Peptidyl-TRNA Hydrolase Domain Containing 1 | 1.98 | N |
| SLC10A1 | Solute Carrier Family 10 Member 1 | 1.97 | N |
| RAPGEF3 | Rap Guanine Nucleotide Exchange Factor 3 | 1.97 | N |
| MAT2B | Methionine Adenosyltransferase 2B | 1.97 | N |
| ITGA3 | Integrin Subunit Alpha 3 | 1.97 | N |
| OPRM1 | Opioid Receptor Mu 1 | 1.97 | N |
| DSPP | Dentin Sialophosphoprotein | 1.97 | N |
| TXNRD1 | Thioredoxin Reductase 1 | 1.97 | N |
| CAPG | Capping Actin Protein, Gelsolin Like | 1.96 | N |
| TYRO3 | TYRO3 Protein Tyrosine Kinase | 1.96 | N |
| PRKCZ | Protein Kinase C Zeta | 1.96 | N |
| MIR134 | MicroRNA 134 | 1.96 | N |
| FUT3 | Fucosyltransferase 3 (Lewis Blood Group) | 1.96 | N |
| SEC23IP | SEC23 Interacting Protein | 1.96 | N |
| FPR1 | Formyl Peptide Receptor 1 | 1.96 | N |
| DMRT1 | Doublesex And Mab-3 Related Transcription Factor 1 | 1.96 | N |
| CDC37 | Cell Division Cycle 37 | 1.95 | N |
| EIF3G | Eukaryotic Translation Initiation Factor 3 Subunit G | 1.95 | N |
| ZNF627 | Zinc Finger Protein 627 | 1.95 | N |
| RAVER1 | Ribonucleoprotein, PTB Binding 1 | 1.95 | N |
| ZNF844 | Zinc Finger Protein 844 | 1.95 | N |
| ZNF700 | Zinc Finger Protein 700 | 1.95 | N |
| ZNF441 | Zinc Finger Protein 441 | 1.95 | N |
| C19orf38 | Chromosome 19 Open Reading Frame 38 | 1.95 | N |
| ZNF491 | Zinc Finger Protein 491 | 1.95 | N |
| ZNF788P | Zinc Finger Family Member 788, Pseudogene | 1.95 | N |
| piR-43107-137 |  | 1.95 | N |
| lnc-SPC24-1 |  | 1.95 | N |
| LDLR-AS1-001 | | 1.95 | N |
| SMAD5 | SMAD Family Member 5 | 1.95 | N |
| ITPR3 | Inositol 1,4,5-Trisphosphate Receptor Type 3 | 1.95 | N |
| LRG1 | Leucine Rich Alpha-2-Glycoprotein 1 | 1.95 | N |
| PTCH1 | Patched 1 | 1.95 | N |
| SEMA3A | Semaphorin 3A | 1.95 | N |
| TSC2 | TSC Complex Subunit 2 | 1.94 | N |
| KCNH5 | Potassium Voltage-Gated Channel Subfamily H Member 5 | 1.94 | N |
| DNAH9 | Dynein Axonemal Heavy Chain 9 | 1.94 | N |
| GNPDA2 | Glucosamine-6-Phosphate Deaminase 2 | 1.94 | N |
| RALYL | RALY RNA Binding Protein Like | 1.94 | N |
| LHFPL1 | LHFPL Tetraspan Subfamily Member 1 | 1.94 | N |
| RPS27P1 | Ribosomal Protein S27 Pseudogene 1 | 1.94 | N |
| POM121L3P | POM121 Transmembrane Nucleoporin Like 3, Pseudogene | 1.94 | N |
| SRIP1 | Sorcin Pseudogene 1 | 1.94 | N |
| TBCAP1 | Tubulin Folding Cofactor A Pseudogene 1 | 1.94 | N |
| RPL32P12 | Ribosomal Protein L32 Pseudogene 12 | 1.94 | N |
| CYCSP14 | CYCS Pseudogene 14 | 1.94 | N |
| RPL7AP58 | Ribosomal Protein L7a Pseudogene 58 | 1.94 | N |
| ERG | ETS Transcription Factor ERG | 1.94 | N |
| CDH1 | Cadherin 1 | 1.93 | N |
| BACH1 | BTB Domain And CNC Homolog 1 | 1.93 | N |
| SHMT1 | Serine Hydroxymethyltransferase 1 | 1.92 | N |
| EBPL | EBP Like | 1.92 | N |
| CNTN5 | Contactin 5 | 1.92 | N |
| HMGA1 | High Mobility Group AT-Hook 1 | 1.92 | N |
| DLGAP1 | DLG Associated Protein 1 | 1.92 | N |
| AIDA | Axin Interactor, Dorsalization Associated | 1.91 | N |
| ENSG00000267052 | | 1.91 | N |
| CD2 | CD2 Molecule | 1.91 | N |
| ATR | ATR Serine/Threonine Kinase | 1.91 | N |
| CFB | Complement Factor B | 1.9 | N |
| XIAP | X-Linked Inhibitor Of Apoptosis | 1.9 | N |
| TOMM40 | Translocase Of Outer Mitochondrial Membrane 40 | 1.9 | N |
| PTPN3 | Protein Tyrosine Phosphatase Non-Receptor Type 3 | 1.89 | N |
| LRPAP1 | LDL Receptor Related Protein Associated Protein 1 | 1.89 | N |
| MIR497 | MicroRNA 497 | 1.88 | N |
| CPQ | Carboxypeptidase Q | 1.88 | N |
| C1QTNF5 | C1q And TNF Related 5 | 1.88 | N |
| CD209 | CD209 Molecule | 1.88 | N |
| CD84 | CD84 Molecule | 1.87 | N |
| MIR148A | MicroRNA 148a | 1.87 | N |
| CXCL3 | C-X-C Motif Chemokine Ligand 3 | 1.87 | N |
| DYM | Dymeclin | 1.87 | N |
| HDAC6 | Histone Deacetylase 6 | 1.87 | N |
| PML | Promyelocytic Leukemia | 1.87 | N |
| SCARA3 | Scavenger Receptor Class A Member 3 | 1.87 | N |
| CLTCL1 | Clathrin Heavy Chain Like 1 | 1.87 | N |
| SUMO4 | Small Ubiquitin Like Modifier 4 | 1.86 | N |
| ITGB5 | Integrin Subunit Beta 5 | 1.86 | N |
| FGF7 | Fibroblast Growth Factor 7 | 1.86 | N |
| NAPG | NSF Attachment Protein Gamma | 1.86 | N |
| PAQR5 | Progestin And AdipoQ Receptor Family Member 5 | 1.86 | N |
| ZNF717 | Zinc Finger Protein 717 | 1.86 | N |
| NRG4 | Neuregulin 4 | 1.85 | N |
| SFRP5 | Secreted Frizzled Related Protein 5 | 1.85 | N |
| lnc-APOC1-1 |  | 1.85 | N |
| TBC1D1 | TBC1 Domain Family Member 1 | 1.85 | N |
| LUM | Lumican | 1.85 | N |
| FCRL6 | Fc Receptor Like 6 | 1.84 | N |
| DLC1 | DLC1 Rho GTPase Activating Protein | 1.84 | N |
| ATG5 | Autophagy Related 5 | 1.84 | N |
| UNC13C | Unc-13 Homolog C | 1.84 | N |
| MIR103A1 | MicroRNA 103a-1 | 1.84 | N |
| PKM | Pyruvate Kinase M1/2 | 1.84 | N |
| KLRK1 | Killer Cell Lectin Like Receptor K1 | 1.84 | N |
| FTH1 | Ferritin Heavy Chain 1 | 1.83 | N |
| CCL22 | C-C Motif Chemokine Ligand 22 | 1.83 | N |
| LSAMP | Limbic System Associated Membrane Protein | 1.83 | N |
| MIR19A | MicroRNA 19a | 1.82 | N |
| TRIB2 | Tribbles Pseudokinase 2 | 1.82 | N |
| RIPK3 | Receptor Interacting Serine/Threonine Kinase 3 | 1.82 | N |
| SERPINA5 | Serpin Family A Member 5 | 1.82 | N |
| HOMER1 | Homer Scaffold Protein 1 | 1.82 | N |
| USO1 | USO1 Vesicle Transport Factor | 1.82 | N |
| BCL2L11 | BCL2 Like 11 | 1.81 | N |
| LRRTM4 | Leucine Rich Repeat Transmembrane Neuronal 4 | 1.81 | N |
| WDFY4 | WDFY Family Member 4 | 1.81 | N |
| LRRC18 | Leucine Rich Repeat Containing 18 | 1.81 | N |
| MARCKS | Myristoylated Alanine Rich Protein Kinase C Substrate | 1.81 | N |
| NPTXR | Neuronal Pentraxin Receptor | 1.81 | N |
| ACSL1 | Acyl-CoA Synthetase Long Chain Family Member 1 | 1.81 | N |
| ADH7 | Alcohol Dehydrogenase 7 (Class IV), Mu Or Sigma Polypeptide | 1.81 | N |
| VPS41 | VPS41 Subunit Of HOPS Complex | 1.81 | N |
| CORT | Cortistatin | 1.8 | N |
| GPIHBP1 | Glycosylphosphatidylinositol Anchored High Density Lipoprotein Binding Protein 1 | 1.79 | N |
| IL11 | Interleukin 11 | 1.79 | N |
| ARHGEF38 | Rho Guanine Nucleotide Exchange Factor 38 | 1.79 | N |
| PDLIM7 | PDZ And LIM Domain 7 | 1.79 | N |
| GLS2 | Glutaminase 2 | 1.79 | N |
| TANK | TRAF Family Member Associated NFKB Activator | 1.78 | N |
| CYP24A1 | Cytochrome P450 Family 24 Subfamily A Member 1 | 1.78 | N |
| MIR381 | MicroRNA 381 | 1.78 | N |
| CLEC12A | C-Type Lectin Domain Family 12 Member A | 1.77 | N |
| CEBPB | CCAAT Enhancer Binding Protein Beta | 1.77 | N |
| MIR212 | MicroRNA 212 | 1.76 | N |
| SERPINA4 | Serpin Family A Member 4 | 1.76 | N |
| HAS2 | Hyaluronan Synthase 2 | 1.76 | N |
| LACTB | Lactamase Beta | 1.76 | N |
| EPHX1 | Epoxide Hydrolase 1 | 1.75 | N |
| OXA1L | OXA1L Mitochondrial Inner Membrane Protein | 1.75 | N |
| NDP | Norrin Cystine Knot Growth Factor NDP | 1.75 | N |
| NCOR2 | Nuclear Receptor Corepressor 2 | 1.75 | N |
| STIM1 | Stromal Interaction Molecule 1 | 1.75 | N |
| EDEM2 | ER Degradation Enhancing Alpha-Mannosidase Like Protein 2 | 1.75 | N |
| CRLF2 | Cytokine Receptor Like Factor 2 | 1.74 | N |
| GOSR2 | Golgi SNAP Receptor Complex Member 2 | 1.74 | N |
| MRC1 | Mannose Receptor C-Type 1 | 1.74 | N |
| FGF9 | Fibroblast Growth Factor 9 | 1.74 | N |
| CDH7 | Cadherin 7 | 1.74 | N |
| PRDX6 | Peroxiredoxin 6 | 1.74 | N |
| DIO2 | Iodothyronine Deiodinase 2 | 1.74 | N |
| VAMP8 | Vesicle Associated Membrane Protein 8 | 1.74 | N |
| CD27 | CD27 Molecule | 1.73 | N |
| EZH2 | Enhancer Of Zeste 2 Polycomb Repressive Complex 2 Subunit | 1.73 | N |
| TIAM1 | TIAM Rac1 Associated GEF 1 | 1.73 | N |
| ENOSF1 | Enolase Superfamily Member 1 | 1.73 | N |
| ABI2 | Abl Interactor 2 | 1.73 | N |
| SEC11B | SEC11 Homolog B, Signal Peptidase Complex Subunit (Pseudogene) | 1.73 | N |
| GPR35 | G Protein-Coupled Receptor 35 | 1.73 | N |
| MIR215 | MicroRNA 215 | 1.73 | N |
| MDK | Midkine | 1.73 | N |
| TRPM1 | Transient Receptor Potential Cation Channel Subfamily M Member 1 | 1.73 | N |
| DPRXP7 | Divergent-Paired Related Homeobox Pseudogene 7 | 1.73 | N |
| HMGCL | 3-Hydroxy-3-Methylglutaryl-CoA Lyase | 1.72 | N |
| PCDH9 | Protocadherin 9 | 1.72 | N |
| PTPRD | Protein Tyrosine Phosphatase Receptor Type D | 1.72 | N |
| HBP1 | HMG-Box Transcription Factor 1 | 1.72 | N |
| LINC02577 | Long Intergenic Non-Protein Coding RNA 2577 | 1.72 | N |
| ENSG00000243797 | | 1.72 | N |
| lnc-PIK3CG-6 |  | 1.72 | N |
| piR-55948-110 |  | 1.72 | N |
| MIR495 | MicroRNA 495 | 1.72 | N |
| SNAI1 | Snail Family Transcriptional Repressor 1 | 1.71 | N |
| CD1C | CD1c Molecule | 1.71 | N |
| MED6 | Mediator Complex Subunit 6 | 1.71 | N |
| PTN | Pleiotrophin | 1.71 | N |
| NPS | Neuropeptide S | 1.71 | N |
| LDAH | Lipid Droplet Associated Hydrolase | 1.71 | N |
| MAPK13 | Mitogen-Activated Protein Kinase 13 | 1.7 | N |
| ROBO2 | Roundabout Guidance Receptor 2 | 1.7 | N |
| EPB41L3 | Erythrocyte Membrane Protein Band 4.1 Like 3 | 1.7 | N |
| GLRA3 | Glycine Receptor Alpha 3 | 1.7 | N |
| PJA1 | Praja Ring Finger Ubiquitin Ligase 1 | 1.7 | N |
| DIP2A | Disco Interacting Protein 2 Homolog A | 1.7 | N |
| OPN3 | Opsin 3 | 1.7 | N |
| BFSP1 | Beaded Filament Structural Protein 1 | 1.7 | N |
| AKAP7 | A-Kinase Anchoring Protein 7 | 1.7 | N |
| FMNL2 | Formin Like 2 | 1.7 | N |
| ASCC3 | Activating Signal Cointegrator 1 Complex Subunit 3 | 1.7 | N |
| TSPAN6 | Tetraspanin 6 | 1.7 | N |
| TOX3 | TOX High Mobility Group Box Family Member 3 | 1.7 | N |
| PPFIA2 | PTPRF Interacting Protein Alpha 2 | 1.7 | N |
| RHOBTB3 | Rho Related BTB Domain Containing 3 | 1.7 | N |
| NETO1 | Neuropilin And Tolloid Like 1 | 1.7 | N |
| FOXB1 | Forkhead Box B1 | 1.7 | N |
| ARHGAP20 | Rho GTPase Activating Protein 20 | 1.7 | N |
| MAGOHB | Mago Homolog B, Exon Junction Complex Subunit | 1.7 | N |
| ENOX1 | Ecto-NOX Disulfide-Thiol Exchanger 1 | 1.7 | N |
| DCBLD1 | Discoidin, CUB And LCCL Domain Containing 1 | 1.7 | N |
| VEPH1 | Ventricular Zone Expressed PH Domain Containing 1 | 1.7 | N |
| CPEB2 | Cytoplasmic Polyadenylation Element Binding Protein 2 | 1.7 | N |
| MNS1 | Meiosis Specific Nuclear Structural 1 | 1.7 | N |
| ZNF326 | Zinc Finger Protein 326 | 1.7 | N |
| CWC22 | CWC22 Spliceosome Associated Protein Homolog | 1.7 | N |
| RNF157 | Ring Finger Protein 157 | 1.7 | N |
| DTWD1 | DTW Domain Containing 1 | 1.7 | N |
| KRTAP11-1 | Keratin Associated Protein 11-1 | 1.7 | N |
| DAOA | D-Amino Acid Oxidase Activator | 1.7 | N |
| C8orf48 | Chromosome 8 Open Reading Frame 48 | 1.7 | N |
| EBLN1 | Endogenous Bornavirus Like Nucleoprotein 1 | 1.7 | N |
| UNGP1 | Uracil-DNA Glycosylase Pseudogene 1 | 1.7 | N |
| RPS26P57 | Ribosomal Protein S26 Pseudogene 57 | 1.7 | N |
| EIF3FP3 | Eukaryotic Translation Initiation Factor 3 Subunit F Pseudogene 3 | 1.7 | N |
| RPL9P21 | Ribosomal Protein L9 Pseudogene 21 | 1.7 | N |
| RPL35AP22 | Ribosomal Protein L35a Pseudogene 22 | 1.7 | N |
| RPL21P42 | Ribosomal Protein L21 Pseudogene 42 | 1.7 | N |
| RPL28P3 | Ribosomal Protein L28 Pseudogene 3 | 1.7 | N |
| RPS27AP2 | Ribosomal Protein S27a Pseudogene 2 | 1.7 | N |
| CYCSP42 | CYCS Pseudogene 42 | 1.7 | N |
| RPL7AP57 | Ribosomal Protein L7a Pseudogene 57 | 1.7 | N |
| GAPDHP28 | Glyceraldehyde 3 Phosphate Dehydrogenase Pseudogene 28 | 1.7 | N |
| TMEM248P1 | Transmembrane Protein 248 Pseudogene 1 | 1.7 | N |
| SUCLA2P2 | SUCLA2 Pseudogene 2 | 1.7 | N |
| RPL5P19 | Ribosomal Protein L5 Pseudogene 19 | 1.7 | N |
| RPL29P29 | Ribosomal Protein L29 Pseudogene 29 | 1.7 | N |
| RPS3AP9 | RPS3A Pseudogene 9 | 1.7 | N |
| OSBPL9P2 | Oxysterol Binding Protein Like 9 Pseudogene 2 | 1.7 | N |
| MIR25 | MicroRNA 25 | 1.7 | N |
| HLA-DRA | Major Histocompatibility Complex, Class II, DR Alpha | 1.69 | N |
| G6PC2 | Glucose-6-Phosphatase Catalytic Subunit 2 | 1.69 | N |
| SLPI | Secretory Leukocyte Peptidase Inhibitor | 1.69 | N |
| SIGLEC1 | Sialic Acid Binding Ig Like Lectin 1 | 1.68 | N |
| IL4R | Interleukin 4 Receptor | 1.68 | N |
| DAPK1 | Death Associated Protein Kinase 1 | 1.68 | N |
| TTPA | Alpha Tocopherol Transfer Protein | 1.68 | N |
| MARS1 | Methionyl-TRNA Synthetase 1 | 1.68 | N |
| SLC18A1 | Solute Carrier Family 18 Member A1 | 1.68 | N |
| LINC-ROR | Long Intergenic Non-Protein Coding RNA, Regulator Of Reprogramming | 1.67 | N |
| UHRF1BP1 | UHRF1 Binding Protein 1 | 1.67 | N |
| PIM1 | Pim-1 Proto-Oncogene, Serine/Threonine Kinase | 1.67 | N |
| FLYWCH1 | FLYWCH-Type Zinc Finger 1 | 1.66 | N |
| SLC17A4 | Solute Carrier Family 17 Member 4 | 1.65 | N |
| ATP6V0A2 | ATPase H+ Transporting V0 Subunit A2 | 1.65 | N |
| GSS | Glutathione Synthetase | 1.65 | N |
| CDK1 | Cyclin Dependent Kinase 1 | 1.65 | N |
| MIRLET7A1 | MicroRNA Let-7a-1 | 1.65 | N |
| EGR3 | Early Growth Response 3 | 1.65 | N |
| LECT2 | Leukocyte Cell Derived Chemotaxin 2 | 1.65 | N |
| CADPS | Calcium Dependent Secretion Activator | 1.65 | N |
| SLC23A2 | Solute Carrier Family 23 Member 2 | 1.65 | N |
| MAP2K3 | Mitogen-Activated Protein Kinase Kinase 3 | 1.65 | N |
| NDUFB8 | NADH:Ubiquinone Oxidoreductase Subunit B8 | 1.65 | N |
| LEPROT | Leptin Receptor Overlapping Transcript | 1.64 | N |
| PRPF8 | Pre-MRNA Processing Factor 8 | 1.64 | N |
| DPP9 | Dipeptidyl Peptidase 9 | 1.64 | N |
| ST6GAL1 | ST6 Beta-Galactoside Alpha-2,6-Sialyltransferase 1 | 1.63 | N |
| CTSF | Cathepsin F | 1.63 | N |
| MIR340 | MicroRNA 340 | 1.63 | N |
| MIR181B1 | MicroRNA 181b-1 | 1.63 | N |
| WWOX | WW Domain Containing Oxidoreductase | 1.63 | N |
| G3BP1 | G3BP Stress Granule Assembly Factor 1 | 1.63 | N |
| TUBA1B | Tubulin Alpha 1b | 1.63 | N |
| GPLD1 | Glycosylphosphatidylinositol Specific Phospholipase D1 | 1.63 | N |
| MAP3K1 | Mitogen-Activated Protein Kinase Kinase Kinase 1 | 1.62 | N |
| CYP2R1 | Cytochrome P450 Family 2 Subfamily R Member 1 | 1.62 | N |
| MIR100 | MicroRNA 100 | 1.62 | N |
| IL1RAP | Interleukin 1 Receptor Accessory Protein | 1.62 | N |
| MIR502 | MicroRNA 502 | 1.62 | N |
| INPP5D | Inositol Polyphosphate-5-Phosphatase D | 1.61 | N |
| SMPD2 | Sphingomyelin Phosphodiesterase 2 | 1.61 | N |
| TM4SF5 | Transmembrane 4 L Six Family Member 5 | 1.61 | N |
| SPRY4 | Sprouty RTK Signaling Antagonist 4 | 1.61 | N |
| CCL15 | C-C Motif Chemokine Ligand 15 | 1.6 | N |
| IL12RB1 | Interleukin 12 Receptor Subunit Beta 1 | 1.6 | N |
| MIR10B | MicroRNA 10b | 1.6 | N |
| DAGLB | Diacylglycerol Lipase Beta | 1.6 | N |
| IL18BP | Interleukin 18 Binding Protein | 1.6 | N |
| HTR4 | 5-Hydroxytryptamine Receptor 4 | 1.59 | N |
| SPTB | Spectrin Beta, Erythrocytic | 1.59 | N |
| CORO1B | Coronin 1B | 1.59 | N |
| ARHGEF2 | Rho/Rac Guanine Nucleotide Exchange Factor 2 | 1.59 | N |
| MTNR1B | Melatonin Receptor 1B | 1.59 | N |
| MIR23B | MicroRNA 23b | 1.59 | N |
| CAMLG | Calcium Modulating Ligand | 1.59 | N |
| TONSL | Tonsoku Like, DNA Repair Protein | 1.59 | N |
| DSCR10 | Down Syndrome Critical Region 10 | 1.59 | N |
| ENSG00000269918 | | 1.59 | N |
| MAATS1 | MYCBP Associated And Testis Expressed 1 | 1.59 | N |
| HDAC3 | Histone Deacetylase 3 | 1.58 | N |
| MCL1 | MCL1 Apoptosis Regulator, BCL2 Family Member | 1.58 | N |
| LOC100507053 | Uncharacterized LOC100507053 | 1.58 | N |
| RPTOR | Regulatory Associated Protein Of MTOR Complex 1 | 1.58 | N |
| CLDN5 | Claudin 5 | 1.58 | N |
| NCOR1 | Nuclear Receptor Corepressor 1 | 1.58 | N |
| SLC17A1 | Solute Carrier Family 17 Member 1 | 1.58 | N |
| GSC2 | Goosecoid Homeobox 2 | 1.58 | N |
| WNT2 | Wnt Family Member 2 | 1.57 | N |
| MIR98 | MicroRNA 98 | 1.57 | N |
| RAC2 | Rac Family Small GTPase 2 | 1.56 | N |
| PKD2L1 | Polycystin 2 Like 1, Transient Receptor Potential Cation Channel | 1.56 | N |
| SKP1 | S-Phase Kinase Associated Protein 1 | 1.56 | N |
| LRP12 | LDL Receptor Related Protein 12 | 1.56 | N |
| GSTA1 | Glutathione S-Transferase Alpha 1 | 1.55 | N |
| CHRNA5 | Cholinergic Receptor Nicotinic Alpha 5 Subunit | 1.55 | N |
| PTPRF | Protein Tyrosine Phosphatase Receptor Type F | 1.55 | N |
| SSTR2 | Somatostatin Receptor 2 | 1.55 | N |
| MFN2 | Mitofusin 2 | 1.55 | N |
| CFD | Complement Factor D | 1.55 | N |
| GYPA | Glycophorin A (MNS Blood Group) | 1.55 | N |
| TRAF1 | TNF Receptor Associated Factor 1 | 1.55 | N |
| CAP1 | Cyclase Associated Actin Cytoskeleton Regulatory Protein 1 | 1.55 | N |
| DIABLO | Diablo IAP-Binding Mitochondrial Protein | 1.55 | N |
| SLC22A11 | Solute Carrier Family 22 Member 11 | 1.54 | N |
| CLEC16A | C-Type Lectin Domain Containing 16A | 1.54 | N |
| SIK2 | Salt Inducible Kinase 2 | 1.54 | N |
| TUBB4A | Tubulin Beta 4A Class IVa | 1.54 | N |
| PCMT1 | Protein-L-Isoaspartate (D-Aspartate) O-Methyltransferase | 1.54 | N |
| VAV2 | Vav Guanine Nucleotide Exchange Factor 2 | 1.54 | N |
| NINJ2 | Ninjurin 2 | 1.54 | N |
| MIR146B | MicroRNA 146b | 1.54 | N |
| MRVI1 | Murine Retrovirus Integration Site 1 Homolog | 1.53 | N |
| RHO | Rhodopsin | 1.53 | N |
| ATF3 | Activating Transcription Factor 3 | 1.53 | N |
| NPY4R | Neuropeptide Y Receptor Y4 | 1.53 | N |
| ARNT | Aryl Hydrocarbon Receptor Nuclear Translocator | 1.53 | N |
| AIM2 | Absent In Melanoma 2 | 1.52 | N |
| MIRLET7G | MicroRNA Let-7g | 1.52 | N |
| FCN2 | Ficolin 2 | 1.52 | N |
| MGLL | Monoglyceride Lipase | 1.52 | N |
| AKR1A1 | Aldo-Keto Reductase Family 1 Member A1 | 1.52 | N |
| NGEF | Neuronal Guanine Nucleotide Exchange Factor | 1.52 | N |
| GPC5 | Glypican 5 | 1.52 | N |
| FCRL3 | Fc Receptor Like 3 | 1.52 | N |
| COPS5 | COP9 Signalosome Subunit 5 | 1.52 | N |
| SCUBE1 | Signal Peptide, CUB Domain And EGF Like Domain Containing 1 | 1.52 | N |
| SYK | Spleen Associated Tyrosine Kinase | 1.52 | N |
| AS3MT | Arsenite Methyltransferase | 1.52 | N |
| CYP20A1 | Cytochrome P450 Family 20 Subfamily A Member 1 | 1.51 | N |
| SRGN | Serglycin | 1.51 | N |
| ATP1A1 | ATPase Na+/K+ Transporting Subunit Alpha 1 | 1.51 | N |
| PTPN6 | Protein Tyrosine Phosphatase Non-Receptor Type 6 | 1.51 | N |
| EIF2AK3 | Eukaryotic Translation Initiation Factor 2 Alpha Kinase 3 | 1.5 | N |
| MIR205 | MicroRNA 205 | 1.5 | N |
| RRM2 | Ribonucleotide Reductase Regulatory Subunit M2 | 1.5 | N |
| CHRM3 | Cholinergic Receptor Muscarinic 3 | 1.5 | N |
| DSTN | Destrin, Actin Depolymerizing Factor | 1.5 | N |
| CD247 | CD247 Molecule | 1.5 | N |
| RARA | Retinoic Acid Receptor Alpha | 1.49 | N |
| IL21 | Interleukin 21 | 1.49 | N |
| PFN1 | Profilin 1 | 1.49 | N |
| SSH1 | Slingshot Protein Phosphatase 1 | 1.49 | N |
| CYP26B1 | Cytochrome P450 Family 26 Subfamily B Member 1 | 1.49 | N |
| RGS14 | Regulator Of G Protein Signaling 14 | 1.48 | N |
| MYH7B | Myosin Heavy Chain 7B | 1.48 | N |
| OXTR | Oxytocin Receptor | 1.48 | N |
| UIMC1 | Ubiquitin Interaction Motif Containing 1 | 1.48 | N |
| IRGM | Immunity Related GTPase M | 1.48 | N |
| RNF111 | Ring Finger Protein 111 | 1.48 | N |
| AGTRAP | Angiotensin II Receptor Associated Protein | 1.48 | N |
| RIPK2 | Receptor Interacting Serine/Threonine Kinase 2 | 1.47 | N |
| RCN3 | Reticulocalbin 3 | 1.47 | N |
| PF4V1 | Platelet Factor 4 Variant 1 | 1.47 | N |
| GADD45A | Growth Arrest And DNA Damage Inducible Alpha | 1.47 | N |
| TNFAIP6 | TNF Alpha Induced Protein 6 | 1.46 | N |
| MIR624 | MicroRNA 624 | 1.46 | N |
| MIR197 | MicroRNA 197 | 1.46 | N |
| PGLYRP1 | Peptidoglycan Recognition Protein 1 | 1.46 | N |
| RORA | RAR Related Orphan Receptor A | 1.46 | N |
| NBL1 | NBL1, DAN Family BMP Antagonist | 1.45 | N |
| PCDH15 | Protocadherin Related 15 | 1.45 | N |
| HACD4 | 3-Hydroxyacyl-CoA Dehydratase 4 | 1.45 | N |
| HSPE1 | Heat Shock Protein Family E (Hsp10) Member 1 | 1.45 | N |
| NDUFA9 | NADH:Ubiquinone Oxidoreductase Subunit A9 | 1.44 | N |
| OGDH | Oxoglutarate Dehydrogenase | 1.44 | N |
| APOBEC1 | Apolipoprotein B MRNA Editing Enzyme Catalytic Subunit 1 | 1.44 | N |
| PRDM1 | PR/SET Domain 1 | 1.43 | N |
| FLNB | Filamin B | 1.43 | N |
| MIR4675 | MicroRNA 4675 | 1.43 | N |
| piR-56133-114 |  | 1.43 | N |
| ENSG00000280087 | | 1.43 | N |
| HNF1B | HNF1 Homeobox B | 1.43 | N |
| RAET1E | Retinoic Acid Early Transcript 1E | 1.43 | N |
| UGT1A12P | UDP Glucuronosyltransferase Family 1 Member A12, Pseudogene | 1.43 | N |
| PSMC6 | Proteasome 26S Subunit, ATPase 6 | 1.43 | N |
| UTRN | Utrophin | 1.42 | N |
| PTPRA | Protein Tyrosine Phosphatase Receptor Type A | 1.42 | N |
| CYP46A1 | Cytochrome P450 Family 46 Subfamily A Member 1 | 1.42 | N |
| RFC4 | Replication Factor C Subunit 4 | 1.42 | N |
| DHFR | Dihydrofolate Reductase | 1.42 | N |
| STC2 | Stanniocalcin 2 | 1.41 | N |
| HSP90AB1 | Heat Shock Protein 90 Alpha Family Class B Member 1 | 1.41 | N |
| ABCA8 | ATP Binding Cassette Subfamily A Member 8 | 1.4 | N |
| TREML4 | Triggering Receptor Expressed On Myeloid Cells Like 4 | 1.4 | N |
| NAXE | NAD(P)HX Epimerase | 1.4 | N |
| CDK5 | Cyclin Dependent Kinase 5 | 1.39 | N |
| AMFR | Autocrine Motility Factor Receptor | 1.39 | N |
| HLA-DRB5 | Major Histocompatibility Complex, Class II, DR Beta 5 | 1.39 | N |
| BCL3 | BCL3 Transcription Coactivator | 1.39 | N |
| HNRNPC | Heterogeneous Nuclear Ribonucleoprotein C | 1.38 | N |
| ZNF213-AS1 | ZNF213 Antisense RNA 1 (Head To Head) | 1.38 | N |
| SLC40A1 | Solute Carrier Family 40 Member 1 | 1.37 | N |
| SEMA3F | Semaphorin 3F | 1.37 | N |
| MIR15B | MicroRNA 15b | 1.37 | N |
| GAL | Galanin And GMAP Prepropeptide | 1.37 | N |
| SRD5A1 | Steroid 5 Alpha-Reductase 1 | 1.37 | N |
| CNTLN | Centlein | 1.37 | N |
| MAP3K7 | Mitogen-Activated Protein Kinase Kinase Kinase 7 | 1.36 | N |
| SEMA3C | Semaphorin 3C | 1.36 | N |
| HAVCR2 | Hepatitis A Virus Cellular Receptor 2 | 1.36 | N |
| LCT | Lactase | 1.35 | N |
| MIR132 | MicroRNA 132 | 1.35 | N |
| AGL | Amylo-Alpha-1, 6-Glucosidase, 4-Alpha-Glucanotransferase | 1.35 | N |
| COL4A4 | Collagen Type IV Alpha 4 Chain | 1.35 | N |
| MIR135B | MicroRNA 135b | 1.35 | N |
| COL6A1 | Collagen Type VI Alpha 1 Chain | 1.35 | N |
| MIR106B | MicroRNA 106b | 1.35 | N |
| ZHX2 | Zinc Fingers And Homeoboxes 2 | 1.34 | N |
| LMF1 | Lipase Maturation Factor 1 | 1.34 | N |
| TIMD4 | T Cell Immunoglobulin And Mucin Domain Containing 4 | 1.34 | N |
| CABIN1 | Calcineurin Binding Protein 1 | 1.34 | N |
| RPL7 | Ribosomal Protein L7 | 1.34 | N |
| MAZ | MYC Associated Zinc Finger Protein | 1.33 | N |
| SLC20A1 | Solute Carrier Family 20 Member 1 | 1.33 | N |
| LOC106728418 | LEP 5' Regulatory Region | 1.32 | N |
| VAMP3 | Vesicle Associated Membrane Protein 3 | 1.32 | N |
| SORBS3 | Sorbin And SH3 Domain Containing 3 | 1.32 | N |
| MIR362 | MicroRNA 362 | 1.31 | N |
| TCF7 | Transcription Factor 7 | 1.31 | N |
| ADAMTS8 | ADAM Metallopeptidase With Thrombospondin Type 1 Motif 8 | 1.31 | N |
| ACAD8 | Acyl-CoA Dehydrogenase Family Member 8 | 1.3 | N |
| SERPINA10 | Serpin Family A Member 10 | 1.3 | N |
| CBL | Cbl Proto-Oncogene | 1.3 | N |
| COL20A1 | Collagen Type XX Alpha 1 Chain | 1.3 | N |
| PRMT3 | Protein Arginine Methyltransferase 3 | 1.29 | N |
| IER3 | Immediate Early Response 3 | 1.29 | N |
| ORMDL3 | ORMDL Sphingolipid Biosynthesis Regulator 3 | 1.28 | N |
| CREB3 | CAMP Responsive Element Binding Protein 3 | 1.28 | N |
| GPR55 | G Protein-Coupled Receptor 55 | 1.28 | N |
| TRIM24 | Tripartite Motif Containing 24 | 1.28 | N |
| TRPA1 | Transient Receptor Potential Cation Channel Subfamily A Member 1 | 1.27 | N |
| SLC1A4 | Solute Carrier Family 1 Member 4 | 1.27 | N |
| MYO1E | Myosin IE | 1.27 | N |
| ALCAM | Activated Leukocyte Cell Adhesion Molecule | 1.27 | N |
| SEMA4D | Semaphorin 4D | 1.26 | N |
| BAK1 | BCL2 Antagonist/Killer 1 | 1.26 | N |
| NEAT1 | Nuclear Paraspeckle Assembly Transcript 1 | 1.26 | N |
| PBK | PDZ Binding Kinase | 1.25 | N |
| E2F8 | E2F Transcription Factor 8 | 1.25 | N |
| COL15A1 | Collagen Type XV Alpha 1 Chain | 1.25 | N |
| PIAS4 | Protein Inhibitor Of Activated STAT 4 | 1.25 | N |
| UNC5B | Unc-5 Netrin Receptor B | 1.24 | N |
| PSMD9 | Proteasome 26S Subunit, Non-ATPase 9 | 1.24 | N |
| ERN1 | Endoplasmic Reticulum To Nucleus Signaling 1 | 1.24 | N |
| HBS1L | HBS1 Like Translational GTPase | 1.24 | N |
| MIR28 | MicroRNA 28 | 1.24 | N |
| KDM5B | Lysine Demethylase 5B | 1.24 | N |
| MIR16-1 | MicroRNA 16-1 | 1.23 | N |
| NAT8 | N-Acetyltransferase 8 (Putative) | 1.23 | N |
| LPIN1 | Lipin 1 | 1.23 | N |
| MSBP2 | Minisatellite Binding Protein 2 | 1.22 | N |
| MIR217 | MicroRNA 217 | 1.21 | N |
| POLR2D | RNA Polymerase II Subunit D | 1.21 | N |
| C1QBP | Complement C1q Binding Protein | 1.21 | N |
| FGF14 | Fibroblast Growth Factor 14 | 1.21 | N |
| INTS12 | Integrator Complex Subunit 12 | 1.21 | N |
| GIMAP5 | GTPase, IMAP Family Member 5 | 1.21 | N |
| MIR130B | MicroRNA 130b | 1.21 | N |
| CCND2 | Cyclin D2 | 1.21 | N |
| MIR30D | MicroRNA 30d | 1.2 | N |
| MAP1LC3B | Microtubule Associated Protein 1 Light Chain 3 Beta | 1.2 | N |
| LILRB1 | Leukocyte Immunoglobulin Like Receptor B1 | 1.2 | N |
| WDR1 | WD Repeat Domain 1 | 1.2 | N |
| RETNLB | Resistin Like Beta | 1.19 | N |
| FCGR1A | Fc Fragment Of IgG Receptor Ia | 1.19 | N |
| CAPN1 | Calpain 1 | 1.19 | N |
| MTHFD2 | Methylenetetrahydrofolate Dehydrogenase (NADP+ Dependent) 2, Methenyltetrahydrofolate Cyclohydrolase | 1.19 | N |
| FERMT3 | Fermitin Family Member 3 | 1.19 | N |
| CASP7 | Caspase 7 | 1.19 | N |
| PPP1R1A | Protein Phosphatase 1 Regulatory Inhibitor Subunit 1A | 1.19 | N |
| PLD2 | Phospholipase D2 | 1.19 | N |
| LRIG1 | Leucine Rich Repeats And Immunoglobulin Like Domains 1 | 1.19 | N |
| RPL21P41 | Ribosomal Protein L21 Pseudogene 41 | 1.19 | N |
| CYP4V2 | Cytochrome P450 Family 4 Subfamily V Member 2 | 1.18 | N |
| TPSAB1 | Tryptase Alpha/Beta 1 | 1.18 | N |
| PRKD2 | Protein Kinase D2 | 1.18 | N |
| TNKS | Tankyrase | 1.18 | N |
| CD38 | CD38 Molecule | 1.18 | N |
| PDE1C | Phosphodiesterase 1C | 1.18 | N |
| GRM8 | Glutamate Metabotropic Receptor 8 | 1.18 | N |
| MAP3K14 | Mitogen-Activated Protein Kinase Kinase Kinase 14 | 1.17 | N |
| RHBDF2 | Rhomboid 5 Homolog 2 | 1.17 | N |
| MPP7 | Membrane Palmitoylated Protein 7 | 1.16 | N |
| MIR216A | MicroRNA 216a | 1.16 | N |
| SURF1 | SURF1 Cytochrome C Oxidase Assembly Factor | 1.16 | N |
| PGK1 | Phosphoglycerate Kinase 1 | 1.16 | N |
| AAMP | Angio Associated Migratory Cell Protein | 1.16 | N |
| ADAM9 | ADAM Metallopeptidase Domain 9 | 1.16 | N |
| ADAM12 | ADAM Metallopeptidase Domain 12 | 1.15 | N |
| CCNB2 | Cyclin B2 | 1.15 | N |
| AQP9 | Aquaporin 9 | 1.15 | N |
| PPM1K | Protein Phosphatase, Mg2+/Mn2+ Dependent 1K | 1.15 | N |
| SPRYD4 | SPRY Domain Containing 4 | 1.15 | N |
| HERC6 | HECT And RLD Domain Containing E3 Ubiquitin Protein Ligase Family Member 6 | 1.15 | N |
| GLTPD2 | Glycolipid Transfer Protein Domain Containing 2 | 1.15 | N |
| PFN3 | Profilin 3 | 1.15 | N |
| CKLF | Chemokine Like Factor | 1.15 | N |
| UGT1A6 | UDP Glucuronosyltransferase Family 1 Member A6 | 1.14 | N |
| STAB2 | Stabilin 2 | 1.14 | N |
| DEFB128 | Defensin Beta 128 | 1.14 | N |
| SLC5A1 | Solute Carrier Family 5 Member 1 | 1.14 | N |
| MIR224 | MicroRNA 224 | 1.13 | N |
| MIR490 | MicroRNA 490 | 1.13 | N |
| KLF10 | Kruppel Like Factor 10 | 1.13 | N |
| CSGALNACT2 | Chondroitin Sulfate N-Acetylgalactosaminyltransferase 2 | 1.13 | N |
| NPY5R | Neuropeptide Y Receptor Y5 | 1.13 | N |
| OSBPL8 | Oxysterol Binding Protein Like 8 | 1.13 | N |
| TNPO1 | Transportin 1 | 1.12 | N |
| ADCY1 | Adenylate Cyclase 1 | 1.12 | N |
| IL18RAP | Interleukin 18 Receptor Accessory Protein | 1.12 | N |
| HHIP | Hedgehog Interacting Protein | 1.11 | N |
| GPR26 | G Protein-Coupled Receptor 26 | 1.11 | N |
| PEX3 | Peroxisomal Biogenesis Factor 3 | 1.11 | N |
| APOBR | Apolipoprotein B Receptor | 1.11 | N |
| MMP26 | Matrix Metallopeptidase 26 | 1.1 | N |
| ATP10D | ATPase Phospholipid Transporting 10D (Putative) | 1.1 | N |
| SIRPA | Signal Regulatory Protein Alpha | 1.1 | N |
| SLC11A1 | Solute Carrier Family 11 Member 1 | 1.09 | N |
| PRMT7 | Protein Arginine Methyltransferase 7 | 1.09 | N |
| IL18R1 | Interleukin 18 Receptor 1 | 1.09 | N |
| TUBB | Tubulin Beta Class I | 1.09 | N |
| SKP2 | S-Phase Kinase Associated Protein 2 | 1.09 | N |
| HLA-DQA2 | Major Histocompatibility Complex, Class II, DQ Alpha 2 | 1.09 | N |
| EIF4E | Eukaryotic Translation Initiation Factor 4E | 1.08 | N |
| DAPK3 | Death Associated Protein Kinase 3 | 1.08 | N |
| IFN1@ | Interferon, Type 1, Cluster | 1.08 | N |
| NME4 | NME/NM23 Nucleoside Diphosphate Kinase 4 | 1.08 | N |
| PIP5KL1 | Phosphatidylinositol-4-Phosphate 5-Kinase Like 1 | 1.07 | N |
| SLC2A2 | Solute Carrier Family 2 Member 2 | 1.07 | N |
| FAM126A | Family With Sequence Similarity 126 Member A | 1.07 | N |
| CDK9 | Cyclin Dependent Kinase 9 | 1.07 | N |
| AKR1C4 | Aldo-Keto Reductase Family 1 Member C4 | 1.07 | N |
| SLC16A3 | Solute Carrier Family 16 Member 3 | 1.07 | N |
| DMWD | DM1 Locus, WD Repeat Containing | 1.07 | N |
| UGT1A9 | UDP Glucuronosyltransferase Family 1 Member A9 | 1.07 | N |
| UGT1A4 | UDP Glucuronosyltransferase Family 1 Member A4 | 1.07 | N |
| UGT1A10 | UDP Glucuronosyltransferase Family 1 Member A10 | 1.07 | N |
| UGT1A3 | UDP Glucuronosyltransferase Family 1 Member A3 | 1.07 | N |
| UGT1A7 | UDP Glucuronosyltransferase Family 1 Member A7 | 1.07 | N |
| UGT1A8 | UDP Glucuronosyltransferase Family 1 Member A8 | 1.07 | N |
| UGT1A5 | UDP Glucuronosyltransferase Family 1 Member A5 | 1.07 | N |
| PLA2G15 | Phospholipase A2 Group XV | 1.06 | N |
| DUSP19 | Dual Specificity Phosphatase 19 | 1.06 | N |
| HSDL2 | Hydroxysteroid Dehydrogenase Like 2 | 1.06 | N |
| RPLP0 | Ribosomal Protein Lateral Stalk Subunit P0 | 1.06 | N |
| SURF4 | Surfeit 4 | 1.06 | N |
| LMAN2 | Lectin, Mannose Binding 2 | 1.06 | N |
| SURF2 | Surfeit 2 | 1.06 | N |
| PRELID1 | PRELI Domain Containing 1 | 1.06 | N |
| MXD3 | MAX Dimerization Protein 3 | 1.06 | N |
| PRR7 | Proline Rich 7, Synaptic | 1.06 | N |
| F11-AS1 | F11 Antisense RNA 1 | 1.06 | N |
| PRICKLE1 | Prickle Planar Cell Polarity Protein 1 | 1.05 | N |
| LRP1B | LDL Receptor Related Protein 1B | 1.05 | N |
| NDUFS2 | NADH:Ubiquinone Oxidoreductase Core Subunit S2 | 1.05 | N |
| MAPKAPK2 | MAPK Activated Protein Kinase 2 | 1.05 | N |
| PLCG2 | Phospholipase C Gamma 2 | 1.04 | N |
| ZFYVE9 | Zinc Finger FYVE-Type Containing 9 | 1.04 | N |
| DPP8 | Dipeptidyl Peptidase 8 | 1.04 | N |
| PBRM1 | Polybromo 1 | 1.04 | N |
| IFIT3 | Interferon Induced Protein With Tetratricopeptide Repeats 3 | 1.04 | N |
| FAM167A | Family With Sequence Similarity 167 Member A | 1.03 | N |
| CCN4 | Cellular Communication Network Factor 4 | 1.03 | N |
| GNA11 | G Protein Subunit Alpha 11 | 1.03 | N |
| MIR141 | MicroRNA 141 | 1.03 | N |
| CCNE1 | Cyclin E1 | 1.02 | N |
| GTF2E2 | General Transcription Factor IIE Subunit 2 | 1.02 | N |
| SCD5 | Stearoyl-CoA Desaturase 5 | 1.02 | N |
| UGCG | UDP-Glucose Ceramide Glucosyltransferase | 1.02 | N |
| PODN | Podocan | 1.02 | N |
| GPC1 | Glypican 1 | 1.01 | N |
| ENPP2 | Ectonucleotide Pyrophosphatase/Phosphodiesterase 2 | 1.01 | N |
| MAST4 | Microtubule Associated Serine/Threonine Kinase Family Member 4 | 1.01 | N |
| ATIC | 5-Aminoimidazole-4-Carboxamide Ribonucleotide Formyltransferase/IMP Cyclohydrolase | 1 | N |
| JMJD6 | Jumonji Domain Containing 6, Arginine Demethylase And Lysine Hydroxylase | 1 | N |
| VSIR | V-Set Immunoregulatory Receptor | 1 | N |
| LIAS | Lipoic Acid Synthetase | 0.99 | N |
| IGFALS | Insulin Like Growth Factor Binding Protein Acid Labile Subunit | 0.99 | N |
| HSPA12B | Heat Shock Protein Family A (Hsp70) Member 12B | 0.99 | N |
| CFP | Complement Factor Properdin | 0.98 | N |
| GSTO2 | Glutathione S-Transferase Omega 2 | 0.98 | N |
| IPO5 | Importin 5 | 0.98 | N |
| SPRR3 | Small Proline Rich Protein 3 | 0.98 | N |
| KAT2B | Lysine Acetyltransferase 2B | 0.97 | N |
| TMPRSS6 | Transmembrane Serine Protease 6 | 0.97 | N |
| BECN1 | Beclin 1 | 0.97 | N |
| CEBPD | CCAAT Enhancer Binding Protein Delta | 0.97 | N |
| SRD5A2 | Steroid 5 Alpha-Reductase 2 | 0.96 | N |
| TRHDE | Thyrotropin Releasing Hormone Degrading Enzyme | 0.96 | N |
| IL6-AS1 | IL6 Antisense RNA 1 | 0.96 | N |
| ACKR1 | Atypical Chemokine Receptor 1 (Duffy Blood Group) | 0.95 | N |
| P4HA1 | Prolyl 4-Hydroxylase Subunit Alpha 1 | 0.95 | N |
| MRPS36P3 | Mitochondrial Ribosomal Protein S36 Pseudogene 3 | 0.95 | N |
| RPL31P26 | Ribosomal Protein L31 Pseudogene 26 | 0.95 | N |
| ESRRA | Estrogen Related Receptor Alpha | 0.94 | N |
| PDXP | Pyridoxal Phosphatase | 0.94 | N |
| HES1 | Hes Family BHLH Transcription Factor 1 | 0.94 | N |
| HDLCQ2 | High Density Lipoprotein Cholesterol Level QTL On Chromosome 8 | 0.94 | N |
| SSRP1 | Structure Specific Recognition Protein 1 | 0.93 | N |
| CD276 | CD276 Molecule | 0.93 | N |
| STXBP5 | Syntaxin Binding Protein 5 | 0.93 | N |
| PLCB3 | Phospholipase C Beta 3 | 0.93 | N |
| ANKRD6 | Ankyrin Repeat Domain 6 | 0.93 | N |
| HCAR2 | Hydroxycarboxylic Acid Receptor 2 | 0.93 | N |
| DGKQ | Diacylglycerol Kinase Theta | 0.92 | N |
| PTS | 6-Pyruvoyltetrahydropterin Synthase | 0.92 | N |
| GPAM | Glycerol-3-Phosphate Acyltransferase, Mitochondrial | 0.92 | N |
| GSK3A | Glycogen Synthase Kinase 3 Alpha | 0.91 | N |
| LTBR | Lymphotoxin Beta Receptor | 0.91 | N |
| NDUFS6 | NADH:Ubiquinone Oxidoreductase Subunit S6 | 0.91 | N |
| CSF1R | Colony Stimulating Factor 1 Receptor | 0.9 | N |
| SERPINB9 | Serpin Family B Member 9 | 0.9 | N |
| CCR8 | C-C Motif Chemokine Receptor 8 | 0.9 | N |
| LEKR1 | Leucine, Glutamate And Lysine Rich 1 | 0.89 | N |
| CD74 | CD74 Molecule | 0.89 | N |
| ABCA7 | ATP Binding Cassette Subfamily A Member 7 | 0.89 | N |
| BCL2A1 | BCL2 Related Protein A1 | 0.89 | N |
| NTM | Neurotrimin | 0.89 | N |
| STAB1 | Stabilin 1 | 0.89 | N |
| IMMT | Inner Membrane Mitochondrial Protein | 0.89 | N |
| RAB5A | RAB5A, Member RAS Oncogene Family | 0.89 | N |
| HSPA6 | Heat Shock Protein Family A (Hsp70) Member 6 | 0.88 | N |
| NUMA1 | Nuclear Mitotic Apparatus Protein 1 | 0.88 | N |
| CLPTM1L | CLPTM1 Like | 0.88 | N |
| CA1 | Carbonic Anhydrase 1 | 0.88 | N |
| TRAM1 | Translocation Associated Membrane Protein 1 | 0.88 | N |
| PDLIM5 | PDZ And LIM Domain 5 | 0.87 | N |
| TRAF3 | TNF Receptor Associated Factor 3 | 0.87 | N |
| HAL | Histidine Ammonia-Lyase | 0.87 | N |
| GBP1 | Guanylate Binding Protein 1 | 0.87 | N |
| LDHA | Lactate Dehydrogenase A | 0.87 | N |
| MIRLET7E | MicroRNA Let-7e | 0.87 | N |
| SGMS2 | Sphingomyelin Synthase 2 | 0.87 | N |
| DLG2 | Discs Large MAGUK Scaffold Protein 2 | 0.86 | N |
| IFI35 | Interferon Induced Protein 35 | 0.86 | N |
| CD160 | CD160 Molecule | 0.85 | N |
| OSCAR | Osteoclast Associated Ig-Like Receptor | 0.85 | N |
| SAA2 | Serum Amyloid A2 | 0.85 | N |
| IL20RB | Interleukin 20 Receptor Subunit Beta | 0.85 | N |
| HSF1 | Heat Shock Transcription Factor 1 | 0.85 | N |
| PDX1 | Pancreatic And Duodenal Homeobox 1 | 0.85 | N |
| STEAP1B | STEAP Family Member 1B | 0.85 | N |
| PDIA2 | Protein Disulfide Isomerase Family A Member 2 | 0.84 | N |
| CCL8 | C-C Motif Chemokine Ligand 8 | 0.84 | N |
| MT1X | Metallothionein 1X | 0.83 | N |
| NAB2 | NGFI-A Binding Protein 2 | 0.83 | N |
| CDK5RAP3 | CDK5 Regulatory Subunit Associated Protein 3 | 0.83 | N |
| LOC100506178 | Uncharacterized LOC100506178 | 0.83 | N |
| KCNN3 | Potassium Calcium-Activated Channel Subfamily N Member 3 | 0.82 | N |
| MIR128-1 | MicroRNA 128-1 | 0.82 | N |
| SRSF3 | Serine And Arginine Rich Splicing Factor 3 | 0.82 | N |
| DSCAML1 | DS Cell Adhesion Molecule Like 1 | 0.82 | N |
| APEH | Acylaminoacyl-Peptide Hydrolase | 0.82 | N |
| MIR9-2 | MicroRNA 9-2 | 0.81 | N |
| CCT8 | Chaperonin Containing TCP1 Subunit 8 | 0.81 | N |
| IRF1-AS1 | IRF1 Antisense RNA 1 | 0.81 | N |
| CCDC80 | Coiled-Coil Domain Containing 80 | 0.81 | N |
| CYLD | CYLD Lysine 63 Deubiquitinase | 0.81 | N |
| MIRLET7B | MicroRNA Let-7b | 0.8 | N |
| INPPL1 | Inositol Polyphosphate Phosphatase Like 1 | 0.8 | N |
| CSNK2A1 | Casein Kinase 2 Alpha 1 | 0.79 | N |
| DNM1L | Dynamin 1 Like | 0.79 | N |
| PRSS2 | Serine Protease 2 | 0.79 | N |
| MT3 | Metallothionein 3 | 0.79 | N |
| PARP14 | Poly(ADP-Ribose) Polymerase Family Member 14 | 0.78 | N |
| PARP9 | Poly(ADP-Ribose) Polymerase Family Member 9 | 0.78 | N |
| MIR1322 | MicroRNA 1322 | 0.78 | N |
| DGAT2 | Diacylglycerol O-Acyltransferase 2 | 0.77 | N |
| NDUFA10 | NADH:Ubiquinone Oxidoreductase Subunit A10 | 0.77 | N |
| SLC15A4 | Solute Carrier Family 15 Member 4 | 0.77 | N |
| FCN3 | Ficolin 3 | 0.77 | N |
| CLEC7A | C-Type Lectin Domain Containing 7A | 0.76 | N |
| ATG7 | Autophagy Related 7 | 0.76 | N |
| HPS5 | HPS5 Biogenesis Of Lysosomal Organelles Complex 2 Subunit 2 | 0.76 | N |
| UGT1A | UDP Glucuronosyltransferase Family 1 Member A Complex Locus | 0.75 | N |
| DNM3 | Dynamin 3 | 0.75 | N |
| EIF2AK2 | Eukaryotic Translation Initiation Factor 2 Alpha Kinase 2 | 0.75 | N |
| NBPF3 | NBPF Member 3 | 0.75 | N |
| AGPAT1 | 1-Acylglycerol-3-Phosphate O-Acyltransferase 1 | 0.74 | N |
| ATG16L1 | Autophagy Related 16 Like 1 | 0.74 | N |
| CD180 | CD180 Molecule | 0.74 | N |
| LY86 | Lymphocyte Antigen 86 | 0.73 | N |
| B4GALNT3 | Beta-1,4-N-Acetyl-Galactosaminyltransferase 3 | 0.73 | N |
| IGF2BP1 | Insulin Like Growth Factor 2 MRNA Binding Protein 1 | 0.73 | N |
| AKAP12 | A-Kinase Anchoring Protein 12 | 0.73 | N |
| MIR133A2 | MicroRNA 133a-2 | 0.73 | N |
| GORASP1 | Golgi Reassembly Stacking Protein 1 | 0.73 | N |
| TAP2 | Transporter 2, ATP Binding Cassette Subfamily B Member | 0.72 | N |
| IL36A | Interleukin 36 Alpha | 0.72 | N |
| CARD16 | Caspase Recruitment Domain Family Member 16 | 0.71 | N |
| TAS2R50 | Taste 2 Receptor Member 50 | 0.71 | N |
| PMVK | Phosphomevalonate Kinase | 0.71 | N |
| ORM1 | Orosomucoid 1 | 0.71 | N |
| UBIAD1 | UbiA Prenyltransferase Domain Containing 1 | 0.71 | N |
| LOC110673971 | CYP11B2 Promoter | 0.71 | N |
| PCK2 | Phosphoenolpyruvate Carboxykinase 2, Mitochondrial | 0.71 | N |
| TFPT | TCF3 Fusion Partner | 0.7 | N |
| MALRD1 | MAM And LDL Receptor Class A Domain Containing 1 | 0.69 | N |
| UBE2Q2P1 | Ubiquitin Conjugating Enzyme E2 Q2 Pseudogene 1 | 0.68 | N |
| RNF39 | Ring Finger Protein 39 | 0.68 | N |
| NEURL1 | Neuralized E3 Ubiquitin Protein Ligase 1 | 0.67 | N |
| PDCD6IP | Programmed Cell Death 6 Interacting Protein | 0.67 | N |
| TRIM63 | Tripartite Motif Containing 63 | 0.67 | N |
| BAIAP2L1 | BAR/IMD Domain Containing Adaptor Protein 2 Like 1 | 0.67 | N |
| CLEC5A | C-Type Lectin Domain Containing 5A | 0.67 | N |
| OAZ1 | Ornithine Decarboxylase Antizyme 1 | 0.67 | N |
| NR1D1 | Nuclear Receptor Subfamily 1 Group D Member 1 | 0.66 | N |
| MSRB1 | Methionine Sulfoxide Reductase B1 | 0.66 | N |
| BIRC3 | Baculoviral IAP Repeat Containing 3 | 0.66 | N |
| TCP1 | T-Complex 1 | 0.65 | N |
| LPAR2 | Lysophosphatidic Acid Receptor 2 | 0.64 | N |
| THRB | Thyroid Hormone Receptor Beta | 0.64 | N |
| CCND3 | Cyclin D3 | 0.64 | N |
| RPL31P23 | Ribosomal Protein L31 Pseudogene 23 | 0.64 | N |
| LEMD2 | LEM Domain Nuclear Envelope Protein 2 | 0.63 | N |
| ACSS2 | Acyl-CoA Synthetase Short Chain Family Member 2 | 0.63 | N |
| RFX5 | Regulatory Factor X5 | 0.63 | N |
| MXRA7 | Matrix Remodeling Associated 7 | 0.62 | N |
| GPR37L1 | G Protein-Coupled Receptor 37 Like 1 | 0.62 | N |
| SEC14L2 | SEC14 Like Lipid Binding 2 | 0.62 | N |
| MRTFB | Myocardin Related Transcription Factor B | 0.61 | N |
| ZBTB46 | Zinc Finger And BTB Domain Containing 46 | 0.6 | N |
| MIR378A | MicroRNA 378a | 0.6 | N |
| AP3D1 | Adaptor Related Protein Complex 3 Subunit Delta 1 | 0.6 | N |
| SGMS1 | Sphingomyelin Synthase 1 | 0.59 | N |
| NAT1 | N-Acetyltransferase 1 | 0.59 | N |
| TNFRSF14 | TNF Receptor Superfamily Member 14 | 0.59 | N |
| ANGPTL5 | Angiopoietin Like 5 | 0.59 | N |
| RPN1 | Ribophorin I | 0.58 | N |
| PTGR1 | Prostaglandin Reductase 1 | 0.58 | N |
| ARHGEF10 | Rho Guanine Nucleotide Exchange Factor 10 | 0.57 | N |
| MIR448 | MicroRNA 448 | 0.57 | N |
| FOXO4 | Forkhead Box O4 | 0.57 | N |
| B4GALT6 | Beta-1,4-Galactosyltransferase 6 | 0.57 | N |
| EPHA1 | EPH Receptor A1 | 0.57 | N |
| IDI1 | Isopentenyl-Diphosphate Delta Isomerase 1 | 0.57 | N |
| TNFRSF13C | TNF Receptor Superfamily Member 13C | 0.56 | N |
| ETS2 | ETS Proto-Oncogene 2, Transcription Factor | 0.56 | N |
| TRH | Thyrotropin Releasing Hormone | 0.56 | N |
| SNX17 | Sorting Nexin 17 | 0.55 | N |
| MIR636 | MicroRNA 636 | 0.55 | N |
| IL17RC | Interleukin 17 Receptor C | 0.55 | N |
| APOL4 | Apolipoprotein L4 | 0.54 | N |
| CA2 | Carbonic Anhydrase 2 | 0.53 | N |
| GATM | Glycine Amidinotransferase | 0.53 | N |
| ST8SIA1 | ST8 Alpha-N-Acetyl-Neuraminide Alpha-2,8-Sialyltransferase 1 | 0.53 | N |
| MTNR1A | Melatonin Receptor 1A | 0.52 | N |
| PKN2 | Protein Kinase N2 | 0.52 | N |
| RLBP1 | Retinaldehyde Binding Protein 1 | 0.52 | N |
| STK38 | Serine/Threonine Kinase 38 | 0.52 | N |
| PNLIPRP1 | Pancreatic Lipase Related Protein 1 | 0.52 | N |
| ANKRD30A | Ankyrin Repeat Domain 30A | 0.52 | N |
| MFSD11 | Major Facilitator Superfamily Domain Containing 11 | 0.51 | N |
| NEDD4 | NEDD4 E3 Ubiquitin Protein Ligase | 0.51 | N |
| GRID1 | Glutamate Ionotropic Receptor Delta Type Subunit 1 | 0.5 | N |
| PI16 | Peptidase Inhibitor 16 | 0.49 | N |
| KLRD1 | Killer Cell Lectin Like Receptor D1 | 0.49 | N |
| DOT1L | DOT1 Like Histone Lysine Methyltransferase | 0.49 | N |
| PDCD5 | Programmed Cell Death 5 | 0.48 | N |
| HDAC1 | Histone Deacetylase 1 | 0.48 | N |
| GOLGB1 | Golgin B1 | 0.48 | N |
| ABCA2 | ATP Binding Cassette Subfamily A Member 2 | 0.46 | N |
| TNFRSF10B | TNF Receptor Superfamily Member 10b | 0.46 | N |
| FBXO38 | F-Box Protein 38 | 0.46 | N |
| ACTG2 | Actin Gamma 2, Smooth Muscle | 0.46 | N |
| CXCL14 | C-X-C Motif Chemokine Ligand 14 | 0.46 | N |
| SF3A2 | Splicing Factor 3a Subunit 2 | 0.45 | N |
| RBFOX1 | RNA Binding Fox-1 Homolog 1 | 0.42 | N |
| BAZ2B | Bromodomain Adjacent To Zinc Finger Domain 2B | 0.42 | N |
| LARP6 | La Ribonucleoprotein 6, Translational Regulator | 0.42 | N |
| KDM6B | Lysine Demethylase 6B | 0.4 | N |
| CSMD2 | CUB And Sushi Multiple Domains 2 | 0.39 | N |
| MLST8 | MTOR Associated Protein, LST8 Homolog | 0.38 | N |
| ACKR2 | Atypical Chemokine Receptor 2 | 0.38 | N |
| PPT2 | Palmitoyl-Protein Thioesterase 2 | 0.38 | N |
| STUB1 | STIP1 Homology And U-Box Containing Protein 1 | 0.37 | N |
| NOSIP | Nitric Oxide Synthase Interacting Protein | 0.37 | N |
| SMILR | Smooth Muscle Induced LncRNA, Enhancer Of Proliferation | 0.35 | N |
| HSPA12A | Heat Shock Protein Family A (Hsp70) Member 12A | 0.34 | N |
| TICAM1 | Toll Like Receptor Adaptor Molecule 1 | 0.34 | N |
| DCD | Dermcidin | 0.33 | N |
| CPA3 | Carboxypeptidase A3 | 0.3 | N |
| KHK | Ketohexokinase | 0.27 | N |
| SIRT5 | Sirtuin 5 | 0.27 | N |
| BTC | Betacellulin | 0.27 | N |
| NRF1 | Nuclear Respiratory Factor 1 | 0.27 | N |
| PAG1 | Phosphoprotein Membrane Anchor With Glycosphingolipid Microdomains 1 | 0.27 | N |
| ZNF592 | Zinc Finger Protein 592 | 0.27 | N |
| PLCL2 | Phospholipase C Like 2 | 0.27 | N |
| TMEM161B | Transmembrane Protein 161B | 0.27 | N |
| RPL21P108 | Ribosomal Protein L21 Pseudogene 108 | 0.27 | N |
| RGS6 | Regulator Of G Protein Signaling 6 | 0.26 | N |
| DUSP6 | Dual Specificity Phosphatase 6 | 0.25 | N |

**Table S12.** Common and unique target in coronary artery atherosclerosis, herbal strategies and familiar remedies

| **Group** | **Members** | **Number** | **Target** |
| --- | --- | --- | --- |
| coronary artery atherosclerosis vs herbal strategy | strategy I/strategy II/strategy III/strategy IV/coronary artery atherosclerosis | 14 | MMP2 |
|  |  |  | XDH |
|  |  |  | MAOA |
|  |  |  | CDK1 |
|  |  |  | GSK3B |
|  |  |  | GLO1 |
|  |  |  | AKR1B1 |
|  |  |  | CYP1B1 |
|  |  |  | ALOX5 |
|  |  |  | NOX4 |
|  |  |  | ABCG2 |
|  |  |  | ADORA1 |
|  |  |  | CA2 |
|  |  |  | MMP9 |
|  | strategy I/strategy III/strategy IV/coronary artery atherosclerosis | 27 | MET |
|  |  |  | PTK2 |
|  |  |  | CYP19A1 |
|  |  |  | AKR1C4 |
|  |  |  | F2 |
|  |  |  | ALOX12 |
|  |  |  | MMP3 |
|  |  |  | AXL |
|  |  |  | CXCR1 |
|  |  |  | EGFR |
|  |  |  | SRC |
|  |  |  | CA3 |
|  |  |  | PIK3R1 |
|  |  |  | ABCC1 |
|  |  |  | AKR1A1 |
|  |  |  | CSNK2A1 |
|  |  |  | MMP13 |
|  |  |  | ADORA2A |
|  |  |  | ALOX15 |
|  |  |  | ABCB1 |
|  |  |  | PIM1 |
|  |  |  | MPO |
|  |  |  | KDR |
|  |  |  | IGF1R |
|  |  |  | AKT1 |
|  |  |  | CA1 |
|  |  |  | DAPK1 |
|  | strategy II/strategy III/strategy IV/coronary artery atherosclerosis | 19 | CCNB1 |
|  |  |  | CD38 |
|  |  |  | MMP12 |
|  |  |  | CCNB2 |
|  |  |  | TERT |
|  |  |  | PTPN6 |
|  |  |  | PTPN11 |
|  |  |  | EZH2 |
|  |  |  | STAT3 |
|  |  |  | ARG1 |
|  |  |  | TNKS |
|  |  |  | ACHE |
|  |  |  | POLB |
|  |  |  | PARP1 |
|  |  |  | TTR |
|  |  |  | SYK |
|  |  |  | CDK5 |
|  |  |  | CES1 |
|  |  |  | APP |
|  | strategy III/strategy IV/coronary artery atherosclerosis | 4 | ESRRA |
|  |  |  | AHR |
|  |  |  | TYR |
|  |  |  | GRK6 |
|  | strategy IV/coronary artery atherosclerosis | 14 | TEK |
|  |  |  | SHBG |
|  |  |  | PTGES |
|  |  |  | PDGFRB |
|  |  |  | CCND1 |
|  |  |  | CCNA2 |
|  |  |  | HTR1A |
|  |  |  | ERBB2 |
|  |  |  | GPR35 |
|  |  |  | CDK2 |
|  |  |  | FLT4 |
|  |  |  | DRD2 |
|  |  |  | HSPA1A |
|  |  |  | INSR |
|  | coronary artery atherosclerosis | 2918 | MSRB1 |
|  |  |  | FSTL1 |
|  |  |  | SLC18A1 |
|  |  |  | RIT2 |
|  |  |  | CD44 |
|  |  |  | ABCA3 |
|  |  |  | KCNMA1 |
|  |  |  | KCNJ2 |
|  |  |  | VPS33A |
|  |  |  | PINK1 |
|  |  |  | RALYL |
|  |  |  | ITGA5 |
|  |  |  | NDUFS6 |
|  |  |  | UTP20 |
|  |  |  | MEFV |
|  |  |  | EDN1 |
|  |  |  | TKT |
|  |  |  | HSPB1 |
|  |  |  | ABCC6 |
|  |  |  | CXCR4 |
|  |  |  | DMP1 |
|  |  |  | CUBN |
|  |  |  | RMRP |
|  |  |  | ADAM12 |
|  |  |  | IRAK1 |
|  |  |  | MSBP1 |
|  |  |  | IGF1 |
|  |  |  | TMSB10 |
|  |  |  | G3BP1 |
|  |  |  | MIR20A |
|  |  |  | CXCL3 |
|  |  |  | LMAN1 |
|  |  |  | BCAR4 |
|  |  |  | GAS5 |
|  |  |  | MIRLET7G |
|  |  |  | NCF1C |
|  |  |  | GALNT3 |
|  |  |  | LDB3 |
|  |  |  | PAK1 |
|  |  |  | SUMO1 |
|  |  |  | MIR6886 |
|  |  |  | GP1BA |
|  |  |  | ANGPTL5 |
|  |  |  | MRPL10 |
|  |  |  | GDF15 |
|  |  |  | CYCS |
|  |  |  | EP300 |
|  |  |  | TLN1 |
|  |  |  | MMP7 |
|  |  |  | NKX2-5 |
|  |  |  | PRKCG |
|  |  |  | NOS2 |
|  |  |  | TRIB3 |
|  |  |  | CACNA2D1 |
|  |  |  | HRG |
|  |  |  | PON3 |
|  |  |  | CDK14 |
|  |  |  | ERG |
|  |  |  | FAT4 |
|  |  |  | SOD2 |
|  |  |  | MIR15A |
|  |  |  | MSH3 |
|  |  |  | AQP9 |
|  |  |  | PDE1C |
|  |  |  | XK |
|  |  |  | SEMA4D |
|  |  |  | BAK1 |
|  |  |  | FGFR1 |
|  |  |  | AGPAT1 |
|  |  |  | MTNR1B |
|  |  |  | HLA-DRB5 |
|  |  |  | PCSK1 |
|  |  |  | OSBPL8 |
|  |  |  | SERPINA12 |
|  |  |  | NR4A2 |
|  |  |  | PPCS |
|  |  |  | UGT1A1 |
|  |  |  | CXCL13 |
|  |  |  | SORL1 |
|  |  |  | MNS1 |
|  |  |  | NAT10 |
|  |  |  | FADS1 |
|  |  |  | LEP |
|  |  |  | CXADR |
|  |  |  | SERPINF1 |
|  |  |  | CACNG5 |
|  |  |  | MAPKAPK2 |
|  |  |  | DIP2A |
|  |  |  | OXT |
|  |  |  | CCL22 |
|  |  |  | SHC1 |
|  |  |  | PTPN22 |
|  |  |  | ITGA3 |
|  |  |  | HSD11B2 |
|  |  |  | ENSG00000280087 |
|  |  |  | BRCA1 |
|  |  |  | GGT2 |
|  |  |  | LINC02618 |
|  |  |  | CRYGC |
|  |  |  | S1PR2 |
|  |  |  | RUNX3 |
|  |  |  | LIMS2 |
|  |  |  | PRKCB |
|  |  |  | C5AR1 |
|  |  |  | CYP2C19 |
|  |  |  | SMO |
|  |  |  | A2M |
|  |  |  | MIR132 |
|  |  |  | UTRN |
|  |  |  | CNN1 |
|  |  |  | BAZ1B |
|  |  |  | APOBR |
|  |  |  | LMNB2 |
|  |  |  | ROCK2 |
|  |  |  | PALLD |
|  |  |  | GPC5 |
|  |  |  | MT-ATP6 |
|  |  |  | OSBPL9P2 |
|  |  |  | TLR1 |
|  |  |  | VKORC1 |
|  |  |  | LRGUK |
|  |  |  | ELANE |
|  |  |  | CD160 |
|  |  |  | COL1A1 |
|  |  |  | PDCD5 |
|  |  |  | SLC8A1 |
|  |  |  | GPR37L1 |
|  |  |  | HES1 |
|  |  |  | OGDH |
|  |  |  | MACROD1 |
|  |  |  | GSS |
|  |  |  | C1QTNF3 |
|  |  |  | NECTIN2 |
|  |  |  | CCN4 |
|  |  |  | SSTR2 |
|  |  |  | FOXP3 |
|  |  |  | BIRC5 |
|  |  |  | CHRNA5 |
|  |  |  | UTS2B |
|  |  |  | MIR624 |
|  |  |  | SELL |
|  |  |  | GNAS |
|  |  |  | ANKRD6 |
|  |  |  | LTBP4 |
|  |  |  | PYY |
|  |  |  | GHR |
|  |  |  | CCL19 |
|  |  |  | SIRT6 |
|  |  |  | GPR55 |
|  |  |  | MTRR |
|  |  |  | NCOR2 |
|  |  |  | SERPINB9 |
|  |  |  | TNFRSF9 |
|  |  |  | CCNE1 |
|  |  |  | MS4A2 |
|  |  |  | PODN |
|  |  |  | BHMT |
|  |  |  | NPC2 |
|  |  |  | IL17RC |
|  |  |  | HS3ST1 |
|  |  |  | GUCY1A1 |
|  |  |  | ITGB1BP2 |
|  |  |  | RBPJ |
|  |  |  | GATA6 |
|  |  |  | APOE |
|  |  |  | PF4 |
|  |  |  | DSTN |
|  |  |  | ALDH2 |
|  |  |  | ST8SIA4 |
|  |  |  | DAPK3 |
|  |  |  | C3 |
|  |  |  | MRAP |
|  |  |  | TPM3 |
|  |  |  | ORM1 |
|  |  |  | HSPD1 |
|  |  |  | UGT1A12P |
|  |  |  | F2RL1 |
|  |  |  | PRDM16 |
|  |  |  | MIR378A |
|  |  |  | APOC4 |
|  |  |  | FABP5 |
|  |  |  | HYOU1 |
|  |  |  | UGT1A10 |
|  |  |  | TUBB |
|  |  |  | MFSD11 |
|  |  |  | SORT1 |
|  |  |  | PLIN2 |
|  |  |  | PPM1K |
|  |  |  | MYH6 |
|  |  |  | RBFOX1 |
|  |  |  | BCHE |
|  |  |  | ENOSF1 |
|  |  |  | SERPING1 |
|  |  |  | TONSL |
|  |  |  | GCLM |
|  |  |  | NFKB2 |
|  |  |  | BCL2 |
|  |  |  | ARL13B |
|  |  |  | CAMTA1 |
|  |  |  | TNFRSF10A |
|  |  |  | KL |
|  |  |  | P4HA3 |
|  |  |  | SAMSN1 |
|  |  |  | ALCAM |
|  |  |  | PLCL2 |
|  |  |  | IL24 |
|  |  |  | TRAM1 |
|  |  |  | BDKRB1 |
|  |  |  | NDUFS4 |
|  |  |  | ID1 |
|  |  |  | PIK3CB |
|  |  |  | COL18A1 |
|  |  |  | ENSG00000243797 |
|  |  |  | TNXB |
|  |  |  | CARD8 |
|  |  |  | BCAM |
|  |  |  | PCK2 |
|  |  |  | FKRP |
|  |  |  | TOX3 |
|  |  |  | TF |
|  |  |  | HLA-DRA |
|  |  |  | GCK |
|  |  |  | THRB |
|  |  |  | RNF39 |
|  |  |  | MYBPC1 |
|  |  |  | ADRA2A |
|  |  |  | NPR1 |
|  |  |  | TERC |
|  |  |  | ABRAXAS2 |
|  |  |  | GSTM4 |
|  |  |  | TMEM59 |
|  |  |  | SEC23IP |
|  |  |  | F7 |
|  |  |  | PDCL2 |
|  |  |  | EDNRA |
|  |  |  | MIR9-1 |
|  |  |  | CCT8 |
|  |  |  | POLG |
|  |  |  | IRS1 |
|  |  |  | GHRL |
|  |  |  | SLC17A3 |
|  |  |  | MFN2 |
|  |  |  | MICU1 |
|  |  |  | LOC111365141 |
|  |  |  | ATR |
|  |  |  | CYP1A1 |
|  |  |  | GNA11 |
|  |  |  | FOXB1 |
|  |  |  | EBPL |
|  |  |  | ARSH |
|  |  |  | TACR1 |
|  |  |  | CAPN1 |
|  |  |  | RYR1 |
|  |  |  | LTBP1 |
|  |  |  | MT3 |
|  |  |  | PPBP |
|  |  |  | ZNF687 |
|  |  |  | HTR3A |
|  |  |  | OXTR |
|  |  |  | ACTC1 |
|  |  |  | SDC2 |
|  |  |  | MAPK10 |
|  |  |  | ABI2 |
|  |  |  | CACNB1 |
|  |  |  | QKI |
|  |  |  | ATF1 |
|  |  |  | IL5 |
|  |  |  | FERMT3 |
|  |  |  | AGT |
|  |  |  | LRRC10 |
|  |  |  | MFAP5 |
|  |  |  | FGF1 |
|  |  |  | CHRM1 |
|  |  |  | IL6R |
|  |  |  | CST3 |
|  |  |  | CHI3L1 |
|  |  |  | LEMD2 |
|  |  |  | CLEC3B |
|  |  |  | CIITA |
|  |  |  | NOMO1 |
|  |  |  | CRP |
|  |  |  | GLA |
|  |  |  | NEBL |
|  |  |  | HSPA8 |
|  |  |  | IMMT |
|  |  |  | CEL |
|  |  |  | SELENOP |
|  |  |  | NEU1 |
|  |  |  | TARID |
|  |  |  | AGGF1 |
|  |  |  | PCCB |
|  |  |  | ENHO |
|  |  |  | NPHS1 |
|  |  |  | SPRY2 |
|  |  |  | GSTP1 |
|  |  |  | LTBR |
|  |  |  | CCR3 |
|  |  |  | NBPF3 |
|  |  |  | MIR340 |
|  |  |  | HBG2 |
|  |  |  | CYP7A1 |
|  |  |  | STAT5A |
|  |  |  | AGTRAP |
|  |  |  | SRD5A2 |
|  |  |  | MIR122 |
|  |  |  | ARHGEF38 |
|  |  |  | AP3B1 |
|  |  |  | MIR206 |
|  |  |  | KISS1R |
|  |  |  | NRG4 |
|  |  |  | MT-ND2 |
|  |  |  | IL5RA |
|  |  |  | TIAM1 |
|  |  |  | RGS14 |
|  |  |  | IL18BP |
|  |  |  | SMPD2 |
|  |  |  | MIR182 |
|  |  |  | FHL1 |
|  |  |  | TNFSF12 |
|  |  |  | CSRP3 |
|  |  |  | MTR |
|  |  |  | FN1 |
|  |  |  | LPIN3 |
|  |  |  | DHFR |
|  |  |  | ALPP |
|  |  |  | PPP1R12A |
|  |  |  | VSIR |
|  |  |  | PHACTR1 |
|  |  |  | GPR132 |
|  |  |  | ATP1A1 |
|  |  |  | LOXL1 |
|  |  |  | MIR144 |
|  |  |  | NFE2L2 |
|  |  |  | IL23R |
|  |  |  | NRF1 |
|  |  |  | SORCS2 |
|  |  |  | NR1I3 |
|  |  |  | ACVR2A |
|  |  |  | KLF4 |
|  |  |  | YWHAZ |
|  |  |  | KLKB1 |
|  |  |  | GAA |
|  |  |  | IFI27 |
|  |  |  | HDAC3 |
|  |  |  | SCN1A |
|  |  |  | HP |
|  |  |  | MAGOHB |
|  |  |  | MED18 |
|  |  |  | ECE1 |
|  |  |  | PARG |
|  |  |  | MT-LIPCAR |
|  |  |  | LIPI |
|  |  |  | UIMC1 |
|  |  |  | MIR29B1 |
|  |  |  | BMP6 |
|  |  |  | CLTCL1 |
|  |  |  | TRB |
|  |  |  | ALG6 |
|  |  |  | NPPA |
|  |  |  | TBX21 |
|  |  |  | PPARGC1A |
|  |  |  | TNFAIP6 |
|  |  |  | FMN1 |
|  |  |  | SSRP1 |
|  |  |  | CCL26 |
|  |  |  | BLOC1S5-TXNDC5 |
|  |  |  | SNAI1 |
|  |  |  | BRD4 |
|  |  |  | CD151 |
|  |  |  | MTHFD1 |
|  |  |  | HRH2 |
|  |  |  | PLA2G6 |
|  |  |  | CHDS3 |
|  |  |  | SOX9 |
|  |  |  | CYP3A5 |
|  |  |  | FTH1 |
|  |  |  | GC |
|  |  |  | IER3 |
|  |  |  | VSTM4 |
|  |  |  | MIR208B |
|  |  |  | CEBPD |
|  |  |  | ADRB1 |
|  |  |  | G6PC2 |
|  |  |  | OMD |
|  |  |  | WFDC21P |
|  |  |  | CSRP1 |
|  |  |  | CTSB |
|  |  |  | lnc-APOC1-1 |
|  |  |  | TMEM170A |
|  |  |  | ADCY3 |
|  |  |  | SVEP1 |
|  |  |  | FETUB |
|  |  |  | PXN |
|  |  |  | TNF |
|  |  |  | NTM |
|  |  |  | SIRT2 |
|  |  |  | C1QBP |
|  |  |  | NDUFB8 |
|  |  |  | GDF2 |
|  |  |  | NGFR |
|  |  |  | IKBKG |
|  |  |  | IL23A |
|  |  |  | TCF21 |
|  |  |  | NSD1 |
|  |  |  | JPH2 |
|  |  |  | C5 |
|  |  |  | SLC2A10 |
|  |  |  | EGF |
|  |  |  | TCP1 |
|  |  |  | GNRH1 |
|  |  |  | IL1A |
|  |  |  | CMA1 |
|  |  |  | DSCR10 |
|  |  |  | CACNA1H |
|  |  |  | PDGFD |
|  |  |  | FST |
|  |  |  | ERCC1 |
|  |  |  | CAV3 |
|  |  |  | KCND3 |
|  |  |  | CREBBP |
|  |  |  | LDLR |
|  |  |  | ITGB1 |
|  |  |  | PARK7 |
|  |  |  | RPN1 |
|  |  |  | PMVK |
|  |  |  | KLF2 |
|  |  |  | BRINP3 |
|  |  |  | COQ10A |
|  |  |  | SGK1 |
|  |  |  | ABCC2 |
|  |  |  | COL5A2 |
|  |  |  | RPL21P42 |
|  |  |  | SELP |
|  |  |  | PGM1 |
|  |  |  | MYOZ2 |
|  |  |  | DLGAP1 |
|  |  |  | PGLYRP1 |
|  |  |  | TANK |
|  |  |  | RCAN1 |
|  |  |  | AOC1 |
|  |  |  | MAP1LC3B |
|  |  |  | NAB2 |
|  |  |  | UGT1A8 |
|  |  |  | THY1 |
|  |  |  | KIT |
|  |  |  | KRT8 |
|  |  |  | SHH |
|  |  |  | THPO |
|  |  |  | RPL29P27 |
|  |  |  | MIR210 |
|  |  |  | EIF2S1 |
|  |  |  | SCN4B |
|  |  |  | PPOX |
|  |  |  | HAND2 |
|  |  |  | JAK2 |
|  |  |  | ANTXR1 |
|  |  |  | CREB3 |
|  |  |  | RAB5A |
|  |  |  | TERF2 |
|  |  |  | TNFRSF1A |
|  |  |  | HERC6 |
|  |  |  | RPL7AP57 |
|  |  |  | CASP1 |
|  |  |  | ATG7 |
|  |  |  | ACADVL |
|  |  |  | LRP1B |
|  |  |  | ZBTB8OS |
|  |  |  | SPP1 |
|  |  |  | HBP1 |
|  |  |  | TNNT2 |
|  |  |  | ABCG8 |
|  |  |  | SFTPD |
|  |  |  | EPS15 |
|  |  |  | HCAR2 |
|  |  |  | XIAP |
|  |  |  | ZNF627 |
|  |  |  | EIF2B2 |
|  |  |  | WNT5A |
|  |  |  | APOA2 |
|  |  |  | TRPA1 |
|  |  |  | KALRN |
|  |  |  | SUCLA2P2 |
|  |  |  | ATP6V0A2 |
|  |  |  | FDFT1 |
|  |  |  | ENO2 |
|  |  |  | PTGS2 |
|  |  |  | BMP7 |
|  |  |  | OSM |
|  |  |  | B2M |
|  |  |  | MIRLET7E |
|  |  |  | OPN3 |
|  |  |  | LRP5 |
|  |  |  | OLR1 |
|  |  |  | DAGLB |
|  |  |  | NRAP |
|  |  |  | TRIM63 |
|  |  |  | NCF1 |
|  |  |  | CD2 |
|  |  |  | SPG7 |
|  |  |  | AGER |
|  |  |  | GALNT2 |
|  |  |  | CTNNB1 |
|  |  |  | CBS |
|  |  |  | MIR221 |
|  |  |  | MICA |
|  |  |  | ADH1C |
|  |  |  | SNTA1 |
|  |  |  | MIR141 |
|  |  |  | TLR5 |
|  |  |  | HAS2 |
|  |  |  | FCGR3A |
|  |  |  | CYP2C9 |
|  |  |  | DNMT3A |
|  |  |  | NT5E |
|  |  |  | UTS2R |
|  |  |  | IL18 |
|  |  |  | NPY |
|  |  |  | ITGA6 |
|  |  |  | RPL23P4 |
|  |  |  | BAG3 |
|  |  |  | OXA1L |
|  |  |  | CTSK |
|  |  |  | IGFBP2 |
|  |  |  | NOTCH2 |
|  |  |  | SIGLEC1 |
|  |  |  | PRKDC |
|  |  |  | CD93 |
|  |  |  | LOC100506472 |
|  |  |  | CCL4 |
|  |  |  | MSR1 |
|  |  |  | PDCD6IP |
|  |  |  | ADD1 |
|  |  |  | TOR2A |
|  |  |  | KDM6B |
|  |  |  | ATXN2 |
|  |  |  | SLC17A4 |
|  |  |  | RRAS |
|  |  |  | S100B |
|  |  |  | BMP2 |
|  |  |  | IPO5 |
|  |  |  | NPY1R |
|  |  |  | MGP |
|  |  |  | PCDH15 |
|  |  |  | GYPA |
|  |  |  | TRPC1 |
|  |  |  | SOST |
|  |  |  | ESR1 |
|  |  |  | SURF4 |
|  |  |  | SLCO1B1 |
|  |  |  | KCNN3 |
|  |  |  | SST |
|  |  |  | ERCC8 |
|  |  |  | PRF1 |
|  |  |  | MAPK7 |
|  |  |  | RRAGC |
|  |  |  | GPX4 |
|  |  |  | ACE2 |
|  |  |  | FBXO3 |
|  |  |  | GCH1 |
|  |  |  | CMKLR1 |
|  |  |  | TYMS |
|  |  |  | DGKQ |
|  |  |  | SCNN1B |
|  |  |  | FMOD |
|  |  |  | IRS2 |
|  |  |  | SIK2 |
|  |  |  | SRRT |
|  |  |  | PDE4D |
|  |  |  | EPHB3 |
|  |  |  | TRPC4 |
|  |  |  | AAT1 |
|  |  |  | RAMP3 |
|  |  |  | CNTN5 |
|  |  |  | ESRRB |
|  |  |  | LRIG1 |
|  |  |  | HRAS |
|  |  |  | NPNT |
|  |  |  | HCG27 |
|  |  |  | TNFRSF10C |
|  |  |  | PDE3A |
|  |  |  | TRHDE |
|  |  |  | ITGB2 |
|  |  |  | SELENOS |
|  |  |  | XYLT2 |
|  |  |  | EPB41L3 |
|  |  |  | MIR361 |
|  |  |  | BCAR3 |
|  |  |  | CDKN2B |
|  |  |  | DHCR7 |
|  |  |  | PROS1 |
|  |  |  | PSMB8 |
|  |  |  | FGFR4 |
|  |  |  | CRAT |
|  |  |  | POR |
|  |  |  | SLC19A1 |
|  |  |  | LIMS1 |
|  |  |  | XRCC1 |
|  |  |  | SREBF2 |
|  |  |  | VEGFA |
|  |  |  | LAMA1 |
|  |  |  | APOL1 |
|  |  |  | ENPP2 |
|  |  |  | FHL5 |
|  |  |  | ACAN |
|  |  |  | TGFB1 |
|  |  |  | ITGA2 |
|  |  |  | GPR182 |
|  |  |  | MME |
|  |  |  | APEX1 |
|  |  |  | ACKR2 |
|  |  |  | MAST4 |
|  |  |  | LPAL2 |
|  |  |  | GAST |
|  |  |  | PSEN2 |
|  |  |  | UBE2L3 |
|  |  |  | MYC |
|  |  |  | ADRA1D |
|  |  |  | NR4A3 |
|  |  |  | MCTP2 |
|  |  |  | ARSA |
|  |  |  | LCMT2 |
|  |  |  | MBL1P |
|  |  |  | PTGIR |
|  |  |  | ENSG00000247287 |
|  |  |  | FOXE3 |
|  |  |  | SOCS3 |
|  |  |  | AGBL1 |
|  |  |  | RNPC3 |
|  |  |  | DAG1 |
|  |  |  | WWOX |
|  |  |  | SLC9A3R2 |
|  |  |  | TIMP1 |
|  |  |  | GAPDHP28 |
|  |  |  | C19orf38 |
|  |  |  | ABCA12 |
|  |  |  | CHIT1 |
|  |  |  | PCSK2 |
|  |  |  | PI3 |
|  |  |  | MRC1 |
|  |  |  | MT-ND6 |
|  |  |  | VCP |
|  |  |  | PCSK9 |
|  |  |  | LAMA3 |
|  |  |  | BGN |
|  |  |  | GAL |
|  |  |  | COCH |
|  |  |  | RAVER1 |
|  |  |  | IL13 |
|  |  |  | CD86 |
|  |  |  | SRSF3 |
|  |  |  | SLC11A1 |
|  |  |  | ICAM2 |
|  |  |  | MIR21 |
|  |  |  | INTS12 |
|  |  |  | CTSF |
|  |  |  | PDXP |
|  |  |  | NFIA-AS1 |
|  |  |  | GPD1L |
|  |  |  | TNFAIP3 |
|  |  |  | PSRC1 |
|  |  |  | PLA2G10 |
|  |  |  | FOXO1 |
|  |  |  | GPT |
|  |  |  | BLK |
|  |  |  | PPP1R17 |
|  |  |  | APOM |
|  |  |  | IBSP |
|  |  |  | PKP2 |
|  |  |  | GLTPD2 |
|  |  |  | MMP1 |
|  |  |  | STAT1 |
|  |  |  | HLA-DQA1 |
|  |  |  | GCLC |
|  |  |  | IGFBP5 |
|  |  |  | COL8A1 |
|  |  |  | SERPINA4 |
|  |  |  | MROH5 |
|  |  |  | FKTN |
|  |  |  | NME4 |
|  |  |  | IL18RAP |
|  |  |  | CARD16 |
|  |  |  | BLVRB |
|  |  |  | TNFSF13 |
|  |  |  | HABP2 |
|  |  |  | VENTXP2 |
|  |  |  | DLL4 |
|  |  |  | MYOCD |
|  |  |  | FBXO15 |
|  |  |  | SLC2A1 |
|  |  |  | LSAMP |
|  |  |  | ATP2A3 |
|  |  |  | MKKS |
|  |  |  | PTGER4 |
|  |  |  | CD68 |
|  |  |  | CCR7 |
|  |  |  | SMARCA1 |
|  |  |  | KCNQ1 |
|  |  |  | RECQL5 |
|  |  |  | APOF |
|  |  |  | ADCY1 |
|  |  |  | NR2F2 |
|  |  |  | BCL2A1 |
|  |  |  | ANGPTL1 |
|  |  |  | RHO |
|  |  |  | GRID1 |
|  |  |  | FHOD3 |
|  |  |  | BANF1 |
|  |  |  | CR1 |
|  |  |  | PRDM1 |
|  |  |  | FAM13A |
|  |  |  | TOR1A |
|  |  |  | BSG |
|  |  |  | P2RY4 |
|  |  |  | TRPV1 |
|  |  |  | UCP2 |
|  |  |  | EFEMP1 |
|  |  |  | ZFYVE9 |
|  |  |  | MIR28 |
|  |  |  | TRIM24 |
|  |  |  | MIRLET7A1 |
|  |  |  | MOCS1 |
|  |  |  | PROC |
|  |  |  | PLXNA4 |
|  |  |  | SLC22A5 |
|  |  |  | GRIN3A |
|  |  |  | DOCK6 |
|  |  |  | FPR1 |
|  |  |  | SYP |
|  |  |  | GNLY |
|  |  |  | FAM223A |
|  |  |  | CCR4 |
|  |  |  | PTPRC |
|  |  |  | CCT7 |
|  |  |  | MYO1E |
|  |  |  | EEF1A2 |
|  |  |  | MIPEP |
|  |  |  | CD14 |
|  |  |  | ABCC9 |
|  |  |  | NDE1 |
|  |  |  | HMGCR |
|  |  |  | PPARD |
|  |  |  | ANXA2 |
|  |  |  | MCF2L |
|  |  |  | SPEG |
|  |  |  | ARHGAP20 |
|  |  |  | RPL35AP15 |
|  |  |  | LGALS2 |
|  |  |  | NR3C2 |
|  |  |  | MIR134 |
|  |  |  | C4B |
|  |  |  | GSR |
|  |  |  | INPPL1 |
|  |  |  | CACNA1C |
|  |  |  | IL19 |
|  |  |  | HDLCQ2 |
|  |  |  | GGT1 |
|  |  |  | EIF2AK2 |
|  |  |  | WNT1 |
|  |  |  | CCL11 |
|  |  |  | LMO7 |
|  |  |  | PSMD9 |
|  |  |  | RHOD |
|  |  |  | IDO1 |
|  |  |  | COL4A2 |
|  |  |  | KDM5B |
|  |  |  | CCL23 |
|  |  |  | LUM |
|  |  |  | OBSL1 |
|  |  |  | C1S |
|  |  |  | HMOX1 |
|  |  |  | SLC27A6 |
|  |  |  | FCGR2A |
|  |  |  | MC4R |
|  |  |  | GPIHBP1 |
|  |  |  | MIR195 |
|  |  |  | FAM126A |
|  |  |  | FOXP2 |
|  |  |  | TRPM7 |
|  |  |  | INS |
|  |  |  | FLT1 |
|  |  |  | PDIA2 |
|  |  |  | RPLP0 |
|  |  |  | EIF4E |
|  |  |  | HOTAIR |
|  |  |  | EBI3 |
|  |  |  | SMAD2 |
|  |  |  | EFNA5 |
|  |  |  | RPL5P19 |
|  |  |  | ADGRE5 |
|  |  |  | MSBP2 |
|  |  |  | F2RL2 |
|  |  |  | IGF2BP2 |
|  |  |  | BGLAP |
|  |  |  | HTR4 |
|  |  |  | PRKCZ |
|  |  |  | EIF2B4 |
|  |  |  | IL20 |
|  |  |  | MIR590 |
|  |  |  | TRAF6 |
|  |  |  | CCL18 |
|  |  |  | LMNB1 |
|  |  |  | PNPLA3 |
|  |  |  | CNTLN |
|  |  |  | CD276 |
|  |  |  | PLD2 |
|  |  |  | F13A1 |
|  |  |  | SLC10A1 |
|  |  |  | GATA3 |
|  |  |  | LDLR-AS1-001 |
|  |  |  | TFAM |
|  |  |  | SYNE3 |
|  |  |  | OGN |
|  |  |  | FMR1 |
|  |  |  | LDLRAP1 |
|  |  |  | ANGPTL4 |
|  |  |  | HHEX |
|  |  |  | SLC12A3 |
|  |  |  | C9orf72 |
|  |  |  | PLCH1 |
|  |  |  | ZPR1 |
|  |  |  | NAMPT |
|  |  |  | PTGER2 |
|  |  |  | MMP8 |
|  |  |  | ESAM |
|  |  |  | MIR34A |
|  |  |  | FOXC2 |
|  |  |  | NGEF |
|  |  |  | HDAC4 |
|  |  |  | GDNF |
|  |  |  | CTNNA1 |
|  |  |  | ZNF717 |
|  |  |  | MIR497 |
|  |  |  | MIR222 |
|  |  |  | TRPC5 |
|  |  |  | SCGB1A1 |
|  |  |  | NTRK2 |
|  |  |  | CCDC3 |
|  |  |  | ADCYAP1 |
|  |  |  | MFGE8 |
|  |  |  | GSTM1 |
|  |  |  | CRH |
|  |  |  | IL10 |
|  |  |  | PRSS2 |
|  |  |  | CES3 |
|  |  |  | WDR33 |
|  |  |  | ACP5 |
|  |  |  | lnc-KDM5D-4 |
|  |  |  | AMFR |
|  |  |  | LIAS |
|  |  |  | MAPK1 |
|  |  |  | GOLGB1 |
|  |  |  | PGF |
|  |  |  | F11-AS1 |
|  |  |  | CORT |
|  |  |  | TLR10 |
|  |  |  | CHD7 |
|  |  |  | HNRNPA2B1 |
|  |  |  | SLC30A8 |
|  |  |  | PYGB |
|  |  |  | GADD45A |
|  |  |  | TSC1 |
|  |  |  | MIR214 |
|  |  |  | OARD1 |
|  |  |  | IAPP |
|  |  |  | ALOX5AP |
|  |  |  | LRP1 |
|  |  |  | POSTN |
|  |  |  | GP1BB |
|  |  |  | ANKH |
|  |  |  | RIPK3 |
|  |  |  | POM121L3P |
|  |  |  | FTO |
|  |  |  | CYP27B1 |
|  |  |  | SAMHD1 |
|  |  |  | PPP3R1 |
|  |  |  | LDLR-AS1 |
|  |  |  | PAX6 |
|  |  |  | SCG2 |
|  |  |  | ZC3HC1 |
|  |  |  | IGF2-AS |
|  |  |  | MMP14 |
|  |  |  | PLAT |
|  |  |  | SF3A1 |
|  |  |  | MDM2 |
|  |  |  | TAP2 |
|  |  |  | NETO1 |
|  |  |  | TNFSF11 |
|  |  |  | THSD7A |
|  |  |  | PTPRF |
|  |  |  | BLVRA |
|  |  |  | ICMT |
|  |  |  | GNAI1 |
|  |  |  | AKT2 |
|  |  |  | PPFIA2 |
|  |  |  | IKBKB |
|  |  |  | SLC25A4 |
|  |  |  | MYL3 |
|  |  |  | THBS4 |
|  |  |  | TNPO1 |
|  |  |  | ENOX1 |
|  |  |  | MYLK |
|  |  |  | CYP4F2 |
|  |  |  | SOD1 |
|  |  |  | B4GALNT3 |
|  |  |  | PRKD2 |
|  |  |  | MIR130A |
|  |  |  | SIRT5 |
|  |  |  | CXCR3 |
|  |  |  | CYSLTR1 |
|  |  |  | MIRLET7B |
|  |  |  | CTLA4 |
|  |  |  | NR4A1 |
|  |  |  | EMC10 |
|  |  |  | PLA2G2D |
|  |  |  | WNT2 |
|  |  |  | GLMN |
|  |  |  | SIRPA |
|  |  |  | LYVE1 |
|  |  |  | CSMD2 |
|  |  |  | APOH |
|  |  |  | CREB1 |
|  |  |  | PDX1 |
|  |  |  | ADAMTS8 |
|  |  |  | SMAD1 |
|  |  |  | TLR7 |
|  |  |  | STAB1 |
|  |  |  | MXRA7 |
|  |  |  | SRD5A1 |
|  |  |  | LGALS3 |
|  |  |  | UGT1A7 |
|  |  |  | CFL2 |
|  |  |  | STEAP1B |
|  |  |  | FUT3 |
|  |  |  | CLEC7A |
|  |  |  | DHX38 |
|  |  |  | HSPA14 |
|  |  |  | KCNA3 |
|  |  |  | DCBLD1 |
|  |  |  | PDGFA |
|  |  |  | IL17F |
|  |  |  | ZHX2 |
|  |  |  | CFL1 |
|  |  |  | IL4 |
|  |  |  | CD28 |
|  |  |  | SELPLG |
|  |  |  | DIABLO |
|  |  |  | SMARCA4 |
|  |  |  | BAZ2B |
|  |  |  | NAT2 |
|  |  |  | LRP8 |
|  |  |  | CNTN1 |
|  |  |  | MASP2 |
|  |  |  | AGL |
|  |  |  | GBP1 |
|  |  |  | MIR30D |
|  |  |  | NISCH |
|  |  |  | DEFA1 |
|  |  |  | LOC106728418 |
|  |  |  | F13B |
|  |  |  | TMEM43 |
|  |  |  | AKAP12 |
|  |  |  | CLPTM1L |
|  |  |  | MEOX2 |
|  |  |  | HLA-DPB1 |
|  |  |  | STAT5B |
|  |  |  | PRPF8 |
|  |  |  | ZNF213-AS1 |
|  |  |  | MIR93 |
|  |  |  | CEP85L |
|  |  |  | MIR224 |
|  |  |  | CD74 |
|  |  |  | HSPA12B |
|  |  |  | LIPG |
|  |  |  | G6PD |
|  |  |  | KLF5 |
|  |  |  | APOD |
|  |  |  | TRPV4 |
|  |  |  | NEK8 |
|  |  |  | PDE5A |
|  |  |  | SCARA3 |
|  |  |  | NOX5 |
|  |  |  | ENSG00000267052 |
|  |  |  | SULT1A3 |
|  |  |  | MAPK8 |
|  |  |  | CPA3 |
|  |  |  | KCNJ12 |
|  |  |  | ELOVL2 |
|  |  |  | C5AR2 |
|  |  |  | NRG3 |
|  |  |  | F10 |
|  |  |  | GSC2 |
|  |  |  | ACVR1 |
|  |  |  | COMP |
|  |  |  | HLA-DQA2 |
|  |  |  | STAR |
|  |  |  | F2R |
|  |  |  | THBS1 |
|  |  |  | MTAP |
|  |  |  | RFC1 |
|  |  |  | ACSL1 |
|  |  |  | H2AX |
|  |  |  | TCAP |
|  |  |  | ABL1 |
|  |  |  | NCOR1 |
|  |  |  | MIR143 |
|  |  |  | MOCOS |
|  |  |  | TXNRD1 |
|  |  |  | GZMB |
|  |  |  | FCGR3B |
|  |  |  | BMP1 |
|  |  |  | VIP |
|  |  |  | SP2 |
|  |  |  | CAPS2 |
|  |  |  | TAZ |
|  |  |  | CWC22 |
|  |  |  | MTFP1 |
|  |  |  | MMP26 |
|  |  |  | HLA-G |
|  |  |  | DIO2 |
|  |  |  | LRP2 |
|  |  |  | GPER1 |
|  |  |  | CYBB |
|  |  |  | MYDGF |
|  |  |  | HSP90AB1 |
|  |  |  | SEMA6D |
|  |  |  | CABIN1 |
|  |  |  | HSPE1 |
|  |  |  | MAPK13 |
|  |  |  | MYCN |
|  |  |  | SYNE1 |
|  |  |  | MTHFD1L |
|  |  |  | MVK |
|  |  |  | C2 |
|  |  |  | GCKR |
|  |  |  | APLN |
|  |  |  | DPP9 |
|  |  |  | NCOA1 |
|  |  |  | GUSB |
|  |  |  | HRH1 |
|  |  |  | DNM2 |
|  |  |  | PLA2G7 |
|  |  |  | CCL1 |
|  |  |  | CD247 |
|  |  |  | NEURL1 |
|  |  |  | KRT18 |
|  |  |  | CEP19 |
|  |  |  | SOCS1 |
|  |  |  | MIR155 |
|  |  |  | CDK5RAP3 |
|  |  |  | GGCX |
|  |  |  | HTRA1 |
|  |  |  | IFT88 |
|  |  |  | RNF111 |
|  |  |  | PTH |
|  |  |  | TNFRSF14 |
|  |  |  | TARDBP |
|  |  |  | PJA1 |
|  |  |  | RB1 |
|  |  |  | CETP |
|  |  |  | APOC2 |
|  |  |  | HADHB |
|  |  |  | ADAR |
|  |  |  | ARMS2 |
|  |  |  | PSMD4 |
|  |  |  | DNAH10 |
|  |  |  | P2RX1 |
|  |  |  | CACNB2 |
|  |  |  | NUMB |
|  |  |  | MIR25 |
|  |  |  | CD70 |
|  |  |  | SLC29A1 |
|  |  |  | GLRX |
|  |  |  | SAA1 |
|  |  |  | PF4V1 |
|  |  |  | ST3GAL4 |
|  |  |  | REG1A |
|  |  |  | HBB |
|  |  |  | HEY1 |
|  |  |  | KNG1 |
|  |  |  | ITGAX |
|  |  |  | CASR |
|  |  |  | MIR30E |
|  |  |  | GPAM |
|  |  |  | CANT1 |
|  |  |  | LIN9 |
|  |  |  | MTPN |
|  |  |  | TFAP2A |
|  |  |  | NFS1 |
|  |  |  | ADTRP |
|  |  |  | CDC37 |
|  |  |  | NUP107 |
|  |  |  | CHDS8 |
|  |  |  | PDE4B |
|  |  |  | PGR |
|  |  |  | RUNX2 |
|  |  |  | MT-CO1 |
|  |  |  | SPATA6L |
|  |  |  | GRK4 |
|  |  |  | LRP12 |
|  |  |  | SEC14L2 |
|  |  |  | FGF10 |
|  |  |  | IL1RN |
|  |  |  | ODC1 |
|  |  |  | MIR296 |
|  |  |  | MIR9-2 |
|  |  |  | ADORA2B |
|  |  |  | MAFB |
|  |  |  | PHEX |
|  |  |  | SOX6 |
|  |  |  | ADIPOR1 |
|  |  |  | TFPI |
|  |  |  | SNF8 |
|  |  |  | SGCD |
|  |  |  | SMTN |
|  |  |  | CYP17A1 |
|  |  |  | TWIST2 |
|  |  |  | MIR22 |
|  |  |  | NAB1 |
|  |  |  | CD209 |
|  |  |  | TNNI1 |
|  |  |  | ENO1 |
|  |  |  | CXCL10 |
|  |  |  | TRPM3 |
|  |  |  | P4HA1 |
|  |  |  | GOSR2 |
|  |  |  | RPL7AP58 |
|  |  |  | BFSP1 |
|  |  |  | CHDS4 |
|  |  |  | PLG |
|  |  |  | LOC102724465 |
|  |  |  | ATP6AP2 |
|  |  |  | COL4A1 |
|  |  |  | SEZ6L |
|  |  |  | VCAN |
|  |  |  | ADAMTS3 |
|  |  |  | RPL21P108 |
|  |  |  | LEKR1 |
|  |  |  | MKI67 |
|  |  |  | TSLP |
|  |  |  | DNAH8 |
|  |  |  | STS |
|  |  |  | IL4R |
|  |  |  | BTNL2 |
|  |  |  | CCL5 |
|  |  |  | ADAM8 |
|  |  |  | TGIF1 |
|  |  |  | PEX5 |
|  |  |  | TP53 |
|  |  |  | MAP1LC3A |
|  |  |  | piR-56133-114 |
|  |  |  | CDH7 |
|  |  |  | LINC-ROR |
|  |  |  | HNRNPA1P10 |
|  |  |  | ENTPD1 |
|  |  |  | CCDC157 |
|  |  |  | MIR140 |
|  |  |  | JUP |
|  |  |  | STK11 |
|  |  |  | MX1 |
|  |  |  | SAA4 |
|  |  |  | PLPP3 |
|  |  |  | LRRC18 |
|  |  |  | EXOSC4 |
|  |  |  | VASP |
|  |  |  | LOX |
|  |  |  | RELB |
|  |  |  | NLRP1 |
|  |  |  | BUD13 |
|  |  |  | HNF1B |
|  |  |  | ACP1 |
|  |  |  | TGFBR3 |
|  |  |  | CCN1 |
|  |  |  | UMOD |
|  |  |  | CASP9 |
|  |  |  | MAP3K7 |
|  |  |  | PDCD1 |
|  |  |  | NBL1 |
|  |  |  | SRA1 |
|  |  |  | PRMT5P1 |
|  |  |  | DYNC2LI1 |
|  |  |  | CDKN1A |
|  |  |  | IL32 |
|  |  |  | LPL |
|  |  |  | LAMP2 |
|  |  |  | CAT |
|  |  |  | LOC102723692 |
|  |  |  | HCN4 |
|  |  |  | MARS1 |
|  |  |  | ATXN1 |
|  |  |  | LIPC |
|  |  |  | CPOX |
|  |  |  | GSTZ1 |
|  |  |  | CD84 |
|  |  |  | APH1B |
|  |  |  | RASA1 |
|  |  |  | ADAM17 |
|  |  |  | LYZ |
|  |  |  | F5 |
|  |  |  | CLEC5A |
|  |  |  | TPM2 |
|  |  |  | ELAVL1 |
|  |  |  | CPS1 |
|  |  |  | LIPJ |
|  |  |  | PIAS4 |
|  |  |  | NCOA2 |
|  |  |  | GATA2 |
|  |  |  | ABCA2 |
|  |  |  | SLC2A2 |
|  |  |  | ATP10D |
|  |  |  | CCDC71L |
|  |  |  | HRC |
|  |  |  | CELSR2 |
|  |  |  | LRG1 |
|  |  |  | CFLAR |
|  |  |  | PRKCH |
|  |  |  | GRP |
|  |  |  | CACNG1 |
|  |  |  | PDGFRA |
|  |  |  | CSTB |
|  |  |  | ADRB3 |
|  |  |  | DMD |
|  |  |  | ADM |
|  |  |  | ANKRD30A |
|  |  |  | MIR150 |
|  |  |  | GTF2E2 |
|  |  |  | FCGR1A |
|  |  |  | FPR2 |
|  |  |  | DLC1 |
|  |  |  | CD47 |
|  |  |  | ACP6 |
|  |  |  | MIR199A1 |
|  |  |  | PPP1R1A |
|  |  |  | KCNQ3 |
|  |  |  | ABCB11 |
|  |  |  | SLC22A12 |
|  |  |  | SPRY4 |
|  |  |  | MXD1 |
|  |  |  | IFIH1 |
|  |  |  | LIPK |
|  |  |  | HADHA |
|  |  |  | CHURC1 |
|  |  |  | XRCC5 |
|  |  |  | CFDP1 |
|  |  |  | AHSG |
|  |  |  | POU5F1 |
|  |  |  | HDAC1 |
|  |  |  | USO1 |
|  |  |  | MASP1 |
|  |  |  | SHOX |
|  |  |  | RPL32P12 |
|  |  |  | FBXO38 |
|  |  |  | ICOS |
|  |  |  | NFKB1 |
|  |  |  | SIRT1 |
|  |  |  | TNFRSF6B |
|  |  |  | CAV2 |
|  |  |  | MIA3 |
|  |  |  | SEMA3F |
|  |  |  | MIR27B |
|  |  |  | UGT1A |
|  |  |  | IL1B |
|  |  |  | MIR505 |
|  |  |  | REN |
|  |  |  | CHRM3 |
|  |  |  | G6PC |
|  |  |  | NEK9 |
|  |  |  | ACTA1 |
|  |  |  | TOMM40 |
|  |  |  | CPE |
|  |  |  | CXCR6 |
|  |  |  | HPSE |
|  |  |  | IGF2R |
|  |  |  | DMWD |
|  |  |  | GSTM5 |
|  |  |  | FGF21 |
|  |  |  | LCAT |
|  |  |  | MIR196A2 |
|  |  |  | IL12B |
|  |  |  | ST6GAL1 |
|  |  |  | APOA4 |
|  |  |  | POMC |
|  |  |  | IL9 |
|  |  |  | ZMPSTE24 |
|  |  |  | APEX2 |
|  |  |  | SLC24A3 |
|  |  |  | RPL21P41 |
|  |  |  | TXNL4B |
|  |  |  | NLRP12 |
|  |  |  | piR-55948-110 |
|  |  |  | DNM1L |
|  |  |  | NPY4R |
|  |  |  | SH3GL2 |
|  |  |  | EGID-106632268 |
|  |  |  | MIR381 |
|  |  |  | ITGA7 |
|  |  |  | NFKBIA |
|  |  |  | CCDC159 |
|  |  |  | KCNIP2 |
|  |  |  | IGFBP3 |
|  |  |  | MPRIP |
|  |  |  | PSMA6 |
|  |  |  | SORBS3 |
|  |  |  | C4BPA |
|  |  |  | ALB |
|  |  |  | CPT1A |
|  |  |  | TERF1 |
|  |  |  | ALMS1 |
|  |  |  | HAMP |
|  |  |  | SREBF1 |
|  |  |  | DCN |
|  |  |  | RPL31P23 |
|  |  |  | NOD2 |
|  |  |  | IL21 |
|  |  |  | HTR2A |
|  |  |  | UCP3 |
|  |  |  | EPAS1 |
|  |  |  | SCNN1G |
|  |  |  | S100A12 |
|  |  |  | TREM1 |
|  |  |  | LOC106560211 |
|  |  |  | AMPD2 |
|  |  |  | ACTN1 |
|  |  |  | IFNAR2 |
|  |  |  | HLA-C |
|  |  |  | LINC00907 |
|  |  |  | TIMP4 |
|  |  |  | HSDL2 |
|  |  |  | LPIN1 |
|  |  |  | CX3CR1 |
|  |  |  | HACD4 |
|  |  |  | POU2F1 |
|  |  |  | HSPB7 |
|  |  |  | GAPDH |
|  |  |  | KISS1 |
|  |  |  | APC |
|  |  |  | TLR2 |
|  |  |  | GPX1 |
|  |  |  | CRLF2 |
|  |  |  | P2RX7 |
|  |  |  | DOCK7 |
|  |  |  | GNA12 |
|  |  |  | LIMK1 |
|  |  |  | KCNJ11 |
|  |  |  | RPL36AP23 |
|  |  |  | ABCC8 |
|  |  |  | MACROD2 |
|  |  |  | IL18R1 |
|  |  |  | BPI |
|  |  |  | GLB1 |
|  |  |  | BNC2 |
|  |  |  | SERPINA3 |
|  |  |  | GAS6 |
|  |  |  | RPL28P3 |
|  |  |  | TIMD4 |
|  |  |  | XKR4 |
|  |  |  | GSTM2 |
|  |  |  | STAB2 |
|  |  |  | NAT1 |
|  |  |  | PCOLCE2 |
|  |  |  | HCRT |
|  |  |  | HGFAC |
|  |  |  | PARP9 |
|  |  |  | MCPH1 |
|  |  |  | PEAR1 |
|  |  |  | MT2A |
|  |  |  | MMP10 |
|  |  |  | FABP2 |
|  |  |  | TCN2 |
|  |  |  | ACTG1 |
|  |  |  | CALM1 |
|  |  |  | TAS2R50 |
|  |  |  | HAVCR2 |
|  |  |  | PLAUR |
|  |  |  | MAP2K3 |
|  |  |  | NDP |
|  |  |  | CKM |
|  |  |  | SCAP |
|  |  |  | ESR2 |
|  |  |  | AGTR2 |
|  |  |  | MIR483 |
|  |  |  | LACTB |
|  |  |  | ADAMTSL1 |
|  |  |  | ZBTB46 |
|  |  |  | EPO |
|  |  |  | COL1A2 |
|  |  |  | DTNA |
|  |  |  | SERPINA5 |
|  |  |  | HNRNPA1 |
|  |  |  | LY96 |
|  |  |  | AKT3 |
|  |  |  | KLK3 |
|  |  |  | IL1R1 |
|  |  |  | ATP5IF1 |
|  |  |  | CTTN |
|  |  |  | KCNJ8 |
|  |  |  | PROCR |
|  |  |  | ITIH4 |
|  |  |  | USP8 |
|  |  |  | GJA5 |
|  |  |  | ITGB5 |
|  |  |  | SLC17A5 |
|  |  |  | HGF |
|  |  |  | MRTFB |
|  |  |  | SOX18 |
|  |  |  | TYMP |
|  |  |  | SERPINA10 |
|  |  |  | INSIG1 |
|  |  |  | NR1D1 |
|  |  |  | CFD |
|  |  |  | HIF1A-AS1 |
|  |  |  | MYBPC2 |
|  |  |  | CELA2A |
|  |  |  | APOC1 |
|  |  |  | MIR29A |
|  |  |  | HSPA12A |
|  |  |  | ITGAL |
|  |  |  | PDE4A |
|  |  |  | NHLRC1 |
|  |  |  | CH25H |
|  |  |  | KAT2B |
|  |  |  | SDHA |
|  |  |  | ALPL |
|  |  |  | STEAP2-AS1 |
|  |  |  | COMT |
|  |  |  | SMAD6 |
|  |  |  | PDLIM5 |
|  |  |  | SPC24 |
|  |  |  | MIR26A1 |
|  |  |  | XYLT1 |
|  |  |  | ADAMTS7 |
|  |  |  | ROS1 |
|  |  |  | C1QTNF1 |
|  |  |  | VEGFB |
|  |  |  | AS3MT |
|  |  |  | MT-TL1 |
|  |  |  | MIR208A |
|  |  |  | ACKR3 |
|  |  |  | MIR4675 |
|  |  |  | HYAL1 |
|  |  |  | ZBTB17 |
|  |  |  | LTA4H |
|  |  |  | HDLBP |
|  |  |  | CD1C |
|  |  |  | S1PR3 |
|  |  |  | GNB3 |
|  |  |  | IFIT3 |
|  |  |  | AVP |
|  |  |  | HMGB1 |
|  |  |  | PDE3B |
|  |  |  | HOPX |
|  |  |  | MEF2C |
|  |  |  | RPS3AP9 |
|  |  |  | SOAT2 |
|  |  |  | LBR |
|  |  |  | STEAP1 |
|  |  |  | PGK1 |
|  |  |  | CCL13 |
|  |  |  | MYB |
|  |  |  | BAIAP2L1 |
|  |  |  | ESM1 |
|  |  |  | PFN3 |
|  |  |  | RCN3 |
|  |  |  | RPTOR |
|  |  |  | ERBB4 |
|  |  |  | RYR3 |
|  |  |  | MRPS36P3 |
|  |  |  | E2F8 |
|  |  |  | UGCG |
|  |  |  | DNASE1 |
|  |  |  | PTN |
|  |  |  | MIR1322 |
|  |  |  | LPP |
|  |  |  | IL12RB1 |
|  |  |  | DMRT1 |
|  |  |  | ADRA2C |
|  |  |  | DHCR24 |
|  |  |  | VAMP8 |
|  |  |  | NBN |
|  |  |  | MIR31 |
|  |  |  | HSPB6 |
|  |  |  | COX5A |
|  |  |  | MYL4 |
|  |  |  | EBLN1 |
|  |  |  | CXCL14 |
|  |  |  | AIFM1 |
|  |  |  | GSTA1 |
|  |  |  | SLC17A1 |
|  |  |  | CYBA |
|  |  |  | MIR146A |
|  |  |  | SOS1 |
|  |  |  | TGFB2 |
|  |  |  | PLAU |
|  |  |  | RAC1 |
|  |  |  | LAMA2 |
|  |  |  | PTEN |
|  |  |  | ANXA1 |
|  |  |  | RLBP1 |
|  |  |  | MAATS1 |
|  |  |  | ZNF385D |
|  |  |  | ID3 |
|  |  |  | TICAM1 |
|  |  |  | FCRL6 |
|  |  |  | PROZ |
|  |  |  | C8A |
|  |  |  | LGR6 |
|  |  |  | MTM1 |
|  |  |  | MIR33A |
|  |  |  | RFX5 |
|  |  |  | SLC6A4 |
|  |  |  | CALR3 |
|  |  |  | NAT8 |
|  |  |  | UCN |
|  |  |  | PTRHD1 |
|  |  |  | SMDT1 |
|  |  |  | SYNE2 |
|  |  |  | SLC22A4 |
|  |  |  | NAXE |
|  |  |  | KCNE1 |
|  |  |  | CD4 |
|  |  |  | DSP |
|  |  |  | ITLN1 |
|  |  |  | NOSIP |
|  |  |  | COL20A1 |
|  |  |  | TUG1 |
|  |  |  | IRF1 |
|  |  |  | PTK2B |
|  |  |  | UNC5B |
|  |  |  | SDHB |
|  |  |  | IRF2BP2 |
|  |  |  | MBL2 |
|  |  |  | NGF |
|  |  |  | APOA1-AS |
|  |  |  | ALPK3 |
|  |  |  | NRP1 |
|  |  |  | ACAD8 |
|  |  |  | CDH13 |
|  |  |  | NLRP3 |
|  |  |  | GJA1 |
|  |  |  | PKD1 |
|  |  |  | IL6-AS1 |
|  |  |  | FASN |
|  |  |  | VEPH1 |
|  |  |  | PRDX6 |
|  |  |  | MYOM1 |
|  |  |  | TTF2 |
|  |  |  | AMBP |
|  |  |  | AKAP7 |
|  |  |  | AIDA |
|  |  |  | DOLK |
|  |  |  | ACVRL1 |
|  |  |  | CYCSP42 |
|  |  |  | ASCC3 |
|  |  |  | TGM2 |
|  |  |  | FCGR2B |
|  |  |  | NFATC4 |
|  |  |  | RXRA |
|  |  |  | MAP3K5 |
|  |  |  | GRM8 |
|  |  |  | PDLIM7 |
|  |  |  | SLC23A2 |
|  |  |  | MIR30A |
|  |  |  | NOS1 |
|  |  |  | TBK1 |
|  |  |  | BMPR1A |
|  |  |  | PRL |
|  |  |  | TIMP3 |
|  |  |  | LOC157273 |
|  |  |  | NR1H3 |
|  |  |  | ACACA |
|  |  |  | LYSMD4 |
|  |  |  | ABCG1 |
|  |  |  | MIR98 |
|  |  |  | SQSTM1 |
|  |  |  | MIR149 |
|  |  |  | DSCAML1 |
|  |  |  | NFATC1 |
|  |  |  | RAC2 |
|  |  |  | CD55 |
|  |  |  | PLN |
|  |  |  | MTHFR |
|  |  |  | ACAT1 |
|  |  |  | PRDM9 |
|  |  |  | CSGALNACT2 |
|  |  |  | SMAD3 |
|  |  |  | ATF3 |
|  |  |  | TBX5 |
|  |  |  | TRDN |
|  |  |  | NAPG |
|  |  |  | MRVI1 |
|  |  |  | HMOX2 |
|  |  |  | C1QL3 |
|  |  |  | MPP7 |
|  |  |  | PLCG1 |
|  |  |  | SLC2A13 |
|  |  |  | FBN2 |
|  |  |  | ST3GAL1 |
|  |  |  | NEDD4 |
|  |  |  | RPS27AP2 |
|  |  |  | MIR370 |
|  |  |  | MIR148A |
|  |  |  | LCT |
|  |  |  | MIR204 |
|  |  |  | DPP4 |
|  |  |  | DICER1 |
|  |  |  | CACNA1E |
|  |  |  | APOC3 |
|  |  |  | NPPB |
|  |  |  | SKP1 |
|  |  |  | MIR19A |
|  |  |  | PAFAH1B1 |
|  |  |  | APEH |
|  |  |  | H2AC18 |
|  |  |  | FBLIM1 |
|  |  |  | IFNA2 |
|  |  |  | PLTP |
|  |  |  | PLA2G2A |
|  |  |  | FOLH1 |
|  |  |  | FLYWCH1 |
|  |  |  | ANGPTL3 |
|  |  |  | LGALS1 |
|  |  |  | COL15A1 |
|  |  |  | DEFB128 |
|  |  |  | SLC25A1 |
|  |  |  | PCMT1 |
|  |  |  | MUC16 |
|  |  |  | SLC25A5 |
|  |  |  | WDR1 |
|  |  |  | FAS |
|  |  |  | PON1 |
|  |  |  | NPB |
|  |  |  | GSTA4 |
|  |  |  | ARNT |
|  |  |  | BDNF |
|  |  |  | UBA7 |
|  |  |  | HBEGF |
|  |  |  | SLC15A4 |
|  |  |  | CDKN1B |
|  |  |  | PRKCI |
|  |  |  | SEMA3A |
|  |  |  | SOD3 |
|  |  |  | FOXC1 |
|  |  |  | CYP21A2 |
|  |  |  | CYSLTR2 |
|  |  |  | ABCA1 |
|  |  |  | MIR223 |
|  |  |  | FGG |
|  |  |  | DDAH1 |
|  |  |  | CSF1 |
|  |  |  | SEMA3E |
|  |  |  | TNFSF10 |
|  |  |  | SPRYD4 |
|  |  |  | LOC110673971 |
|  |  |  | MIR502 |
|  |  |  | CRYAB |
|  |  |  | KRTAP11-1 |
|  |  |  | CKB |
|  |  |  | MUC1 |
|  |  |  | RTN3 |
|  |  |  | SKP2 |
|  |  |  | SLC2A3 |
|  |  |  | MAP3K14 |
|  |  |  | SLC40A1 |
|  |  |  | PTHLH |
|  |  |  | ARID3A |
|  |  |  | FEM1B |
|  |  |  | TNFRSF11B |
|  |  |  | C3AR1 |
|  |  |  | MIR27A |
|  |  |  | MALRD1 |
|  |  |  | CD36 |
|  |  |  | UBLCP1 |
|  |  |  | USP24 |
|  |  |  | MYH9 |
|  |  |  | LBP |
|  |  |  | MIR10A |
|  |  |  | LMF1 |
|  |  |  | C8orf48 |
|  |  |  | MIR215 |
|  |  |  | TNFSF15 |
|  |  |  | DUSP2 |
|  |  |  | NCF2 |
|  |  |  | BDKRB2 |
|  |  |  | IDH2 |
|  |  |  | MIR320A |
|  |  |  | HDGFL1 |
|  |  |  | PAG1 |
|  |  |  | MEF2A |
|  |  |  | TLR6 |
|  |  |  | C1QTNF5 |
|  |  |  | TNNC2 |
|  |  |  | APOL4 |
|  |  |  | HTN3 |
|  |  |  | CDKN2B-AS1 |
|  |  |  | ABO |
|  |  |  | STIM1 |
|  |  |  | KCNH2 |
|  |  |  | TRAF3 |
|  |  |  | CXCR2 |
|  |  |  | CAMK2D |
|  |  |  | F9 |
|  |  |  | LIPE |
|  |  |  | B4GALT1 |
|  |  |  | TMEM161B |
|  |  |  | RPS27A |
|  |  |  | ADCY5 |
|  |  |  | HMCN1 |
|  |  |  | MIR181B1 |
|  |  |  | TNNI3 |
|  |  |  | CD40 |
|  |  |  | LY86 |
|  |  |  | SGCB |
|  |  |  | IGFALS |
|  |  |  | E2F1 |
|  |  |  | PLCB3 |
|  |  |  | ARHGEF2 |
|  |  |  | APOB |
|  |  |  | CASP8 |
|  |  |  | CCM2 |
|  |  |  | CFH |
|  |  |  | PIGR |
|  |  |  | TSC2 |
|  |  |  | CYP11B2 |
|  |  |  | CHDS9 |
|  |  |  | MIR23A |
|  |  |  | ETS2 |
|  |  |  | CS |
|  |  |  | IGFBP7 |
|  |  |  | FCRL3 |
|  |  |  | SIRT4 |
|  |  |  | PRDX1 |
|  |  |  | FGB |
|  |  |  | CDH2 |
|  |  |  | ITGA1 |
|  |  |  | ECE2 |
|  |  |  | FLNC |
|  |  |  | ARF1 |
|  |  |  | ARID5B |
|  |  |  | HAL |
|  |  |  | MYOT |
|  |  |  | SGIP1 |
|  |  |  | SCD5 |
|  |  |  | TMEM248P1 |
|  |  |  | CAVIN1 |
|  |  |  | C4A |
|  |  |  | PPARG |
|  |  |  | AQP4 |
|  |  |  | MERTK |
|  |  |  | DSG2 |
|  |  |  | APLNR |
|  |  |  | HOXC-AS1 |
|  |  |  | ORMDL3 |
|  |  |  | JAG1 |
|  |  |  | TANC1 |
|  |  |  | TBXA2R |
|  |  |  | CHGA |
|  |  |  | ST2 |
|  |  |  | HSPA4 |
|  |  |  | PAPPA |
|  |  |  | DMPK |
|  |  |  | NEAT1 |
|  |  |  | RHOA |
|  |  |  | MIR494 |
|  |  |  | TRH |
|  |  |  | CCL7 |
|  |  |  | CALCA |
|  |  |  | PRKCD |
|  |  |  | VPS41 |
|  |  |  | LPXN |
|  |  |  | DSC2 |
|  |  |  | CHDS1 |
|  |  |  | CXCL11 |
|  |  |  | TMPRSS6 |
|  |  |  | SERPINA1 |
|  |  |  | TNNC1 |
|  |  |  | MIR92A1 |
|  |  |  | PPIG |
|  |  |  | SUN1 |
|  |  |  | LDAH |
|  |  |  | MAPT |
|  |  |  | PRKAA1 |
|  |  |  | FADS2 |
|  |  |  | IL1RL1 |
|  |  |  | SYNE4 |
|  |  |  | HLA-DQB1 |
|  |  |  | MT-CYB |
|  |  |  | GABRG1 |
|  |  |  | ZNF441 |
|  |  |  | AHCY |
|  |  |  | MTTP |
|  |  |  | CXCL8 |
|  |  |  | MAPK9 |
|  |  |  | EGR1 |
|  |  |  | CCL17 |
|  |  |  | THSD1 |
|  |  |  | KHK |
|  |  |  | RIPK2 |
|  |  |  | CHRNB4 |
|  |  |  | HDAC9 |
|  |  |  | GLP1R |
|  |  |  | OGG1 |
|  |  |  | PARP14 |
|  |  |  | DYM |
|  |  |  | RPL15P15 |
|  |  |  | RPL9P21 |
|  |  |  | TUBA1B |
|  |  |  | SELE |
|  |  |  | MSX2 |
|  |  |  | PSMC6 |
|  |  |  | CNR1 |
|  |  |  | PBRM1 |
|  |  |  | FBXO33 |
|  |  |  | CPB2 |
|  |  |  | LINC01535 |
|  |  |  | FGF19 |
|  |  |  | CTF1 |
|  |  |  | EGFL7 |
|  |  |  | PIK3R3 |
|  |  |  | STAP1 |
|  |  |  | KLF6 |
|  |  |  | SLC34A1 |
|  |  |  | THBD |
|  |  |  | NR3C1 |
|  |  |  | CYP4V2 |
|  |  |  | FCN2 |
|  |  |  | PIK3C2A |
|  |  |  | CORO1B |
|  |  |  | CHKB |
|  |  |  | SCUBE1 |
|  |  |  | F11 |
|  |  |  | CLEC4C |
|  |  |  | MAP2K6 |
|  |  |  | DCD |
|  |  |  | CD180 |
|  |  |  | EMP1 |
|  |  |  | IL17A |
|  |  |  | ATP2A1 |
|  |  |  | TMPO |
|  |  |  | COG2 |
|  |  |  | PNLIP |
|  |  |  | LEPR |
|  |  |  | MTOR |
|  |  |  | MIR124-1 |
|  |  |  | ABCA8 |
|  |  |  | PDE9A |
|  |  |  | PITPNA |
|  |  |  | NDUFS2 |
|  |  |  | STUB1 |
|  |  |  | GP6 |
|  |  |  | WT1 |
|  |  |  | ILK |
|  |  |  | LEPROT |
|  |  |  | MAPK14 |
|  |  |  | SERPINB2 |
|  |  |  | TXNRD2 |
|  |  |  | AOC3 |
|  |  |  | PITX2 |
|  |  |  | TNFRSF4 |
|  |  |  | MTHFD2 |
|  |  |  | GJA4 |
|  |  |  | MYBPC3 |
|  |  |  | FABP3 |
|  |  |  | TRPV2 |
|  |  |  | SLC27A1 |
|  |  |  | MYPN |
|  |  |  | CCN2 |
|  |  |  | HPR |
|  |  |  | ANXA6 |
|  |  |  | SLC22A11 |
|  |  |  | P2RY1 |
|  |  |  | TFPT |
|  |  |  | CSK |
|  |  |  | CTSS |
|  |  |  | MIR448 |
|  |  |  | SMPD1 |
|  |  |  | BHMT2 |
|  |  |  | RPL7 |
|  |  |  | TH |
|  |  |  | FBLN1 |
|  |  |  | GSTO2 |
|  |  |  | PRKAB2 |
|  |  |  | HSPB2 |
|  |  |  | RAF1 |
|  |  |  | MIR19B2 |
|  |  |  | STC1 |
|  |  |  | KLRK1 |
|  |  |  | FURIN |
|  |  |  | ADH7 |
|  |  |  | LTB4R2 |
|  |  |  | UNGP1 |
|  |  |  | ST8SIA1 |
|  |  |  | APOA5 |
|  |  |  | CCR2 |
|  |  |  | EDN3 |
|  |  |  | ANK2 |
|  |  |  | SPON1 |
|  |  |  | PLA2G4A |
|  |  |  | SF3A2 |
|  |  |  | VLDLR |
|  |  |  | VEGFC |
|  |  |  | GATAD1 |
|  |  |  | ITGB6 |
|  |  |  | PPP1R3B |
|  |  |  | SDC4 |
|  |  |  | LMOD1 |
|  |  |  | FHL2 |
|  |  |  | PDGFB |
|  |  |  | ELN |
|  |  |  | CALR |
|  |  |  | VIM |
|  |  |  | CADPS |
|  |  |  | ADRA1B |
|  |  |  | LTB |
|  |  |  | NUDT6 |
|  |  |  | TLR8 |
|  |  |  | KCNMB1 |
|  |  |  | DES |
|  |  |  | LGALS3BP |
|  |  |  | ITGAV |
|  |  |  | FGFR2 |
|  |  |  | XRCC3 |
|  |  |  | CSF3 |
|  |  |  | PCNA |
|  |  |  | MMACHC |
|  |  |  | RBM20 |
|  |  |  | B4GALT6 |
|  |  |  | PRKAR1A |
|  |  |  | ATG5 |
|  |  |  | HLA-A |
|  |  |  | SUPT3H |
|  |  |  | ADAMTS4 |
|  |  |  | TNNI2 |
|  |  |  | LDHA |
|  |  |  | EDNRB |
|  |  |  | CD27 |
|  |  |  | FAM167A |
|  |  |  | SCD |
|  |  |  | ARMH3 |
|  |  |  | MIR106B |
|  |  |  | FEM1A |
|  |  |  | HSPA6 |
|  |  |  | CHUK |
|  |  |  | CHAT |
|  |  |  | NR1H2 |
|  |  |  | SEC11B |
|  |  |  | CAPG |
|  |  |  | SPHK1 |
|  |  |  | ATN1 |
|  |  |  | MYH10 |
|  |  |  | ARSB |
|  |  |  | IL22 |
|  |  |  | ORAI1 |
|  |  |  | PARPBP |
|  |  |  | ADAM9 |
|  |  |  | TPH1 |
|  |  |  | STXBP5 |
|  |  |  | AMPD1 |
|  |  |  | TBXAS1 |
|  |  |  | MIR103A1 |
|  |  |  | MYH11 |
|  |  |  | IFNGR2 |
|  |  |  | AGK |
|  |  |  | CAP1 |
|  |  |  | KIF2C |
|  |  |  | DUSP1 |
|  |  |  | DDAH2 |
|  |  |  | FGF4 |
|  |  |  | NPR3 |
|  |  |  | SLPI |
|  |  |  | DMRTA1 |
|  |  |  | BCAR1 |
|  |  |  | ADAM10 |
|  |  |  | ATXN3 |
|  |  |  | PRPS1 |
|  |  |  | LIPA |
|  |  |  | P2RY2 |
|  |  |  | PLD5 |
|  |  |  | H19 |
|  |  |  | CAMLG |
|  |  |  | TREML4 |
|  |  |  | TGFBR1 |
|  |  |  | P2RY12 |
|  |  |  | GRK2 |
|  |  |  | CDC42 |
|  |  |  | ENSG00000269918 |
|  |  |  | VWF |
|  |  |  | VTN |
|  |  |  | TPM1 |
|  |  |  | MIR24-1 |
|  |  |  | CIDEC |
|  |  |  | EBF1 |
|  |  |  | NINJ2 |
|  |  |  | CYP2E1 |
|  |  |  | FLNB |
|  |  |  | WDFY4 |
|  |  |  | CTH |
|  |  |  | MFSD10 |
|  |  |  | IL15 |
|  |  |  | ATM |
|  |  |  | TIE1 |
|  |  |  | PLEKHA1 |
|  |  |  | TP53COR1 |
|  |  |  | ANGPTL8 |
|  |  |  | KCNK1 |
|  |  |  | C1orf210 |
|  |  |  | LIG4 |
|  |  |  | EREG |
|  |  |  | MIR30C2 |
|  |  |  | BMP4 |
|  |  |  | SFRP4 |
|  |  |  | AGTR1 |
|  |  |  | MCAM |
|  |  |  | IPMK |
|  |  |  | CAMP |
|  |  |  | OBSCN |
|  |  |  | ABCG4 |
|  |  |  | UGT1A4 |
|  |  |  | FCAMR |
|  |  |  | IL1RAP |
|  |  |  | MAT2B |
|  |  |  | SMILR |
|  |  |  | SRGN |
|  |  |  | PRDX5 |
|  |  |  | PPIA |
|  |  |  | CASP7 |
|  |  |  | MED6 |
|  |  |  | MCI2 |
|  |  |  | TRIB1 |
|  |  |  | PRKAG3 |
|  |  |  | TJP1 |
|  |  |  | EDEM2 |
|  |  |  | CD46 |
|  |  |  | PTX3 |
|  |  |  | ICOSLG |
|  |  |  | MIR128-1 |
|  |  |  | UGT1A6 |
|  |  |  | ANG |
|  |  |  | CCK |
|  |  |  | ADRB2 |
|  |  |  | TRIM9 |
|  |  |  | SMAD4 |
|  |  |  | CSMD1 |
|  |  |  | RETNLB |
|  |  |  | CTSD |
|  |  |  | SGMS1 |
|  |  |  | STAT6 |
|  |  |  | JCAD |
|  |  |  | FKBP1B |
|  |  |  | TRERF1 |
|  |  |  | SCARB1 |
|  |  |  | IL3 |
|  |  |  | VAV2 |
|  |  |  | PTCH1 |
|  |  |  | TP53BP1 |
|  |  |  | S100A1 |
|  |  |  | PTPA |
|  |  |  | GREM1 |
|  |  |  | SUN2 |
|  |  |  | CYP4A11 |
|  |  |  | TES |
|  |  |  | PRKAG2 |
|  |  |  | AGXT2 |
|  |  |  | EPRS1 |
|  |  |  | MIR197 |
|  |  |  | ILF3 |
|  |  |  | C1QTNF9 |
|  |  |  | MT1X |
|  |  |  | KEAP1 |
|  |  |  | RFC4 |
|  |  |  | LARP6 |
|  |  |  | SERPINF2 |
|  |  |  | MIAT |
|  |  |  | FBN1 |
|  |  |  | SGCG |
|  |  |  | TYRO3 |
|  |  |  | S100A8 |
|  |  |  | HAVCR1 |
|  |  |  | SLC10A2 |
|  |  |  | KRI1 |
|  |  |  | LEMD3 |
|  |  |  | CHKA |
|  |  |  | CPQ |
|  |  |  | TNFRSF13C |
|  |  |  | AIF1 |
|  |  |  | MAP3K1 |
|  |  |  | PBK |
|  |  |  | SOAT1 |
|  |  |  | AZU1 |
|  |  |  | CTNNA3 |
|  |  |  | MT-ND1 |
|  |  |  | HDLC3 |
|  |  |  | F8 |
|  |  |  | MIR133A1 |
|  |  |  | NOMO3 |
|  |  |  | TNFRSF12A |
|  |  |  | RPL29P29 |
|  |  |  | EGR3 |
|  |  |  | SERPIND1 |
|  |  |  | IL12A |
|  |  |  | ARNTL |
|  |  |  | BMPER |
|  |  |  | CTCF |
|  |  |  | MTNR1A |
|  |  |  | PPP1R3A |
|  |  |  | MCL1 |
|  |  |  | CCL2 |
|  |  |  | SURF2 |
|  |  |  | RGS9BP |
|  |  |  | MIR125A |
|  |  |  | ACTN2 |
|  |  |  | TXNIP |
|  |  |  | ATG9B |
|  |  |  | SHMT1 |
|  |  |  | ADCY10 |
|  |  |  | CCND3 |
|  |  |  | M6PR |
|  |  |  | ADA |
|  |  |  | SPATA7 |
|  |  |  | DOT1L |
|  |  |  | MAZ |
|  |  |  | IL27 |
|  |  |  | EIF2AK3 |
|  |  |  | PDCD10 |
|  |  |  | KLK15 |
|  |  |  | CAMK2G |
|  |  |  | IL6 |
|  |  |  | TLR3 |
|  |  |  | FGF14 |
|  |  |  | ANXA5 |
|  |  |  | EPHX2 |
|  |  |  | SSNA1 |
|  |  |  | LPAR2 |
|  |  |  | CCL24 |
|  |  |  | PRICKLE2 |
|  |  |  | ANGPT1 |
|  |  |  | RAN |
|  |  |  | CPT2 |
|  |  |  | GPR26 |
|  |  |  | GLRA3 |
|  |  |  | ADPRS |
|  |  |  | POLR2D |
|  |  |  | STAG3 |
|  |  |  | EPHA1 |
|  |  |  | MIR15B |
|  |  |  | EPOR |
|  |  |  | MIR185 |
|  |  |  | HNRNPC |
|  |  |  | LTB4R |
|  |  |  | RPL35AP22 |
|  |  |  | CASP3 |
|  |  |  | HSP90AA1 |
|  |  |  | ANGPT2 |
|  |  |  | CACNA2D3 |
|  |  |  | DUOX1 |
|  |  |  | TSBP1 |
|  |  |  | RNF213 |
|  |  |  | ACSS2 |
|  |  |  | PLIN1 |
|  |  |  | SUCLG1 |
|  |  |  | PKN2 |
|  |  |  | ITGA4 |
|  |  |  | VDR |
|  |  |  | ACE |
|  |  |  | CLOCK |
|  |  |  | MLX |
|  |  |  | NPY5R |
|  |  |  | PRKCE |
|  |  |  | ITGAM |
|  |  |  | ROCK1 |
|  |  |  | BLOC1S1 |
|  |  |  | CD34 |
|  |  |  | DSPP |
|  |  |  | BCO1 |
|  |  |  | RGS2 |
|  |  |  | CHDS2 |
|  |  |  | TAGLN |
|  |  |  | TPSAB1 |
|  |  |  | PDPN |
|  |  |  | MIR423 |
|  |  |  | DLL3 |
|  |  |  | PPT2 |
|  |  |  | FASLG |
|  |  |  | RTN4 |
|  |  |  | COL3A1 |
|  |  |  | ENG |
|  |  |  | MIR126 |
|  |  |  | LIPM |
|  |  |  | LIPF |
|  |  |  | RXRG |
|  |  |  | CACNA1D |
|  |  |  | PRKAA2 |
|  |  |  | UHRF1BP1 |
|  |  |  | CD163 |
|  |  |  | MIR125B1 |
|  |  |  | DUSP19 |
|  |  |  | KCNK2 |
|  |  |  | RGS5 |
|  |  |  | SORCS1 |
|  |  |  | TBC1D4 |
|  |  |  | DNM3 |
|  |  |  | RLN2 |
|  |  |  | BBS2 |
|  |  |  | IL1RAPL2 |
|  |  |  | TUBA4A |
|  |  |  | HPD |
|  |  |  | MYLIP |
|  |  |  | SPAG17 |
|  |  |  | HNF1A |
|  |  |  | EPHA3 |
|  |  |  | LMAN2 |
|  |  |  | MFAP4 |
|  |  |  | INSIG2 |
|  |  |  | TYROBP |
|  |  |  | DPRXP7 |
|  |  |  | RARA |
|  |  |  | B3GALT4 |
|  |  |  | OSCAR |
|  |  |  | PECAM1 |
|  |  |  | SFRP5 |
|  |  |  | MIR205 |
|  |  |  | MYD88 |
|  |  |  | TNC |
|  |  |  | PTPN1 |
|  |  |  | DLG2 |
|  |  |  | PML |
|  |  |  | BRAP |
|  |  |  | CCR8 |
|  |  |  | MYH14 |
|  |  |  | PPP1R12B |
|  |  |  | DAB2 |
|  |  |  | TBX4 |
|  |  |  | MIR636 |
|  |  |  | MARCKS |
|  |  |  | LRP6 |
|  |  |  | MIR145 |
|  |  |  | SERPINH1 |
|  |  |  | PPARA |
|  |  |  | CNR2 |
|  |  |  | KRIT1 |
|  |  |  | MIR30B |
|  |  |  | MGLL |
|  |  |  | RORA |
|  |  |  | PTPRD |
|  |  |  | ALOX15B |
|  |  |  | CDK9 |
|  |  |  | IL2RA |
|  |  |  | CD59 |
|  |  |  | PLCG2 |
|  |  |  | FAP |
|  |  |  | KCNJ1 |
|  |  |  | LRRTM4 |
|  |  |  | CCR1 |
|  |  |  | CLDN5 |
|  |  |  | ITGA2B |
|  |  |  | MAOB |
|  |  |  | PTPRN2 |
|  |  |  | PTGDS |
|  |  |  | PSAP |
|  |  |  | UGT1A9 |
|  |  |  | CRYAA |
|  |  |  | ATP1A2 |
|  |  |  | CYP1A2 |
|  |  |  | COPS5 |
|  |  |  | TNFSF4 |
|  |  |  | TPM4 |
|  |  |  | BCL2L11 |
|  |  |  | HERPUD1 |
|  |  |  | DUSP6 |
|  |  |  | TGFB3 |
|  |  |  | ELK1 |
|  |  |  | HGD |
|  |  |  | INHBA |
|  |  |  | BMPR2 |
|  |  |  | RBP4 |
|  |  |  | LOC100506178 |
|  |  |  | NME2 |
|  |  |  | ADAM33 |
|  |  |  | ZNF491 |
|  |  |  | S100A4 |
|  |  |  | BTC |
|  |  |  | MSH5 |
|  |  |  | LGMN |
|  |  |  | TCN1 |
|  |  |  | LEPQTL1 |
|  |  |  | CPEB2 |
|  |  |  | IGF2 |
|  |  |  | LILRB1 |
|  |  |  | CTSG |
|  |  |  | ADM2 |
|  |  |  | LINC02577 |
|  |  |  | CD80 |
|  |  |  | NUCB2 |
|  |  |  | RARRES2 |
|  |  |  | FOXO4 |
|  |  |  | ADPRH |
|  |  |  | DNMT1 |
|  |  |  | NOD1 |
|  |  |  | JMJD6 |
|  |  |  | KIF6 |
|  |  |  | ADAMTS1 |
|  |  |  | MIR486-1 |
|  |  |  | PRKG1 |
|  |  |  | CFI |
|  |  |  | SBF2 |
|  |  |  | CDH9 |
|  |  |  | ADRA2B |
|  |  |  | SUV39H1 |
|  |  |  | PLA2G5 |
|  |  |  | AAMP |
|  |  |  | CYP20A1 |
|  |  |  | RNASE3 |
|  |  |  | PIK3CG |
|  |  |  | ACKR1 |
|  |  |  | LPA |
|  |  |  | COL4A4 |
|  |  |  | FGF7 |
|  |  |  | IL37 |
|  |  |  | NPC1L1 |
|  |  |  | UTS2 |
|  |  |  | TAT |
|  |  |  | RREB1 |
|  |  |  | MB |
|  |  |  | C12orf43 |
|  |  |  | MIR92A2 |
|  |  |  | LIFR |
|  |  |  | NQO1 |
|  |  |  | CBL |
|  |  |  | SP1 |
|  |  |  | APOBEC1 |
|  |  |  | CACNG8 |
|  |  |  | SURF1 |
|  |  |  | MIR23B |
|  |  |  | CD79A |
|  |  |  | S1PR1 |
|  |  |  | IL1R2 |
|  |  |  | PIK3CA |
|  |  |  | SGCA |
|  |  |  | MAGI2 |
|  |  |  | LARGE1 |
|  |  |  | IRGM |
|  |  |  | CKLF |
|  |  |  | TSPO |
|  |  |  | SRIP1 |
|  |  |  | UCP1 |
|  |  |  | DAB2IP |
|  |  |  | FGF9 |
|  |  |  | IL2 |
|  |  |  | FADD |
|  |  |  | ZNF844 |
|  |  |  | SDC1 |
|  |  |  | FGA |
|  |  |  | SERPINB1 |
|  |  |  | IGFBP1 |
|  |  |  | SLC1A1 |
|  |  |  | SLC1A4 |
|  |  |  | DDIT3 |
|  |  |  | HOMER1 |
|  |  |  | PLA2G15 |
|  |  |  | CDH1 |
|  |  |  | PTGR1 |
|  |  |  | CALU |
|  |  |  | PNPLA5 |
|  |  |  | TBC1D1 |
|  |  |  | PLAG1 |
|  |  |  | FOS |
|  |  |  | IFNG |
|  |  |  | AGPAT2 |
|  |  |  | CREG1 |
|  |  |  | EMD |
|  |  |  | HSPA5 |
|  |  |  | MIR616 |
|  |  |  | FNDC5 |
|  |  |  | RETN |
|  |  |  | CCL8 |
|  |  |  | CYP27A1 |
|  |  |  | PAFAH2 |
|  |  |  | ALDH8A1 |
|  |  |  | IGES |
|  |  |  | CCDC80 |
|  |  |  | SSH1 |
|  |  |  | MIR135B |
|  |  |  | RHOB |
|  |  |  | H6PD |
|  |  |  | GNAQ |
|  |  |  | CAVIN4 |
|  |  |  | XPA |
|  |  |  | MIR142 |
|  |  |  | FADS3 |
|  |  |  | PEX3 |
|  |  |  | FGF2 |
|  |  |  | CLU |
|  |  |  | AMH |
|  |  |  | PRODH |
|  |  |  | IRF5 |
|  |  |  | ACAT2 |
|  |  |  | CXCL12 |
|  |  |  | TRPM1 |
|  |  |  | NTN1 |
|  |  |  | IL36A |
|  |  |  | TET2 |
|  |  |  | COL2A1 |
|  |  |  | IDI1 |
|  |  |  | LHFPL1 |
|  |  |  | RPA1 |
|  |  |  | EEF1E1 |
|  |  |  | FCGR2C |
|  |  |  | CA10 |
|  |  |  | RRM2 |
|  |  |  | HK2 |
|  |  |  | MDK |
|  |  |  | MRE11 |
|  |  |  | AKAP9 |
|  |  |  | METRNL |
|  |  |  | EPHX1 |
|  |  |  | CD63 |
|  |  |  | SLC20A1 |
|  |  |  | CX3CL1 |
|  |  |  | AIMP1 |
|  |  |  | LDB2 |
|  |  |  | FEN1 |
|  |  |  | PPIF |
|  |  |  | SPTB |
|  |  |  | MIR124-3 |
|  |  |  | EFEMP2 |
|  |  |  | ICAM1 |
|  |  |  | CXCL9 |
|  |  |  | RECK |
|  |  |  | CAV1 |
|  |  |  | GNPDA2 |
|  |  |  | H3-2 |
|  |  |  | DGAT2 |
|  |  |  | BCL2L1 |
|  |  |  | DNAH9 |
|  |  |  | RAPGEF3 |
|  |  |  | HELLS |
|  |  |  | TNFRSF1B |
|  |  |  | LGALS9 |
|  |  |  | MIR495 |
|  |  |  | MAPK3 |
|  |  |  | SLC20A2 |
|  |  |  | AEBP1 |
|  |  |  | PAQR5 |
|  |  |  | SLC16A3 |
|  |  |  | ANKRD2 |
|  |  |  | HMGCL |
|  |  |  | SLC9A1 |
|  |  |  | SLC12A5-AS1 |
|  |  |  | TNNI3K |
|  |  |  | CACNA1S |
|  |  |  | HDAC2 |
|  |  |  | MSI2 |
|  |  |  | CORIN |
|  |  |  | PRR7 |
|  |  |  | MRTFA |
|  |  |  | SPARCL1 |
|  |  |  | NEXN |
|  |  |  | CTSL |
|  |  |  | THBS2 |
|  |  |  | PLEC |
|  |  |  | IL6ST |
|  |  |  | LTF |
|  |  |  | TPO |
|  |  |  | ANK1 |
|  |  |  | ETS1 |
|  |  |  | MLST8 |
|  |  |  | AGRP |
|  |  |  | GATA5 |
|  |  |  | MIR181A1 |
|  |  |  | HSP90B1 |
|  |  |  | APCS |
|  |  |  | CXCL1 |
|  |  |  | MIR92B |
|  |  |  | ABCB4 |
|  |  |  | MIR100 |
|  |  |  | SGMS2 |
|  |  |  | MXD3 |
|  |  |  | FAH |
|  |  |  | SFTPB |
|  |  |  | VAMP3 |
|  |  |  | RCE1 |
|  |  |  | HNF4A |
|  |  |  | CNTNAP2 |
|  |  |  | MYH7 |
|  |  |  | TNFRSF10B |
|  |  |  | lnc-PIK3CG-6 |
|  |  |  | SH2B3 |
|  |  |  | TCIRG1 |
|  |  |  | LOC110973015 |
|  |  |  | MIR503 |
|  |  |  | RELA |
|  |  |  | HIF1A |
|  |  |  | TRAF1 |
|  |  |  | RYR2 |
|  |  |  | IRF1-AS1 |
|  |  |  | GRN |
|  |  |  | DBN1 |
|  |  |  | MIR133B |
|  |  |  | SLC2A4 |
|  |  |  | AP3D1 |
|  |  |  | PTS |
|  |  |  | KCNK13 |
|  |  |  | MAP2 |
|  |  |  | ADCY6 |
|  |  |  | TNFRSF11A |
|  |  |  | NOS3 |
|  |  |  | TGFA |
|  |  |  | NPR2 |
|  |  |  | MMP17 |
|  |  |  | ANPEP |
|  |  |  | LOC100507053 |
|  |  |  | PROM1 |
|  |  |  | MIR10B |
|  |  |  | PAOD1 |
|  |  |  | CFTR |
|  |  |  | TUSC1 |
|  |  |  | GSTM3 |
|  |  |  | KLF14 |
|  |  |  | PTPRA |
|  |  |  | TBX20 |
|  |  |  | KCNA5 |
|  |  |  | FLRT2 |
|  |  |  | HSF1 |
|  |  |  | WRN |
|  |  |  | NFATC2 |
|  |  |  | NOMO2 |
|  |  |  | NPPC |
|  |  |  | CYLD |
|  |  |  | ZEB2 |
|  |  |  | TBCAP1 |
|  |  |  | RHBDF2 |
|  |  |  | ABCG5 |
|  |  |  | MIR16-1 |
|  |  |  | USF1 |
|  |  |  | JMJD1C |
|  |  |  | NFKBIB |
|  |  |  | CHRM2 |
|  |  |  | DKK1 |
|  |  |  | CAPN5 |
|  |  |  | CYCSP14 |
|  |  |  | KCNH5 |
|  |  |  | NR1H4 |
|  |  |  | RRM1 |
|  |  |  | ATP6V1G2-DDX39B |
|  |  |  | OLFML2B |
|  |  |  | ERCC6 |
|  |  |  | NOS1AP |
|  |  |  | PRMT3 |
|  |  |  | NOTCH4 |
|  |  |  | INPP5D |
|  |  |  | CLEC12A |
|  |  |  | CDKN1C |
|  |  |  | ABCA4 |
|  |  |  | SIRT3 |
|  |  |  | AQP1 |
|  |  |  | OPRM1 |
|  |  |  | GFAP |
|  |  |  | DPP8 |
|  |  |  | CYP26B1 |
|  |  |  | ACTB |
|  |  |  | IL7R |
|  |  |  | MSRA |
|  |  |  | IFI35 |
|  |  |  | TLR4 |
|  |  |  | RGS9 |
|  |  |  | LECT2 |
|  |  |  | SRSF2 |
|  |  |  | BSCL2 |
|  |  |  | PCDH9 |
|  |  |  | CCL21 |
|  |  |  | ADAMTS13 |
|  |  |  | IL16 |
|  |  |  | MSTN |
|  |  |  | VCL |
|  |  |  | UBA52P6 |
|  |  |  | RPL31P26 |
|  |  |  | SERPINE1 |
|  |  |  | HFE |
|  |  |  | POU2F3 |
|  |  |  | CYP2C8 |
|  |  |  | BACH1 |
|  |  |  | MYO7A |
|  |  |  | MIR17 |
|  |  |  | VCAM1 |
|  |  |  | MIR19B1 |
|  |  |  | PTAFR |
|  |  |  | ACTG2 |
|  |  |  | ROBO2 |
|  |  |  | NOX1 |
|  |  |  | IFNGR1 |
|  |  |  | CSN1S1 |
|  |  |  | SRFBP1 |
|  |  |  | TSPAN6 |
|  |  |  | PRELID1 |
|  |  |  | RSAD2 |
|  |  |  | TGFBR2 |
|  |  |  | CDH23 |
|  |  |  | TLR9 |
|  |  |  | TTN |
|  |  |  | IL33 |
|  |  |  | RGS6 |
|  |  |  | ITGA11 |
|  |  |  | ZNF700 |
|  |  |  | WWTR1 |
|  |  |  | IFNA1 |
|  |  |  | HHIP |
|  |  |  | HLA-DRB1 |
|  |  |  | MEG3 |
|  |  |  | FGF3 |
|  |  |  | RAD51 |
|  |  |  | CLEC16A |
|  |  |  | ADH1B |
|  |  |  | DYRK1A |
|  |  |  | MIR146B |
|  |  |  | PEMT |
|  |  |  | SARDH |
|  |  |  | NFKBIE |
|  |  |  | ANGPTL2 |
|  |  |  | NIT2 |
|  |  |  | NOG |
|  |  |  | HBS1L |
|  |  |  | HLA-B |
|  |  |  | ATP5F1B |
|  |  |  | TCF7L2 |
|  |  |  | PKM |
|  |  |  | HSPA1B |
|  |  |  | HEY2 |
|  |  |  | GCG |
|  |  |  | piR-43107-137 |
|  |  |  | GPD2 |
|  |  |  | CXCL5 |
|  |  |  | SPRR3 |
|  |  |  | GSTO1 |
|  |  |  | DAOA |
|  |  |  | ENPP1 |
|  |  |  | SCNN1A |
|  |  |  | FABP4 |
|  |  |  | UNC13C |
|  |  |  | KCNE2 |
|  |  |  | RHOBTB3 |
|  |  |  | P2RX4 |
|  |  |  | CCND2 |
|  |  |  | CFB |
|  |  |  | PDZK1 |
|  |  |  | DDX58 |
|  |  |  | ADIPOR2 |
|  |  |  | UBIAD1 |
|  |  |  | FCN3 |
|  |  |  | PTPN3 |
|  |  |  | ABCA7 |
|  |  |  | TMEM258 |
|  |  |  | RPS27P1 |
|  |  |  | CES5A |
|  |  |  | ACTA2-AS1 |
|  |  |  | MIR362 |
|  |  |  | CAPN10 |
|  |  |  | CCR5 |
|  |  |  | MIR130B |
|  |  |  | SLC5A1 |
|  |  |  | FABP12 |
|  |  |  | PDLIM3 |
|  |  |  | ZNHIT3 |
|  |  |  | VEGFD |
|  |  |  | ZFHX3 |
|  |  |  | PCSK5 |
|  |  |  | GIMAP5 |
|  |  |  | NDUFA9 |
|  |  |  | CYP2B6 |
|  |  |  | FDXR |
|  |  |  | NES |
|  |  |  | PITX3 |
|  |  |  | PNLIPRP1 |
|  |  |  | EYA4 |
|  |  |  | PRKAB1 |
|  |  |  | PON2 |
|  |  |  | GPX3 |
|  |  |  | EDN2 |
|  |  |  | YAP1 |
|  |  |  | HPX |
|  |  |  | MALAT1 |
|  |  |  | KLK1 |
|  |  |  | L3MBTL3 |
|  |  |  | CLEC4A |
|  |  |  | MIF |
|  |  |  | MLXIPL |
|  |  |  | PIP5KL1 |
|  |  |  | C4BPB |
|  |  |  | MAP2K1 |
|  |  |  | GH1 |
|  |  |  | PFN1 |
|  |  |  | AIM2 |
|  |  |  | ARL15 |
|  |  |  | HPS5 |
|  |  |  | ANK3 |
|  |  |  | BCL3 |
|  |  |  | TIMP2 |
|  |  |  | CCN3 |
|  |  |  | ITGB3 |
|  |  |  | PKD2L1 |
|  |  |  | F2RL3 |
|  |  |  | BMPR1B |
|  |  |  | CLIP1 |
|  |  |  | CCL15 |
|  |  |  | GP5 |
|  |  |  | AAT2 |
|  |  |  | LTC4S |
|  |  |  | SEMA5A |
|  |  |  | P4HB |
|  |  |  | MIR217 |
|  |  |  | STC2 |
|  |  |  | TNXA |
|  |  |  | TLL1 |
|  |  |  | TMEM132B |
|  |  |  | COL6A1 |
|  |  |  | ZNF788P |
|  |  |  | OAZ1 |
|  |  |  | IL11 |
|  |  |  | NUMA1 |
|  |  |  | F12 |
|  |  |  | TCF7 |
|  |  |  | F11R |
|  |  |  | DTWD1 |
|  |  |  | HSD11B1 |
|  |  |  | KITLG |
|  |  |  | FOXD4 |
|  |  |  | MMRN1 |
|  |  |  | GSK3A |
|  |  |  | LCN2 |
|  |  |  | PI16 |
|  |  |  | SUMO4 |
|  |  |  | TXN |
|  |  |  | SPARC |
|  |  |  | ZNF326 |
|  |  |  | CTSH |
|  |  |  | COL5A1 |
|  |  |  | ACTA2 |
|  |  |  | BECN1 |
|  |  |  | TRPC4AP |
|  |  |  | ANKRD1 |
|  |  |  | IL20RB |
|  |  |  | SAA2 |
|  |  |  | ZNF592 |
|  |  |  | IFN1@ |
|  |  |  | LAMA4 |
|  |  |  | PTGS1 |
|  |  |  | RNF157 |
|  |  |  | SERPINC1 |
|  |  |  | NPS |
|  |  |  | CFP |
|  |  |  | F3 |
|  |  |  | MIR133A2 |
|  |  |  | PLEKHM2 |
|  |  |  | NR1I2 |
|  |  |  | CES4A |
|  |  |  | IGFBP4 |
|  |  |  | KCNJ5 |
|  |  |  | LMNA |
|  |  |  | NDUFA10 |
|  |  |  | CASQ2 |
|  |  |  | EIF3FP3 |
|  |  |  | APOA1 |
|  |  |  | PRICKLE1 |
|  |  |  | CCDC92 |
|  |  |  | KLF10 |
|  |  |  | FOXO3 |
|  |  |  | CD69 |
|  |  |  | LTA |
|  |  |  | TTPA |
|  |  |  | MYOZ1 |
|  |  |  | IFNB1 |
|  |  |  | PSEN1 |
|  |  |  | HULC |
|  |  |  | MIR499A |
|  |  |  | CYP46A1 |
|  |  |  | RAET1E |
|  |  |  | SLC2A9 |
|  |  |  | CDKN2A |
|  |  |  | PHOSPHO1 |
|  |  |  | LPAR1 |
|  |  |  | ETV6 |
|  |  |  | PMM2 |
|  |  |  | SCN2B |
|  |  |  | CEBPB |
|  |  |  | HAS1 |
|  |  |  | FMNL2 |
|  |  |  | TRPS1 |
|  |  |  | HMGA1 |
|  |  |  | RPS26P57 |
|  |  |  | CCL3 |
|  |  |  | lnc-SPC24-1 |
|  |  |  | TFPI2 |
|  |  |  | ATHS |
|  |  |  | STAG3L4 |
|  |  |  | ATG16L1 |
|  |  |  | ERN1 |
|  |  |  | CSF1R |
|  |  |  | JUN |
|  |  |  | TRA |
|  |  |  | IRAK4 |
|  |  |  | MYL2 |
|  |  |  | STK38 |
|  |  |  | ADAMTS5 |
|  |  |  | NOTCH3 |
|  |  |  | ATP2A2 |
|  |  |  | MYOM3 |
|  |  |  | ATIC |
|  |  |  | CXCL16 |
|  |  |  | NF2 |
|  |  |  | NRG1 |
|  |  |  | TREX1 |
|  |  |  | TNFSF14 |
|  |  |  | FBXO32 |
|  |  |  | TGFBI |
|  |  |  | AR |
|  |  |  | MYH7B |
|  |  |  | KCNN4 |
|  |  |  | LEXM |
|  |  |  | GATM |
|  |  |  | MHRT |
|  |  |  | CDKN3 |
|  |  |  | CDH4 |
|  |  |  | ARHGEF10 |
|  |  |  | JPH3 |
|  |  |  | CYP2J2 |
|  |  |  | MIR30C1 |
|  |  |  | HDC |
|  |  |  | PXK |
|  |  |  | HSPG2 |
|  |  |  | FLNA |
|  |  |  | HEYL |
|  |  |  | IL2RB |
|  |  |  | ADCY9 |
|  |  |  | NOTCH1 |
|  |  |  | BAX |
|  |  |  | CD274 |
|  |  |  | LRPAP1 |
|  |  |  | CP |
|  |  |  | IGHE |
|  |  |  | TM4SF5 |
|  |  |  | PDYN |
|  |  |  | CDKAL1 |
|  |  |  | GORASP1 |
|  |  |  | CDH5 |
|  |  |  | FKBP1A |
|  |  |  | PINX1 |
|  |  |  | CYP24A1 |
|  |  |  | SCML4 |
|  |  |  | EIF3G |
|  |  |  | ANO6 |
|  |  |  | SMARCAL1 |
|  |  |  | FOSL1 |
|  |  |  | GSTT1 |
|  |  |  | NPC1 |
|  |  |  | PRKCA |
|  |  |  | UGT1A3 |
|  |  |  | TRIB2 |
|  |  |  | PRMT7 |
|  |  |  | ALDH9A1 |
|  |  |  | CD40LG |
|  |  |  | AHSP |
|  |  |  | UGT1A5 |
|  |  |  | DNAH5 |
|  |  |  | SRF |
|  |  |  | GHSR |
|  |  |  | MAT2A |
|  |  |  | TUBB4A |
|  |  |  | CYP3A4 |
|  |  |  | TRPC3 |
|  |  |  | TMEM106B |
|  |  |  | KLRD1 |
|  |  |  | FMN2 |
|  |  |  | NPTXR |
|  |  |  | IL7 |
|  |  |  | SEMA3C |
|  |  |  | SCN9A |
|  |  |  | SCN5A |
|  |  |  | UBE2Q2P1 |
|  |  |  | GPC1 |
|  |  |  | ZNF383 |
|  |  |  | HOTTIP |
|  |  |  | PDGFC |
|  |  |  | PEPD |
|  |  |  | GLS2 |
|  |  |  | GATA4 |
|  |  |  | ATP2B1 |
|  |  |  | VHL |
|  |  |  | PRKAG1 |
|  |  |  | SMAD5 |
|  |  |  | IGF2BP1 |
|  |  |  | MIR212 |
|  |  |  | ANKRD23 |
|  |  |  | FGF23 |
|  |  |  | PRSS1 |
|  |  |  | PNPLA2 |
|  |  |  | HLX |
|  |  |  | PTGIS |
|  |  |  | PAH |
|  |  |  | CEACAM3 |
|  |  |  | MIR216A |
|  |  |  | SNX17 |
|  |  |  | HDAC6 |
|  |  |  | MIR33B |
|  |  |  | CYP2R1 |
|  |  |  | FBLN5 |
|  |  |  | MIR490 |
|  |  |  | TNNT1 |
|  |  |  | S100A9 |
|  |  |  | CSF2 |
|  |  |  | ITPR3 |
|  |  |  | BIRC3 |
|  |  |  | RPS6KB1 |
|  |  |  | GPLD1 |
|  |  |  | DNAJC19 |
|  |  |  | RNLS |
|  |  |  | SRY |
|  |  |  | HBA1 |
|  |  |  | DCAF8 |
|  |  |  | AQP7 |
|  |  |  | ADIPOQ |
|  |  |  | RXRB |
| coronary artery atherosclerosis vs familiar remedy | Atenolol/Pravastatin/coronary artery atherosclerosis | 1 | ABCB11 |
|  | Atenolol/Propranolol/coronary artery atherosclerosis | 2 | ADRB1 |
|  |  |  | ADRB2 |
|  | Pravastatin/coronary artery atherosclerosis | 6 | ABCC2 |
|  |  |  | SLCO1B1 |
|  |  |  | HMGCR |
|  |  |  | SLC22A11 |
|  |  |  | ABCG2 |
|  |  |  | HDAC2 |
|  | Propranolol/coronary artery atherosclerosis | 9 | CYP2C19 |
|  |  |  | MAOA |
|  |  |  | ORM1 |
|  |  |  | CYP1A1 |
|  |  |  | CYP3A5 |
|  |  |  | HTR1A |
|  |  |  | ADRB3 |
|  |  |  | CYP1A2 |
|  |  |  | CYP3A4 |
|  | Atenolol/Propranolol | 1 | CYP2D6 |
|  | coronary artery atherosclerosis | 2978 | MSRB1 |
|  |  |  | FSTL1 |
|  |  |  | SLC18A1 |
|  |  |  | RIT2 |
|  |  |  | CD44 |
|  |  |  | ABCA3 |
|  |  |  | MMP2 |
|  |  |  | KCNMA1 |
|  |  |  | KCNJ2 |
|  |  |  | VPS33A |
|  |  |  | PINK1 |
|  |  |  | RALYL |
|  |  |  | ITGA5 |
|  |  |  | NDUFS6 |
|  |  |  | UTP20 |
|  |  |  | MEFV |
|  |  |  | XDH |
|  |  |  | EDN1 |
|  |  |  | TKT |
|  |  |  | HSPB1 |
|  |  |  | ABCC6 |
|  |  |  | CXCR4 |
|  |  |  | DMP1 |
|  |  |  | CUBN |
|  |  |  | RMRP |
|  |  |  | ADAM12 |
|  |  |  | IRAK1 |
|  |  |  | MSBP1 |
|  |  |  | IGF1 |
|  |  |  | TMSB10 |
|  |  |  | G3BP1 |
|  |  |  | MIR20A |
|  |  |  | CXCL3 |
|  |  |  | LMAN1 |
|  |  |  | BCAR4 |
|  |  |  | GAS5 |
|  |  |  | MIRLET7G |
|  |  |  | NCF1C |
|  |  |  | GALNT3 |
|  |  |  | LDB3 |
|  |  |  | PAK1 |
|  |  |  | SUMO1 |
|  |  |  | MIR6886 |
|  |  |  | GP1BA |
|  |  |  | ANGPTL5 |
|  |  |  | MRPL10 |
|  |  |  | GDF15 |
|  |  |  | CYCS |
|  |  |  | EP300 |
|  |  |  | TLN1 |
|  |  |  | MMP7 |
|  |  |  | NKX2-5 |
|  |  |  | PRKCG |
|  |  |  | NOS2 |
|  |  |  | TRIB3 |
|  |  |  | CACNA2D1 |
|  |  |  | CCNB1 |
|  |  |  | HRG |
|  |  |  | PON3 |
|  |  |  | CDK14 |
|  |  |  | ERG |
|  |  |  | FAT4 |
|  |  |  | SOD2 |
|  |  |  | MIR15A |
|  |  |  | MSH3 |
|  |  |  | AQP9 |
|  |  |  | PDE1C |
|  |  |  | XK |
|  |  |  | SEMA4D |
|  |  |  | BAK1 |
|  |  |  | FGFR1 |
|  |  |  | AGPAT1 |
|  |  |  | MET |
|  |  |  | MTNR1B |
|  |  |  | HLA-DRB5 |
|  |  |  | PCSK1 |
|  |  |  | OSBPL8 |
|  |  |  | SERPINA12 |
|  |  |  | NR4A2 |
|  |  |  | PPCS |
|  |  |  | UGT1A1 |
|  |  |  | CXCL13 |
|  |  |  | SORL1 |
|  |  |  | MNS1 |
|  |  |  | NAT10 |
|  |  |  | FADS1 |
|  |  |  | LEP |
|  |  |  | CXADR |
|  |  |  | SERPINF1 |
|  |  |  | CACNG5 |
|  |  |  | MAPKAPK2 |
|  |  |  | DIP2A |
|  |  |  | OXT |
|  |  |  | CCL22 |
|  |  |  | SHC1 |
|  |  |  | PTPN22 |
|  |  |  | ITGA3 |
|  |  |  | HSD11B2 |
|  |  |  | ENSG00000280087 |
|  |  |  | BRCA1 |
|  |  |  | GGT2 |
|  |  |  | LINC02618 |
|  |  |  | CRYGC |
|  |  |  | S1PR2 |
|  |  |  | RUNX3 |
|  |  |  | LIMS2 |
|  |  |  | PRKCB |
|  |  |  | C5AR1 |
|  |  |  | SMO |
|  |  |  | A2M |
|  |  |  | MIR132 |
|  |  |  | UTRN |
|  |  |  | CNN1 |
|  |  |  | BAZ1B |
|  |  |  | APOBR |
|  |  |  | LMNB2 |
|  |  |  | ROCK2 |
|  |  |  | PALLD |
|  |  |  | GPC5 |
|  |  |  | MT-ATP6 |
|  |  |  | OSBPL9P2 |
|  |  |  | TLR1 |
|  |  |  | VKORC1 |
|  |  |  | LRGUK |
|  |  |  | ELANE |
|  |  |  | TEK |
|  |  |  | CD160 |
|  |  |  | COL1A1 |
|  |  |  | PDCD5 |
|  |  |  | SLC8A1 |
|  |  |  | GPR37L1 |
|  |  |  | HES1 |
|  |  |  | OGDH |
|  |  |  | MACROD1 |
|  |  |  | GSS |
|  |  |  | C1QTNF3 |
|  |  |  | NECTIN2 |
|  |  |  | CCN4 |
|  |  |  | SSTR2 |
|  |  |  | FOXP3 |
|  |  |  | BIRC5 |
|  |  |  | CHRNA5 |
|  |  |  | UTS2B |
|  |  |  | MIR624 |
|  |  |  | SELL |
|  |  |  | GNAS |
|  |  |  | ANKRD6 |
|  |  |  | LTBP4 |
|  |  |  | PYY |
|  |  |  | GHR |
|  |  |  | CCL19 |
|  |  |  | SIRT6 |
|  |  |  | GPR55 |
|  |  |  | MTRR |
|  |  |  | NCOR2 |
|  |  |  | CD38 |
|  |  |  | SERPINB9 |
|  |  |  | TNFRSF9 |
|  |  |  | CCNE1 |
|  |  |  | MS4A2 |
|  |  |  | PODN |
|  |  |  | BHMT |
|  |  |  | NPC2 |
|  |  |  | IL17RC |
|  |  |  | HS3ST1 |
|  |  |  | GUCY1A1 |
|  |  |  | ITGB1BP2 |
|  |  |  | RBPJ |
|  |  |  | GATA6 |
|  |  |  | APOE |
|  |  |  | PF4 |
|  |  |  | DSTN |
|  |  |  | ALDH2 |
|  |  |  | ST8SIA4 |
|  |  |  | DAPK3 |
|  |  |  | C3 |
|  |  |  | MRAP |
|  |  |  | TPM3 |
|  |  |  | HSPD1 |
|  |  |  | UGT1A12P |
|  |  |  | F2RL1 |
|  |  |  | PRDM16 |
|  |  |  | MIR378A |
|  |  |  | CDK1 |
|  |  |  | APOC4 |
|  |  |  | PTK2 |
|  |  |  | SHBG |
|  |  |  | FABP5 |
|  |  |  | HYOU1 |
|  |  |  | UGT1A10 |
|  |  |  | TUBB |
|  |  |  | MFSD11 |
|  |  |  | SORT1 |
|  |  |  | PLIN2 |
|  |  |  | PPM1K |
|  |  |  | MYH6 |
|  |  |  | RBFOX1 |
|  |  |  | BCHE |
|  |  |  | ENOSF1 |
|  |  |  | SERPING1 |
|  |  |  | TONSL |
|  |  |  | GCLM |
|  |  |  | NFKB2 |
|  |  |  | BCL2 |
|  |  |  | ARL13B |
|  |  |  | CAMTA1 |
|  |  |  | TNFRSF10A |
|  |  |  | KL |
|  |  |  | P4HA3 |
|  |  |  | SAMSN1 |
|  |  |  | ALCAM |
|  |  |  | PLCL2 |
|  |  |  | IL24 |
|  |  |  | TRAM1 |
|  |  |  | BDKRB1 |
|  |  |  | NDUFS4 |
|  |  |  | ID1 |
|  |  |  | PIK3CB |
|  |  |  | COL18A1 |
|  |  |  | ENSG00000243797 |
|  |  |  | TNXB |
|  |  |  | CARD8 |
|  |  |  | BCAM |
|  |  |  | PCK2 |
|  |  |  | FKRP |
|  |  |  | TOX3 |
|  |  |  | TF |
|  |  |  | HLA-DRA |
|  |  |  | GCK |
|  |  |  | THRB |
|  |  |  | RNF39 |
|  |  |  | MYBPC1 |
|  |  |  | ADRA2A |
|  |  |  | NPR1 |
|  |  |  | TERC |
|  |  |  | ABRAXAS2 |
|  |  |  | GSTM4 |
|  |  |  | TMEM59 |
|  |  |  | SEC23IP |
|  |  |  | F7 |
|  |  |  | PDCL2 |
|  |  |  | EDNRA |
|  |  |  | MIR9-1 |
|  |  |  | CCT8 |
|  |  |  | POLG |
|  |  |  | IRS1 |
|  |  |  | GHRL |
|  |  |  | SLC17A3 |
|  |  |  | MFN2 |
|  |  |  | MICU1 |
|  |  |  | LOC111365141 |
|  |  |  | ESRRA |
|  |  |  | ATR |
|  |  |  | GNA11 |
|  |  |  | FOXB1 |
|  |  |  | EBPL |
|  |  |  | ARSH |
|  |  |  | TACR1 |
|  |  |  | CAPN1 |
|  |  |  | RYR1 |
|  |  |  | LTBP1 |
|  |  |  | MT3 |
|  |  |  | PPBP |
|  |  |  | ZNF687 |
|  |  |  | HTR3A |
|  |  |  | OXTR |
|  |  |  | ACTC1 |
|  |  |  | SDC2 |
|  |  |  | MAPK10 |
|  |  |  | ABI2 |
|  |  |  | CACNB1 |
|  |  |  | QKI |
|  |  |  | ATF1 |
|  |  |  | IL5 |
|  |  |  | FERMT3 |
|  |  |  | AGT |
|  |  |  | PTGES |
|  |  |  | LRRC10 |
|  |  |  | MFAP5 |
|  |  |  | FGF1 |
|  |  |  | CHRM1 |
|  |  |  | IL6R |
|  |  |  | CST3 |
|  |  |  | CHI3L1 |
|  |  |  | LEMD2 |
|  |  |  | CLEC3B |
|  |  |  | CIITA |
|  |  |  | NOMO1 |
|  |  |  | CRP |
|  |  |  | GLA |
|  |  |  | NEBL |
|  |  |  | HSPA8 |
|  |  |  | IMMT |
|  |  |  | CEL |
|  |  |  | SELENOP |
|  |  |  | NEU1 |
|  |  |  | TARID |
|  |  |  | AGGF1 |
|  |  |  | PCCB |
|  |  |  | ENHO |
|  |  |  | NPHS1 |
|  |  |  | SPRY2 |
|  |  |  | GSTP1 |
|  |  |  | LTBR |
|  |  |  | CCR3 |
|  |  |  | NBPF3 |
|  |  |  | MIR340 |
|  |  |  | HBG2 |
|  |  |  | CYP7A1 |
|  |  |  | STAT5A |
|  |  |  | AGTRAP |
|  |  |  | SRD5A2 |
|  |  |  | MIR122 |
|  |  |  | ARHGEF38 |
|  |  |  | AP3B1 |
|  |  |  | MIR206 |
|  |  |  | KISS1R |
|  |  |  | NRG4 |
|  |  |  | MT-ND2 |
|  |  |  | IL5RA |
|  |  |  | TIAM1 |
|  |  |  | RGS14 |
|  |  |  | IL18BP |
|  |  |  | SMPD2 |
|  |  |  | MIR182 |
|  |  |  | FHL1 |
|  |  |  | TNFSF12 |
|  |  |  | CSRP3 |
|  |  |  | MTR |
|  |  |  | FN1 |
|  |  |  | LPIN3 |
|  |  |  | AHR |
|  |  |  | DHFR |
|  |  |  | ALPP |
|  |  |  | PPP1R12A |
|  |  |  | VSIR |
|  |  |  | PHACTR1 |
|  |  |  | GPR132 |
|  |  |  | ATP1A1 |
|  |  |  | LOXL1 |
|  |  |  | MIR144 |
|  |  |  | NFE2L2 |
|  |  |  | IL23R |
|  |  |  | NRF1 |
|  |  |  | SORCS2 |
|  |  |  | NR1I3 |
|  |  |  | ACVR2A |
|  |  |  | KLF4 |
|  |  |  | YWHAZ |
|  |  |  | KLKB1 |
|  |  |  | GAA |
|  |  |  | IFI27 |
|  |  |  | HDAC3 |
|  |  |  | SCN1A |
|  |  |  | HP |
|  |  |  | MAGOHB |
|  |  |  | MED18 |
|  |  |  | ECE1 |
|  |  |  | PARG |
|  |  |  | MT-LIPCAR |
|  |  |  | LIPI |
|  |  |  | UIMC1 |
|  |  |  | MIR29B1 |
|  |  |  | BMP6 |
|  |  |  | CLTCL1 |
|  |  |  | TRB |
|  |  |  | ALG6 |
|  |  |  | NPPA |
|  |  |  | TBX21 |
|  |  |  | PPARGC1A |
|  |  |  | TNFAIP6 |
|  |  |  | FMN1 |
|  |  |  | SSRP1 |
|  |  |  | CCL26 |
|  |  |  | BLOC1S5-TXNDC5 |
|  |  |  | SNAI1 |
|  |  |  | BRD4 |
|  |  |  | CD151 |
|  |  |  | CYP19A1 |
|  |  |  | MTHFD1 |
|  |  |  | HRH2 |
|  |  |  | PLA2G6 |
|  |  |  | CHDS3 |
|  |  |  | SOX9 |
|  |  |  | FTH1 |
|  |  |  | GC |
|  |  |  | IER3 |
|  |  |  | VSTM4 |
|  |  |  | MIR208B |
|  |  |  | PDGFRB |
|  |  |  | CEBPD |
|  |  |  | G6PC2 |
|  |  |  | OMD |
|  |  |  | WFDC21P |
|  |  |  | CSRP1 |
|  |  |  | CTSB |
|  |  |  | lnc-APOC1-1 |
|  |  |  | TMEM170A |
|  |  |  | ADCY3 |
|  |  |  | SVEP1 |
|  |  |  | FETUB |
|  |  |  | PXN |
|  |  |  | TNF |
|  |  |  | NTM |
|  |  |  | SIRT2 |
|  |  |  | C1QBP |
|  |  |  | NDUFB8 |
|  |  |  | GDF2 |
|  |  |  | NGFR |
|  |  |  | IKBKG |
|  |  |  | IL23A |
|  |  |  | TCF21 |
|  |  |  | NSD1 |
|  |  |  | JPH2 |
|  |  |  | C5 |
|  |  |  | SLC2A10 |
|  |  |  | EGF |
|  |  |  | TCP1 |
|  |  |  | GNRH1 |
|  |  |  | IL1A |
|  |  |  | CMA1 |
|  |  |  | DSCR10 |
|  |  |  | CACNA1H |
|  |  |  | PDGFD |
|  |  |  | FST |
|  |  |  | ERCC1 |
|  |  |  | CAV3 |
|  |  |  | KCND3 |
|  |  |  | CREBBP |
|  |  |  | LDLR |
|  |  |  | ITGB1 |
|  |  |  | AKR1C4 |
|  |  |  | PARK7 |
|  |  |  | RPN1 |
|  |  |  | PMVK |
|  |  |  | KLF2 |
|  |  |  | BRINP3 |
|  |  |  | COQ10A |
|  |  |  | SGK1 |
|  |  |  | COL5A2 |
|  |  |  | RPL21P42 |
|  |  |  | SELP |
|  |  |  | PGM1 |
|  |  |  | MYOZ2 |
|  |  |  | DLGAP1 |
|  |  |  | PGLYRP1 |
|  |  |  | TANK |
|  |  |  | RCAN1 |
|  |  |  | AOC1 |
|  |  |  | MAP1LC3B |
|  |  |  | NAB2 |
|  |  |  | UGT1A8 |
|  |  |  | THY1 |
|  |  |  | KIT |
|  |  |  | KRT8 |
|  |  |  | SHH |
|  |  |  | THPO |
|  |  |  | RPL29P27 |
|  |  |  | MIR210 |
|  |  |  | EIF2S1 |
|  |  |  | SCN4B |
|  |  |  | PPOX |
|  |  |  | HAND2 |
|  |  |  | JAK2 |
|  |  |  | ANTXR1 |
|  |  |  | CREB3 |
|  |  |  | RAB5A |
|  |  |  | TERF2 |
|  |  |  | TNFRSF1A |
|  |  |  | HERC6 |
|  |  |  | RPL7AP57 |
|  |  |  | CASP1 |
|  |  |  | ATG7 |
|  |  |  | ACADVL |
|  |  |  | LRP1B |
|  |  |  | ZBTB8OS |
|  |  |  | SPP1 |
|  |  |  | HBP1 |
|  |  |  | TNNT2 |
|  |  |  | ABCG8 |
|  |  |  | SFTPD |
|  |  |  | EPS15 |
|  |  |  | F2 |
|  |  |  | HCAR2 |
|  |  |  | XIAP |
|  |  |  | ZNF627 |
|  |  |  | EIF2B2 |
|  |  |  | WNT5A |
|  |  |  | APOA2 |
|  |  |  | TRPA1 |
|  |  |  | KALRN |
|  |  |  | SUCLA2P2 |
|  |  |  | ATP6V0A2 |
|  |  |  | FDFT1 |
|  |  |  | ENO2 |
|  |  |  | PTGS2 |
|  |  |  | BMP7 |
|  |  |  | OSM |
|  |  |  | B2M |
|  |  |  | MIRLET7E |
|  |  |  | OPN3 |
|  |  |  | LRP5 |
|  |  |  | OLR1 |
|  |  |  | DAGLB |
|  |  |  | NRAP |
|  |  |  | TRIM63 |
|  |  |  | NCF1 |
|  |  |  | CD2 |
|  |  |  | SPG7 |
|  |  |  | AGER |
|  |  |  | GALNT2 |
|  |  |  | CTNNB1 |
|  |  |  | CBS |
|  |  |  | MIR221 |
|  |  |  | MICA |
|  |  |  | ADH1C |
|  |  |  | SNTA1 |
|  |  |  | MIR141 |
|  |  |  | TLR5 |
|  |  |  | HAS2 |
|  |  |  | FCGR3A |
|  |  |  | CYP2C9 |
|  |  |  | DNMT3A |
|  |  |  | NT5E |
|  |  |  | UTS2R |
|  |  |  | IL18 |
|  |  |  | NPY |
|  |  |  | ITGA6 |
|  |  |  | RPL23P4 |
|  |  |  | BAG3 |
|  |  |  | OXA1L |
|  |  |  | CCND1 |
|  |  |  | CTSK |
|  |  |  | IGFBP2 |
|  |  |  | NOTCH2 |
|  |  |  | SIGLEC1 |
|  |  |  | PRKDC |
|  |  |  | CD93 |
|  |  |  | LOC100506472 |
|  |  |  | CCL4 |
|  |  |  | MSR1 |
|  |  |  | PDCD6IP |
|  |  |  | ADD1 |
|  |  |  | TOR2A |
|  |  |  | KDM6B |
|  |  |  | ATXN2 |
|  |  |  | SLC17A4 |
|  |  |  | RRAS |
|  |  |  | S100B |
|  |  |  | BMP2 |
|  |  |  | IPO5 |
|  |  |  | NPY1R |
|  |  |  | MGP |
|  |  |  | PCDH15 |
|  |  |  | GYPA |
|  |  |  | TRPC1 |
|  |  |  | SOST |
|  |  |  | ESR1 |
|  |  |  | SURF4 |
|  |  |  | KCNN3 |
|  |  |  | SST |
|  |  |  | ERCC8 |
|  |  |  | PRF1 |
|  |  |  | MAPK7 |
|  |  |  | RRAGC |
|  |  |  | GPX4 |
|  |  |  | ACE2 |
|  |  |  | FBXO3 |
|  |  |  | GCH1 |
|  |  |  | CMKLR1 |
|  |  |  | TYMS |
|  |  |  | DGKQ |
|  |  |  | SCNN1B |
|  |  |  | FMOD |
|  |  |  | IRS2 |
|  |  |  | SIK2 |
|  |  |  | SRRT |
|  |  |  | PDE4D |
|  |  |  | EPHB3 |
|  |  |  | TRPC4 |
|  |  |  | AAT1 |
|  |  |  | RAMP3 |
|  |  |  | CNTN5 |
|  |  |  | ESRRB |
|  |  |  | LRIG1 |
|  |  |  | HRAS |
|  |  |  | NPNT |
|  |  |  | HCG27 |
|  |  |  | TNFRSF10C |
|  |  |  | PDE3A |
|  |  |  | TRHDE |
|  |  |  | ITGB2 |
|  |  |  | SELENOS |
|  |  |  | XYLT2 |
|  |  |  | EPB41L3 |
|  |  |  | MIR361 |
|  |  |  | BCAR3 |
|  |  |  | CDKN2B |
|  |  |  | DHCR7 |
|  |  |  | PROS1 |
|  |  |  | PSMB8 |
|  |  |  | FGFR4 |
|  |  |  | CRAT |
|  |  |  | POR |
|  |  |  | SLC19A1 |
|  |  |  | LIMS1 |
|  |  |  | XRCC1 |
|  |  |  | SREBF2 |
|  |  |  | VEGFA |
|  |  |  | LAMA1 |
|  |  |  | APOL1 |
|  |  |  | ENPP2 |
|  |  |  | FHL5 |
|  |  |  | ACAN |
|  |  |  | TGFB1 |
|  |  |  | ITGA2 |
|  |  |  | GPR182 |
|  |  |  | MME |
|  |  |  | APEX1 |
|  |  |  | ACKR2 |
|  |  |  | MAST4 |
|  |  |  | LPAL2 |
|  |  |  | GAST |
|  |  |  | PSEN2 |
|  |  |  | UBE2L3 |
|  |  |  | MYC |
|  |  |  | ADRA1D |
|  |  |  | NR4A3 |
|  |  |  | MCTP2 |
|  |  |  | ARSA |
|  |  |  | LCMT2 |
|  |  |  | MBL1P |
|  |  |  | PTGIR |
|  |  |  | ENSG00000247287 |
|  |  |  | FOXE3 |
|  |  |  | SOCS3 |
|  |  |  | AGBL1 |
|  |  |  | CCNA2 |
|  |  |  | RNPC3 |
|  |  |  | DAG1 |
|  |  |  | WWOX |
|  |  |  | SLC9A3R2 |
|  |  |  | TIMP1 |
|  |  |  | GAPDHP28 |
|  |  |  | C19orf38 |
|  |  |  | ABCA12 |
|  |  |  | CHIT1 |
|  |  |  | PCSK2 |
|  |  |  | PI3 |
|  |  |  | MRC1 |
|  |  |  | MT-ND6 |
|  |  |  | VCP |
|  |  |  | PCSK9 |
|  |  |  | LAMA3 |
|  |  |  | BGN |
|  |  |  | GAL |
|  |  |  | COCH |
|  |  |  | RAVER1 |
|  |  |  | IL13 |
|  |  |  | CD86 |
|  |  |  | SRSF3 |
|  |  |  | SLC11A1 |
|  |  |  | ICAM2 |
|  |  |  | MIR21 |
|  |  |  | INTS12 |
|  |  |  | CTSF |
|  |  |  | PDXP |
|  |  |  | GSK3B |
|  |  |  | NFIA-AS1 |
|  |  |  | GPD1L |
|  |  |  | TNFAIP3 |
|  |  |  | PSRC1 |
|  |  |  | PLA2G10 |
|  |  |  | FOXO1 |
|  |  |  | GPT |
|  |  |  | BLK |
|  |  |  | PPP1R17 |
|  |  |  | APOM |
|  |  |  | IBSP |
|  |  |  | PKP2 |
|  |  |  | GLTPD2 |
|  |  |  | MMP1 |
|  |  |  | STAT1 |
|  |  |  | HLA-DQA1 |
|  |  |  | GCLC |
|  |  |  | IGFBP5 |
|  |  |  | COL8A1 |
|  |  |  | SERPINA4 |
|  |  |  | MROH5 |
|  |  |  | FKTN |
|  |  |  | MMP12 |
|  |  |  | NME4 |
|  |  |  | IL18RAP |
|  |  |  | CARD16 |
|  |  |  | BLVRB |
|  |  |  | TNFSF13 |
|  |  |  | HABP2 |
|  |  |  | VENTXP2 |
|  |  |  | DLL4 |
|  |  |  | MYOCD |
|  |  |  | FBXO15 |
|  |  |  | SLC2A1 |
|  |  |  | LSAMP |
|  |  |  | ATP2A3 |
|  |  |  | MKKS |
|  |  |  | PTGER4 |
|  |  |  | CD68 |
|  |  |  | CCR7 |
|  |  |  | SMARCA1 |
|  |  |  | KCNQ1 |
|  |  |  | RECQL5 |
|  |  |  | APOF |
|  |  |  | ADCY1 |
|  |  |  | NR2F2 |
|  |  |  | BCL2A1 |
|  |  |  | ANGPTL1 |
|  |  |  | RHO |
|  |  |  | GRID1 |
|  |  |  | FHOD3 |
|  |  |  | BANF1 |
|  |  |  | CR1 |
|  |  |  | PRDM1 |
|  |  |  | FAM13A |
|  |  |  | TOR1A |
|  |  |  | BSG |
|  |  |  | P2RY4 |
|  |  |  | TRPV1 |
|  |  |  | UCP2 |
|  |  |  | EFEMP1 |
|  |  |  | ZFYVE9 |
|  |  |  | MIR28 |
|  |  |  | TRIM24 |
|  |  |  | MIRLET7A1 |
|  |  |  | MOCS1 |
|  |  |  | PROC |
|  |  |  | PLXNA4 |
|  |  |  | SLC22A5 |
|  |  |  | GRIN3A |
|  |  |  | DOCK6 |
|  |  |  | FPR1 |
|  |  |  | SYP |
|  |  |  | GNLY |
|  |  |  | FAM223A |
|  |  |  | CCR4 |
|  |  |  | PTPRC |
|  |  |  | CCT7 |
|  |  |  | MYO1E |
|  |  |  | EEF1A2 |
|  |  |  | MIPEP |
|  |  |  | CD14 |
|  |  |  | ABCC9 |
|  |  |  | NDE1 |
|  |  |  | PPARD |
|  |  |  | ANXA2 |
|  |  |  | MCF2L |
|  |  |  | SPEG |
|  |  |  | ARHGAP20 |
|  |  |  | RPL35AP15 |
|  |  |  | LGALS2 |
|  |  |  | NR3C2 |
|  |  |  | MIR134 |
|  |  |  | C4B |
|  |  |  | GSR |
|  |  |  | INPPL1 |
|  |  |  | CACNA1C |
|  |  |  | IL19 |
|  |  |  | HDLCQ2 |
|  |  |  | GGT1 |
|  |  |  | EIF2AK2 |
|  |  |  | WNT1 |
|  |  |  | CCL11 |
|  |  |  | LMO7 |
|  |  |  | PSMD9 |
|  |  |  | RHOD |
|  |  |  | IDO1 |
|  |  |  | COL4A2 |
|  |  |  | KDM5B |
|  |  |  | CCL23 |
|  |  |  | LUM |
|  |  |  | OBSL1 |
|  |  |  | C1S |
|  |  |  | HMOX1 |
|  |  |  | SLC27A6 |
|  |  |  | FCGR2A |
|  |  |  | GLO1 |
|  |  |  | MC4R |
|  |  |  | GPIHBP1 |
|  |  |  | MIR195 |
|  |  |  | FAM126A |
|  |  |  | FOXP2 |
|  |  |  | TRPM7 |
|  |  |  | INS |
|  |  |  | FLT1 |
|  |  |  | PDIA2 |
|  |  |  | ALOX12 |
|  |  |  | RPLP0 |
|  |  |  | EIF4E |
|  |  |  | HOTAIR |
|  |  |  | EBI3 |
|  |  |  | SMAD2 |
|  |  |  | EFNA5 |
|  |  |  | RPL5P19 |
|  |  |  | ADGRE5 |
|  |  |  | MSBP2 |
|  |  |  | F2RL2 |
|  |  |  | IGF2BP2 |
|  |  |  | MMP3 |
|  |  |  | BGLAP |
|  |  |  | HTR4 |
|  |  |  | PRKCZ |
|  |  |  | EIF2B4 |
|  |  |  | IL20 |
|  |  |  | MIR590 |
|  |  |  | TRAF6 |
|  |  |  | CCL18 |
|  |  |  | AXL |
|  |  |  | LMNB1 |
|  |  |  | PNPLA3 |
|  |  |  | CNTLN |
|  |  |  | CD276 |
|  |  |  | PLD2 |
|  |  |  | F13A1 |
|  |  |  | SLC10A1 |
|  |  |  | GATA3 |
|  |  |  | LDLR-AS1-001 |
|  |  |  | TFAM |
|  |  |  | SYNE3 |
|  |  |  | OGN |
|  |  |  | FMR1 |
|  |  |  | LDLRAP1 |
|  |  |  | ANGPTL4 |
|  |  |  | HHEX |
|  |  |  | CXCR1 |
|  |  |  | SLC12A3 |
|  |  |  | C9orf72 |
|  |  |  | PLCH1 |
|  |  |  | ZPR1 |
|  |  |  | NAMPT |
|  |  |  | PTGER2 |
|  |  |  | MMP8 |
|  |  |  | ESAM |
|  |  |  | MIR34A |
|  |  |  | FOXC2 |
|  |  |  | NGEF |
|  |  |  | HDAC4 |
|  |  |  | GDNF |
|  |  |  | CTNNA1 |
|  |  |  | ZNF717 |
|  |  |  | MIR497 |
|  |  |  | MIR222 |
|  |  |  | TRPC5 |
|  |  |  | SCGB1A1 |
|  |  |  | NTRK2 |
|  |  |  | CCDC3 |
|  |  |  | ADCYAP1 |
|  |  |  | MFGE8 |
|  |  |  | GSTM1 |
|  |  |  | CRH |
|  |  |  | IL10 |
|  |  |  | PRSS2 |
|  |  |  | CES3 |
|  |  |  | WDR33 |
|  |  |  | ACP5 |
|  |  |  | lnc-KDM5D-4 |
|  |  |  | AMFR |
|  |  |  | LIAS |
|  |  |  | MAPK1 |
|  |  |  | GOLGB1 |
|  |  |  | PGF |
|  |  |  | F11-AS1 |
|  |  |  | CORT |
|  |  |  | TLR10 |
|  |  |  | CHD7 |
|  |  |  | HNRNPA2B1 |
|  |  |  | SLC30A8 |
|  |  |  | PYGB |
|  |  |  | GADD45A |
|  |  |  | TSC1 |
|  |  |  | MIR214 |
|  |  |  | OARD1 |
|  |  |  | IAPP |
|  |  |  | ALOX5AP |
|  |  |  | LRP1 |
|  |  |  | POSTN |
|  |  |  | GP1BB |
|  |  |  | ANKH |
|  |  |  | RIPK3 |
|  |  |  | POM121L3P |
|  |  |  | FTO |
|  |  |  | CYP27B1 |
|  |  |  | SAMHD1 |
|  |  |  | PPP3R1 |
|  |  |  | LDLR-AS1 |
|  |  |  | PAX6 |
|  |  |  | SCG2 |
|  |  |  | ZC3HC1 |
|  |  |  | IGF2-AS |
|  |  |  | MMP14 |
|  |  |  | PLAT |
|  |  |  | SF3A1 |
|  |  |  | MDM2 |
|  |  |  | TAP2 |
|  |  |  | NETO1 |
|  |  |  | TNFSF11 |
|  |  |  | THSD7A |
|  |  |  | PTPRF |
|  |  |  | BLVRA |
|  |  |  | ICMT |
|  |  |  | GNAI1 |
|  |  |  | AKT2 |
|  |  |  | EGFR |
|  |  |  | PPFIA2 |
|  |  |  | IKBKB |
|  |  |  | SLC25A4 |
|  |  |  | MYL3 |
|  |  |  | THBS4 |
|  |  |  | TNPO1 |
|  |  |  | ENOX1 |
|  |  |  | MYLK |
|  |  |  | CYP4F2 |
|  |  |  | SOD1 |
|  |  |  | B4GALNT3 |
|  |  |  | PRKD2 |
|  |  |  | MIR130A |
|  |  |  | SIRT5 |
|  |  |  | CXCR3 |
|  |  |  | CYSLTR1 |
|  |  |  | MIRLET7B |
|  |  |  | CTLA4 |
|  |  |  | CCNB2 |
|  |  |  | NR4A1 |
|  |  |  | EMC10 |
|  |  |  | PLA2G2D |
|  |  |  | WNT2 |
|  |  |  | GLMN |
|  |  |  | SIRPA |
|  |  |  | LYVE1 |
|  |  |  | CSMD2 |
|  |  |  | ERBB2 |
|  |  |  | APOH |
|  |  |  | CREB1 |
|  |  |  | PDX1 |
|  |  |  | ADAMTS8 |
|  |  |  | SMAD1 |
|  |  |  | TLR7 |
|  |  |  | STAB1 |
|  |  |  | MXRA7 |
|  |  |  | SRD5A1 |
|  |  |  | LGALS3 |
|  |  |  | UGT1A7 |
|  |  |  | CFL2 |
|  |  |  | STEAP1B |
|  |  |  | FUT3 |
|  |  |  | CLEC7A |
|  |  |  | DHX38 |
|  |  |  | HSPA14 |
|  |  |  | KCNA3 |
|  |  |  | DCBLD1 |
|  |  |  | PDGFA |
|  |  |  | IL17F |
|  |  |  | ZHX2 |
|  |  |  | CFL1 |
|  |  |  | IL4 |
|  |  |  | CD28 |
|  |  |  | SELPLG |
|  |  |  | DIABLO |
|  |  |  | SMARCA4 |
|  |  |  | BAZ2B |
|  |  |  | NAT2 |
|  |  |  | LRP8 |
|  |  |  | CNTN1 |
|  |  |  | MASP2 |
|  |  |  | AGL |
|  |  |  | GBP1 |
|  |  |  | MIR30D |
|  |  |  | NISCH |
|  |  |  | DEFA1 |
|  |  |  | LOC106728418 |
|  |  |  | F13B |
|  |  |  | TMEM43 |
|  |  |  | AKAP12 |
|  |  |  | CLPTM1L |
|  |  |  | MEOX2 |
|  |  |  | HLA-DPB1 |
|  |  |  | STAT5B |
|  |  |  | PRPF8 |
|  |  |  | ZNF213-AS1 |
|  |  |  | MIR93 |
|  |  |  | CEP85L |
|  |  |  | MIR224 |
|  |  |  | CD74 |
|  |  |  | HSPA12B |
|  |  |  | LIPG |
|  |  |  | G6PD |
|  |  |  | KLF5 |
|  |  |  | APOD |
|  |  |  | TRPV4 |
|  |  |  | NEK8 |
|  |  |  | PDE5A |
|  |  |  | SCARA3 |
|  |  |  | NOX5 |
|  |  |  | ENSG00000267052 |
|  |  |  | SULT1A3 |
|  |  |  | MAPK8 |
|  |  |  | CPA3 |
|  |  |  | KCNJ12 |
|  |  |  | ELOVL2 |
|  |  |  | C5AR2 |
|  |  |  | NRG3 |
|  |  |  | GPR35 |
|  |  |  | F10 |
|  |  |  | GSC2 |
|  |  |  | ACVR1 |
|  |  |  | COMP |
|  |  |  | HLA-DQA2 |
|  |  |  | STAR |
|  |  |  | F2R |
|  |  |  | THBS1 |
|  |  |  | MTAP |
|  |  |  | RFC1 |
|  |  |  | ACSL1 |
|  |  |  | H2AX |
|  |  |  | TCAP |
|  |  |  | ABL1 |
|  |  |  | NCOR1 |
|  |  |  | MIR143 |
|  |  |  | MOCOS |
|  |  |  | TXNRD1 |
|  |  |  | GZMB |
|  |  |  | FCGR3B |
|  |  |  | BMP1 |
|  |  |  | VIP |
|  |  |  | SP2 |
|  |  |  | CAPS2 |
|  |  |  | TAZ |
|  |  |  | AKR1B1 |
|  |  |  | CWC22 |
|  |  |  | MTFP1 |
|  |  |  | MMP26 |
|  |  |  | HLA-G |
|  |  |  | DIO2 |
|  |  |  | LRP2 |
|  |  |  | GPER1 |
|  |  |  | CYBB |
|  |  |  | MYDGF |
|  |  |  | HSP90AB1 |
|  |  |  | SEMA6D |
|  |  |  | CABIN1 |
|  |  |  | HSPE1 |
|  |  |  | MAPK13 |
|  |  |  | MYCN |
|  |  |  | SYNE1 |
|  |  |  | MTHFD1L |
|  |  |  | MVK |
|  |  |  | C2 |
|  |  |  | GCKR |
|  |  |  | APLN |
|  |  |  | DPP9 |
|  |  |  | NCOA1 |
|  |  |  | GUSB |
|  |  |  | HRH1 |
|  |  |  | DNM2 |
|  |  |  | PLA2G7 |
|  |  |  | CCL1 |
|  |  |  | TERT |
|  |  |  | CD247 |
|  |  |  | NEURL1 |
|  |  |  | KRT18 |
|  |  |  | CEP19 |
|  |  |  | SOCS1 |
|  |  |  | MIR155 |
|  |  |  | CDK5RAP3 |
|  |  |  | GGCX |
|  |  |  | HTRA1 |
|  |  |  | IFT88 |
|  |  |  | RNF111 |
|  |  |  | PTH |
|  |  |  | TNFRSF14 |
|  |  |  | TARDBP |
|  |  |  | PJA1 |
|  |  |  | RB1 |
|  |  |  | CETP |
|  |  |  | APOC2 |
|  |  |  | PTPN6 |
|  |  |  | HADHB |
|  |  |  | ADAR |
|  |  |  | ARMS2 |
|  |  |  | PSMD4 |
|  |  |  | DNAH10 |
|  |  |  | P2RX1 |
|  |  |  | CACNB2 |
|  |  |  | NUMB |
|  |  |  | MIR25 |
|  |  |  | CD70 |
|  |  |  | SLC29A1 |
|  |  |  | GLRX |
|  |  |  | SAA1 |
|  |  |  | PF4V1 |
|  |  |  | ST3GAL4 |
|  |  |  | REG1A |
|  |  |  | SRC |
|  |  |  | HBB |
|  |  |  | HEY1 |
|  |  |  | KNG1 |
|  |  |  | ITGAX |
|  |  |  | CASR |
|  |  |  | MIR30E |
|  |  |  | GPAM |
|  |  |  | CDK2 |
|  |  |  | CANT1 |
|  |  |  | LIN9 |
|  |  |  | MTPN |
|  |  |  | TFAP2A |
|  |  |  | NFS1 |
|  |  |  | ADTRP |
|  |  |  | CDC37 |
|  |  |  | NUP107 |
|  |  |  | CHDS8 |
|  |  |  | PDE4B |
|  |  |  | PGR |
|  |  |  | RUNX2 |
|  |  |  | MT-CO1 |
|  |  |  | SPATA6L |
|  |  |  | GRK4 |
|  |  |  | LRP12 |
|  |  |  | SEC14L2 |
|  |  |  | FGF10 |
|  |  |  | IL1RN |
|  |  |  | ODC1 |
|  |  |  | MIR296 |
|  |  |  | MIR9-2 |
|  |  |  | ADORA2B |
|  |  |  | MAFB |
|  |  |  | PHEX |
|  |  |  | SOX6 |
|  |  |  | ADIPOR1 |
|  |  |  | TFPI |
|  |  |  | SNF8 |
|  |  |  | SGCD |
|  |  |  | SMTN |
|  |  |  | CYP17A1 |
|  |  |  | CA3 |
|  |  |  | TWIST2 |
|  |  |  | MIR22 |
|  |  |  | NAB1 |
|  |  |  | CD209 |
|  |  |  | TNNI1 |
|  |  |  | ENO1 |
|  |  |  | CXCL10 |
|  |  |  | TRPM3 |
|  |  |  | P4HA1 |
|  |  |  | GOSR2 |
|  |  |  | RPL7AP58 |
|  |  |  | BFSP1 |
|  |  |  | CHDS4 |
|  |  |  | PLG |
|  |  |  | LOC102724465 |
|  |  |  | ATP6AP2 |
|  |  |  | TYR |
|  |  |  | COL4A1 |
|  |  |  | SEZ6L |
|  |  |  | VCAN |
|  |  |  | ADAMTS3 |
|  |  |  | RPL21P108 |
|  |  |  | LEKR1 |
|  |  |  | MKI67 |
|  |  |  | TSLP |
|  |  |  | DNAH8 |
|  |  |  | STS |
|  |  |  | IL4R |
|  |  |  | BTNL2 |
|  |  |  | CCL5 |
|  |  |  | ADAM8 |
|  |  |  | TGIF1 |
|  |  |  | PEX5 |
|  |  |  | TP53 |
|  |  |  | MAP1LC3A |
|  |  |  | piR-56133-114 |
|  |  |  | CDH7 |
|  |  |  | LINC-ROR |
|  |  |  | HNRNPA1P10 |
|  |  |  | ENTPD1 |
|  |  |  | CCDC157 |
|  |  |  | MIR140 |
|  |  |  | JUP |
|  |  |  | STK11 |
|  |  |  | MX1 |
|  |  |  | SAA4 |
|  |  |  | PLPP3 |
|  |  |  | LRRC18 |
|  |  |  | EXOSC4 |
|  |  |  | VASP |
|  |  |  | LOX |
|  |  |  | RELB |
|  |  |  | NLRP1 |
|  |  |  | BUD13 |
|  |  |  | HNF1B |
|  |  |  | ACP1 |
|  |  |  | TGFBR3 |
|  |  |  | CCN1 |
|  |  |  | UMOD |
|  |  |  | CASP9 |
|  |  |  | MAP3K7 |
|  |  |  | PDCD1 |
|  |  |  | NBL1 |
|  |  |  | SRA1 |
|  |  |  | PRMT5P1 |
|  |  |  | DYNC2LI1 |
|  |  |  | CDKN1A |
|  |  |  | IL32 |
|  |  |  | LPL |
|  |  |  | LAMP2 |
|  |  |  | CAT |
|  |  |  | LOC102723692 |
|  |  |  | HCN4 |
|  |  |  | MARS1 |
|  |  |  | ATXN1 |
|  |  |  | LIPC |
|  |  |  | CPOX |
|  |  |  | GSTZ1 |
|  |  |  | CD84 |
|  |  |  | APH1B |
|  |  |  | RASA1 |
|  |  |  | ADAM17 |
|  |  |  | LYZ |
|  |  |  | F5 |
|  |  |  | CLEC5A |
|  |  |  | TPM2 |
|  |  |  | ELAVL1 |
|  |  |  | CPS1 |
|  |  |  | LIPJ |
|  |  |  | PIAS4 |
|  |  |  | NCOA2 |
|  |  |  | GATA2 |
|  |  |  | ABCA2 |
|  |  |  | SLC2A2 |
|  |  |  | ATP10D |
|  |  |  | CCDC71L |
|  |  |  | HRC |
|  |  |  | CELSR2 |
|  |  |  | LRG1 |
|  |  |  | CFLAR |
|  |  |  | PRKCH |
|  |  |  | PIK3R1 |
|  |  |  | GRP |
|  |  |  | CACNG1 |
|  |  |  | PDGFRA |
|  |  |  | CSTB |
|  |  |  | DMD |
|  |  |  | ADM |
|  |  |  | ANKRD30A |
|  |  |  | MIR150 |
|  |  |  | GTF2E2 |
|  |  |  | FCGR1A |
|  |  |  | FPR2 |
|  |  |  | DLC1 |
|  |  |  | CD47 |
|  |  |  | ACP6 |
|  |  |  | MIR199A1 |
|  |  |  | PPP1R1A |
|  |  |  | KCNQ3 |
|  |  |  | SLC22A12 |
|  |  |  | SPRY4 |
|  |  |  | MXD1 |
|  |  |  | IFIH1 |
|  |  |  | LIPK |
|  |  |  | HADHA |
|  |  |  | CHURC1 |
|  |  |  | XRCC5 |
|  |  |  | CFDP1 |
|  |  |  | AHSG |
|  |  |  | POU5F1 |
|  |  |  | HDAC1 |
|  |  |  | USO1 |
|  |  |  | MASP1 |
|  |  |  | SHOX |
|  |  |  | RPL32P12 |
|  |  |  | FBXO38 |
|  |  |  | ICOS |
|  |  |  | NFKB1 |
|  |  |  | SIRT1 |
|  |  |  | TNFRSF6B |
|  |  |  | ABCC1 |
|  |  |  | CAV2 |
|  |  |  | MIA3 |
|  |  |  | SEMA3F |
|  |  |  | MIR27B |
|  |  |  | UGT1A |
|  |  |  | IL1B |
|  |  |  | MIR505 |
|  |  |  | REN |
|  |  |  | CHRM3 |
|  |  |  | G6PC |
|  |  |  | NEK9 |
|  |  |  | ACTA1 |
|  |  |  | TOMM40 |
|  |  |  | CPE |
|  |  |  | CXCR6 |
|  |  |  | HPSE |
|  |  |  | IGF2R |
|  |  |  | DMWD |
|  |  |  | GSTM5 |
|  |  |  | FGF21 |
|  |  |  | LCAT |
|  |  |  | MIR196A2 |
|  |  |  | IL12B |
|  |  |  | ST6GAL1 |
|  |  |  | APOA4 |
|  |  |  | POMC |
|  |  |  | IL9 |
|  |  |  | ZMPSTE24 |
|  |  |  | APEX2 |
|  |  |  | SLC24A3 |
|  |  |  | RPL21P41 |
|  |  |  | TXNL4B |
|  |  |  | NLRP12 |
|  |  |  | piR-55948-110 |
|  |  |  | DNM1L |
|  |  |  | NPY4R |
|  |  |  | SH3GL2 |
|  |  |  | EGID-106632268 |
|  |  |  | MIR381 |
|  |  |  | ITGA7 |
|  |  |  | NFKBIA |
|  |  |  | CCDC159 |
|  |  |  | KCNIP2 |
|  |  |  | IGFBP3 |
|  |  |  | MPRIP |
|  |  |  | PSMA6 |
|  |  |  | SORBS3 |
|  |  |  | C4BPA |
|  |  |  | ALB |
|  |  |  | CPT1A |
|  |  |  | TERF1 |
|  |  |  | ALMS1 |
|  |  |  | HAMP |
|  |  |  | SREBF1 |
|  |  |  | DCN |
|  |  |  | RPL31P23 |
|  |  |  | NOD2 |
|  |  |  | IL21 |
|  |  |  | HTR2A |
|  |  |  | UCP3 |
|  |  |  | EPAS1 |
|  |  |  | SCNN1G |
|  |  |  | S100A12 |
|  |  |  | TREM1 |
|  |  |  | LOC106560211 |
|  |  |  | AMPD2 |
|  |  |  | ACTN1 |
|  |  |  | IFNAR2 |
|  |  |  | HLA-C |
|  |  |  | LINC00907 |
|  |  |  | TIMP4 |
|  |  |  | HSDL2 |
|  |  |  | LPIN1 |
|  |  |  | CX3CR1 |
|  |  |  | AKR1A1 |
|  |  |  | HACD4 |
|  |  |  | POU2F1 |
|  |  |  | HSPB7 |
|  |  |  | GAPDH |
|  |  |  | KISS1 |
|  |  |  | APC |
|  |  |  | TLR2 |
|  |  |  | GPX1 |
|  |  |  | CRLF2 |
|  |  |  | P2RX7 |
|  |  |  | DOCK7 |
|  |  |  | GNA12 |
|  |  |  | LIMK1 |
|  |  |  | KCNJ11 |
|  |  |  | RPL36AP23 |
|  |  |  | ABCC8 |
|  |  |  | MACROD2 |
|  |  |  | IL18R1 |
|  |  |  | BPI |
|  |  |  | GLB1 |
|  |  |  | BNC2 |
|  |  |  | SERPINA3 |
|  |  |  | GAS6 |
|  |  |  | RPL28P3 |
|  |  |  | TIMD4 |
|  |  |  | XKR4 |
|  |  |  | GSTM2 |
|  |  |  | STAB2 |
|  |  |  | NAT1 |
|  |  |  | PCOLCE2 |
|  |  |  | HCRT |
|  |  |  | HGFAC |
|  |  |  | PARP9 |
|  |  |  | MCPH1 |
|  |  |  | PEAR1 |
|  |  |  | MT2A |
|  |  |  | MMP10 |
|  |  |  | FABP2 |
|  |  |  | TCN2 |
|  |  |  | ACTG1 |
|  |  |  | CALM1 |
|  |  |  | TAS2R50 |
|  |  |  | HAVCR2 |
|  |  |  | PLAUR |
|  |  |  | MAP2K3 |
|  |  |  | FLT4 |
|  |  |  | NDP |
|  |  |  | CKM |
|  |  |  | SCAP |
|  |  |  | ESR2 |
|  |  |  | AGTR2 |
|  |  |  | MIR483 |
|  |  |  | LACTB |
|  |  |  | ADAMTSL1 |
|  |  |  | CSNK2A1 |
|  |  |  | ZBTB46 |
|  |  |  | EPO |
|  |  |  | COL1A2 |
|  |  |  | DTNA |
|  |  |  | SERPINA5 |
|  |  |  | HNRNPA1 |
|  |  |  | LY96 |
|  |  |  | AKT3 |
|  |  |  | KLK3 |
|  |  |  | IL1R1 |
|  |  |  | ATP5IF1 |
|  |  |  | CTTN |
|  |  |  | KCNJ8 |
|  |  |  | PROCR |
|  |  |  | ITIH4 |
|  |  |  | USP8 |
|  |  |  | GJA5 |
|  |  |  | ITGB5 |
|  |  |  | SLC17A5 |
|  |  |  | HGF |
|  |  |  | MRTFB |
|  |  |  | SOX18 |
|  |  |  | TYMP |
|  |  |  | SERPINA10 |
|  |  |  | INSIG1 |
|  |  |  | NR1D1 |
|  |  |  | CFD |
|  |  |  | HIF1A-AS1 |
|  |  |  | MYBPC2 |
|  |  |  | CELA2A |
|  |  |  | APOC1 |
|  |  |  | MIR29A |
|  |  |  | HSPA12A |
|  |  |  | ITGAL |
|  |  |  | PDE4A |
|  |  |  | NHLRC1 |
|  |  |  | CH25H |
|  |  |  | KAT2B |
|  |  |  | SDHA |
|  |  |  | CYP1B1 |
|  |  |  | ALPL |
|  |  |  | STEAP2-AS1 |
|  |  |  | COMT |
|  |  |  | SMAD6 |
|  |  |  | PDLIM5 |
|  |  |  | SPC24 |
|  |  |  | MIR26A1 |
|  |  |  | XYLT1 |
|  |  |  | ADAMTS7 |
|  |  |  | ROS1 |
|  |  |  | C1QTNF1 |
|  |  |  | VEGFB |
|  |  |  | AS3MT |
|  |  |  | MT-TL1 |
|  |  |  | MIR208A |
|  |  |  | ACKR3 |
|  |  |  | MIR4675 |
|  |  |  | HYAL1 |
|  |  |  | ZBTB17 |
|  |  |  | LTA4H |
|  |  |  | HDLBP |
|  |  |  | CD1C |
|  |  |  | S1PR3 |
|  |  |  | GNB3 |
|  |  |  | IFIT3 |
|  |  |  | AVP |
|  |  |  | HMGB1 |
|  |  |  | PDE3B |
|  |  |  | HOPX |
|  |  |  | MEF2C |
|  |  |  | RPS3AP9 |
|  |  |  | SOAT2 |
|  |  |  | LBR |
|  |  |  | STEAP1 |
|  |  |  | PGK1 |
|  |  |  | CCL13 |
|  |  |  | MYB |
|  |  |  | BAIAP2L1 |
|  |  |  | ESM1 |
|  |  |  | PFN3 |
|  |  |  | RCN3 |
|  |  |  | RPTOR |
|  |  |  | ERBB4 |
|  |  |  | RYR3 |
|  |  |  | MRPS36P3 |
|  |  |  | E2F8 |
|  |  |  | UGCG |
|  |  |  | DNASE1 |
|  |  |  | PTN |
|  |  |  | MIR1322 |
|  |  |  | LPP |
|  |  |  | IL12RB1 |
|  |  |  | DMRT1 |
|  |  |  | ADRA2C |
|  |  |  | DHCR24 |
|  |  |  | VAMP8 |
|  |  |  | NBN |
|  |  |  | MIR31 |
|  |  |  | HSPB6 |
|  |  |  | COX5A |
|  |  |  | MYL4 |
|  |  |  | EBLN1 |
|  |  |  | CXCL14 |
|  |  |  | AIFM1 |
|  |  |  | GSTA1 |
|  |  |  | SLC17A1 |
|  |  |  | CYBA |
|  |  |  | MIR146A |
|  |  |  | SOS1 |
|  |  |  | TGFB2 |
|  |  |  | PLAU |
|  |  |  | RAC1 |
|  |  |  | LAMA2 |
|  |  |  | PTEN |
|  |  |  | ANXA1 |
|  |  |  | RLBP1 |
|  |  |  | MAATS1 |
|  |  |  | ZNF385D |
|  |  |  | ID3 |
|  |  |  | TICAM1 |
|  |  |  | FCRL6 |
|  |  |  | MMP13 |
|  |  |  | PROZ |
|  |  |  | C8A |
|  |  |  | LGR6 |
|  |  |  | MTM1 |
|  |  |  | MIR33A |
|  |  |  | RFX5 |
|  |  |  | SLC6A4 |
|  |  |  | CALR3 |
|  |  |  | NAT8 |
|  |  |  | UCN |
|  |  |  | PTRHD1 |
|  |  |  | SMDT1 |
|  |  |  | SYNE2 |
|  |  |  | SLC22A4 |
|  |  |  | NAXE |
|  |  |  | KCNE1 |
|  |  |  | CD4 |
|  |  |  | DSP |
|  |  |  | ITLN1 |
|  |  |  | NOSIP |
|  |  |  | COL20A1 |
|  |  |  | TUG1 |
|  |  |  | IRF1 |
|  |  |  | PTK2B |
|  |  |  | UNC5B |
|  |  |  | SDHB |
|  |  |  | IRF2BP2 |
|  |  |  | MBL2 |
|  |  |  | NGF |
|  |  |  | APOA1-AS |
|  |  |  | ALPK3 |
|  |  |  | ALOX5 |
|  |  |  | NRP1 |
|  |  |  | ACAD8 |
|  |  |  | CDH13 |
|  |  |  | NLRP3 |
|  |  |  | GJA1 |
|  |  |  | PKD1 |
|  |  |  | IL6-AS1 |
|  |  |  | FASN |
|  |  |  | VEPH1 |
|  |  |  | PRDX6 |
|  |  |  | MYOM1 |
|  |  |  | TTF2 |
|  |  |  | AMBP |
|  |  |  | AKAP7 |
|  |  |  | AIDA |
|  |  |  | DOLK |
|  |  |  | ACVRL1 |
|  |  |  | CYCSP42 |
|  |  |  | ASCC3 |
|  |  |  | TGM2 |
|  |  |  | FCGR2B |
|  |  |  | NFATC4 |
|  |  |  | RXRA |
|  |  |  | MAP3K5 |
|  |  |  | GRM8 |
|  |  |  | PDLIM7 |
|  |  |  | SLC23A2 |
|  |  |  | MIR30A |
|  |  |  | NOS1 |
|  |  |  | GRK6 |
|  |  |  | TBK1 |
|  |  |  | BMPR1A |
|  |  |  | PRL |
|  |  |  | TIMP3 |
|  |  |  | LOC157273 |
|  |  |  | NR1H3 |
|  |  |  | ACACA |
|  |  |  | LYSMD4 |
|  |  |  | ABCG1 |
|  |  |  | MIR98 |
|  |  |  | SQSTM1 |
|  |  |  | MIR149 |
|  |  |  | DSCAML1 |
|  |  |  | NFATC1 |
|  |  |  | RAC2 |
|  |  |  | CD55 |
|  |  |  | PLN |
|  |  |  | MTHFR |
|  |  |  | ACAT1 |
|  |  |  | PRDM9 |
|  |  |  | CSGALNACT2 |
|  |  |  | PTPN11 |
|  |  |  | SMAD3 |
|  |  |  | ATF3 |
|  |  |  | TBX5 |
|  |  |  | TRDN |
|  |  |  | NAPG |
|  |  |  | MRVI1 |
|  |  |  | HMOX2 |
|  |  |  | C1QL3 |
|  |  |  | MPP7 |
|  |  |  | PLCG1 |
|  |  |  | SLC2A13 |
|  |  |  | FBN2 |
|  |  |  | ST3GAL1 |
|  |  |  | NEDD4 |
|  |  |  | RPS27AP2 |
|  |  |  | MIR370 |
|  |  |  | MIR148A |
|  |  |  | LCT |
|  |  |  | MIR204 |
|  |  |  | DPP4 |
|  |  |  | DICER1 |
|  |  |  | CACNA1E |
|  |  |  | APOC3 |
|  |  |  | NPPB |
|  |  |  | SKP1 |
|  |  |  | MIR19A |
|  |  |  | PAFAH1B1 |
|  |  |  | APEH |
|  |  |  | H2AC18 |
|  |  |  | FBLIM1 |
|  |  |  | IFNA2 |
|  |  |  | PLTP |
|  |  |  | PLA2G2A |
|  |  |  | FOLH1 |
|  |  |  | FLYWCH1 |
|  |  |  | ANGPTL3 |
|  |  |  | LGALS1 |
|  |  |  | COL15A1 |
|  |  |  | DEFB128 |
|  |  |  | EZH2 |
|  |  |  | SLC25A1 |
|  |  |  | PCMT1 |
|  |  |  | MUC16 |
|  |  |  | SLC25A5 |
|  |  |  | WDR1 |
|  |  |  | FAS |
|  |  |  | PON1 |
|  |  |  | NPB |
|  |  |  | GSTA4 |
|  |  |  | ARNT |
|  |  |  | BDNF |
|  |  |  | UBA7 |
|  |  |  | HBEGF |
|  |  |  | SLC15A4 |
|  |  |  | CDKN1B |
|  |  |  | PRKCI |
|  |  |  | SEMA3A |
|  |  |  | SOD3 |
|  |  |  | FOXC1 |
|  |  |  | CYP21A2 |
|  |  |  | CYSLTR2 |
|  |  |  | ABCA1 |
|  |  |  | MIR223 |
|  |  |  | FGG |
|  |  |  | DDAH1 |
|  |  |  | CSF1 |
|  |  |  | SEMA3E |
|  |  |  | TNFSF10 |
|  |  |  | SPRYD4 |
|  |  |  | LOC110673971 |
|  |  |  | MIR502 |
|  |  |  | CRYAB |
|  |  |  | KRTAP11-1 |
|  |  |  | CKB |
|  |  |  | MUC1 |
|  |  |  | RTN3 |
|  |  |  | SKP2 |
|  |  |  | SLC2A3 |
|  |  |  | MAP3K14 |
|  |  |  | SLC40A1 |
|  |  |  | PTHLH |
|  |  |  | ARID3A |
|  |  |  | FEM1B |
|  |  |  | TNFRSF11B |
|  |  |  | C3AR1 |
|  |  |  | MIR27A |
|  |  |  | MALRD1 |
|  |  |  | CD36 |
|  |  |  | UBLCP1 |
|  |  |  | USP24 |
|  |  |  | MYH9 |
|  |  |  | LBP |
|  |  |  | MIR10A |
|  |  |  | LMF1 |
|  |  |  | C8orf48 |
|  |  |  | MIR215 |
|  |  |  | TNFSF15 |
|  |  |  | DUSP2 |
|  |  |  | NCF2 |
|  |  |  | BDKRB2 |
|  |  |  | IDH2 |
|  |  |  | MIR320A |
|  |  |  | HDGFL1 |
|  |  |  | PAG1 |
|  |  |  | MEF2A |
|  |  |  | TLR6 |
|  |  |  | C1QTNF5 |
|  |  |  | TNNC2 |
|  |  |  | APOL4 |
|  |  |  | HTN3 |
|  |  |  | ADORA2A |
|  |  |  | CDKN2B-AS1 |
|  |  |  | ABO |
|  |  |  | STIM1 |
|  |  |  | KCNH2 |
|  |  |  | TRAF3 |
|  |  |  | CXCR2 |
|  |  |  | CAMK2D |
|  |  |  | F9 |
|  |  |  | LIPE |
|  |  |  | B4GALT1 |
|  |  |  | TMEM161B |
|  |  |  | RPS27A |
|  |  |  | ADCY5 |
|  |  |  | HMCN1 |
|  |  |  | MIR181B1 |
|  |  |  | TNNI3 |
|  |  |  | CD40 |
|  |  |  | LY86 |
|  |  |  | SGCB |
|  |  |  | IGFALS |
|  |  |  | E2F1 |
|  |  |  | PLCB3 |
|  |  |  | ARHGEF2 |
|  |  |  | APOB |
|  |  |  | CASP8 |
|  |  |  | CCM2 |
|  |  |  | CFH |
|  |  |  | PIGR |
|  |  |  | TSC2 |
|  |  |  | CYP11B2 |
|  |  |  | CHDS9 |
|  |  |  | MIR23A |
|  |  |  | ETS2 |
|  |  |  | CS |
|  |  |  | IGFBP7 |
|  |  |  | FCRL3 |
|  |  |  | SIRT4 |
|  |  |  | PRDX1 |
|  |  |  | FGB |
|  |  |  | CDH2 |
|  |  |  | ITGA1 |
|  |  |  | ECE2 |
|  |  |  | FLNC |
|  |  |  | ARF1 |
|  |  |  | ARID5B |
|  |  |  | HAL |
|  |  |  | MYOT |
|  |  |  | SGIP1 |
|  |  |  | SCD5 |
|  |  |  | TMEM248P1 |
|  |  |  | CAVIN1 |
|  |  |  | C4A |
|  |  |  | PPARG |
|  |  |  | AQP4 |
|  |  |  | MERTK |
|  |  |  | DSG2 |
|  |  |  | APLNR |
|  |  |  | HOXC-AS1 |
|  |  |  | ORMDL3 |
|  |  |  | JAG1 |
|  |  |  | TANC1 |
|  |  |  | TBXA2R |
|  |  |  | CHGA |
|  |  |  | ALOX15 |
|  |  |  | ST2 |
|  |  |  | HSPA4 |
|  |  |  | PAPPA |
|  |  |  | DMPK |
|  |  |  | NEAT1 |
|  |  |  | RHOA |
|  |  |  | MIR494 |
|  |  |  | TRH |
|  |  |  | CCL7 |
|  |  |  | CALCA |
|  |  |  | PRKCD |
|  |  |  | VPS41 |
|  |  |  | LPXN |
|  |  |  | DSC2 |
|  |  |  | CHDS1 |
|  |  |  | CXCL11 |
|  |  |  | TMPRSS6 |
|  |  |  | SERPINA1 |
|  |  |  | TNNC1 |
|  |  |  | MIR92A1 |
|  |  |  | PPIG |
|  |  |  | SUN1 |
|  |  |  | LDAH |
|  |  |  | MAPT |
|  |  |  | PRKAA1 |
|  |  |  | FADS2 |
|  |  |  | IL1RL1 |
|  |  |  | SYNE4 |
|  |  |  | HLA-DQB1 |
|  |  |  | MT-CYB |
|  |  |  | GABRG1 |
|  |  |  | ZNF441 |
|  |  |  | AHCY |
|  |  |  | MTTP |
|  |  |  | CXCL8 |
|  |  |  | MAPK9 |
|  |  |  | EGR1 |
|  |  |  | CCL17 |
|  |  |  | THSD1 |
|  |  |  | KHK |
|  |  |  | RIPK2 |
|  |  |  | CHRNB4 |
|  |  |  | HDAC9 |
|  |  |  | GLP1R |
|  |  |  | OGG1 |
|  |  |  | PARP14 |
|  |  |  | DYM |
|  |  |  | RPL15P15 |
|  |  |  | RPL9P21 |
|  |  |  | TUBA1B |
|  |  |  | SELE |
|  |  |  | MSX2 |
|  |  |  | PSMC6 |
|  |  |  | ABCB1 |
|  |  |  | CNR1 |
|  |  |  | PBRM1 |
|  |  |  | FBXO33 |
|  |  |  | CPB2 |
|  |  |  | LINC01535 |
|  |  |  | FGF19 |
|  |  |  | PIM1 |
|  |  |  | CTF1 |
|  |  |  | EGFL7 |
|  |  |  | PIK3R3 |
|  |  |  | STAP1 |
|  |  |  | KLF6 |
|  |  |  | SLC34A1 |
|  |  |  | THBD |
|  |  |  | NR3C1 |
|  |  |  | CYP4V2 |
|  |  |  | FCN2 |
|  |  |  | PIK3C2A |
|  |  |  | CORO1B |
|  |  |  | CHKB |
|  |  |  | SCUBE1 |
|  |  |  | F11 |
|  |  |  | CLEC4C |
|  |  |  | MAP2K6 |
|  |  |  | DCD |
|  |  |  | CD180 |
|  |  |  | EMP1 |
|  |  |  | IL17A |
|  |  |  | ATP2A1 |
|  |  |  | TMPO |
|  |  |  | COG2 |
|  |  |  | PNLIP |
|  |  |  | LEPR |
|  |  |  | MTOR |
|  |  |  | MIR124-1 |
|  |  |  | ABCA8 |
|  |  |  | PDE9A |
|  |  |  | PITPNA |
|  |  |  | NDUFS2 |
|  |  |  | STUB1 |
|  |  |  | GP6 |
|  |  |  | WT1 |
|  |  |  | ILK |
|  |  |  | LEPROT |
|  |  |  | MAPK14 |
|  |  |  | SERPINB2 |
|  |  |  | TXNRD2 |
|  |  |  | AOC3 |
|  |  |  | PITX2 |
|  |  |  | TNFRSF4 |
|  |  |  | MTHFD2 |
|  |  |  | GJA4 |
|  |  |  | MYBPC3 |
|  |  |  | FABP3 |
|  |  |  | TRPV2 |
|  |  |  | SLC27A1 |
|  |  |  | MYPN |
|  |  |  | CCN2 |
|  |  |  | HPR |
|  |  |  | ANXA6 |
|  |  |  | P2RY1 |
|  |  |  | TFPT |
|  |  |  | CSK |
|  |  |  | CTSS |
|  |  |  | MIR448 |
|  |  |  | SMPD1 |
|  |  |  | BHMT2 |
|  |  |  | RPL7 |
|  |  |  | TH |
|  |  |  | FBLN1 |
|  |  |  | GSTO2 |
|  |  |  | PRKAB2 |
|  |  |  | HSPB2 |
|  |  |  | RAF1 |
|  |  |  | MIR19B2 |
|  |  |  | STC1 |
|  |  |  | KLRK1 |
|  |  |  | FURIN |
|  |  |  | ADH7 |
|  |  |  | LTB4R2 |
|  |  |  | UNGP1 |
|  |  |  | ST8SIA1 |
|  |  |  | APOA5 |
|  |  |  | CCR2 |
|  |  |  | EDN3 |
|  |  |  | ANK2 |
|  |  |  | SPON1 |
|  |  |  | PLA2G4A |
|  |  |  | SF3A2 |
|  |  |  | DRD2 |
|  |  |  | VLDLR |
|  |  |  | VEGFC |
|  |  |  | GATAD1 |
|  |  |  | ITGB6 |
|  |  |  | PPP1R3B |
|  |  |  | SDC4 |
|  |  |  | MPO |
|  |  |  | LMOD1 |
|  |  |  | FHL2 |
|  |  |  | PDGFB |
|  |  |  | ELN |
|  |  |  | CALR |
|  |  |  | VIM |
|  |  |  | CADPS |
|  |  |  | ADRA1B |
|  |  |  | LTB |
|  |  |  | NUDT6 |
|  |  |  | TLR8 |
|  |  |  | KCNMB1 |
|  |  |  | DES |
|  |  |  | LGALS3BP |
|  |  |  | ITGAV |
|  |  |  | FGFR2 |
|  |  |  | XRCC3 |
|  |  |  | CSF3 |
|  |  |  | PCNA |
|  |  |  | MMACHC |
|  |  |  | RBM20 |
|  |  |  | B4GALT6 |
|  |  |  | PRKAR1A |
|  |  |  | ATG5 |
|  |  |  | HLA-A |
|  |  |  | SUPT3H |
|  |  |  | ADAMTS4 |
|  |  |  | TNNI2 |
|  |  |  | LDHA |
|  |  |  | EDNRB |
|  |  |  | CD27 |
|  |  |  | FAM167A |
|  |  |  | SCD |
|  |  |  | ARMH3 |
|  |  |  | MIR106B |
|  |  |  | FEM1A |
|  |  |  | HSPA6 |
|  |  |  | CHUK |
|  |  |  | CHAT |
|  |  |  | NR1H2 |
|  |  |  | SEC11B |
|  |  |  | STAT3 |
|  |  |  | CAPG |
|  |  |  | SPHK1 |
|  |  |  | ATN1 |
|  |  |  | MYH10 |
|  |  |  | ARSB |
|  |  |  | IL22 |
|  |  |  | ORAI1 |
|  |  |  | PARPBP |
|  |  |  | ADAM9 |
|  |  |  | TPH1 |
|  |  |  | STXBP5 |
|  |  |  | AMPD1 |
|  |  |  | TBXAS1 |
|  |  |  | MIR103A1 |
|  |  |  | MYH11 |
|  |  |  | IFNGR2 |
|  |  |  | AGK |
|  |  |  | CAP1 |
|  |  |  | ARG1 |
|  |  |  | KIF2C |
|  |  |  | DUSP1 |
|  |  |  | DDAH2 |
|  |  |  | FGF4 |
|  |  |  | NPR3 |
|  |  |  | SLPI |
|  |  |  | DMRTA1 |
|  |  |  | BCAR1 |
|  |  |  | ADAM10 |
|  |  |  | ATXN3 |
|  |  |  | PRPS1 |
|  |  |  | LIPA |
|  |  |  | P2RY2 |
|  |  |  | PLD5 |
|  |  |  | H19 |
|  |  |  | CAMLG |
|  |  |  | TREML4 |
|  |  |  | TGFBR1 |
|  |  |  | P2RY12 |
|  |  |  | NOX4 |
|  |  |  | GRK2 |
|  |  |  | CDC42 |
|  |  |  | ENSG00000269918 |
|  |  |  | VWF |
|  |  |  | VTN |
|  |  |  | TPM1 |
|  |  |  | MIR24-1 |
|  |  |  | CIDEC |
|  |  |  | EBF1 |
|  |  |  | NINJ2 |
|  |  |  | TNKS |
|  |  |  | CYP2E1 |
|  |  |  | FLNB |
|  |  |  | WDFY4 |
|  |  |  | CTH |
|  |  |  | MFSD10 |
|  |  |  | IL15 |
|  |  |  | ATM |
|  |  |  | TIE1 |
|  |  |  | PLEKHA1 |
|  |  |  | TP53COR1 |
|  |  |  | HSPA1A |
|  |  |  | ANGPTL8 |
|  |  |  | KCNK1 |
|  |  |  | C1orf210 |
|  |  |  | LIG4 |
|  |  |  | EREG |
|  |  |  | MIR30C2 |
|  |  |  | BMP4 |
|  |  |  | SFRP4 |
|  |  |  | AGTR1 |
|  |  |  | MCAM |
|  |  |  | IPMK |
|  |  |  | CAMP |
|  |  |  | OBSCN |
|  |  |  | ABCG4 |
|  |  |  | UGT1A4 |
|  |  |  | FCAMR |
|  |  |  | IL1RAP |
|  |  |  | MAT2B |
|  |  |  | SMILR |
|  |  |  | SRGN |
|  |  |  | PRDX5 |
|  |  |  | PPIA |
|  |  |  | CASP7 |
|  |  |  | MED6 |
|  |  |  | MCI2 |
|  |  |  | TRIB1 |
|  |  |  | PRKAG3 |
|  |  |  | TJP1 |
|  |  |  | EDEM2 |
|  |  |  | CD46 |
|  |  |  | PTX3 |
|  |  |  | ICOSLG |
|  |  |  | MIR128-1 |
|  |  |  | UGT1A6 |
|  |  |  | ANG |
|  |  |  | CCK |
|  |  |  | TRIM9 |
|  |  |  | SMAD4 |
|  |  |  | CSMD1 |
|  |  |  | RETNLB |
|  |  |  | CTSD |
|  |  |  | SGMS1 |
|  |  |  | STAT6 |
|  |  |  | JCAD |
|  |  |  | FKBP1B |
|  |  |  | TRERF1 |
|  |  |  | SCARB1 |
|  |  |  | IL3 |
|  |  |  | VAV2 |
|  |  |  | PTCH1 |
|  |  |  | TP53BP1 |
|  |  |  | S100A1 |
|  |  |  | PTPA |
|  |  |  | GREM1 |
|  |  |  | SUN2 |
|  |  |  | CYP4A11 |
|  |  |  | TES |
|  |  |  | PRKAG2 |
|  |  |  | AGXT2 |
|  |  |  | EPRS1 |
|  |  |  | MIR197 |
|  |  |  | ILF3 |
|  |  |  | C1QTNF9 |
|  |  |  | MT1X |
|  |  |  | KEAP1 |
|  |  |  | RFC4 |
|  |  |  | LARP6 |
|  |  |  | SERPINF2 |
|  |  |  | MIAT |
|  |  |  | FBN1 |
|  |  |  | SGCG |
|  |  |  | TYRO3 |
|  |  |  | S100A8 |
|  |  |  | HAVCR1 |
|  |  |  | SLC10A2 |
|  |  |  | KRI1 |
|  |  |  | LEMD3 |
|  |  |  | CHKA |
|  |  |  | CPQ |
|  |  |  | TNFRSF13C |
|  |  |  | AIF1 |
|  |  |  | MAP3K1 |
|  |  |  | PBK |
|  |  |  | SOAT1 |
|  |  |  | AZU1 |
|  |  |  | CTNNA3 |
|  |  |  | MT-ND1 |
|  |  |  | HDLC3 |
|  |  |  | F8 |
|  |  |  | MIR133A1 |
|  |  |  | ACHE |
|  |  |  | NOMO3 |
|  |  |  | TNFRSF12A |
|  |  |  | RPL29P29 |
|  |  |  | EGR3 |
|  |  |  | SERPIND1 |
|  |  |  | IL12A |
|  |  |  | ARNTL |
|  |  |  | BMPER |
|  |  |  | CTCF |
|  |  |  | MTNR1A |
|  |  |  | PPP1R3A |
|  |  |  | MCL1 |
|  |  |  | CCL2 |
|  |  |  | SURF2 |
|  |  |  | RGS9BP |
|  |  |  | MIR125A |
|  |  |  | ACTN2 |
|  |  |  | TXNIP |
|  |  |  | ATG9B |
|  |  |  | SHMT1 |
|  |  |  | ADCY10 |
|  |  |  | CCND3 |
|  |  |  | M6PR |
|  |  |  | ADA |
|  |  |  | SPATA7 |
|  |  |  | DOT1L |
|  |  |  | MAZ |
|  |  |  | IL27 |
|  |  |  | EIF2AK3 |
|  |  |  | PDCD10 |
|  |  |  | KLK15 |
|  |  |  | CAMK2G |
|  |  |  | IL6 |
|  |  |  | TLR3 |
|  |  |  | FGF14 |
|  |  |  | POLB |
|  |  |  | ANXA5 |
|  |  |  | EPHX2 |
|  |  |  | SSNA1 |
|  |  |  | LPAR2 |
|  |  |  | CCL24 |
|  |  |  | PRICKLE2 |
|  |  |  | ANGPT1 |
|  |  |  | RAN |
|  |  |  | CPT2 |
|  |  |  | GPR26 |
|  |  |  | GLRA3 |
|  |  |  | ADPRS |
|  |  |  | POLR2D |
|  |  |  | STAG3 |
|  |  |  | EPHA1 |
|  |  |  | MIR15B |
|  |  |  | EPOR |
|  |  |  | MIR185 |
|  |  |  | HNRNPC |
|  |  |  | LTB4R |
|  |  |  | RPL35AP22 |
|  |  |  | CASP3 |
|  |  |  | HSP90AA1 |
|  |  |  | ANGPT2 |
|  |  |  | CACNA2D3 |
|  |  |  | DUOX1 |
|  |  |  | TSBP1 |
|  |  |  | RNF213 |
|  |  |  | ACSS2 |
|  |  |  | PARP1 |
|  |  |  | PLIN1 |
|  |  |  | SUCLG1 |
|  |  |  | PKN2 |
|  |  |  | ITGA4 |
|  |  |  | VDR |
|  |  |  | ACE |
|  |  |  | CLOCK |
|  |  |  | MLX |
|  |  |  | NPY5R |
|  |  |  | PRKCE |
|  |  |  | ITGAM |
|  |  |  | ROCK1 |
|  |  |  | BLOC1S1 |
|  |  |  | CD34 |
|  |  |  | DSPP |
|  |  |  | BCO1 |
|  |  |  | RGS2 |
|  |  |  | CHDS2 |
|  |  |  | TAGLN |
|  |  |  | TPSAB1 |
|  |  |  | PDPN |
|  |  |  | MIR423 |
|  |  |  | DLL3 |
|  |  |  | PPT2 |
|  |  |  | FASLG |
|  |  |  | RTN4 |
|  |  |  | COL3A1 |
|  |  |  | ENG |
|  |  |  | MIR126 |
|  |  |  | LIPM |
|  |  |  | LIPF |
|  |  |  | RXRG |
|  |  |  | CACNA1D |
|  |  |  | PRKAA2 |
|  |  |  | UHRF1BP1 |
|  |  |  | CD163 |
|  |  |  | MIR125B1 |
|  |  |  | DUSP19 |
|  |  |  | KDR |
|  |  |  | KCNK2 |
|  |  |  | RGS5 |
|  |  |  | SORCS1 |
|  |  |  | TBC1D4 |
|  |  |  | DNM3 |
|  |  |  | RLN2 |
|  |  |  | BBS2 |
|  |  |  | IL1RAPL2 |
|  |  |  | TUBA4A |
|  |  |  | HPD |
|  |  |  | MYLIP |
|  |  |  | SPAG17 |
|  |  |  | HNF1A |
|  |  |  | EPHA3 |
|  |  |  | LMAN2 |
|  |  |  | MFAP4 |
|  |  |  | INSIG2 |
|  |  |  | TYROBP |
|  |  |  | DPRXP7 |
|  |  |  | RARA |
|  |  |  | B3GALT4 |
|  |  |  | OSCAR |
|  |  |  | PECAM1 |
|  |  |  | SFRP5 |
|  |  |  | MIR205 |
|  |  |  | MYD88 |
|  |  |  | TNC |
|  |  |  | PTPN1 |
|  |  |  | DLG2 |
|  |  |  | PML |
|  |  |  | BRAP |
|  |  |  | CCR8 |
|  |  |  | MYH14 |
|  |  |  | PPP1R12B |
|  |  |  | DAB2 |
|  |  |  | TBX4 |
|  |  |  | MIR636 |
|  |  |  | MARCKS |
|  |  |  | LRP6 |
|  |  |  | MIR145 |
|  |  |  | SERPINH1 |
|  |  |  | PPARA |
|  |  |  | CNR2 |
|  |  |  | KRIT1 |
|  |  |  | MIR30B |
|  |  |  | MGLL |
|  |  |  | RORA |
|  |  |  | PTPRD |
|  |  |  | ALOX15B |
|  |  |  | CDK9 |
|  |  |  | IL2RA |
|  |  |  | CD59 |
|  |  |  | PLCG2 |
|  |  |  | FAP |
|  |  |  | KCNJ1 |
|  |  |  | LRRTM4 |
|  |  |  | TTR |
|  |  |  | CCR1 |
|  |  |  | CLDN5 |
|  |  |  | ITGA2B |
|  |  |  | MAOB |
|  |  |  | PTPRN2 |
|  |  |  | PTGDS |
|  |  |  | PSAP |
|  |  |  | UGT1A9 |
|  |  |  | CRYAA |
|  |  |  | ATP1A2 |
|  |  |  | COPS5 |
|  |  |  | TNFSF4 |
|  |  |  | TPM4 |
|  |  |  | BCL2L11 |
|  |  |  | HERPUD1 |
|  |  |  | DUSP6 |
|  |  |  | TGFB3 |
|  |  |  | ELK1 |
|  |  |  | HGD |
|  |  |  | INHBA |
|  |  |  | BMPR2 |
|  |  |  | RBP4 |
|  |  |  | LOC100506178 |
|  |  |  | NME2 |
|  |  |  | ADAM33 |
|  |  |  | ZNF491 |
|  |  |  | S100A4 |
|  |  |  | BTC |
|  |  |  | MSH5 |
|  |  |  | LGMN |
|  |  |  | TCN1 |
|  |  |  | LEPQTL1 |
|  |  |  | CPEB2 |
|  |  |  | IGF2 |
|  |  |  | LILRB1 |
|  |  |  | CTSG |
|  |  |  | ADM2 |
|  |  |  | LINC02577 |
|  |  |  | CD80 |
|  |  |  | NUCB2 |
|  |  |  | RARRES2 |
|  |  |  | FOXO4 |
|  |  |  | ADPRH |
|  |  |  | DNMT1 |
|  |  |  | NOD1 |
|  |  |  | JMJD6 |
|  |  |  | KIF6 |
|  |  |  | ADAMTS1 |
|  |  |  | MIR486-1 |
|  |  |  | PRKG1 |
|  |  |  | CFI |
|  |  |  | SBF2 |
|  |  |  | CDH9 |
|  |  |  | ADRA2B |
|  |  |  | SUV39H1 |
|  |  |  | PLA2G5 |
|  |  |  | AAMP |
|  |  |  | CYP20A1 |
|  |  |  | RNASE3 |
|  |  |  | PIK3CG |
|  |  |  | ACKR1 |
|  |  |  | LPA |
|  |  |  | COL4A4 |
|  |  |  | FGF7 |
|  |  |  | IL37 |
|  |  |  | NPC1L1 |
|  |  |  | UTS2 |
|  |  |  | TAT |
|  |  |  | RREB1 |
|  |  |  | MB |
|  |  |  | C12orf43 |
|  |  |  | MIR92A2 |
|  |  |  | LIFR |
|  |  |  | NQO1 |
|  |  |  | CBL |
|  |  |  | SP1 |
|  |  |  | APOBEC1 |
|  |  |  | CACNG8 |
|  |  |  | SURF1 |
|  |  |  | MIR23B |
|  |  |  | CD79A |
|  |  |  | S1PR1 |
|  |  |  | IL1R2 |
|  |  |  | PIK3CA |
|  |  |  | SGCA |
|  |  |  | MAGI2 |
|  |  |  | LARGE1 |
|  |  |  | IRGM |
|  |  |  | CKLF |
|  |  |  | TSPO |
|  |  |  | SRIP1 |
|  |  |  | ADORA1 |
|  |  |  | UCP1 |
|  |  |  | DAB2IP |
|  |  |  | FGF9 |
|  |  |  | IL2 |
|  |  |  | FADD |
|  |  |  | ZNF844 |
|  |  |  | SDC1 |
|  |  |  | SYK |
|  |  |  | FGA |
|  |  |  | SERPINB1 |
|  |  |  | IGFBP1 |
|  |  |  | SLC1A1 |
|  |  |  | SLC1A4 |
|  |  |  | DDIT3 |
|  |  |  | HOMER1 |
|  |  |  | PLA2G15 |
|  |  |  | CDH1 |
|  |  |  | PTGR1 |
|  |  |  | CALU |
|  |  |  | PNPLA5 |
|  |  |  | TBC1D1 |
|  |  |  | PLAG1 |
|  |  |  | FOS |
|  |  |  | IFNG |
|  |  |  | AGPAT2 |
|  |  |  | CREG1 |
|  |  |  | EMD |
|  |  |  | HSPA5 |
|  |  |  | MIR616 |
|  |  |  | FNDC5 |
|  |  |  | RETN |
|  |  |  | CCL8 |
|  |  |  | CYP27A1 |
|  |  |  | PAFAH2 |
|  |  |  | ALDH8A1 |
|  |  |  | IGES |
|  |  |  | CCDC80 |
|  |  |  | SSH1 |
|  |  |  | MIR135B |
|  |  |  | RHOB |
|  |  |  | H6PD |
|  |  |  | GNAQ |
|  |  |  | CAVIN4 |
|  |  |  | XPA |
|  |  |  | MIR142 |
|  |  |  | FADS3 |
|  |  |  | PEX3 |
|  |  |  | FGF2 |
|  |  |  | CLU |
|  |  |  | AMH |
|  |  |  | PRODH |
|  |  |  | IRF5 |
|  |  |  | ACAT2 |
|  |  |  | CXCL12 |
|  |  |  | TRPM1 |
|  |  |  | NTN1 |
|  |  |  | IL36A |
|  |  |  | TET2 |
|  |  |  | COL2A1 |
|  |  |  | IDI1 |
|  |  |  | LHFPL1 |
|  |  |  | RPA1 |
|  |  |  | EEF1E1 |
|  |  |  | FCGR2C |
|  |  |  | CA10 |
|  |  |  | RRM2 |
|  |  |  | HK2 |
|  |  |  | MDK |
|  |  |  | MRE11 |
|  |  |  | AKAP9 |
|  |  |  | METRNL |
|  |  |  | EPHX1 |
|  |  |  | CD63 |
|  |  |  | SLC20A1 |
|  |  |  | CX3CL1 |
|  |  |  | AIMP1 |
|  |  |  | LDB2 |
|  |  |  | FEN1 |
|  |  |  | IGF1R |
|  |  |  | PPIF |
|  |  |  | SPTB |
|  |  |  | MIR124-3 |
|  |  |  | EFEMP2 |
|  |  |  | ICAM1 |
|  |  |  | CXCL9 |
|  |  |  | RECK |
|  |  |  | CAV1 |
|  |  |  | GNPDA2 |
|  |  |  | H3-2 |
|  |  |  | DGAT2 |
|  |  |  | BCL2L1 |
|  |  |  | DNAH9 |
|  |  |  | RAPGEF3 |
|  |  |  | HELLS |
|  |  |  | TNFRSF1B |
|  |  |  | LGALS9 |
|  |  |  | MIR495 |
|  |  |  | MAPK3 |
|  |  |  | SLC20A2 |
|  |  |  | AEBP1 |
|  |  |  | PAQR5 |
|  |  |  | SLC16A3 |
|  |  |  | ANKRD2 |
|  |  |  | HMGCL |
|  |  |  | SLC9A1 |
|  |  |  | SLC12A5-AS1 |
|  |  |  | TNNI3K |
|  |  |  | CACNA1S |
|  |  |  | MSI2 |
|  |  |  | CORIN |
|  |  |  | PRR7 |
|  |  |  | MRTFA |
|  |  |  | SPARCL1 |
|  |  |  | NEXN |
|  |  |  | CTSL |
|  |  |  | THBS2 |
|  |  |  | PLEC |
|  |  |  | CA2 |
|  |  |  | IL6ST |
|  |  |  | LTF |
|  |  |  | TPO |
|  |  |  | ANK1 |
|  |  |  | ETS1 |
|  |  |  | MLST8 |
|  |  |  | AGRP |
|  |  |  | GATA5 |
|  |  |  | MIR181A1 |
|  |  |  | HSP90B1 |
|  |  |  | APCS |
|  |  |  | CXCL1 |
|  |  |  | MIR92B |
|  |  |  | ABCB4 |
|  |  |  | MIR100 |
|  |  |  | SGMS2 |
|  |  |  | MXD3 |
|  |  |  | FAH |
|  |  |  | SFTPB |
|  |  |  | VAMP3 |
|  |  |  | RCE1 |
|  |  |  | HNF4A |
|  |  |  | CNTNAP2 |
|  |  |  | MYH7 |
|  |  |  | TNFRSF10B |
|  |  |  | lnc-PIK3CG-6 |
|  |  |  | SH2B3 |
|  |  |  | TCIRG1 |
|  |  |  | LOC110973015 |
|  |  |  | MIR503 |
|  |  |  | RELA |
|  |  |  | HIF1A |
|  |  |  | TRAF1 |
|  |  |  | RYR2 |
|  |  |  | IRF1-AS1 |
|  |  |  | GRN |
|  |  |  | DBN1 |
|  |  |  | MIR133B |
|  |  |  | SLC2A4 |
|  |  |  | AP3D1 |
|  |  |  | PTS |
|  |  |  | KCNK13 |
|  |  |  | MAP2 |
|  |  |  | ADCY6 |
|  |  |  | TNFRSF11A |
|  |  |  | NOS3 |
|  |  |  | TGFA |
|  |  |  | NPR2 |
|  |  |  | MMP17 |
|  |  |  | ANPEP |
|  |  |  | LOC100507053 |
|  |  |  | PROM1 |
|  |  |  | MIR10B |
|  |  |  | PAOD1 |
|  |  |  | CFTR |
|  |  |  | TUSC1 |
|  |  |  | GSTM3 |
|  |  |  | KLF14 |
|  |  |  | PTPRA |
|  |  |  | TBX20 |
|  |  |  | KCNA5 |
|  |  |  | FLRT2 |
|  |  |  | HSF1 |
|  |  |  | WRN |
|  |  |  | NFATC2 |
|  |  |  | NOMO2 |
|  |  |  | NPPC |
|  |  |  | CYLD |
|  |  |  | ZEB2 |
|  |  |  | TBCAP1 |
|  |  |  | RHBDF2 |
|  |  |  | ABCG5 |
|  |  |  | MIR16-1 |
|  |  |  | USF1 |
|  |  |  | JMJD1C |
|  |  |  | NFKBIB |
|  |  |  | CHRM2 |
|  |  |  | DKK1 |
|  |  |  | CAPN5 |
|  |  |  | CYCSP14 |
|  |  |  | KCNH5 |
|  |  |  | NR1H4 |
|  |  |  | RRM1 |
|  |  |  | ATP6V1G2-DDX39B |
|  |  |  | OLFML2B |
|  |  |  | ERCC6 |
|  |  |  | NOS1AP |
|  |  |  | PRMT3 |
|  |  |  | NOTCH4 |
|  |  |  | INPP5D |
|  |  |  | CLEC12A |
|  |  |  | CDKN1C |
|  |  |  | ABCA4 |
|  |  |  | SIRT3 |
|  |  |  | AQP1 |
|  |  |  | OPRM1 |
|  |  |  | GFAP |
|  |  |  | DPP8 |
|  |  |  | CYP26B1 |
|  |  |  | ACTB |
|  |  |  | IL7R |
|  |  |  | MSRA |
|  |  |  | IFI35 |
|  |  |  | TLR4 |
|  |  |  | RGS9 |
|  |  |  | LECT2 |
|  |  |  | SRSF2 |
|  |  |  | BSCL2 |
|  |  |  | PCDH9 |
|  |  |  | CCL21 |
|  |  |  | ADAMTS13 |
|  |  |  | IL16 |
|  |  |  | MSTN |
|  |  |  | VCL |
|  |  |  | UBA52P6 |
|  |  |  | RPL31P26 |
|  |  |  | SERPINE1 |
|  |  |  | HFE |
|  |  |  | POU2F3 |
|  |  |  | CYP2C8 |
|  |  |  | BACH1 |
|  |  |  | MYO7A |
|  |  |  | MIR17 |
|  |  |  | VCAM1 |
|  |  |  | MIR19B1 |
|  |  |  | PTAFR |
|  |  |  | ACTG2 |
|  |  |  | ROBO2 |
|  |  |  | NOX1 |
|  |  |  | IFNGR1 |
|  |  |  | CSN1S1 |
|  |  |  | SRFBP1 |
|  |  |  | TSPAN6 |
|  |  |  | PRELID1 |
|  |  |  | RSAD2 |
|  |  |  | INSR |
|  |  |  | TGFBR2 |
|  |  |  | CDH23 |
|  |  |  | TLR9 |
|  |  |  | TTN |
|  |  |  | IL33 |
|  |  |  | RGS6 |
|  |  |  | ITGA11 |
|  |  |  | ZNF700 |
|  |  |  | WWTR1 |
|  |  |  | IFNA1 |
|  |  |  | HHIP |
|  |  |  | HLA-DRB1 |
|  |  |  | MEG3 |
|  |  |  | FGF3 |
|  |  |  | RAD51 |
|  |  |  | CLEC16A |
|  |  |  | ADH1B |
|  |  |  | DYRK1A |
|  |  |  | MIR146B |
|  |  |  | PEMT |
|  |  |  | CDK5 |
|  |  |  | SARDH |
|  |  |  | NFKBIE |
|  |  |  | ANGPTL2 |
|  |  |  | NIT2 |
|  |  |  | NOG |
|  |  |  | HBS1L |
|  |  |  | HLA-B |
|  |  |  | ATP5F1B |
|  |  |  | TCF7L2 |
|  |  |  | PKM |
|  |  |  | HSPA1B |
|  |  |  | HEY2 |
|  |  |  | GCG |
|  |  |  | piR-43107-137 |
|  |  |  | GPD2 |
|  |  |  | CXCL5 |
|  |  |  | SPRR3 |
|  |  |  | GSTO1 |
|  |  |  | DAOA |
|  |  |  | ENPP1 |
|  |  |  | SCNN1A |
|  |  |  | FABP4 |
|  |  |  | UNC13C |
|  |  |  | KCNE2 |
|  |  |  | RHOBTB3 |
|  |  |  | P2RX4 |
|  |  |  | CCND2 |
|  |  |  | CFB |
|  |  |  | PDZK1 |
|  |  |  | DDX58 |
|  |  |  | ADIPOR2 |
|  |  |  | AKT1 |
|  |  |  | UBIAD1 |
|  |  |  | FCN3 |
|  |  |  | PTPN3 |
|  |  |  | ABCA7 |
|  |  |  | TMEM258 |
|  |  |  | RPS27P1 |
|  |  |  | CES5A |
|  |  |  | ACTA2-AS1 |
|  |  |  | MIR362 |
|  |  |  | CAPN10 |
|  |  |  | CCR5 |
|  |  |  | MIR130B |
|  |  |  | SLC5A1 |
|  |  |  | FABP12 |
|  |  |  | PDLIM3 |
|  |  |  | ZNHIT3 |
|  |  |  | VEGFD |
|  |  |  | ZFHX3 |
|  |  |  | PCSK5 |
|  |  |  | GIMAP5 |
|  |  |  | NDUFA9 |
|  |  |  | CYP2B6 |
|  |  |  | FDXR |
|  |  |  | NES |
|  |  |  | PITX3 |
|  |  |  | PNLIPRP1 |
|  |  |  | EYA4 |
|  |  |  | PRKAB1 |
|  |  |  | PON2 |
|  |  |  | GPX3 |
|  |  |  | EDN2 |
|  |  |  | YAP1 |
|  |  |  | HPX |
|  |  |  | MALAT1 |
|  |  |  | KLK1 |
|  |  |  | L3MBTL3 |
|  |  |  | CLEC4A |
|  |  |  | MIF |
|  |  |  | MLXIPL |
|  |  |  | PIP5KL1 |
|  |  |  | C4BPB |
|  |  |  | MAP2K1 |
|  |  |  | GH1 |
|  |  |  | PFN1 |
|  |  |  | AIM2 |
|  |  |  | ARL15 |
|  |  |  | HPS5 |
|  |  |  | ANK3 |
|  |  |  | BCL3 |
|  |  |  | TIMP2 |
|  |  |  | CCN3 |
|  |  |  | ITGB3 |
|  |  |  | PKD2L1 |
|  |  |  | F2RL3 |
|  |  |  | BMPR1B |
|  |  |  | CLIP1 |
|  |  |  | CCL15 |
|  |  |  | GP5 |
|  |  |  | AAT2 |
|  |  |  | LTC4S |
|  |  |  | SEMA5A |
|  |  |  | P4HB |
|  |  |  | MIR217 |
|  |  |  | STC2 |
|  |  |  | TNXA |
|  |  |  | TLL1 |
|  |  |  | TMEM132B |
|  |  |  | COL6A1 |
|  |  |  | ZNF788P |
|  |  |  | OAZ1 |
|  |  |  | IL11 |
|  |  |  | NUMA1 |
|  |  |  | F12 |
|  |  |  | TCF7 |
|  |  |  | F11R |
|  |  |  | DTWD1 |
|  |  |  | HSD11B1 |
|  |  |  | KITLG |
|  |  |  | FOXD4 |
|  |  |  | MMRN1 |
|  |  |  | GSK3A |
|  |  |  | LCN2 |
|  |  |  | PI16 |
|  |  |  | SUMO4 |
|  |  |  | TXN |
|  |  |  | SPARC |
|  |  |  | ZNF326 |
|  |  |  | CTSH |
|  |  |  | COL5A1 |
|  |  |  | ACTA2 |
|  |  |  | BECN1 |
|  |  |  | TRPC4AP |
|  |  |  | ANKRD1 |
|  |  |  | IL20RB |
|  |  |  | SAA2 |
|  |  |  | ZNF592 |
|  |  |  | IFN1@ |
|  |  |  | LAMA4 |
|  |  |  | PTGS1 |
|  |  |  | RNF157 |
|  |  |  | SERPINC1 |
|  |  |  | NPS |
|  |  |  | CFP |
|  |  |  | F3 |
|  |  |  | MIR133A2 |
|  |  |  | PLEKHM2 |
|  |  |  | NR1I2 |
|  |  |  | CES4A |
|  |  |  | IGFBP4 |
|  |  |  | KCNJ5 |
|  |  |  | LMNA |
|  |  |  | NDUFA10 |
|  |  |  | CASQ2 |
|  |  |  | EIF3FP3 |
|  |  |  | APOA1 |
|  |  |  | PRICKLE1 |
|  |  |  | CCDC92 |
|  |  |  | KLF10 |
|  |  |  | FOXO3 |
|  |  |  | CD69 |
|  |  |  | LTA |
|  |  |  | TTPA |
|  |  |  | MYOZ1 |
|  |  |  | IFNB1 |
|  |  |  | PSEN1 |
|  |  |  | HULC |
|  |  |  | MIR499A |
|  |  |  | CYP46A1 |
|  |  |  | RAET1E |
|  |  |  | SLC2A9 |
|  |  |  | CDKN2A |
|  |  |  | PHOSPHO1 |
|  |  |  | LPAR1 |
|  |  |  | ETV6 |
|  |  |  | PMM2 |
|  |  |  | SCN2B |
|  |  |  | CEBPB |
|  |  |  | HAS1 |
|  |  |  | FMNL2 |
|  |  |  | TRPS1 |
|  |  |  | HMGA1 |
|  |  |  | RPS26P57 |
|  |  |  | CCL3 |
|  |  |  | lnc-SPC24-1 |
|  |  |  | CA1 |
|  |  |  | TFPI2 |
|  |  |  | ATHS |
|  |  |  | STAG3L4 |
|  |  |  | ATG16L1 |
|  |  |  | ERN1 |
|  |  |  | CSF1R |
|  |  |  | JUN |
|  |  |  | TRA |
|  |  |  | IRAK4 |
|  |  |  | MYL2 |
|  |  |  | STK38 |
|  |  |  | ADAMTS5 |
|  |  |  | NOTCH3 |
|  |  |  | ATP2A2 |
|  |  |  | MYOM3 |
|  |  |  | ATIC |
|  |  |  | CXCL16 |
|  |  |  | NF2 |
|  |  |  | NRG1 |
|  |  |  | TREX1 |
|  |  |  | TNFSF14 |
|  |  |  | FBXO32 |
|  |  |  | TGFBI |
|  |  |  | AR |
|  |  |  | MYH7B |
|  |  |  | KCNN4 |
|  |  |  | LEXM |
|  |  |  | GATM |
|  |  |  | MHRT |
|  |  |  | CDKN3 |
|  |  |  | CDH4 |
|  |  |  | ARHGEF10 |
|  |  |  | JPH3 |
|  |  |  | CYP2J2 |
|  |  |  | MIR30C1 |
|  |  |  | HDC |
|  |  |  | PXK |
|  |  |  | HSPG2 |
|  |  |  | FLNA |
|  |  |  | HEYL |
|  |  |  | IL2RB |
|  |  |  | ADCY9 |
|  |  |  | NOTCH1 |
|  |  |  | BAX |
|  |  |  | CD274 |
|  |  |  | LRPAP1 |
|  |  |  | CP |
|  |  |  | IGHE |
|  |  |  | TM4SF5 |
|  |  |  | PDYN |
|  |  |  | CDKAL1 |
|  |  |  | DAPK1 |
|  |  |  | GORASP1 |
|  |  |  | CDH5 |
|  |  |  | FKBP1A |
|  |  |  | PINX1 |
|  |  |  | CYP24A1 |
|  |  |  | SCML4 |
|  |  |  | EIF3G |
|  |  |  | ANO6 |
|  |  |  | SMARCAL1 |
|  |  |  | FOSL1 |
|  |  |  | GSTT1 |
|  |  |  | NPC1 |
|  |  |  | PRKCA |
|  |  |  | UGT1A3 |
|  |  |  | TRIB2 |
|  |  |  | PRMT7 |
|  |  |  | ALDH9A1 |
|  |  |  | CD40LG |
|  |  |  | AHSP |
|  |  |  | CES1 |
|  |  |  | UGT1A5 |
|  |  |  | DNAH5 |
|  |  |  | SRF |
|  |  |  | GHSR |
|  |  |  | MAT2A |
|  |  |  | TUBB4A |
|  |  |  | TRPC3 |
|  |  |  | TMEM106B |
|  |  |  | KLRD1 |
|  |  |  | FMN2 |
|  |  |  | NPTXR |
|  |  |  | IL7 |
|  |  |  | SEMA3C |
|  |  |  | SCN9A |
|  |  |  | SCN5A |
|  |  |  | UBE2Q2P1 |
|  |  |  | GPC1 |
|  |  |  | ZNF383 |
|  |  |  | HOTTIP |
|  |  |  | PDGFC |
|  |  |  | PEPD |
|  |  |  | GLS2 |
|  |  |  | APP |
|  |  |  | GATA4 |
|  |  |  | ATP2B1 |
|  |  |  | VHL |
|  |  |  | PRKAG1 |
|  |  |  | SMAD5 |
|  |  |  | IGF2BP1 |
|  |  |  | MIR212 |
|  |  |  | ANKRD23 |
|  |  |  | FGF23 |
|  |  |  | PRSS1 |
|  |  |  | PNPLA2 |
|  |  |  | HLX |
|  |  |  | PTGIS |
|  |  |  | PAH |
|  |  |  | CEACAM3 |
|  |  |  | MIR216A |
|  |  |  | SNX17 |
|  |  |  | HDAC6 |
|  |  |  | MIR33B |
|  |  |  | CYP2R1 |
|  |  |  | FBLN5 |
|  |  |  | MIR490 |
|  |  |  | TNNT1 |
|  |  |  | S100A9 |
|  |  |  | CSF2 |
|  |  |  | ITPR3 |
|  |  |  | BIRC3 |
|  |  |  | RPS6KB1 |
|  |  |  | GPLD1 |
|  |  |  | MMP9 |
|  |  |  | DNAJC19 |
|  |  |  | RNLS |
|  |  |  | SRY |
|  |  |  | HBA1 |
|  |  |  | DCAF8 |
|  |  |  | AQP7 |
|  |  |  | ADIPOQ |
|  |  |  | RXRB |
|  | Pravastatin | 7 | SLCO2B1 |
|  |  |  | SLC22A6 |
|  |  |  | SLC22A7 |
|  |  |  | SLC16A1 |
|  |  |  | SLCO1B3 |
|  |  |  | SLC22A8 |
|  |  |  | SLCO1A2 |
|  | Propranolol | 3 | SLC22A2 |
|  |  |  | HTR1B |
|  |  |  | CYP3A7 |
| coronary artery atherosclerosis vs herbal strategies vs familiar remedies | coronary artery atherosclerosis/familiar remedies/herbal strategies | 3 | MAOA |
|  |  |  | HTR1A |
|  |  |  | ABCG2 |
|  | coronary artery atherosclerosis/herbal strategies | 75 | MMP2 |
|  |  |  | XDH |
|  |  |  | CCNB1 |
|  |  |  | MET |
|  |  |  | TEK |
|  |  |  | CD38 |
|  |  |  | CDK1 |
|  |  |  | PTK2 |
|  |  |  | SHBG |
|  |  |  | ESRRA |
|  |  |  | PTGES |
|  |  |  | AHR |
|  |  |  | CYP19A1 |
|  |  |  | PDGFRB |
|  |  |  | AKR1C4 |
|  |  |  | F2 |
|  |  |  | CCND1 |
|  |  |  | CCNA2 |
|  |  |  | GSK3B |
|  |  |  | MMP12 |
|  |  |  | GLO1 |
|  |  |  | ALOX12 |
|  |  |  | MMP3 |
|  |  |  | AXL |
|  |  |  | CXCR1 |
|  |  |  | EGFR |
|  |  |  | CCNB2 |
|  |  |  | ERBB2 |
|  |  |  | GPR35 |
|  |  |  | AKR1B1 |
|  |  |  | TERT |
|  |  |  | PTPN6 |
|  |  |  | SRC |
|  |  |  | CDK2 |
|  |  |  | CA3 |
|  |  |  | TYR |
|  |  |  | PIK3R1 |
|  |  |  | ABCC1 |
|  |  |  | AKR1A1 |
|  |  |  | FLT4 |
|  |  |  | CSNK2A1 |
|  |  |  | CYP1B1 |
|  |  |  | MMP13 |
|  |  |  | ALOX5 |
|  |  |  | GRK6 |
|  |  |  | PTPN11 |
|  |  |  | EZH2 |
|  |  |  | ADORA2A |
|  |  |  | ALOX15 |
|  |  |  | ABCB1 |
|  |  |  | PIM1 |
|  |  |  | DRD2 |
|  |  |  | MPO |
|  |  |  | STAT3 |
|  |  |  | ARG1 |
|  |  |  | NOX4 |
|  |  |  | TNKS |
|  |  |  | HSPA1A |
|  |  |  | ACHE |
|  |  |  | POLB |
|  |  |  | PARP1 |
|  |  |  | KDR |
|  |  |  | TTR |
|  |  |  | ADORA1 |
|  |  |  | SYK |
|  |  |  | IGF1R |
|  |  |  | CA2 |
|  |  |  | INSR |
|  |  |  | CDK5 |
|  |  |  | AKT1 |
|  |  |  | CA1 |
|  |  |  | DAPK1 |
|  |  |  | CES1 |
|  |  |  | APP |
|  |  |  | MMP9 |
|  | coronary artery atherosclerosis/familiar remedies | 15 | CYP2C19 |
|  |  |  | ORM1 |
|  |  |  | CYP1A1 |
|  |  |  | CYP3A5 |
|  |  |  | ADRB1 |
|  |  |  | ABCC2 |
|  |  |  | SLCO1B1 |
|  |  |  | HMGCR |
|  |  |  | ADRB3 |
|  |  |  | ABCB11 |
|  |  |  | SLC22A11 |
|  |  |  | ADRB2 |
|  |  |  | CYP1A2 |
|  |  |  | HDAC2 |
|  |  |  | CYP3A4 |
|  | coronary artery atherosclerosis | 2903 | MSRB1 |
|  |  |  | FSTL1 |
|  |  |  | SLC18A1 |
|  |  |  | RIT2 |
|  |  |  | CD44 |
|  |  |  | ABCA3 |
|  |  |  | KCNMA1 |
|  |  |  | KCNJ2 |
|  |  |  | VPS33A |
|  |  |  | PINK1 |
|  |  |  | RALYL |
|  |  |  | ITGA5 |
|  |  |  | NDUFS6 |
|  |  |  | UTP20 |
|  |  |  | MEFV |
|  |  |  | EDN1 |
|  |  |  | TKT |
|  |  |  | HSPB1 |
|  |  |  | ABCC6 |
|  |  |  | CXCR4 |
|  |  |  | DMP1 |
|  |  |  | CUBN |
|  |  |  | RMRP |
|  |  |  | ADAM12 |
|  |  |  | IRAK1 |
|  |  |  | MSBP1 |
|  |  |  | IGF1 |
|  |  |  | TMSB10 |
|  |  |  | G3BP1 |
|  |  |  | MIR20A |
|  |  |  | CXCL3 |
|  |  |  | LMAN1 |
|  |  |  | BCAR4 |
|  |  |  | GAS5 |
|  |  |  | MIRLET7G |
|  |  |  | NCF1C |
|  |  |  | GALNT3 |
|  |  |  | LDB3 |
|  |  |  | PAK1 |
|  |  |  | SUMO1 |
|  |  |  | MIR6886 |
|  |  |  | GP1BA |
|  |  |  | ANGPTL5 |
|  |  |  | MRPL10 |
|  |  |  | GDF15 |
|  |  |  | CYCS |
|  |  |  | EP300 |
|  |  |  | TLN1 |
|  |  |  | MMP7 |
|  |  |  | NKX2-5 |
|  |  |  | PRKCG |
|  |  |  | NOS2 |
|  |  |  | TRIB3 |
|  |  |  | CACNA2D1 |
|  |  |  | HRG |
|  |  |  | PON3 |
|  |  |  | CDK14 |
|  |  |  | ERG |
|  |  |  | FAT4 |
|  |  |  | SOD2 |
|  |  |  | MIR15A |
|  |  |  | MSH3 |
|  |  |  | AQP9 |
|  |  |  | PDE1C |
|  |  |  | XK |
|  |  |  | SEMA4D |
|  |  |  | BAK1 |
|  |  |  | FGFR1 |
|  |  |  | AGPAT1 |
|  |  |  | MTNR1B |
|  |  |  | HLA-DRB5 |
|  |  |  | PCSK1 |
|  |  |  | OSBPL8 |
|  |  |  | SERPINA12 |
|  |  |  | NR4A2 |
|  |  |  | PPCS |
|  |  |  | UGT1A1 |
|  |  |  | CXCL13 |
|  |  |  | SORL1 |
|  |  |  | MNS1 |
|  |  |  | NAT10 |
|  |  |  | FADS1 |
|  |  |  | LEP |
|  |  |  | CXADR |
|  |  |  | SERPINF1 |
|  |  |  | CACNG5 |
|  |  |  | MAPKAPK2 |
|  |  |  | DIP2A |
|  |  |  | OXT |
|  |  |  | CCL22 |
|  |  |  | SHC1 |
|  |  |  | PTPN22 |
|  |  |  | ITGA3 |
|  |  |  | HSD11B2 |
|  |  |  | ENSG00000280087 |
|  |  |  | BRCA1 |
|  |  |  | GGT2 |
|  |  |  | LINC02618 |
|  |  |  | CRYGC |
|  |  |  | S1PR2 |
|  |  |  | RUNX3 |
|  |  |  | LIMS2 |
|  |  |  | PRKCB |
|  |  |  | C5AR1 |
|  |  |  | SMO |
|  |  |  | A2M |
|  |  |  | MIR132 |
|  |  |  | UTRN |
|  |  |  | CNN1 |
|  |  |  | BAZ1B |
|  |  |  | APOBR |
|  |  |  | LMNB2 |
|  |  |  | ROCK2 |
|  |  |  | PALLD |
|  |  |  | GPC5 |
|  |  |  | MT-ATP6 |
|  |  |  | OSBPL9P2 |
|  |  |  | TLR1 |
|  |  |  | VKORC1 |
|  |  |  | LRGUK |
|  |  |  | ELANE |
|  |  |  | CD160 |
|  |  |  | COL1A1 |
|  |  |  | PDCD5 |
|  |  |  | SLC8A1 |
|  |  |  | GPR37L1 |
|  |  |  | HES1 |
|  |  |  | OGDH |
|  |  |  | MACROD1 |
|  |  |  | GSS |
|  |  |  | C1QTNF3 |
|  |  |  | NECTIN2 |
|  |  |  | CCN4 |
|  |  |  | SSTR2 |
|  |  |  | FOXP3 |
|  |  |  | BIRC5 |
|  |  |  | CHRNA5 |
|  |  |  | UTS2B |
|  |  |  | MIR624 |
|  |  |  | SELL |
|  |  |  | GNAS |
|  |  |  | ANKRD6 |
|  |  |  | LTBP4 |
|  |  |  | PYY |
|  |  |  | GHR |
|  |  |  | CCL19 |
|  |  |  | SIRT6 |
|  |  |  | GPR55 |
|  |  |  | MTRR |
|  |  |  | NCOR2 |
|  |  |  | SERPINB9 |
|  |  |  | TNFRSF9 |
|  |  |  | CCNE1 |
|  |  |  | MS4A2 |
|  |  |  | PODN |
|  |  |  | BHMT |
|  |  |  | NPC2 |
|  |  |  | IL17RC |
|  |  |  | HS3ST1 |
|  |  |  | GUCY1A1 |
|  |  |  | ITGB1BP2 |
|  |  |  | RBPJ |
|  |  |  | GATA6 |
|  |  |  | APOE |
|  |  |  | PF4 |
|  |  |  | DSTN |
|  |  |  | ALDH2 |
|  |  |  | ST8SIA4 |
|  |  |  | DAPK3 |
|  |  |  | C3 |
|  |  |  | MRAP |
|  |  |  | TPM3 |
|  |  |  | HSPD1 |
|  |  |  | UGT1A12P |
|  |  |  | F2RL1 |
|  |  |  | PRDM16 |
|  |  |  | MIR378A |
|  |  |  | APOC4 |
|  |  |  | FABP5 |
|  |  |  | HYOU1 |
|  |  |  | UGT1A10 |
|  |  |  | TUBB |
|  |  |  | MFSD11 |
|  |  |  | SORT1 |
|  |  |  | PLIN2 |
|  |  |  | PPM1K |
|  |  |  | MYH6 |
|  |  |  | RBFOX1 |
|  |  |  | BCHE |
|  |  |  | ENOSF1 |
|  |  |  | SERPING1 |
|  |  |  | TONSL |
|  |  |  | GCLM |
|  |  |  | NFKB2 |
|  |  |  | BCL2 |
|  |  |  | ARL13B |
|  |  |  | CAMTA1 |
|  |  |  | TNFRSF10A |
|  |  |  | KL |
|  |  |  | P4HA3 |
|  |  |  | SAMSN1 |
|  |  |  | ALCAM |
|  |  |  | PLCL2 |
|  |  |  | IL24 |
|  |  |  | TRAM1 |
|  |  |  | BDKRB1 |
|  |  |  | NDUFS4 |
|  |  |  | ID1 |
|  |  |  | PIK3CB |
|  |  |  | COL18A1 |
|  |  |  | ENSG00000243797 |
|  |  |  | TNXB |
|  |  |  | CARD8 |
|  |  |  | BCAM |
|  |  |  | PCK2 |
|  |  |  | FKRP |
|  |  |  | TOX3 |
|  |  |  | TF |
|  |  |  | HLA-DRA |
|  |  |  | GCK |
|  |  |  | THRB |
|  |  |  | RNF39 |
|  |  |  | MYBPC1 |
|  |  |  | ADRA2A |
|  |  |  | NPR1 |
|  |  |  | TERC |
|  |  |  | ABRAXAS2 |
|  |  |  | GSTM4 |
|  |  |  | TMEM59 |
|  |  |  | SEC23IP |
|  |  |  | F7 |
|  |  |  | PDCL2 |
|  |  |  | EDNRA |
|  |  |  | MIR9-1 |
|  |  |  | CCT8 |
|  |  |  | POLG |
|  |  |  | IRS1 |
|  |  |  | GHRL |
|  |  |  | SLC17A3 |
|  |  |  | MFN2 |
|  |  |  | MICU1 |
|  |  |  | LOC111365141 |
|  |  |  | ATR |
|  |  |  | GNA11 |
|  |  |  | FOXB1 |
|  |  |  | EBPL |
|  |  |  | ARSH |
|  |  |  | TACR1 |
|  |  |  | CAPN1 |
|  |  |  | RYR1 |
|  |  |  | LTBP1 |
|  |  |  | MT3 |
|  |  |  | PPBP |
|  |  |  | ZNF687 |
|  |  |  | HTR3A |
|  |  |  | OXTR |
|  |  |  | ACTC1 |
|  |  |  | SDC2 |
|  |  |  | MAPK10 |
|  |  |  | ABI2 |
|  |  |  | CACNB1 |
|  |  |  | QKI |
|  |  |  | ATF1 |
|  |  |  | IL5 |
|  |  |  | FERMT3 |
|  |  |  | AGT |
|  |  |  | LRRC10 |
|  |  |  | MFAP5 |
|  |  |  | FGF1 |
|  |  |  | CHRM1 |
|  |  |  | IL6R |
|  |  |  | CST3 |
|  |  |  | CHI3L1 |
|  |  |  | LEMD2 |
|  |  |  | CLEC3B |
|  |  |  | CIITA |
|  |  |  | NOMO1 |
|  |  |  | CRP |
|  |  |  | GLA |
|  |  |  | NEBL |
|  |  |  | HSPA8 |
|  |  |  | IMMT |
|  |  |  | CEL |
|  |  |  | SELENOP |
|  |  |  | NEU1 |
|  |  |  | TARID |
|  |  |  | AGGF1 |
|  |  |  | PCCB |
|  |  |  | ENHO |
|  |  |  | NPHS1 |
|  |  |  | SPRY2 |
|  |  |  | GSTP1 |
|  |  |  | LTBR |
|  |  |  | CCR3 |
|  |  |  | NBPF3 |
|  |  |  | MIR340 |
|  |  |  | HBG2 |
|  |  |  | CYP7A1 |
|  |  |  | STAT5A |
|  |  |  | AGTRAP |
|  |  |  | SRD5A2 |
|  |  |  | MIR122 |
|  |  |  | ARHGEF38 |
|  |  |  | AP3B1 |
|  |  |  | MIR206 |
|  |  |  | KISS1R |
|  |  |  | NRG4 |
|  |  |  | MT-ND2 |
|  |  |  | IL5RA |
|  |  |  | TIAM1 |
|  |  |  | RGS14 |
|  |  |  | IL18BP |
|  |  |  | SMPD2 |
|  |  |  | MIR182 |
|  |  |  | FHL1 |
|  |  |  | TNFSF12 |
|  |  |  | CSRP3 |
|  |  |  | MTR |
|  |  |  | FN1 |
|  |  |  | LPIN3 |
|  |  |  | DHFR |
|  |  |  | ALPP |
|  |  |  | PPP1R12A |
|  |  |  | VSIR |
|  |  |  | PHACTR1 |
|  |  |  | GPR132 |
|  |  |  | ATP1A1 |
|  |  |  | LOXL1 |
|  |  |  | MIR144 |
|  |  |  | NFE2L2 |
|  |  |  | IL23R |
|  |  |  | NRF1 |
|  |  |  | SORCS2 |
|  |  |  | NR1I3 |
|  |  |  | ACVR2A |
|  |  |  | KLF4 |
|  |  |  | YWHAZ |
|  |  |  | KLKB1 |
|  |  |  | GAA |
|  |  |  | IFI27 |
|  |  |  | HDAC3 |
|  |  |  | SCN1A |
|  |  |  | HP |
|  |  |  | MAGOHB |
|  |  |  | MED18 |
|  |  |  | ECE1 |
|  |  |  | PARG |
|  |  |  | MT-LIPCAR |
|  |  |  | LIPI |
|  |  |  | UIMC1 |
|  |  |  | MIR29B1 |
|  |  |  | BMP6 |
|  |  |  | CLTCL1 |
|  |  |  | TRB |
|  |  |  | ALG6 |
|  |  |  | NPPA |
|  |  |  | TBX21 |
|  |  |  | PPARGC1A |
|  |  |  | TNFAIP6 |
|  |  |  | FMN1 |
|  |  |  | SSRP1 |
|  |  |  | CCL26 |
|  |  |  | BLOC1S5-TXNDC5 |
|  |  |  | SNAI1 |
|  |  |  | BRD4 |
|  |  |  | CD151 |
|  |  |  | MTHFD1 |
|  |  |  | HRH2 |
|  |  |  | PLA2G6 |
|  |  |  | CHDS3 |
|  |  |  | SOX9 |
|  |  |  | FTH1 |
|  |  |  | GC |
|  |  |  | IER3 |
|  |  |  | VSTM4 |
|  |  |  | MIR208B |
|  |  |  | CEBPD |
|  |  |  | G6PC2 |
|  |  |  | OMD |
|  |  |  | WFDC21P |
|  |  |  | CSRP1 |
|  |  |  | CTSB |
|  |  |  | lnc-APOC1-1 |
|  |  |  | TMEM170A |
|  |  |  | ADCY3 |
|  |  |  | SVEP1 |
|  |  |  | FETUB |
|  |  |  | PXN |
|  |  |  | TNF |
|  |  |  | NTM |
|  |  |  | SIRT2 |
|  |  |  | C1QBP |
|  |  |  | NDUFB8 |
|  |  |  | GDF2 |
|  |  |  | NGFR |
|  |  |  | IKBKG |
|  |  |  | IL23A |
|  |  |  | TCF21 |
|  |  |  | NSD1 |
|  |  |  | JPH2 |
|  |  |  | C5 |
|  |  |  | SLC2A10 |
|  |  |  | EGF |
|  |  |  | TCP1 |
|  |  |  | GNRH1 |
|  |  |  | IL1A |
|  |  |  | CMA1 |
|  |  |  | DSCR10 |
|  |  |  | CACNA1H |
|  |  |  | PDGFD |
|  |  |  | FST |
|  |  |  | ERCC1 |
|  |  |  | CAV3 |
|  |  |  | KCND3 |
|  |  |  | CREBBP |
|  |  |  | LDLR |
|  |  |  | ITGB1 |
|  |  |  | PARK7 |
|  |  |  | RPN1 |
|  |  |  | PMVK |
|  |  |  | KLF2 |
|  |  |  | BRINP3 |
|  |  |  | COQ10A |
|  |  |  | SGK1 |
|  |  |  | COL5A2 |
|  |  |  | RPL21P42 |
|  |  |  | SELP |
|  |  |  | PGM1 |
|  |  |  | MYOZ2 |
|  |  |  | DLGAP1 |
|  |  |  | PGLYRP1 |
|  |  |  | TANK |
|  |  |  | RCAN1 |
|  |  |  | AOC1 |
|  |  |  | MAP1LC3B |
|  |  |  | NAB2 |
|  |  |  | UGT1A8 |
|  |  |  | THY1 |
|  |  |  | KIT |
|  |  |  | KRT8 |
|  |  |  | SHH |
|  |  |  | THPO |
|  |  |  | RPL29P27 |
|  |  |  | MIR210 |
|  |  |  | EIF2S1 |
|  |  |  | SCN4B |
|  |  |  | PPOX |
|  |  |  | HAND2 |
|  |  |  | JAK2 |
|  |  |  | ANTXR1 |
|  |  |  | CREB3 |
|  |  |  | RAB5A |
|  |  |  | TERF2 |
|  |  |  | TNFRSF1A |
|  |  |  | HERC6 |
|  |  |  | RPL7AP57 |
|  |  |  | CASP1 |
|  |  |  | ATG7 |
|  |  |  | ACADVL |
|  |  |  | LRP1B |
|  |  |  | ZBTB8OS |
|  |  |  | SPP1 |
|  |  |  | HBP1 |
|  |  |  | TNNT2 |
|  |  |  | ABCG8 |
|  |  |  | SFTPD |
|  |  |  | EPS15 |
|  |  |  | HCAR2 |
|  |  |  | XIAP |
|  |  |  | ZNF627 |
|  |  |  | EIF2B2 |
|  |  |  | WNT5A |
|  |  |  | APOA2 |
|  |  |  | TRPA1 |
|  |  |  | KALRN |
|  |  |  | SUCLA2P2 |
|  |  |  | ATP6V0A2 |
|  |  |  | FDFT1 |
|  |  |  | ENO2 |
|  |  |  | PTGS2 |
|  |  |  | BMP7 |
|  |  |  | OSM |
|  |  |  | B2M |
|  |  |  | MIRLET7E |
|  |  |  | OPN3 |
|  |  |  | LRP5 |
|  |  |  | OLR1 |
|  |  |  | DAGLB |
|  |  |  | NRAP |
|  |  |  | TRIM63 |
|  |  |  | NCF1 |
|  |  |  | CD2 |
|  |  |  | SPG7 |
|  |  |  | AGER |
|  |  |  | GALNT2 |
|  |  |  | CTNNB1 |
|  |  |  | CBS |
|  |  |  | MIR221 |
|  |  |  | MICA |
|  |  |  | ADH1C |
|  |  |  | SNTA1 |
|  |  |  | MIR141 |
|  |  |  | TLR5 |
|  |  |  | HAS2 |
|  |  |  | FCGR3A |
|  |  |  | CYP2C9 |
|  |  |  | DNMT3A |
|  |  |  | NT5E |
|  |  |  | UTS2R |
|  |  |  | IL18 |
|  |  |  | NPY |
|  |  |  | ITGA6 |
|  |  |  | RPL23P4 |
|  |  |  | BAG3 |
|  |  |  | OXA1L |
|  |  |  | CTSK |
|  |  |  | IGFBP2 |
|  |  |  | NOTCH2 |
|  |  |  | SIGLEC1 |
|  |  |  | PRKDC |
|  |  |  | CD93 |
|  |  |  | LOC100506472 |
|  |  |  | CCL4 |
|  |  |  | MSR1 |
|  |  |  | PDCD6IP |
|  |  |  | ADD1 |
|  |  |  | TOR2A |
|  |  |  | KDM6B |
|  |  |  | ATXN2 |
|  |  |  | SLC17A4 |
|  |  |  | RRAS |
|  |  |  | S100B |
|  |  |  | BMP2 |
|  |  |  | IPO5 |
|  |  |  | NPY1R |
|  |  |  | MGP |
|  |  |  | PCDH15 |
|  |  |  | GYPA |
|  |  |  | TRPC1 |
|  |  |  | SOST |
|  |  |  | ESR1 |
|  |  |  | SURF4 |
|  |  |  | KCNN3 |
|  |  |  | SST |
|  |  |  | ERCC8 |
|  |  |  | PRF1 |
|  |  |  | MAPK7 |
|  |  |  | RRAGC |
|  |  |  | GPX4 |
|  |  |  | ACE2 |
|  |  |  | FBXO3 |
|  |  |  | GCH1 |
|  |  |  | CMKLR1 |
|  |  |  | TYMS |
|  |  |  | DGKQ |
|  |  |  | SCNN1B |
|  |  |  | FMOD |
|  |  |  | IRS2 |
|  |  |  | SIK2 |
|  |  |  | SRRT |
|  |  |  | PDE4D |
|  |  |  | EPHB3 |
|  |  |  | TRPC4 |
|  |  |  | AAT1 |
|  |  |  | RAMP3 |
|  |  |  | CNTN5 |
|  |  |  | ESRRB |
|  |  |  | LRIG1 |
|  |  |  | HRAS |
|  |  |  | NPNT |
|  |  |  | HCG27 |
|  |  |  | TNFRSF10C |
|  |  |  | PDE3A |
|  |  |  | TRHDE |
|  |  |  | ITGB2 |
|  |  |  | SELENOS |
|  |  |  | XYLT2 |
|  |  |  | EPB41L3 |
|  |  |  | MIR361 |
|  |  |  | BCAR3 |
|  |  |  | CDKN2B |
|  |  |  | DHCR7 |
|  |  |  | PROS1 |
|  |  |  | PSMB8 |
|  |  |  | FGFR4 |
|  |  |  | CRAT |
|  |  |  | POR |
|  |  |  | SLC19A1 |
|  |  |  | LIMS1 |
|  |  |  | XRCC1 |
|  |  |  | SREBF2 |
|  |  |  | VEGFA |
|  |  |  | LAMA1 |
|  |  |  | APOL1 |
|  |  |  | ENPP2 |
|  |  |  | FHL5 |
|  |  |  | ACAN |
|  |  |  | TGFB1 |
|  |  |  | ITGA2 |
|  |  |  | GPR182 |
|  |  |  | MME |
|  |  |  | APEX1 |
|  |  |  | ACKR2 |
|  |  |  | MAST4 |
|  |  |  | LPAL2 |
|  |  |  | GAST |
|  |  |  | PSEN2 |
|  |  |  | UBE2L3 |
|  |  |  | MYC |
|  |  |  | ADRA1D |
|  |  |  | NR4A3 |
|  |  |  | MCTP2 |
|  |  |  | ARSA |
|  |  |  | LCMT2 |
|  |  |  | MBL1P |
|  |  |  | PTGIR |
|  |  |  | ENSG00000247287 |
|  |  |  | FOXE3 |
|  |  |  | SOCS3 |
|  |  |  | AGBL1 |
|  |  |  | RNPC3 |
|  |  |  | DAG1 |
|  |  |  | WWOX |
|  |  |  | SLC9A3R2 |
|  |  |  | TIMP1 |
|  |  |  | GAPDHP28 |
|  |  |  | C19orf38 |
|  |  |  | ABCA12 |
|  |  |  | CHIT1 |
|  |  |  | PCSK2 |
|  |  |  | PI3 |
|  |  |  | MRC1 |
|  |  |  | MT-ND6 |
|  |  |  | VCP |
|  |  |  | PCSK9 |
|  |  |  | LAMA3 |
|  |  |  | BGN |
|  |  |  | GAL |
|  |  |  | COCH |
|  |  |  | RAVER1 |
|  |  |  | IL13 |
|  |  |  | CD86 |
|  |  |  | SRSF3 |
|  |  |  | SLC11A1 |
|  |  |  | ICAM2 |
|  |  |  | MIR21 |
|  |  |  | INTS12 |
|  |  |  | CTSF |
|  |  |  | PDXP |
|  |  |  | NFIA-AS1 |
|  |  |  | GPD1L |
|  |  |  | TNFAIP3 |
|  |  |  | PSRC1 |
|  |  |  | PLA2G10 |
|  |  |  | FOXO1 |
|  |  |  | GPT |
|  |  |  | BLK |
|  |  |  | PPP1R17 |
|  |  |  | APOM |
|  |  |  | IBSP |
|  |  |  | PKP2 |
|  |  |  | GLTPD2 |
|  |  |  | MMP1 |
|  |  |  | STAT1 |
|  |  |  | HLA-DQA1 |
|  |  |  | GCLC |
|  |  |  | IGFBP5 |
|  |  |  | COL8A1 |
|  |  |  | SERPINA4 |
|  |  |  | MROH5 |
|  |  |  | FKTN |
|  |  |  | NME4 |
|  |  |  | IL18RAP |
|  |  |  | CARD16 |
|  |  |  | BLVRB |
|  |  |  | TNFSF13 |
|  |  |  | HABP2 |
|  |  |  | VENTXP2 |
|  |  |  | DLL4 |
|  |  |  | MYOCD |
|  |  |  | FBXO15 |
|  |  |  | SLC2A1 |
|  |  |  | LSAMP |
|  |  |  | ATP2A3 |
|  |  |  | MKKS |
|  |  |  | PTGER4 |
|  |  |  | CD68 |
|  |  |  | CCR7 |
|  |  |  | SMARCA1 |
|  |  |  | KCNQ1 |
|  |  |  | RECQL5 |
|  |  |  | APOF |
|  |  |  | ADCY1 |
|  |  |  | NR2F2 |
|  |  |  | BCL2A1 |
|  |  |  | ANGPTL1 |
|  |  |  | RHO |
|  |  |  | GRID1 |
|  |  |  | FHOD3 |
|  |  |  | BANF1 |
|  |  |  | CR1 |
|  |  |  | PRDM1 |
|  |  |  | FAM13A |
|  |  |  | TOR1A |
|  |  |  | BSG |
|  |  |  | P2RY4 |
|  |  |  | TRPV1 |
|  |  |  | UCP2 |
|  |  |  | EFEMP1 |
|  |  |  | ZFYVE9 |
|  |  |  | MIR28 |
|  |  |  | TRIM24 |
|  |  |  | MIRLET7A1 |
|  |  |  | MOCS1 |
|  |  |  | PROC |
|  |  |  | PLXNA4 |
|  |  |  | SLC22A5 |
|  |  |  | GRIN3A |
|  |  |  | DOCK6 |
|  |  |  | FPR1 |
|  |  |  | SYP |
|  |  |  | GNLY |
|  |  |  | FAM223A |
|  |  |  | CCR4 |
|  |  |  | PTPRC |
|  |  |  | CCT7 |
|  |  |  | MYO1E |
|  |  |  | EEF1A2 |
|  |  |  | MIPEP |
|  |  |  | CD14 |
|  |  |  | ABCC9 |
|  |  |  | NDE1 |
|  |  |  | PPARD |
|  |  |  | ANXA2 |
|  |  |  | MCF2L |
|  |  |  | SPEG |
|  |  |  | ARHGAP20 |
|  |  |  | RPL35AP15 |
|  |  |  | LGALS2 |
|  |  |  | NR3C2 |
|  |  |  | MIR134 |
|  |  |  | C4B |
|  |  |  | GSR |
|  |  |  | INPPL1 |
|  |  |  | CACNA1C |
|  |  |  | IL19 |
|  |  |  | HDLCQ2 |
|  |  |  | GGT1 |
|  |  |  | EIF2AK2 |
|  |  |  | WNT1 |
|  |  |  | CCL11 |
|  |  |  | LMO7 |
|  |  |  | PSMD9 |
|  |  |  | RHOD |
|  |  |  | IDO1 |
|  |  |  | COL4A2 |
|  |  |  | KDM5B |
|  |  |  | CCL23 |
|  |  |  | LUM |
|  |  |  | OBSL1 |
|  |  |  | C1S |
|  |  |  | HMOX1 |
|  |  |  | SLC27A6 |
|  |  |  | FCGR2A |
|  |  |  | MC4R |
|  |  |  | GPIHBP1 |
|  |  |  | MIR195 |
|  |  |  | FAM126A |
|  |  |  | FOXP2 |
|  |  |  | TRPM7 |
|  |  |  | INS |
|  |  |  | FLT1 |
|  |  |  | PDIA2 |
|  |  |  | RPLP0 |
|  |  |  | EIF4E |
|  |  |  | HOTAIR |
|  |  |  | EBI3 |
|  |  |  | SMAD2 |
|  |  |  | EFNA5 |
|  |  |  | RPL5P19 |
|  |  |  | ADGRE5 |
|  |  |  | MSBP2 |
|  |  |  | F2RL2 |
|  |  |  | IGF2BP2 |
|  |  |  | BGLAP |
|  |  |  | HTR4 |
|  |  |  | PRKCZ |
|  |  |  | EIF2B4 |
|  |  |  | IL20 |
|  |  |  | MIR590 |
|  |  |  | TRAF6 |
|  |  |  | CCL18 |
|  |  |  | LMNB1 |
|  |  |  | PNPLA3 |
|  |  |  | CNTLN |
|  |  |  | CD276 |
|  |  |  | PLD2 |
|  |  |  | F13A1 |
|  |  |  | SLC10A1 |
|  |  |  | GATA3 |
|  |  |  | LDLR-AS1-001 |
|  |  |  | TFAM |
|  |  |  | SYNE3 |
|  |  |  | OGN |
|  |  |  | FMR1 |
|  |  |  | LDLRAP1 |
|  |  |  | ANGPTL4 |
|  |  |  | HHEX |
|  |  |  | SLC12A3 |
|  |  |  | C9orf72 |
|  |  |  | PLCH1 |
|  |  |  | ZPR1 |
|  |  |  | NAMPT |
|  |  |  | PTGER2 |
|  |  |  | MMP8 |
|  |  |  | ESAM |
|  |  |  | MIR34A |
|  |  |  | FOXC2 |
|  |  |  | NGEF |
|  |  |  | HDAC4 |
|  |  |  | GDNF |
|  |  |  | CTNNA1 |
|  |  |  | ZNF717 |
|  |  |  | MIR497 |
|  |  |  | MIR222 |
|  |  |  | TRPC5 |
|  |  |  | SCGB1A1 |
|  |  |  | NTRK2 |
|  |  |  | CCDC3 |
|  |  |  | ADCYAP1 |
|  |  |  | MFGE8 |
|  |  |  | GSTM1 |
|  |  |  | CRH |
|  |  |  | IL10 |
|  |  |  | PRSS2 |
|  |  |  | CES3 |
|  |  |  | WDR33 |
|  |  |  | ACP5 |
|  |  |  | lnc-KDM5D-4 |
|  |  |  | AMFR |
|  |  |  | LIAS |
|  |  |  | MAPK1 |
|  |  |  | GOLGB1 |
|  |  |  | PGF |
|  |  |  | F11-AS1 |
|  |  |  | CORT |
|  |  |  | TLR10 |
|  |  |  | CHD7 |
|  |  |  | HNRNPA2B1 |
|  |  |  | SLC30A8 |
|  |  |  | PYGB |
|  |  |  | GADD45A |
|  |  |  | TSC1 |
|  |  |  | MIR214 |
|  |  |  | OARD1 |
|  |  |  | IAPP |
|  |  |  | ALOX5AP |
|  |  |  | LRP1 |
|  |  |  | POSTN |
|  |  |  | GP1BB |
|  |  |  | ANKH |
|  |  |  | RIPK3 |
|  |  |  | POM121L3P |
|  |  |  | FTO |
|  |  |  | CYP27B1 |
|  |  |  | SAMHD1 |
|  |  |  | PPP3R1 |
|  |  |  | LDLR-AS1 |
|  |  |  | PAX6 |
|  |  |  | SCG2 |
|  |  |  | ZC3HC1 |
|  |  |  | IGF2-AS |
|  |  |  | MMP14 |
|  |  |  | PLAT |
|  |  |  | SF3A1 |
|  |  |  | MDM2 |
|  |  |  | TAP2 |
|  |  |  | NETO1 |
|  |  |  | TNFSF11 |
|  |  |  | THSD7A |
|  |  |  | PTPRF |
|  |  |  | BLVRA |
|  |  |  | ICMT |
|  |  |  | GNAI1 |
|  |  |  | AKT2 |
|  |  |  | PPFIA2 |
|  |  |  | IKBKB |
|  |  |  | SLC25A4 |
|  |  |  | MYL3 |
|  |  |  | THBS4 |
|  |  |  | TNPO1 |
|  |  |  | ENOX1 |
|  |  |  | MYLK |
|  |  |  | CYP4F2 |
|  |  |  | SOD1 |
|  |  |  | B4GALNT3 |
|  |  |  | PRKD2 |
|  |  |  | MIR130A |
|  |  |  | SIRT5 |
|  |  |  | CXCR3 |
|  |  |  | CYSLTR1 |
|  |  |  | MIRLET7B |
|  |  |  | CTLA4 |
|  |  |  | NR4A1 |
|  |  |  | EMC10 |
|  |  |  | PLA2G2D |
|  |  |  | WNT2 |
|  |  |  | GLMN |
|  |  |  | SIRPA |
|  |  |  | LYVE1 |
|  |  |  | CSMD2 |
|  |  |  | APOH |
|  |  |  | CREB1 |
|  |  |  | PDX1 |
|  |  |  | ADAMTS8 |
|  |  |  | SMAD1 |
|  |  |  | TLR7 |
|  |  |  | STAB1 |
|  |  |  | MXRA7 |
|  |  |  | SRD5A1 |
|  |  |  | LGALS3 |
|  |  |  | UGT1A7 |
|  |  |  | CFL2 |
|  |  |  | STEAP1B |
|  |  |  | FUT3 |
|  |  |  | CLEC7A |
|  |  |  | DHX38 |
|  |  |  | HSPA14 |
|  |  |  | KCNA3 |
|  |  |  | DCBLD1 |
|  |  |  | PDGFA |
|  |  |  | IL17F |
|  |  |  | ZHX2 |
|  |  |  | CFL1 |
|  |  |  | IL4 |
|  |  |  | CD28 |
|  |  |  | SELPLG |
|  |  |  | DIABLO |
|  |  |  | SMARCA4 |
|  |  |  | BAZ2B |
|  |  |  | NAT2 |
|  |  |  | LRP8 |
|  |  |  | CNTN1 |
|  |  |  | MASP2 |
|  |  |  | AGL |
|  |  |  | GBP1 |
|  |  |  | MIR30D |
|  |  |  | NISCH |
|  |  |  | DEFA1 |
|  |  |  | LOC106728418 |
|  |  |  | F13B |
|  |  |  | TMEM43 |
|  |  |  | AKAP12 |
|  |  |  | CLPTM1L |
|  |  |  | MEOX2 |
|  |  |  | HLA-DPB1 |
|  |  |  | STAT5B |
|  |  |  | PRPF8 |
|  |  |  | ZNF213-AS1 |
|  |  |  | MIR93 |
|  |  |  | CEP85L |
|  |  |  | MIR224 |
|  |  |  | CD74 |
|  |  |  | HSPA12B |
|  |  |  | LIPG |
|  |  |  | G6PD |
|  |  |  | KLF5 |
|  |  |  | APOD |
|  |  |  | TRPV4 |
|  |  |  | NEK8 |
|  |  |  | PDE5A |
|  |  |  | SCARA3 |
|  |  |  | NOX5 |
|  |  |  | ENSG00000267052 |
|  |  |  | SULT1A3 |
|  |  |  | MAPK8 |
|  |  |  | CPA3 |
|  |  |  | KCNJ12 |
|  |  |  | ELOVL2 |
|  |  |  | C5AR2 |
|  |  |  | NRG3 |
|  |  |  | F10 |
|  |  |  | GSC2 |
|  |  |  | ACVR1 |
|  |  |  | COMP |
|  |  |  | HLA-DQA2 |
|  |  |  | STAR |
|  |  |  | F2R |
|  |  |  | THBS1 |
|  |  |  | MTAP |
|  |  |  | RFC1 |
|  |  |  | ACSL1 |
|  |  |  | H2AX |
|  |  |  | TCAP |
|  |  |  | ABL1 |
|  |  |  | NCOR1 |
|  |  |  | MIR143 |
|  |  |  | MOCOS |
|  |  |  | TXNRD1 |
|  |  |  | GZMB |
|  |  |  | FCGR3B |
|  |  |  | BMP1 |
|  |  |  | VIP |
|  |  |  | SP2 |
|  |  |  | CAPS2 |
|  |  |  | TAZ |
|  |  |  | CWC22 |
|  |  |  | MTFP1 |
|  |  |  | MMP26 |
|  |  |  | HLA-G |
|  |  |  | DIO2 |
|  |  |  | LRP2 |
|  |  |  | GPER1 |
|  |  |  | CYBB |
|  |  |  | MYDGF |
|  |  |  | HSP90AB1 |
|  |  |  | SEMA6D |
|  |  |  | CABIN1 |
|  |  |  | HSPE1 |
|  |  |  | MAPK13 |
|  |  |  | MYCN |
|  |  |  | SYNE1 |
|  |  |  | MTHFD1L |
|  |  |  | MVK |
|  |  |  | C2 |
|  |  |  | GCKR |
|  |  |  | APLN |
|  |  |  | DPP9 |
|  |  |  | NCOA1 |
|  |  |  | GUSB |
|  |  |  | HRH1 |
|  |  |  | DNM2 |
|  |  |  | PLA2G7 |
|  |  |  | CCL1 |
|  |  |  | CD247 |
|  |  |  | NEURL1 |
|  |  |  | KRT18 |
|  |  |  | CEP19 |
|  |  |  | SOCS1 |
|  |  |  | MIR155 |
|  |  |  | CDK5RAP3 |
|  |  |  | GGCX |
|  |  |  | HTRA1 |
|  |  |  | IFT88 |
|  |  |  | RNF111 |
|  |  |  | PTH |
|  |  |  | TNFRSF14 |
|  |  |  | TARDBP |
|  |  |  | PJA1 |
|  |  |  | RB1 |
|  |  |  | CETP |
|  |  |  | APOC2 |
|  |  |  | HADHB |
|  |  |  | ADAR |
|  |  |  | ARMS2 |
|  |  |  | PSMD4 |
|  |  |  | DNAH10 |
|  |  |  | P2RX1 |
|  |  |  | CACNB2 |
|  |  |  | NUMB |
|  |  |  | MIR25 |
|  |  |  | CD70 |
|  |  |  | SLC29A1 |
|  |  |  | GLRX |
|  |  |  | SAA1 |
|  |  |  | PF4V1 |
|  |  |  | ST3GAL4 |
|  |  |  | REG1A |
|  |  |  | HBB |
|  |  |  | HEY1 |
|  |  |  | KNG1 |
|  |  |  | ITGAX |
|  |  |  | CASR |
|  |  |  | MIR30E |
|  |  |  | GPAM |
|  |  |  | CANT1 |
|  |  |  | LIN9 |
|  |  |  | MTPN |
|  |  |  | TFAP2A |
|  |  |  | NFS1 |
|  |  |  | ADTRP |
|  |  |  | CDC37 |
|  |  |  | NUP107 |
|  |  |  | CHDS8 |
|  |  |  | PDE4B |
|  |  |  | PGR |
|  |  |  | RUNX2 |
|  |  |  | MT-CO1 |
|  |  |  | SPATA6L |
|  |  |  | GRK4 |
|  |  |  | LRP12 |
|  |  |  | SEC14L2 |
|  |  |  | FGF10 |
|  |  |  | IL1RN |
|  |  |  | ODC1 |
|  |  |  | MIR296 |
|  |  |  | MIR9-2 |
|  |  |  | ADORA2B |
|  |  |  | MAFB |
|  |  |  | PHEX |
|  |  |  | SOX6 |
|  |  |  | ADIPOR1 |
|  |  |  | TFPI |
|  |  |  | SNF8 |
|  |  |  | SGCD |
|  |  |  | SMTN |
|  |  |  | CYP17A1 |
|  |  |  | TWIST2 |
|  |  |  | MIR22 |
|  |  |  | NAB1 |
|  |  |  | CD209 |
|  |  |  | TNNI1 |
|  |  |  | ENO1 |
|  |  |  | CXCL10 |
|  |  |  | TRPM3 |
|  |  |  | P4HA1 |
|  |  |  | GOSR2 |
|  |  |  | RPL7AP58 |
|  |  |  | BFSP1 |
|  |  |  | CHDS4 |
|  |  |  | PLG |
|  |  |  | LOC102724465 |
|  |  |  | ATP6AP2 |
|  |  |  | COL4A1 |
|  |  |  | SEZ6L |
|  |  |  | VCAN |
|  |  |  | ADAMTS3 |
|  |  |  | RPL21P108 |
|  |  |  | LEKR1 |
|  |  |  | MKI67 |
|  |  |  | TSLP |
|  |  |  | DNAH8 |
|  |  |  | STS |
|  |  |  | IL4R |
|  |  |  | BTNL2 |
|  |  |  | CCL5 |
|  |  |  | ADAM8 |
|  |  |  | TGIF1 |
|  |  |  | PEX5 |
|  |  |  | TP53 |
|  |  |  | MAP1LC3A |
|  |  |  | piR-56133-114 |
|  |  |  | CDH7 |
|  |  |  | LINC-ROR |
|  |  |  | HNRNPA1P10 |
|  |  |  | ENTPD1 |
|  |  |  | CCDC157 |
|  |  |  | MIR140 |
|  |  |  | JUP |
|  |  |  | STK11 |
|  |  |  | MX1 |
|  |  |  | SAA4 |
|  |  |  | PLPP3 |
|  |  |  | LRRC18 |
|  |  |  | EXOSC4 |
|  |  |  | VASP |
|  |  |  | LOX |
|  |  |  | RELB |
|  |  |  | NLRP1 |
|  |  |  | BUD13 |
|  |  |  | HNF1B |
|  |  |  | ACP1 |
|  |  |  | TGFBR3 |
|  |  |  | CCN1 |
|  |  |  | UMOD |
|  |  |  | CASP9 |
|  |  |  | MAP3K7 |
|  |  |  | PDCD1 |
|  |  |  | NBL1 |
|  |  |  | SRA1 |
|  |  |  | PRMT5P1 |
|  |  |  | DYNC2LI1 |
|  |  |  | CDKN1A |
|  |  |  | IL32 |
|  |  |  | LPL |
|  |  |  | LAMP2 |
|  |  |  | CAT |
|  |  |  | LOC102723692 |
|  |  |  | HCN4 |
|  |  |  | MARS1 |
|  |  |  | ATXN1 |
|  |  |  | LIPC |
|  |  |  | CPOX |
|  |  |  | GSTZ1 |
|  |  |  | CD84 |
|  |  |  | APH1B |
|  |  |  | RASA1 |
|  |  |  | ADAM17 |
|  |  |  | LYZ |
|  |  |  | F5 |
|  |  |  | CLEC5A |
|  |  |  | TPM2 |
|  |  |  | ELAVL1 |
|  |  |  | CPS1 |
|  |  |  | LIPJ |
|  |  |  | PIAS4 |
|  |  |  | NCOA2 |
|  |  |  | GATA2 |
|  |  |  | ABCA2 |
|  |  |  | SLC2A2 |
|  |  |  | ATP10D |
|  |  |  | CCDC71L |
|  |  |  | HRC |
|  |  |  | CELSR2 |
|  |  |  | LRG1 |
|  |  |  | CFLAR |
|  |  |  | PRKCH |
|  |  |  | GRP |
|  |  |  | CACNG1 |
|  |  |  | PDGFRA |
|  |  |  | CSTB |
|  |  |  | DMD |
|  |  |  | ADM |
|  |  |  | ANKRD30A |
|  |  |  | MIR150 |
|  |  |  | GTF2E2 |
|  |  |  | FCGR1A |
|  |  |  | FPR2 |
|  |  |  | DLC1 |
|  |  |  | CD47 |
|  |  |  | ACP6 |
|  |  |  | MIR199A1 |
|  |  |  | PPP1R1A |
|  |  |  | KCNQ3 |
|  |  |  | SLC22A12 |
|  |  |  | SPRY4 |
|  |  |  | MXD1 |
|  |  |  | IFIH1 |
|  |  |  | LIPK |
|  |  |  | HADHA |
|  |  |  | CHURC1 |
|  |  |  | XRCC5 |
|  |  |  | CFDP1 |
|  |  |  | AHSG |
|  |  |  | POU5F1 |
|  |  |  | HDAC1 |
|  |  |  | USO1 |
|  |  |  | MASP1 |
|  |  |  | SHOX |
|  |  |  | RPL32P12 |
|  |  |  | FBXO38 |
|  |  |  | ICOS |
|  |  |  | NFKB1 |
|  |  |  | SIRT1 |
|  |  |  | TNFRSF6B |
|  |  |  | CAV2 |
|  |  |  | MIA3 |
|  |  |  | SEMA3F |
|  |  |  | MIR27B |
|  |  |  | UGT1A |
|  |  |  | IL1B |
|  |  |  | MIR505 |
|  |  |  | REN |
|  |  |  | CHRM3 |
|  |  |  | G6PC |
|  |  |  | NEK9 |
|  |  |  | ACTA1 |
|  |  |  | TOMM40 |
|  |  |  | CPE |
|  |  |  | CXCR6 |
|  |  |  | HPSE |
|  |  |  | IGF2R |
|  |  |  | DMWD |
|  |  |  | GSTM5 |
|  |  |  | FGF21 |
|  |  |  | LCAT |
|  |  |  | MIR196A2 |
|  |  |  | IL12B |
|  |  |  | ST6GAL1 |
|  |  |  | APOA4 |
|  |  |  | POMC |
|  |  |  | IL9 |
|  |  |  | ZMPSTE24 |
|  |  |  | APEX2 |
|  |  |  | SLC24A3 |
|  |  |  | RPL21P41 |
|  |  |  | TXNL4B |
|  |  |  | NLRP12 |
|  |  |  | piR-55948-110 |
|  |  |  | DNM1L |
|  |  |  | NPY4R |
|  |  |  | SH3GL2 |
|  |  |  | EGID-106632268 |
|  |  |  | MIR381 |
|  |  |  | ITGA7 |
|  |  |  | NFKBIA |
|  |  |  | CCDC159 |
|  |  |  | KCNIP2 |
|  |  |  | IGFBP3 |
|  |  |  | MPRIP |
|  |  |  | PSMA6 |
|  |  |  | SORBS3 |
|  |  |  | C4BPA |
|  |  |  | ALB |
|  |  |  | CPT1A |
|  |  |  | TERF1 |
|  |  |  | ALMS1 |
|  |  |  | HAMP |
|  |  |  | SREBF1 |
|  |  |  | DCN |
|  |  |  | RPL31P23 |
|  |  |  | NOD2 |
|  |  |  | IL21 |
|  |  |  | HTR2A |
|  |  |  | UCP3 |
|  |  |  | EPAS1 |
|  |  |  | SCNN1G |
|  |  |  | S100A12 |
|  |  |  | TREM1 |
|  |  |  | LOC106560211 |
|  |  |  | AMPD2 |
|  |  |  | ACTN1 |
|  |  |  | IFNAR2 |
|  |  |  | HLA-C |
|  |  |  | LINC00907 |
|  |  |  | TIMP4 |
|  |  |  | HSDL2 |
|  |  |  | LPIN1 |
|  |  |  | CX3CR1 |
|  |  |  | HACD4 |
|  |  |  | POU2F1 |
|  |  |  | HSPB7 |
|  |  |  | GAPDH |
|  |  |  | KISS1 |
|  |  |  | APC |
|  |  |  | TLR2 |
|  |  |  | GPX1 |
|  |  |  | CRLF2 |
|  |  |  | P2RX7 |
|  |  |  | DOCK7 |
|  |  |  | GNA12 |
|  |  |  | LIMK1 |
|  |  |  | KCNJ11 |
|  |  |  | RPL36AP23 |
|  |  |  | ABCC8 |
|  |  |  | MACROD2 |
|  |  |  | IL18R1 |
|  |  |  | BPI |
|  |  |  | GLB1 |
|  |  |  | BNC2 |
|  |  |  | SERPINA3 |
|  |  |  | GAS6 |
|  |  |  | RPL28P3 |
|  |  |  | TIMD4 |
|  |  |  | XKR4 |
|  |  |  | GSTM2 |
|  |  |  | STAB2 |
|  |  |  | NAT1 |
|  |  |  | PCOLCE2 |
|  |  |  | HCRT |
|  |  |  | HGFAC |
|  |  |  | PARP9 |
|  |  |  | MCPH1 |
|  |  |  | PEAR1 |
|  |  |  | MT2A |
|  |  |  | MMP10 |
|  |  |  | FABP2 |
|  |  |  | TCN2 |
|  |  |  | ACTG1 |
|  |  |  | CALM1 |
|  |  |  | TAS2R50 |
|  |  |  | HAVCR2 |
|  |  |  | PLAUR |
|  |  |  | MAP2K3 |
|  |  |  | NDP |
|  |  |  | CKM |
|  |  |  | SCAP |
|  |  |  | ESR2 |
|  |  |  | AGTR2 |
|  |  |  | MIR483 |
|  |  |  | LACTB |
|  |  |  | ADAMTSL1 |
|  |  |  | ZBTB46 |
|  |  |  | EPO |
|  |  |  | COL1A2 |
|  |  |  | DTNA |
|  |  |  | SERPINA5 |
|  |  |  | HNRNPA1 |
|  |  |  | LY96 |
|  |  |  | AKT3 |
|  |  |  | KLK3 |
|  |  |  | IL1R1 |
|  |  |  | ATP5IF1 |
|  |  |  | CTTN |
|  |  |  | KCNJ8 |
|  |  |  | PROCR |
|  |  |  | ITIH4 |
|  |  |  | USP8 |
|  |  |  | GJA5 |
|  |  |  | ITGB5 |
|  |  |  | SLC17A5 |
|  |  |  | HGF |
|  |  |  | MRTFB |
|  |  |  | SOX18 |
|  |  |  | TYMP |
|  |  |  | SERPINA10 |
|  |  |  | INSIG1 |
|  |  |  | NR1D1 |
|  |  |  | CFD |
|  |  |  | HIF1A-AS1 |
|  |  |  | MYBPC2 |
|  |  |  | CELA2A |
|  |  |  | APOC1 |
|  |  |  | MIR29A |
|  |  |  | HSPA12A |
|  |  |  | ITGAL |
|  |  |  | PDE4A |
|  |  |  | NHLRC1 |
|  |  |  | CH25H |
|  |  |  | KAT2B |
|  |  |  | SDHA |
|  |  |  | ALPL |
|  |  |  | STEAP2-AS1 |
|  |  |  | COMT |
|  |  |  | SMAD6 |
|  |  |  | PDLIM5 |
|  |  |  | SPC24 |
|  |  |  | MIR26A1 |
|  |  |  | XYLT1 |
|  |  |  | ADAMTS7 |
|  |  |  | ROS1 |
|  |  |  | C1QTNF1 |
|  |  |  | VEGFB |
|  |  |  | AS3MT |
|  |  |  | MT-TL1 |
|  |  |  | MIR208A |
|  |  |  | ACKR3 |
|  |  |  | MIR4675 |
|  |  |  | HYAL1 |
|  |  |  | ZBTB17 |
|  |  |  | LTA4H |
|  |  |  | HDLBP |
|  |  |  | CD1C |
|  |  |  | S1PR3 |
|  |  |  | GNB3 |
|  |  |  | IFIT3 |
|  |  |  | AVP |
|  |  |  | HMGB1 |
|  |  |  | PDE3B |
|  |  |  | HOPX |
|  |  |  | MEF2C |
|  |  |  | RPS3AP9 |
|  |  |  | SOAT2 |
|  |  |  | LBR |
|  |  |  | STEAP1 |
|  |  |  | PGK1 |
|  |  |  | CCL13 |
|  |  |  | MYB |
|  |  |  | BAIAP2L1 |
|  |  |  | ESM1 |
|  |  |  | PFN3 |
|  |  |  | RCN3 |
|  |  |  | RPTOR |
|  |  |  | ERBB4 |
|  |  |  | RYR3 |
|  |  |  | MRPS36P3 |
|  |  |  | E2F8 |
|  |  |  | UGCG |
|  |  |  | DNASE1 |
|  |  |  | PTN |
|  |  |  | MIR1322 |
|  |  |  | LPP |
|  |  |  | IL12RB1 |
|  |  |  | DMRT1 |
|  |  |  | ADRA2C |
|  |  |  | DHCR24 |
|  |  |  | VAMP8 |
|  |  |  | NBN |
|  |  |  | MIR31 |
|  |  |  | HSPB6 |
|  |  |  | COX5A |
|  |  |  | MYL4 |
|  |  |  | EBLN1 |
|  |  |  | CXCL14 |
|  |  |  | AIFM1 |
|  |  |  | GSTA1 |
|  |  |  | SLC17A1 |
|  |  |  | CYBA |
|  |  |  | MIR146A |
|  |  |  | SOS1 |
|  |  |  | TGFB2 |
|  |  |  | PLAU |
|  |  |  | RAC1 |
|  |  |  | LAMA2 |
|  |  |  | PTEN |
|  |  |  | ANXA1 |
|  |  |  | RLBP1 |
|  |  |  | MAATS1 |
|  |  |  | ZNF385D |
|  |  |  | ID3 |
|  |  |  | TICAM1 |
|  |  |  | FCRL6 |
|  |  |  | PROZ |
|  |  |  | C8A |
|  |  |  | LGR6 |
|  |  |  | MTM1 |
|  |  |  | MIR33A |
|  |  |  | RFX5 |
|  |  |  | SLC6A4 |
|  |  |  | CALR3 |
|  |  |  | NAT8 |
|  |  |  | UCN |
|  |  |  | PTRHD1 |
|  |  |  | SMDT1 |
|  |  |  | SYNE2 |
|  |  |  | SLC22A4 |
|  |  |  | NAXE |
|  |  |  | KCNE1 |
|  |  |  | CD4 |
|  |  |  | DSP |
|  |  |  | ITLN1 |
|  |  |  | NOSIP |
|  |  |  | COL20A1 |
|  |  |  | TUG1 |
|  |  |  | IRF1 |
|  |  |  | PTK2B |
|  |  |  | UNC5B |
|  |  |  | SDHB |
|  |  |  | IRF2BP2 |
|  |  |  | MBL2 |
|  |  |  | NGF |
|  |  |  | APOA1-AS |
|  |  |  | ALPK3 |
|  |  |  | NRP1 |
|  |  |  | ACAD8 |
|  |  |  | CDH13 |
|  |  |  | NLRP3 |
|  |  |  | GJA1 |
|  |  |  | PKD1 |
|  |  |  | IL6-AS1 |
|  |  |  | FASN |
|  |  |  | VEPH1 |
|  |  |  | PRDX6 |
|  |  |  | MYOM1 |
|  |  |  | TTF2 |
|  |  |  | AMBP |
|  |  |  | AKAP7 |
|  |  |  | AIDA |
|  |  |  | DOLK |
|  |  |  | ACVRL1 |
|  |  |  | CYCSP42 |
|  |  |  | ASCC3 |
|  |  |  | TGM2 |
|  |  |  | FCGR2B |
|  |  |  | NFATC4 |
|  |  |  | RXRA |
|  |  |  | MAP3K5 |
|  |  |  | GRM8 |
|  |  |  | PDLIM7 |
|  |  |  | SLC23A2 |
|  |  |  | MIR30A |
|  |  |  | NOS1 |
|  |  |  | TBK1 |
|  |  |  | BMPR1A |
|  |  |  | PRL |
|  |  |  | TIMP3 |
|  |  |  | LOC157273 |
|  |  |  | NR1H3 |
|  |  |  | ACACA |
|  |  |  | LYSMD4 |
|  |  |  | ABCG1 |
|  |  |  | MIR98 |
|  |  |  | SQSTM1 |
|  |  |  | MIR149 |
|  |  |  | DSCAML1 |
|  |  |  | NFATC1 |
|  |  |  | RAC2 |
|  |  |  | CD55 |
|  |  |  | PLN |
|  |  |  | MTHFR |
|  |  |  | ACAT1 |
|  |  |  | PRDM9 |
|  |  |  | CSGALNACT2 |
|  |  |  | SMAD3 |
|  |  |  | ATF3 |
|  |  |  | TBX5 |
|  |  |  | TRDN |
|  |  |  | NAPG |
|  |  |  | MRVI1 |
|  |  |  | HMOX2 |
|  |  |  | C1QL3 |
|  |  |  | MPP7 |
|  |  |  | PLCG1 |
|  |  |  | SLC2A13 |
|  |  |  | FBN2 |
|  |  |  | ST3GAL1 |
|  |  |  | NEDD4 |
|  |  |  | RPS27AP2 |
|  |  |  | MIR370 |
|  |  |  | MIR148A |
|  |  |  | LCT |
|  |  |  | MIR204 |
|  |  |  | DPP4 |
|  |  |  | DICER1 |
|  |  |  | CACNA1E |
|  |  |  | APOC3 |
|  |  |  | NPPB |
|  |  |  | SKP1 |
|  |  |  | MIR19A |
|  |  |  | PAFAH1B1 |
|  |  |  | APEH |
|  |  |  | H2AC18 |
|  |  |  | FBLIM1 |
|  |  |  | IFNA2 |
|  |  |  | PLTP |
|  |  |  | PLA2G2A |
|  |  |  | FOLH1 |
|  |  |  | FLYWCH1 |
|  |  |  | ANGPTL3 |
|  |  |  | LGALS1 |
|  |  |  | COL15A1 |
|  |  |  | DEFB128 |
|  |  |  | SLC25A1 |
|  |  |  | PCMT1 |
|  |  |  | MUC16 |
|  |  |  | SLC25A5 |
|  |  |  | WDR1 |
|  |  |  | FAS |
|  |  |  | PON1 |
|  |  |  | NPB |
|  |  |  | GSTA4 |
|  |  |  | ARNT |
|  |  |  | BDNF |
|  |  |  | UBA7 |
|  |  |  | HBEGF |
|  |  |  | SLC15A4 |
|  |  |  | CDKN1B |
|  |  |  | PRKCI |
|  |  |  | SEMA3A |
|  |  |  | SOD3 |
|  |  |  | FOXC1 |
|  |  |  | CYP21A2 |
|  |  |  | CYSLTR2 |
|  |  |  | ABCA1 |
|  |  |  | MIR223 |
|  |  |  | FGG |
|  |  |  | DDAH1 |
|  |  |  | CSF1 |
|  |  |  | SEMA3E |
|  |  |  | TNFSF10 |
|  |  |  | SPRYD4 |
|  |  |  | LOC110673971 |
|  |  |  | MIR502 |
|  |  |  | CRYAB |
|  |  |  | KRTAP11-1 |
|  |  |  | CKB |
|  |  |  | MUC1 |
|  |  |  | RTN3 |
|  |  |  | SKP2 |
|  |  |  | SLC2A3 |
|  |  |  | MAP3K14 |
|  |  |  | SLC40A1 |
|  |  |  | PTHLH |
|  |  |  | ARID3A |
|  |  |  | FEM1B |
|  |  |  | TNFRSF11B |
|  |  |  | C3AR1 |
|  |  |  | MIR27A |
|  |  |  | MALRD1 |
|  |  |  | CD36 |
|  |  |  | UBLCP1 |
|  |  |  | USP24 |
|  |  |  | MYH9 |
|  |  |  | LBP |
|  |  |  | MIR10A |
|  |  |  | LMF1 |
|  |  |  | C8orf48 |
|  |  |  | MIR215 |
|  |  |  | TNFSF15 |
|  |  |  | DUSP2 |
|  |  |  | NCF2 |
|  |  |  | BDKRB2 |
|  |  |  | IDH2 |
|  |  |  | MIR320A |
|  |  |  | HDGFL1 |
|  |  |  | PAG1 |
|  |  |  | MEF2A |
|  |  |  | TLR6 |
|  |  |  | C1QTNF5 |
|  |  |  | TNNC2 |
|  |  |  | APOL4 |
|  |  |  | HTN3 |
|  |  |  | CDKN2B-AS1 |
|  |  |  | ABO |
|  |  |  | STIM1 |
|  |  |  | KCNH2 |
|  |  |  | TRAF3 |
|  |  |  | CXCR2 |
|  |  |  | CAMK2D |
|  |  |  | F9 |
|  |  |  | LIPE |
|  |  |  | B4GALT1 |
|  |  |  | TMEM161B |
|  |  |  | RPS27A |
|  |  |  | ADCY5 |
|  |  |  | HMCN1 |
|  |  |  | MIR181B1 |
|  |  |  | TNNI3 |
|  |  |  | CD40 |
|  |  |  | LY86 |
|  |  |  | SGCB |
|  |  |  | IGFALS |
|  |  |  | E2F1 |
|  |  |  | PLCB3 |
|  |  |  | ARHGEF2 |
|  |  |  | APOB |
|  |  |  | CASP8 |
|  |  |  | CCM2 |
|  |  |  | CFH |
|  |  |  | PIGR |
|  |  |  | TSC2 |
|  |  |  | CYP11B2 |
|  |  |  | CHDS9 |
|  |  |  | MIR23A |
|  |  |  | ETS2 |
|  |  |  | CS |
|  |  |  | IGFBP7 |
|  |  |  | FCRL3 |
|  |  |  | SIRT4 |
|  |  |  | PRDX1 |
|  |  |  | FGB |
|  |  |  | CDH2 |
|  |  |  | ITGA1 |
|  |  |  | ECE2 |
|  |  |  | FLNC |
|  |  |  | ARF1 |
|  |  |  | ARID5B |
|  |  |  | HAL |
|  |  |  | MYOT |
|  |  |  | SGIP1 |
|  |  |  | SCD5 |
|  |  |  | TMEM248P1 |
|  |  |  | CAVIN1 |
|  |  |  | C4A |
|  |  |  | PPARG |
|  |  |  | AQP4 |
|  |  |  | MERTK |
|  |  |  | DSG2 |
|  |  |  | APLNR |
|  |  |  | HOXC-AS1 |
|  |  |  | ORMDL3 |
|  |  |  | JAG1 |
|  |  |  | TANC1 |
|  |  |  | TBXA2R |
|  |  |  | CHGA |
|  |  |  | ST2 |
|  |  |  | HSPA4 |
|  |  |  | PAPPA |
|  |  |  | DMPK |
|  |  |  | NEAT1 |
|  |  |  | RHOA |
|  |  |  | MIR494 |
|  |  |  | TRH |
|  |  |  | CCL7 |
|  |  |  | CALCA |
|  |  |  | PRKCD |
|  |  |  | VPS41 |
|  |  |  | LPXN |
|  |  |  | DSC2 |
|  |  |  | CHDS1 |
|  |  |  | CXCL11 |
|  |  |  | TMPRSS6 |
|  |  |  | SERPINA1 |
|  |  |  | TNNC1 |
|  |  |  | MIR92A1 |
|  |  |  | PPIG |
|  |  |  | SUN1 |
|  |  |  | LDAH |
|  |  |  | MAPT |
|  |  |  | PRKAA1 |
|  |  |  | FADS2 |
|  |  |  | IL1RL1 |
|  |  |  | SYNE4 |
|  |  |  | HLA-DQB1 |
|  |  |  | MT-CYB |
|  |  |  | GABRG1 |
|  |  |  | ZNF441 |
|  |  |  | AHCY |
|  |  |  | MTTP |
|  |  |  | CXCL8 |
|  |  |  | MAPK9 |
|  |  |  | EGR1 |
|  |  |  | CCL17 |
|  |  |  | THSD1 |
|  |  |  | KHK |
|  |  |  | RIPK2 |
|  |  |  | CHRNB4 |
|  |  |  | HDAC9 |
|  |  |  | GLP1R |
|  |  |  | OGG1 |
|  |  |  | PARP14 |
|  |  |  | DYM |
|  |  |  | RPL15P15 |
|  |  |  | RPL9P21 |
|  |  |  | TUBA1B |
|  |  |  | SELE |
|  |  |  | MSX2 |
|  |  |  | PSMC6 |
|  |  |  | CNR1 |
|  |  |  | PBRM1 |
|  |  |  | FBXO33 |
|  |  |  | CPB2 |
|  |  |  | LINC01535 |
|  |  |  | FGF19 |
|  |  |  | CTF1 |
|  |  |  | EGFL7 |
|  |  |  | PIK3R3 |
|  |  |  | STAP1 |
|  |  |  | KLF6 |
|  |  |  | SLC34A1 |
|  |  |  | THBD |
|  |  |  | NR3C1 |
|  |  |  | CYP4V2 |
|  |  |  | FCN2 |
|  |  |  | PIK3C2A |
|  |  |  | CORO1B |
|  |  |  | CHKB |
|  |  |  | SCUBE1 |
|  |  |  | F11 |
|  |  |  | CLEC4C |
|  |  |  | MAP2K6 |
|  |  |  | DCD |
|  |  |  | CD180 |
|  |  |  | EMP1 |
|  |  |  | IL17A |
|  |  |  | ATP2A1 |
|  |  |  | TMPO |
|  |  |  | COG2 |
|  |  |  | PNLIP |
|  |  |  | LEPR |
|  |  |  | MTOR |
|  |  |  | MIR124-1 |
|  |  |  | ABCA8 |
|  |  |  | PDE9A |
|  |  |  | PITPNA |
|  |  |  | NDUFS2 |
|  |  |  | STUB1 |
|  |  |  | GP6 |
|  |  |  | WT1 |
|  |  |  | ILK |
|  |  |  | LEPROT |
|  |  |  | MAPK14 |
|  |  |  | SERPINB2 |
|  |  |  | TXNRD2 |
|  |  |  | AOC3 |
|  |  |  | PITX2 |
|  |  |  | TNFRSF4 |
|  |  |  | MTHFD2 |
|  |  |  | GJA4 |
|  |  |  | MYBPC3 |
|  |  |  | FABP3 |
|  |  |  | TRPV2 |
|  |  |  | SLC27A1 |
|  |  |  | MYPN |
|  |  |  | CCN2 |
|  |  |  | HPR |
|  |  |  | ANXA6 |
|  |  |  | P2RY1 |
|  |  |  | TFPT |
|  |  |  | CSK |
|  |  |  | CTSS |
|  |  |  | MIR448 |
|  |  |  | SMPD1 |
|  |  |  | BHMT2 |
|  |  |  | RPL7 |
|  |  |  | TH |
|  |  |  | FBLN1 |
|  |  |  | GSTO2 |
|  |  |  | PRKAB2 |
|  |  |  | HSPB2 |
|  |  |  | RAF1 |
|  |  |  | MIR19B2 |
|  |  |  | STC1 |
|  |  |  | KLRK1 |
|  |  |  | FURIN |
|  |  |  | ADH7 |
|  |  |  | LTB4R2 |
|  |  |  | UNGP1 |
|  |  |  | ST8SIA1 |
|  |  |  | APOA5 |
|  |  |  | CCR2 |
|  |  |  | EDN3 |
|  |  |  | ANK2 |
|  |  |  | SPON1 |
|  |  |  | PLA2G4A |
|  |  |  | SF3A2 |
|  |  |  | VLDLR |
|  |  |  | VEGFC |
|  |  |  | GATAD1 |
|  |  |  | ITGB6 |
|  |  |  | PPP1R3B |
|  |  |  | SDC4 |
|  |  |  | LMOD1 |
|  |  |  | FHL2 |
|  |  |  | PDGFB |
|  |  |  | ELN |
|  |  |  | CALR |
|  |  |  | VIM |
|  |  |  | CADPS |
|  |  |  | ADRA1B |
|  |  |  | LTB |
|  |  |  | NUDT6 |
|  |  |  | TLR8 |
|  |  |  | KCNMB1 |
|  |  |  | DES |
|  |  |  | LGALS3BP |
|  |  |  | ITGAV |
|  |  |  | FGFR2 |
|  |  |  | XRCC3 |
|  |  |  | CSF3 |
|  |  |  | PCNA |
|  |  |  | MMACHC |
|  |  |  | RBM20 |
|  |  |  | B4GALT6 |
|  |  |  | PRKAR1A |
|  |  |  | ATG5 |
|  |  |  | HLA-A |
|  |  |  | SUPT3H |
|  |  |  | ADAMTS4 |
|  |  |  | TNNI2 |
|  |  |  | LDHA |
|  |  |  | EDNRB |
|  |  |  | CD27 |
|  |  |  | FAM167A |
|  |  |  | SCD |
|  |  |  | ARMH3 |
|  |  |  | MIR106B |
|  |  |  | FEM1A |
|  |  |  | HSPA6 |
|  |  |  | CHUK |
|  |  |  | CHAT |
|  |  |  | NR1H2 |
|  |  |  | SEC11B |
|  |  |  | CAPG |
|  |  |  | SPHK1 |
|  |  |  | ATN1 |
|  |  |  | MYH10 |
|  |  |  | ARSB |
|  |  |  | IL22 |
|  |  |  | ORAI1 |
|  |  |  | PARPBP |
|  |  |  | ADAM9 |
|  |  |  | TPH1 |
|  |  |  | STXBP5 |
|  |  |  | AMPD1 |
|  |  |  | TBXAS1 |
|  |  |  | MIR103A1 |
|  |  |  | MYH11 |
|  |  |  | IFNGR2 |
|  |  |  | AGK |
|  |  |  | CAP1 |
|  |  |  | KIF2C |
|  |  |  | DUSP1 |
|  |  |  | DDAH2 |
|  |  |  | FGF4 |
|  |  |  | NPR3 |
|  |  |  | SLPI |
|  |  |  | DMRTA1 |
|  |  |  | BCAR1 |
|  |  |  | ADAM10 |
|  |  |  | ATXN3 |
|  |  |  | PRPS1 |
|  |  |  | LIPA |
|  |  |  | P2RY2 |
|  |  |  | PLD5 |
|  |  |  | H19 |
|  |  |  | CAMLG |
|  |  |  | TREML4 |
|  |  |  | TGFBR1 |
|  |  |  | P2RY12 |
|  |  |  | GRK2 |
|  |  |  | CDC42 |
|  |  |  | ENSG00000269918 |
|  |  |  | VWF |
|  |  |  | VTN |
|  |  |  | TPM1 |
|  |  |  | MIR24-1 |
|  |  |  | CIDEC |
|  |  |  | EBF1 |
|  |  |  | NINJ2 |
|  |  |  | CYP2E1 |
|  |  |  | FLNB |
|  |  |  | WDFY4 |
|  |  |  | CTH |
|  |  |  | MFSD10 |
|  |  |  | IL15 |
|  |  |  | ATM |
|  |  |  | TIE1 |
|  |  |  | PLEKHA1 |
|  |  |  | TP53COR1 |
|  |  |  | ANGPTL8 |
|  |  |  | KCNK1 |
|  |  |  | C1orf210 |
|  |  |  | LIG4 |
|  |  |  | EREG |
|  |  |  | MIR30C2 |
|  |  |  | BMP4 |
|  |  |  | SFRP4 |
|  |  |  | AGTR1 |
|  |  |  | MCAM |
|  |  |  | IPMK |
|  |  |  | CAMP |
|  |  |  | OBSCN |
|  |  |  | ABCG4 |
|  |  |  | UGT1A4 |
|  |  |  | FCAMR |
|  |  |  | IL1RAP |
|  |  |  | MAT2B |
|  |  |  | SMILR |
|  |  |  | SRGN |
|  |  |  | PRDX5 |
|  |  |  | PPIA |
|  |  |  | CASP7 |
|  |  |  | MED6 |
|  |  |  | MCI2 |
|  |  |  | TRIB1 |
|  |  |  | PRKAG3 |
|  |  |  | TJP1 |
|  |  |  | EDEM2 |
|  |  |  | CD46 |
|  |  |  | PTX3 |
|  |  |  | ICOSLG |
|  |  |  | MIR128-1 |
|  |  |  | UGT1A6 |
|  |  |  | ANG |
|  |  |  | CCK |
|  |  |  | TRIM9 |
|  |  |  | SMAD4 |
|  |  |  | CSMD1 |
|  |  |  | RETNLB |
|  |  |  | CTSD |
|  |  |  | SGMS1 |
|  |  |  | STAT6 |
|  |  |  | JCAD |
|  |  |  | FKBP1B |
|  |  |  | TRERF1 |
|  |  |  | SCARB1 |
|  |  |  | IL3 |
|  |  |  | VAV2 |
|  |  |  | PTCH1 |
|  |  |  | TP53BP1 |
|  |  |  | S100A1 |
|  |  |  | PTPA |
|  |  |  | GREM1 |
|  |  |  | SUN2 |
|  |  |  | CYP4A11 |
|  |  |  | TES |
|  |  |  | PRKAG2 |
|  |  |  | AGXT2 |
|  |  |  | EPRS1 |
|  |  |  | MIR197 |
|  |  |  | ILF3 |
|  |  |  | C1QTNF9 |
|  |  |  | MT1X |
|  |  |  | KEAP1 |
|  |  |  | RFC4 |
|  |  |  | LARP6 |
|  |  |  | SERPINF2 |
|  |  |  | MIAT |
|  |  |  | FBN1 |
|  |  |  | SGCG |
|  |  |  | TYRO3 |
|  |  |  | S100A8 |
|  |  |  | HAVCR1 |
|  |  |  | SLC10A2 |
|  |  |  | KRI1 |
|  |  |  | LEMD3 |
|  |  |  | CHKA |
|  |  |  | CPQ |
|  |  |  | TNFRSF13C |
|  |  |  | AIF1 |
|  |  |  | MAP3K1 |
|  |  |  | PBK |
|  |  |  | SOAT1 |
|  |  |  | AZU1 |
|  |  |  | CTNNA3 |
|  |  |  | MT-ND1 |
|  |  |  | HDLC3 |
|  |  |  | F8 |
|  |  |  | MIR133A1 |
|  |  |  | NOMO3 |
|  |  |  | TNFRSF12A |
|  |  |  | RPL29P29 |
|  |  |  | EGR3 |
|  |  |  | SERPIND1 |
|  |  |  | IL12A |
|  |  |  | ARNTL |
|  |  |  | BMPER |
|  |  |  | CTCF |
|  |  |  | MTNR1A |
|  |  |  | PPP1R3A |
|  |  |  | MCL1 |
|  |  |  | CCL2 |
|  |  |  | SURF2 |
|  |  |  | RGS9BP |
|  |  |  | MIR125A |
|  |  |  | ACTN2 |
|  |  |  | TXNIP |
|  |  |  | ATG9B |
|  |  |  | SHMT1 |
|  |  |  | ADCY10 |
|  |  |  | CCND3 |
|  |  |  | M6PR |
|  |  |  | ADA |
|  |  |  | SPATA7 |
|  |  |  | DOT1L |
|  |  |  | MAZ |
|  |  |  | IL27 |
|  |  |  | EIF2AK3 |
|  |  |  | PDCD10 |
|  |  |  | KLK15 |
|  |  |  | CAMK2G |
|  |  |  | IL6 |
|  |  |  | TLR3 |
|  |  |  | FGF14 |
|  |  |  | ANXA5 |
|  |  |  | EPHX2 |
|  |  |  | SSNA1 |
|  |  |  | LPAR2 |
|  |  |  | CCL24 |
|  |  |  | PRICKLE2 |
|  |  |  | ANGPT1 |
|  |  |  | RAN |
|  |  |  | CPT2 |
|  |  |  | GPR26 |
|  |  |  | GLRA3 |
|  |  |  | ADPRS |
|  |  |  | POLR2D |
|  |  |  | STAG3 |
|  |  |  | EPHA1 |
|  |  |  | MIR15B |
|  |  |  | EPOR |
|  |  |  | MIR185 |
|  |  |  | HNRNPC |
|  |  |  | LTB4R |
|  |  |  | RPL35AP22 |
|  |  |  | CASP3 |
|  |  |  | HSP90AA1 |
|  |  |  | ANGPT2 |
|  |  |  | CACNA2D3 |
|  |  |  | DUOX1 |
|  |  |  | TSBP1 |
|  |  |  | RNF213 |
|  |  |  | ACSS2 |
|  |  |  | PLIN1 |
|  |  |  | SUCLG1 |
|  |  |  | PKN2 |
|  |  |  | ITGA4 |
|  |  |  | VDR |
|  |  |  | ACE |
|  |  |  | CLOCK |
|  |  |  | MLX |
|  |  |  | NPY5R |
|  |  |  | PRKCE |
|  |  |  | ITGAM |
|  |  |  | ROCK1 |
|  |  |  | BLOC1S1 |
|  |  |  | CD34 |
|  |  |  | DSPP |
|  |  |  | BCO1 |
|  |  |  | RGS2 |
|  |  |  | CHDS2 |
|  |  |  | TAGLN |
|  |  |  | TPSAB1 |
|  |  |  | PDPN |
|  |  |  | MIR423 |
|  |  |  | DLL3 |
|  |  |  | PPT2 |
|  |  |  | FASLG |
|  |  |  | RTN4 |
|  |  |  | COL3A1 |
|  |  |  | ENG |
|  |  |  | MIR126 |
|  |  |  | LIPM |
|  |  |  | LIPF |
|  |  |  | RXRG |
|  |  |  | CACNA1D |
|  |  |  | PRKAA2 |
|  |  |  | UHRF1BP1 |
|  |  |  | CD163 |
|  |  |  | MIR125B1 |
|  |  |  | DUSP19 |
|  |  |  | KCNK2 |
|  |  |  | RGS5 |
|  |  |  | SORCS1 |
|  |  |  | TBC1D4 |
|  |  |  | DNM3 |
|  |  |  | RLN2 |
|  |  |  | BBS2 |
|  |  |  | IL1RAPL2 |
|  |  |  | TUBA4A |
|  |  |  | HPD |
|  |  |  | MYLIP |
|  |  |  | SPAG17 |
|  |  |  | HNF1A |
|  |  |  | EPHA3 |
|  |  |  | LMAN2 |
|  |  |  | MFAP4 |
|  |  |  | INSIG2 |
|  |  |  | TYROBP |
|  |  |  | DPRXP7 |
|  |  |  | RARA |
|  |  |  | B3GALT4 |
|  |  |  | OSCAR |
|  |  |  | PECAM1 |
|  |  |  | SFRP5 |
|  |  |  | MIR205 |
|  |  |  | MYD88 |
|  |  |  | TNC |
|  |  |  | PTPN1 |
|  |  |  | DLG2 |
|  |  |  | PML |
|  |  |  | BRAP |
|  |  |  | CCR8 |
|  |  |  | MYH14 |
|  |  |  | PPP1R12B |
|  |  |  | DAB2 |
|  |  |  | TBX4 |
|  |  |  | MIR636 |
|  |  |  | MARCKS |
|  |  |  | LRP6 |
|  |  |  | MIR145 |
|  |  |  | SERPINH1 |
|  |  |  | PPARA |
|  |  |  | CNR2 |
|  |  |  | KRIT1 |
|  |  |  | MIR30B |
|  |  |  | MGLL |
|  |  |  | RORA |
|  |  |  | PTPRD |
|  |  |  | ALOX15B |
|  |  |  | CDK9 |
|  |  |  | IL2RA |
|  |  |  | CD59 |
|  |  |  | PLCG2 |
|  |  |  | FAP |
|  |  |  | KCNJ1 |
|  |  |  | LRRTM4 |
|  |  |  | CCR1 |
|  |  |  | CLDN5 |
|  |  |  | ITGA2B |
|  |  |  | MAOB |
|  |  |  | PTPRN2 |
|  |  |  | PTGDS |
|  |  |  | PSAP |
|  |  |  | UGT1A9 |
|  |  |  | CRYAA |
|  |  |  | ATP1A2 |
|  |  |  | COPS5 |
|  |  |  | TNFSF4 |
|  |  |  | TPM4 |
|  |  |  | BCL2L11 |
|  |  |  | HERPUD1 |
|  |  |  | DUSP6 |
|  |  |  | TGFB3 |
|  |  |  | ELK1 |
|  |  |  | HGD |
|  |  |  | INHBA |
|  |  |  | BMPR2 |
|  |  |  | RBP4 |
|  |  |  | LOC100506178 |
|  |  |  | NME2 |
|  |  |  | ADAM33 |
|  |  |  | ZNF491 |
|  |  |  | S100A4 |
|  |  |  | BTC |
|  |  |  | MSH5 |
|  |  |  | LGMN |
|  |  |  | TCN1 |
|  |  |  | LEPQTL1 |
|  |  |  | CPEB2 |
|  |  |  | IGF2 |
|  |  |  | LILRB1 |
|  |  |  | CTSG |
|  |  |  | ADM2 |
|  |  |  | LINC02577 |
|  |  |  | CD80 |
|  |  |  | NUCB2 |
|  |  |  | RARRES2 |
|  |  |  | FOXO4 |
|  |  |  | ADPRH |
|  |  |  | DNMT1 |
|  |  |  | NOD1 |
|  |  |  | JMJD6 |
|  |  |  | KIF6 |
|  |  |  | ADAMTS1 |
|  |  |  | MIR486-1 |
|  |  |  | PRKG1 |
|  |  |  | CFI |
|  |  |  | SBF2 |
|  |  |  | CDH9 |
|  |  |  | ADRA2B |
|  |  |  | SUV39H1 |
|  |  |  | PLA2G5 |
|  |  |  | AAMP |
|  |  |  | CYP20A1 |
|  |  |  | RNASE3 |
|  |  |  | PIK3CG |
|  |  |  | ACKR1 |
|  |  |  | LPA |
|  |  |  | COL4A4 |
|  |  |  | FGF7 |
|  |  |  | IL37 |
|  |  |  | NPC1L1 |
|  |  |  | UTS2 |
|  |  |  | TAT |
|  |  |  | RREB1 |
|  |  |  | MB |
|  |  |  | C12orf43 |
|  |  |  | MIR92A2 |
|  |  |  | LIFR |
|  |  |  | NQO1 |
|  |  |  | CBL |
|  |  |  | SP1 |
|  |  |  | APOBEC1 |
|  |  |  | CACNG8 |
|  |  |  | SURF1 |
|  |  |  | MIR23B |
|  |  |  | CD79A |
|  |  |  | S1PR1 |
|  |  |  | IL1R2 |
|  |  |  | PIK3CA |
|  |  |  | SGCA |
|  |  |  | MAGI2 |
|  |  |  | LARGE1 |
|  |  |  | IRGM |
|  |  |  | CKLF |
|  |  |  | TSPO |
|  |  |  | SRIP1 |
|  |  |  | UCP1 |
|  |  |  | DAB2IP |
|  |  |  | FGF9 |
|  |  |  | IL2 |
|  |  |  | FADD |
|  |  |  | ZNF844 |
|  |  |  | SDC1 |
|  |  |  | FGA |
|  |  |  | SERPINB1 |
|  |  |  | IGFBP1 |
|  |  |  | SLC1A1 |
|  |  |  | SLC1A4 |
|  |  |  | DDIT3 |
|  |  |  | HOMER1 |
|  |  |  | PLA2G15 |
|  |  |  | CDH1 |
|  |  |  | PTGR1 |
|  |  |  | CALU |
|  |  |  | PNPLA5 |
|  |  |  | TBC1D1 |
|  |  |  | PLAG1 |
|  |  |  | FOS |
|  |  |  | IFNG |
|  |  |  | AGPAT2 |
|  |  |  | CREG1 |
|  |  |  | EMD |
|  |  |  | HSPA5 |
|  |  |  | MIR616 |
|  |  |  | FNDC5 |
|  |  |  | RETN |
|  |  |  | CCL8 |
|  |  |  | CYP27A1 |
|  |  |  | PAFAH2 |
|  |  |  | ALDH8A1 |
|  |  |  | IGES |
|  |  |  | CCDC80 |
|  |  |  | SSH1 |
|  |  |  | MIR135B |
|  |  |  | RHOB |
|  |  |  | H6PD |
|  |  |  | GNAQ |
|  |  |  | CAVIN4 |
|  |  |  | XPA |
|  |  |  | MIR142 |
|  |  |  | FADS3 |
|  |  |  | PEX3 |
|  |  |  | FGF2 |
|  |  |  | CLU |
|  |  |  | AMH |
|  |  |  | PRODH |
|  |  |  | IRF5 |
|  |  |  | ACAT2 |
|  |  |  | CXCL12 |
|  |  |  | TRPM1 |
|  |  |  | NTN1 |
|  |  |  | IL36A |
|  |  |  | TET2 |
|  |  |  | COL2A1 |
|  |  |  | IDI1 |
|  |  |  | LHFPL1 |
|  |  |  | RPA1 |
|  |  |  | EEF1E1 |
|  |  |  | FCGR2C |
|  |  |  | CA10 |
|  |  |  | RRM2 |
|  |  |  | HK2 |
|  |  |  | MDK |
|  |  |  | MRE11 |
|  |  |  | AKAP9 |
|  |  |  | METRNL |
|  |  |  | EPHX1 |
|  |  |  | CD63 |
|  |  |  | SLC20A1 |
|  |  |  | CX3CL1 |
|  |  |  | AIMP1 |
|  |  |  | LDB2 |
|  |  |  | FEN1 |
|  |  |  | PPIF |
|  |  |  | SPTB |
|  |  |  | MIR124-3 |
|  |  |  | EFEMP2 |
|  |  |  | ICAM1 |
|  |  |  | CXCL9 |
|  |  |  | RECK |
|  |  |  | CAV1 |
|  |  |  | GNPDA2 |
|  |  |  | H3-2 |
|  |  |  | DGAT2 |
|  |  |  | BCL2L1 |
|  |  |  | DNAH9 |
|  |  |  | RAPGEF3 |
|  |  |  | HELLS |
|  |  |  | TNFRSF1B |
|  |  |  | LGALS9 |
|  |  |  | MIR495 |
|  |  |  | MAPK3 |
|  |  |  | SLC20A2 |
|  |  |  | AEBP1 |
|  |  |  | PAQR5 |
|  |  |  | SLC16A3 |
|  |  |  | ANKRD2 |
|  |  |  | HMGCL |
|  |  |  | SLC9A1 |
|  |  |  | SLC12A5-AS1 |
|  |  |  | TNNI3K |
|  |  |  | CACNA1S |
|  |  |  | MSI2 |
|  |  |  | CORIN |
|  |  |  | PRR7 |
|  |  |  | MRTFA |
|  |  |  | SPARCL1 |
|  |  |  | NEXN |
|  |  |  | CTSL |
|  |  |  | THBS2 |
|  |  |  | PLEC |
|  |  |  | IL6ST |
|  |  |  | LTF |
|  |  |  | TPO |
|  |  |  | ANK1 |
|  |  |  | ETS1 |
|  |  |  | MLST8 |
|  |  |  | AGRP |
|  |  |  | GATA5 |
|  |  |  | MIR181A1 |
|  |  |  | HSP90B1 |
|  |  |  | APCS |
|  |  |  | CXCL1 |
|  |  |  | MIR92B |
|  |  |  | ABCB4 |
|  |  |  | MIR100 |
|  |  |  | SGMS2 |
|  |  |  | MXD3 |
|  |  |  | FAH |
|  |  |  | SFTPB |
|  |  |  | VAMP3 |
|  |  |  | RCE1 |
|  |  |  | HNF4A |
|  |  |  | CNTNAP2 |
|  |  |  | MYH7 |
|  |  |  | TNFRSF10B |
|  |  |  | lnc-PIK3CG-6 |
|  |  |  | SH2B3 |
|  |  |  | TCIRG1 |
|  |  |  | LOC110973015 |
|  |  |  | MIR503 |
|  |  |  | RELA |
|  |  |  | HIF1A |
|  |  |  | TRAF1 |
|  |  |  | RYR2 |
|  |  |  | IRF1-AS1 |
|  |  |  | GRN |
|  |  |  | DBN1 |
|  |  |  | MIR133B |
|  |  |  | SLC2A4 |
|  |  |  | AP3D1 |
|  |  |  | PTS |
|  |  |  | KCNK13 |
|  |  |  | MAP2 |
|  |  |  | ADCY6 |
|  |  |  | TNFRSF11A |
|  |  |  | NOS3 |
|  |  |  | TGFA |
|  |  |  | NPR2 |
|  |  |  | MMP17 |
|  |  |  | ANPEP |
|  |  |  | LOC100507053 |
|  |  |  | PROM1 |
|  |  |  | MIR10B |
|  |  |  | PAOD1 |
|  |  |  | CFTR |
|  |  |  | TUSC1 |
|  |  |  | GSTM3 |
|  |  |  | KLF14 |
|  |  |  | PTPRA |
|  |  |  | TBX20 |
|  |  |  | KCNA5 |
|  |  |  | FLRT2 |
|  |  |  | HSF1 |
|  |  |  | WRN |
|  |  |  | NFATC2 |
|  |  |  | NOMO2 |
|  |  |  | NPPC |
|  |  |  | CYLD |
|  |  |  | ZEB2 |
|  |  |  | TBCAP1 |
|  |  |  | RHBDF2 |
|  |  |  | ABCG5 |
|  |  |  | MIR16-1 |
|  |  |  | USF1 |
|  |  |  | JMJD1C |
|  |  |  | NFKBIB |
|  |  |  | CHRM2 |
|  |  |  | DKK1 |
|  |  |  | CAPN5 |
|  |  |  | CYCSP14 |
|  |  |  | KCNH5 |
|  |  |  | NR1H4 |
|  |  |  | RRM1 |
|  |  |  | ATP6V1G2-DDX39B |
|  |  |  | OLFML2B |
|  |  |  | ERCC6 |
|  |  |  | NOS1AP |
|  |  |  | PRMT3 |
|  |  |  | NOTCH4 |
|  |  |  | INPP5D |
|  |  |  | CLEC12A |
|  |  |  | CDKN1C |
|  |  |  | ABCA4 |
|  |  |  | SIRT3 |
|  |  |  | AQP1 |
|  |  |  | OPRM1 |
|  |  |  | GFAP |
|  |  |  | DPP8 |
|  |  |  | CYP26B1 |
|  |  |  | ACTB |
|  |  |  | IL7R |
|  |  |  | MSRA |
|  |  |  | IFI35 |
|  |  |  | TLR4 |
|  |  |  | RGS9 |
|  |  |  | LECT2 |
|  |  |  | SRSF2 |
|  |  |  | BSCL2 |
|  |  |  | PCDH9 |
|  |  |  | CCL21 |
|  |  |  | ADAMTS13 |
|  |  |  | IL16 |
|  |  |  | MSTN |
|  |  |  | VCL |
|  |  |  | UBA52P6 |
|  |  |  | RPL31P26 |
|  |  |  | SERPINE1 |
|  |  |  | HFE |
|  |  |  | POU2F3 |
|  |  |  | CYP2C8 |
|  |  |  | BACH1 |
|  |  |  | MYO7A |
|  |  |  | MIR17 |
|  |  |  | VCAM1 |
|  |  |  | MIR19B1 |
|  |  |  | PTAFR |
|  |  |  | ACTG2 |
|  |  |  | ROBO2 |
|  |  |  | NOX1 |
|  |  |  | IFNGR1 |
|  |  |  | CSN1S1 |
|  |  |  | SRFBP1 |
|  |  |  | TSPAN6 |
|  |  |  | PRELID1 |
|  |  |  | RSAD2 |
|  |  |  | TGFBR2 |
|  |  |  | CDH23 |
|  |  |  | TLR9 |
|  |  |  | TTN |
|  |  |  | IL33 |
|  |  |  | RGS6 |
|  |  |  | ITGA11 |
|  |  |  | ZNF700 |
|  |  |  | WWTR1 |
|  |  |  | IFNA1 |
|  |  |  | HHIP |
|  |  |  | HLA-DRB1 |
|  |  |  | MEG3 |
|  |  |  | FGF3 |
|  |  |  | RAD51 |
|  |  |  | CLEC16A |
|  |  |  | ADH1B |
|  |  |  | DYRK1A |
|  |  |  | MIR146B |
|  |  |  | PEMT |
|  |  |  | SARDH |
|  |  |  | NFKBIE |
|  |  |  | ANGPTL2 |
|  |  |  | NIT2 |
|  |  |  | NOG |
|  |  |  | HBS1L |
|  |  |  | HLA-B |
|  |  |  | ATP5F1B |
|  |  |  | TCF7L2 |
|  |  |  | PKM |
|  |  |  | HSPA1B |
|  |  |  | HEY2 |
|  |  |  | GCG |
|  |  |  | piR-43107-137 |
|  |  |  | GPD2 |
|  |  |  | CXCL5 |
|  |  |  | SPRR3 |
|  |  |  | GSTO1 |
|  |  |  | DAOA |
|  |  |  | ENPP1 |
|  |  |  | SCNN1A |
|  |  |  | FABP4 |
|  |  |  | UNC13C |
|  |  |  | KCNE2 |
|  |  |  | RHOBTB3 |
|  |  |  | P2RX4 |
|  |  |  | CCND2 |
|  |  |  | CFB |
|  |  |  | PDZK1 |
|  |  |  | DDX58 |
|  |  |  | ADIPOR2 |
|  |  |  | UBIAD1 |
|  |  |  | FCN3 |
|  |  |  | PTPN3 |
|  |  |  | ABCA7 |
|  |  |  | TMEM258 |
|  |  |  | RPS27P1 |
|  |  |  | CES5A |
|  |  |  | ACTA2-AS1 |
|  |  |  | MIR362 |
|  |  |  | CAPN10 |
|  |  |  | CCR5 |
|  |  |  | MIR130B |
|  |  |  | SLC5A1 |
|  |  |  | FABP12 |
|  |  |  | PDLIM3 |
|  |  |  | ZNHIT3 |
|  |  |  | VEGFD |
|  |  |  | ZFHX3 |
|  |  |  | PCSK5 |
|  |  |  | GIMAP5 |
|  |  |  | NDUFA9 |
|  |  |  | CYP2B6 |
|  |  |  | FDXR |
|  |  |  | NES |
|  |  |  | PITX3 |
|  |  |  | PNLIPRP1 |
|  |  |  | EYA4 |
|  |  |  | PRKAB1 |
|  |  |  | PON2 |
|  |  |  | GPX3 |
|  |  |  | EDN2 |
|  |  |  | YAP1 |
|  |  |  | HPX |
|  |  |  | MALAT1 |
|  |  |  | KLK1 |
|  |  |  | L3MBTL3 |
|  |  |  | CLEC4A |
|  |  |  | MIF |
|  |  |  | MLXIPL |
|  |  |  | PIP5KL1 |
|  |  |  | C4BPB |
|  |  |  | MAP2K1 |
|  |  |  | GH1 |
|  |  |  | PFN1 |
|  |  |  | AIM2 |
|  |  |  | ARL15 |
|  |  |  | HPS5 |
|  |  |  | ANK3 |
|  |  |  | BCL3 |
|  |  |  | TIMP2 |
|  |  |  | CCN3 |
|  |  |  | ITGB3 |
|  |  |  | PKD2L1 |
|  |  |  | F2RL3 |
|  |  |  | BMPR1B |
|  |  |  | CLIP1 |
|  |  |  | CCL15 |
|  |  |  | GP5 |
|  |  |  | AAT2 |
|  |  |  | LTC4S |
|  |  |  | SEMA5A |
|  |  |  | P4HB |
|  |  |  | MIR217 |
|  |  |  | STC2 |
|  |  |  | TNXA |
|  |  |  | TLL1 |
|  |  |  | TMEM132B |
|  |  |  | COL6A1 |
|  |  |  | ZNF788P |
|  |  |  | OAZ1 |
|  |  |  | IL11 |
|  |  |  | NUMA1 |
|  |  |  | F12 |
|  |  |  | TCF7 |
|  |  |  | F11R |
|  |  |  | DTWD1 |
|  |  |  | HSD11B1 |
|  |  |  | KITLG |
|  |  |  | FOXD4 |
|  |  |  | MMRN1 |
|  |  |  | GSK3A |
|  |  |  | LCN2 |
|  |  |  | PI16 |
|  |  |  | SUMO4 |
|  |  |  | TXN |
|  |  |  | SPARC |
|  |  |  | ZNF326 |
|  |  |  | CTSH |
|  |  |  | COL5A1 |
|  |  |  | ACTA2 |
|  |  |  | BECN1 |
|  |  |  | TRPC4AP |
|  |  |  | ANKRD1 |
|  |  |  | IL20RB |
|  |  |  | SAA2 |
|  |  |  | ZNF592 |
|  |  |  | IFN1@ |
|  |  |  | LAMA4 |
|  |  |  | PTGS1 |
|  |  |  | RNF157 |
|  |  |  | SERPINC1 |
|  |  |  | NPS |
|  |  |  | CFP |
|  |  |  | F3 |
|  |  |  | MIR133A2 |
|  |  |  | PLEKHM2 |
|  |  |  | NR1I2 |
|  |  |  | CES4A |
|  |  |  | IGFBP4 |
|  |  |  | KCNJ5 |
|  |  |  | LMNA |
|  |  |  | NDUFA10 |
|  |  |  | CASQ2 |
|  |  |  | EIF3FP3 |
|  |  |  | APOA1 |
|  |  |  | PRICKLE1 |
|  |  |  | CCDC92 |
|  |  |  | KLF10 |
|  |  |  | FOXO3 |
|  |  |  | CD69 |
|  |  |  | LTA |
|  |  |  | TTPA |
|  |  |  | MYOZ1 |
|  |  |  | IFNB1 |
|  |  |  | PSEN1 |
|  |  |  | HULC |
|  |  |  | MIR499A |
|  |  |  | CYP46A1 |
|  |  |  | RAET1E |
|  |  |  | SLC2A9 |
|  |  |  | CDKN2A |
|  |  |  | PHOSPHO1 |
|  |  |  | LPAR1 |
|  |  |  | ETV6 |
|  |  |  | PMM2 |
|  |  |  | SCN2B |
|  |  |  | CEBPB |
|  |  |  | HAS1 |
|  |  |  | FMNL2 |
|  |  |  | TRPS1 |
|  |  |  | HMGA1 |
|  |  |  | RPS26P57 |
|  |  |  | CCL3 |
|  |  |  | lnc-SPC24-1 |
|  |  |  | TFPI2 |
|  |  |  | ATHS |
|  |  |  | STAG3L4 |
|  |  |  | ATG16L1 |
|  |  |  | ERN1 |
|  |  |  | CSF1R |
|  |  |  | JUN |
|  |  |  | TRA |
|  |  |  | IRAK4 |
|  |  |  | MYL2 |
|  |  |  | STK38 |
|  |  |  | ADAMTS5 |
|  |  |  | NOTCH3 |
|  |  |  | ATP2A2 |
|  |  |  | MYOM3 |
|  |  |  | ATIC |
|  |  |  | CXCL16 |
|  |  |  | NF2 |
|  |  |  | NRG1 |
|  |  |  | TREX1 |
|  |  |  | TNFSF14 |
|  |  |  | FBXO32 |
|  |  |  | TGFBI |
|  |  |  | AR |
|  |  |  | MYH7B |
|  |  |  | KCNN4 |
|  |  |  | LEXM |
|  |  |  | GATM |
|  |  |  | MHRT |
|  |  |  | CDKN3 |
|  |  |  | CDH4 |
|  |  |  | ARHGEF10 |
|  |  |  | JPH3 |
|  |  |  | CYP2J2 |
|  |  |  | MIR30C1 |
|  |  |  | HDC |
|  |  |  | PXK |
|  |  |  | HSPG2 |
|  |  |  | FLNA |
|  |  |  | HEYL |
|  |  |  | IL2RB |
|  |  |  | ADCY9 |
|  |  |  | NOTCH1 |
|  |  |  | BAX |
|  |  |  | CD274 |
|  |  |  | LRPAP1 |
|  |  |  | CP |
|  |  |  | IGHE |
|  |  |  | TM4SF5 |
|  |  |  | PDYN |
|  |  |  | CDKAL1 |
|  |  |  | GORASP1 |
|  |  |  | CDH5 |
|  |  |  | FKBP1A |
|  |  |  | PINX1 |
|  |  |  | CYP24A1 |
|  |  |  | SCML4 |
|  |  |  | EIF3G |
|  |  |  | ANO6 |
|  |  |  | SMARCAL1 |
|  |  |  | FOSL1 |
|  |  |  | GSTT1 |
|  |  |  | NPC1 |
|  |  |  | PRKCA |
|  |  |  | UGT1A3 |
|  |  |  | TRIB2 |
|  |  |  | PRMT7 |
|  |  |  | ALDH9A1 |
|  |  |  | CD40LG |
|  |  |  | AHSP |
|  |  |  | UGT1A5 |
|  |  |  | DNAH5 |
|  |  |  | SRF |
|  |  |  | GHSR |
|  |  |  | MAT2A |
|  |  |  | TUBB4A |
|  |  |  | TRPC3 |
|  |  |  | TMEM106B |
|  |  |  | KLRD1 |
|  |  |  | FMN2 |
|  |  |  | NPTXR |
|  |  |  | IL7 |
|  |  |  | SEMA3C |
|  |  |  | SCN9A |
|  |  |  | SCN5A |
|  |  |  | UBE2Q2P1 |
|  |  |  | GPC1 |
|  |  |  | ZNF383 |
|  |  |  | HOTTIP |
|  |  |  | PDGFC |
|  |  |  | PEPD |
|  |  |  | GLS2 |
|  |  |  | GATA4 |
|  |  |  | ATP2B1 |
|  |  |  | VHL |
|  |  |  | PRKAG1 |
|  |  |  | SMAD5 |
|  |  |  | IGF2BP1 |
|  |  |  | MIR212 |
|  |  |  | ANKRD23 |
|  |  |  | FGF23 |
|  |  |  | PRSS1 |
|  |  |  | PNPLA2 |
|  |  |  | HLX |
|  |  |  | PTGIS |
|  |  |  | PAH |
|  |  |  | CEACAM3 |
|  |  |  | MIR216A |
|  |  |  | SNX17 |
|  |  |  | HDAC6 |
|  |  |  | MIR33B |
|  |  |  | CYP2R1 |
|  |  |  | FBLN5 |
|  |  |  | MIR490 |
|  |  |  | TNNT1 |
|  |  |  | S100A9 |
|  |  |  | CSF2 |
|  |  |  | ITPR3 |
|  |  |  | BIRC3 |
|  |  |  | RPS6KB1 |
|  |  |  | GPLD1 |
|  |  |  | DNAJC19 |
|  |  |  | RNLS |
|  |  |  | SRY |
|  |  |  | HBA1 |
|  |  |  | DCAF8 |
|  |  |  | AQP7 |
|  |  |  | ADIPOQ |
|  |  |  | RXRB |
|  | herbal strategies | 54 | HSD17B1 |
|  |  |  | HSD17B2 |
|  |  |  | FGR |
|  |  |  | EED |
|  |  |  | CES2 |
|  |  |  | HTR7 |
|  |  |  | PREP |
|  |  |  | AURKA |
|  |  |  | NEK6 |
|  |  |  | AURKB |
|  |  |  | NUAK1 |
|  |  |  | CAMK2B |
|  |  |  | TNKS2 |
|  |  |  | CA9 |
|  |  |  | SQLE |
|  |  |  | CA5A |
|  |  |  | CDK4 |
|  |  |  | HTR5A |
|  |  |  | CA12 |
|  |  |  | CA4 |
|  |  |  | PLA2G1B |
|  |  |  | DRD4 |
|  |  |  | SUZ12 |
|  |  |  | AKR1C1 |
|  |  |  | BRAF |
|  |  |  | EPHB4 |
|  |  |  | NEK2 |
|  |  |  | DRD1 |
|  |  |  | CCNA1 |
|  |  |  | CA13 |
|  |  |  | AKR1B10 |
|  |  |  | CA14 |
|  |  |  | PLK1 |
|  |  |  | ALK |
|  |  |  | CCNB3 |
|  |  |  | AKR1C2 |
|  |  |  | POLA1 |
|  |  |  | MAP3K8 |
|  |  |  | AVPR2 |
|  |  |  | PLK4 |
|  |  |  | CA7 |
|  |  |  | KDM4E |
|  |  |  | HTR2B |
|  |  |  | BACE1 |
|  |  |  | LYN |
|  |  |  | PYGL |
|  |  |  | FLT3 |
|  |  |  | HTR6 |
|  |  |  | CDK5R1 |
|  |  |  | PKN1 |
|  |  |  | AKR1C3 |
|  |  |  | CA6 |
|  |  |  | CBR1 |
|  |  |  | TOP1 |
|  | familiar remedies | 11 | SLC22A2 |
|  |  |  | CYP2D6 |
|  |  |  | SLCO2B1 |
|  |  |  | SLC22A6 |
|  |  |  | SLC22A7 |
|  |  |  | SLC16A1 |
|  |  |  | SLCO1B3 |
|  |  |  | SLC22A8 |
|  |  |  | SLCO1A2 |
|  |  |  | HTR1B |
|  |  |  | CYP3A7 |
